# Sociodemographic variation and childhood predictors of showing love and care for others in 22 countries

**DOI:** 10.1038/s41598-025-31380-9

**Published:** 2025-12-26

**Authors:** Matthew T. Lee, Renae Wilkinson, Katelyn N. G. Long, Brendan W. Case, James L. Ritchie-Dunham, Matt Bradshaw, R. Noah Padgett, Byron R. Johnson, Tyler J. VanderWeele

**Affiliations:** 1 Institute for Studies of Religion, Baylor University, Waco, TX USA; 2 Human Flourishing Program, Institute for Quantitative Social Science, Harvard University, Cambridge, MA USA; 3 Rosenthal Department of Management, McCombs School of Business, University of Texas, Austin, TX USA; 4 School of Public Policy, Pepperdine University, Malibu, CA USA; 5 Department of Epidemiology, Harvard T.H. Chan School of Public Health, Boston, MA USA; 6 Department of Biostatistics, Boston University School of Public Health, Boston, MA USA

**Keywords:** Love, Care, Childhood experiences, Global Flourishing Study, Cross-national, Well-being

## Abstract

Showing love and care for other people is a vital aspect of human relationships. However, little is known about how levels of love/care expression differ across cultures and across sociodemographic groups within those different cultures, or about the potential childhood antecedents that are associated with love/care expression in adulthood. Based on nationally-representative data from 22 countries in six continents in the Global Flourishing Study (N = 202,898), we present ordered means of love/care expression across countries, observe its distributions across key sociodemographic characteristics, and evaluate the strength of its childhood predictors (with *E*-values as a robustness check), revealing the extent to which the distributions and associations are uniform throughout the world or differ by country. The mean levels of love/care expression (scaled 0–10) ranged from 9.05 in the Philippines to 5.96 in Japan and tended to be higher in countries in the Global South. Based on a random effects meta-analysis of means and a multivariate regression analysis of childhood predictors, we find evidence of both universal and country-specific influences. Age cohort, gender, self-rated health, and religious service attendance were the strongest childhood predictors. These findings enhance our understanding of the country-specific variations in love/care expression and early-life predictors of adult outcomes, providing a foundation for future investigations into sociocultural influences that might shape love/care expression.

**Supplementary Information:**

The online version contains supplementary material available at 10.1038/s41598-025-31380-9.

## Introduction

Love and care are desired ends over the life course, across relationship types, and throughout the world^1–10^. Indeed, love and care appear to be “pan-cultural universal(s)”^11,12^. More specifically, the act of showing generous love to others—sometimes designated as “loving care”^13,14^, “caring love”^15^, or “loving-kindness”^16^—is a principal source of happiness, and often the highest virtue or value, in a variety of religious and philosophical traditions^17–22^. Prior research has revealed strong associations between the frequency of showing various types of love and care for other people and a variety of well-being outcomes such as physical health, social and emotional well-being, virtue, and financial security that, taken together, comprise some of the core elements of human flourishing^3,18,23,24^.

While there have been efforts to understand love and care across contexts^9^, less is known about how levels of love/care *expression* differ across cultures and across sociodemographic groups within those different cultures. Even less is known about the potential childhood antecedents that are associated with love/care expression in adulthood across cultures. To address these gaps, we use nationally-representative data from 22 countries (*N* = 202,898) in the Global Flourishing Study (GFS)^25–28^ to present an in-depth, cross-national exploration of the frequency of showing love/care for others across cultures, its variations across key sociodemographic groups, and its childhood antecedents. This paper is part of a coordinated effort to understand such variations and antecedents across a large number of outcomes by using a common methodology in all studies in the collection, in an effort to enhance the ability to compare findings. Our primary aim is to better understand the extent to which the distributions and associations are similar throughout the world or reflect country-specific sociocultural influences. This reflects an epidemiological goal of revealing distributions of outcomes across groups, as well as associations with predictors and covariates, to encourage future research on hypotheses about causal mechanisms to explain these patterns. After presenting the main findings, we offer speculative post hoc analyses to suggest candidate variables for such future work.

### Conceptualizing and assessing cross-national variations in love/care expression

In everyday language, the word *love* is often associated with powerful feelings of attachment or attraction^29^ and *care* tends to signify focused attention on a person^12^ and a “deliberate response that empowers and enables^30^.” But both words are also used to indicate the promotion of the well-being of the beloved^5,12^. Love and/or care involves either one or both of two phenomena: “(i) a disposition towards either desiring a perceived good or desiring union with it, either as an end itself or with it being a source of delight in itself or (ii) a disposition towards desiring good for a particular object for its own sake^31^.” The former might be labeled as “unitive love” and the latter as “contributory love^31^.” In the realm of interpersonal relations, which is the focus of our paper, the expression of love or care for another person might involve showing affection to them or nurturing their growth and flourishing (i.e., contributory love), as well as various forms of positive involvement in their life, the formation of healthy social bonds, and co-presence during both good times and bad (i.e., unitive love). Such expressions contain an element of volition—the will to act in a loving/caring manner—that enhances relational resonance and strengthens attachments among people. On this view, spending quality time with the beloved, showing them compassion when they are suffering, listening to their joys and sorrows, and helping them to grow in virtue all qualify as expressions of loving care^13^.

The words *love* and *care* have distinct meanings for many English-speakers, and we expect similar cultural variations in meaning across the terms used to render “love or care” in the 46 languages into which the Global Flourishing Study was translated. The English “love” likely has different connotations than the Hindi “*pyaar*,” the Hebrew “*‘ahav*,” or the Spanish “*amor*.” We do not aim to measure the many varieties, or “flavors,” of love and care^15^. Instead, we use a validated survey item^32^ that reflects the heart of love or care (or loving care) as expressed in many of the world’s wisdom traditions: desire for the good of the other in both unitive and contributory senses.

Cross-cultural research affirms the utility of studying loving care as distinct from other types of interpersonal love^15^. In many contexts, love is frequently expressed as “embodied care” and other forms of caring, while some groups prefer to “speak of care”^33^ rather than “love,” possibly because love is often associated with romantic relationships. For example, the word “care” might be more effective to use in a business setting^34^. However, “care” by itself may not fully capture the “loving” aspect of “loving care” and the word “love” is also used in some businesses^35^. People can be paid to provide “care” without really loving the other. We therefore believe that it is useful to include both love and care in a single survey item in order to capture the core meaning of “loving care.”

A large body of research has revealed the profound ways that culture shapes the expression of love, including the very different meanings of the word “love” itself, the extent to which different sociodemographic groups are expected to engage in such expressions, and the relative strength of social institutions that foster love (e.g., family, religion)^9^. Most cross-cultural research seems to have concentrated on love as a positive feeling, and survey data suggests that this facet of love is prominent in societies characterized by high relational mobility: the ability to use personal preferences to form new relationships^11^. When relational mobility is high, friendship and romantic forms of love are particularly robust^36^. Gallup has also studied the feeling of love in 136 countries with a single survey item that asked whether respondents had experienced the feeling of love “during a lot of the day yesterday^37^.” Other studies have found that some types of love, such as romantic love, are biosocial universals but that they are expressed differently depending on cultural context^38^, particularly in individualist or collectivist contexts^39^. For example, collectivism and higher temperatures are associated with love in romantic relationships across 45 countries^40^. But none of these works directly explored the *expression* of love or care for others regardless of relationship type. Cross-cultural research on benevolent (agapic, altruistic, or compassionate) expressions of love has revealed some differences across nations, as well as generally strong support for this form of love around the world^16,41^. But these forms of love are generally narrower than loving care because they require a cost to self or the suffering of the other. “Caregiving”—also not synonymous with love and care—is not equally distributed across sociodemographic categories over time within a specific culture or cross-nationally^42–44^. Such findings give us reason to believe that love/care expression will vary across groups and contexts, including across nations.

### Assessing childhood predictors of love/care expression across countries

A vast conceptual and empirical literature supports the claim that experiences of love and care during childhood, especially with regard to secure attachment or emotional bond with caregiver(s), interact with and influence the quality of a person’s adult relationships and their well-being^3,45–50^. One of the primary ways that this occurs is through representations of relationships with parents, which refers to the “mental templates thought to derive from cumulative, affective interchanges with primary caregiving figures^51^.” Children have both basic (e.g., food, shelter) and higher (e.g., tenderness, feeling loved) needs that must be met by caregivers, and they experience different parenting styles (e.g., warm, responsive, permissive, demanding, authoritarian, authoritative) as parents seek to meet these needs^3^.

According to attachment theory, depending on the quality of their relationships with caregivers, children experience one of the following general attachment styles: secure, avoidant, or anxious^52,53^. These styles powerfully shape the child’s sense of self, view of the environment as safe or unsafe, and subsequent well-being later in life^3,54,55^. Greater attachment security is associated with increased subsequent expressions of love and care, including altruism, compassion, and volunteering^53^. Attachment insecurities are associated with lower prosocial tendencies later in life, including reduced empathic concern and willingness to help others^56^. Attachment styles are also related to a variety of religious experiences and practices. For example, God has been found to “function psychologically as an attachment figure” and anxious attachment to God has been associated with negative affect and neuroticism^57^. Various contextual and family variables also exert a strong influence on children, such as the presence of two parents while growing up. In the context of two-parent families, research shows that one parent can increase their involvement and quality of caregiving to make up for deficits in these critical areas on the part of the other parent^58^. But inadequate parenting, across a range of family structures, is associated with delinquency and other forms of antisocial behavior as well as adult criminality, and such deficient manifestations parenting might be seen as crude indicators of the opposite of showing love and care for others^59,60^.

Most of the literature on this topic to date has been based on samples drawn from Global North countries, most frequently the United States and Western Europe^61^. But similar findings have been found outside of these contexts and such results generally support the contention that adults will tend to engage in “loving like I was loved” when they were children^45,62,63^. However, there are important cultural variations in the quality and kind of love expressions found throughout the world and ways that these expressions change over time. For example, fathers in some countries have been portrayed as “strict, stern, and inexpressive disciplinarians^61^,” but empirical research finds that these same fathers do display warmth toward children, although not necessarily through verbal expressions of love and affection.

It is important to note that “considerably more studies have been conducted on love in intimate [romantic] relationships”^48^ than in other types of relationships, including among parents and children, friends, neighbors, coworkers, and others. Unlike the large number of studies on volunteering and other forms of prosocial behavior, relatively few studies directly address the topic of the childhood predictors of showing love and care to others in general. The studies that do exist often involve very small sample sizes, limited relationship types, and specific cultural groups. For example, qualitative interviews with 12 Malay Muslim mothers and daughters explored how childhood relationships and cultural factors were related to love in the mother–child relationship^45^. The findings of such studies, although important, are not generalizable to nations. Even less is known about how childhood predictors of love/care expression differ across cultures and across sociodemographic groups within those different cultures.

### The present study

The Global Flourishing Study—a study of over 200,000 individuals in 22 countries in all six populated continents with nationally-representative sampling in each country—represents the first time, to the best of our knowledge, that a survey item on *showing love or care* for others has been used outside a religious setting, let alone with nationally-representative data in more than one country. While the extant research on love as feeling, as compassion, as caregiving, and as agape is not equivalent to the broad framing of love/care expression as we have defined it, the present analysis allows for exploration of levels of love/care expression across countries and observation of sociodemographic distributions. We also present a cross-national analysis of a range of childhood predictors^64^ of showing love/care for others in adulthood. Our goal is to better understand how the means of love/care expression are ordered across different countries, how the frequency of showing love/care to others varies across key sociodemographic groups, and how different aspects of a child’s upbringing predict love/care expression in adulthood.

## Methods

The description of the methods below has been adapted from VanderWeele et al.^65^. Further methodological detail is available elsewhere^25–28,64–69^.

### Study sample

This study used data from the Global Flourishing Study (GFS), which examines the distribution of determinants of well-being across a sample of 202,898 participants from 22 geographically and culturally diverse countries, with nationally-representative sampling within each country. Wave 1 of the data included the following countries and territories: Argentina, Australia, Brazil, Egypt, Germany, Hong Kong (Special Administrative Region of China), India, Indonesia, Israel, Japan, Kenya, Mexico, Nigeria, the Philippines, Poland, South Africa, Spain, Sweden, Tanzania, Turkey, the United Kingdom, and the United States. These countries were selected to (a) maximize coverage of the world’s population, (b) ensure geographic, cultural, and religious diversity, and (c) prioritize feasibility and existing data collection infrastructure. Data collection for Wave 1 was conducted by Gallup Inc. primarily in 2023, although some countries began data collection in 2022; the exact timing of data collection varied by country^68^. The precise sampling design to ensure nationally-representative samples also varied by country, and further details are available elsewhere^68^. The data are publicly available through the Center for Open Science (https://www.cos.io/gfs). The translation process adhered to the TRAPD model (translation, review, adjudication, pretesting, and documentation) for cross-cultural survey research (ccsg.isr.umich.edu/chapters/translation/overview). Further details about the GFS study methodology and survey development were reported elsewhere^67–69^.

Our analysis used data from all participants in Wave 1 (*N* = 202,898). Poststratification and nonresponse adjustments were performed to ensure the sample was representative of the adult population in each country^25,68^.

### Outcome

Love/care expression (which, for brevity, we sometimes refer to as “loving care” or “love/care”) was assessed with the following question: “How often do you show someone in your life that you love or care for them?” Response options were a Likert-type scale ranging from 0 (never) to 10 (always). We analyzed this indicator as a continuous variable, with higher scores indicating higher frequency of love/care expression. This item has been used in religious settings^32^ but was adapted for general community surveys in the GFS^68^.

### Sociodemographic characteristics

Participants self-reported their sociodemographic characteristics, including: gender (male, female, other), marital status (single/never married, married, separated, divorced, widowed, or domestic partner), employment status (employed, self-employed, retired, student, homemaker, unemployed and searching, or other), educational attainment (up to 8 years, 9–15 years, or 16 + years), religious service attendance (> 1x/week, 1x/week, 1–3x/month, a few times a year, or never), and immigration status (born in this country, born in another country). Birth year/current age was assessed using participant reports of their current age (in years), and birth year was calculated based on the year of data collection minus their current age. We created the following age groups: 18–24, 25–29, 30–39, 40–49, 50–59, 60–69, 70–79, and 80 or older (see tables for birth year ranges). Religious affiliation in adulthood was also assessed, and response options included 15 major religions [e.g., Christianity, Islam, Hinduism, Buddhism, Judaism, etc.], “some other religion”, or “no religion/atheist/agnostic”; response categories varied by country^70^. Race and ethnicity were assessed in most but not all countries (Germany, Japan, Spain, and Sweden had restrictions on collecting such data), and response categories were unique to each country. Race and ethnicity was dichotomized as racial/ethnic plurality (the category with the largest proportion) and minority (collapsing other categories) in each country.

### Childhood factors

*Relationships with parents.* Participants were asked to assess the quality of their relationships with their mother and father separately: “Please think about your relationship with your [mother/father] when you were growing up. In general, would you say that relationship was very good, somewhat good, somewhat bad, or very bad?” Responses were dichotomized to “very/somewhat good” versus “very/somewhat bad” to reduce collinearity in regression models. “Does not apply” was treated as a dichotomous control variable for respondents who did not have a mother or father due to death or absence.

*Parents’ marital status*. Marital status of parents during childhood was assessed with the question: “Were your parents married to each other when you were around 12 years old?” Response options included “married,” “divorced,” “never married,” and “one or both of them had died.”

*Subjective financial status growing up.* Participants reported their subjective assessment of the financial status of their family while growing up with the question: “Which one of these phrases comes closest to your own feelings about your family’s household income when you were growing up, such as when YOU were around 12 years old?” Response options included “lived comfortably,” “got by,” “found it difficult,” and “found it very difficult.”

*Childhood abuse*. Abuse during childhood was assessed with the question: “Were you ever physically or sexually abused when you were growing up?” Response options were “yes” and “no.”

*Outsider in family growing up*. Participants were asked about their feelings of family belonging during childhood using the question: “When you were growing up, did you feel like an outsider in your family?” Response options were “yes” and “no.”

*Childhood health*. Self-rated health was assessed using responses to the question: “In general, how was your health when you were growing up? Was it excellent, very good, good, fair, or poor?”.

*Childhood religious service attendance*. The frequency of religious attendance during childhood was assessed using the question: “How often did YOU attend religious services or worship at a temple, mosque, shrine, church, or other religious building when YOU were around 12 years old?” Response options included > 1x/week, 1x/week, 1–3x/month, a few times a year, or never.

*Religious affiliation*. Childhood religious tradition/affiliation was assessed using the question: “What was your religion when you were 12 years old?” Response options included 15 major religious traditions (e.g., Christianity, Islam, Hinduism, Buddhism, Judaism) “some other religion,” and “no religion/atheist/agnostic.” Precise response categories varied by country^70^. To reduce data sparsity, in regression models the response categories of religious affiliation with a prevalence < 3% were collapsed. The “no religion/atheist/agnostic” group was used as the reference group when at least 3% of the observed sample within the country endorsed this category; otherwise, the most prominent religious group was used as the reference category.

### Statistical analyses

Descriptive statistics for the full sample, weighted to be nationally representative within each country, were estimated for each sociodemographic characteristic and childhood factors. Nationally-representative means for love/care expression were estimated separately for each country and ordered from highest to lowest, along with 95% confidence intervals, standard deviations, and Gini coefficients. The Gini coefficient, most well-known as a measure of income inequality, can also be used to quantify disparities well-being indicators across a population. It ranges from 0 to 1, with higher values indicating greater disparity. In this study, the Gini coefficient was used to assess disparities in the distribution of love/care expression within each country. Variation in love/care expression by the sociodemographic categories was estimated, with all analyses initially conducted by country (see online supplement). In the analyses on childhood predictors, a weighted linear regression model with complex survey adjusted standard errors was used to regress love/care expression on all childhood factors simultaneously. Primary analyses used random effects meta-analysis of the country-specific estimates^71,72^, along with 95% confidence intervals, standard errors, lower and upper limits of 95% prediction intervals, heterogeneity (τ), and I^2^ for estimating variation within a particular sociodemographic variable across countries^73^. Within each country, a global test of variation (or association) of love/care expression across levels of each sociodemographic characteristic (or childhood predictor) was conducted, and a pooled *p* value^74^ was reported across countries to evaluate if a given association for the sociodemographic characteristic (or childhood predictor) with love/care expression holds within at least one country. Bonferroni corrected *p* value thresholds are provided based on the number of sociodemographic characteristics or number of childhood predictors^75,76^. For each childhood predictor, in both the meta-analyses and country-specific analyses, we calculated *E*-values to evaluate sensitivity of the results to potential unmeasured confounding. An *E*-value is the minimum strength of the association an unmeasured confounder must have with both the outcome and the predictor, above and beyond all measured covariates, for an unmeasured confounder to explain away the observed association^77^. Religious affiliation and race and ethnicity, when available, were used as control variables in the childhood predictor analyses within countries and were also used in the sociodemographic analyses, but they were not included in the meta-analyses because their response options varied by country.

As a supplementary analysis, population weighted meta-analyses were performed to pool the regression coefficients from the country-specific analyses. These can be found in the online supplement, which contains Tables S1a–S24 and Figs. S1–S132. All analyses were pre-registered with COS prior to data access, with only slight subsequent modification in the regression analyses for childhood predictors due to multicollinearity (10.17605/OSF.IO/R7DHA; https://osf.io/a6rq5). Although the pre-registrations did not specify the operationalization of variables (e.g., dichotomization), we used approaches that were determined prior to the pre-registrations, as described in the GFS methodology articles^27,28^. All code to reproduce analyses are openly available in an online repository^26,27^. All meta-analyses were conducted in **R** (R Core Team 2024) using the *metafor* package^78^, and country-specific analyses were performed using StataMP 17. All statistical tests were 2-sided.

### Missing data

Missing data on all variables was imputed using multivariate imputation by chained equations, with five imputed datasets generated^79–82^. The imputation process incorporated the outcome, all sociodemographic and childhood variables, including race/ethnicity and religious affiliation when available, and sampling weights. Sampling weights were included in the imputation models to account for specific-variable missingness that may have been related to the probability of inclusion in the study. To account for variation in the assessment of religious affiliation and race/ethnicity across countries, the imputation process was conducted separately in each country. This within-country imputation approach ensured that the imputation models accurately reflected country-specific contexts and assessment methods.

### Accounting for complex sampling design

The GFS used different sampling schemes across countries based on availability of existing panels and recruitment needs^68^. All analyses accounted for the complex survey design components by including weights, primary sampling units, and strata.

## Results

Table 1 presents descriptive statistics for the variables employed in our sociodemographic and childhood-predictors analyses. Within each country, the sample is weighted so that the data are nationally representative. About half of survey respondents were middle aged (between 30 and 59 years old [53%]), women (51%), married (52%), employed (57%), and had between 9 and 15 years of education (57%). Almost two-thirds attended religious services at least a few times per year or more (62%), and the vast majority were born in the country in which the survey was conducted (94%). The majority of respondents reported a “very good” relationship with their mother (63%) and father (53%) growing up, but 14% reported experiencing child abuse and 24% reported that their financial status during childhood was “difficult” or “very difficult.” Forty-one percent attended religious services at least once per week during childhood. The United States had the most respondents (19%), while Turkey (0.7%) had the fewest. Tables S1a–S22a in the Supplementary Information display the distributions of respondents in each sociodemographic group within each of the countries.

**Table 1:** Nationally-representative descriptive statistics of the observed sample.

Variable	Proportion	Frequency
Sociodemographic characteristics		
Year of birth/age group		
1998–2005; age 18–24	0.14	28,557
1993–1998; age 25–29	0.10	20,635
1983–1993; age 30–39	0.20	40,018
1973–1983; age 40–49	0.17	34,182
1963–1973; age 50–59	0.16	31,489
1953–1963; age 60–69	0.14	27,420
1943–1953; age 70–79	0.08	16,496
1943 or earlier; 80 or older	0.02	4081
Missing	< 0.01	20
Gender		
Male	0.48	98,255
Female	0.51	103,624
Other	< 0.01	609
Missing	< 0.01	410
Marital status		
Single, never married	0.26	53,338
Married	0.52	106,405
Separated	0.03	5184
Divorced	0.06	11,492
Widowed	0.05	9736
Domestic partner	0.07	14,923
Missing	0.01	1820
Employment		
Employed for an employer	0.39	78,163
Self-employed	0.18	36,234
Retired	0.14	29,107
Student	0.06	11,648
Homemaker	0.11	21,619
Unemployed and looking for a job	0.08	16,922
None of these/other	0.04	8413
Missing	< 0.01	792
Education		
Up to 8 years	0.22	45,090
9–15 years	0.57	115,107
16 + years	0.21	42,555
Missing	< 0.01	146
Religious service attendance		
> 1x/week	0.13	26,519
1x/week	0.19	39,145
1−3x/month	0.10	19,758
A few times a year	0.20	41,434
Never	0.37	75,326
Missing	< 0.01	715
Immigration status		
Born in this country	0.94	191,006
Born in another country	0.05	9800
Missing	0.01	2092
Childhood factors		
Relationship with mother		
Very good	0.63	127,889
Somewhat good	0.26	52,362
Somewhat bad	0.05	11,056
Very bad	0.02	4638
Not applicable	0.03	5994
Missing	< 0.01	958
Relationship with father		
Very good	0.53	107,763
Somewhat good	0.27	55,663
Somewhat bad	0.08	15,782
Very bad	0.04	8295
Not applicable	0.07	14,026
Missing	0.01	1369
Parent marital status		
Married	0.75	151,884
Divorced	0.09	17,767
Never married	0.08	15,587
One or both had died	0.04	7772
Missing	0.05	9889
Subjective financial status growing up		
Lived comfortably	0.35	71,117
Got by	0.41	82,762
Found it difficult	0.18	35,769
Found it very difficult	0.06	12,578
Missing	< 0.01	673
Childhood abuse		
Yes	0.14	29,147
No	0.82	167,279
Missing	0.03	6473
Outsider growing up		
Yes	0.14	28,758
No	0.84	170,513
Not applicable	0.01	2749
Missing	< 0.01	879
Childhood health		
Excellent	0.33	67,188
Very good	0.31	62,993
Good	0.23	47,347
Fair	0.10	19,891
Poor	0.02	4956
Missing	< 0.01	523
Childhood religious service attendance		
At least 1x/week	0.41	83,225
1−3x/month	0.16	33,292
< 1x/month	0.18	36,938
Never	0.23	47,471
Missing	0.01	1973
Country		
Argentina	0.03	6724
Australia	0.02	3844
Brazil	0.07	13,204
Egypt	0.02	4729
Germany	0.05	9506
Hong Kong	0.01	3012
India	0.06	12,765
Indonesia	0.03	6992
Israel	0.02	3669
Japan	0.10	20,543
Kenya	0.06	11,389
Mexico	0.03	5776
Nigeria	0.03	6827
Philippines	0.03	5292
Poland	0.05	10,389
South Africa	0.01	2651
Spain	0.03	6290
Sweden	0.07	15,068
Tanzania	0.04	9075
Turkey	0.01	1473
United Kingdom	0.03	5368
United States	0.19	38,312

*N* = 202,898. Country-specific descriptive statistics are available in the Online Supplement.

### Cross-national analysis of love/care expression

Perhaps our most important finding is that showing love/care to others is a common practice across the countries in the sample. This is substantiated by the relatively high population-weighted mean in our meta-analysis for all 22 countries: 8.08 on a 0–10 scale (95% CI 7.99, 8.16) (see Table S23). Table 2 presents the ordered means of love/care expression by country, as well as measures of dispersion and the Gini-coefficient of inequality. Seventeen of the 22 countries had means of 8 or higher (the range was 5.96–9.05) and only two countries were below 7: Hong Kong and Japan, which were also the two countries with the greatest inequality in love/care expression (as measured by the Gini coefficient). Hong Kong and Japan were also among the seven countries with the highest within-country variation (as measured by standard deviation or Gini coefficient), along with Tanzania, Kenya, Egypt, India, and Turkey. In general, we see an inverse relationship between the Gini coefficient and mean scores of showing love/care: those with the highest Gini coefficients were in the bottom half of the distribution of showing love/care and those with the lowest Gini coefficients were in the top half of the distribution. The countries with the highest means of love/care expression were generally in the Global South (e.g., the top four were the Philippines, Indonesia, Tanzania, Mexico), while Western, developed countries generally placed somewhat lower (e.g., the U.K is 16th; Sweden, Germany, and Poland were still lower, although Israel was 5th). Although these findings are informative, we caution against interpreting them as a definitive “country ranking” because of cross-cultural complexities related to translation, response scales, and social norms^83^.

**Table 2:** Ordered means of showing love and care to others in each country.

Country	Mean	LCI	UCI	SD	Gini
Philippines	9.05	8.99	9.10	1.71	0.08
Indonesia	8.85	8.79	8.91	1.78	0.09
Tanzania	8.72	8.61	8.83	2.31	0.11
Mexico	8.71	8.65	8.77	1.85	0.10
Israel	8.60	8.47	8.72	1.66	0.10
South Africa	8.59	8.47	8.71	2.02	0.11
Kenya	8.57	8.50	8.64	2.42	0.13
United States	8.56	8.51	8.61	1.71	0.10
Argentina	8.51	8.43	8.58	2.13	0.12
Brazil	8.47	8.42	8.52	2.18	0.12
Nigeria	8.38	8.31	8.45	1.98	0.12
Australia	8.31	8.24	8.38	1.75	0.11
Spain	8.28	8.22	8.35	1.96	0.12
Egypt	8.27	8.17	8.37	2.67	0.15
India	8.20	8.13	8.27	3.03	0.17
United Kingdom	8.14	8.06	8.21	1.98	0.13
Sweden	8.11	8.08	8.15	1.87	0.12
Turkey	7.89	7.72	8.05	2.62	0.17
Germany	7.81	7.76	7.87	2.02	0.14
Poland	7.80	7.68	7.92	1.98	0.13
Hong Kong	6.52	6.40	6.64	2.55	0.22
Japan	5.96	5.92	5.99	2.20	0.20

*N* = 202,898. LCI, lower limit of 95% confidence interval; UCI, upper limit of 95% confidence interval; SD, standard deviation.

### Sociodemographic variation in love/care expression

Table 3 presents the meta-analysis of mean levels of love/care expression across sociodemographic factors and pooled over the 22 countries. Consistent with prior research, showing love/care varies across key sociodemographic categories. Across all countries, the mean scores of showing love/care were highest among older age groups, women, the married and the widowed, homemakers or retired individuals, the most highly educated (9–15 or 16 + years), those who attended religious services more than once a week, and the native-born. The greatest mean difference within a single sociodemographic category was for religious service attendance (0.69, ranging from mean = 8.60 for “ > 1x/week” and mean = 7.91 for “never”) and the smallest was for education (0.09, ranging from mean = 8.21 for “16 + years” and mean = 8.12 for “up to 8 years”). Indeed, the 95% confidence intervals for meta-analytic means overlap across all sociodemographics except for these two specific categories of religious service attendance (comparing the “ > 1x/week” and “never” groups, mean = 8.60, 95% CI 8.41, 8.79 and mean = 7.91, 95% CI 7.59, 8.24, respectively).

**Table 3:** Random effects meta-analysis of means of showing love and care to others by sociodemographic categories.

Variable	Category	Mean	95% CI	SE	Prediction interval		Heterogeneity (τ)	*I* ^2^	Global *p* value
					Lower	Upper			
Overall		8.20	(7.90, 8.49)	0.15	5.96	9.05	0.71	99.8	
Age group	18–24	7.90	(7.60, 8.21)	0.16	5.96	8.92	0.72	98.6	< 0.001**
	25–29	8.12	(7.80, 8.43)	0.16	5.83	9.17	0.74	98.5	
	30–39	8.10	(7.75, 8.45)	0.18	5.66	9.16	0.84	99.4	
	40–49	8.22	(7.89, 8.56)	0.17	5.66	9.08	0.79	99.2	
	50–59	8.27	(7.95, 8.59)	0.16	5.69	8.97	0.75	99.1	
	60–69	8.33	(8.03, 8.64)	0.16	6.04	9.11	0.72	99.0	
	70–79	8.32	(8.04, 8.60)	0.14	6.59	9.22	0.62	98.1	
	80 or older	8.40	(8.10, 8.69)	0.15	6.81	9.07	0.59	91.4	
Gender	Male	8.02	(7.71, 8.34)	0.16	5.61	8.98	0.76	99.6	< 0.001**
	Female	8.36	(8.07, 8.65)	0.15	6.28	9.12	0.69	99.6	
	Other	7.36	(6.70, 8.01)	0.33	5.41	8.48	1.07	85.3	
Marital status	Married	8.39	(8.10, 8.67)	0.14	6.20	9.10	0.67	99.6	< 0.001**
	Separated	8.06	(7.72, 8.39)	0.17	6.01	8.79	0.74	94.8	
	Divorced	8.07	(7.72, 8.42)	0.18	5.86	9.03	0.80	97.8	
	Widowed	8.42	(8.07, 8.77)	0.18	5.81	9.10	0.80	97.4	
	Domestic partner	8.23	(7.78, 8.68)	0.23	5.31	9.77	1.02	99.3	
	Single, never married	7.78	(7.39, 8.16)	0.20	5.25	8.86	0.91	99.5	
Employment status	Employed for an employer	8.18	(7.87, 8.49)	0.16	5.83	9.04	0.73	99.5	< 0.001**
	Self-employed	8.25	(7.99, 8.51)	0.13	6.09	9.06	0.62	98.5	
	Retired	8.35	(8.04, 8.66)	0.16	6.16	9.12	0.72	99.0	
	Student	7.89	(7.58, 8.20)	0.16	6.28	9.10	0.72	97.4	
	Homemaker	8.36	(8.04, 8.69)	0.17	5.95	9.07	0.77	98.6	
	Unemployed and looking for a job	7.85	(7.38, 8.31)	0.24	4.65	9.07	1.09	98.9	
	None of these/other	8.09	(7.76, 8.41)	0.17	6.20	9.07	0.73	95.5	
Education	Up to 8 years	8.12	(7.77, 8.47)	0.18	5.28	9.04	0.82	98.9	< 0.001**
	9–15 years	8.20	(7.89, 8.50)	0.16	5.87	9.05	0.73	99.7	
	16 + years	8.21	(7.91, 8.51)	0.15	6.17	8.99	0.71	99.4	
Religious service attendance	> 1x/week	8.60	(8.41, 8.79)	0.10	7.34	9.23	0.45	96.8	< 0.001**
	1x/week	8.38	(8.16, 8.60)	0.11	6.96	9.14	0.52	98.2	
	1−3x/month	8.16	(7.91, 8.41)	0.13	6.25	9.06	0.59	97.3	
	A few times a year	8.16	(7.87, 8.44)	0.15	6.28	8.90	0.67	99.1	
	Never	7.91	(7.59, 8.24)	0.17	5.64	8.59	0.78	99.5	
Immigration status	Born in this country	8.20	(7.90, 8.50)	0.15	5.96	9.05	0.71	99.8	0.007*
	Born in another country	8.06	(7.74,8.37)	0.16	6.04	8.86	0.71	95.9	

CI, confidence interval; SE, standard error. τ is the standard deviation of the distribution of means across countries, which is an indicator of cross-national heterogeneity. *I*^2^ is an estimate of the variability in means due to heterogeneity across countries vs. sampling variability. *p < 0.05; **p < 0.007 (Bonferroni corrected threshold). Global *p* value corresponds to a test of the null hypothesis that there are no differences between the groups for that sociodemographic characteristic in any of the 22 countries.

Our pooled analysis suggests that mean levels of love/care expression exhibited a monotonic trend decreasing with age groups, ranging from 8.40 (95% CI 8.10, 8.69) for the oldest group (80 years and older) to 7.90 (95% CI 7.60, 8.21) for the youngest group (aged 18–24), but the tau statistic revealed substantial variation across countries (tau = 0.59–0.84). There are also important exceptions to this trend when the individual country data are examined (see Figs. S1–S8 and S35–S62 in the Supplementary Information). For example, respondents in the Philippines reported the highest mean scores across several age groups (i.e., all except 50–59, 70–79, and 80 years or older), but the highest mean in the Philippines was for the 30–39 year group (mean = 9.16, 95% CI 9.07, 9.25), while the 70–79 year group’s mean was lower (mean = 8.40, 95% CI 7.93, 9.02), as was the mean for those who were 80 years or older (mean = 8.60, 95% CI 8.22, 8.98). Across most countries, women (8.36, 95% CI 8.07, 8.65) scored higher than men (mean = 8.02, 95% CI 7.71, 8.34)—except for Tanzania, South Africa, and Hong Kong, where the scores were virtually identical. Native-born respondents (mean = 8.20, 95% CI 7.90, 8.50) scored higher than those who were born in a different country (mean = 8.06, 95% CI 7.74, 8.37), except in Indonesia, Tanzania, Israel, Egypt, Spain, and Japan, where the pattern was reversed or the scores were virtually identical. It is also worth noting that although married individuals (mean = 8.39, 95% CI 8.10, 8.67) displayed relatively high levels of love/care, single individuals in some specific countries had even higher means (e.g., singles in Indonesia: mean = 9.05, 95% CI 8.75, 9.34, see Fig. S13). This suggests that the influence of the nation may sometimes be as important for love/care expression as are specific social statuses. Indeed, married individuals in Indonesia (mean = 8.90, 95% CI 8.83, 8.97, see Fig. S15) score lower than singles in Indonesia, but higher than the mean for married participants in all countries (reported above).

### Childhood predictors of love/care expression

Turning to the meta-analytic regression results for love/care expression on childhood predictor variables, in Table 4 we see the strongest associations for earlier birth cohorts relative to those born between 1998 and 2005 (e.g., those born 1953–1963: β = 0.41, 95% CI 0.25, 0.56; 1943–1953: β = 0.43, 95% CI 0.20, 0.67; 1943 or earlier: β = 0.50, 95% CI 0.22, 0.78), for women (versus men) (β = 0.36, 95% CI 0.26, 0.47), for excellent (versus good) self-rated health growing up (β = 0.34, 95% CI 0.20, 0.48), and for attending religious services in childhood weekly or more (versus “never”) (β = 0.28, 95% CI 014, 0.41). Positive associations were also evidenced for relationship with mother (β = 0.16, 95% CI 0.06, 0.26), relationship with father (β = 0.10, 95% CI 0.02, 0.18), subjective financial status (lived comfortably versus got by only; β = 0.13, 95% CI 0.04, 0.22), very good (versus good) self-rated health (β = 0.14, 95% CI 0.07, 0.21), religious service attendance 1–3x/month and less than 1x/month (versus never; β = 0.18, 95% CI 0.04, 0.31, β = 0.10, 95% CI 0.02, 0.18, respectively), and all of the other age categories. Negative associations were shown for experiencing childhood abuse (β = −0.09, 95% CI −0.17, −0.02), feeling like an outsider in one’s family growing up (β = −0.16, 95% CI −0.23, −0.08), and fair (versus good) self-rated health (β = −0.13, 95% CI −0.24, −0.01). Worse subjective financial status growing up (found it difficult and found it very difficult, versus got by) and all of the categories of parent marital status (compared to parents who were married) did not exhibit evidence of strong associations with showing love/care in adulthood.

**Table 4:** Random effects meta-analysis of regression of showing love and care for others on childhood predictors.

Variable	Category	Est	95% CI	SE	Estimated proportion of effects by threshold		Heterogeneity (τ)	*I* ^2^	Global *p* value
					< −0.10	> 0.10			
Relationship with mother	(Ref: Very bad/somewhat bad)								< 0.001**
	Very/somewhat good	0.16	(0.06, 0.26)	0.05	0.05	0.59	0.16	58.6	
Relationship with father	(Ref: Very bad/somewhat bad)								< 0.001**
	Very/somewhat good	0.10	(0.02, 0.18)	0.04	0.09	0.50	0.14	62.7	
Parent marital status	(Ref: Parents married)								0.001**
	No, divorced	−0.06	(−0.16, 0.05)	0.05	0.41	0.23	0.20	72.9	
	Single, never married	−0.08	(−0.16, 0.00)	0.04	0.45	0.00	0.11	39.7	
	No, one or both had died	0.02	(−0.09, 0.13)	0.06	0.18	0.23	0.18	51.9	
Subjective financial status growing up	(Ref: Got by)								< 0.001**
	Lived comfortably	0.13	(0.04, 0.22)	0.05	0.05	0.55	0.20	90.0	
	Found it difficult	0.00	(−0.05, 0.05)	0.02	0.00	0.09	0.06	27.6	
	Found it very difficult	−0.06	(−0.14, 0.03)	0.04	0.41	0.00	0.10	24.7	
Childhood abuse	(Ref: No)								< 0.001**
	Yes	−0.09	(−0.17, −0.02)	0.04	0.52	0.10	0.13	63.7	
Outsider growing up	(Ref: No)								< 0.001**
	Yes	−0.16	(−0.23, −0.08)	0.04	0.59	0.05	0.12	56.7	
Self-rated health growing up	(Ref: Good)								< 0.001**
	Excellent	0.34	(0.20, 0.48)	0.07	0.00	0.73	0.31	92.6	
	Very good	0.14	(0.07, 0.21)	0.04	0.00	0.59	0.14	73.0	
	Fair	−0.13	(−0.24,-0.01)	0.06	0.50	0.18	0.23	79.6	
	Poor	−0.12	(−0.33, 0.10)	0.11	0.55	0.18	0.41	75.9	
Immigration status	(Ref: Born in this country)								< 0.001**
	No	0.02	(−0.07, 0.11)	0.05	0.27	0.14	0.12	39.2	
Childhood religious service attendance	(Ref: Never)								< 0.001**
	At least 1x/week	0.28	(0.14, 0.41)	0.07	0.00	0.73	0.28	84.8	
	1–3x/month	0.18	(0.04, 0.31)	0.07	0.00	0.50	0.28	84.5	
	Less than 1x/month	0.10	(0.02, 0.18)	0.04	0.05	0.41	0.13	64.1	
Year of birth	(Ref: 1998–2005; age 18–24)								< 0.001**
	1993–1998; age 25–29	0.19	(0.09, 0.29)	0.05	0.05	0.68	0.19	70.1	
	1983–1993; age 30–39	0.21	(0.10, 0.32)	0.06	0.14	0.73	0.23	82.0	
	1973–1983; age 40–49	0.32	(0.16, 0.47)	0.08	0.14	0.68	0.35	90.2	
	1963–1973; age 50–59	0.35	(0.18, 0.51)	0.08	0.18	0.68	0.37	89.9	
	1953–1963; age 60–69	0.41	(0.25, 0.56)	0.08	0.09	0.82	0.34	86.0	
	1943–1953; age 70–79	0.43	(0.20, 0.67)	0.12	0.23	0.68	0.49	89.2	
	1943 or earlier; age 80 or older	0.50	(0.22, 0.78)	0.14	0.27	0.73	0.55	81.1	
Gender	(Ref: Male)								< 0.001**
	Female	0.36	(0.26, 0.47)	0.05	0.00	0.86	0.24	94.1	
	Other	−0.30	(−0.85, 0.24)	0.28	0.56	0.39	1.05	89.1	

*N* = 202,898. CI, confidence interval; SE, standard error. I^2^ is an estimate of the variability in means due to heterogeneity across countries vs. sampling variability. Global *p* value corresponds to a test of the null hypothesis that there is no association between the predictor and showing love and care for others in adulthood in any of the 22 countries. *p < 0.05; **p < 0.004 (Bonferroni corrected threshold).

We also found high heterogeneity in how some early-life conditions shape love/care expression in adulthood across the 22 countries, which suggests that the associations depend on social context (e.g., excellent self-rated health in childhood [τ = 0.31, I^2^ = 92.6%]). Looking at the Supplemental Tables (S1c–S22c), we see that relationships between childhood predictors and adult love/care expression often vary by country. For example, the negative relationship for experiencing childhood abuse with love/care expression had strong evidence only in Kenya, Mexico, the Philippines, and South Africa; curiously, the relationship is positive in the United States; and in the 17 other countries, the association includes the null value. Other seemingly anomalous findings include the positive relation between poor (versus good) childhood self-rated health and love/care expression in Sweden and the negative association between very good (versus good) health and showing love/care in the Philippines.

In the country-specific analyses (Supplementary Tables S1c–S22c), three of the childhood factors were each associated with love/care expression in at least half of the countries in the sample. Gender and birth year/current age were each associated with the outcome in nearly all countries, while childhood health was associated with the outcome in more than two-thirds of countries. Women (versus men) reported higher love/care in all countries with the lowest difference in the Philippines (β = 0.13, 95% CI 0.02, 0.24) and the highest score in Sweden (β = 0.74, 95% CI 0.66, 0.81). The results for “Other” gender (versus men) were more mixed due to the small sample size of the gender other group in many countries leading to large uncertainty in the estimates. There was strong evidence that all levels of religious service attendance (versus never attending) during childhood were associated with love/care expression in two countries (Hong Kong, Japan) and that weekly (versus never) religious service attendance during childhood specifically was associated with love/care expression in a larger number of countries (Argentina, Brazil, Germany, Spain, Mexico, Sweden, and Turkey). Interestingly, in Israel, less than weekly religious service attendance (versus never)—but not weekly attendance—in childhood was associated with higher love/care expression, and in Poland, at least monthly religious service attendance (versus never)—but not less than monthly attendance—in childhood was associated with higher love/care expression.

Some individual country-level findings seem especially worth noting. Showing love/care was negatively associated with age in India (*p* < 0.001), Philippines (*p* = 0.02), and Tanzania (*p* = 0.01). In Spain, being an immigrant had a strong, positive association with love/care expression (β = 0.49, 95% CI 0.32, 0.66). For childhood health, reports of the highest self-rated health assessment (i.e., excellent versus good) were associated with higher scores of love/care expression in more than half of the countries in the sample, with the highest estimate in Hong Kong (β = 1.34, 95% CI 0.94, 1.74) and the lowest estimate in Mexico (β = 0.15, 95% CI 0.00, 0.29). In the United States, the association between love/care expression and relationship with mother (β = 0.25, 95% CI 0.07, 0.42) and father (β = 0.26, 95% CI 0.11, 041) was roughly the same. In Japan, relationship with father (β = 0.37, 95% CI 0.28, 0.46) was higher than relationship with mother (β = 0.26, 95% CI 0.16, 0.36). In Spain, relationship with mother was very positive (β = 0.66, 95% CI 0.32, 1.00) but there was little evidence of an association with relationship with father (β = −0.02, 95% CI −0.27, 0.22). The findings for parent marital status varied across some countries. If one or both parents were deceased when the respondent was 12 years old, in India the association with love/care expression was positive (β = 0.48, 95% CI 0.22, 0.74) and in Nigeria it was negative (β = −0.54, 95% CI −0.87, −0.22). If the parents were divorced, the association was especially strong and negative in the Philippines (β = −0.95, 95% CI −1.74, −0.15).

### Robustness analysis

Table 5 presents the *E*-values and suggests that many of the observed associations between love/care expression and childhood predictors are moderately robust to unmeasured confounding. A particularly robust association was found between excellent (compared to good) self-rated health growing up and love/care expression (*E*-value = 1.58; E-value for 95% CI = 1.40). This suggests that in order to explain away the association between the relationship of excellent health and showing love/care in adulthood, an unmeasured confounder would need to be associated with both excellent health and love/care by risk ratios of 1.58 each (beyond the other covariates in the model). To shift the confidence interval to include the null, risk ratios of 1.40 would be required.

**Table 5:** Sensitivity of meta-analyzed childhood predictors to unmeasured confounding.

Variable	Predictor (level)	*E*-values^a^	
		Effect estimate^b^	95% CI
Relationship with mother	(Ref: Very bad/somewhat bad)		
	Very/somewhat good	1.35	1.20
Relationship with father	(Ref: Very bad/somewhat bad)		
	Very/somewhat good	1.25	1.09
Parent marital status	(Ref: Parents married)		
	No, divorced	1.19	1.00
	Single, never married	1.22	1.00
	No, one or both had died	1.11	1.00
Subjective financial status growing up	(Ref: Got by)		
	Lived comfortably	1.31	1.15
	Found it difficult	1.01	1.00
	Found it very difficult	1.18	1.00
Childhood abuse	(Ref: No)		
	Yes	1.25	1.10
Outsider growing up	(Ref: No)		
	Yes	1.34	1.23
Self-rated health growing up	(Ref: Good)		
	Excellent	1.58	1.40
	Very good	1.32	1.21
	Fair	1.30	1.07
	Poor	1.28	1.00
Immigration status	(Ref: Born in this country)		
	No	1.11	1.00
Childhood religious service attendance	(Ref: Never)		
	At least 1x/week	1.50	1.32
	1–3x/month	1.37	1.16
	Less than 1x/month	1.26	1.12
Year of birth	(Ref: 1998–2005; age 18–24)		
	1993–1998; age 25–29	1.39	1.25
	1983–1993; age 30–39	1.42	1.26
	1973–1983; age 40–49	1.56	1.35
	1963–1973; age 50–59	1.59	1.38
	1953–1963; age 60–69	1.67	1.47
	1943–1953; age 70–79	1.70	1.41
	1943 or earlier; age 80 or older	1.78	1.43
Gender	(Ref: Male)		
	Female	1.61	1.48
	Other	1.54	1.00

*N* = 202,898. CI, confidence interval. ^a^The formula for calculating *E*-values can be found in VanderWeele and Ding (2017).^75^ ^b^The *E*-value for the effect estimate is the minimum strength of association (on the risk ratio scale) that an unmeasured confounder would need to have with both the predictor and the outcome to entirely explain away the observed association between them, conditional on the measured covariates. ^c^The *E*-value for the limit of the 95% confidence interval closest to the null denote the minimum strength of association (on the risk ratio scale) that an unmeasured confounder would need to have with both the predictor and the outcome to shift the confidence interval to include the null value, conditional on the measured covariates.

As an additional sensitivity analysis and alternative to the primary random effect meta-analysis of childhood predictors, we ran a set of fixed effects meta-analyses using total 2023-population size weights reflecting differences in the number of people in each country. This involves weighting by individuals in each country, rather than by country, which elevates the influence of more populous countries such as India. For the sociodemographic population-weighted meta-analysis (Table S23 in the in the Supplementary Information), some of the means were lower (e.g., for gender) but others were higher (e.g., for religious service attendance) and the patterns remained largely unchanged. Table S24 in the Supplementary Information shows that the association for relationships with mother and father, childhood abuse, and feeling like an outsider were all attenuated relative to the random-effects meta-analysis. But the associations for subjective financial status, self-rated health, religious service attendance, and year of birth (all groups) were all stronger in terms of their association with love/care expression. In general, the overall pattern of findings remained fairly consistent with the primary analyses, suggesting that our findings are relatively robust.

## Discussion

**Fig. 1:**
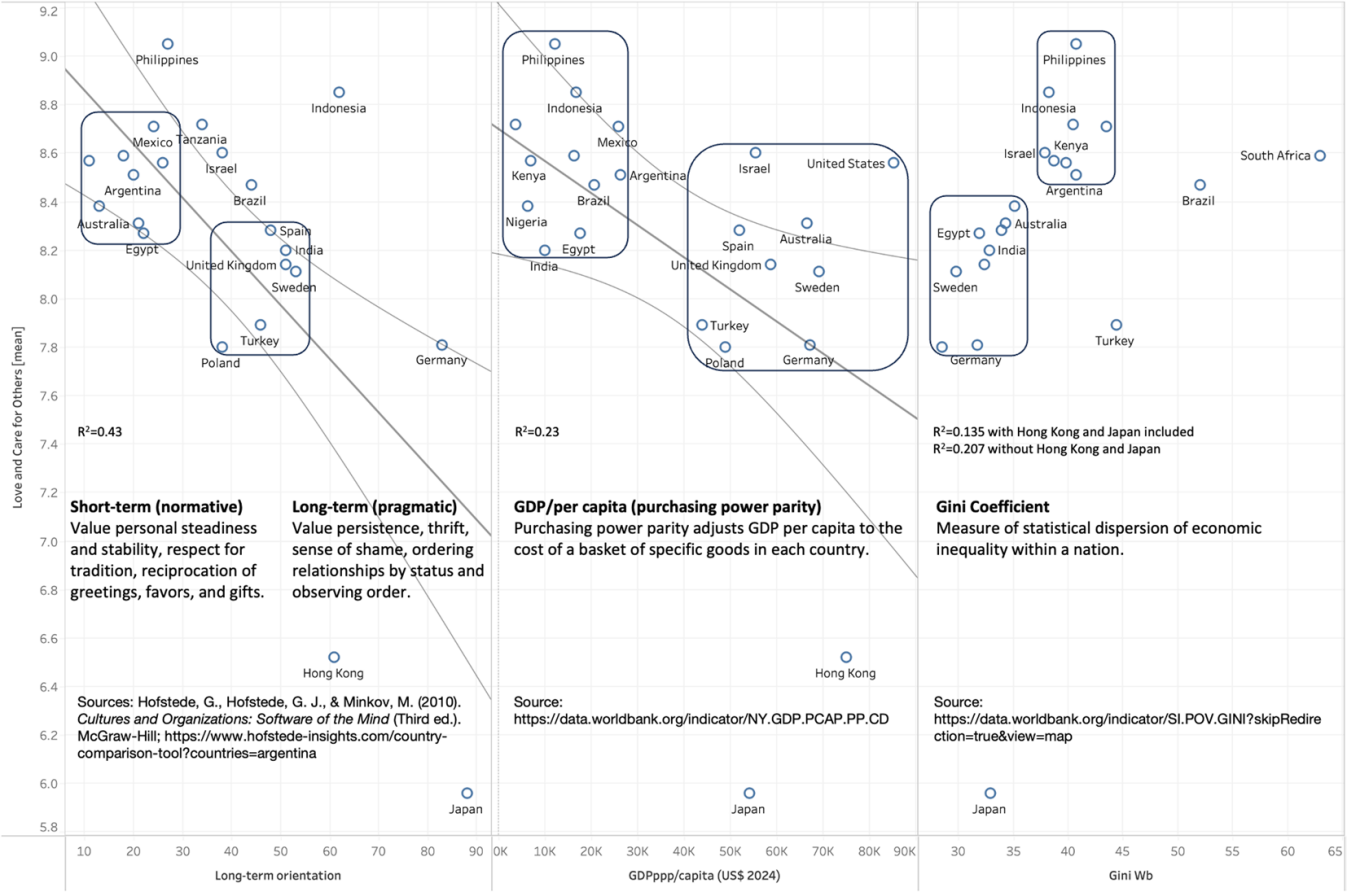
Relationship among love/care expression (y axis) and cultural and economic indicators by country (x axis), starting with long-term orientation in the left column, Gross Domestic Product in the middle column, and Gini coefficient in the right column.

Our results suggest that although showing love/care to others is indeed a “pan-cultural universal^11,12^,” there are also important cross-national differences in mean levels, sociodemographic associations, and childhood predictors. One striking finding was that nations in the Global South tended to have higher mean scores of love/care expression. To understand these cross-national differences, we examined a broad range of economic and cultural factors in a series of simple, post-hoc analyses. Our purpose was to suggest possible variables for future multivariate studies of causal mechanisms, rather than to prove such relationships. Western countries do seem to cluster at a lower level on some important indicators, compared with countries in the Global South (see Fig. 1). Countries with higher economic inequality as measured by the economic Gini coefficient^84^ show higher love/care, as do countries with a short-term orientation (i.e., those that emphasize the pursuit of immediate rewards rather than future rewards)^85^ and low GDP per capita^86^. Beyond these relationships, there is a moderately strong correlation (r = 0.49) between love/care and the Global Collectivism Index (GCI)^87^ and the GCI is negatively correlated (r = −0.86) with GDP for these countries. As with romantic love^39^, love/care expression in general seems to be shaped by collectivism, economics, and other sociodemographic variables. Uncovering why this is the case requires future research. For such studies, it might be helpful to note that in further post-hoc analyses, we found no significant correlations between love/care expression and (i) all of the Global Comparison Framework measures (e.g., climate, air quality, population, death rate, gender parity, social capital)^88^ (ii) Hofstede et al.^85^ power distance, individualism vs. collectivism (IDV), motivation towards achievement and success, uncertainty avoidance, and indulgence, and (iii) clustering by geographic region and primary religion. The disparate results for the IDV and CGI might be related to the specific aspects of collectivism that were measured, with the IDV focusing on job characteristics and the CGI aimed at more general life concerns.

The extant literature suggests that some aspects of life in WEIRD (Western, Educated, Industrialized, Rich, and Democratic) countries are perhaps less conducive to fostering love/care expression than elsewhere^89,90^. This might help explain why research has found much higher self-reported flourishing among samples of financially and materially precarious factory workers in the Global South relative to much more financially and materially stable samples in the U.S., including both factory workers and internal medicine and psychiatry residents^91,92^. Strong social connections and social support—and both of these factors are likely to be highly related to love/care expression—helped to account for the inverse relationship between material stability and flourishing^91^. In the *World Happiness Report*, one measure of social support related to the reliable loving care provided by friends was “the only highly significant predictor of life satisfaction, positive affect, and negative affect worldwide^91,93^.”

Strong associations between showing love and care and the quality of one’s social network appear in the GFS data as well: in addition to the individual correlations noted above between showing love and care and religious service attendance, the correlation between country-level means of showing love and care and those for relationship satisfaction, having an intimate friend, or self-rated level of social support are *r* = 0.75, 0.59, and 0.39 respectively, reflecting strong to moderate associations (analyses available on request). Causality in these associations might well be bi-directional: stronger social connections and support might be a function of a greater underlying disposition to show loving care, promoted by religion or other traditional norms; but on the other hand, a denser social network, including one’s spouse, children, friends, and acquaintances, might provide a wider range of opportunities for showing loving care. On either the “obligation” or the “opportunity” model of loving care’s causes, WEIRDer countries are arguably at a relative disadvantage: norms in favor of loving care might be sapped by the decline of religious affiliation and participation across the (economically) developed world, while the decline of social connection—as measured by declining rates of marriage, birth, or religious or civic participation, and rising rates of loneliness and social isolation—might leave individuals in wealthier countries with fewer daily opportunities for showing love and care than in the developing world^94–96^. And that these cross-national differences in showing love and care are at least in part culturally mediated is suggested by the relatively strong inverse correlation (*r* = −0.59) between these scores and Muthukrishna et al.^97^ cultural distance scale (calculated using the Philippines as the benchmark, and excluding Israel, Kenya, and Tanzania, for which data is missing from the online tool).

Japan provides the clearest illustration in the GFS of the coincidence of these trends, representing as it does an outlier within this sample on measures of showing love and care, relationship quality, intimate friendship, religious participation, and fertility. This apparent fraying of social bonds is reflected in the ongoing public debates within Japan about, e.g., the million-or-so men who have embraced a permanent condition of extreme withdrawal and isolation (*hikikomori*), eschewing virtually all social contact^98^, or about the collapse of small towns amid general population decline, where abandoned homes (*akiya*) can regularly be purchased for as little as $45^99^. These data are perhaps suggestive rather than definitive. But it does seem that “development” is not a simple or linear process, but instead potentially involves significant tradeoffs among determinants or domains of flourishing, which thus exhibit significant heterogeneity globally^100,101^. As such, discussions of cross-cultural flourishing and international development, which currently focuses on economic growth, personal financial and material stability, and various domains related to happiness and life satisfaction, may be enhanced by a consideration of love/care expression and an integral component of the good life.

Despite the important cross-cultural variations that surfaced in our findings, it is nonetheless also true that mean levels of showing love/care across nations were mostly above 8 (scale 0–10), suggesting that this practice is quite common around the world. As assessed by the Gini coefficient, inequality in love/care expression was also rather low across countries (ranging from 0.08 to 0.22). Showing love and care to another is therefore widely practiced both across and within nations. It is worth noting that inequality tended to be higher in nations with a lower mean, but all scores leaned strongly towards equality rather than inequality. A closer look at countries in Asia reveals the nuance at both country and regional levels. The fact that East Asian countries had the two lowest means (Japan [5.96], Hong Kong [6.52]) and highest Gini coefficients (0.20 and 0.22, respectively), as well as comparatively high standard deviations (2.20 and 2.50, respectively; the 22-country range was 1.66 to 3.03) seems noteworthy, especially when we consider that the mean (8.20) of the only South Asian country (India) in the sample ranked 15 out of 22, that its Gini coefficient was among the highest (0.17), and that its standard deviation was the highest of all (3.03). Importantly, two Southeast Asian countries had the highest means (Philippines [9.05], Indonesia [8.85]), the lowest Gini coefficients (0.08 and 0.09, respectively), and among the lowest standard deviations (1.71 and 1.78). Just as recent research has complicated standard interpretations of overarching cultural explanations, such as East vs. West or Individualism vs. Collectivism^102^, so do the patterns and variations in our findings seem to favor nuanced regional or country-specific explanations. And it is also possible that some, but not all, Asian respondents favor the midpoint of response scales rather than a high or low extreme^103^, suggesting that the relevant cultural norms might affect response patterns more than actual practices. Caution is also needed in interpreting cross-national differences due to other factors such as matters of translation, different modes of assessment, cultural norms, and seasonal effects arising from data being collected in different countries at different times of the year. Future research is required to better understand these possibilities.

It is not surprising that religious service attendance emerged as a strong and robust sociodemographic correlate and childhood predictor of love/care expression because religious traditions tend to emphasize showing loving care to others^17–22^. In addition to the meta-analytic summaries for the sociodemographic analyses, which affirmed this expectation, we also found that levels of love/care expression for those attending services greater than once per week as adults were significantly higher than for those never attending in all but three countries (i.e., Egypt, South Africa, and Spain). The associations for at least weekly service attendance in the childhood predictor analyses were also significant in the meta-analysis and were additionally evident in the country-specific analyses in 10 out of 22 nations.

The findings for older age were quite strong. Love/care generally increased as age increased, which is consistent with the perspective that showing love and care to others is part of the maturing process and consistent with later-life developmental concerns such as generativity: “leaving meaningful legacies and being concerned for the next generations [and] mastering the competence of care^104^.” Older adults have had more opportunities to care for others, including their own children and grandchildren and their elderly parents, and thus might be more skillful at caring and therefore more motivated to express love and care to others. The toward increasing frequency of love/care expression with age may not extend to the oldest ages when the capacity to demonstrate love may wane as one nears the very end of life, and this seemed especially apparent in countries like Brazil, Germany, Indonesia, Mexico, Nigeria, Poland, Tanzania, and Turkey.

There are several limitations to this study. The use of cross-sectional data for the sociodemographic associations with love/care expression prevents us from making causal interpretations. The childhood predictors were reported retrospectively, raising the possibility of recall bias. However, for recall bias to completely explain away the observed associations would require that the effect of adult love/care on biasing retrospective assessments of the childhood predictors would essentially have to be at least as strong as the observed associations themselves^105^, and some of these (e.g. excellent childhood health, weekly childhood religious service attendance) were moderately substantial. Social desirability bias is possible, and as we have noted, there are cross-cultural complexities related to translation and response scales which also potentially affect how respondents in some nations rated their love/care expression and other items^83,103^. It would be preferable to measure our outcome with a validated scale rather than a single item, but we were limited to the variables in the dataset. All research of this kind is subject to possible unmeasured confounding, although we have tried to mitigate this by simultaneously controlling for numerous childhood predictors and by reporting E-values. Some unmeasured variables that could be associated with love/care expression include attachment styles (secure, anxious, avoidant), degree and quality of experience of religious forms of love, and exposure to media messages related to prosocial expressions of love, although these would likely be somewhat correlated with the variables that were included in our analyses. Despite these limitations, our study had many strengths, including large, representative samples in each country, coverage of 22 culturally diverse nations on six continents, inclusion of key sociodemographic and childhood predictor variables vetted by a broad cross-cultural dialogue, and deployment of a novel survey item on showing love or care for others outside of a religious context.

## Conclusion

This study is the first to address important gaps in understanding how levels of love/care expression differ across nations and across sociodemographic groups within those different countries, as well as the potential childhood antecedents that are associated with love/care expression in adulthood across nations. Previous research has tended to address love as an emotion or feeling, many studies have been limited to relatively small samples in one or a handful of countries, and many published articles have focused on the United States or Western Europe. Our primary focus was on the findings of our meta-analysis of country means and our multivariate regression analysis of childhood predictors for a geographically and culturally diverse set of countries. There are important nuances to consider when looking at country-specific data and we caution against making simplistic, direct country comparisons. However, the patterns revealed in country-specific tables could provide the basis for constructing hypotheses for testing in future research, and we do believe that it is significant that we found relatively high levels of showing love and care both across and within 22 diverse countries, with somewhat higher levels reported in Global South countries. In adulthood, there was a range of sociodemographic correlates of higher love/care including older age, female gender, being married, various forms of employment or retirement, having more education, attending religious services more than one time each week, and being a native citizen. Childhood predictors of higher love/care included good relationships with parents, parents being married, financial security, not experiencing abuse, not feeling like an outsider, having very good or excellent health, attending services at least once each week, birth year, and female gender.

Further research into the reasons why countries in the Global South had somewhat higher levels of love/care expression, along with consideration of correlated factors (e.g., lower GDP, higher short-term orientation) that might account for this pattern, also seems warranted. Such research might inform social policy and shed light on the causes and consequences of a cultural emphasis on economic development versus warm, supportive relations. Perhaps studies will reveal policy levers that might encourage a more successful pursuit of both, in the service of holistic forms of flourishing. There is also great value in analyzing and synthesizing findings across articles published using the GFS data and a common methodology, in order to better understand how sociodemographic and childhood predictors are related to a range of important health and well-being outcomes^106^. In the meantime, we hope that our study contributes to the cross-cultural understanding of showing love and care, which is a principal source of happiness for many people around the world and often considered the highest virtue or value in many cultures.

## Supplementary Information

Below is the link to the electronic supplementary material.

Supplementary Material 1

Supplementary Material 2

## Data Availability

The data are publicly available through the Center for Open Science (https:/www.cos.io/gfs). All analyses were pre-registered with COS prior to data access (10.17605/OSF.IO/R7DHA; https://osf.io/a6rq5; all code to reproduce analyses are openly available in an online repository^26,27^.
