# Supplementary material for: Sociodemographic variation and childhood predictors of showing love and care for others in 22 countries
Source: Sci Rep. 2025 Dec 26;16:1796. doi: 10.1038/s41598-025-31380-9 (PMC12804932; doi:10.1038/s41598-025-31380-9)
Supplement: Supplementary file 1 — Supplementary Material 1 [file 41598_2025_31380_MOESM1_ESM.docx]

**Supplemental Tables for “Sociodemographic Variation and Childhood Predictors of Showing Love and Care for Others Across 22 Countries: A Cross-National Analysis”**

Matthew T. Lee, Renae Wilkinson, Katelyn N. G. Long, Brendan W. Case,

James L. Ritchie-Dunham, Matt Bradshaw, R. Noah Padgett,

Byron R. Johnson, Tyler J. VanderWeele

*Table S1a. Nationally Representative Descriptive Statistics of the Observed Sample (Argentina)*

| Variable | Proportion | Frequency |
| --- | --- | --- |
| Sociodemographic characteristics |  |  |
| Birth year/current age |  |  |
| 1998-2005; age 18-24 | 0.16 | 1108 |
| 1993-1998; age 25-29 | 0.11 | 719 |
| 1983-1993; age 30-39 | 0.21 | 1432 |
| 1973-1983; age 40-49 | 0.19 | 1254 |
| 1963-1973; age 50-59 | 0.15 | 1014 |
| 1953-1963; age 60-69 | 0.11 | 730 |
| 1943-1953; age 70-79 | 0.05 | 356 |
| 1943 or earlier; 80 or older | 0.02 | 112 |
| Missing | . | . |
| Gender |  |  |
| Male | 0.47 | 3143 |
| Female | 0.53 | 3542 |
| Other | 0.00 | 21 |
| Missing | 0.00 | 18 |
| Marital status |  |  |
| Single, never married | 0.35 | 2381 |
| Married | 0.23 | 1565 |
| Separated | 0.07 | 455 |
| Divorced | 0.05 | 321 |
| Widowed | 0.06 | 401 |
| Domestic partner | 0.23 | 1514 |
| Missing | 0.01 | 88 |
| Employment |  |  |
| Employed for an employer | 0.36 | 2440 |
| Self-employed | 0.26 | 1748 |
| Retired | 0.11 | 773 |
| Student | 0.05 | 354 |
| Homemaker | 0.10 | 639 |
| Unemployed and looking for a job | 0.08 | 569 |
| None of these/other | 0.03 | 179 |
| Missing | 0.00 | 22 |
| Education |  |  |
| Up to 8 years | 0.34 | 2263 |
| 9-15 years | 0.57 | 3823 |
| 16+ years | 0.09 | 635 |
| Missing | 0.00 | 3 |
| Religious service attendance |  |  |
| >1x/week | 0.08 | 532 |
| 1x/week | 0.12 | 773 |
| 1-3x/month | 0.07 | 461 |
| A few times a year | 0.29 | 1949 |
| Never | 0.44 | 2982 |
| Missing | 0.00 | 27 |
| Immigration status |  |  |
| Born in this country | 0.94 | 6346 |
| Born in another country | 0.05 | 348 |
| Missing | 0.00 | 29 |
| Religion |  |  |
| Christianity | 0.74 | 4992 |
| Islam | 0.00 | 9 |
| Hinduism | 0.00 | 6 |
| Buddhism | 0.01 | 35 |
| Judaism | 0.01 | 40 |
| Sikhism | 0.00 | 0 |
| Baha'i | . | . |
| Jainism | . | . |
| Shinto | . | . |
| Taoism | 0.00 | 2 |
| Confucianism | 0.00 | 0 |
| Primal, Animist, or Folk Religion | 0.00 | 19 |
| Spiritism | . | . |
| African-derived | . | . |
| Chinese | . | . |
| Some other religion | 0.02 | 156 |
| No religion/atheist/agnostic | 0.20 | 1352 |
| Missing | 0.02 | 111 |
| Race/ethnicity |  |  |
| Asian | 0.01 | 43 |
| Black | 0.01 | 95 |
| Indigenous | 0.02 | 129 |
| Mestizo(a) | 0.27 | 1801 |
| Mullato(a) | 0.01 | 75 |
| White | 0.51 | 3407 |
| Other | 0.02 | 104 |
| Missing | 0.16 | 1070 |
| Childhood factors |  |  |
| Relationship with mother |  |  |
| Very good | 0.66 | 4463 |
| Somewhat good | 0.21 | 1436 |
| Somewhat bad | 0.04 | 299 |
| Very bad | 0.03 | 216 |
| Not applicable | 0.04 | 273 |
| Missing | 0.01 | 36 |
| Relationship with father |  |  |
| Very good | 0.54 | 3612 |
| Somewhat good | 0.23 | 1537 |
| Somewhat bad | 0.07 | 440 |
| Very bad | 0.06 | 401 |
| Not applicable | 0.10 | 694 |
| Missing | 0.01 | 39 |
| Parent marital status |  |  |
| Married | 0.61 | 4110 |
| Divorced | 0.09 | 637 |
| Never married | 0.20 | 1368 |
| One or both parents had died | 0.03 | 199 |
| Missing | 0.06 | 410 |
| Subjective financial status growing up |  |  |
| Lived comfortably | 0.30 | 2042 |
| Got by | 0.34 | 2305 |
| Found it difficult | 0.27 | 1789 |
| Found it very difficult | 0.08 | 569 |
| Missing | 0.00 | 19 |
| Childhood abuse |  |  |
| Yes | 0.19 | 1302 |
| No | 0.78 | 5271 |
| Missing | 0.02 | 151 |
| Outsider growing up |  |  |
| Yes | 0.17 | 1165 |
| No | 0.81 | 5458 |
| Not applicable | 0.01 | 68 |
| Missing | 0.00 | 33 |
| Childhood health |  |  |
| Excellent | 0.36 | 2402 |
| Very good | 0.27 | 1819 |
| Good | 0.27 | 1830 |
| Fair | 0.08 | 505 |
| Poor | 0.02 | 156 |
| Missing | 0.00 | 12 |
| Childhood religious service attendance |  |  |
| At least 1x/week | 0.39 | 2601 |
| 1-3x/month | 0.18 | 1204 |
| <1x/month | 0.16 | 1059 |
| Never | 0.27 | 1808 |
| Missing | 0.01 | 53 |
| Childhood religion |  |  |
| Christianity | 0.86 | 5805 |
| Islam | 0.00 | 11 |
| Hinduism | 0.00 | 2 |
| Buddhism | 0.00 | 3 |
| Judaism | 0.01 | 51 |
| Sikhism | 0.00 | 5 |
| Baha'i | . | . |
| Jainism | . | . |
| Shinto | . | . |
| Taoism | 0.00 | 1 |
| Confucianism | . | . |
| Primal, Animist, or Folk Religion | 0.00 | 17 |
| Spiritism | . | . |
| African-derived | . | . |
| Chinese | . | . |
| Some other religion | 0.00 | 10 |
| No religion/atheist/agnostic | 0.10 | 697 |
| Missing | 0.02 | 122 |

*Note*. *N* = 6,724.

*Table S1b. Variations Across Sociodemographic Characteristics (Argentina)*

| Characteristic | Mean | SE | LCI | UCI | Global *p*-value |
| --- | --- | --- | --- | --- | --- |
| Age group |  |  |  |  |  |
| 18-24 | 8.06 | 0.10 | 7.87 | 8.26 | 0.00 |
| 25-29 | 8.07 | 0.14 | 7.80 | 8.35 | . |
| 30-39 | 8.40 | 0.09 | 8.23 | 8.58 | . |
| 40-49 | 8.70 | 0.08 | 8.55 | 8.85 | . |
| 50-59 | 8.85 | 0.09 | 8.68 | 9.02 | . |
| 60-69 | 8.78 | 0.11 | 8.56 | 9.00 | . |
| 70-79 | 8.78 | 0.16 | 8.47 | 9.09 | . |
| 80 or older | 9.05 | 0.23 | 8.59 | 9.52 | . |
| Gender |  |  |  |  |  |
| Male | 8.17 | 0.06 | 8.05 | 8.29 | 0.00 |
| Female | 8.80 | 0.05 | 8.72 | 8.89 | . |
| Other | 8.57 | 0.44 | 7.57 | 9.57 | . |
| Marital status |  |  |  |  |  |
| Single, never married | 8.11 | 0.07 | 7.97 | 8.26 | 0.00 |
| Married | 8.78 | 0.07 | 8.64 | 8.92 | . |
| Separated | 8.56 | 0.14 | 8.30 | 8.83 | . |
| Divorced | 8.65 | 0.16 | 8.34 | 8.96 | . |
| Widowed | 9.11 | 0.11 | 8.90 | 9.32 | . |
| Domestic partner | 8.64 | 0.07 | 8.50 | 8.79 | . |
| Employment |  |  |  |  |  |
| Employed for an employer | 8.45 | 0.06 | 8.32 | 8.57 | 0.00 |
| Self-employed | 8.42 | 0.08 | 8.27 | 8.57 | . |
| Retired | 8.88 | 0.11 | 8.68 | 9.09 | . |
| Student | 8.11 | 0.12 | 7.86 | 8.35 | . |
| Homemaker | 8.93 | 0.12 | 8.71 | 9.16 | . |
| Unemployed and looking for a job | 8.40 | 0.14 | 8.12 | 8.69 | . |
| None of these/other | 8.18 | 0.31 | 7.56 | 8.79 | . |
| Education |  |  |  |  |  |
| Up to 8 years | 8.60 | 0.08 | 8.44 | 8.77 | 0.21 |
| 9-15 years | 8.47 | 0.04 | 8.39 | 8.55 | . |
| 16+ years | 8.39 | 0.09 | 8.21 | 8.57 | . |
| Religious service attendance |  |  |  |  |  |
| >1x/week | 9.24 | 0.08 | 9.07 | 9.40 | 0.00 |
| 1x/week | 8.65 | 0.12 | 8.42 | 8.88 | . |
| 1-3x/month | 8.41 | 0.17 | 8.08 | 8.75 | . |
| A few times a year | 8.67 | 0.06 | 8.54 | 8.79 | . |
| Never | 8.25 | 0.06 | 8.13 | 8.37 | . |
| Immigration status |  |  |  |  |  |
| Born in this country | 8.51 | 0.04 | 8.43 | 8.58 | 0.81 |
| Born in another country | 8.46 | 0.19 | 8.10 | 8.83 | . |
| Religion |  |  |  |  |  |
| Christianity | 8.62 | 0.04 | 8.54 | 8.70 | 0.00 |
| Islam | 8.38 | 1.46 | 3.55 | 13.22 | . |
| Hinduism | 5.48 | 1.39 | -0.52 | 11.47 | . |
| Buddhism | 8.51 | 0.29 | 7.92 | 9.10 | . |
| Judaism | 8.52 | 0.39 | 7.70 | 9.33 | . |
| Sikhism | 7.00 | . | . | . | . |
| Baha'i | . | . | . | . | . |
| Jainism | . | . | . | . | . |
| Shinto | . | . | . | . | . |
| Taoism | 7.41 | 0.74 | -114.37 | 129.19 | . |
| Confucianism | 6.00 | . | . | . | . |
| Primal, Animist, or Folk Religion | 8.74 | 0.43 | 7.78 | 9.70 | . |
| Spiritism | . | . | . | . | . |
| African-derived | . | . | . | . | . |
| Chinese | . | . | . | . | . |
| Some other religion | 8.79 | 0.32 | 8.15 | 9.43 | . |
| No religion/atheist/agnostic | 8.07 | 0.09 | 7.89 | 8.25 | . |
| Race/ethnicity |  |  |  |  |  |
| Asian | 8.58 | 0.44 | 7.67 | 9.50 | 0.31 |
| Black | 8.77 | 0.39 | 7.98 | 9.55 | . |
| Indigenous | 7.86 | 0.32 | 7.21 | 8.50 | . |
| Mestizo(a) | 8.54 | 0.07 | 8.41 | 8.68 | . |
| Mullato(a) | 8.43 | 0.42 | 7.58 | 9.28 | . |
| White | 8.52 | 0.05 | 8.42 | 8.62 | . |
| Other | 8.08 | 0.29 | 7.51 | 8.65 | . |

*Note*. *N* = 6,724. SE, standard error; LCI, lower confidence interval; UCI, upper confidence interval.

*Table S1c. Variations Across Childhood Predictors (Argentina)*

| Characteristic | Coef. | SE | Prob. | LCI | UCI | Global *p*-value |
| --- | --- | --- | --- | --- | --- | --- |
| Relationship with mother (Ref: Very/somewhat bad) |  |  |  |  |  |  |
| Very/somewhat good | 0.25 | 0.16 | 0.12 | -0.06 | 0.57 | 0.12 |
| Relationship with father (Ref: Very/somewhat bad) |  |  |  |  |  |  |
| Very/somewhat good | 0.21 | 0.13 | 0.10 | -0.04 | 0.46 | 0.10 |
| Parent marital status (Ref: Married) |  |  |  |  |  |  |
| Divorced | 0.13 | 0.13 | 0.31 | -0.12 | 0.38 | 0.80 |
| Never married | 0.01 | 0.11 | 0.90 | -0.21 | 0.23 | . |
| One or both parents had died | -0.03 | 0.26 | 0.92 | -0.55 | 0.50 | . |
| Subjective financial status growing up (Ref: Got by) |  |  |  |  |  |  |
| Lived comfortably | 0.24 | 0.09 | 0.01 | 0.07 | 0.41 | 0.05 |
| Found it difficult | 0.15 | 0.10 | 0.11 | -0.04 | 0.34 | . |
| Found it very difficult | 0.05 | 0.19 | 0.77 | -0.31 | 0.42 | . |
| Childhood abuse (Ref: No) |  |  |  |  |  |  |
| Yes | -0.18 | 0.09 | 0.06 | -0.36 | 0.01 | 0.06 |
| Outsider growing up (Ref: No) |  |  |  |  |  |  |
| Yes | -0.38 | 0.14 | 0.01 | -0.64 | -0.11 | 0.01 |
| Childhood health (Ref: Good) |  |  |  |  |  |  |
| Excellent | 0.44 | 0.09 | 0.00 | 0.25 | 0.62 | 0.00 |
| Very good | 0.13 | 0.10 | 0.19 | -0.06 | 0.32 | . |
| Fair | 0.15 | 0.16 | 0.35 | -0.17 | 0.47 | . |
| Poor | -0.68 | 0.46 | 0.14 | -1.59 | 0.22 | . |
| Immigration status (Ref: Born in this country) |  |  |  |  |  |  |
| Born in another country | -0.06 | 0.17 | 0.71 | -0.41 | 0.28 | 0.71 |
| Childhood religious service attendance (Ref: Never) |  |  |  |  |  |  |
| At least 1x/week | 0.24 | 0.10 | 0.02 | 0.04 | 0.44 | 0.00 |
| 1-3x/month | -0.02 | 0.12 | 0.87 | -0.25 | 0.22 | . |
| <1x/month | -0.14 | 0.12 | 0.27 | -0.38 | 0.10 | . |
| Gender (Ref: Male) |  |  |  |  |  |  |
| Female | 0.72 | 0.08 | 0.00 | 0.57 | 0.87 | 0.00 |
| Other | 0.89 | 0.40 | 0.03 | 0.11 | 1.67 | . |
| Birth year/current age (Ref: 1998-2005; age 18-24) |  |  |  |  |  |  |
| 1993-1998; age 25-29 | -0.06 | 0.16 | 0.71 | -0.38 | 0.26 | 0.00 |
| 1983-1993; age 30-39 | 0.31 | 0.13 | 0.02 | 0.05 | 0.57 | . |
| 1973-1983; age 40-49 | 0.59 | 0.12 | 0.00 | 0.35 | 0.84 | . |
| 1963-1973; age 50-59 | 0.74 | 0.13 | 0.00 | 0.48 | 1.01 | . |
| 1953-1963; age 60-69 | 0.67 | 0.15 | 0.00 | 0.38 | 0.96 | . |
| 1943-1953; age 70-79 | 0.66 | 0.18 | 0.00 | 0.31 | 1.02 | . |
| 1943 or earlier; age 80 or older | 0.72 | 0.26 | 0.01 | 0.22 | 1.23 | . |
| Mother absence/presence (Ref: Present) |  |  |  |  |  |  |
| Absent | 0.01 | 0.17 | 0.94 | -0.32 | 0.35 | 0.94 |
| Father absence/presence (Ref: Present) |  |  |  |  |  |  |
| Absent | 0.06 | 0.14 | 0.70 | -0.23 | 0.34 | 0.70 |
| Childhood religion (Ref: No religion/atheist/agnostic) |  |  |  |  |  |  |
| Christianity | -0.13 | 0.14 | 0.36 | -0.41 | 0.15 | 0.65 |
| Some other religion | -0.15 | 0.27 | 0.58 | -0.67 | 0.37 | . |
| Race/ethnicity (Ref: Ethnic plurality) |  |  |  |  |  |  |
| Ethnic minority | 0.06 | 0.09 | 0.50 | -0.12 | 0.24 | 0.50 |
| *Note*. *N* = 6,724. SE, standard error; LCI, lower confidence interval; UCI, upper confidence interval. | | | | | | |

*Table S1d. E-Values and E-Value Limits for the Coefficients Shown in Table S1c (Argentina)*

| Characteristic | *E*-value for estimate^a^ | *E*-value for 95% CI^b^ |
| --- | --- | --- |
| Relationship with mother (Ref: Very/somewhat bad) |  |  |
| Very/somewhat good | 1.38 | 1.00 |
| Relationship with father (Ref: Very/somewhat bad) |  |  |
| Very/somewhat good | 1.34 | 1.00 |
| Parent marital status (Ref: Married) |  |  |
| Divorced | 1.25 | 1.00 |
| Never married | 1.07 | 1.00 |
| One or both parents had died | 1.10 | 1.00 |
| Subjective financial status growing up (Ref: Got by) |  |  |
| Lived comfortably | 1.37 | 1.17 |
| Found it difficult | 1.27 | 1.00 |
| Found it very difficult | 1.15 | 1.00 |
| Childhood abuse (Ref: No) |  |  |
| Yes | 1.30 | 1.00 |
| Outsider growing up (Ref: No) |  |  |
| Yes | 1.50 | 1.23 |
| Childhood health (Ref: Good) |  |  |
| Excellent | 1.56 | 1.38 |
| Very good | 1.25 | 1.00 |
| Fair | 1.27 | 1.00 |
| Poor | 1.78 | 1.00 |
| Immigration status (Ref: Born in this country) |  |  |
| Born in another country | 1.16 | 1.00 |
| Childhood religious service attendance (Ref: Never) |  |  |
| At least 1x/week | 1.37 | 1.13 |
| 1-3x/month | 1.08 | 1.00 |
| <1x/month | 1.26 | 1.00 |
| Gender (Ref: Male) |  |  |
| Female | 1.81 | 1.68 |
| Other | 1.97 | 1.23 |
| Birth year/current age (Ref: 1998-2005; age 18-24) |  |  |
| 1993-1998; age 25-29 | 1.16 | 1.00 |
| 1983-1993; age 30-39 | 1.43 | 1.13 |
| 1973-1983; age 40-49 | 1.70 | 1.47 |
| 1963-1973; age 50-59 | 1.83 | 1.59 |
| 1953-1963; age 60-69 | 1.77 | 1.50 |
| 1943-1953; age 70-79 | 1.76 | 1.43 |
| 1943 or earlier; age 80 or older | 1.81 | 1.34 |
| Mother absence/presence (Ref: Present) |  |  |
| Absent | 1.07 | 1.00 |
| Father absence/presence (Ref: Present) |  |  |
| Absent | 1.15 | 1.00 |
| Childhood religion (Ref: No religion/atheist/agnostic) |  |  |
| Christianity | 1.25 | 1.00 |
| Some other religion | 1.27 | 1.00 |
| Race/ethnicity (Ref: Ethnic plurality) |  |  |
| Ethnic minority | 1.16 | 1.00 |

*Note*. CI, confidence interval. ^a^The formula for calculating *E*-values can be found in VanderWeele and Ding (2017) The *E*-value for the effect estimate is the minimum strength of association (on the risk ratio scale) that an unmeasured confounder would need to have with both the predictor and the outcome to entirely explain away the observed association between them, conditional on the measured covariates. ^b^The *E*-value for the limit of the 95% confidence interval closest to the null denote the minimum strength of association (on the risk ratio scale) that an unmeasured confounder would need to have with both the predictor and the outcome to shift the confidence interval to include the null value, conditional on the measured covariates.

*Table S2a. Nationally Representative Descriptive Statistics of the Observed Sample (Australia)*

| Variable | Proportion | Frequency |
| --- | --- | --- |
| Sociodemographic characteristic |  |  |
| Birth year/current age |  |  |
| 1998-2005; age 18-24 | 0.09 | 345 |
| 1993-1998; age 25-29 | 0.07 | 282 |
| 1983-1993; age 30-39 | 0.17 | 641 |
| 1973-1983; age 40-49 | 0.16 | 618 |
| 1963-1973; age 50-59 | 0.18 | 691 |
| 1953-1963; age 60-69 | 0.15 | 589 |
| 1943-1953; age 70-79 | 0.13 | 498 |
| 1943 or earlier; 80 or older | 0.05 | 178 |
| Missing | 0.00 | 2 |
| Gender |  |  |
| Male | 0.48 | 1861 |
| Female | 0.50 | 1941 |
| Other | 0.01 | 36 |
| Missing | 0.00 | 6 |
| Marital status |  |  |
| Single, never married | 0.22 | 855 |
| Married | 0.47 | 1797 |
| Separated | 0.04 | 158 |
| Divorced | 0.09 | 332 |
| Widowed | 0.06 | 215 |
| Domestic partner | 0.12 | 450 |
| Missing | 0.01 | 38 |
| Employment |  |  |
| Employed for an employer | 0.49 | 1881 |
| Self-employed | 0.10 | 380 |
| Retired | 0.24 | 912 |
| Student | 0.05 | 190 |
| Homemaker | 0.04 | 137 |
| Unemployed and looking for a job | 0.03 | 134 |
| None of these/other | 0.05 | 206 |
| Missing | 0.00 | 4 |
| Education |  |  |
| Up to 8 years | 0.02 | 70 |
| 9-15 years | 0.63 | 2434 |
| 16+ years | 0.35 | 1330 |
| Missing | 0.00 | 10 |
| Religious service attendance |  |  |
| >1x/week | 0.04 | 162 |
| 1x/week | 0.08 | 299 |
| 1-3x/month | 0.04 | 135 |
| A few times a year | 0.17 | 656 |
| Never | 0.67 | 2584 |
| Missing | 0.00 | 7 |
| Immigration status |  |  |
| Born in this country | 0.77 | 2953 |
| Born in another country | 0.23 | 885 |
| Missing | 0.00 | 6 |
| Religion |  |  |
| Christianity | 0.41 | 1592 |
| Islam | 0.01 | 45 |
| Hinduism | 0.01 | 31 |
| Buddhism | 0.01 | 36 |
| Judaism | 0.01 | 26 |
| Sikhism | 0.00 | 8 |
| Baha'i | 0.00 | 7 |
| Jainism | . | . |
| Shinto | . | . |
| Taoism | 0.00 | 5 |
| Confucianism | . | . |
| Primal, Animist, or Folk Religion | 0.01 | 23 |
| Spiritism | . | . |
| African-derived | . | . |
| Chinese | . | . |
| Some other religion | 0.01 | 39 |
| No religion/atheist/agnostic | 0.53 | 2020 |
| Missing | 0.00 | 15 |
| Race/ethnicity |  |  |
| Aboriginal | 0.01 | 53 |
| Australian | 0.51 | 1946 |
| Australian /British/European | 0.27 | 1047 |
| Chinese | 0.02 | 75 |
| Indian | 0.02 | 58 |
| Japanese | 0.00 | 1 |
| Malay | 0.00 | 11 |
| Sinhalese | 0.00 | 1 |
| Spanish | 0.00 | 2 |
| Sri Lankan Moor | 0.00 | 1 |
| Sri Lankan Tamil | 0.00 | 7 |
| Vietnamese | 0.00 | 7 |
| Taiwanese/Holo | . | . |
| Russian | 0.00 | 7 |
| Samoan | 0.00 | 4 |
| New Zealander | 0.02 | 91 |
| Other European | 0.09 | 357 |
| Other | 0.04 | 163 |
| Missing | 0.00 | 14 |
| Childhood factors |  |  |
| Relationship with mother |  |  |
| Very good | 0.66 | 2554 |
| Somewhat good | 0.24 | 925 |
| Somewhat bad | 0.06 | 218 |
| Very bad | 0.03 | 107 |
| Not applicable | 0.01 | 32 |
| Missing | 0.00 | 7 |
| Relationship with father |  |  |
| Very good | 0.53 | 2032 |
| Somewhat good | 0.30 | 1144 |
| Somewhat bad | 0.08 | 315 |
| Very bad | 0.05 | 196 |
| Not applicable | 0.04 | 148 |
| Missing | 0.00 | 9 |
| Parent marital status |  |  |
| Married | 0.79 | 3048 |
| Divorced | 0.12 | 462 |
| Never married | 0.05 | 187 |
| One or both parents had died | 0.02 | 96 |
| Missing | 0.01 | 52 |
| Subjective financial status growing up |  |  |
| Lived comfortably | 0.46 | 1756 |
| Got by | 0.39 | 1496 |
| Found it difficult | 0.11 | 422 |
| Found it very difficult | 0.04 | 154 |
| Missing | 0.00 | 16 |
| Childhood abuse |  |  |
| Yes | 0.26 | 995 |
| No | 0.73 | 2790 |
| Missing | 0.02 | 59 |
| Outsider growing up |  |  |
| Yes | 0.20 | 756 |
| No | 0.80 | 3062 |
| Not applicable | 0.00 | 6 |
| Missing | 0.00 | 19 |
| Childhood health |  |  |
| Excellent | 0.45 | 1736 |
| Very good | 0.28 | 1087 |
| Good | 0.16 | 603 |
| Fair | 0.08 | 308 |
| Poor | 0.03 | 106 |
| Missing | 0.00 | 4 |
| Childhood religious service attendance |  |  |
| At least 1x/week | 0.35 | 1362 |
| 1-3x/month | 0.13 | 486 |
| <1x/month | 0.16 | 600 |
| Never | 0.34 | 1307 |
| Missing | 0.02 | 90 |
| Childhood religion |  |  |
| Christianity | 0.70 | 2678 |
| Islam | 0.01 | 48 |
| Hinduism | 0.01 | 39 |
| Buddhism | 0.00 | 16 |
| Judaism | 0.01 | 29 |
| Sikhism | 0.00 | 6 |
| Baha'i | 0.00 | 5 |
| Jainism | . | . |
| Shinto | . | . |
| Taoism | 0.00 | 1 |
| Confucianism | . | . |
| Primal, Animist, or Folk Religion | 0.00 | 4 |
| Spiritism | . | . |
| African-derived | . | . |
| Chinese | . | . |
| Some other religion | 0.00 | 8 |
| No religion/atheist/agnostic | 0.26 | 990 |
| Missing | 0.01 | 21 |

*Note. N* = 3,844.

*Table S2b. Variations Across Sociodemographic Characteristics (Australia)*

| Characteristic | Mean | SE | LCI | UCI | Global *p*-value |
| --- | --- | --- | --- | --- | --- |
| Age group |  |  |  |  |  |
| 18-24 | 7.42 | 0.18 | 7.06 | 7.78 | 0.00 |
| 25-29 | 8.12 | 0.15 | 7.83 | 8.42 | . |
| 30-39 | 8.11 | 0.10 | 7.91 | 8.31 | . |
| 40-49 | 8.43 | 0.09 | 8.26 | 8.60 | . |
| 50-59 | 8.39 | 0.07 | 8.25 | 8.53 | . |
| 60-69 | 8.37 | 0.07 | 8.23 | 8.51 | . |
| 70-79 | 8.79 | 0.06 | 8.67 | 8.91 | . |
| 80 or older | 8.72 | 0.17 | 8.38 | 9.07 | . |
| Gender |  |  |  |  |  |
| Male | 7.92 | 0.06 | 7.81 | 8.03 | 0.00 |
| Female | 8.68 | 0.04 | 8.59 | 8.76 | . |
| Other | 8.50 | 0.33 | 7.79 | 9.21 | . |
| Marital status |  |  |  |  |  |
| Single, never married | 7.40 | 0.10 | 7.20 | 7.61 | 0.00 |
| Married | 8.62 | 0.04 | 8.55 | 8.70 | . |
| Separated | 8.12 | 0.20 | 7.73 | 8.50 | . |
| Divorced | 8.45 | 0.12 | 8.21 | 8.69 | . |
| Widowed | 8.69 | 0.11 | 8.46 | 8.91 | . |
| Domestic partner | 8.55 | 0.09 | 8.38 | 8.73 | . |
| Employment |  |  |  |  |  |
| Employed for an employer | 8.26 | 0.05 | 8.16 | 8.35 | 0.00 |
| Self-employed | 8.39 | 0.11 | 8.18 | 8.60 | . |
| Retired | 8.61 | 0.06 | 8.50 | 8.73 | . |
| Student | 7.42 | 0.25 | 6.93 | 7.91 | . |
| Homemaker | 8.73 | 0.18 | 8.37 | 9.09 | . |
| Unemployed and looking for a job | 7.97 | 0.28 | 7.43 | 8.52 | . |
| None of these/other | 8.05 | 0.19 | 7.66 | 8.43 | . |
| Education |  |  |  |  |  |
| Up to 8 years | 8.33 | 0.46 | 7.38 | 9.28 | 0.89 |
| 9-15 years | 8.32 | 0.05 | 8.22 | 8.42 | . |
| 16+ years | 8.29 | 0.05 | 8.19 | 8.38 | . |
| Religious service attendance |  |  |  |  |  |
| >1x/week | 8.54 | 0.15 | 8.25 | 8.84 | 0.00 |
| 1x/week | 8.69 | 0.11 | 8.47 | 8.91 | . |
| 1-3x/month | 8.27 | 0.19 | 7.89 | 8.65 | . |
| A few times a year | 8.42 | 0.07 | 8.27 | 8.56 | . |
| Never | 8.22 | 0.05 | 8.13 | 8.32 | . |
| Immigration status |  |  |  |  |  |
| Born in this country | 8.31 | 0.04 | 8.23 | 8.39 | 0.97 |
| Born in another country | 8.31 | 0.07 | 8.16 | 8.45 | . |
| Religion |  |  |  |  |  |
| Christianity | 8.51 | 0.05 | 8.41 | 8.60 | 0.00 |
| Islam | 7.64 | 0.43 | 6.75 | 8.53 | . |
| Hinduism | 7.73 | 0.49 | 6.71 | 8.75 | . |
| Buddhism | 8.32 | 0.28 | 7.75 | 8.89 | . |
| Judaism | 8.64 | 0.29 | 8.04 | 9.23 | . |
| Sikhism | 8.31 | . | . | . | . |
| Baha'i | 9.57 | 0.22 | 7.66 | 11.48 | . |
| Jainism | . | . | . | . | . |
| Shinto | . | . | . | . | . |
| Taoism | 8.25 | . | . | . | . |
| Confucianism | . | . | . | . | . |
| Primal, Animist, or Folk Religion | 8.64 | 0.65 | 7.15 | 10.12 | . |
| Spiritism | . | . | . | . | . |
| African-derived | . | . | . | . | . |
| Chinese | . | . | . | . | . |
| Some other religion | 9.02 | 0.26 | 8.48 | 9.55 | . |
| No religion/atheist/agnostic | 8.15 | 0.06 | 8.04 | 8.26 | . |
| Race/ethnicity |  |  |  |  |  |
| Aboriginal | 8.30 | 0.44 | 7.39 | 9.20 | 0.00 |
| Australian | 8.43 | 0.05 | 8.33 | 8.52 | . |
| Australian /British/European | 8.29 | 0.07 | 8.14 | 8.43 | . |
| Chinese | 7.38 | 0.33 | 6.71 | 8.05 | . |
| Indian | 8.10 | 0.23 | 7.62 | 8.57 | . |
| Japanese | 6.91 | 0.99 | -156.45 | 170.28 | . |
| Malay | 7.65 | 0.91 | 3.74 | 11.56 | . |
| Sinhalese | 6.39 | . | . | . | . |
| Spanish | 8.88 | . | . | . | . |
| Sri Lankan Moor | 8.61 | 1.54 | -245.62 | 262.85 | . |
| Sri Lankan Tamil | 6.01 | 1.39 | 1.45 | 10.58 | . |
| Vietnamese | 7.42 | 0.22 | 5.50 | 9.34 | . |
| Taiwanese/Holo | . | . | . | . | . |
| Russian | 7.03 | 0.49 | 5.42 | 8.64 | . |
| Samoan | 6.22 | . | . | . | . |
| New Zealander | 8.56 | 0.19 | 8.19 | 8.94 | . |
| Other European | 8.22 | 0.12 | 7.98 | 8.45 | . |
| Other | 7.91 | 0.22 | 7.48 | 8.34 | . |

*Note*. *N* = 3,844. SE, standard error; LCI, lower confidence interval; UCI, upper confidence interval.

*Table S2c. Variations Across Childhood Predictors (Australia)*

| Characteristic | Coef. | SE | Prob. | LCI | UCI | Global *p*-value |
| --- | --- | --- | --- | --- | --- | --- |
| Relationship with mother (Ref: Very/somewhat bad) |  |  |  |  |  |  |
| Very/somewhat good | 0.35 | 0.16 | 0.03 | 0.03 | 0.68 | 0.03 |
| Relationship with father (Ref: Very/somewhat bad) |  |  |  |  |  |  |
| Very/somewhat good | 0.17 | 0.12 | 0.18 | -0.08 | 0.41 | 0.18 |
| Parent marital status (Ref: Married) |  |  |  |  |  |  |
| Divorced | 0.17 | 0.13 | 0.19 | -0.08 | 0.42 | 0.26 |
| Never married | 0.09 | 0.24 | 0.71 | -0.38 | 0.57 | . |
| One or both parents had died | 0.43 | 0.26 | 0.10 | -0.08 | 0.95 | . |
| Subjective financial status growing up (Ref: Got by) |  |  |  |  |  |  |
| Lived comfortably | 0.04 | 0.07 | 0.55 | -0.10 | 0.19 | 0.66 |
| Found it difficult | 0.15 | 0.12 | 0.23 | -0.09 | 0.39 | . |
| Found it very difficult | 0.11 | 0.24 | 0.65 | -0.36 | 0.57 | . |
| Childhood abuse (Ref: No) |  |  |  |  |  |  |
| Yes | -0.01 | 0.08 | 0.89 | -0.18 | 0.15 | 0.89 |
| Outsider growing up (Ref: No) |  |  |  |  |  |  |
| Yes | -0.07 | 0.11 | 0.51 | -0.29 | 0.14 | 0.51 |
| Childhood health (Ref: Good) |  |  |  |  |  |  |
| Excellent | 0.40 | 0.11 | 0.00 | 0.18 | 0.62 | 0.00 |
| Very good | 0.08 | 0.12 | 0.49 | -0.15 | 0.32 | . |
| Fair | 0.16 | 0.18 | 0.37 | -0.19 | 0.51 | . |
| Poor | -0.12 | 0.33 | 0.73 | -0.76 | 0.53 | . |
| Immigration status (Ref: Born in this country) |  |  |  |  |  |  |
| Born in another country | 0.07 | 0.09 | 0.46 | -0.11 | 0.24 | 0.46 |
| Childhood religious service attendance (Ref: Never) |  |  |  |  |  |  |
| At least 1x/week | 0.01 | 0.10 | 0.92 | -0.18 | 0.20 | 0.90 |
| 1-3x/month | -0.07 | 0.11 | 0.56 | -0.29 | 0.16 | . |
| <1x/month | 0.00 | 0.11 | 0.99 | -0.22 | 0.22 | . |
| Gender (Ref: Male) |  |  |  |  |  |  |
| Female | 0.73 | 0.07 | 0.00 | 0.59 | 0.86 | 0.00 |
| Other | 1.21 | 0.34 | 0.00 | 0.54 | 1.89 | . |
| Birth year/current age (Ref: 1998-2005; age 18-24) |  |  |  |  |  |  |
| 1993-1998; age 25-29 | 0.61 | 0.23 | 0.01 | 0.15 | 1.06 | 0.00 |
| 1983-1993; age 30-39 | 0.57 | 0.20 | 0.00 | 0.17 | 0.97 | . |
| 1973-1983; age 40-49 | 0.86 | 0.20 | 0.00 | 0.48 | 1.25 | . |
| 1963-1973; age 50-59 | 0.77 | 0.19 | 0.00 | 0.39 | 1.14 | . |
| 1953-1963; age 60-69 | 0.74 | 0.19 | 0.00 | 0.36 | 1.12 | . |
| 1943-1953; age 70-79 | 1.11 | 0.19 | 0.00 | 0.73 | 1.48 | . |
| 1943 or earlier; age 80 or older | 1.08 | 0.24 | 0.00 | 0.60 | 1.55 | . |
| Mother absence/presence (Ref: Present) |  |  |  |  |  |  |
| Absent | 0.40 | 0.20 | 0.05 | 0.00 | 0.80 | 0.05 |
| Father absence/presence (Ref: Present) |  |  |  |  |  |  |
| Absent | -0.30 | 0.23 | 0.19 | -0.76 | 0.15 | 0.19 |
| Childhood religion (Ref: No religion/atheist/agnostic) |  |  |  |  |  |  |
| Christianity | 0.15 | 0.10 | 0.14 | -0.05 | 0.35 | 0.28 |
| Some other religion | -0.01 | 0.22 | 0.98 | -0.44 | 0.43 | . |
| Race/ethnicity (Ref: Ethnic plurality) |  |  |  |  |  |  |
| Ethnic minority | -0.13 | 0.08 | 0.09 | -0.29 | 0.02 | 0.09 |
| *Note*. *N* = 3,844. SE, standard error; LCI, lower confidence interval; UCI, upper confidence interval. | | | | | | |

*Table S2d. E-Values and E-Value Limits for the Coefficients Shown in Table S2c (Australia)*

| Characteristic | *E*-value for estimate^a^ | *E*-value for 95% CI^b^ |
| --- | --- | --- |
| Relationship with mother (Ref: Very/somewhat bad) |  |  |
| Very/somewhat good | 1.48 | 1.11 |
| Relationship with father (Ref: Very/somewhat bad) |  |  |
| Very/somewhat good | 1.29 | 1.00 |
| Parent marital status (Ref: Married) |  |  |
| Divorced | 1.29 | 1.00 |
| Never married | 1.20 | 1.00 |
| One or both parents had died | 1.56 | 1.00 |
| Subjective financial status growing up (Ref: Got by) |  |  |
| Lived comfortably | 1.13 | 1.00 |
| Found it difficult | 1.27 | 1.00 |
| Found it very difficult | 1.22 | 1.00 |
| Childhood abuse (Ref: No) |  |  |
| Yes | 1.07 | 1.00 |
| Outsider growing up (Ref: No) |  |  |
| Yes | 1.18 | 1.00 |
| Childhood health (Ref: Good) |  |  |
| Excellent | 1.53 | 1.31 |
| Very good | 1.19 | 1.00 |
| Fair | 1.29 | 1.00 |
| Poor | 1.23 | 1.00 |
| Immigration status (Ref: Born in this country) |  |  |
| Born in another country | 1.17 | 1.00 |
| Childhood religious service attendance (Ref: Never) |  |  |
| At least 1x/week | 1.06 | 1.00 |
| 1-3x/month | 1.17 | 1.00 |
| <1x/month | 1.02 | 1.00 |
| Gender (Ref: Male) |  |  |
| Female | 1.83 | 1.70 |
| Other | 2.29 | 1.65 |
| Birth year/current age (Ref: 1998-2005; age 18-24) |  |  |
| 1993-1998; age 25-29 | 1.72 | 1.28 |
| 1983-1993; age 30-39 | 1.68 | 1.30 |
| 1973-1983; age 40-49 | 1.96 | 1.60 |
| 1963-1973; age 50-59 | 1.86 | 1.51 |
| 1953-1963; age 60-69 | 1.84 | 1.49 |
| 1943-1953; age 70-79 | 2.19 | 1.83 |
| 1943 or earlier; age 80 or older | 2.16 | 1.71 |
| Mother absence/presence (Ref: Present) |  |  |
| Absent | 1.52 | 1.00 |
| Father absence/presence (Ref: Present) |  |  |
| Absent | 1.43 | 1.00 |
| Childhood religion (Ref: No religion/atheist/agnostic) |  |  |
| Christianity | 1.27 | 1.00 |
| Some other religion | 1.04 | 1.00 |
| Race/ethnicity (Ref: Ethnic plurality) |  |  |
| Ethnic minority | 1.26 | 1.00 |

*Note*. CI, confidence interval. ^a^The formula for calculating *E*-values can be found in VanderWeele and Ding (2017) The *E*-value for the effect estimate is the minimum strength of association (on the risk ratio scale) that an unmeasured confounder would need to have with both the predictor and the outcome to entirely explain away the observed association between them, conditional on the measured covariates. ^b^The *E*-value for the limit of the 95% confidence interval closest to the null denote the minimum strength of association (on the risk ratio scale) that an unmeasured confounder would need to have with both the predictor and the outcome to shift the confidence interval to include the null value, conditional on the measured covariates.

*Table S3a. Nationally Representative Descriptive Statistics of the Observed Sample (Brazil)*

| Variable | Proportion | Frequency |
| --- | --- | --- |
| Sociodemographic characteristics |  |  |
| Birth year/current age |  |  |
| 1998-2005; age 18-24 | 0.15 | 1986 |
| 1993-1998; age 25-29 | 0.11 | 1468 |
| 1983-1993; age 30-39 | 0.22 | 2908 |
| 1973-1983; age 40-49 | 0.20 | 2638 |
| 1963-1973; age 50-59 | 0.16 | 2131 |
| 1953-1963; age 60-69 | 0.11 | 1435 |
| 1943-1953; age 70-79 | 0.04 | 510 |
| 1943 or earlier; 80 or older | 0.01 | 126 |
| Missing | . | . |
| Gender |  |  |
| Male | 0.48 | 6320 |
| Female | 0.52 | 6820 |
| Other | 0.00 | 35 |
| Missing | 0.00 | 30 |
| Marital status |  |  |
| Single, never married | 0.33 | 4347 |
| Married | 0.35 | 4646 |
| Separated | 0.04 | 594 |
| Divorced | 0.07 | 865 |
| Widowed | 0.03 | 408 |
| Domestic partner | 0.16 | 2081 |
| Missing | 0.02 | 263 |
| Employment |  |  |
| Employed for an employer | 0.28 | 3756 |
| Self-employed | 0.22 | 2918 |
| Retired | 0.12 | 1536 |
| Student | 0.05 | 624 |
| Homemaker | 0.10 | 1305 |
| Unemployed and looking for a job | 0.18 | 2419 |
| None of these/other | 0.03 | 448 |
| Missing | 0.02 | 199 |
| Education |  |  |
| Up to 8 years | 0.24 | 3139 |
| 9-15 years | 0.58 | 7665 |
| 16+ years | 0.18 | 2390 |
| Missing | 0.00 | 10 |
| Religious service attendance |  |  |
| >1x/week | 0.18 | 2386 |
| 1x/week | 0.17 | 2272 |
| 1-3x/month | 0.11 | 1398 |
| A few times a year | 0.30 | 3978 |
| Never | 0.24 | 3110 |
| Missing | 0.00 | 61 |
| Immigration status |  |  |
| Born in this country | 0.96 | 12688 |
| Born in another country | 0.01 | 153 |
| Missing | 0.03 | 363 |
| Religion |  |  |
| Christianity | 0.75 | 9911 |
| Islam | 0.00 | 6 |
| Hinduism | 0.00 | 1 |
| Buddhism | 0.00 | 37 |
| Judaism | 0.00 | 31 |
| Sikhism | . | . |
| Baha'i | 0.00 | 2 |
| Jainism | 0.00 | 2 |
| Shinto | 0.00 | 1 |
| Taoism | 0.00 | 2 |
| Confucianism | 0.00 | 6 |
| Primal, Animist, or Folk Religion | 0.00 | 15 |
| Spiritism | 0.05 | 696 |
| African-derived | 0.04 | 525 |
| Chinese | . | . |
| Some other religion | 0.01 | 144 |
| No religion/atheist/agnostic | 0.13 | 1712 |
| Missing | 0.01 | 113 |
| Race/ethnicity |  |  |
| Branca | 0.39 | 5169 |
| Preta | 0.12 | 1615 |
| Parda | 0.39 | 5125 |
| Amarela | 0.02 | 238 |
| Indigena | 0.01 | 131 |
| Other | 0.00 | 61 |
| Missing | 0.07 | 865 |
| Childhood factors |  |  |
| Relationship with mother |  |  |
| Very good | 0.63 | 8369 |
| Somewhat good | 0.27 | 3559 |
| Somewhat bad | 0.04 | 483 |
| Very bad | 0.02 | 214 |
| Not applicable | 0.04 | 507 |
| Missing | 0.01 | 73 |
| Relationship with father |  |  |
| Very good | 0.48 | 6364 |
| Somewhat good | 0.28 | 3654 |
| Somewhat bad | 0.08 | 1035 |
| Very bad | 0.06 | 756 |
| Not applicable | 0.10 | 1303 |
| Missing | 0.01 | 93 |
| Parent marital status |  |  |
| Married | 0.65 | 8546 |
| Divorced | 0.10 | 1384 |
| Never married | 0.15 | 1985 |
| One or both parents had died | 0.04 | 508 |
| Missing | 0.06 | 781 |
| Subjective financial status growing up |  |  |
| Lived comfortably | 0.38 | 4998 |
| Got by | 0.35 | 4616 |
| Found it difficult | 0.19 | 2484 |
| Found it very difficult | 0.08 | 1027 |
| Missing | 0.01 | 79 |
| Childhood abuse |  |  |
| Yes | 0.20 | 2606 |
| No | 0.77 | 10147 |
| Missing | 0.03 | 451 |
| Outsider growing up |  |  |
| Yes | 0.13 | 1659 |
| No | 0.85 | 11234 |
| Not applicable | 0.02 | 229 |
| Missing | 0.01 | 82 |
| Childhood health |  |  |
| Excellent | 0.40 | 5312 |
| Very good | 0.26 | 3392 |
| Good | 0.22 | 2873 |
| Fair | 0.10 | 1368 |
| Poor | 0.02 | 228 |
| Missing | 0.00 | 30 |
| Childhood religious service attendance |  |  |
| At least 1x/week | 0.48 | 6306 |
| 1-3x/month | 0.19 | 2491 |
| <1x/month | 0.20 | 2629 |
| Never | 0.13 | 1707 |
| Missing | 0.01 | 71 |
| Childhood religion |  |  |
| Christianity | 0.86 | 11403 |
| Islam | 0.00 | 15 |
| Hinduism | 0.00 | 1 |
| Buddhism | 0.00 | 27 |
| Judaism | 0.00 | 40 |
| Sikhism | . | . |
| Baha'i | 0.00 | 1 |
| Jainism | 0.00 | 4 |
| Shinto | 0.00 | 4 |
| Taoism | 0.00 | 1 |
| Confucianism | 0.00 | 7 |
| Primal, Animist, or Folk Religion | 0.00 | 17 |
| Spiritism | 0.03 | 336 |
| African-derived | 0.02 | 262 |
| Chinese | . | . |
| Some other religion | 0.01 | 87 |
| No religion/atheist/agnostic | 0.07 | 908 |
| Missing | 0.01 | 94 |

*Note*. *N* = 13,204.

*Table S3b. Variations Across Sociodemographic Characteristics (Brazil)*

| Characteristic | Mean | SE | LCI | UCI | Global *p*-value |
| --- | --- | --- | --- | --- | --- |
| Age group |  |  |  |  |  |
| 18-24 | 7.90 | 0.07 | 7.76 | 8.03 | 0.00 |
| 25-29 | 8.17 | 0.08 | 8.01 | 8.32 | . |
| 30-39 | 8.34 | 0.05 | 8.24 | 8.44 | . |
| 40-49 | 8.59 | 0.05 | 8.49 | 8.69 | . |
| 50-59 | 8.76 | 0.06 | 8.64 | 8.89 | . |
| 60-69 | 8.86 | 0.08 | 8.71 | 9.02 | . |
| 70-79 | 9.24 | 0.11 | 9.02 | 9.45 | . |
| 80 or older | 8.82 | 0.25 | 8.31 | 9.32 | . |
| Gender |  |  |  |  |  |
| Male | 8.32 | 0.04 | 8.24 | 8.40 | 0.00 |
| Female | 8.61 | 0.03 | 8.55 | 8.68 | . |
| Other | 8.20 | 0.43 | 7.32 | 9.08 | . |
| Marital status |  |  |  |  |  |
| Single, never married | 7.97 | 0.05 | 7.87 | 8.07 | 0.00 |
| Married | 8.82 | 0.03 | 8.75 | 8.88 | . |
| Separated | 8.50 | 0.12 | 8.26 | 8.74 | . |
| Divorced | 8.64 | 0.10 | 8.44 | 8.83 | . |
| Widowed | 9.04 | 0.14 | 8.77 | 9.31 | . |
| Domestic partner | 8.55 | 0.06 | 8.43 | 8.67 | . |
| Employment |  |  |  |  |  |
| Employed for an employer | 8.46 | 0.04 | 8.38 | 8.54 | 0.00 |
| Self-employed | 8.44 | 0.05 | 8.34 | 8.55 | . |
| Retired | 8.92 | 0.07 | 8.79 | 9.06 | . |
| Student | 7.88 | 0.12 | 7.64 | 8.12 | . |
| Homemaker | 8.70 | 0.07 | 8.56 | 8.84 | . |
| Unemployed and looking for a job | 8.29 | 0.07 | 8.16 | 8.42 | . |
| None of these/other | 8.26 | 0.17 | 7.92 | 8.60 | . |
| Education |  |  |  |  |  |
| Up to 8 years | 8.50 | 0.06 | 8.38 | 8.62 | 0.07 |
| 9-15 years | 8.43 | 0.03 | 8.36 | 8.49 | . |
| 16+ years | 8.55 | 0.05 | 8.47 | 8.64 | . |
| Religious service attendance |  |  |  |  |  |
| >1x/week | 8.91 | 0.05 | 8.81 | 9.02 | 0.00 |
| 1x/week | 8.66 | 0.05 | 8.55 | 8.77 | . |
| 1-3x/month | 8.62 | 0.06 | 8.49 | 8.74 | . |
| A few times a year | 8.48 | 0.04 | 8.40 | 8.57 | . |
| Never | 7.91 | 0.06 | 7.79 | 8.03 | . |
| Immigration status |  |  |  |  |  |
| Born in this country | 8.47 | 0.03 | 8.42 | 8.52 | 0.28 |
| Born in another country | 8.22 | 0.24 | 7.75 | 8.68 | . |
| Religion |  |  |  |  |  |
| Christianity | 8.59 | 0.03 | 8.54 | 8.64 | 0.00 |
| Islam | 7.33 | 1.15 | 4.75 | 9.92 | . |
| Hinduism | 8.60 | 0.57 | 2.67 | 14.54 | . |
| Buddhism | 8.30 | 0.35 | 7.59 | 9.01 | . |
| Judaism | 5.49 | 1.06 | 3.28 | 7.69 | . |
| Sikhism | . | . | . | . | . |
| Baha'i | 9.65 | 0.44 | 5.87 | 13.43 | . |
| Jainism | 7.70 | 0.55 | 5.33 | 10.07 | . |
| Shinto | 5.65 | 1.79 | -288.24 | 299.55 | . |
| Taoism | 8.41 | 0.88 | 4.64 | 12.17 | . |
| Confucianism | 8.93 | 1.12 | 4.10 | 13.76 | . |
| Primal, Animist, or Folk Religion | 8.04 | 0.82 | 6.29 | 9.79 | . |
| Spiritism | 8.65 | 0.09 | 8.47 | 8.82 | . |
| African-derived | 8.32 | 0.14 | 8.05 | 8.59 | . |
| Chinese | . | . | . | . | . |
| Some other religion | 8.21 | 0.24 | 7.74 | 8.68 | . |
| No religion/atheist/agnostic | 7.83 | 0.08 | 7.67 | 7.98 | . |
| Race/ethnicity |  |  |  |  |  |
| Branca | 8.51 | 0.04 | 8.43 | 8.58 | 0.51 |
| Preta | 8.45 | 0.07 | 8.31 | 8.60 | . |
| Parda | 8.46 | 0.04 | 8.39 | 8.54 | . |
| Amarela | 8.10 | 0.23 | 7.64 | 8.55 | . |
| Indigena | 8.36 | 0.24 | 7.90 | 8.83 | . |
| Other | 8.11 | 0.51 | 7.08 | 9.14 | . |

*Note*. *N* = 13,204. SE, standard error; LCI, lower confidence interval; UCI, upper confidence interval.

*Table 3c. Variations Across Childhood Predictors (Brazil)*

| Characteristic | Coef. | SE | Prob. | LCI | UCI | Global *p*-value |
| --- | --- | --- | --- | --- | --- | --- |
| Relationship with mother (Ref: Very/somewhat bad) |  |  |  |  |  |  |
| Very/somewhat good | 0.18 | 0.12 | 0.13 | -0.05 | 0.41 | 0.13 |
| Relationship with father (Ref: Very/somewhat bad) |  |  |  |  |  |  |
| Very/somewhat good | 0.18 | 0.08 | 0.02 | 0.03 | 0.33 | 0.02 |
| Parent marital status (Ref: Married) |  |  |  |  |  |  |
| Divorced | -0.16 | 0.09 | 0.07 | -0.33 | 0.01 | 0.19 |
| Never married | -0.14 | 0.09 | 0.14 | -0.33 | 0.05 | . |
| One or both parents had died | 0.02 | 0.14 | 0.91 | -0.26 | 0.30 | . |
| Subjective financial status growing up (Ref: Got by) |  |  |  |  |  |  |
| Lived comfortably | 0.01 | 0.06 | 0.82 | -0.10 | 0.13 | 0.61 |
| Found it difficult | -0.01 | 0.07 | 0.84 | -0.16 | 0.13 | . |
| Found it very difficult | 0.14 | 0.11 | 0.22 | -0.08 | 0.37 | . |
| Childhood abuse (Ref: No) |  |  |  |  |  |  |
| Yes | -0.04 | 0.07 | 0.59 | -0.17 | 0.10 | 0.59 |
| Outsider growing up (Ref: No) |  |  |  |  |  |  |
| Yes | -0.34 | 0.08 | 0.00 | -0.50 | -0.17 | 0.00 |
| Childhood health (Ref: Good) |  |  |  |  |  |  |
| Excellent | 0.37 | 0.07 | 0.00 | 0.24 | 0.50 | 0.00 |
| Very good | 0.06 | 0.07 | 0.40 | -0.08 | 0.20 | . |
| Fair | -0.13 | 0.11 | 0.24 | -0.34 | 0.08 | . |
| Poor | 0.35 | 0.20 | 0.07 | -0.03 | 0.74 | . |
| Immigration status (Ref: Born in this country) |  |  |  |  |  |  |
| Born in another country | -0.25 | 0.22 | 0.26 | -0.69 | 0.19 | 0.26 |
| Childhood religious service attendance (Ref: Never) |  |  |  |  |  |  |
| At least 1x/week | 0.37 | 0.09 | 0.00 | 0.19 | 0.55 | 0.00 |
| 1-3x/month | 0.19 | 0.10 | 0.06 | 0.00 | 0.39 | . |
| <1x/month | 0.17 | 0.10 | 0.09 | -0.02 | 0.37 | . |
| Gender (Ref: Male) |  |  |  |  |  |  |
| Female | 0.42 | 0.05 | 0.00 | 0.32 | 0.52 | 0.00 |
| Other | 0.06 | 0.57 | 0.92 | -1.06 | 1.18 | . |
| Birth year/current age (Ref: 1998-2005; age 18-24) |  |  |  |  |  |  |
| 1993-1998; age 25-29 | 0.23 | 0.10 | 0.02 | 0.03 | 0.43 | 0.00 |
| 1983-1993; age 30-39 | 0.38 | 0.09 | 0.00 | 0.21 | 0.55 | . |
| 1973-1983; age 40-49 | 0.65 | 0.09 | 0.00 | 0.47 | 0.82 | . |
| 1963-1973; age 50-59 | 0.78 | 0.10 | 0.00 | 0.59 | 0.97 | . |
| 1953-1963; age 60-69 | 0.88 | 0.11 | 0.00 | 0.67 | 1.10 | . |
| 1943-1953; age 70-79 | 1.27 | 0.13 | 0.00 | 1.01 | 1.52 | . |
| 1943 or earlier; age 80 or older | 0.93 | 0.28 | 0.00 | 0.39 | 1.48 | . |
| Mother absence/presence (Ref: Present) |  |  |  |  |  |  |
| Absent | 0.06 | 0.12 | 0.63 | -0.17 | 0.29 | 0.63 |
| Father absence/presence (Ref: Present) |  |  |  |  |  |  |
| Absent | -0.08 | 0.10 | 0.39 | -0.27 | 0.11 | 0.39 |
| Childhood religion (Ref: No religion/atheist/agnostic) |  |  |  |  |  |  |
| Christianity | 0.20 | 0.12 | 0.09 | -0.03 | 0.44 | 0.24 |
| Some other religion | 0.17 | 0.16 | 0.27 | -0.14 | 0.48 | . |
| Race/ethnicity (Ref: Ethnic plurality) |  |  |  |  |  |  |
| Ethnic minority | 0.10 | 0.06 | 0.07 | -0.01 | 0.21 | 0.07 |
| *Note*. *N* = 13,204. SE, standard error; LCI, lower confidence interval; UCI, upper confidence interval. | | | | | | |

*Table 3d. E-Values and E-Value Limits for the Coefficients Shown in Table 3c (Brazil)*

| Characteristic | *E*-value for estimate^a^ | *E*-value for 95% CI^b^ |
| --- | --- | --- |
| Relationship with mother (Ref: Very/somewhat bad) |  |  |
| Very/somewhat good | 1.30 | 1.00 |
| Relationship with father (Ref: Very/somewhat bad) |  |  |
| Very/somewhat good | 1.30 | 1.11 |
| Parent marital status (Ref: Married) |  |  |
| Divorced | 1.28 | 1.00 |
| Never married | 1.26 | 1.00 |
| One or both parents had died | 1.07 | 1.00 |
| Subjective financial status growing up (Ref: Got by) |  |  |
| Lived comfortably | 1.07 | 1.00 |
| Found it difficult | 1.07 | 1.00 |
| Found it very difficult | 1.26 | 1.00 |
| Childhood abuse (Ref: No) |  |  |
| Yes | 1.12 | 1.00 |
| Outsider growing up (Ref: No) |  |  |
| Yes | 1.46 | 1.29 |
| Childhood health (Ref: Good) |  |  |
| Excellent | 1.50 | 1.37 |
| Very good | 1.16 | 1.00 |
| Fair | 1.25 | 1.00 |
| Poor | 1.48 | 1.00 |
| Immigration status (Ref: Born in this country) |  |  |
| Born in another country | 1.38 | 1.00 |
| Childhood religious service attendance (Ref: Never) |  |  |
| At least 1x/week | 1.49 | 1.32 |
| 1-3x/month | 1.32 | 1.00 |
| <1x/month | 1.30 | 1.00 |
| Gender (Ref: Male) |  |  |
| Female | 1.54 | 1.44 |
| Other | 1.16 | 1.00 |
| Birth year/current age (Ref: 1998-2005; age 18-24) |  |  |
| 1993-1998; age 25-29 | 1.36 | 1.11 |
| 1983-1993; age 30-39 | 1.51 | 1.34 |
| 1973-1983; age 40-49 | 1.75 | 1.59 |
| 1963-1973; age 50-59 | 1.87 | 1.70 |
| 1953-1963; age 60-69 | 1.97 | 1.77 |
| 1943-1953; age 70-79 | 2.34 | 2.09 |
| 1943 or earlier; age 80 or older | 2.01 | 1.51 |
| Mother absence/presence (Ref: Present) |  |  |
| Absent | 1.15 | 1.00 |
| Father absence/presence (Ref: Present) |  |  |
| Absent | 1.19 | 1.00 |
| Childhood religion (Ref: No religion/atheist/agnostic) |  |  |
| Christianity | 1.33 | 1.00 |
| Some other religion | 1.30 | 1.00 |
| Race/ethnicity (Ref: Ethnic plurality) |  |  |
| Ethnic minority | 1.22 | 1.00 |

*Note*. CI, confidence interval. ^a^The formula for calculating *E*-values can be found in VanderWeele and Ding (2017) The *E*-value for the effect estimate is the minimum strength of association (on the risk ratio scale) that an unmeasured confounder would need to have with both the predictor and the outcome to entirely explain away the observed association between them, conditional on the measured covariates. ^b^The *E*-value for the limit of the 95% confidence interval closest to the null denote the minimum strength of association (on the risk ratio scale) that an unmeasured confounder would need to have with both the predictor and the outcome to shift the confidence interval to include the null value, conditional on the measured covariates.

*Table S4a. Nationally Representative Descriptive Statistics of the Observed Sample (Egypt)*

| Variable | Proportion | Frequency |
| --- | --- | --- |
| Sociodemographic characteristics |  |  |
| Birth year/current age |  |  |
| 1998-2005; age 18-24 | 0.20 | 960 |
| 1993-1998; age 25-29 | 0.13 | 607 |
| 1983-1993; age 30-39 | 0.25 | 1204 |
| 1973-1983; age 40-49 | 0.19 | 897 |
| 1963-1973; age 50-59 | 0.13 | 613 |
| 1953-1963; age 60-69 | 0.08 | 387 |
| 1943-1953; age 70-79 | 0.01 | 54 |
| 1943 or earlier; 80 or older | 0.00 | 7 |
| Missing | . | . |
| Gender |  |  |
| Male | 0.51 | 2394 |
| Female | 0.49 | 2334 |
| Other | . | . |
| Missing | 0.00 | 0 |
| Marital status |  |  |
| Single, never married | 0.20 | 947 |
| Married | 0.72 | 3387 |
| Separated | 0.01 | 39 |
| Divorced | 0.02 | 101 |
| Widowed | 0.05 | 238 |
| Domestic partner | . | . |
| Missing | 0.00 | 17 |
| Employment |  |  |
| Employed for an employer | 0.27 | 1267 |
| Self-employed | 0.19 | 892 |
| Retired | 0.05 | 253 |
| Student | 0.06 | 297 |
| Homemaker | 0.37 | 1772 |
| Unemployed and looking for a job | 0.05 | 224 |
| None of these/other | 0.00 | 21 |
| Missing | 0.00 | 3 |
| Education |  |  |
| Up to 8 years | 0.53 | 2486 |
| 9-15 years | 0.34 | 1599 |
| 16+ years | 0.14 | 643 |
| Missing | 0.00 | 1 |
| Religious service attendance |  |  |
| >1x/week | 0.18 | 839 |
| 1x/week | 0.20 | 960 |
| 1-3x/month | 0.08 | 368 |
| A few times a year | 0.10 | 458 |
| Never | 0.44 | 2091 |
| Missing | 0.00 | 12 |
| Immigration status |  |  |
| Born in this country | 1.00 | 4713 |
| Born in another country | 0.00 | 16 |
| Missing | 0.00 | 1 |
| Religion |  |  |
| Christianity | 0.03 | 120 |
| Islam | 0.97 | 4607 |
| Hinduism | . | . |
| Buddhism | . | . |
| Judaism | . | . |
| Sikhism | . | . |
| Baha'i | . | . |
| Jainism | . | . |
| Shinto | . | . |
| Taoism | 0.00 | 0 |
| Confucianism | . | . |
| Primal, Animist, or Folk Religion | . | . |
| Spiritism | . | . |
| African-derived | . | . |
| Chinese | . | . |
| Some other religion | . | . |
| No religion/atheist/agnostic | . | . |
| Missing | 0.00 | 1 |
| Race/ethnicity |  |  |
| Arab | 0.97 | 4585 |
| Turkish | 0.00 | 9 |
| Greek | 0.00 | 1 |
| Abazas | . | . |
| Bedouin Arab | 0.00 | 4 |
| Swiss | . | . |
| Nubian | 0.01 | 27 |
| Other | . | . |
| Missing | 0.02 | 102 |
| Childhood factors |  |  |
| Relationship with mother |  |  |
| Very good | 0.87 | 4110 |
| Somewhat good | 0.11 | 505 |
| Somewhat bad | 0.00 | 21 |
| Very bad | 0.00 | 10 |
| Not applicable | 0.02 | 83 |
| Missing | . | . |
| Relationship with father |  |  |
| Very good | 0.79 | 3713 |
| Somewhat good | 0.14 | 683 |
| Somewhat bad | 0.01 | 56 |
| Very bad | 0.01 | 30 |
| Not applicable | 0.05 | 233 |
| Missing | 0.00 | 14 |
| Parent marital status |  |  |
| Married | 0.86 | 4049 |
| Divorced | 0.03 | 131 |
| Never married | 0.00 | 9 |
| One or both parents had died | 0.10 | 485 |
| Missing | 0.01 | 55 |
| Subjective financial status growing up |  |  |
| Lived comfortably | 0.26 | 1251 |
| Got by | 0.50 | 2352 |
| Found it difficult | 0.18 | 857 |
| Found it very difficult | 0.06 | 268 |
| Missing | 0.00 | 1 |
| Childhood abuse |  |  |
| Yes | 0.09 | 405 |
| No | 0.91 | 4293 |
| Missing | 0.01 | 30 |
| Outsider growing up |  |  |
| Yes | 0.05 | 260 |
| No | 0.94 | 4456 |
| Not applicable | 0.00 | 4 |
| Missing | 0.00 | 10 |
| Childhood health |  |  |
| Excellent | 0.57 | 2687 |
| Very good | 0.25 | 1174 |
| Good | 0.11 | 497 |
| Fair | 0.06 | 265 |
| Poor | 0.02 | 106 |
| Missing | 0.00 | 1 |
| Childhood religious service attendance |  |  |
| At least 1x/week | 0.49 | 2307 |
| 1-3x/month | 0.12 | 570 |
| <1x/month | 0.13 | 629 |
| Never | 0.25 | 1165 |
| Missing | 0.01 | 57 |
| Childhood religion |  |  |
| Christianity | 0.03 | 123 |
| Islam | 0.97 | 4602 |
| Hinduism | . | . |
| Buddhism | . | . |
| Judaism | . | . |
| Sikhism | . | . |
| Baha'i | . | . |
| Jainism | 0.00 | 1 |
| Shinto | . | . |
| Taoism | 0.00 | 1 |
| Confucianism | . | . |
| Primal, Animist, or Folk Religion | . | . |
| Spiritism | . | . |
| African-derived | . | . |
| Chinese | . | . |
| Some other religion | . | . |
| No religion/atheist/agnostic | . | . |
| Missing | 0.00 | 3 |

*Note*. *N* = 4,729.

*Table S4b. Variations Across Sociodemographic Characteristics (Egypt)*

| Characteristic | Mean | SE | LCI | UCI | Global *p*-value |
| --- | --- | --- | --- | --- | --- |
| Age group |  |  |  |  |  |
| 18-24 | 7.82 | 0.13 | 7.55 | 8.08 | 0.00 |
| 25-29 | 8.12 | 0.15 | 7.82 | 8.41 | . |
| 30-39 | 8.34 | 0.08 | 8.18 | 8.50 | . |
| 40-49 | 8.52 | 0.09 | 8.35 | 8.70 | . |
| 50-59 | 8.52 | 0.11 | 8.29 | 8.75 | . |
| 60-69 | 8.55 | 0.15 | 8.24 | 8.86 | . |
| 70-79 | 7.66 | 0.58 | 6.47 | 8.85 | . |
| 80 or older | 5.55 | 1.56 | -250.98 | 262.08 | . |
| Gender |  |  |  |  |  |
| Male | 8.04 | 0.08 | 7.88 | 8.19 | 0.00 |
| Female | 8.51 | 0.06 | 8.39 | 8.63 | . |
| Other | . | . | . | . | . |
| Marital status |  |  |  |  |  |
| Single, never married | 7.63 | 0.14 | 7.36 | 7.91 | 0.00 |
| Married | 8.47 | 0.05 | 8.37 | 8.58 | . |
| Separated | 7.19 | 0.53 | 6.06 | 8.32 | . |
| Divorced | 7.62 | 0.39 | 6.83 | 8.41 | . |
| Widowed | 8.38 | 0.20 | 7.99 | 8.77 | . |
| Domestic partner | . | . | . | . | . |
| Employment |  |  |  |  |  |
| Employed for an employer | 8.08 | 0.12 | 7.85 | 8.32 | 0.00 |
| Self-employed | 8.19 | 0.11 | 7.96 | 8.41 | . |
| Retired | 8.15 | 0.22 | 7.72 | 8.58 | . |
| Student | 7.60 | 0.21 | 7.19 | 8.02 | . |
| Homemaker | 8.57 | 0.07 | 8.44 | 8.70 | . |
| Unemployed and looking for a job | 8.24 | 0.21 | 7.82 | 8.67 | . |
| None of these/other | 8.76 | 0.88 | 5.87 | 11.64 | . |
| Education |  |  |  |  |  |
| Up to 8 years | 8.31 | 0.07 | 8.17 | 8.44 | 0.18 |
| 9-15 years | 8.16 | 0.06 | 8.04 | 8.28 | . |
| 16+ years | 8.38 | 0.13 | 8.12 | 8.64 | . |
| Religious service attendance |  |  |  |  |  |
| >1x/week | 8.24 | 0.12 | 8.00 | 8.48 | 0.16 |
| 1x/week | 8.41 | 0.11 | 8.18 | 8.63 | . |
| 1-3x/month | 7.97 | 0.17 | 7.64 | 8.30 | . |
| A few times a year | 8.11 | 0.15 | 7.82 | 8.40 | . |
| Never | 8.31 | 0.07 | 8.17 | 8.44 | . |
| Immigration status |  |  |  |  |  |
| Born in this country | 8.27 | 0.05 | 8.17 | 8.37 | 0.64 |
| Born in another country | 8.55 | 0.53 | 7.04 | 10.07 | . |
| Religion |  |  |  |  |  |
| Christianity | 8.06 | 0.42 | 7.20 | 8.93 | 0.61 |
| Islam | 8.27 | 0.05 | 8.18 | 8.37 | . |
| Hinduism | . | . | . | . | . |
| Buddhism | . | . | . | . | . |
| Judaism | . | . | . | . | . |
| Sikhism | . | . | . | . | . |
| Baha'i | . | . | . | . | . |
| Jainism | . | . | . | . | . |
| Shinto | . | . | . | . | . |
| Taoism | 10.00 | . | . | . | . |
| Confucianism | . | . | . | . | . |
| Primal, Animist, or Folk Religion | . | . | . | . | . |
| Spiritism | . | . | . | . | . |
| African-derived | . | . | . | . | . |
| Chinese | . | . | . | . | . |
| Some other religion | . | . | . | . | . |
| No religion/atheist/agnostic | . | . | . | . | . |
| Race/ethnicity |  |  |  |  |  |
| Arab | 8.26 | 0.05 | 8.16 | 8.36 | 0.00 |
| Turkish | 9.42 | 0.30 | 8.41 | 10.44 | . |
| Greek | 10.00 | . | . | . | . |
| Abazas | . | . | . | . | . |
| Bedouin Arab | 6.29 | . | . | . | . |
| Swiss | . | . | . | . | . |
| Nubian | 8.96 | . | . | . | . |
| Other | . | . | . | . | . |

*Note*. *N* = 4,729. SE, standard error; LCI, lower confidence interval; UCI, upper confidence interval.

*Table 4c. Variations Across Childhood Predictors (Egypt)*

| Characteristic | Coef. | SE | Prob. | LCI | UCI | Global *p*-value |
| --- | --- | --- | --- | --- | --- | --- |
| Relationship with mother (Ref: Very/somewhat bad) |  |  |  |  |  |  |
| Very/somewhat good | 0.77 | 0.53 | 0.15 | -0.28 | 1.82 | 0.15 |
| Relationship with father (Ref: Very/somewhat bad) |  |  |  |  |  |  |
| Very/somewhat good | 0.04 | 0.27 | 0.88 | -0.50 | 0.58 | 0.88 |
| Parent marital status (Ref: Married) |  |  |  |  |  |  |
| Divorced | -0.06 | 0.33 | 0.85 | -0.72 | 0.60 | 0.54 |
| Never married | 0.48 | 0.78 | 0.54 | -1.09 | 2.05 | . |
| One or both parents had died | -0.24 | 0.17 | 0.15 | -0.57 | 0.09 | . |
| Subjective financial status growing up (Ref: Got by) |  |  |  |  |  |  |
| Lived comfortably | 0.12 | 0.13 | 0.33 | -0.13 | 0.37 | 0.65 |
| Found it difficult | -0.08 | 0.15 | 0.59 | -0.38 | 0.22 | . |
| Found it very difficult | 0.05 | 0.20 | 0.78 | -0.33 | 0.44 | . |
| Childhood abuse (Ref: No) |  |  |  |  |  |  |
| Yes | -0.06 | 0.17 | 0.73 | -0.40 | 0.28 | 0.73 |
| Outsider growing up (Ref: No) |  |  |  |  |  |  |
| Yes | -0.19 | 0.22 | 0.41 | -0.63 | 0.26 | 0.41 |
| Childhood health (Ref: Good) |  |  |  |  |  |  |
| Excellent | -0.03 | 0.16 | 0.87 | -0.34 | 0.29 | 0.37 |
| Very good | -0.04 | 0.17 | 0.81 | -0.37 | 0.29 | . |
| Fair | -0.51 | 0.27 | 0.06 | -1.04 | 0.02 | . |
| Poor | -0.10 | 0.47 | 0.83 | -1.04 | 0.83 | . |
| Immigration status (Ref: Born in this country) |  |  |  |  |  |  |
| Born in another country | 0.19 | 0.63 | 0.77 | -1.06 | 1.44 | 0.77 |
| Childhood religious service attendance (Ref: Never) |  |  |  |  |  |  |
| At least 1x/week | 0.18 | 0.12 | 0.15 | -0.06 | 0.42 | 0.14 |
| 1-3x/month | 0.02 | 0.15 | 0.88 | -0.27 | 0.32 | . |
| <1x/month | 0.30 | 0.14 | 0.03 | 0.03 | 0.57 | . |
| Gender (Ref: Male) |  |  |  |  |  |  |
| Female | 0.52 | 0.10 | 0.00 | 0.32 | 0.71 | 0.00 |
| Other | . | . | . | . | . | . |
| Birth year/current age (Ref: 1998-2005; age 18-24) |  |  |  |  |  |  |
| 1993-1998; age 25-29 | 0.30 | 0.19 | 0.11 | -0.07 | 0.68 | 0.00 |
| 1983-1993; age 30-39 | 0.54 | 0.15 | 0.00 | 0.24 | 0.84 | . |
| 1973-1983; age 40-49 | 0.74 | 0.15 | 0.00 | 0.44 | 1.04 | . |
| 1963-1973; age 50-59 | 0.75 | 0.19 | 0.00 | 0.38 | 1.12 | . |
| 1953-1963; age 60-69 | 0.79 | 0.20 | 0.00 | 0.39 | 1.18 | . |
| 1943-1953; age 70-79 | -0.12 | 0.64 | 0.85 | -1.39 | 1.15 | . |
| 1943 or earlier; age 80 or older | -2.25 | 1.48 | 0.13 | -5.18 | 0.68 | . |
| Mother absence/presence (Ref: Present) |  |  |  |  |  |  |
| Absent | 0.63 | 0.46 | 0.18 | -0.29 | 1.55 | 0.18 |
| Father absence/presence (Ref: Present) |  |  |  |  |  |  |
| Absent | 0.17 | 0.35 | 0.63 | -0.53 | 0.86 | 0.63 |
| Childhood religion (Ref: Islam) |  |  |  |  |  |  |
| Some other religion | -0.18 | 0.39 | 0.64 | -0.95 | 0.59 | 0.64 |
| Race/ethnicity (Ref: Ethnic plurality) |  |  |  |  |  |  |
| Ethnic minority | 0.71 | 0.25 | 0.01 | 0.22 | 1.21 | 0.01 |
| *Note*. *N* = 4,729. SE, standard error; LCI, lower confidence interval; UCI, upper confidence interval. | | | | | | |

*Table 4d. E-Values and E-Value Limits for the Coefficients Shown in Table 4c (Egypt)*

| Characteristic | *E*-value for estimate^a^ | *E*-value for 95% CI^b^ |
| --- | --- | --- |
| Relationship with mother (Ref: Very/somewhat bad) |  |  |
| Very/somewhat good | 1.87 | 1.00 |
| Relationship with father (Ref: Very/somewhat bad) |  |  |
| Very/somewhat good | 1.13 | 1.00 |
| Parent marital status (Ref: Married) |  |  |
| Divorced | 1.16 | 1.00 |
| Never married | 1.60 | 1.00 |
| One or both parents had died | 1.37 | 1.00 |
| Subjective financial status growing up (Ref: Got by) |  |  |
| Lived comfortably | 1.24 | 1.00 |
| Found it difficult | 1.19 | 1.00 |
| Found it very difficult | 1.15 | 1.00 |
| Childhood abuse (Ref: No) |  |  |
| Yes | 1.16 | 1.00 |
| Outsider growing up (Ref: No) |  |  |
| Yes | 1.31 | 1.00 |
| Childhood health (Ref: Good) |  |  |
| Excellent | 1.10 | 1.00 |
| Very good | 1.13 | 1.00 |
| Fair | 1.63 | 1.00 |
| Poor | 1.22 | 1.00 |
| Immigration status (Ref: Born in this country) |  |  |
| Born in another country | 1.32 | 1.00 |
| Childhood religious service attendance (Ref: Never) |  |  |
| At least 1x/week | 1.30 | 1.00 |
| 1-3x/month | 1.09 | 1.00 |
| <1x/month | 1.43 | 1.11 |
| Gender (Ref: Male) |  |  |
| Female | 1.63 | 1.45 |
| Other | . | . |
| Birth year/current age (Ref: 1998-2005; age 18-24) |  |  |
| 1993-1998; age 25-29 | 1.44 | 1.00 |
| 1983-1993; age 30-39 | 1.66 | 1.37 |
| 1973-1983; age 40-49 | 1.84 | 1.57 |
| 1963-1973; age 50-59 | 1.85 | 1.52 |
| 1953-1963; age 60-69 | 1.89 | 1.53 |
| 1943-1953; age 70-79 | 1.24 | 1.00 |
| 1943 or earlier; age 80 or older | 3.49 | 1.00 |
| Mother absence/presence (Ref: Present) |  |  |
| Absent | 1.74 | 1.00 |
| Father absence/presence (Ref: Present) |  |  |
| Absent | 1.30 | 1.00 |
| Childhood religion (Ref: Islam) |  |  |
| Some other religion | 1.31 | 1.00 |
| Race/ethnicity (Ref: Ethnic plurality) |  |  |
| Ethnic minority | 1.82 | 1.35 |

*Note*. CI, confidence interval. ^a^The formula for calculating *E*-values can be found in VanderWeele and Ding (2017) The *E*-value for the effect estimate is the minimum strength of association (on the risk ratio scale) that an unmeasured confounder would need to have with both the predictor and the outcome to entirely explain away the observed association between them, conditional on the measured covariates. ^b^The *E*-value for the limit of the 95% confidence interval closest to the null denote the minimum strength of association (on the risk ratio scale) that an unmeasured confounder would need to have with both the predictor and the outcome to shift the confidence interval to include the null value, conditional on the measured covariates.

*Table S5a. Nationally Representative Descriptive Statistics of the Observed Sample (Germany)*

| Variable | Proportion | Frequency |
| --- | --- | --- |
| Sociodemographic characteristics |  |  |
| Birth year/current age |  |  |
| 1998-2005; age 18-24 | 0.09 | 829 |
| 1993-1998; age 25-29 | 0.08 | 774 |
| 1983-1993; age 30-39 | 0.15 | 1438 |
| 1973-1983; age 40-49 | 0.16 | 1494 |
| 1963-1973; age 50-59 | 0.18 | 1729 |
| 1953-1963; age 60-69 | 0.20 | 1915 |
| 1943-1953; age 70-79 | 0.12 | 1137 |
| 1943 or earlier; 80 or older | 0.02 | 190 |
| Missing | . | . |
| Gender |  |  |
| Male | 0.49 | 4641 |
| Female | 0.51 | 4843 |
| Other | 0.00 | 11 |
| Missing | 0.00 | 11 |
| Marital status |  |  |
| Single, never married | 0.28 | 2627 |
| Married | 0.50 | 4784 |
| Separated | 0.02 | 219 |
| Divorced | 0.08 | 767 |
| Widowed | 0.04 | 409 |
| Domestic partner | 0.07 | 619 |
| Missing | 0.01 | 81 |
| Employment |  |  |
| Employed for an employer | 0.52 | 4950 |
| Self-employed | 0.07 | 712 |
| Retired | 0.26 | 2480 |
| Student | 0.06 | 605 |
| Homemaker | 0.03 | 251 |
| Unemployed and looking for a job | 0.03 | 288 |
| None of these/other | 0.02 | 204 |
| Missing | 0.00 | 14 |
| Education |  |  |
| Up to 8 years | 0.02 | 235 |
| 9-15 years | 0.64 | 6094 |
| 16+ years | 0.33 | 3164 |
| Missing | 0.00 | 13 |
| Religious service attendance |  |  |
| >1x/week | 0.03 | 285 |
| 1x/week | 0.04 | 424 |
| 1-3x/month | 0.06 | 550 |
| A few times a year | 0.25 | 2362 |
| Never | 0.62 | 5876 |
| Missing | 0.00 | 9 |
| Immigration status |  |  |
| Born in this country | 0.92 | 8722 |
| Born in another country | 0.08 | 744 |
| Missing | 0.00 | 40 |
| Religion |  |  |
| Christianity | 0.53 | 5052 |
| Islam | 0.04 | 351 |
| Hinduism | 0.00 | 12 |
| Buddhism | 0.01 | 51 |
| Judaism | 0.00 | 19 |
| Sikhism | 0.00 | 5 |
| Baha'i | 0.00 | 3 |
| Jainism | . | . |
| Shinto | 0.00 | 2 |
| Taoism | 0.00 | 0 |
| Confucianism | 0.00 | 4 |
| Primal, Animist, or Folk Religion | 0.00 | 34 |
| Spiritism | . | . |
| African-derived | . | . |
| Chinese | . | . |
| Some other religion | 0.01 | 60 |
| No religion/atheist/agnostic | 0.40 | 3815 |
| Missing | 0.01 | 99 |
| Race/ethnicity |  |  |
| No data | . | . |
| Childhood factors |  |  |
| Relationship with mother |  |  |
| Very good | 0.58 | 5497 |
| Somewhat good | 0.32 | 3031 |
| Somewhat bad | 0.05 | 496 |
| Very bad | 0.02 | 187 |
| Not applicable | 0.03 | 241 |
| Missing | 0.01 | 54 |
| Relationship with father |  |  |
| Very good | 0.49 | 4652 |
| Somewhat good | 0.32 | 3012 |
| Somewhat bad | 0.09 | 846 |
| Very bad | 0.04 | 385 |
| Not applicable | 0.06 | 538 |
| Missing | 0.01 | 73 |
| Parent marital status |  |  |
| Married | 0.80 | 7620 |
| Divorced | 0.10 | 927 |
| Never married | 0.06 | 578 |
| One or both parents had died | 0.03 | 245 |
| Missing | 0.01 | 136 |
| Subjective financial status growing up |  |  |
| Lived comfortably | 0.33 | 3177 |
| Got by | 0.47 | 4508 |
| Found it difficult | 0.16 | 1481 |
| Found it very difficult | 0.03 | 314 |
| Missing | 0.00 | 26 |
| Childhood abuse |  |  |
| Yes | 0.11 | 1086 |
| No | 0.88 | 8321 |
| Missing | 0.01 | 99 |
| Outsider growing up |  |  |
| Yes | 0.12 | 1105 |
| No | 0.87 | 8262 |
| Not applicable | 0.01 | 114 |
| Missing | 0.00 | 25 |
| Childhood health |  |  |
| Excellent | 0.28 | 2633 |
| Very good | 0.37 | 3518 |
| Good | 0.27 | 2582 |
| Fair | 0.06 | 612 |
| Poor | 0.01 | 134 |
| Missing | 0.00 | 26 |
| Childhood religious service attendance |  |  |
| At least 1x/week | 0.20 | 1943 |
| 1-3x/month | 0.20 | 1899 |
| <1x/month | 0.30 | 2887 |
| Never | 0.29 | 2749 |
| Missing | 0.00 | 27 |
| Childhood religion |  |  |
| Christianity | 0.61 | 5751 |
| Islam | 0.04 | 350 |
| Hinduism | 0.00 | 15 |
| Buddhism | 0.00 | 25 |
| Judaism | 0.00 | 18 |
| Sikhism | 0.00 | 5 |
| Baha'i | 0.00 | 2 |
| Jainism | 0.00 | 1 |
| Shinto | . | . |
| Taoism | . | . |
| Confucianism | 0.00 | 4 |
| Primal, Animist, or Folk Religion | 0.00 | 19 |
| Spiritism | . | . |
| African-derived | . | . |
| Chinese | . | . |
| Some other religion | 0.01 | 67 |
| No religion/atheist/agnostic | 0.33 | 3163 |
| Missing | 0.01 | 85 |

*Note. N* = 9,506.

*Table S5b. Variations Across Sociodemographic Characteristics (Germany)*

| Characteristic | Mean | SE | LCI | UCI | Global *p*-value |
| --- | --- | --- | --- | --- | --- |
| Age group |  |  |  |  |  |
| 18-24 | 7.22 | 0.11 | 7.00 | 7.43 | 0.00 |
| 25-29 | 7.67 | 0.09 | 7.50 | 7.85 | . |
| 30-39 | 7.61 | 0.06 | 7.49 | 7.74 | . |
| 40-49 | 7.79 | 0.07 | 7.66 | 7.91 | . |
| 50-59 | 7.81 | 0.06 | 7.70 | 7.92 | . |
| 60-69 | 8.02 | 0.05 | 7.92 | 8.13 | . |
| 70-79 | 8.24 | 0.08 | 8.09 | 8.39 | . |
| 80 or older | 8.07 | 0.16 | 7.75 | 8.39 | . |
| Gender |  |  |  |  |  |
| Male | 7.64 | 0.04 | 7.57 | 7.72 | 0.00 |
| Female | 7.98 | 0.04 | 7.91 | 8.05 | . |
| Other | 5.27 | 0.41 | 4.39 | 6.16 | . |
| Marital status |  |  |  |  |  |
| Single, never married | 7.25 | 0.06 | 7.14 | 7.37 | 0.00 |
| Married | 8.06 | 0.03 | 8.00 | 8.13 | . |
| Separated | 7.62 | 0.21 | 7.21 | 8.03 | . |
| Divorced | 8.04 | 0.08 | 7.87 | 8.20 | . |
| Widowed | 8.15 | 0.12 | 7.91 | 8.38 | . |
| Domestic partner | 7.84 | 0.10 | 7.65 | 8.02 | . |
| Employment |  |  |  |  |  |
| Employed for an employer | 7.77 | 0.04 | 7.70 | 7.84 | 0.00 |
| Self-employed | 7.88 | 0.10 | 7.68 | 8.08 | . |
| Retired | 8.04 | 0.05 | 7.94 | 8.14 | . |
| Student | 7.38 | 0.12 | 7.15 | 7.62 | . |
| Homemaker | 7.96 | 0.14 | 7.70 | 8.23 | . |
| Unemployed and looking for a job | 7.41 | 0.15 | 7.12 | 7.71 | . |
| None of these/other | 7.48 | 0.18 | 7.12 | 7.84 | . |
| Education |  |  |  |  |  |
| Up to 8 years | 7.40 | 0.20 | 7.01 | 7.79 | 0.02 |
| 9-15 years | 7.79 | 0.03 | 7.73 | 7.86 | . |
| 16+ years | 7.89 | 0.04 | 7.81 | 7.97 | . |
| Religious service attendance |  |  |  |  |  |
| >1x/week | 7.89 | 0.15 | 7.58 | 8.19 | 0.00 |
| 1x/week | 7.86 | 0.12 | 7.62 | 8.10 | . |
| 1-3x/month | 8.04 | 0.11 | 7.83 | 8.26 | . |
| A few times a year | 8.02 | 0.05 | 7.92 | 8.11 | . |
| Never | 7.70 | 0.03 | 7.64 | 7.77 | . |
| Immigration status |  |  |  |  |  |
| Born in this country | 7.82 | 0.03 | 7.77 | 7.88 | 0.25 |
| Born in another country | 7.71 | 0.09 | 7.52 | 7.90 | . |
| Religion |  |  |  |  |  |
| Christianity | 7.95 | 0.03 | 7.89 | 8.02 | 0.00 |
| Islam | 7.42 | 0.17 | 7.09 | 7.75 | . |
| Hinduism | 7.11 | 0.35 | 5.60 | 8.63 | . |
| Buddhism | 8.13 | 0.17 | 7.78 | 8.48 | . |
| Judaism | 8.58 | 0.36 | 7.52 | 9.63 | . |
| Sikhism | 8.47 | 1.40 | -221.10 | 238.05 | . |
| Baha'i | 9.29 | . | . | . | . |
| Jainism | . | . | . | . | . |
| Shinto | 7.00 | . | . | . | . |
| Taoism | 10.00 | . | . | . | . |
| Confucianism | 3.86 | . | . | . | . |
| Primal, Animist, or Folk Religion | 7.43 | 0.35 | 6.69 | 8.18 | . |
| Spiritism | . | . | . | . | . |
| African-derived | . | . | . | . | . |
| Chinese | . | . | . | . | . |
| Some other religion | 7.44 | 0.40 | 6.64 | 8.25 | . |
| No religion/atheist/agnostic | 7.67 | 0.04 | 7.59 | 7.76 | . |
| Race/ethnicity |  |  |  |  |  |
| No data | . | . | . | . | . |

*Note*. *N* = 9,506. SE, standard error; LCI, lower confidence interval; UCI, upper confidence interval.

*Table 5c. Variations Across Childhood Predictors (Germany)*

| Characteristic | Coef. | SE | Prob. | LCI | UCI | Global *p*-value |
| --- | --- | --- | --- | --- | --- | --- |
| Relationship with mother (Ref: Very/somewhat bad) |  |  |  |  |  |  |
| Very/somewhat good | 0.24 | 0.11 | 0.02 | 0.03 | 0.45 | 0.02 |
| Relationship with father (Ref: Very/somewhat bad) |  |  |  |  |  |  |
| Very/somewhat good | 0.06 | 0.09 | 0.49 | -0.11 | 0.23 | 0.49 |
| Parent marital status (Ref: Married) |  |  |  |  |  |  |
| Divorced | -0.05 | 0.09 | 0.60 | -0.23 | 0.13 | 0.77 |
| Never married | -0.11 | 0.11 | 0.32 | -0.32 | 0.10 | . |
| One or both parents had died | -0.04 | 0.18 | 0.83 | -0.39 | 0.32 | . |
| Subjective financial status growing up (Ref: Got by) |  |  |  |  |  |  |
| Lived comfortably | 0.08 | 0.06 | 0.19 | -0.04 | 0.20 | 0.46 |
| Found it difficult | -0.05 | 0.08 | 0.51 | -0.21 | 0.11 | . |
| Found it very difficult | -0.03 | 0.16 | 0.83 | -0.34 | 0.27 | . |
| Childhood abuse (Ref: No) |  |  |  |  |  |  |
| Yes | -0.05 | 0.08 | 0.55 | -0.21 | 0.11 | 0.55 |
| Outsider growing up (Ref: No) |  |  |  |  |  |  |
| Yes | -0.30 | 0.09 | 0.00 | -0.47 | -0.13 | 0.00 |
| Childhood health (Ref: Good) |  |  |  |  |  |  |
| Excellent | 0.45 | 0.08 | 0.00 | 0.30 | 0.60 | 0.00 |
| Very good | 0.19 | 0.07 | 0.00 | 0.06 | 0.32 | . |
| Fair | 0.04 | 0.12 | 0.74 | -0.19 | 0.27 | . |
| Poor | 0.33 | 0.25 | 0.18 | -0.15 | 0.81 | . |
| Immigration status (Ref: Born in this country) |  |  |  |  |  |  |
| Born in another country | 0.16 | 0.10 | 0.11 | -0.04 | 0.35 | 0.11 |
| Childhood religious service attendance (Ref: Never) |  |  |  |  |  |  |
| At least 1x/week | 0.21 | 0.08 | 0.01 | 0.05 | 0.37 | 0.08 |
| 1-3x/month | 0.10 | 0.08 | 0.17 | -0.05 | 0.25 | . |
| <1x/month | 0.07 | 0.07 | 0.31 | -0.07 | 0.21 | . |
| Gender (Ref: Male) |  |  |  |  |  |  |
| Female | 0.40 | 0.05 | 0.00 | 0.30 | 0.50 | 0.00 |
| Other | -1.59 | 0.84 | 0.06 | -3.23 | 0.05 | . |
| Birth year/current age (Ref: 1998-2005; age 18-24) |  |  |  |  |  |  |
| 1993-1998; age 25-29 | 0.44 | 0.14 | 0.00 | 0.16 | 0.71 | 0.00 |
| 1983-1993; age 30-39 | 0.36 | 0.12 | 0.00 | 0.12 | 0.60 | . |
| 1973-1983; age 40-49 | 0.56 | 0.13 | 0.00 | 0.31 | 0.80 | . |
| 1963-1973; age 50-59 | 0.57 | 0.12 | 0.00 | 0.33 | 0.81 | . |
| 1953-1963; age 60-69 | 0.80 | 0.12 | 0.00 | 0.56 | 1.05 | . |
| 1943-1953; age 70-79 | 0.99 | 0.13 | 0.00 | 0.73 | 1.25 | . |
| 1943 or earlier; age 80 or older | 0.83 | 0.20 | 0.00 | 0.43 | 1.23 | . |
| Mother absence/presence (Ref: Present) |  |  |  |  |  |  |
| Absent | -0.14 | 0.14 | 0.33 | -0.42 | 0.14 | 0.33 |
| Father absence/presence (Ref: Present) |  |  |  |  |  |  |
| Absent | -0.01 | 0.12 | 0.92 | -0.24 | 0.22 | 0.92 |
| Childhood religion (Ref: No religion/atheist/agnostic) |  |  |  |  |  |  |
| Christianity | 0.26 | 0.06 | 0.00 | 0.14 | 0.38 | 0.00 |
| Islam | -0.20 | 0.17 | 0.25 | -0.53 | 0.14 | . |
| Some other religion | -0.07 | 0.24 | 0.76 | -0.55 | 0.40 | . |
| *Note*. *N* = 9,506. SE, standard error; LCI, lower confidence interval; UCI, upper confidence interval. | | | | | | |

*Table 5d. E-Values and E-Value Limits for the Coefficients Shown in Table 5c (Germany)*

| Characteristic | *E*-value for estimate^a^ | *E*-value for 95% CI^b^ |
| --- | --- | --- |
| Relationship with mother (Ref: Very/somewhat bad) |  |  |
| Very/somewhat good | 1.38 | 1.11 |
| Relationship with father (Ref: Very/somewhat bad) |  |  |
| Very/somewhat good | 1.16 | 1.00 |
| Parent marital status (Ref: Married) |  |  |
| Divorced | 1.14 | 1.00 |
| Never married | 1.23 | 1.00 |
| One or both parents had died | 1.12 | 1.00 |
| Subjective financial status growing up (Ref: Got by) |  |  |
| Lived comfortably | 1.19 | 1.00 |
| Found it difficult | 1.15 | 1.00 |
| Found it very difficult | 1.12 | 1.00 |
| Childhood abuse (Ref: No) |  |  |
| Yes | 1.14 | 1.00 |
| Outsider growing up (Ref: No) |  |  |
| Yes | 1.44 | 1.25 |
| Childhood health (Ref: Good) |  |  |
| Excellent | 1.58 | 1.44 |
| Very good | 1.32 | 1.16 |
| Fair | 1.13 | 1.00 |
| Poor | 1.47 | 1.00 |
| Immigration status (Ref: Born in this country) |  |  |
| Born in another country | 1.29 | 1.00 |
| Childhood religious service attendance (Ref: Never) |  |  |
| At least 1x/week | 1.34 | 1.15 |
| 1-3x/month | 1.22 | 1.00 |
| <1x/month | 1.18 | 1.00 |
| Gender (Ref: Male) |  |  |
| Female | 1.54 | 1.44 |
| Other | 2.75 | 1.00 |
| Birth year/current age (Ref: 1998-2005; age 18-24) |  |  |
| 1993-1998; age 25-29 | 1.57 | 1.30 |
| 1983-1993; age 30-39 | 1.50 | 1.24 |
| 1973-1983; age 40-49 | 1.69 | 1.45 |
| 1963-1973; age 50-59 | 1.70 | 1.47 |
| 1953-1963; age 60-69 | 1.92 | 1.69 |
| 1943-1953; age 70-79 | 2.11 | 1.85 |
| 1943 or earlier; age 80 or older | 1.95 | 1.57 |
| Mother absence/presence (Ref: Present) |  |  |
| Absent | 1.27 | 1.00 |
| Father absence/presence (Ref: Present) |  |  |
| Absent | 1.06 | 1.00 |
| Childhood religion (Ref: No religion/atheist/agnostic) |  |  |
| Christianity | 1.40 | 1.27 |
| Islam | 1.33 | 1.00 |
| Some other religion | 1.18 | 1.00 |

*Note*. CI, confidence interval. ^a^The formula for calculating *E*-values can be found in VanderWeele and Ding (2017) The *E*-value for the effect estimate is the minimum strength of association (on the risk ratio scale) that an unmeasured confounder would need to have with both the predictor and the outcome to entirely explain away the observed association between them, conditional on the measured covariates. ^b^The *E*-value for the limit of the 95% confidence interval closest to the null denote the minimum strength of association (on the risk ratio scale) that an unmeasured confounder would need to have with both the predictor and the outcome to shift the confidence interval to include the null value, conditional on the measured covariates.

*Table S6a. Nationally Representative Descriptive Statistics of the Observed Sample (Hong Kong)*

| Variable | Proportion | Frequency |
| --- | --- | --- |
| Sociodemographic characteristics |  |  |
| Birth year/current age |  |  |
| 1998-2005; age 18-24 | 0.07 | 217 |
| 1993-1998; age 25-29 | 0.07 | 198 |
| 1983-1993; age 30-39 | 0.17 | 507 |
| 1973-1983; age 40-49 | 0.19 | 580 |
| 1963-1973; age 50-59 | 0.24 | 711 |
| 1953-1963; age 60-69 | 0.21 | 620 |
| 1943-1953; age 70-79 | 0.05 | 164 |
| 1943 or earlier; 80 or older | 0.00 | 15 |
| Missing | . | . |
| Gender |  |  |
| Male | 0.46 | 1390 |
| Female | 0.54 | 1620 |
| Other | 0.00 | 2 |
| Missing | . | . |
| Marital status |  |  |
| Single, never married | 0.24 | 723 |
| Married | 0.69 | 2080 |
| Separated | 0.01 | 21 |
| Divorced | 0.03 | 105 |
| Widowed | 0.01 | 45 |
| Domestic partner | 0.01 | 37 |
| Missing | 0.00 | 1 |
| Employment |  |  |
| Employed for an employer | 0.68 | 2056 |
| Self-employed | 0.08 | 245 |
| Retired | 0.14 | 423 |
| Student | 0.02 | 55 |
| Homemaker | 0.04 | 114 |
| Unemployed and looking for a job | 0.02 | 62 |
| None of these/other | 0.01 | 39 |
| Missing | 0.01 | 18 |
| Education |  |  |
| Up to 8 years | 0.14 | 433 |
| 9-15 years | 0.67 | 2031 |
| 16+ years | 0.18 | 547 |
| Missing | . | . |
| Religious service attendance |  |  |
| >1x/week | 0.08 | 237 |
| 1x/week | 0.19 | 567 |
| 1-3x/month | 0.11 | 332 |
| A few times a year | 0.18 | 543 |
| Never | 0.44 | 1332 |
| Missing | 0.00 | 1 |
| Immigration status |  |  |
| Born in this country | 0.88 | 2637 |
| Born in another country | 0.11 | 321 |
| Missing | 0.02 | 53 |
| Religion |  |  |
| Christianity | 0.25 | 757 |
| Islam | 0.03 | 86 |
| Hinduism | 0.01 | 20 |
| Buddhism | 0.12 | 349 |
| Judaism | 0.00 | 10 |
| Sikhism | 0.00 | 2 |
| Baha'i | 0.00 | 3 |
| Jainism | 0.00 | 1 |
| Shinto | 0.01 | 19 |
| Taoism | 0.03 | 97 |
| Confucianism | 0.00 | 11 |
| Primal, Animist, or Folk Religion | 0.01 | 27 |
| Spiritism | . | . |
| African-derived | . | . |
| Chinese | 0.04 | 106 |
| Some other religion | 0.00 | 4 |
| No religion/atheist/agnostic | 0.50 | 1518 |
| Missing | 0.00 | 5 |
| Race/ethnicity |  |  |
| Chinese (Cantonese) | 0.64 | 1930 |
| Chinese (Chaoshan) | 0.07 | 201 |
| Chinese (Fujianese) | 0.04 | 117 |
| Chinese (Hakka) | 0.04 | 121 |
| Chinese (Shanghainese) | 0.03 | 89 |
| Chinese (other ethnicity) | 0.09 | 264 |
| East Asian (Korean, Japanese) | 0.00 | 10 |
| Southeast Asian (Filipino, Indonesian, Thailand) | 0.02 | 46 |
| South Asian (Indian, Nepalese, Pakistani) | 0.01 | 17 |
| Taiwanese | 0.00 | 14 |
| White | 0.00 | 15 |
| Other | 0.00 | 4 |
| Missing | 0.06 | 184 |
| Childhood factors |  |  |
| Relationship with mother |  |  |
| Very good | 0.36 | 1077 |
| Somewhat good | 0.39 | 1164 |
| Somewhat bad | 0.10 | 293 |
| Very bad | 0.02 | 49 |
| Not applicable | 0.14 | 426 |
| Missing | 0.00 | 3 |
| Relationship with father |  |  |
| Very good | 0.29 | 868 |
| Somewhat good | 0.36 | 1089 |
| Somewhat bad | 0.13 | 393 |
| Very bad | 0.03 | 102 |
| Not applicable | 0.19 | 557 |
| Missing | 0.00 | 3 |
| Parent marital status |  |  |
| Married | 0.91 | 2752 |
| Divorced | 0.04 | 114 |
| Never married | 0.01 | 40 |
| One or both parents had died | 0.02 | 50 |
| Missing | 0.02 | 56 |
| Subjective financial status growing up |  |  |
| Lived comfortably | 0.30 | 906 |
| Got by | 0.51 | 1527 |
| Found it difficult | 0.16 | 473 |
| Found it very difficult | 0.03 | 84 |
| Missing | 0.01 | 22 |
| Childhood abuse |  |  |
| Yes | 0.11 | 318 |
| No | 0.89 | 2688 |
| Missing | 0.00 | 5 |
| Outsider growing up |  |  |
| Yes | 0.22 | 664 |
| No | 0.74 | 2224 |
| Not applicable | 0.04 | 110 |
| Missing | 0.00 | 14 |
| Childhood health |  |  |
| Excellent | 0.18 | 545 |
| Very good | 0.36 | 1073 |
| Good | 0.29 | 863 |
| Fair | 0.14 | 426 |
| Poor | 0.03 | 91 |
| Missing | 0.00 | 13 |
| Childhood religious service attendance |  |  |
| At least 1x/week | 0.14 | 432 |
| 1-3x/month | 0.18 | 528 |
| <1x/month | 0.25 | 753 |
| Never | 0.43 | 1295 |
| Missing | 0.00 | 4 |
| Childhood religion |  |  |
| Christianity | 0.24 | 715 |
| Islam | 0.03 | 86 |
| Hinduism | 0.01 | 27 |
| Buddhism | 0.11 | 323 |
| Judaism | 0.01 | 16 |
| Sikhism | 0.00 | 4 |
| Baha'i | . | . |
| Jainism | 0.00 | 1 |
| Shinto | 0.01 | 18 |
| Taoism | 0.03 | 81 |
| Confucianism | 0.00 | 10 |
| Primal, Animist, or Folk Religion | 0.00 | 15 |
| Spiritism | . | . |
| African-derived | . | . |
| Chinese | 0.04 | 108 |
| Some other religion | 0.00 | 5 |
| No religion/atheist/agnostic | 0.53 | 1601 |
| Missing | 0.00 | 1 |

*Note. N* = 3,012.

*Table S6b. Variations Across Sociodemographic Characteristics (Hong Kong)*

| Characteristic | Mean | SE | LCI | UCI | Global *p*-value |
| --- | --- | --- | --- | --- | --- |
| Age group |  |  |  |  |  |
| 18-24 | 6.68 | 0.13 | 6.42 | 6.95 | 0.00 |
| 25-29 | 6.44 | 0.26 | 5.93 | 6.96 | . |
| 30-39 | 5.95 | 0.13 | 5.70 | 6.20 | . |
| 40-49 | 6.36 | 0.12 | 6.12 | 6.60 | . |
| 50-59 | 6.84 | 0.11 | 6.63 | 7.04 | . |
| 60-69 | 6.70 | 0.19 | 6.33 | 7.07 | . |
| 70-79 | 6.55 | 0.37 | 5.80 | 7.31 | . |
| 80 or older | 8.00 | . | . | . | . |
| Gender |  |  |  |  |  |
| Male | 6.53 | 0.09 | 6.35 | 6.70 | 0.02 |
| Female | 6.52 | 0.09 | 6.35 | 6.69 | . |
| Other | 5.55 | 0.50 | -76.03 | 87.12 | . |
| Marital status |  |  |  |  |  |
| Single, never married | 5.64 | 0.11 | 5.42 | 5.86 | 0.00 |
| Married | 6.92 | 0.07 | 6.77 | 7.06 | . |
| Separated | 5.71 | 0.86 | 3.58 | 7.84 | . |
| Divorced | 6.36 | 0.39 | 5.57 | 7.14 | . |
| Widowed | 4.37 | 0.96 | 2.36 | 6.38 | . |
| Domestic partner | 5.11 | 0.39 | 4.32 | 5.89 | . |
| Employment |  |  |  |  |  |
| Employed for an employer | 6.58 | 0.06 | 6.45 | 6.70 | 0.00 |
| Self-employed | 7.31 | 0.22 | 6.87 | 7.75 | . |
| Retired | 6.39 | 0.23 | 5.94 | 6.85 | . |
| Student | 6.32 | 0.26 | 5.79 | 6.85 | . |
| Homemaker | 5.71 | 0.35 | 5.01 | 6.41 | . |
| Unemployed and looking for a job | 4.74 | 0.60 | 3.54 | 5.95 | . |
| None of these/other | 5.51 | 1.06 | 3.33 | 7.69 | . |
| Education |  |  |  |  |  |
| Up to 8 years | 6.90 | 0.22 | 6.46 | 7.34 | 0.00 |
| 9-15 years | 6.54 | 0.07 | 6.40 | 6.69 | . |
| 16+ years | 6.14 | 0.12 | 5.89 | 6.38 | . |
| Religious service attendance |  |  |  |  |  |
| >1x/week | 8.89 | 0.10 | 8.69 | 9.10 | 0.00 |
| 1x/week | 7.39 | 0.13 | 7.13 | 7.64 | . |
| 1-3x/month | 7.27 | 0.12 | 7.03 | 7.51 | . |
| A few times a year | 6.33 | 0.16 | 6.02 | 6.64 | . |
| Never | 5.62 | 0.10 | 5.43 | 5.82 | . |
| Immigration status |  |  |  |  |  |
| Born in this country | 6.57 | 0.06 | 6.44 | 6.69 | 0.12 |
| Born in another country | 6.14 | 0.26 | 5.62 | 6.66 | . |
| Religion |  |  |  |  |  |
| Christianity | 6.85 | 0.12 | 6.62 | 7.08 | 0.00 |
| Islam | 7.41 | 0.57 | 6.26 | 8.56 | . |
| Hinduism | 8.16 | 0.39 | 7.31 | 9.00 | . |
| Buddhism | 7.43 | 0.16 | 7.11 | 7.74 | . |
| Judaism | 8.14 | 0.30 | 7.47 | 8.80 | . |
| Sikhism | 8.26 | 1.20 | -189.39 | 205.92 | . |
| Baha'i | 8.17 | 0.88 | 5.29 | 11.04 | . |
| Jainism | 10.00 | . | . | . | . |
| Shinto | 9.14 | 0.27 | 8.50 | 9.78 | . |
| Taoism | 7.34 | 0.27 | 6.81 | 7.87 | . |
| Confucianism | 7.75 | 0.27 | 7.15 | 8.34 | . |
| Primal, Animist, or Folk Religion | 5.62 | 0.49 | 4.61 | 6.62 | . |
| Spiritism | . | . | . | . | . |
| African-derived | . | . | . | . | . |
| Chinese | 7.11 | 0.32 | 6.48 | 7.74 | . |
| Some other religion | 9.63 | 0.51 | 5.25 | 14.01 | . |
| No religion/atheist/agnostic | 5.94 | 0.09 | 5.76 | 6.11 | . |
| Race/ethnicity |  |  |  |  |  |
| Chinese (Cantonese) | 6.45 | 0.07 | 6.30 | 6.59 | 0.00 |
| Chinese (Chaoshan) | 6.37 | 0.27 | 5.83 | 6.91 | . |
| Chinese (Fujianese) | 6.72 | 0.31 | 6.09 | 7.35 | . |
| Chinese (Hakka) | 6.12 | 0.27 | 5.58 | 6.65 | . |
| Chinese (Shanghainese) | 6.91 | 0.47 | 5.94 | 7.87 | . |
| Chinese (other ethnicity) | 6.65 | 0.22 | 6.22 | 7.08 | . |
| East Asian (Korean, Japanese) | 6.60 | 0.55 | 5.01 | 8.19 | . |
| Southeast Asian (Filipino, Indonesian, Thailand) | 7.98 | 0.80 | 6.31 | 9.65 | . |
| South Asian (Indian, Nepalese, Pakistani) | 7.80 | 0.90 | 5.63 | 9.97 | . |
| Taiwanese | 7.67 | 0.57 | 6.27 | 9.07 | . |
| White | 8.07 | 0.88 | 6.10 | 10.04 | . |
| Other | 7.43 | 0.37 | 5.57 | 9.29 | . |

*Note*. *N* = 3,012. SE, standard error; LCI, lower confidence interval; UCI, upper confidence interval.

*Table 6c. Variations Across Childhood Predictors (Hong Kong)*

| Characteristic | Coef. | SE | Prob. | LCI | UCI | Global *p*-value |
| --- | --- | --- | --- | --- | --- | --- |
| Relationship with mother (Ref: Very/somewhat bad) |  |  |  |  |  |  |
| Very/somewhat good | 0.00 | 0.17 | 1.00 | -0.33 | 0.33 | 1.00 |
| Relationship with father (Ref: Very/somewhat bad) |  |  |  |  |  |  |
| Very/somewhat good | 0.18 | 0.16 | 0.27 | -0.14 | 0.49 | 0.27 |
| Parent marital status (Ref: Married) |  |  |  |  |  |  |
| Divorced | -0.07 | 0.34 | 0.83 | -0.75 | 0.60 | 0.58 |
| Never married | 0.66 | 0.51 | 0.20 | -0.35 | 1.66 | . |
| One or both parents had died | -0.24 | 0.68 | 0.73 | -1.58 | 1.11 | . |
| Subjective financial status growing up (Ref: Got by) |  |  |  |  |  |  |
| Lived comfortably | 1.00 | 0.13 | 0.00 | 0.74 | 1.26 | 0.00 |
| Found it difficult | -0.25 | 0.18 | 0.17 | -0.60 | 0.11 | . |
| Found it very difficult | -1.03 | 0.47 | 0.03 | -1.96 | -0.10 | . |
| Childhood abuse (Ref: No) |  |  |  |  |  |  |
| Yes | 0.23 | 0.17 | 0.18 | -0.11 | 0.58 | 0.18 |
| Outsider growing up (Ref: No) |  |  |  |  |  |  |
| Yes | -0.19 | 0.14 | 0.16 | -0.47 | 0.08 | 0.16 |
| Childhood health (Ref: Good) |  |  |  |  |  |  |
| Excellent | 1.34 | 0.20 | 0.00 | 0.94 | 1.74 | 0.00 |
| Very good | 0.64 | 0.14 | 0.00 | 0.37 | 0.90 | . |
| Fair | -0.83 | 0.19 | 0.00 | -1.20 | -0.45 | . |
| Poor | -1.48 | 0.62 | 0.02 | -2.68 | -0.27 | . |
| Immigration status (Ref: Born in this country) |  |  |  |  |  |  |
| Born in another country | 0.01 | 0.22 | 0.97 | -0.42 | 0.44 | 0.97 |
| Childhood religious service attendance (Ref: Never) |  |  |  |  |  |  |
| At least 1x/week | 1.42 | 0.21 | 0.00 | 1.01 | 1.82 | 0.00 |
| 1-3x/month | 1.24 | 0.17 | 0.00 | 0.91 | 1.57 | . |
| <1x/month | 0.51 | 0.16 | 0.00 | 0.21 | 0.82 | . |
| Gender (Ref: Male) |  |  |  |  |  |  |
| Female | 0.20 | 0.10 | 0.05 | 0.00 | 0.40 | 0.09 |
| Other | -0.58 | 0.71 | 0.42 | -1.97 | 0.82 | . |
| Birth year/current age (Ref: 1998-2005; age 18-24) |  |  |  |  |  |  |
| 1993-1998; age 25-29 | 0.14 | 0.21 | 0.50 | -0.27 | 0.55 | 0.00 |
| 1983-1993; age 30-39 | -0.08 | 0.16 | 0.63 | -0.40 | 0.24 | . |
| 1973-1983; age 40-49 | 0.03 | 0.15 | 0.84 | -0.27 | 0.33 | . |
| 1963-1973; age 50-59 | 0.31 | 0.15 | 0.04 | 0.01 | 0.60 | . |
| 1953-1963; age 60-69 | 0.55 | 0.21 | 0.01 | 0.14 | 0.95 | . |
| 1943-1953; age 70-79 | 0.91 | 0.38 | 0.02 | 0.17 | 1.65 | . |
| 1943 or earlier; age 80 or older | 1.51 | 0.33 | 0.00 | 0.85 | 2.16 | . |
| Mother absence/presence (Ref: Present) |  |  |  |  |  |  |
| Absent | 0.19 | 0.21 | 0.37 | -0.23 | 0.61 | 0.37 |
| Father absence/presence (Ref: Present) |  |  |  |  |  |  |
| Absent | 0.15 | 0.21 | 0.48 | -0.27 | 0.57 | 0.48 |
| Childhood religion (Ref: No religion/atheist/agnostic) |  |  |  |  |  |  |
| Christianity | -0.10 | 0.18 | 0.58 | -0.46 | 0.26 | 0.01 |
| Buddhism | 0.16 | 0.18 | 0.36 | -0.19 | 0.52 | . |
| Chinese | 0.31 | 0.31 | 0.32 | -0.30 | 0.92 | . |
| Some other religion | 0.56 | 0.21 | 0.01 | 0.15 | 0.98 | . |
| Race/ethnicity (Ref: Ethnic plurality) |  |  |  |  |  |  |
| Ethnic minority | -0.03 | 0.13 | 0.81 | -0.28 | 0.22 | 0.81 |
| *Note*. *N* = 3,012. SE, standard error; LCI, lower confidence interval; UCI, upper confidence interval. | | | | | | |

*Table 6d. E-Values and E-Value Limits for the Coefficients Shown in Table 6c (Hong Kong)*

| Characteristic | *E*-value for estimate^a^ | *E*-value for 95% CI^b^ |
| --- | --- | --- |
| Relationship with mother (Ref: Very/somewhat bad) |  |  |
| Very/somewhat good | 1.02 | 1.00 |
| Relationship with father (Ref: Very/somewhat bad) |  |  |
| Very/somewhat good | 1.33 | 1.00 |
| Parent marital status (Ref: Married) |  |  |
| Divorced | 1.19 | 1.00 |
| Never married | 1.84 | 1.00 |
| One or both parents had died | 1.40 | 1.00 |
| Subjective financial status growing up (Ref: Got by) |  |  |
| Lived comfortably | 2.21 | 1.93 |
| Found it difficult | 1.41 | 1.00 |
| Found it very difficult | 2.25 | 1.24 |
| Childhood abuse (Ref: No) |  |  |
| Yes | 1.40 | 1.00 |
| Outsider growing up (Ref: No) |  |  |
| Yes | 1.35 | 1.00 |
| Childhood health (Ref: Good) |  |  |
| Excellent | 2.60 | 2.14 |
| Very good | 1.82 | 1.54 |
| Fair | 2.02 | 1.63 |
| Poor | 2.78 | 1.44 |
| Immigration status (Ref: Born in this country) |  |  |
| Born in another country | 1.06 | 1.00 |
| Childhood religious service attendance (Ref: Never) |  |  |
| At least 1x/week | 2.70 | 2.23 |
| 1-3x/month | 2.49 | 2.11 |
| <1x/month | 1.69 | 1.36 |
| Gender (Ref: Male) |  |  |
| Female | 1.36 | 1.02 |
| Other | 1.76 | 1.00 |
| Birth year/current age (Ref: 1998-2005; age 18-24) |  |  |
| 1993-1998; age 25-29 | 1.28 | 1.00 |
| 1983-1993; age 30-39 | 1.20 | 1.00 |
| 1973-1983; age 40-49 | 1.12 | 1.00 |
| 1963-1973; age 50-59 | 1.47 | 1.07 |
| 1953-1963; age 60-69 | 1.73 | 1.29 |
| 1943-1953; age 70-79 | 2.11 | 1.32 |
| 1943 or earlier; age 80 or older | 2.81 | 2.05 |
| Mother absence/presence (Ref: Present) |  |  |
| Absent | 1.35 | 1.00 |
| Father absence/presence (Ref: Present) |  |  |
| Absent | 1.30 | 1.00 |
| Childhood religion (Ref: No religion/atheist/agnostic) |  |  |
| Christianity | 1.23 | 1.00 |
| Buddhism | 1.31 | 1.00 |
| Chinese | 1.48 | 1.00 |
| Some other religion | 1.75 | 1.30 |
| Race/ethnicity (Ref: Ethnic plurality) |  |  |
| Ethnic minority | 1.12 | 1.00 |

*Note*. CI, confidence interval. ^a^The formula for calculating *E*-values can be found in VanderWeele and Ding (2017) The *E*-value for the effect estimate is the minimum strength of association (on the risk ratio scale) that an unmeasured confounder would need to have with both the predictor and the outcome to entirely explain away the observed association between them, conditional on the measured covariates. ^b^The *E*-value for the limit of the 95% confidence interval closest to the null denote the minimum strength of association (on the risk ratio scale) that an unmeasured confounder would need to have with both the predictor and the outcome to shift the confidence interval to include the null value, conditional on the measured covariates.

*Table S7a. Nationally Representative Descriptive Statistics of the Observed Sample (India)*

| Variable | Proportion | Frequency |
| --- | --- | --- |
| Sociodemographic characteristics |  |  |
| Birth year/current age |  |  |
| 1998-2005; age 18-24 | 0.20 | 2543 |
| 1993-1998; age 25-29 | 0.13 | 1640 |
| 1983-1993; age 30-39 | 0.24 | 3109 |
| 1973-1983; age 40-49 | 0.18 | 2275 |
| 1963-1973; age 50-59 | 0.12 | 1574 |
| 1953-1963; age 60-69 | 0.09 | 1188 |
| 1943-1953; age 70-79 | 0.03 | 370 |
| 1943 or earlier; 80 or older | 0.01 | 67 |
| Missing | . | . |
| Gender |  |  |
| Male | 0.51 | 6473 |
| Female | 0.49 | 6292 |
| Other | . | . |
| Missing | . | . |
| Marital status |  |  |
| Single, never married | 0.16 | 2065 |
| Married | 0.77 | 9848 |
| Separated | 0.00 | 45 |
| Divorced | 0.00 | 25 |
| Widowed | 0.03 | 445 |
| Domestic partner | 0.02 | 269 |
| Missing | 0.01 | 69 |
| Employment |  |  |
| Employed for an employer | 0.21 | 2660 |
| Self-employed | 0.27 | 3401 |
| Retired | 0.02 | 286 |
| Student | 0.04 | 532 |
| Homemaker | 0.33 | 4221 |
| Unemployed and looking for a job | 0.07 | 902 |
| None of these/other | 0.06 | 715 |
| Missing | 0.00 | 48 |
| Education |  |  |
| Up to 8 years | 0.89 | 11422 |
| 9-15 years | 0.09 | 1194 |
| 16+ years | 0.01 | 145 |
| Missing | 0.00 | 4 |
| Religious service attendance |  |  |
| >1x/week | 0.23 | 2875 |
| 1x/week | 0.25 | 3166 |
| 1-3x/month | 0.21 | 2740 |
| A few times a year | 0.16 | 2090 |
| Never | 0.14 | 1823 |
| Missing | 0.01 | 71 |
| Immigration status |  |  |
| Born in this country | 0.99 | 12629 |
| Born in another country | 0.01 | 110 |
| Missing | 0.00 | 26 |
| Religion |  |  |
| Christianity | 0.02 | 306 |
| Islam | 0.12 | 1555 |
| Hinduism | 0.81 | 10362 |
| Buddhism | 0.02 | 230 |
| Judaism | . | . |
| Sikhism | 0.01 | 127 |
| Baha'i | . | . |
| Jainism | 0.00 | 10 |
| Shinto | 0.00 | 1 |
| Taoism | . | . |
| Confucianism | . | . |
| Primal, Animist, or Folk Religion | 0.00 | 30 |
| Spiritism | . | . |
| African-derived | . | . |
| Chinese | . | . |
| Some other religion | 0.01 | 67 |
| No religion/atheist/agnostic | 0.00 | 13 |
| Missing | 0.00 | 62 |
| Race/ethnicity |  |  |
| General | 0.28 | 3538 |
| Other Backward Caste | 0.33 | 4177 |
| Schedule Caste | 0.28 | 3599 |
| Schedule Tribe | 0.09 | 1185 |
| Other | . | . |
| Missing | 0.02 | 267 |
| Childhood factors |  |  |
| Relationship with mother |  |  |
| Very good | 0.90 | 11465 |
| Somewhat good | 0.06 | 788 |
| Somewhat bad | 0.01 | 88 |
| Very bad | 0.01 | 73 |
| Not applicable | 0.02 | 269 |
| Missing | 0.01 | 82 |
| Relationship with father |  |  |
| Very good | 0.86 | 10923 |
| Somewhat good | 0.08 | 995 |
| Somewhat bad | 0.01 | 126 |
| Very bad | 0.01 | 100 |
| Not applicable | 0.04 | 481 |
| Missing | 0.01 | 141 |
| Parent marital status |  |  |
| Married | 0.44 | 5578 |
| Divorced | 0.02 | 236 |
| Never married | 0.08 | 1055 |
| One or both parents had died | 0.07 | 940 |
| Missing | 0.39 | 4956 |
| Subjective financial status growing up |  |  |
| Lived comfortably | 0.39 | 4946 |
| Got by | 0.24 | 3010 |
| Found it difficult | 0.21 | 2703 |
| Found it very difficult | 0.16 | 2035 |
| Missing | 0.01 | 70 |
| Childhood abuse |  |  |
| Yes | 0.11 | 1468 |
| No | 0.82 | 10526 |
| Missing | 0.06 | 771 |
| Outsider growing up |  |  |
| Yes | 0.15 | 1926 |
| No | 0.84 | 10780 |
| Not applicable | 0.00 | 15 |
| Missing | 0.00 | 44 |
| Childhood health |  |  |
| Excellent | 0.17 | 2182 |
| Very good | 0.30 | 3882 |
| Good | 0.32 | 4028 |
| Fair | 0.17 | 2202 |
| Poor | 0.03 | 424 |
| Missing | 0.00 | 47 |
| Childhood religious service attendance |  |  |
| At least 1x/week | 0.41 | 5288 |
| 1-3x/month | 0.23 | 2959 |
| <1x/month | 0.21 | 2719 |
| Never | 0.12 | 1478 |
| Missing | 0.03 | 321 |
| Childhood religion |  |  |
| Christianity | 0.02 | 254 |
| Islam | 0.12 | 1550 |
| Hinduism | 0.82 | 10417 |
| Buddhism | 0.01 | 180 |
| Judaism | . | . |
| Sikhism | 0.01 | 126 |
| Baha'i | . | . |
| Jainism | 0.00 | 9 |
| Shinto | 0.00 | 4 |
| Taoism | . | . |
| Confucianism | . | . |
| Primal, Animist, or Folk Religion | 0.00 | 27 |
| Spiritism | . | . |
| African-derived | . | . |
| Chinese | . | . |
| Some other religion | 0.00 | 59 |
| No religion/atheist/agnostic | 0.00 | 7 |
| Missing | 0.01 | 131 |

*Note*. *N* = 12,765.

*Table S7b. Variations Across Sociodemographic Characteristics (India)*

| Characteristic | Mean | SE | LCI | UCI | Global *p*-value |
| --- | --- | --- | --- | --- | --- |
| Age group |  |  |  |  |  |
| 18-24 | 8.38 | 0.08 | 8.22 | 8.54 | 0.02 |
| 25-29 | 8.36 | 0.08 | 8.19 | 8.53 | . |
| 30-39 | 8.22 | 0.06 | 8.10 | 8.35 | . |
| 40-49 | 8.13 | 0.08 | 7.98 | 8.28 | . |
| 50-59 | 8.03 | 0.10 | 7.84 | 8.22 | . |
| 60-69 | 7.98 | 0.11 | 7.76 | 8.20 | . |
| 70-79 | 8.07 | 0.17 | 7.74 | 8.41 | . |
| 80 or older | 8.16 | 0.43 | 7.27 | 9.05 | . |
| Gender |  |  |  |  |  |
| Male | 8.11 | 0.05 | 8.01 | 8.21 | 0.00 |
| Female | 8.30 | 0.05 | 8.21 | 8.39 | . |
| Other | . | . | . | . | . |
| Marital status |  |  |  |  |  |
| Single, never married | 8.08 | 0.09 | 7.90 | 8.26 | 0.00 |
| Married | 8.28 | 0.04 | 8.20 | 8.36 | . |
| Separated | 6.64 | 0.69 | 5.16 | 8.11 | . |
| Divorced | 6.47 | 0.79 | 4.49 | 8.44 | . |
| Widowed | 7.80 | 0.19 | 7.42 | 8.17 | . |
| Domestic partner | 7.50 | 0.26 | 6.98 | 8.03 | . |
| Employment |  |  |  |  |  |
| Employed for an employer | 8.09 | 0.08 | 7.94 | 8.25 | 0.28 |
| Self-employed | 8.23 | 0.07 | 8.09 | 8.36 | . |
| Retired | 8.34 | 0.21 | 7.93 | 8.75 | . |
| Student | 8.16 | 0.16 | 7.84 | 8.48 | . |
| Homemaker | 8.29 | 0.06 | 8.18 | 8.40 | . |
| Unemployed and looking for a job | 8.01 | 0.12 | 7.77 | 8.24 | . |
| None of these/other | 8.21 | 0.14 | 7.94 | 8.48 | . |
| Education |  |  |  |  |  |
| Up to 8 years | 8.19 | 0.04 | 8.12 | 8.27 | 0.51 |
| 9-15 years | 8.29 | 0.09 | 8.12 | 8.46 | . |
| 16+ years | 8.29 | 0.18 | 7.94 | 8.64 | . |
| Religious service attendance |  |  |  |  |  |
| >1x/week | 8.22 | 0.07 | 8.08 | 8.36 | 0.00 |
| 1x/week | 8.38 | 0.06 | 8.25 | 8.50 | . |
| 1-3x/month | 8.24 | 0.07 | 8.10 | 8.38 | . |
| A few times a year | 8.07 | 0.07 | 7.92 | 8.22 | . |
| Never | 7.97 | 0.10 | 7.78 | 8.16 | . |
| Immigration status |  |  |  |  |  |
| Born in this country | 8.21 | 0.04 | 8.14 | 8.28 | 0.37 |
| Born in another country | 7.97 | 0.25 | 7.46 | 8.48 | . |
| Religion |  |  |  |  |  |
| Christianity | 8.69 | 0.13 | 8.42 | 8.96 | 0.00 |
| Islam | 8.30 | 0.10 | 8.11 | 8.50 | . |
| Hinduism | 8.18 | 0.04 | 8.10 | 8.26 | . |
| Buddhism | 8.09 | 0.20 | 7.68 | 8.50 | . |
| Judaism | . | . | . | . | . |
| Sikhism | 7.85 | 0.25 | 7.29 | 8.42 | . |
| Baha'i | . | . | . | . | . |
| Jainism | 6.42 | 0.63 | 1.00 | 11.83 | . |
| Shinto | 10.00 | . | . | . | . |
| Taoism | . | . | . | . | . |
| Confucianism | . | . | . | . | . |
| Primal, Animist, or Folk Religion | 8.43 | 0.54 | 7.14 | 9.72 | . |
| Spiritism | . | . | . | . | . |
| African-derived | . | . | . | . | . |
| Chinese | . | . | . | . | . |
| Some other religion | 8.69 | 0.18 | 8.22 | 9.15 | . |
| No religion/atheist/agnostic | 8.00 | . | . | . | . |
| Race/ethnicity |  |  |  |  |  |
| General | 8.14 | 0.07 | 8.00 | 8.28 | 0.71 |
| Other Backward Caste | 8.24 | 0.06 | 8.12 | 8.35 | . |
| Schedule Caste | 8.24 | 0.06 | 8.12 | 8.35 | . |
| Schedule Tribe | 8.18 | 0.10 | 7.97 | 8.38 | . |
| Other | . | . | . | . | . |

*Note*. *N* = 12,765. SE, standard error; LCI, lower confidence interval; UCI, upper confidence interval.

*Table 7c. Variations Across Childhood Predictors (India)*

| Characteristic | Coef. | SE | Prob. | LCI | UCI | Global *p*-value |
| --- | --- | --- | --- | --- | --- | --- |
| Relationship with mother (Ref: Very/somewhat bad) |  |  |  |  |  |  |
| Very/somewhat good | 0.01 | 0.27 | 0.97 | -0.51 | 0.53 | 0.97 |
| Relationship with father (Ref: Very/somewhat bad) |  |  |  |  |  |  |
| Very/somewhat good | -0.13 | 0.20 | 0.53 | -0.52 | 0.27 | 0.53 |
| Parent marital status (Ref: Married) |  |  |  |  |  |  |
| Divorced | -0.12 | 0.23 | 0.59 | -0.58 | 0.33 | 0.00 |
| Never married | -0.17 | 0.13 | 0.21 | -0.44 | 0.10 | . |
| One or both parents had died | 0.48 | 0.13 | 0.00 | 0.22 | 0.74 | . |
| Subjective financial status growing up (Ref: Got by) |  |  |  |  |  |  |
| Lived comfortably | -0.05 | 0.09 | 0.56 | -0.22 | 0.12 | 0.27 |
| Found it difficult | -0.15 | 0.11 | 0.16 | -0.36 | 0.06 | . |
| Found it very difficult | -0.20 | 0.11 | 0.07 | -0.43 | 0.02 | . |
| Childhood abuse (Ref: No) |  |  |  |  |  |  |
| Yes | -0.07 | 0.11 | 0.52 | -0.29 | 0.15 | 0.52 |
| Outsider growing up (Ref: No) |  |  |  |  |  |  |
| Yes | 0.00 | 0.10 | 1.00 | -0.19 | 0.19 | 1.00 |
| Childhood health (Ref: Good) |  |  |  |  |  |  |
| Excellent | -0.07 | 0.11 | 0.52 | -0.28 | 0.14 | 0.40 |
| Very good | -0.03 | 0.08 | 0.75 | -0.18 | 0.13 | . |
| Fair | -0.19 | 0.10 | 0.06 | -0.40 | 0.01 | . |
| Poor | -0.01 | 0.18 | 0.96 | -0.36 | 0.34 | . |
| Immigration status (Ref: Born in this country) |  |  |  |  |  |  |
| Born in another country | -0.13 | 0.27 | 0.64 | -0.66 | 0.41 | 0.64 |
| Childhood religious service attendance (Ref: Never) |  |  |  |  |  |  |
| At least 1x/week | 0.08 | 0.11 | 0.48 | -0.14 | 0.29 | 0.78 |
| 1-3x/month | 0.04 | 0.11 | 0.70 | -0.18 | 0.26 | . |
| <1x/month | 0.11 | 0.11 | 0.35 | -0.12 | 0.33 | . |
| Gender (Ref: Male) |  |  |  |  |  |  |
| Female | 0.18 | 0.07 | 0.01 | 0.05 | 0.31 | 0.01 |
| Other | . | . | . | . | . | . |
| Birth year/current age (Ref: 1998-2005; age 18-24) |  |  |  |  |  |  |
| 1993-1998; age 25-29 | -0.02 | 0.12 | 0.88 | -0.26 | 0.22 | 0.00 |
| 1983-1993; age 30-39 | -0.17 | 0.10 | 0.11 | -0.37 | 0.04 | . |
| 1973-1983; age 40-49 | -0.27 | 0.12 | 0.02 | -0.50 | -0.04 | . |
| 1963-1973; age 50-59 | -0.38 | 0.13 | 0.00 | -0.63 | -0.13 | . |
| 1953-1963; age 60-69 | -0.44 | 0.14 | 0.00 | -0.72 | -0.16 | . |
| 1943-1953; age 70-79 | -0.35 | 0.19 | 0.06 | -0.72 | 0.01 | . |
| 1943 or earlier; age 80 or older | -0.32 | 0.50 | 0.52 | -1.30 | 0.66 | . |
| Mother absence/presence (Ref: Present) |  |  |  |  |  |  |
| Absent | 0.09 | 0.26 | 0.73 | -0.42 | 0.59 | 0.73 |
| Father absence/presence (Ref: Present) |  |  |  |  |  |  |
| Absent | -0.14 | 0.23 | 0.55 | -0.59 | 0.32 | 0.55 |
| Childhood religion (Ref: Hinduism) |  |  |  |  |  |  |
| Islam | 0.10 | 0.11 | 0.35 | -0.12 | 0.33 | 0.03 |
| Some other religion | 0.29 | 0.12 | 0.01 | 0.06 | 0.53 | . |
| Race/ethnicity (Ref: Ethnic plurality) |  |  |  |  |  |  |
| Ethnic minority | -0.04 | 0.07 | 0.58 | -0.18 | 0.10 | 0.58 |
| *Note*. *N* = 12,765. SE, standard error; LCI, lower confidence interval; UCI, upper confidence interval. | | | | | | |

*Table 7d. E-Values and E-Value Limits for the Coefficients Shown in Table 7c (India)*

| Characteristic | *E*-value for estimate^a^ | *E*-value for 95% CI^b^ |
| --- | --- | --- |
| Relationship with mother (Ref: Very/somewhat bad) |  |  |
| Very/somewhat good | 1.06 | 1.00 |
| Relationship with father (Ref: Very/somewhat bad) |  |  |
| Very/somewhat good | 1.25 | 1.00 |
| Parent marital status (Ref: Married) |  |  |
| Divorced | 1.24 | 1.00 |
| Never married | 1.30 | 1.00 |
| One or both parents had died | 1.60 | 1.36 |
| Subjective financial status growing up (Ref: Got by) |  |  |
| Lived comfortably | 1.14 | 1.00 |
| Found it difficult | 1.27 | 1.00 |
| Found it very difficult | 1.34 | 1.00 |
| Childhood abuse (Ref: No) |  |  |
| Yes | 1.18 | 1.00 |
| Outsider growing up (Ref: No) |  |  |
| Yes | 1.01 | 1.00 |
| Childhood health (Ref: Good) |  |  |
| Excellent | 1.17 | 1.00 |
| Very good | 1.10 | 1.00 |
| Fair | 1.32 | 1.00 |
| Poor | 1.06 | 1.00 |
| Immigration status (Ref: Born in this country) |  |  |
| Born in another country | 1.25 | 1.00 |
| Childhood religious service attendance (Ref: Never) |  |  |
| At least 1x/week | 1.18 | 1.00 |
| 1-3x/month | 1.13 | 1.00 |
| <1x/month | 1.22 | 1.00 |
| Gender (Ref: Male) |  |  |
| Female | 1.31 | 1.15 |
| Other | . | . |
| Birth year/current age (Ref: 1998-2005; age 18-24) |  |  |
| 1993-1998; age 25-29 | 1.08 | 1.00 |
| 1983-1993; age 30-39 | 1.29 | 1.00 |
| 1973-1983; age 40-49 | 1.40 | 1.12 |
| 1963-1973; age 50-59 | 1.51 | 1.25 |
| 1953-1963; age 60-69 | 1.57 | 1.29 |
| 1943-1953; age 70-79 | 1.48 | 1.00 |
| 1943 or earlier; age 80 or older | 1.45 | 1.00 |
| Mother absence/presence (Ref: Present) |  |  |
| Absent | 1.20 | 1.00 |
| Father absence/presence (Ref: Present) |  |  |
| Absent | 1.26 | 1.00 |
| Childhood religion (Ref: Hinduism) |  |  |
| Islam | 1.22 | 1.00 |
| Some other religion | 1.43 | 1.16 |
| Race/ethnicity (Ref: Ethnic plurality) |  |  |
| Ethnic minority | 1.13 | 1.00 |

*Note*. CI, confidence interval. ^a^The formula for calculating *E*-values can be found in VanderWeele and Ding (2017) The *E*-value for the effect estimate is the minimum strength of association (on the risk ratio scale) that an unmeasured confounder would need to have with both the predictor and the outcome to entirely explain away the observed association between them, conditional on the measured covariates. ^b^The *E*-value for the limit of the 95% confidence interval closest to the null denote the minimum strength of association (on the risk ratio scale) that an unmeasured confounder would need to have with both the predictor and the outcome to shift the confidence interval to include the null value, conditional on the measured covariates.

*Table S8a. Nationally Representative Descriptive Statistics of the Observed Sample (Indonesia)*

| Variable | Proportion | Frequency |
| --- | --- | --- |
| Sociodemographic characteristics |  |  |
| Birth year/current age |  |  |
| 1998-2005; age 18-24 | 0.17 | 1216 |
| 1993-1998; age 25-29 | 0.12 | 849 |
| 1983-1993; age 30-39 | 0.23 | 1591 |
| 1973-1983; age 40-49 | 0.23 | 1576 |
| 1963-1973; age 50-59 | 0.17 | 1169 |
| 1953-1963; age 60-69 | 0.07 | 490 |
| 1943-1953; age 70-79 | 0.01 | 83 |
| 1943 or earlier; 80 or older | 0.00 | 17 |
| Missing | . | . |
| Gender |  |  |
| Male | 0.50 | 3461 |
| Female | 0.50 | 3513 |
| Other | 0.00 | 7 |
| Missing | 0.00 | 11 |
| Marital status |  |  |
| Single, never married | 0.20 | 1381 |
| Married | 0.69 | 4846 |
| Separated | 0.01 | 82 |
| Divorced | 0.03 | 196 |
| Widowed | 0.06 | 425 |
| Domestic partner | 0.00 | 18 |
| Missing | 0.01 | 45 |
| Employment |  |  |
| Employed for an employer | 0.19 | 1323 |
| Self-employed | 0.31 | 2187 |
| Retired | 0.01 | 78 |
| Student | 0.04 | 272 |
| Homemaker | 0.31 | 2138 |
| Unemployed and looking for a job | 0.08 | 529 |
| None of these/other | 0.06 | 448 |
| Missing | 0.00 | 18 |
| Education |  |  |
| Up to 8 years | 0.44 | 3079 |
| 9-15 years | 0.50 | 3491 |
| 16+ years | 0.06 | 419 |
| Missing | 0.00 | 2 |
| Religious service attendance |  |  |
| >1x/week | 0.38 | 2667 |
| 1x/week | 0.36 | 2529 |
| 1-3x/month | 0.11 | 786 |
| A few times a year | 0.09 | 659 |
| Never | 0.05 | 332 |
| Missing | 0.00 | 18 |
| Immigration status |  |  |
| Born in this country | 1.00 | 6958 |
| Born in another country | 0.00 | 34 |
| Missing | . | . |
| Religion |  |  |
| Christianity | 0.07 | 504 |
| Islam | 0.92 | 6406 |
| Hinduism | 0.01 | 73 |
| Buddhism | 0.00 | 3 |
| Judaism | . | . |
| Sikhism | . | . |
| Baha'i | . | . |
| Jainism | . | . |
| Shinto | . | . |
| Taoism | 0.00 | 1 |
| Confucianism | . | . |
| Primal, Animist, or Folk Religion | . | . |
| Spiritism | . | . |
| African-derived | . | . |
| Chinese | . | . |
| Some other religion | 0.00 | 1 |
| No religion/atheist/agnostic | . | . |
| Missing | 0.00 | 4 |
| Race/ethnicity |  |  |
| Banjar/Melayu Banjar | 0.05 | 320 |
| Betawi | 0.04 | 251 |
| Bugis | 0.03 | 243 |
| Jawa | 0.41 | 2846 |
| Madura | 0.04 | 262 |
| Minangkabau | 0.04 | 273 |
| Sunda/Parahyangan | 0.17 | 1172 |
| Bali | 0.01 | 69 |
| Batak | 0.02 | 165 |
| Makasar | 0.01 | 91 |
| Other | 0.18 | 1262 |
| Missing | 0.01 | 38 |
| Childhood factors |  |  |
| Relationship with mother |  |  |
| Very good | 0.89 | 6238 |
| Somewhat good | 0.08 | 583 |
| Somewhat bad | 0.01 | 50 |
| Very bad | 0.00 | 26 |
| Not applicable | 0.01 | 68 |
| Missing | 0.00 | 27 |
| Relationship with father |  |  |
| Very good | 0.87 | 6067 |
| Somewhat good | 0.09 | 628 |
| Somewhat bad | 0.01 | 68 |
| Very bad | 0.01 | 52 |
| Not applicable | 0.02 | 115 |
| Missing | 0.01 | 61 |
| Parent marital status |  |  |
| Married | 0.79 | 5557 |
| Divorced | 0.06 | 448 |
| Never married | 0.01 | 47 |
| One or both parents had died | 0.11 | 735 |
| Missing | 0.03 | 205 |
| Subjective financial status growing up |  |  |
| Lived comfortably | 0.49 | 3408 |
| Got by | 0.42 | 2955 |
| Found it difficult | 0.06 | 439 |
| Found it very difficult | 0.03 | 181 |
| Missing | 0.00 | 9 |
| Childhood abuse |  |  |
| Yes | 0.07 | 486 |
| No | 0.92 | 6427 |
| Missing | 0.01 | 79 |
| Outsider growing up |  |  |
| Yes | 0.05 | 343 |
| No | 0.95 | 6639 |
| Not applicable | 0.00 | 1 |
| Missing | 0.00 | 9 |
| Childhood health |  |  |
| Excellent | 0.18 | 1246 |
| Very good | 0.28 | 1968 |
| Good | 0.36 | 2490 |
| Fair | 0.18 | 1233 |
| Poor | 0.01 | 55 |
| Missing | 0.00 | 1 |
| Childhood religious service attendance |  |  |
| At least 1x/week | 0.77 | 5363 |
| 1-3x/month | 0.14 | 973 |
| <1x/month | 0.05 | 329 |
| Never | 0.04 | 275 |
| Missing | 0.01 | 51 |
| Childhood religion |  |  |
| Christianity | 0.08 | 528 |
| Islam | 0.91 | 6373 |
| Hinduism | 0.01 | 75 |
| Buddhism | 0.00 | 5 |
| Judaism | . | . |
| Sikhism | . | . |
| Baha'i | . | . |
| Jainism | 0.00 | 1 |
| Shinto | . | . |
| Taoism | 0.00 | 0 |
| Confucianism | 0.00 | 1 |
| Primal, Animist, or Folk Religion | 0.00 | 1 |
| Spiritism | . | . |
| African-derived | . | . |
| Chinese | . | . |
| Some other religion | . | . |
| No religion/atheist/agnostic | 0.00 | 2 |
| Missing | 0.00 | 8 |

*Note. N* = 6,992.

*Table S8b. Variations Across Sociodemographic Characteristics (Indonesia)*

| Characteristic | Mean | SE | LCI | UCI | Global *p*-value |
| --- | --- | --- | --- | --- | --- |
| Age group |  |  |  |  |  |
| 18-24 | 8.68 | 0.07 | 8.55 | 8.82 | 0.00 |
| 25-29 | 9.00 | 0.06 | 8.88 | 9.11 | . |
| 30-39 | 8.96 | 0.05 | 8.87 | 9.05 | . |
| 40-49 | 9.01 | 0.06 | 8.90 | 9.13 | . |
| 50-59 | 8.67 | 0.09 | 8.50 | 8.84 | . |
| 60-69 | 8.69 | 0.14 | 8.41 | 8.96 | . |
| 70-79 | 8.31 | 0.49 | 7.30 | 9.32 | . |
| 80 or older | . | . | . | . | . |
| Gender |  |  |  |  |  |
| Male | 8.79 | 0.05 | 8.69 | 8.88 | 0.00 |
| Female | 8.92 | 0.03 | 8.85 | 8.99 | . |
| Other | 6.70 | 0.90 | . | . | . |
| Marital status |  |  |  |  |  |
| Single, never married | 8.70 | 0.07 | 8.57 | 8.83 | 0.00 |
| Married | 8.90 | 0.04 | 8.83 | 8.97 | . |
| Separated | 8.83 | 0.24 | 8.35 | 9.31 | . |
| Divorced | 9.05 | 0.15 | 8.75 | 9.34 | . |
| Widowed | 8.64 | 0.14 | 8.36 | 8.92 | . |
| Domestic partner | 9.78 | 0.09 | 9.52 | 10.03 | . |
| Employment |  |  |  |  |  |
| Employed for an employer | 8.82 | 0.07 | 8.68 | 8.95 | 0.29 |
| Self-employed | 8.85 | 0.05 | 8.75 | 8.95 | . |
| Retired | 8.66 | 0.16 | 8.35 | 8.98 | . |
| Student | 8.69 | 0.12 | 8.45 | 8.93 | . |
| Homemaker | 8.94 | 0.05 | 8.85 | 9.04 | . |
| Unemployed and looking for a job | 8.85 | 0.12 | 8.62 | 9.08 | . |
| None of these/other | 8.68 | 0.15 | 8.38 | 8.97 | . |
| Education |  |  |  |  |  |
| Up to 8 years | 8.87 | 0.05 | 8.77 | 8.98 | 0.41 |
| 9-15 years | 8.84 | 0.03 | 8.78 | 8.91 | . |
| 16+ years | 8.76 | 0.07 | 8.62 | 8.89 | . |
| Religious service attendance |  |  |  |  |  |
| >1x/week | 8.99 | 0.05 | 8.90 | 9.08 | 0.00 |
| 1x/week | 8.83 | 0.05 | 8.74 | 8.93 | . |
| 1-3x/month | 8.73 | 0.08 | 8.57 | 8.89 | . |
| A few times a year | 8.63 | 0.09 | 8.45 | 8.81 | . |
| Never | 8.60 | 0.17 | 8.27 | 8.94 | . |
| Immigration status |  |  |  |  |  |
| Born in this country | 8.85 | 0.03 | 8.79 | 8.91 | 0.80 |
| Born in another country | 8.94 | 0.33 | 8.16 | 9.72 | . |
| Religion |  |  |  |  |  |
| Christianity | 9.01 | 0.11 | 8.79 | 9.23 | 0.00 |
| Islam | 8.84 | 0.03 | 8.78 | 8.90 | . |
| Hinduism | 8.88 | 0.14 | 8.57 | 9.19 | . |
| Buddhism | 8.00 | . | . | . | . |
| Judaism | . | . | . | . | . |
| Sikhism | . | . | . | . | . |
| Baha'i | . | . | . | . | . |
| Jainism | . | . | . | . | . |
| Shinto | . | . | . | . | . |
| Taoism | 10.00 | . | . | . | . |
| Confucianism | . | . | . | . | . |
| Primal, Animist, or Folk Religion | . | . | . | . | . |
| Spiritism | . | . | . | . | . |
| African-derived | . | . | . | . | . |
| Chinese | . | . | . | . | . |
| Some other religion | 8.00 | . | . | . | . |
| No religion/atheist/agnostic | . | . | . | . | . |
| Race/ethnicity |  |  |  |  |  |
| Banjar/Melayu Banjar | 8.65 | 0.14 | 8.36 | 8.94 | 0.03 |
| Betawi | 8.78 | 0.16 | 8.45 | 9.11 | . |
| Bugis | 9.01 | 0.10 | 8.81 | 9.20 | . |
| Jawa | 8.82 | 0.05 | 8.73 | 8.91 | . |
| Madura | 9.00 | 0.18 | 8.63 | 9.36 | . |
| Minangkabau | 8.72 | 0.10 | 8.51 | 8.92 | . |
| Sunda/Parahyangan | 8.86 | 0.08 | 8.70 | 9.02 | . |
| Bali | 8.83 | 0.17 | 8.46 | 9.21 | . |
| Batak | 8.67 | 0.13 | 8.42 | 8.92 | . |
| Makasar | 9.21 | 0.08 | 9.01 | 9.41 | . |
| Other | 8.96 | 0.07 | 8.81 | 9.11 | . |

*Note*. *N* = 6,992. SE, standard error; LCI, lower confidence interval; UCI, upper confidence interval.

*Table 8c. Variations Across Childhood Predictors (Indonesia)*

| Characteristic | Coef. | SE | Prob. | LCI | UCI | Global *p*-value |
| --- | --- | --- | --- | --- | --- | --- |
| Relationship with mother (Ref: Very/somewhat bad) |  |  |  |  |  |  |
| Very/somewhat good | 0.02 | 0.26 | 0.93 | -0.48 | 0.53 | 0.93 |
| Relationship with father (Ref: Very/somewhat bad) |  |  |  |  |  |  |
| Very/somewhat good | 0.27 | 0.21 | 0.21 | -0.15 | 0.69 | 0.21 |
| Parent marital status (Ref: Married) |  |  |  |  |  |  |
| Divorced | 0.05 | 0.11 | 0.68 | -0.18 | 0.27 | 0.19 |
| Never married | 0.17 | 0.37 | 0.64 | -0.55 | 0.89 | . |
| One or both parents had died | 0.22 | 0.10 | 0.03 | 0.02 | 0.42 | . |
| Subjective financial status growing up (Ref: Got by) |  |  |  |  |  |  |
| Lived comfortably | 0.16 | 0.06 | 0.01 | 0.04 | 0.27 | 0.03 |
| Found it difficult | -0.07 | 0.15 | 0.64 | -0.36 | 0.22 | . |
| Found it very difficult | 0.00 | 0.22 | 0.99 | -0.44 | 0.43 | . |
| Childhood abuse (Ref: No) |  |  |  |  |  |  |
| Yes | -0.22 | 0.14 | 0.12 | -0.49 | 0.05 | 0.12 |
| Outsider growing up (Ref: No) |  |  |  |  |  |  |
| Yes | 0.14 | 0.15 | 0.34 | -0.15 | 0.44 | 0.34 |
| Childhood health (Ref: Good) |  |  |  |  |  |  |
| Excellent | 0.32 | 0.08 | 0.00 | 0.17 | 0.48 | 0.00 |
| Very good | 0.16 | 0.07 | 0.03 | 0.02 | 0.29 | . |
| Fair | 0.00 | 0.09 | 0.97 | -0.17 | 0.16 | . |
| Poor | -0.15 | 0.35 | 0.67 | -0.84 | 0.54 | . |
| Immigration status (Ref: Born in this country) |  |  |  |  |  |  |
| Born in another country | 0.04 | 0.37 | 0.91 | -0.68 | 0.76 | 0.91 |
| Childhood religious service attendance (Ref: Never) |  |  |  |  |  |  |
| At least 1x/week | 0.00 | 0.15 | 0.99 | -0.28 | 0.29 | 0.55 |
| 1-3x/month | -0.14 | 0.17 | 0.41 | -0.46 | 0.19 | . |
| <1x/month | -0.06 | 0.18 | 0.74 | -0.43 | 0.30 | . |
| Gender (Ref: Male) |  |  |  |  |  |  |
| Female | 0.13 | 0.06 | 0.02 | 0.02 | 0.24 | 0.00 |
| Other | -2.04 | 0.90 | 0.02 | -3.80 | -0.28 | . |
| Birth year/current age (Ref: 1998-2005; age 18-24) |  |  |  |  |  |  |
| 1993-1998; age 25-29 | 0.33 | 0.09 | 0.00 | 0.15 | 0.52 | 0.00 |
| 1983-1993; age 30-39 | 0.32 | 0.08 | 0.00 | 0.16 | 0.48 | . |
| 1973-1983; age 40-49 | 0.39 | 0.09 | 0.00 | 0.21 | 0.56 | . |
| 1963-1973; age 50-59 | 0.07 | 0.11 | 0.54 | -0.15 | 0.28 | . |
| 1953-1963; age 60-69 | 0.10 | 0.16 | 0.53 | -0.21 | 0.40 | . |
| 1943-1953; age 70-79 | -0.33 | 0.48 | 0.49 | -1.26 | 0.61 | . |
| 1943 or earlier; age 80 or older | 0.22 | 0.62 | 0.72 | -1.00 | 1.45 | . |
| Mother absence/presence (Ref: Present) |  |  |  |  |  |  |
| Absent | -0.15 | 0.29 | 0.60 | -0.73 | 0.42 | 0.60 |
| Father absence/presence (Ref: Present) |  |  |  |  |  |  |
| Absent | -0.18 | 0.22 | 0.40 | -0.61 | 0.24 | 0.40 |
| Childhood religion (Ref: Islam) |  |  |  |  |  |  |
| Christianity | 0.22 | 0.11 | 0.05 | 0.00 | 0.44 | 0.15 |
| Some other religion | 0.04 | 0.20 | 0.83 | -0.34 | 0.43 | . |
| Race/ethnicity (Ref: Ethnic plurality) |  |  |  |  |  |  |
| Ethnic minority | 0.01 | 0.06 | 0.93 | -0.12 | 0.13 | 0.93 |
| *Note*. *N* = 6,992. SE, standard error; LCI, lower confidence interval; UCI, upper confidence interval. | | | | | | |

*Table 8d. E-Values and E-Value Limits for the Coefficients Shown in Table 8c (Indonesia)*

| Characteristic | *E*-value for estimate^a^ | *E*-value for 95% CI^b^ |
| --- | --- | --- |
| Relationship with mother (Ref: Very/somewhat bad) |  |  |
| Very/somewhat good | 1.09 | 1.00 |
| Relationship with father (Ref: Very/somewhat bad) |  |  |
| Very/somewhat good | 1.39 | 1.00 |
| Parent marital status (Ref: Married) |  |  |
| Divorced | 1.14 | 1.00 |
| Never married | 1.29 | 1.00 |
| One or both parents had died | 1.34 | 1.08 |
| Subjective financial status growing up (Ref: Got by) |  |  |
| Lived comfortably | 1.28 | 1.13 |
| Found it difficult | 1.17 | 1.00 |
| Found it very difficult | 1.03 | 1.00 |
| Childhood abuse (Ref: No) |  |  |
| Yes | 1.34 | 1.00 |
| Outsider growing up (Ref: No) |  |  |
| Yes | 1.26 | 1.00 |
| Childhood health (Ref: Good) |  |  |
| Excellent | 1.44 | 1.29 |
| Very good | 1.27 | 1.08 |
| Fair | 1.03 | 1.00 |
| Poor | 1.27 | 1.00 |
| Immigration status (Ref: Born in this country) |  |  |
| Born in another country | 1.13 | 1.00 |
| Childhood religious service attendance (Ref: Never) |  |  |
| At least 1x/week | 1.03 | 1.00 |
| 1-3x/month | 1.25 | 1.00 |
| <1x/month | 1.16 | 1.00 |
| Gender (Ref: Male) |  |  |
| Female | 1.25 | 1.08 |
| Other | 3.14 | 1.41 |
| Birth year/current age (Ref: 1998-2005; age 18-24) |  |  |
| 1993-1998; age 25-29 | 1.45 | 1.27 |
| 1983-1993; age 30-39 | 1.44 | 1.28 |
| 1973-1983; age 40-49 | 1.50 | 1.34 |
| 1963-1973; age 50-59 | 1.17 | 1.00 |
| 1953-1963; age 60-69 | 1.21 | 1.00 |
| 1943-1953; age 70-79 | 1.45 | 1.00 |
| 1943 or earlier; age 80 or older | 1.35 | 1.00 |
| Mother absence/presence (Ref: Present) |  |  |
| Absent | 1.27 | 1.00 |
| Father absence/presence (Ref: Present) |  |  |
| Absent | 1.30 | 1.00 |
| Childhood religion (Ref: Islam) |  |  |
| Christianity | 1.34 | 1.00 |
| Some other religion | 1.13 | 1.00 |
| Race/ethnicity (Ref: Ethnic plurality) |  |  |
| Ethnic minority | 1.04 | 1.00 |

*Note*. CI, confidence interval. ^a^The formula for calculating *E*-values can be found in VanderWeele and Ding (2017) The *E*-value for the effect estimate is the minimum strength of association (on the risk ratio scale) that an unmeasured confounder would need to have with both the predictor and the outcome to entirely explain away the observed association between them, conditional on the measured covariates. ^b^The *E*-value for the limit of the 95% confidence interval closest to the null denote the minimum strength of association (on the risk ratio scale) that an unmeasured confounder would need to have with both the predictor and the outcome to shift the confidence interval to include the null value, conditional on the measured covariates.

*Table S9a. Nationally Representative Descriptive Statistics of the Observed Sample (Israel)*

| Variable | Proportion | Frequency |
| --- | --- | --- |
| Sociodemographic characteristics |  |  |
| Birth year/current age |  |  |
| 1998-2005; age 18-24 | 0.15 | 553 |
| 1993-1998; age 25-29 | 0.11 | 407 |
| 1983-1993; age 30-39 | 0.18 | 666 |
| 1973-1983; age 40-49 | 0.17 | 616 |
| 1963-1973; age 50-59 | 0.15 | 542 |
| 1953-1963; age 60-69 | 0.13 | 469 |
| 1943-1953; age 70-79 | 0.09 | 336 |
| 1943 or earlier; 80 or older | 0.02 | 79 |
| Missing | . | . |
| Gender |  |  |
| Male | 0.49 | 1791 |
| Female | 0.51 | 1872 |
| Other | 0.00 | 0 |
| Missing | 0.00 | 6 |
| Marital status |  |  |
| Single, never married | 0.23 | 834 |
| Married | 0.56 | 2056 |
| Separated | 0.01 | 48 |
| Divorced | 0.07 | 258 |
| Widowed | 0.06 | 212 |
| Domestic partner | 0.05 | 193 |
| Missing | 0.02 | 69 |
| Employment |  |  |
| Employed for an employer | 0.49 | 1793 |
| Self-employed | 0.12 | 424 |
| Retired | 0.16 | 576 |
| Student | 0.11 | 388 |
| Homemaker | 0.06 | 211 |
| Unemployed and looking for a job | 0.04 | 148 |
| None of these/other | 0.03 | 118 |
| Missing | 0.00 | 10 |
| Education |  |  |
| Up to 8 years | 0.06 | 224 |
| 9-15 years | 0.41 | 1517 |
| 16+ years | 0.52 | 1926 |
| Missing | 0.00 | 2 |
| Religious service attendance |  |  |
| >1x/week | 0.18 | 649 |
| 1x/week | 0.14 | 495 |
| 1-3x/month | 0.10 | 374 |
| A few times a year | 0.28 | 1014 |
| Never | 0.31 | 1122 |
| Missing | 0.00 | 14 |
| Immigration status |  |  |
| Born in this country | 0.76 | 2796 |
| Born in another country | 0.24 | 868 |
| Missing | 0.00 | 5 |
| Religion |  |  |
| Christianity | 0.01 | 39 |
| Islam | 0.18 | 656 |
| Hinduism | . | . |
| Buddhism | . | . |
| Judaism | 0.79 | 2897 |
| Sikhism | . | . |
| Baha'i | 0.00 | 2 |
| Jainism | . | . |
| Shinto | . | . |
| Taoism | 0.00 | 1 |
| Confucianism | . | . |
| Primal, Animist, or Folk Religion | 0.00 | 1 |
| Spiritism | . | . |
| African-derived | . | . |
| Chinese | . | . |
| Some other religion | 0.00 | 5 |
| No religion/atheist/agnostic | 0.02 | 64 |
| Missing | 0.00 | 4 |
| Race/ethnicity |  |  |
| Jewish | 0.80 | 2926 |
| Arab | 0.18 | 674 |
| Other | 0.01 | 39 |
| Missing | 0.01 | 30 |
| Childhood factors |  |  |
| Relationship with mother |  |  |
| Very good | 0.73 | 2686 |
| Somewhat good | 0.22 | 793 |
| Somewhat bad | 0.03 | 110 |
| Very bad | 0.00 | 18 |
| Not applicable | 0.01 | 45 |
| Missing | 0.00 | 17 |
| Relationship with father |  |  |
| Very good | 0.62 | 2290 |
| Somewhat good | 0.25 | 912 |
| Somewhat bad | 0.06 | 234 |
| Very bad | 0.01 | 37 |
| Not applicable | 0.05 | 171 |
| Missing | 0.01 | 25 |
| Parent marital status |  |  |
| Married | 0.86 | 3172 |
| Divorced | 0.08 | 284 |
| Never married | 0.01 | 36 |
| One or both parents had died | 0.04 | 130 |
| Missing | 0.01 | 47 |
| Subjective financial status growing up |  |  |
| Lived comfortably | 0.25 | 923 |
| Got by | 0.50 | 1822 |
| Found it difficult | 0.18 | 667 |
| Found it very difficult | 0.07 | 239 |
| Missing | 0.00 | 17 |
| Childhood abuse |  |  |
| Yes | . | . |
| No | . | . |
| Missing | . | . |
| Outsider growing up |  |  |
| Yes | 0.10 | 371 |
| No | 0.88 | 3228 |
| Not applicable | 0.01 | 36 |
| Missing | 0.01 | 34 |
| Childhood health |  |  |
| Excellent | 0.49 | 1785 |
| Very good | 0.35 | 1284 |
| Good | 0.13 | 480 |
| Fair | 0.03 | 105 |
| Poor | 0.00 | 6 |
| Missing | 0.00 | 8 |
| Childhood religious service attendance |  |  |
| At least 1x/week | 0.24 | 867 |
| 1-3x/month | 0.12 | 435 |
| <1x/month | 0.22 | 810 |
| Never | 0.42 | 1539 |
| Missing | 0.00 | 17 |
| Childhood religion |  |  |
| Christianity | 0.02 | 60 |
| Islam | 0.18 | 647 |
| Hinduism | . | . |
| Buddhism | . | . |
| Judaism | 0.78 | 2873 |
| Sikhism | 0.00 | 1 |
| Baha'i | 0.00 | 1 |
| Jainism | . | . |
| Shinto | . | . |
| Taoism | . | . |
| Confucianism | . | . |
| Primal, Animist, or Folk Religion | 0.00 | 3 |
| Spiritism | . | . |
| African-derived | . | . |
| Chinese | . | . |
| Some other religion | 0.00 | 5 |
| No religion/atheist/agnostic | 0.02 | 69 |
| Missing | 0.00 | 10 |

*Note. N* = 3,669.

*Table S9b. Variations Across Sociodemographic Characteristics (Israel)*

| Characteristic | Mean | SE | LCI | UCI | Global *p*-value |
| --- | --- | --- | --- | --- | --- |
| Age group |  |  |  |  |  |
| 18-24 | 8.46 | 0.07 | 8.33 | 8.59 | 0.01 |
| 25-29 | 8.81 | 0.09 | 8.63 | 8.99 | . |
| 30-39 | 8.57 | 0.11 | 8.35 | 8.79 | . |
| 40-49 | 8.62 | 0.09 | 8.43 | 8.80 | . |
| 50-59 | 8.51 | 0.11 | 8.30 | 8.73 | . |
| 60-69 | 8.71 | 0.11 | 8.48 | 8.93 | . |
| 70-79 | 8.57 | 0.13 | 8.30 | 8.83 | . |
| 80 or older | 8.66 | 0.20 | 8.24 | 9.07 | . |
| Gender |  |  |  |  |  |
| Male | 8.41 | 0.08 | 8.26 | 8.57 | 0.00 |
| Female | 8.77 | 0.06 | 8.65 | 8.89 | . |
| Other | 7.00 | . | . | . | . |
| Marital status |  |  |  |  |  |
| Single, never married | 8.33 | 0.08 | 8.17 | 8.50 | 0.00 |
| Married | 8.76 | 0.07 | 8.62 | 8.89 | . |
| Separated | 8.31 | 0.16 | 7.96 | 8.65 | . |
| Divorced | 8.19 | 0.15 | 7.89 | 8.49 | . |
| Widowed | 8.76 | 0.18 | 8.40 | 9.12 | . |
| Domestic partner | 8.46 | 0.13 | 8.19 | 8.74 | . |
| Employment |  |  |  |  |  |
| Employed for an employer | 8.62 | 0.07 | 8.48 | 8.77 | 0.19 |
| Self-employed | 8.69 | 0.10 | 8.49 | 8.90 | . |
| Retired | 8.65 | 0.13 | 8.39 | 8.91 | . |
| Student | 8.52 | 0.10 | 8.33 | 8.72 | . |
| Homemaker | 8.55 | 0.21 | 8.11 | 8.99 | . |
| Unemployed and looking for a job | 8.13 | 0.23 | 7.66 | 8.59 | . |
| None of these/other | 8.49 | 0.19 | 8.12 | 8.87 | . |
| Education |  |  |  |  |  |
| Up to 8 years | 8.15 | 0.19 | 7.75 | 8.54 | 0.11 |
| 9-15 years | 8.57 | 0.08 | 8.42 | 8.72 | . |
| 16+ years | 8.67 | 0.07 | 8.54 | 8.81 | . |
| Religious service attendance |  |  |  |  |  |
| >1x/week | 8.88 | 0.08 | 8.73 | 9.03 | 0.00 |
| 1x/week | 8.74 | 0.13 | 8.49 | 9.00 | . |
| 1-3x/month | 8.56 | 0.13 | 8.30 | 8.83 | . |
| A few times a year | 8.52 | 0.09 | 8.34 | 8.70 | . |
| Never | 8.45 | 0.09 | 8.28 | 8.63 | . |
| Immigration status |  |  |  |  |  |
| Born in this country | 8.60 | 0.07 | 8.47 | 8.73 | 0.92 |
| Born in another country | 8.59 | 0.10 | 8.39 | 8.79 | . |
| Religion |  |  |  |  |  |
| Christianity | 8.37 | 0.23 | 7.62 | 9.11 | 0.00 |
| Islam | 7.99 | 0.16 | 7.64 | 8.33 | . |
| Hinduism | . | . | . | . | . |
| Buddhism | . | . | . | . | . |
| Judaism | 8.75 | 0.07 | 8.62 | 8.88 | . |
| Sikhism | . | . | . | . | . |
| Baha'i | 10.00 | . | . | . | . |
| Jainism | . | . | . | . | . |
| Shinto | . | . | . | . | . |
| Taoism | 9.00 | . | . | . | . |
| Confucianism | . | . | . | . | . |
| Primal, Animist, or Folk Religion | 8.00 | . | . | . | . |
| Spiritism | . | . | . | . | . |
| African-derived | . | . | . | . | . |
| Chinese | . | . | . | . | . |
| Some other religion | 8.91 | . | . | . | . |
| No religion/atheist/agnostic | 7.89 | 0.21 | 7.41 | 8.37 | . |
| Race/ethnicity |  |  |  |  |  |
| Jewish | 8.75 | 0.07 | 8.62 | 8.88 | 0.00 |
| Arab | 7.97 | 0.16 | 7.63 | 8.31 | . |
| Other | 8.21 | 0.30 | 7.51 | 8.90 | . |

*Note*. *N* = 3,669. SE, standard error; LCI, lower confidence interval; UCI, upper confidence interval.

*Table 9c. Variations Across Childhood Predictors (Israel)*

| Characteristic | Coef. | SE | Prob. | LCI | UCI | Global *p*-value |
| --- | --- | --- | --- | --- | --- | --- |
| Relationship with mother (Ref: Very/somewhat bad) |  |  |  |  |  |  |
| Very/somewhat good | -0.14 | 0.19 | 0.47 | -0.52 | 0.24 | 0.47 |
| Relationship with father (Ref: Very/somewhat bad) |  |  |  |  |  |  |
| Very/somewhat good | 0.16 | 0.19 | 0.41 | -0.22 | 0.53 | 0.41 |
| Parent marital status (Ref: Married) |  |  |  |  |  |  |
| Divorced | -0.45 | 0.12 | 0.00 | -0.68 | -0.21 | 0.00 |
| Never married | -0.45 | 0.28 | 0.11 | -0.99 | 0.10 | . |
| One or both parents had died | 0.02 | 0.14 | 0.87 | -0.26 | 0.31 | . |
| Subjective financial status growing up (Ref: Got by) |  |  |  |  |  |  |
| Lived comfortably | 0.03 | 0.07 | 0.63 | -0.11 | 0.18 | 0.03 |
| Found it difficult | 0.19 | 0.08 | 0.03 | 0.02 | 0.35 | . |
| Found it very difficult | -0.26 | 0.20 | 0.19 | -0.66 | 0.13 | . |
| Childhood abuse (Ref: No) |  |  |  |  |  |  |
| Yes | . | . | . | . | . | . |
| Outsider growing up (Ref: No) |  |  |  |  |  |  |
| Yes | -0.30 | 0.12 | 0.01 | -0.53 | -0.06 | 0.01 |
| Childhood health (Ref: Good) |  |  |  |  |  |  |
| Excellent | 0.32 | 0.16 | 0.04 | 0.01 | 0.63 | 0.00 |
| Very good | 0.31 | 0.14 | 0.03 | 0.03 | 0.59 | . |
| Fair | -0.06 | 0.24 | 0.82 | -0.53 | 0.42 | . |
| Poor | 1.89 | 0.53 | 0.00 | 0.84 | 2.93 | . |
| Immigration status (Ref: Born in this country) |  |  |  |  |  |  |
| Born in another country | -0.03 | 0.09 | 0.77 | -0.20 | 0.15 | 0.77 |
| Childhood religious service attendance (Ref: Never) |  |  |  |  |  |  |
| At least 1x/week | 0.06 | 0.12 | 0.58 | -0.16 | 0.29 | 0.00 |
| 1-3x/month | 0.35 | 0.11 | 0.00 | 0.14 | 0.57 | . |
| <1x/month | 0.27 | 0.09 | 0.00 | 0.09 | 0.44 | . |
| Gender (Ref: Male) |  |  |  |  |  |  |
| Female | 0.36 | 0.07 | 0.00 | 0.22 | 0.50 | 0.00 |
| Other | -1.60 | 0.18 | 0.00 | -1.97 | -1.24 | . |
| Birth year/current age (Ref: 1998-2005; age 18-24) |  |  |  |  |  |  |
| 1993-1998; age 25-29 | 0.31 | 0.10 | 0.00 | 0.11 | 0.50 | 0.04 |
| 1983-1993; age 30-39 | 0.12 | 0.12 | 0.34 | -0.13 | 0.36 | . |
| 1973-1983; age 40-49 | 0.13 | 0.11 | 0.23 | -0.09 | 0.35 | . |
| 1963-1973; age 50-59 | 0.01 | 0.12 | 0.94 | -0.23 | 0.24 | . |
| 1953-1963; age 60-69 | 0.16 | 0.11 | 0.14 | -0.05 | 0.37 | . |
| 1943-1953; age 70-79 | -0.02 | 0.14 | 0.90 | -0.29 | 0.26 | . |
| 1943 or earlier; age 80 or older | 0.17 | 0.22 | 0.45 | -0.28 | 0.62 | . |
| Mother absence/presence (Ref: Present) |  |  |  |  |  |  |
| Absent | -0.01 | 0.27 | 0.96 | -0.55 | 0.52 | 0.96 |
| Father absence/presence (Ref: Present) |  |  |  |  |  |  |
| Absent | 0.25 | 0.21 | 0.22 | -0.16 | 0.67 | 0.22 |
| Childhood religion (Ref: Judaism) |  |  |  |  |  |  |
| Islam | -0.36 | 0.33 | 0.28 | -1.02 | 0.30 | 0.52 |
| Some other religion | -0.18 | 0.22 | 0.40 | -0.61 | 0.25 | . |
| Race/ethnicity (Ref: Ethnic plurality) |  |  |  |  |  |  |
| Ethnic minority | -0.46 | 0.32 | 0.15 | -1.10 | 0.17 | 0.15 |
| *Note*. *N* = 3,669. SE, standard error; LCI, lower confidence interval; UCI, upper confidence interval. | | | | | | |

*Table 9d. E-Values and E-Value Limits for the Coefficients Shown in Table 9c (Israel)*

| Characteristic | *E*-value for estimate^a^ | *E*-value for 95% CI^b^ |
| --- | --- | --- |
| Relationship with mother (Ref: Very/somewhat bad) |  |  |
| Very/somewhat good | 1.26 | 1.00 |
| Relationship with father (Ref: Very/somewhat bad) |  |  |
| Very/somewhat good | 1.28 | 1.00 |
| Parent marital status (Ref: Married) |  |  |
| Divorced | 1.56 | 1.34 |
| Never married | 1.56 | 1.00 |
| One or both parents had died | 1.09 | 1.00 |
| Subjective financial status growing up (Ref: Got by) |  |  |
| Lived comfortably | 1.12 | 1.00 |
| Found it difficult | 1.31 | 1.09 |
| Found it very difficult | 1.39 | 1.00 |
| Childhood abuse (Ref: No) |  |  |
| Yes | . | . |
| Outsider growing up (Ref: No) |  |  |
| Yes | 1.42 | 1.17 |
| Childhood health (Ref: Good) |  |  |
| Excellent | 1.44 | 1.07 |
| Very good | 1.43 | 1.11 |
| Fair | 1.15 | 1.00 |
| Poor | 2.99 | 1.93 |
| Immigration status (Ref: Born in this country) |  |  |
| Born in another country | 1.10 | 1.00 |
| Childhood religious service attendance (Ref: Never) |  |  |
| At least 1x/week | 1.16 | 1.00 |
| 1-3x/month | 1.48 | 1.26 |
| <1x/month | 1.39 | 1.21 |
| Gender (Ref: Male) |  |  |
| Female | 1.48 | 1.35 |
| Other | 2.67 | 2.31 |
| Birth year/current age (Ref: 1998-2005; age 18-24) |  |  |
| 1993-1998; age 25-29 | 1.43 | 1.23 |
| 1983-1993; age 30-39 | 1.24 | 1.00 |
| 1973-1983; age 40-49 | 1.25 | 1.00 |
| 1963-1973; age 50-59 | 1.05 | 1.00 |
| 1953-1963; age 60-69 | 1.28 | 1.00 |
| 1943-1953; age 70-79 | 1.08 | 1.00 |
| 1943 or earlier; age 80 or older | 1.29 | 1.00 |
| Mother absence/presence (Ref: Present) |  |  |
| Absent | 1.07 | 1.00 |
| Father absence/presence (Ref: Present) |  |  |
| Absent | 1.38 | 1.00 |
| Childhood religion (Ref: Judaism) |  |  |
| Islam | 1.48 | 1.00 |
| Some other religion | 1.31 | 1.00 |
| Race/ethnicity (Ref: Ethnic plurality) |  |  |
| Ethnic minority | 1.58 | 1.00 |

*Note.* CI, confidence interval. ^a^The formula for calculating *E*-values can be found in VanderWeele and Ding (2017) The *E*-value for the effect estimate is the minimum strength of association (on the risk ratio scale) that an unmeasured confounder would need to have with both the predictor and the outcome to entirely explain away the observed association between them, conditional on the measured covariates. ^b^The *E*-value for the limit of the 95% confidence interval closest to the null denote the minimum strength of association (on the risk ratio scale) that an unmeasured confounder would need to have with both the predictor and the outcome to shift the confidence interval to include the null value, conditional on the measured covariates.

*Table S10a. Nationally Representative Descriptive Statistics of the Observed Sample (Japan)*

| Variable | Proportion | Frequency |
| --- | --- | --- |
| Sociodemographic characteristics |  |  |
| Birth year/current age |  |  |
| 1998-2005; age 18-24 | 0.08 | 1589 |
| 1993-1998; age 25-29 | 0.04 | 806 |
| 1983-1993; age 30-39 | 0.14 | 2851 |
| 1973-1983; age 40-49 | 0.16 | 3363 |
| 1963-1973; age 50-59 | 0.18 | 3770 |
| 1953-1963; age 60-69 | 0.20 | 4118 |
| 1943-1953; age 70-79 | 0.17 | 3554 |
| 1943 or earlier; 80 or older | 0.02 | 493 |
| Missing | . | . |
| Gender |  |  |
| Male | 0.48 | 9847 |
| Female | 0.52 | 10602 |
| Other | 0.00 | 28 |
| Missing | 0.00 | 66 |
| Marital status |  |  |
| Single, never married | 0.24 | 5004 |
| Married | 0.58 | 11837 |
| Separated | 0.01 | 190 |
| Divorced | 0.10 | 2126 |
| Widowed | 0.06 | 1179 |
| Domestic partner | 0.01 | 144 |
| Missing | 0.00 | 64 |
| Employment |  |  |
| Employed for an employer | 0.53 | 10853 |
| Self-employed | 0.09 | 1748 |
| Retired | 0.12 | 2535 |
| Student | 0.02 | 491 |
| Homemaker | 0.06 | 1276 |
| Unemployed and looking for a job | 0.03 | 622 |
| None of these/other | 0.15 | 2983 |
| Missing | 0.00 | 36 |
| Education |  |  |
| Up to 8 years | 0.03 | 567 |
| 9-15 years | 0.72 | 14893 |
| 16+ years | 0.25 | 5083 |
| Missing | . | . |
| Religious service attendance |  |  |
| >1x/week | 0.02 | 316 |
| 1x/week | 0.02 | 348 |
| 1-3x/month | 0.04 | 862 |
| A few times a year | 0.15 | 3112 |
| Never | 0.77 | 15788 |
| Missing | 0.01 | 117 |
| Immigration status |  |  |
| Born in this country | 0.95 | 19548 |
| Born in another country | 0.01 | 158 |
| Missing | 0.04 | 837 |
| Religion |  |  |
| Christianity | 0.02 | 381 |
| Islam | 0.00 | 10 |
| Hinduism | 0.00 | 5 |
| Buddhism | 0.33 | 6709 |
| Judaism | 0.00 | 10 |
| Sikhism | 0.00 | 6 |
| Baha'i | 0.00 | 2 |
| Jainism | 0.00 | 11 |
| Shinto | 0.02 | 469 |
| Taoism | 0.00 | 7 |
| Confucianism | 0.00 | 17 |
| Primal, Animist, or Folk Religion | 0.00 | 19 |
| Spiritism | . | . |
| African-derived | . | . |
| Chinese | . | . |
| Some other religion | 0.00 | 46 |
| No religion/atheist/agnostic | 0.61 | 12497 |
| Missing | 0.02 | 355 |
| Race/ethnicity |  |  |
| No data | . | . |
| Childhood factors |  |  |
| Relationship with mother |  |  |
| Very good | 0.27 | 5630 |
| Somewhat good | 0.46 | 9461 |
| Somewhat bad | 0.13 | 2750 |
| Very bad | 0.04 | 799 |
| Not applicable | 0.09 | 1838 |
| Missing | 0.00 | 66 |
| Relationship with father |  |  |
| Very good | 0.20 | 4156 |
| Somewhat good | 0.44 | 9081 |
| Somewhat bad | 0.17 | 3446 |
| Very bad | 0.06 | 1223 |
| Not applicable | 0.13 | 2580 |
| Missing | 0.00 | 57 |
| Parent marital status |  |  |
| Married | 0.86 | 17713 |
| Divorced | 0.05 | 1127 |
| Never married | 0.03 | 591 |
| One or both parents had died | 0.04 | 754 |
| Missing | 0.02 | 359 |
| Subjective financial status growing up |  |  |
| Lived comfortably | 0.41 | 8320 |
| Got by | 0.43 | 8799 |
| Found it difficult | 0.12 | 2398 |
| Found it very difficult | 0.05 | 973 |
| Missing | 0.00 | 52 |
| Childhood abuse |  |  |
| Yes | 0.07 | 1482 |
| No | 0.92 | 18964 |
| Missing | 0.00 | 96 |
| Outsider growing up |  |  |
| Yes | 0.10 | 1963 |
| No | 0.83 | 17136 |
| Not applicable | 0.07 | 1402 |
| Missing | 0.00 | 42 |
| Childhood health |  |  |
| Excellent | 0.13 | 2711 |
| Very good | 0.35 | 7106 |
| Good | 0.33 | 6689 |
| Fair | 0.16 | 3199 |
| Poor | 0.04 | 758 |
| Missing | 0.00 | 80 |
| Childhood religious service attendance |  |  |
| At least 1x/week | 0.02 | 398 |
| 1-3x/month | 0.04 | 883 |
| <1x/month | 0.24 | 5023 |
| Never | 0.69 | 14117 |
| Missing | 0.01 | 123 |
| Childhood religion |  |  |
| Christianity | 0.02 | 343 |
| Islam | 0.00 | 7 |
| Hinduism | 0.00 | 4 |
| Buddhism | 0.32 | 6536 |
| Judaism | . | . |
| Sikhism | . | . |
| Baha'i | 0.00 | 7 |
| Jainism | 0.00 | 1 |
| Shinto | 0.02 | 382 |
| Taoism | 0.00 | 14 |
| Confucianism | 0.00 | 25 |
| Primal, Animist, or Folk Religion | 0.00 | 13 |
| Spiritism | . | . |
| African-derived | . | . |
| Chinese | . | . |
| Some other religion | 0.00 | 46 |
| No religion/atheist/agnostic | 0.63 | 12950 |
| Missing | 0.01 | 215 |

*Note*. *N* = 20,543.

*Table S10b. Variations Across Sociodemographic Characteristics (Japan)*

| Characteristic | Mean | SE | LCI | UCI | Global *p*-value |
| --- | --- | --- | --- | --- | --- |
| Age group |  |  |  |  |  |
| 18-24 | 5.95 | 0.07 | 5.81 | 6.09 | 0.00 |
| 25-29 | 5.82 | 0.09 | 5.63 | 6.00 | . |
| 30-39 | 5.66 | 0.05 | 5.56 | 5.76 | . |
| 40-49 | 5.66 | 0.05 | 5.56 | 5.75 | . |
| 50-59 | 5.69 | 0.04 | 5.61 | 5.76 | . |
| 60-69 | 6.04 | 0.04 | 5.96 | 6.11 | . |
| 70-79 | 6.59 | 0.04 | 6.52 | 6.66 | . |
| 80 or older | 6.78 | 0.12 | 6.55 | 7.01 | . |
| Gender |  |  |  |  |  |
| Male | 5.61 | 0.02 | 5.56 | 5.66 | 0.00 |
| Female | 6.28 | 0.02 | 6.23 | 6.33 | . |
| Other | 5.73 | 0.37 | 4.98 | 6.47 | . |
| Marital status |  |  |  |  |  |
| Single, never married | 5.25 | 0.03 | 5.18 | 5.31 | 0.00 |
| Married | 6.20 | 0.02 | 6.16 | 6.24 | . |
| Separated | 5.94 | 0.20 | 5.56 | 6.33 | . |
| Divorced | 5.86 | 0.06 | 5.73 | 5.98 | . |
| Widowed | 6.72 | 0.08 | 6.56 | 6.89 | . |
| Domestic partner | 5.74 | 0.29 | 5.17 | 6.31 | . |
| Employment |  |  |  |  |  |
| Employed for an employer | 5.83 | 0.03 | 5.78 | 5.88 | 0.00 |
| Self-employed | 6.08 | 0.06 | 5.96 | 6.21 | . |
| Retired | 6.16 | 0.04 | 6.07 | 6.24 | . |
| Student | 6.27 | 0.10 | 6.07 | 6.47 | . |
| Homemaker | 6.42 | 0.06 | 6.30 | 6.54 | . |
| Unemployed and looking for a job | 4.63 | 0.10 | 4.45 | 4.82 | . |
| None of these/other | 6.19 | 0.04 | 6.12 | 6.27 | . |
| Education |  |  |  |  |  |
| Up to 8 years | 5.25 | 0.12 | 5.01 | 5.49 | 0.00 |
| 9-15 years | 5.87 | 0.02 | 5.82 | 5.91 | . |
| 16+ years | 6.30 | 0.04 | 6.23 | 6.37 | . |
| Religious service attendance |  |  |  |  |  |
| >1x/week | 7.28 | 0.14 | 7.01 | 7.55 | 0.00 |
| 1x/week | 6.91 | 0.14 | 6.63 | 7.18 | . |
| 1-3x/month | 6.24 | 0.08 | 6.09 | 6.39 | . |
| A few times a year | 6.27 | 0.04 | 6.19 | 6.35 | . |
| Never | 5.83 | 0.02 | 5.79 | 5.87 | . |
| Immigration status |  |  |  |  |  |
| Born in this country | 5.96 | 0.02 | 5.92 | 5.99 | 0.96 |
| Born in another country | 5.97 | 0.19 | 5.58 | 6.35 | . |
| Religion |  |  |  |  |  |
| Christianity | 7.04 | 0.12 | 6.81 | 7.28 | 0.00 |
| Islam | 6.09 | 0.97 | 3.69 | 8.49 | . |
| Hinduism | 6.37 | 1.11 | 2.70 | 10.03 | . |
| Buddhism | 6.27 | 0.03 | 6.22 | 6.33 | . |
| Judaism | 5.04 | 0.47 | 3.49 | 6.58 | . |
| Sikhism | 6.24 | 0.64 | 4.65 | 7.84 | . |
| Baha'i | 4.84 | 0.27 | -38.93 | 48.61 | . |
| Jainism | 4.80 | 1.31 | 1.07 | 8.52 | . |
| Shinto | 6.27 | 0.13 | 6.01 | 6.52 | . |
| Taoism | 7.20 | 0.57 | 5.87 | 8.53 | . |
| Confucianism | 6.33 | 0.50 | 5.27 | 7.39 | . |
| Primal, Animist, or Folk Religion | 5.32 | 0.36 | 4.53 | 6.12 | . |
| Spiritism | . | . | . | . | . |
| African-derived | . | . | . | . | . |
| Chinese | . | . | . | . | . |
| Some other religion | 5.60 | 0.64 | 4.31 | 6.90 | . |
| No religion/atheist/agnostic | 5.74 | 0.02 | 5.70 | 5.79 | . |
| Race/ethnicity |  |  |  |  |  |
| No data | . | . | . | . | . |

*Note*. *N* = 20,543. SE, standard error; LCI, lower confidence interval; UCI, upper confidence interval.

*Table S10c. Variations Across Childhood Predictors (Japan)*

| Characteristic | Coef. | SE | Prob. | LCI | UCI | Global *p*-value |
| --- | --- | --- | --- | --- | --- | --- |
| Relationship with mother (Ref: Very/somewhat bad) |  |  |  |  |  |  |
| Very/somewhat good | 0.26 | 0.05 | 0.00 | 0.16 | 0.36 | 0.00 |
| Relationship with father (Ref: Very/somewhat bad) |  |  |  |  |  |  |
| Very/somewhat good | 0.37 | 0.04 | 0.00 | 0.28 | 0.46 | 0.00 |
| Parent marital status (Ref: Married) |  |  |  |  |  |  |
| Divorced | 0.14 | 0.08 | 0.11 | -0.03 | 0.30 | 0.24 |
| Never married | -0.08 | 0.10 | 0.44 | -0.27 | 0.12 | . |
| One or both parents had died | 0.10 | 0.09 | 0.29 | -0.09 | 0.28 | . |
| Subjective financial status growing up (Ref: Got by) |  |  |  |  |  |  |
| Lived comfortably | 0.40 | 0.04 | 0.00 | 0.33 | 0.48 | 0.00 |
| Found it difficult | -0.12 | 0.06 | 0.03 | -0.23 | -0.01 | . |
| Found it very difficult | -0.29 | 0.11 | 0.01 | -0.49 | -0.08 | . |
| Childhood abuse (Ref: No) |  |  |  |  |  |  |
| Yes | -0.10 | 0.08 | 0.21 | -0.26 | 0.06 | 0.21 |
| Outsider growing up (Ref: No) |  |  |  |  |  |  |
| Yes | -0.21 | 0.07 | 0.00 | -0.35 | -0.08 | 0.00 |
| Childhood health (Ref: Good) |  |  |  |  |  |  |
| Excellent | 1.03 | 0.06 | 0.00 | 0.91 | 1.14 | 0.00 |
| Very good | 0.39 | 0.04 | 0.00 | 0.31 | 0.46 | . |
| Fair | -0.35 | 0.05 | 0.00 | -0.45 | -0.25 | . |
| Poor | -0.51 | 0.12 | 0.00 | -0.75 | -0.28 | . |
| Immigration status (Ref: Born in this country) |  |  |  |  |  |  |
| Born in another country | 0.16 | 0.17 | 0.33 | -0.16 | 0.49 | 0.33 |
| Childhood religious service attendance (Ref: Never) |  |  |  |  |  |  |
| At least 1x/week | 0.74 | 0.13 | 0.00 | 0.48 | 1.00 | 0.00 |
| 1-3x/month | 0.73 | 0.08 | 0.00 | 0.58 | 0.89 | . |
| <1x/month | 0.35 | 0.04 | 0.00 | 0.27 | 0.42 | . |
| Gender (Ref: Male) |  |  |  |  |  |  |
| Female | 0.64 | 0.03 | 0.00 | 0.57 | 0.70 | 0.00 |
| Other | 0.82 | 0.34 | 0.02 | 0.16 | 1.49 | . |
| Birth year/current age (Ref: 1998-2005; age 18-24) |  |  |  |  |  |  |
| 1993-1998; age 25-29 | -0.11 | 0.11 | 0.29 | -0.32 | 0.09 | 0.00 |
| 1983-1993; age 30-39 | -0.19 | 0.08 | 0.02 | -0.35 | -0.04 | . |
| 1973-1983; age 40-49 | -0.24 | 0.08 | 0.00 | -0.39 | -0.08 | . |
| 1963-1973; age 50-59 | -0.18 | 0.08 | 0.02 | -0.32 | -0.03 | . |
| 1953-1963; age 60-69 | 0.06 | 0.07 | 0.42 | -0.09 | 0.21 | . |
| 1943-1953; age 70-79 | 0.52 | 0.07 | 0.00 | 0.37 | 0.67 | . |
| 1943 or earlier; age 80 or older | 0.74 | 0.14 | 0.00 | 0.47 | 1.01 | . |
| Mother absence/presence (Ref: Present) |  |  |  |  |  |  |
| Absent | -0.03 | 0.07 | 0.70 | -0.16 | 0.11 | 0.70 |
| Father absence/presence (Ref: Present) |  |  |  |  |  |  |
| Absent | 0.09 | 0.06 | 0.16 | -0.03 | 0.21 | 0.16 |
| Childhood religion (Ref: No religion/atheist/agnostic) |  |  |  |  |  |  |
| Buddhism | 0.22 | 0.04 | 0.00 | 0.15 | 0.29 | 0.00 |
| Some other religion | 0.15 | 0.10 | 0.15 | -0.05 | 0.34 | . |
| *Note*. *N* = 20,543. SE, standard error; LCI, lower confidence interval; UCI, upper confidence interval. | | | | | | |

*Table S10d. E-Values and E-Value Limits for the Coefficients Shown in Table S10c (Japan)*

| Characteristic | *E*-value for estimate^a^ | *E*-value for 95% CI^b^ |
| --- | --- | --- |
| Relationship with mother (Ref: Very/somewhat bad) |  |  |
| Very/somewhat good | 1.44 | 1.32 |
| Relationship with father (Ref: Very/somewhat bad) |  |  |
| Very/somewhat good | 1.56 | 1.46 |
| Parent marital status (Ref: Married) |  |  |
| Divorced | 1.29 | 1.00 |
| Never married | 1.20 | 1.00 |
| One or both parents had died | 1.24 | 1.00 |
| Subjective financial status growing up (Ref: Got by) |  |  |
| Lived comfortably | 1.60 | 1.52 |
| Found it difficult | 1.27 | 1.08 |
| Found it very difficult | 1.47 | 1.21 |
| Childhood abuse (Ref: No) |  |  |
| Yes | 1.24 | 1.00 |
| Outsider growing up (Ref: No) |  |  |
| Yes | 1.38 | 1.21 |
| Childhood health (Ref: Good) |  |  |
| Excellent | 2.29 | 2.15 |
| Very good | 1.58 | 1.50 |
| Fair | 1.53 | 1.42 |
| Poor | 1.72 | 1.46 |
| Immigration status (Ref: Born in this country) |  |  |
| Born in another country | 1.32 | 1.00 |
| Childhood religious service attendance (Ref: Never) |  |  |
| At least 1x/week | 1.96 | 1.68 |
| 1-3x/month | 1.96 | 1.78 |
| <1x/month | 1.54 | 1.45 |
| Gender (Ref: Male) |  |  |
| Female | 1.85 | 1.78 |
| Other | 2.06 | 1.31 |
| Birth year/current age (Ref: 1998-2005; age 18-24) |  |  |
| 1993-1998; age 25-29 | 1.26 | 1.00 |
| 1983-1993; age 30-39 | 1.36 | 1.13 |
| 1973-1983; age 40-49 | 1.41 | 1.21 |
| 1963-1973; age 50-59 | 1.34 | 1.12 |
| 1953-1963; age 60-69 | 1.17 | 1.00 |
| 1943-1953; age 70-79 | 1.72 | 1.56 |
| 1943 or earlier; age 80 or older | 1.96 | 1.67 |
| Mother absence/presence (Ref: Present) |  |  |
| Absent | 1.11 | 1.00 |
| Father absence/presence (Ref: Present) |  |  |
| Absent | 1.22 | 1.00 |
| Childhood religion (Ref: No religion/atheist/agnostic) |  |  |
| Buddhism | 1.39 | 1.30 |
| Some other religion | 1.30 | 1.00 |

*Note*. CI, confidence interval. ^a^The formula for calculating *E*-values can be found in VanderWeele and Ding (2017) The *E*-value for the effect estimate is the minimum strength of association (on the risk ratio scale) that an unmeasured confounder would need to have with both the predictor and the outcome to entirely explain away the observed association between them, conditional on the measured covariates. ^b^The *E*-value for the limit of the 95% confidence interval closest to the null denote the minimum strength of association (on the risk ratio scale) that an unmeasured confounder would need to have with both the predictor and the outcome to shift the confidence interval to include the null value, conditional on the measured covariates.

*Table S11a. Nationally Representative Descriptive Statistics of the Observed Sample (Kenya)*

| Variable | Proportion | Frequency |
| --- | --- | --- |
| Sociodemographic characteristics |  |  |
| Birth year/current age |  |  |
| 1998-2005; age 18-24 | 0.25 | 2868 |
| 1993-1998; age 25-29 | 0.18 | 2035 |
| 1983-1993; age 30-39 | 0.23 | 2564 |
| 1973-1983; age 40-49 | 0.15 | 1708 |
| 1963-1973; age 50-59 | 0.09 | 1072 |
| 1953-1963; age 60-69 | 0.06 | 710 |
| 1943-1953; age 70-79 | 0.03 | 360 |
| 1943 or earlier; 80 or older | 0.01 | 67 |
| Missing | 0.00 | 5 |
| Gender |  |  |
| Male | 0.49 | 5567 |
| Female | 0.51 | 5813 |
| Other | 0.00 | 2 |
| Missing | 0.00 | 7 |
| Marital status |  |  |
| Single, never married | 0.31 | 3531 |
| Married | 0.58 | 6626 |
| Separated | 0.04 | 467 |
| Divorced | 0.01 | 111 |
| Widowed | 0.04 | 464 |
| Domestic partner | 0.01 | 146 |
| Missing | 0.00 | 43 |
| Employment |  |  |
| Employed for an employer | 0.13 | 1467 |
| Self-employed | 0.32 | 3630 |
| Retired | 0.03 | 319 |
| Student | 0.10 | 1136 |
| Homemaker | 0.13 | 1537 |
| Unemployed and looking for a job | 0.28 | 3153 |
| None of these/other | 0.01 | 138 |
| Missing | 0.00 | 9 |
| Education |  |  |
| Up to 8 years | 0.39 | 4485 |
| 9-15 years | 0.54 | 6115 |
| 16+ years | 0.07 | 783 |
| Missing | 0.00 | 6 |
| Religious service attendance |  |  |
| >1x/week | 0.24 | 2774 |
| 1x/week | 0.53 | 6063 |
| 1-3x/month | 0.11 | 1219 |
| A few times a year | 0.08 | 855 |
| Never | 0.04 | 465 |
| Missing | 0.00 | 13 |
| Immigration status |  |  |
| Born in this country | 0.99 | 11270 |
| Born in another country | 0.01 | 117 |
| Missing | 0.00 | 2 |
| Religion |  |  |
| Christianity | 0.91 | 10334 |
| Islam | 0.08 | 918 |
| Hinduism | . | . |
| Buddhism | 0.00 | 1 |
| Judaism | 0.00 | 3 |
| Sikhism | . | . |
| Baha'i | 0.00 | 1 |
| Jainism | 0.00 | 1 |
| Shinto | . | . |
| Taoism | . | . |
| Confucianism | 0.00 | 3 |
| Primal, Animist, or Folk Religion | 0.00 | 7 |
| Spiritism | . | . |
| African-derived | . | . |
| Chinese | . | . |
| Some other religion | 0.00 | 5 |
| No religion/atheist/agnostic | 0.01 | 108 |
| Missing | 0.00 | 9 |
| Race/ethnicity |  |  |
| Luhya | 0.17 | 1943 |
| Luo | 0.10 | 1120 |
| Kalenjin | 0.12 | 1377 |
| Kamba | 0.11 | 1299 |
| Kikuyu | 0.19 | 2119 |
| Kisii | 0.07 | 789 |
| Maasai | 0.02 | 237 |
| Meru | 0.06 | 630 |
| Kenan Somali/Somali | 0.03 | 396 |
| Miji Kenda Tribes | 0.06 | 708 |
| Embu | 0.02 | 197 |
| Other | 0.05 | 548 |
| Missing | 0.00 | 27 |
| Childhood factors |  |  |
| Relationship with mother |  |  |
| Very good | 0.83 | 9418 |
| Somewhat good | 0.13 | 1435 |
| Somewhat bad | 0.01 | 130 |
| Very bad | 0.01 | 100 |
| Not applicable | 0.02 | 240 |
| Missing | 0.01 | 66 |
| Relationship with father |  |  |
| Very good | 0.70 | 7958 |
| Somewhat good | 0.17 | 1896 |
| Somewhat bad | 0.02 | 216 |
| Very bad | 0.02 | 220 |
| Not applicable | 0.08 | 967 |
| Missing | 0.01 | 132 |
| Parent marital status |  |  |
| Married | 0.81 | 9238 |
| Divorced | 0.06 | 697 |
| Never married | 0.06 | 681 |
| One or both parents had died | 0.04 | 471 |
| Missing | 0.03 | 301 |
| Subjective financial status growing up |  |  |
| Lived comfortably | 0.27 | 3026 |
| Got by | 0.29 | 3279 |
| Found it difficult | 0.36 | 4071 |
| Found it very difficult | 0.09 | 994 |
| Missing | 0.00 | 19 |
| Childhood abuse |  |  |
| Yes | 0.11 | 1300 |
| No | 0.88 | 10040 |
| Missing | 0.00 | 49 |
| Outsider growing up |  |  |
| Yes | 0.11 | 1223 |
| No | 0.89 | 10114 |
| Not applicable | 0.00 | 23 |
| Missing | 0.00 | 29 |
| Childhood health |  |  |
| Excellent | 0.39 | 4449 |
| Very good | 0.23 | 2598 |
| Good | 0.23 | 2582 |
| Fair | 0.12 | 1384 |
| Poor | 0.03 | 349 |
| Missing | 0.00 | 26 |
| Childhood religious service attendance |  |  |
| At least 1x/week | 0.81 | 9189 |
| 1-3x/month | 0.15 | 1687 |
| <1x/month | 0.02 | 236 |
| Never | 0.02 | 198 |
| Missing | 0.01 | 79 |
| Childhood religion |  |  |
| Christianity | 0.91 | 10369 |
| Islam | 0.08 | 916 |
| Hinduism | . | . |
| Buddhism | 0.00 | 5 |
| Judaism | 0.00 | 6 |
| Sikhism | 0.00 | 0 |
| Baha'i | 0.00 | 3 |
| Jainism | 0.00 | 1 |
| Shinto | . | . |
| Taoism | . | . |
| Confucianism | . | . |
| Primal, Animist, or Folk Religion | 0.00 | 13 |
| Spiritism | . | . |
| African-derived | . | . |
| Chinese | . | . |
| Some other religion | 0.00 | 0 |
| No religion/atheist/agnostic | 0.01 | 67 |
| Missing | 0.00 | 9 |

*Note*. *N* = 11,389.

*Table S11b. Variations Across Sociodemographic Characteristics (Kenya)*

| Characteristic | Mean | SE | LCI | UCI | Global *p*-value |
| --- | --- | --- | --- | --- | --- |
| Age group |  |  |  |  |  |
| 18-24 | 8.47 | 0.05 | 8.37 | 8.58 | 0.21 |
| 25-29 | 8.56 | 0.06 | 8.44 | 8.67 | . |
| 30-39 | 8.61 | 0.06 | 8.49 | 8.73 | . |
| 40-49 | 8.52 | 0.09 | 8.34 | 8.69 | . |
| 50-59 | 8.59 | 0.10 | 8.39 | 8.79 | . |
| 60-69 | 8.79 | 0.12 | 8.54 | 9.04 | . |
| 70-79 | 8.82 | 0.18 | 8.46 | 9.17 | . |
| 80 or older | 8.84 | 0.64 | 7.47 | 10.21 | . |
| Gender |  |  |  |  |  |
| Male | 8.55 | 0.05 | 8.45 | 8.64 | 0.06 |
| Female | 8.59 | 0.04 | 8.50 | 8.68 | . |
| Other | 9.60 | . | . | . | . |
| Marital status |  |  |  |  |  |
| Single, never married | 8.49 | 0.05 | 8.39 | 8.60 | 0.00 |
| Married | 8.62 | 0.04 | 8.54 | 8.71 | . |
| Separated | 8.52 | 0.15 | 8.22 | 8.82 | . |
| Divorced | 7.79 | 0.25 | 7.29 | 8.29 | . |
| Widowed | 8.75 | 0.16 | 8.44 | 9.06 | . |
| Domestic partner | 8.12 | 0.31 | 7.50 | 8.75 | . |
| Employment |  |  |  |  |  |
| Employed for an employer | 8.61 | 0.07 | 8.47 | 8.75 | 0.46 |
| Self-employed | 8.59 | 0.05 | 8.49 | 8.70 | . |
| Retired | 8.35 | 0.20 | 7.95 | 8.75 | . |
| Student | 8.49 | 0.07 | 8.35 | 8.63 | . |
| Homemaker | 8.53 | 0.09 | 8.36 | 8.71 | . |
| Unemployed and looking for a job | 8.61 | 0.06 | 8.50 | 8.72 | . |
| None of these/other | 8.04 | 0.39 | 7.26 | 8.82 | . |
| Education |  |  |  |  |  |
| Up to 8 years | 8.63 | 0.06 | 8.52 | 8.74 | 0.00 |
| 9-15 years | 8.56 | 0.04 | 8.49 | 8.64 | . |
| 16+ years | 8.25 | 0.10 | 8.06 | 8.45 | . |
| Religious service attendance |  |  |  |  |  |
| >1x/week | 8.79 | 0.06 | 8.68 | 8.90 | 0.00 |
| 1x/week | 8.67 | 0.04 | 8.58 | 8.75 | . |
| 1-3x/month | 8.21 | 0.09 | 8.03 | 8.39 | . |
| A few times a year | 8.02 | 0.10 | 7.81 | 8.23 | . |
| Never | 7.92 | 0.17 | 7.58 | 8.27 | . |
| Immigration status |  |  |  |  |  |
| Born in this country | 8.57 | 0.04 | 8.50 | 8.64 | 0.25 |
| Born in another country | 8.16 | 0.36 | 7.43 | 8.89 | . |
| Religion |  |  |  |  |  |
| Christianity | 8.58 | 0.04 | 8.51 | 8.66 | 0.00 |
| Islam | 8.37 | 0.15 | 8.08 | 8.67 | . |
| Hinduism | . | . | . | . | . |
| Buddhism | 10.00 | . | . | . | . |
| Judaism | 10.00 | . | . | . | . |
| Sikhism | . | . | . | . | . |
| Baha'i | 10.00 | . | . | . | . |
| Jainism | 10.00 | . | . | . | . |
| Shinto | . | . | . | . | . |
| Taoism | . | . | . | . | . |
| Confucianism | 9.00 | . | . | . | . |
| Primal, Animist, or Folk Religion | 7.23 | . | . | . | . |
| Spiritism | . | . | . | . | . |
| African-derived | . | . | . | . | . |
| Chinese | . | . | . | . | . |
| Some other religion | 8.86 | 0.18 | 7.32 | 10.41 | . |
| No religion/atheist/agnostic | 8.84 | 0.20 | 8.43 | 9.25 | . |
| Race/ethnicity |  |  |  |  |  |
| Luhya | 8.73 | 0.08 | 8.57 | 8.89 | 0.00 |
| Luo | 8.46 | 0.11 | 8.24 | 8.68 | . |
| Kalenjin | 8.62 | 0.09 | 8.44 | 8.81 | . |
| Kamba | 8.71 | 0.11 | 8.50 | 8.92 | . |
| Kikuyu | 8.50 | 0.08 | 8.34 | 8.65 | . |
| Kisii | 8.09 | 0.12 | 7.86 | 8.32 | . |
| Maasai | 9.01 | 0.22 | 8.56 | 9.46 | . |
| Meru | 8.51 | 0.10 | 8.31 | 8.71 | . |
| Kenan Somali/Somali | 7.96 | 0.26 | 7.40 | 8.53 | . |
| Miji Kenda Tribes | 8.91 | 0.08 | 8.74 | 9.07 | . |
| Embu | 8.72 | 0.13 | 8.45 | 8.98 | . |
| Other | 8.53 | 0.15 | 8.23 | 8.83 | . |

*Note*. *N* = 11,389. SE, standard error; LCI, lower confidence interval; UCI, upper confidence interval.

*Table S11c. Variations Across Childhood Predictors (Kenya)*

| Characteristic | Coef. | SE | Prob. | LCI | UCI | Global *p*-value |
| --- | --- | --- | --- | --- | --- | --- |
| Relationship with mother (Ref: Very/somewhat bad) |  |  |  |  |  |  |
| Very/somewhat good | 0.16 | 0.18 | 0.39 | -0.20 | 0.51 | 0.39 |
| Relationship with father (Ref: Very/somewhat bad) |  |  |  |  |  |  |
| Very/somewhat good | -0.02 | 0.13 | 0.91 | -0.28 | 0.25 | 0.91 |
| Parent marital status (Ref: Married) |  |  |  |  |  |  |
| Divorced | -0.12 | 0.12 | 0.32 | -0.36 | 0.12 | 0.45 |
| Never married | -0.20 | 0.15 | 0.20 | -0.50 | 0.10 | . |
| One or both parents had died | -0.03 | 0.15 | 0.85 | -0.33 | 0.27 | . |
| Subjective financial status growing up (Ref: Got by) |  |  |  |  |  |  |
| Lived comfortably | 0.14 | 0.07 | 0.06 | -0.01 | 0.29 | 0.03 |
| Found it difficult | -0.03 | 0.08 | 0.68 | -0.19 | 0.12 | . |
| Found it very difficult | -0.15 | 0.13 | 0.25 | -0.42 | 0.11 | . |
| Childhood abuse (Ref: No) |  |  |  |  |  |  |
| Yes | -0.38 | 0.09 | 0.00 | -0.57 | -0.20 | 0.00 |
| Outsider growing up (Ref: No) |  |  |  |  |  |  |
| Yes | -0.07 | 0.11 | 0.55 | -0.29 | 0.15 | 0.55 |
| Childhood health (Ref: Good) |  |  |  |  |  |  |
| Excellent | 0.05 | 0.08 | 0.48 | -0.10 | 0.20 | 0.19 |
| Very good | -0.13 | 0.09 | 0.13 | -0.31 | 0.04 | . |
| Fair | -0.05 | 0.09 | 0.57 | -0.23 | 0.13 | . |
| Poor | 0.04 | 0.18 | 0.82 | -0.31 | 0.40 | . |
| Immigration status (Ref: Born in this country) |  |  |  |  |  |  |
| Born in another country | -0.38 | 0.36 | 0.30 | -1.09 | 0.34 | 0.30 |
| Childhood religious service attendance (Ref: Never) |  |  |  |  |  |  |
| At least 1x/week | 0.34 | 0.30 | 0.26 | -0.26 | 0.93 | 0.33 |
| 1-3x/month | 0.43 | 0.32 | 0.18 | -0.20 | 1.05 | . |
| <1x/month | 0.10 | 0.34 | 0.76 | -0.57 | 0.78 | . |
| Gender (Ref: Male) |  |  |  |  |  |  |
| Female | 0.04 | 0.06 | 0.49 | -0.07 | 0.15 | 0.03 |
| Other | 1.15 | 0.45 | 0.01 | 0.27 | 2.03 | . |
| Birth year/current age (Ref: 1998-2005; age 18-24) |  |  |  |  |  |  |
| 1993-1998; age 25-29 | 0.09 | 0.07 | 0.23 | -0.06 | 0.23 | 0.11 |
| 1983-1993; age 30-39 | 0.16 | 0.07 | 0.04 | 0.01 | 0.31 | . |
| 1973-1983; age 40-49 | 0.08 | 0.10 | 0.40 | -0.11 | 0.28 | . |
| 1963-1973; age 50-59 | 0.15 | 0.11 | 0.18 | -0.07 | 0.37 | . |
| 1953-1963; age 60-69 | 0.36 | 0.13 | 0.01 | 0.10 | 0.61 | . |
| 1943-1953; age 70-79 | 0.36 | 0.21 | 0.08 | -0.05 | 0.77 | . |
| 1943 or earlier; age 80 or older | 0.42 | 0.60 | 0.48 | -0.76 | 1.61 | . |
| Mother absence/presence (Ref: Present) |  |  |  |  |  |  |
| Absent | 0.24 | 0.17 | 0.17 | -0.10 | 0.57 | 0.17 |
| Father absence/presence (Ref: Present) |  |  |  |  |  |  |
| Absent | -0.05 | 0.15 | 0.73 | -0.34 | 0.24 | 0.73 |
| Childhood religion (Ref: Christianity) |  |  |  |  |  |  |
| Islam | -0.19 | 0.13 | 0.15 | -0.45 | 0.07 | 0.22 |
| Some other religion | -0.47 | 0.53 | 0.38 | -1.50 | 0.57 | . |
| Race/ethnicity (Ref: Ethnic plurality) |  |  |  |  |  |  |
| Ethnic minority | 0.15 | 0.08 | 0.07 | -0.01 | 0.32 | 0.07 |
| *Note*. *N* = 11,389. SE, standard error; LCI, lower confidence interval; UCI, upper confidence interval. | | | | | | |

*Table S11d. E-Values and E-Value Limits for the Coefficients Shown in Table S11c (Kenya)*

| Characteristic | *E*-value for estimate^a^ | *E*-value for 95% CI^b^ |
| --- | --- | --- |
| Relationship with mother (Ref: Very/somewhat bad) |  |  |
| Very/somewhat good | 1.28 | 1.00 |
| Relationship with father (Ref: Very/somewhat bad) |  |  |
| Very/somewhat good | 1.08 | 1.00 |
| Parent marital status (Ref: Married) |  |  |
| Divorced | 1.24 | 1.00 |
| Never married | 1.32 | 1.00 |
| One or both parents had died | 1.10 | 1.00 |
| Subjective financial status growing up (Ref: Got by) |  |  |
| Lived comfortably | 1.26 | 1.00 |
| Found it difficult | 1.11 | 1.00 |
| Found it very difficult | 1.28 | 1.00 |
| Childhood abuse (Ref: No) |  |  |
| Yes | 1.51 | 1.33 |
| Outsider growing up (Ref: No) |  |  |
| Yes | 1.17 | 1.00 |
| Childhood health (Ref: Good) |  |  |
| Excellent | 1.15 | 1.00 |
| Very good | 1.25 | 1.00 |
| Fair | 1.15 | 1.00 |
| Poor | 1.13 | 1.00 |
| Immigration status (Ref: Born in this country) |  |  |
| Born in another country | 1.50 | 1.00 |
| Childhood religious service attendance (Ref: Never) |  |  |
| At least 1x/week | 1.46 | 1.00 |
| 1-3x/month | 1.54 | 1.00 |
| <1x/month | 1.22 | 1.00 |
| Gender (Ref: Male) |  |  |
| Female | 1.12 | 1.00 |
| Other | 2.21 | 1.40 |
| Birth year/current age (Ref: 1998-2005; age 18-24) |  |  |
| 1993-1998; age 25-29 | 1.20 | 1.00 |
| 1983-1993; age 30-39 | 1.28 | 1.06 |
| 1973-1983; age 40-49 | 1.19 | 1.00 |
| 1963-1973; age 50-59 | 1.27 | 1.00 |
| 1953-1963; age 60-69 | 1.48 | 1.21 |
| 1943-1953; age 70-79 | 1.48 | 1.00 |
| 1943 or earlier; age 80 or older | 1.54 | 1.00 |
| Mother absence/presence (Ref: Present) |  |  |
| Absent | 1.36 | 1.00 |
| Father absence/presence (Ref: Present) |  |  |
| Absent | 1.14 | 1.00 |
| Childhood religion (Ref: Christianity) |  |  |
| Islam | 1.32 | 1.00 |
| Some other religion | 1.58 | 1.00 |
| Race/ethnicity (Ref: Ethnic plurality) |  |  |
| Ethnic minority | 1.27 | 1.00 |

*Note*. CI, confidence interval. ^a^The formula for calculating *E*-values can be found in VanderWeele and Ding (2017) The *E*-value for the effect estimate is the minimum strength of association (on the risk ratio scale) that an unmeasured confounder would need to have with both the predictor and the outcome to entirely explain away the observed association between them, conditional on the measured covariates. ^b^The *E*-value for the limit of the 95% confidence interval closest to the null denote the minimum strength of association (on the risk ratio scale) that an unmeasured confounder would need to have with both the predictor and the outcome to shift the confidence interval to include the null value, conditional on the measured covariates.

*Table S12a. Nationally Representative Descriptive Statistics of the Observed Sample (Mexico)*

| Variable | Proportion | Frequency |
| --- | --- | --- |
| Sociodemographic characteristics |  |  |
| Birth year/current age |  |  |
| 1998-2005; age 18-24 | 0.17 | 986 |
| 1993-1998; age 25-29 | 0.11 | 623 |
| 1983-1993; age 30-39 | 0.23 | 1312 |
| 1973-1983; age 40-49 | 0.18 | 1027 |
| 1963-1973; age 50-59 | 0.15 | 873 |
| 1953-1963; age 60-69 | 0.11 | 611 |
| 1943-1953; age 70-79 | 0.05 | 277 |
| 1943 or earlier; 80 or older | 0.01 | 68 |
| Missing | . | . |
| Gender |  |  |
| Male | 0.48 | 2755 |
| Female | 0.52 | 2997 |
| Other | 0.00 | 3 |
| Missing | 0.00 | 21 |
| Marital status |  |  |
| Single, never married | 0.25 | 1432 |
| Married | 0.36 | 2089 |
| Separated | 0.07 | 403 |
| Divorced | 0.04 | 230 |
| Widowed | 0.06 | 347 |
| Domestic partner | 0.19 | 1109 |
| Missing | 0.03 | 166 |
| Employment |  |  |
| Employed for an employer | 0.33 | 1921 |
| Self-employed | 0.19 | 1091 |
| Retired | 0.07 | 386 |
| Student | 0.04 | 247 |
| Homemaker | 0.22 | 1257 |
| Unemployed and looking for a job | 0.10 | 564 |
| None of these/other | 0.03 | 169 |
| Missing | 0.02 | 141 |
| Education |  |  |
| Up to 8 years | 0.22 | 1291 |
| 9-15 years | 0.55 | 3180 |
| 16+ years | 0.23 | 1304 |
| Missing | 0.00 | 1 |
| Religious service attendance |  |  |
| >1x/week | 0.11 | 609 |
| 1x/week | 0.22 | 1261 |
| 1-3x/month | 0.12 | 676 |
| A few times a year | 0.36 | 2054 |
| Never | 0.20 | 1134 |
| Missing | 0.01 | 43 |
| Immigration status |  |  |
| Born in this country | 0.96 | 5517 |
| Born in another country | 0.02 | 108 |
| Missing | 0.03 | 151 |
| Religion |  |  |
| Christianity | 0.84 | 4844 |
| Islam | 0.00 | 2 |
| Hinduism | 0.00 | 3 |
| Buddhism | 0.00 | 6 |
| Judaism | 0.00 | 7 |
| Sikhism | . | . |
| Baha'i | 0.00 | 1 |
| Jainism | 0.00 | 1 |
| Shinto | 0.00 | 2 |
| Taoism | 0.00 | 4 |
| Confucianism | 0.00 | 1 |
| Primal, Animist, or Folk Religion | 0.00 | 20 |
| Spiritism | . | . |
| African-derived | . | . |
| Chinese | . | . |
| Some other religion | 0.01 | 41 |
| No religion/atheist/agnostic | 0.13 | 770 |
| Missing | 0.01 | 75 |
| Race/ethnicity |  |  |
| White | 0.19 | 1116 |
| Mestizo | 0.48 | 2762 |
| Indigenous | 0.10 | 594 |
| Black | 0.02 | 108 |
| Mulatto | 0.01 | 63 |
| Other | 0.06 | 339 |
| Missing | 0.14 | 794 |
| Childhood factors |  |  |
| Relationship with mother |  |  |
| Very good | 0.68 | 3912 |
| Somewhat good | 0.23 | 1340 |
| Somewhat bad | 0.03 | 177 |
| Very bad | 0.02 | 90 |
| Not applicable | 0.03 | 177 |
| Missing | 0.01 | 80 |
| Relationship with father |  |  |
| Very good | 0.53 | 3089 |
| Somewhat good | 0.27 | 1556 |
| Somewhat bad | 0.06 | 335 |
| Very bad | 0.05 | 267 |
| Not applicable | 0.08 | 470 |
| Missing | 0.01 | 60 |
| Parent marital status |  |  |
| Married | 0.69 | 3999 |
| Divorced | 0.06 | 341 |
| Never married | 0.14 | 827 |
| One or both parents had died | 0.03 | 176 |
| Missing | 0.07 | 432 |
| Subjective financial status growing up |  |  |
| Lived comfortably | 0.31 | 1775 |
| Got by | 0.32 | 1872 |
| Found it difficult | 0.30 | 1712 |
| Found it very difficult | 0.06 | 369 |
| Missing | 0.01 | 48 |
| Childhood abuse |  |  |
| Yes | 0.16 | 905 |
| No | 0.80 | 4604 |
| Missing | 0.05 | 267 |
| Outsider growing up |  |  |
| Yes | 0.13 | 772 |
| No | 0.85 | 4897 |
| Not applicable | 0.01 | 58 |
| Missing | 0.01 | 49 |
| Childhood health |  |  |
| Excellent | 0.32 | 1860 |
| Very good | 0.23 | 1350 |
| Good | 0.29 | 1677 |
| Fair | 0.13 | 743 |
| Poor | 0.02 | 133 |
| Missing | 0.00 | 14 |
| Childhood religious service attendance |  |  |
| At least 1x/week | 0.44 | 2514 |
| 1-3x/month | 0.20 | 1162 |
| <1x/month | 0.19 | 1087 |
| Never | 0.16 | 944 |
| Missing | 0.01 | 69 |
| Childhood religion |  |  |
| Christianity | 0.92 | 5337 |
| Islam | 0.00 | 6 |
| Hinduism | 0.00 | 1 |
| Buddhism | 0.00 | 1 |
| Judaism | 0.00 | 8 |
| Sikhism | 0.00 | 4 |
| Baha'i | 0.00 | 1 |
| Jainism | . | . |
| Shinto | 0.00 | 2 |
| Taoism | 0.00 | 5 |
| Confucianism | . | . |
| Primal, Animist, or Folk Religion | 0.00 | 2 |
| Spiritism | . | . |
| African-derived | . | . |
| Chinese | . | . |
| Some other religion | 0.00 | 7 |
| No religion/atheist/agnostic | 0.06 | 328 |
| Missing | 0.01 | 74 |

*Note*. *N* = 5,776.

*Table S12b. Variations Across Sociodemographic Characteristics (Mexico)*

| Characteristic | Mean | SE | LCI | UCI | Global *p*-value |
| --- | --- | --- | --- | --- | --- |
| Age group |  |  |  |  |  |
| 18-24 | 8.34 | 0.08 | 8.18 | 8.49 | 0.00 |
| 25-29 | 8.62 | 0.09 | 8.45 | 8.80 | . |
| 30-39 | 8.75 | 0.07 | 8.62 | 8.87 | . |
| 40-49 | 8.86 | 0.07 | 8.73 | 8.99 | . |
| 50-59 | 8.97 | 0.07 | 8.83 | 9.11 | . |
| 60-69 | 8.62 | 0.12 | 8.38 | 8.86 | . |
| 70-79 | 8.91 | 0.12 | 8.67 | 9.16 | . |
| 80 or older | 8.63 | 0.25 | 8.13 | 9.13 | . |
| Gender |  |  |  |  |  |
| Male | 8.62 | 0.05 | 8.53 | 8.71 | 0.01 |
| Female | 8.80 | 0.04 | 8.71 | 8.88 | . |
| Other | 7.18 | 1.09 | 4.32 | 10.03 | . |
| Marital status |  |  |  |  |  |
| Single, never married | 8.34 | 0.06 | 8.22 | 8.47 | 0.00 |
| Married | 8.88 | 0.05 | 8.78 | 8.98 | . |
| Separated | 8.65 | 0.12 | 8.42 | 8.88 | . |
| Divorced | 8.84 | 0.16 | 8.53 | 9.14 | . |
| Widowed | 8.86 | 0.13 | 8.61 | 9.11 | . |
| Domestic partner | 8.81 | 0.07 | 8.68 | 8.94 | . |
| Employment |  |  |  |  |  |
| Employed for an employer | 8.68 | 0.06 | 8.57 | 8.79 | 0.00 |
| Self-employed | 8.77 | 0.07 | 8.64 | 8.90 | . |
| Retired | 8.76 | 0.12 | 8.53 | 8.99 | . |
| Student | 8.19 | 0.14 | 7.91 | 8.47 | . |
| Homemaker | 8.89 | 0.07 | 8.76 | 9.02 | . |
| Unemployed and looking for a job | 8.45 | 0.10 | 8.26 | 8.64 | . |
| None of these/other | 8.81 | 0.17 | 8.47 | 9.15 | . |
| Education |  |  |  |  |  |
| Up to 8 years | 8.61 | 0.08 | 8.46 | 8.76 | 0.22 |
| 9-15 years | 8.76 | 0.04 | 8.68 | 8.84 | . |
| 16+ years | 8.70 | 0.06 | 8.59 | 8.81 | . |
| Religious service attendance |  |  |  |  |  |
| >1x/week | 9.00 | 0.08 | 8.84 | 9.17 | 0.00 |
| 1x/week | 8.84 | 0.06 | 8.71 | 8.96 | . |
| 1-3x/month | 8.61 | 0.10 | 8.42 | 8.80 | . |
| A few times a year | 8.77 | 0.05 | 8.67 | 8.87 | . |
| Never | 8.37 | 0.08 | 8.22 | 8.52 | . |
| Immigration status |  |  |  |  |  |
| Born in this country | 8.72 | 0.03 | 8.66 | 8.78 | 0.10 |
| Born in another country | 8.29 | 0.25 | 7.80 | 8.79 | . |
| Religion |  |  |  |  |  |
| Christianity | 8.78 | 0.03 | 8.71 | 8.84 | 0.00 |
| Islam | 9.76 | . | . | . | . |
| Hinduism | 8.76 | 1.25 | 3.38 | 14.15 | . |
| Buddhism | 8.13 | 0.17 | 7.63 | 8.62 | . |
| Judaism | 9.10 | 0.59 | 7.56 | 10.64 | . |
| Sikhism | . | . | . | . | . |
| Baha'i | 9.09 | . | . | . | . |
| Jainism | 7.00 | . | . | . | . |
| Shinto | 9.67 | 0.44 | -63.45 | 82.78 | . |
| Taoism | 7.00 | . | . | . | . |
| Confucianism | 7.00 | . | . | . | . |
| Primal, Animist, or Folk Religion | 8.70 | 0.37 | 7.88 | 9.53 | . |
| Spiritism | . | . | . | . | . |
| African-derived | . | . | . | . | . |
| Chinese | . | . | . | . | . |
| Some other religion | 9.14 | 0.20 | 8.74 | 9.53 | . |
| No religion/atheist/agnostic | 8.28 | 0.08 | 8.12 | 8.45 | . |
| Race/ethnicity |  |  |  |  |  |
| White | 8.77 | 0.07 | 8.62 | 8.92 | 0.92 |
| Mestizo | 8.71 | 0.04 | 8.63 | 8.78 | . |
| Indigenous | 8.63 | 0.11 | 8.42 | 8.84 | . |
| Black | 8.59 | 0.27 | 8.05 | 9.13 | . |
| Mulatto | 8.77 | 0.32 | 8.14 | 9.41 | . |
| Other | 8.73 | 0.13 | 8.47 | 8.99 | . |

*Note*. *N* = 5,776. SE, standard error; LCI, lower confidence interval; UCI, upper confidence interval.

*Table S12c. Variations Across Childhood Predictors (Mexico)*

| Characteristic | Coef. | SE | Prob. | LCI | UCI | Global *p*-value |
| --- | --- | --- | --- | --- | --- | --- |
| Relationship with mother (Ref: Very/somewhat bad) |  |  |  |  |  |  |
| Very/somewhat good | 0.41 | 0.17 | 0.02 | 0.08 | 0.74 | 0.02 |
| Relationship with father (Ref: Very/somewhat bad) |  |  |  |  |  |  |
| Very/somewhat good | -0.03 | 0.11 | 0.79 | -0.25 | 0.19 | 0.79 |
| Parent marital status (Ref: Married) |  |  |  |  |  |  |
| Divorced | 0.08 | 0.14 | 0.55 | -0.19 | 0.35 | 0.92 |
| Never married | 0.05 | 0.09 | 0.61 | -0.13 | 0.23 | . |
| One or both parents had died | 0.05 | 0.19 | 0.80 | -0.34 | 0.43 | . |
| Subjective financial status growing up (Ref: Got by) |  |  |  |  |  |  |
| Lived comfortably | 0.21 | 0.07 | 0.00 | 0.07 | 0.35 | 0.01 |
| Found it difficult | 0.05 | 0.08 | 0.51 | -0.10 | 0.21 | . |
| Found it very difficult | -0.19 | 0.19 | 0.33 | -0.56 | 0.19 | . |
| Childhood abuse (Ref: No) |  |  |  |  |  |  |
| Yes | -0.22 | 0.10 | 0.02 | -0.41 | -0.03 | 0.02 |
| Outsider growing up (Ref: No) |  |  |  |  |  |  |
| Yes | -0.21 | 0.11 | 0.06 | -0.44 | 0.01 | 0.06 |
| Childhood health (Ref: Good) |  |  |  |  |  |  |
| Excellent | 0.15 | 0.07 | 0.05 | 0.00 | 0.29 | 0.00 |
| Very good | 0.03 | 0.07 | 0.62 | -0.10 | 0.17 | . |
| Fair | -0.51 | 0.13 | 0.00 | -0.76 | -0.25 | . |
| Poor | -0.25 | 0.23 | 0.27 | -0.70 | 0.19 | . |
| Immigration status (Ref: Born in this country) |  |  |  |  |  |  |
| Born in another country | -0.29 | 0.25 | 0.23 | -0.78 | 0.19 | 0.23 |
| Childhood religious service attendance (Ref: Never) |  |  |  |  |  |  |
| At least 1x/week | 0.33 | 0.11 | 0.00 | 0.12 | 0.55 | 0.00 |
| 1-3x/month | 0.06 | 0.12 | 0.60 | -0.18 | 0.30 | . |
| <1x/month | 0.09 | 0.12 | 0.43 | -0.14 | 0.33 | . |
| Gender (Ref: Male) |  |  |  |  |  |  |
| Female | 0.24 | 0.06 | 0.00 | 0.13 | 0.36 | 0.00 |
| Other | -1.02 | 1.08 | 0.34 | -3.14 | 1.09 | . |
| Birth year/current age (Ref: 1998-2005; age 18-24) |  |  |  |  |  |  |
| 1993-1998; age 25-29 | 0.29 | 0.12 | 0.02 | 0.05 | 0.52 | 0.00 |
| 1983-1993; age 30-39 | 0.41 | 0.10 | 0.00 | 0.21 | 0.61 | . |
| 1973-1983; age 40-49 | 0.51 | 0.10 | 0.00 | 0.31 | 0.71 | . |
| 1963-1973; age 50-59 | 0.65 | 0.10 | 0.00 | 0.45 | 0.85 | . |
| 1953-1963; age 60-69 | 0.30 | 0.15 | 0.04 | 0.01 | 0.58 | . |
| 1943-1953; age 70-79 | 0.58 | 0.15 | 0.00 | 0.29 | 0.87 | . |
| 1943 or earlier; age 80 or older | 0.24 | 0.31 | 0.44 | -0.37 | 0.84 | . |
| Mother absence/presence (Ref: Present) |  |  |  |  |  |  |
| Absent | -0.11 | 0.18 | 0.52 | -0.46 | 0.23 | 0.52 |
| Father absence/presence (Ref: Present) |  |  |  |  |  |  |
| Absent | 0.10 | 0.12 | 0.40 | -0.13 | 0.34 | 0.40 |
| Childhood religion (Ref: No religion/atheist/agnostic) |  |  |  |  |  |  |
| Christianity | 0.17 | 0.14 | 0.22 | -0.10 | 0.44 | 0.39 |
| Some other religion | 0.31 | 0.30 | 0.30 | -0.27 | 0.89 | . |
| Race/ethnicity (Ref: Ethnic plurality) |  |  |  |  |  |  |
| Ethnic minority | 0.02 | 0.07 | 0.78 | -0.11 | 0.15 | 0.78 |
| *Note*. *N* = 5,776. SE, standard error; LCI, lower confidence interval; UCI, upper confidence interval. | | | | | | |

*Table S12d. E-Values and E-Value Limits for the Coefficients Shown in Table S12c (Mexico)*

| Characteristic | *E*-value for estimate^a^ | *E*-value for 95% CI^b^ |
| --- | --- | --- |
| Relationship with mother (Ref: Very/somewhat bad) |  |  |
| Very/somewhat good | 1.52 | 1.18 |
| Relationship with father (Ref: Very/somewhat bad) |  |  |
| Very/somewhat good | 1.11 | 1.00 |
| Parent marital status (Ref: Married) |  |  |
| Divorced | 1.19 | 1.00 |
| Never married | 1.13 | 1.00 |
| One or both parents had died | 1.14 | 1.00 |
| Subjective financial status growing up (Ref: Got by) |  |  |
| Lived comfortably | 1.34 | 1.18 |
| Found it difficult | 1.14 | 1.00 |
| Found it very difficult | 1.31 | 1.00 |
| Childhood abuse (Ref: No) |  |  |
| Yes | 1.34 | 1.11 |
| Outsider growing up (Ref: No) |  |  |
| Yes | 1.34 | 1.00 |
| Childhood health (Ref: Good) |  |  |
| Excellent | 1.27 | 1.03 |
| Very good | 1.11 | 1.00 |
| Fair | 1.61 | 1.38 |
| Poor | 1.38 | 1.00 |
| Immigration status (Ref: Born in this country) |  |  |
| Born in another country | 1.42 | 1.00 |
| Childhood religious service attendance (Ref: Never) |  |  |
| At least 1x/week | 1.46 | 1.24 |
| 1-3x/month | 1.16 | 1.00 |
| <1x/month | 1.20 | 1.00 |
| Gender (Ref: Male) |  |  |
| Female | 1.37 | 1.25 |
| Other | 2.08 | 1.00 |
| Birth year/current age (Ref: 1998-2005; age 18-24) |  |  |
| 1993-1998; age 25-29 | 1.41 | 1.15 |
| 1983-1993; age 30-39 | 1.52 | 1.33 |
| 1973-1983; age 40-49 | 1.62 | 1.43 |
| 1963-1973; age 50-59 | 1.74 | 1.56 |
| 1953-1963; age 60-69 | 1.42 | 1.06 |
| 1943-1953; age 70-79 | 1.68 | 1.41 |
| 1943 or earlier; age 80 or older | 1.36 | 1.00 |
| Mother absence/presence (Ref: Present) |  |  |
| Absent | 1.23 | 1.00 |
| Father absence/presence (Ref: Present) |  |  |
| Absent | 1.21 | 1.00 |
| Childhood religion (Ref: No religion/atheist/agnostic) |  |  |
| Christianity | 1.29 | 1.00 |
| Some other religion | 1.43 | 1.00 |
| Race/ethnicity (Ref: Ethnic plurality) |  |  |
| Ethnic minority | 1.08 | 1.00 |

*Note*. CI, confidence interval. ^a^The formula for calculating *E*-values can be found in VanderWeele and Ding (2017) The *E*-value for the effect estimate is the minimum strength of association (on the risk ratio scale) that an unmeasured confounder would need to have with both the predictor and the outcome to entirely explain away the observed association between them, conditional on the measured covariates. ^b^The *E*-value for the limit of the 95% confidence interval closest to the null denote the minimum strength of association (on the risk ratio scale) that an unmeasured confounder would need to have with both the predictor and the outcome to shift the confidence interval to include the null value, conditional on the measured covariates.

*Table S13a. Nationally Representative Descriptive Statistics of the Observed Sample (Nigeria)*

| Variable | Proportion | Frequency |
| --- | --- | --- |
| Sociodemographic characteristics |  |  |
| Birth year/current age |  |  |
| 1998-2005; age 18-24 | 0.22 | 1533 |
| 1993-1998; age 25-29 | 0.17 | 1193 |
| 1983-1993; age 30-39 | 0.28 | 1943 |
| 1973-1983; age 40-49 | 0.16 | 1059 |
| 1963-1973; age 50-59 | 0.09 | 619 |
| 1953-1963; age 60-69 | 0.04 | 296 |
| 1943-1953; age 70-79 | 0.02 | 133 |
| 1943 or earlier; 80 or older | 0.01 | 50 |
| Missing | . | . |
| Gender |  |  |
| Male | 0.49 | 3371 |
| Female | 0.51 | 3456 |
| Other | 0.00 | 0 |
| Missing | . | . |
| Marital status |  |  |
| Single, never married | 0.34 | 2289 |
| Married | 0.60 | 4065 |
| Separated | 0.02 | 117 |
| Divorced | 0.01 | 71 |
| Widowed | 0.03 | 231 |
| Domestic partner | 0.00 | 12 |
| Missing | 0.01 | 42 |
| Employment |  |  |
| Employed for an employer | 0.10 | 699 |
| Self-employed | 0.57 | 3898 |
| Retired | 0.03 | 178 |
| Student | 0.10 | 650 |
| Homemaker | 0.07 | 499 |
| Unemployed and looking for a job | 0.10 | 684 |
| None of these/other | 0.03 | 211 |
| Missing | 0.00 | 8 |
| Education |  |  |
| Up to 8 years | 0.38 | 2575 |
| 9-15 years | 0.60 | 4120 |
| 16+ years | 0.02 | 130 |
| Missing | 0.00 | 2 |
| Religious service attendance |  |  |
| >1x/week | 0.59 | 4049 |
| 1x/week | 0.28 | 1895 |
| 1-3x/month | 0.08 | 531 |
| A few times a year | 0.04 | 254 |
| Never | 0.01 | 77 |
| Missing | 0.00 | 20 |
| Immigration status |  |  |
| Born in this country | 0.99 | 6779 |
| Born in another country | 0.01 | 47 |
| Missing | 0.00 | 1 |
| Religion |  |  |
| Christianity | 0.51 | 3476 |
| Islam | 0.48 | 3302 |
| Hinduism | . | . |
| Buddhism | . | . |
| Judaism | . | . |
| Sikhism | . | . |
| Baha'i | . | . |
| Jainism | . | . |
| Shinto | 0.00 | 1 |
| Taoism | . | . |
| Confucianism | 0.00 | 0 |
| Primal, Animist, or Folk Religion | 0.00 | 24 |
| Spiritism | . | . |
| African-derived | . | . |
| Chinese | . | . |
| Some other religion | 0.00 | 1 |
| No religion/atheist/agnostic | 0.00 | 15 |
| Missing | 0.00 | 9 |
| Race/ethnicity |  |  |
| Hausa | 0.34 | 2342 |
| Yoruba | 0.18 | 1230 |
| Igbo (Ibo) | 0.16 | 1112 |
| Edo | 0.02 | 116 |
| Urhobo | 0.01 | 38 |
| Fulani | 0.04 | 266 |
| Kanuri | 0.00 | 31 |
| Tiv | 0.03 | 198 |
| Efik | 0.01 | 48 |
| Ijaw | 0.02 | 110 |
| Igala | 0.01 | 77 |
| Ibibio | 0.03 | 180 |
| Idoma | 0.01 | 61 |
| Other | 0.15 | 1014 |
| Missing | 0.00 | 4 |
| Childhood factors |  |  |
| Relationship with mother |  |  |
| Very good | 0.88 | 5986 |
| Somewhat good | 0.09 | 648 |
| Somewhat bad | 0.01 | 62 |
| Very bad | 0.00 | 18 |
| Not applicable | 0.02 | 104 |
| Missing | 0.00 | 9 |
| Relationship with father |  |  |
| Very good | 0.82 | 5578 |
| Somewhat good | 0.14 | 924 |
| Somewhat bad | 0.01 | 76 |
| Very bad | 0.01 | 43 |
| Not applicable | 0.03 | 177 |
| Missing | 0.00 | 29 |
| Parent marital status |  |  |
| Married | 0.82 | 5568 |
| Divorced | 0.05 | 307 |
| Never married | 0.05 | 335 |
| One or both parents had died | 0.07 | 462 |
| Missing | 0.02 | 154 |
| Subjective financial status growing up |  |  |
| Lived comfortably | 0.32 | 2192 |
| Got by | 0.35 | 2381 |
| Found it difficult | 0.24 | 1661 |
| Found it very difficult | 0.08 | 563 |
| Missing | 0.00 | 29 |
| Childhood abuse |  |  |
| Yes | 0.13 | 880 |
| No | 0.86 | 5851 |
| Missing | 0.01 | 96 |
| Outsider growing up |  |  |
| Yes | 0.10 | 669 |
| No | 0.89 | 6059 |
| Not applicable | 0.01 | 86 |
| Missing | 0.00 | 13 |
| Childhood health |  |  |
| Excellent | 0.39 | 2644 |
| Very good | 0.38 | 2613 |
| Good | 0.17 | 1152 |
| Fair | 0.04 | 306 |
| Poor | 0.01 | 98 |
| Missing | 0.00 | 14 |
| Childhood religious service attendance |  |  |
| At least 1x/week | 0.87 | 5907 |
| 1-3x/month | 0.09 | 600 |
| <1x/month | 0.02 | 136 |
| Never | 0.02 | 138 |
| Missing | 0.01 | 45 |
| Childhood religion |  |  |
| Christianity | 0.51 | 3463 |
| Islam | 0.49 | 3314 |
| Hinduism | . | . |
| Buddhism | 0.00 | 0 |
| Judaism | . | . |
| Sikhism | . | . |
| Baha'i | . | . |
| Jainism | . | . |
| Shinto | . | . |
| Taoism | . | . |
| Confucianism | 0.00 | 0 |
| Primal, Animist, or Folk Religion | 0.00 | 17 |
| Spiritism | . | . |
| African-derived | . | . |
| Chinese | . | . |
| Some other religion | . | . |
| No religion/atheist/agnostic | 0.00 | 19 |
| Missing | 0.00 | 14 |

*Note. N* = 6,827.

*Table S13b. Variations Across Sociodemographic Characteristics (Nigeria)*

| Characteristic | Mean | SE | LCI | UCI | Global *p*-value |
| --- | --- | --- | --- | --- | --- |
| Age group |  |  |  |  |  |
| 18-24 | 8.29 | 0.07 | 8.16 | 8.42 | 0.04 |
| 25-29 | 8.25 | 0.07 | 8.11 | 8.39 | . |
| 30-39 | 8.42 | 0.05 | 8.32 | 8.53 | . |
| 40-49 | 8.33 | 0.09 | 8.15 | 8.51 | . |
| 50-59 | 8.58 | 0.14 | 8.30 | 8.87 | . |
| 60-69 | 8.87 | 0.18 | 8.50 | 9.24 | . |
| 70-79 | 8.34 | 0.39 | 7.49 | 9.19 | . |
| 80 or older | 7.72 | 0.67 | 5.54 | 9.90 | . |
| Gender |  |  |  |  |  |
| Male | 8.29 | 0.05 | 8.19 | 8.39 | 0.00 |
| Female | 8.46 | 0.05 | 8.37 | 8.56 | . |
| Other | 8.00 | . | . | . | . |
| Marital status |  |  |  |  |  |
| Single, never married | 8.37 | 0.05 | 8.26 | 8.47 | 0.13 |
| Married | 8.36 | 0.05 | 8.27 | 8.46 | . |
| Separated | 7.99 | 0.30 | 7.40 | 8.59 | . |
| Divorced | 8.55 | 0.31 | 7.91 | 9.19 | . |
| Widowed | 8.89 | 0.20 | 8.48 | 9.29 | . |
| Domestic partner | 8.67 | 0.20 | 6.81 | 10.53 | . |
| Employment |  |  |  |  |  |
| Employed for an employer | 8.41 | 0.08 | 8.25 | 8.58 | 0.37 |
| Self-employed | 8.35 | 0.05 | 8.26 | 8.44 | . |
| Retired | 8.60 | 0.31 | 7.97 | 9.24 | . |
| Student | 8.26 | 0.08 | 8.10 | 8.42 | . |
| Homemaker | 8.40 | 0.13 | 8.14 | 8.67 | . |
| Unemployed and looking for a job | 8.54 | 0.09 | 8.36 | 8.73 | . |
| None of these/other | 8.29 | 0.18 | 7.92 | 8.66 | . |
| Education |  |  |  |  |  |
| Up to 8 years | 8.38 | 0.08 | 8.23 | 8.53 | 0.99 |
| 9-15 years | 8.38 | 0.03 | 8.31 | 8.44 | . |
| 16+ years | 8.36 | 0.15 | 8.07 | 8.65 | . |
| Religious service attendance |  |  |  |  |  |
| >1x/week | 8.54 | 0.04 | 8.45 | 8.62 | 0.00 |
| 1x/week | 8.28 | 0.06 | 8.16 | 8.41 | . |
| 1-3x/month | 7.67 | 0.13 | 7.41 | 7.93 | . |
| A few times a year | 8.10 | 0.22 | 7.66 | 8.54 | . |
| Never | 7.99 | 0.36 | 7.27 | 8.71 | . |
| Immigration status |  |  |  |  |  |
| Born in this country | 8.38 | 0.04 | 8.31 | 8.45 | 0.71 |
| Born in another country | 8.24 | 0.34 | 7.51 | 8.98 | . |
| Religion |  |  |  |  |  |
| Christianity | 8.48 | 0.05 | 8.39 | 8.57 | 0.00 |
| Islam | 8.28 | 0.05 | 8.17 | 8.38 | . |
| Hinduism | . | . | . | . | . |
| Buddhism | . | . | . | . | . |
| Judaism | . | . | . | . | . |
| Sikhism | . | . | . | . | . |
| Baha'i | . | . | . | . | . |
| Jainism | . | . | . | . | . |
| Shinto | 8.00 | . | . | . | . |
| Taoism | . | . | . | . | . |
| Confucianism | 9.00 | . | . | . | . |
| Primal, Animist, or Folk Religion | 7.75 | 0.30 | 6.96 | 8.55 | . |
| Spiritism | . | . | . | . | . |
| African-derived | . | . | . | . | . |
| Chinese | . | . | . | . | . |
| Some other religion | 7.00 | . | . | . | . |
| No religion/atheist/agnostic | 8.65 | 0.31 | 6.00 | 11.29 | . |
| Race/ethnicity |  |  |  |  |  |
| Hausa | 8.23 | 0.07 | 8.08 | 8.37 | 0.02 |
| Yoruba | 8.42 | 0.08 | 8.26 | 8.59 | . |
| Igbo (Ibo) | 8.45 | 0.07 | 8.32 | 8.58 | . |
| Edo | 8.75 | 0.23 | 8.27 | 9.22 | . |
| Urhobo | 8.55 | 0.34 | 7.82 | 9.27 | . |
| Fulani | 8.59 | 0.15 | 8.28 | 8.90 | . |
| Kanuri | 8.23 | 0.14 | 7.90 | 8.57 | . |
| Tiv | 8.60 | 0.17 | 8.25 | 8.96 | . |
| Efik | 8.76 | 0.26 | 8.20 | 9.32 | . |
| Ijaw | 8.81 | 0.20 | 8.35 | 9.27 | . |
| Igala | 8.08 | 0.38 | 7.29 | 8.86 | . |
| Ibibio | 8.71 | 0.15 | 8.38 | 9.04 | . |
| Idoma | 8.76 | 0.21 | 8.33 | 9.20 | . |
| Other | 8.32 | 0.09 | 8.14 | 8.49 | . |

*Note*. *N* = 6,827. SE, standard error; LCI, lower confidence interval; UCI, upper confidence interval.

*Table S13c. Variations Across Childhood Predictors (Nigeria)*

| Characteristic | Coef. | SE | Prob. | LCI | UCI | Global *p*-value |
| --- | --- | --- | --- | --- | --- | --- |
| Relationship with mother (Ref: Very/somewhat bad) |  |  |  |  |  |  |
| Very/somewhat good | -0.49 | 0.27 | 0.07 | -1.02 | 0.04 | 0.07 |
| Relationship with father (Ref: Very/somewhat bad) |  |  |  |  |  |  |
| Very/somewhat good | -0.21 | 0.20 | 0.30 | -0.61 | 0.19 | 0.30 |
| Parent marital status (Ref: Married) |  |  |  |  |  |  |
| Divorced | -0.28 | 0.17 | 0.11 | -0.62 | 0.07 | 0.00 |
| Never married | -0.45 | 0.17 | 0.01 | -0.78 | -0.12 | . |
| One or both parents had died | -0.54 | 0.16 | 0.00 | -0.87 | -0.22 | . |
| Subjective financial status growing up (Ref: Got by) |  |  |  |  |  |  |
| Lived comfortably | -0.01 | 0.09 | 0.94 | -0.18 | 0.16 | 0.49 |
| Found it difficult | -0.09 | 0.09 | 0.28 | -0.27 | 0.08 | . |
| Found it very difficult | -0.19 | 0.15 | 0.22 | -0.49 | 0.12 | . |
| Childhood abuse (Ref: No) |  |  |  |  |  |  |
| Yes | -0.11 | 0.10 | 0.28 | -0.31 | 0.09 | 0.28 |
| Outsider growing up (Ref: No) |  |  |  |  |  |  |
| Yes | 0.19 | 0.11 | 0.07 | -0.02 | 0.41 | 0.07 |
| Childhood health (Ref: Good) |  |  |  |  |  |  |
| Excellent | 0.02 | 0.11 | 0.88 | -0.20 | 0.24 | 0.39 |
| Very good | 0.00 | 0.11 | 0.97 | -0.21 | 0.22 | . |
| Fair | -0.34 | 0.21 | 0.10 | -0.75 | 0.07 | . |
| Poor | 0.14 | 0.22 | 0.52 | -0.29 | 0.57 | . |
| Immigration status (Ref: Born in this country) |  |  |  |  |  |  |
| Born in another country | -0.06 | 0.36 | 0.87 | -0.76 | 0.64 | 0.87 |
| Childhood religious service attendance (Ref: Never) |  |  |  |  |  |  |
| At least 1x/week | -0.15 | 0.23 | 0.52 | -0.60 | 0.31 | 0.31 |
| 1-3x/month | -0.19 | 0.26 | 0.46 | -0.69 | 0.31 | . |
| <1x/month | -0.41 | 0.24 | 0.09 | -0.89 | 0.07 | . |
| Gender (Ref: Male) |  |  |  |  |  |  |
| Female | 0.14 | 0.07 | 0.04 | 0.00 | 0.27 | 0.00 |
| Other | -0.45 | 0.14 | 0.00 | -0.72 | -0.19 | . |
| Birth year/current age (Ref: 1998-2005; age 18-24) |  |  |  |  |  |  |
| 1993-1998; age 25-29 | -0.02 | 0.09 | 0.83 | -0.20 | 0.16 | 0.04 |
| 1983-1993; age 30-39 | 0.13 | 0.09 | 0.13 | -0.04 | 0.30 | . |
| 1973-1983; age 40-49 | 0.05 | 0.11 | 0.67 | -0.17 | 0.27 | . |
| 1963-1973; age 50-59 | 0.32 | 0.16 | 0.05 | 0.01 | 0.63 | . |
| 1953-1963; age 60-69 | 0.56 | 0.22 | 0.01 | 0.13 | 0.99 | . |
| 1943-1953; age 70-79 | 0.05 | 0.40 | 0.90 | -0.75 | 0.84 | . |
| 1943 or earlier; age 80 or older | -0.65 | 0.52 | 0.21 | -1.66 | 0.37 | . |
| Mother absence/presence (Ref: Present) |  |  |  |  |  |  |
| Absent | -0.95 | 0.28 | 0.00 | -1.50 | -0.39 | 0.00 |
| Father absence/presence (Ref: Present) |  |  |  |  |  |  |
| Absent | -0.04 | 0.22 | 0.85 | -0.47 | 0.39 | 0.85 |
| Childhood religion (Ref: Christianity) |  |  |  |  |  |  |
| Islam | -0.09 | 0.09 | 0.33 | -0.27 | 0.09 | 0.61 |
| Some other religion | -0.01 | 0.53 | 0.99 | -1.06 | 1.04 | . |
| Race/ethnicity (Ref: Ethnic plurality) |  |  |  |  |  |  |
| Ethnic minority | 0.12 | 0.10 | 0.25 | -0.08 | 0.33 | 0.25 |
| *Note*. *N* = 6,827. SE, standard error; LCI, lower confidence interval; UCI, upper confidence interval. | | | | | | |

*Table S13d. E-Values and E-Value Limits for the Coefficients Shown in Table S13c (Nigeria)*

| Characteristic | *E*-value for estimate^a^ | *E*-value for 95% CI^b^ |
| --- | --- | --- |
| Relationship with mother (Ref: Very/somewhat bad) |  |  |
| Very/somewhat good | 1.61 | 1.00 |
| Relationship with father (Ref: Very/somewhat bad) |  |  |
| Very/somewhat good | 1.34 | 1.00 |
| Parent marital status (Ref: Married) |  |  |
| Divorced | 1.41 | 1.00 |
| Never married | 1.57 | 1.25 |
| One or both parents had died | 1.66 | 1.35 |
| Subjective financial status growing up (Ref: Got by) |  |  |
| Lived comfortably | 1.05 | 1.00 |
| Found it difficult | 1.21 | 1.00 |
| Found it very difficult | 1.31 | 1.00 |
| Childhood abuse (Ref: No) |  |  |
| Yes | 1.22 | 1.00 |
| Outsider growing up (Ref: No) |  |  |
| Yes | 1.32 | 1.00 |
| Childhood health (Ref: Good) |  |  |
| Excellent | 1.08 | 1.00 |
| Very good | 1.04 | 1.00 |
| Fair | 1.47 | 1.00 |
| Poor | 1.26 | 1.00 |
| Immigration status (Ref: Born in this country) |  |  |
| Born in another country | 1.15 | 1.00 |
| Childhood religious service attendance (Ref: Never) |  |  |
| At least 1x/week | 1.27 | 1.00 |
| 1-3x/month | 1.32 | 1.00 |
| <1x/month | 1.54 | 1.00 |
| Gender (Ref: Male) |  |  |
| Female | 1.26 | 1.04 |
| Other | 1.57 | 1.32 |
| Birth year/current age (Ref: 1998-2005; age 18-24) |  |  |
| 1993-1998; age 25-29 | 1.08 | 1.00 |
| 1983-1993; age 30-39 | 1.25 | 1.00 |
| 1973-1983; age 40-49 | 1.14 | 1.00 |
| 1963-1973; age 50-59 | 1.45 | 1.05 |
| 1953-1963; age 60-69 | 1.67 | 1.25 |
| 1943-1953; age 70-79 | 1.14 | 1.00 |
| 1943 or earlier; age 80 or older | 1.75 | 1.00 |
| Mother absence/presence (Ref: Present) |  |  |
| Absent | 2.03 | 1.52 |
| Father absence/presence (Ref: Present) |  |  |
| Absent | 1.13 | 1.00 |
| Childhood religion (Ref: Christianity) |  |  |
| Islam | 1.20 | 1.00 |
| Some other religion | 1.05 | 1.00 |
| Race/ethnicity (Ref: Ethnic plurality) |  |  |
| Ethnic minority | 1.24 | 1.00 |

*Note.* CI, confidence interval. ^a^The formula for calculating *E*-values can be found in VanderWeele and Ding (2017) The *E*-value for the effect estimate is the minimum strength of association (on the risk ratio scale) that an unmeasured confounder would need to have with both the predictor and the outcome to entirely explain away the observed association between them, conditional on the measured covariates. ^b^The *E*-value for the limit of the 95% confidence interval closest to the null denote the minimum strength of association (on the risk ratio scale) that an unmeasured confounder would need to have with both the predictor and the outcome to shift the confidence interval to include the null value, conditional on the measured covariates.

*Table S14a. Nationally Representative Descriptive Statistics of the Observed Sample (Philippines)*

| Variable | Proportion | Frequency |
| --- | --- | --- |
| Sociodemographic characteristics |  |  |
| Birth year/current age |  |  |
| 1998-2005; age 18-24 | 0.20 | 1073 |
| 1993-1998; age 25-29 | 0.13 | 695 |
| 1983-1993; age 30-39 | 0.22 | 1160 |
| 1973-1983; age 40-49 | 0.18 | 972 |
| 1963-1973; age 50-59 | 0.14 | 732 |
| 1953-1963; age 60-69 | 0.09 | 495 |
| 1943-1953; age 70-79 | 0.03 | 143 |
| 1943 or earlier; 80 or older | 0.00 | 23 |
| Missing | . | . |
| Gender |  |  |
| Male | 0.50 | 2625 |
| Female | 0.50 | 2643 |
| Other | 0.00 | 13 |
| Missing | 0.00 | 11 |
| Marital status |  |  |
| Single, never married | 0.23 | 1206 |
| Married | 0.45 | 2385 |
| Separated | 0.05 | 249 |
| Divorced | 0.00 | 9 |
| Widowed | 0.05 | 274 |
| Domestic partner | 0.22 | 1152 |
| Missing | 0.00 | 16 |
| Employment |  |  |
| Employed for an employer | 0.26 | 1350 |
| Self-employed | 0.26 | 1379 |
| Retired | 0.03 | 158 |
| Student | 0.11 | 585 |
| Homemaker | 0.20 | 1049 |
| Unemployed and looking for a job | 0.12 | 658 |
| None of these/other | 0.02 | 113 |
| Missing | . | . |
| Education |  |  |
| Up to 8 years | 0.22 | 1188 |
| 9-15 years | 0.70 | 3722 |
| 16+ years | 0.07 | 381 |
| Missing | 0.00 | 1 |
| Religious service attendance |  |  |
| >1x/week | 0.16 | 844 |
| 1x/week | 0.36 | 1929 |
| 1-3x/month | 0.26 | 1374 |
| A few times a year | 0.18 | 929 |
| Never | 0.04 | 210 |
| Missing | 0.00 | 6 |
| Immigration status |  |  |
| Born in this country | 1.00 | 5284 |
| Born in another country | 0.00 | 8 |
| Missing | . | . |
| Religion |  |  |
| Christianity | 0.93 | 4914 |
| Islam | 0.06 | 297 |
| Hinduism | . | . |
| Buddhism | 0.00 | 4 |
| Judaism | 0.00 | 4 |
| Sikhism | . | . |
| Baha'i | 0.00 | 1 |
| Jainism | . | . |
| Shinto | . | . |
| Taoism | . | . |
| Confucianism | . | . |
| Primal, Animist, or Folk Religion | 0.00 | 5 |
| Spiritism | . | . |
| African-derived | . | . |
| Chinese | . | . |
| Some other religion | 0.01 | 35 |
| No religion/atheist/agnostic | 0.00 | 23 |
| Missing | 0.00 | 9 |
| Race/ethnicity |  |  |
| Tagalog | 0.32 | 1691 |
| Cebuana | 0.12 | 656 |
| Ilocano/Ilokano | 0.08 | 429 |
| Visayan/Bisaya | 0.14 | 739 |
| Ilonggo/Hiligaynon | 0.08 | 428 |
| Bicolano/Bikolano | 0.06 | 300 |
| Waray | 0.04 | 216 |
| Tausug | 0.02 | 94 |
| Maranao | 0.01 | 39 |
| Maguindanaoan | 0.02 | 84 |
| Chinese-Filipino | 0.00 | 3 |
| Kapampangan | 0.02 | 107 |
| Pangasinese | 0.02 | 107 |
| Zamboangueno | 0.01 | 51 |
| Malay | . | . |
| Masbateno | 0.01 | 54 |
| Aeta | 0.00 | 1 |
| Igorot | 0.01 | 42 |
| Mangyan | 0.00 | 2 |
| Badjao | 0.00 | 2 |
| Other | 0.05 | 244 |
| Missing | 0.00 | 3 |
| Childhood factors |  |  |
| Relationship with mother |  |  |
| Very good | 0.63 | 3333 |
| Somewhat good | 0.32 | 1703 |
| Somewhat bad | 0.02 | 124 |
| Very bad | 0.01 | 39 |
| Not applicable | 0.01 | 59 |
| Missing | 0.01 | 35 |
| Relationship with father |  |  |
| Very good | 0.65 | 3443 |
| Somewhat good | 0.27 | 1429 |
| Somewhat bad | 0.03 | 159 |
| Very bad | 0.01 | 58 |
| Not applicable | 0.02 | 108 |
| Missing | 0.02 | 95 |
| Parent marital status |  |  |
| Married | 0.86 | 4575 |
| Divorced | 0.01 | 64 |
| Never married | 0.10 | 517 |
| One or both parents had died | 0.01 | 51 |
| Missing | 0.02 | 86 |
| Subjective financial status growing up |  |  |
| Lived comfortably | 0.18 | 937 |
| Got by | 0.57 | 3006 |
| Found it difficult | 0.20 | 1055 |
| Found it very difficult | 0.06 | 291 |
| Missing | 0.00 | 3 |
| Childhood abuse |  |  |
| Yes | 0.08 | 420 |
| No | 0.91 | 4837 |
| Missing | 0.01 | 35 |
| Outsider growing up |  |  |
| Yes | 0.07 | 395 |
| No | 0.92 | 4884 |
| Not applicable | 0.00 | 3 |
| Missing | 0.00 | 9 |
| Childhood health |  |  |
| Excellent | 0.20 | 1041 |
| Very good | 0.11 | 559 |
| Good | 0.41 | 2174 |
| Fair | 0.24 | 1246 |
| Poor | 0.05 | 272 |
| Missing | 0.00 | 0 |
| Childhood religious service attendance |  |  |
| At least 1x/week | 0.46 | 2453 |
| 1-3x/month | 0.32 | 1699 |
| <1x/month | 0.17 | 892 |
| Never | 0.04 | 201 |
| Missing | 0.01 | 47 |
| Childhood religion |  |  |
| Christianity | 0.94 | 4968 |
| Islam | 0.05 | 276 |
| Hinduism | . | . |
| Buddhism | 0.00 | 1 |
| Judaism | . | . |
| Sikhism | 0.00 | 4 |
| Baha'i | 0.00 | 1 |
| Jainism | . | . |
| Shinto | . | . |
| Taoism | . | . |
| Confucianism | . | . |
| Primal, Animist, or Folk Religion | 0.00 | 14 |
| Spiritism | . | . |
| African-derived | . | . |
| Chinese | . | . |
| Some other religion | 0.00 | 9 |
| No religion/atheist/agnostic | 0.00 | 9 |
| Missing | 0.00 | 11 |

*Note. N* = 5,292.

*Table S14b. Variations Across Sociodemographic Characteristics (Philippines)*

| Characteristic | Mean | SE | LCI | UCI | Global *p*-value |
| --- | --- | --- | --- | --- | --- |
| Age group |  |  |  |  |  |
| 18-24 | 8.93 | 0.07 | 8.79 | 9.06 | 0.01 |
| 25-29 | 9.18 | 0.07 | 9.03 | 9.33 | . |
| 30-39 | 9.16 | 0.05 | 9.06 | 9.25 | . |
| 40-49 | 9.08 | 0.06 | 8.96 | 9.20 | . |
| 50-59 | 8.94 | 0.09 | 8.78 | 9.11 | . |
| 60-69 | 9.12 | 0.10 | 8.93 | 9.31 | . |
| 70-79 | 8.48 | 0.28 | 7.92 | 9.03 | . |
| 80 or older | 8.60 | 0.19 | 7.62 | 9.58 | . |
| Gender |  |  |  |  |  |
| Male | 8.98 | 0.05 | 8.88 | 9.08 | 0.00 |
| Female | 9.12 | 0.03 | 9.05 | 9.18 | . |
| Other | 8.08 | 0.43 | 7.02 | 9.14 | . |
| Marital status |  |  |  |  |  |
| Single, never married | 8.87 | 0.07 | 8.74 | 8.99 | 0.00 |
| Married | 9.11 | 0.05 | 9.01 | 9.20 | . |
| Separated | 8.78 | 0.12 | 8.55 | 9.01 | . |
| Divorced | 8.41 | 0.40 | 4.98 | 11.85 | . |
| Widowed | 9.08 | 0.12 | 8.85 | 9.31 | . |
| Domestic partner | 9.17 | 0.06 | 9.05 | 9.28 | . |
| Employment |  |  |  |  |  |
| Employed for an employer | 9.05 | 0.06 | 8.92 | 9.17 | 0.72 |
| Self-employed | 9.06 | 0.05 | 8.96 | 9.17 | . |
| Retired | 9.12 | 0.11 | 8.91 | 9.34 | . |
| Student | 8.93 | 0.07 | 8.79 | 9.06 | . |
| Homemaker | 9.07 | 0.06 | 8.95 | 9.18 | . |
| Unemployed and looking for a job | 9.08 | 0.10 | 8.89 | 9.27 | . |
| None of these/other | 8.97 | 0.13 | 8.71 | 9.23 | . |
| Education |  |  |  |  |  |
| Up to 8 years | 9.05 | 0.08 | 8.90 | 9.19 | 0.76 |
| 9-15 years | 9.05 | 0.03 | 8.99 | 9.11 | . |
| 16+ years | 8.98 | 0.09 | 8.80 | 9.16 | . |
| Religious service attendance |  |  |  |  |  |
| >1x/week | 9.09 | 0.08 | 8.93 | 9.25 | 0.00 |
| 1x/week | 9.14 | 0.05 | 9.05 | 9.23 | . |
| 1-3x/month | 9.06 | 0.05 | 8.95 | 9.17 | . |
| A few times a year | 8.90 | 0.07 | 8.77 | 9.04 | . |
| Never | 8.57 | 0.20 | 8.16 | 8.97 | . |
| Immigration status |  |  |  |  |  |
| Born in this country | 9.05 | 0.03 | 8.99 | 9.10 | 0.72 |
| Born in another country | 8.83 | 0.34 | 7.36 | 10.29 | . |
| Religion |  |  |  |  |  |
| Christianity | 9.04 | 0.03 | 8.98 | 9.10 | 0.00 |
| Islam | 9.16 | 0.13 | 8.91 | 9.41 | . |
| Hinduism | . | . | . | . | . |
| Buddhism | 9.21 | 0.07 | -2.62 | 21.04 | . |
| Judaism | 10.00 | . | . | . | . |
| Sikhism | . | . | . | . | . |
| Baha'i | 10.00 | . | . | . | . |
| Jainism | . | . | . | . | . |
| Shinto | . | . | . | . | . |
| Taoism | . | . | . | . | . |
| Confucianism | . | . | . | . | . |
| Primal, Animist, or Folk Religion | 7.21 | 1.60 | -6.66 | 21.09 | . |
| Spiritism | . | . | . | . | . |
| African-derived | . | . | . | . | . |
| Chinese | . | . | . | . | . |
| Some other religion | 8.90 | 0.21 | 8.48 | 9.32 | . |
| No religion/atheist/agnostic | 8.82 | 0.25 | 8.21 | 9.44 | . |
| Race/ethnicity |  |  |  |  |  |
| Tagalog | 9.16 | 0.04 | 9.07 | 9.25 | 0.10 |
| Cebuana | 8.95 | 0.08 | 8.80 | 9.11 | . |
| Ilocano/Ilokano | 8.99 | 0.11 | 8.76 | 9.21 | . |
| Visayan/Bisaya | 8.96 | 0.07 | 8.83 | 9.09 | . |
| Ilonggo/Hiligaynon | 9.08 | 0.11 | 8.86 | 9.30 | . |
| Bicolano/Bikolano | 8.96 | 0.15 | 8.66 | 9.26 | . |
| Waray | 8.82 | 0.17 | 8.47 | 9.17 | . |
| Tausug | 9.29 | 0.26 | 8.65 | 9.93 | . |
| Maranao | 9.17 | 0.02 | 9.01 | 9.34 | . |
| Maguindanaoan | 9.24 | 0.30 | 8.53 | 9.95 | . |
| Chinese-Filipino | 8.97 | 0.19 | -22.74 | 40.67 | . |
| Kapampangan | 9.13 | 0.24 | 8.63 | 9.64 | . |
| Pangasinese | 8.78 | 0.51 | 7.72 | 9.83 | . |
| Zamboangueno | 9.27 | 0.20 | 8.76 | 9.78 | . |
| Malay | . | . | . | . | . |
| Masbateno | 9.21 | 0.19 | 8.66 | 9.77 | . |
| Aeta | 9.73 | . | . | . | . |
| Igorot | 8.64 | 0.39 | 7.52 | 9.76 | . |
| Mangyan | 9.32 | . | . | . | . |
| Badjao | 5.87 | 1.01 | -159.53 | 171.26 | . |
| Other | 9.01 | 0.14 | 8.73 | 9.30 | . |

*Note*. *N* = 5,292. SE, standard error; LCI, lower confidence interval; UCI, upper confidence interval.

*Table S14c. Variations Across Childhood Predictors (Philippines)*

| Characteristic | Coef. | SE | Prob. | LCI | UCI | Global *p*-value |
| --- | --- | --- | --- | --- | --- | --- |
| Relationship with mother (Ref: Very/somewhat bad) |  |  |  |  |  |  |
| Very/somewhat good | 0.06 | 0.15 | 0.69 | -0.23 | 0.35 | 0.69 |
| Relationship with father (Ref: Very/somewhat bad) |  |  |  |  |  |  |
| Very/somewhat good | -0.16 | 0.13 | 0.21 | -0.40 | 0.09 | 0.21 |
| Parent marital status (Ref: Married) |  |  |  |  |  |  |
| Divorced | -0.95 | 0.41 | 0.02 | -1.74 | -0.15 | 0.05 |
| Never married | -0.17 | 0.10 | 0.09 | -0.38 | 0.03 | . |
| One or both parents had died | -0.12 | 0.29 | 0.68 | -0.69 | 0.45 | . |
| Subjective financial status growing up (Ref: Got by) |  |  |  |  |  |  |
| Lived comfortably | 0.11 | 0.08 | 0.20 | -0.06 | 0.28 | 0.24 |
| Found it difficult | -0.04 | 0.08 | 0.56 | -0.19 | 0.11 | . |
| Found it very difficult | 0.19 | 0.15 | 0.21 | -0.11 | 0.49 | . |
| Childhood abuse (Ref: No) |  |  |  |  |  |  |
| Yes | -0.30 | 0.12 | 0.01 | -0.53 | -0.06 | 0.01 |
| Outsider growing up (Ref: No) |  |  |  |  |  |  |
| Yes | 0.02 | 0.10 | 0.87 | -0.18 | 0.22 | 0.87 |
| Childhood health (Ref: Good) |  |  |  |  |  |  |
| Excellent | 0.01 | 0.07 | 0.85 | -0.12 | 0.15 | 0.02 |
| Very good | -0.04 | 0.09 | 0.67 | -0.22 | 0.14 | . |
| Fair | -0.19 | 0.07 | 0.01 | -0.34 | -0.04 | . |
| Poor | -0.42 | 0.16 | 0.01 | -0.74 | -0.10 | . |
| Immigration status (Ref: Born in this country) |  |  |  |  |  |  |
| Born in another country | -0.29 | 0.70 | 0.68 | -1.67 | 1.10 | 0.68 |
| Childhood religious service attendance (Ref: Never) |  |  |  |  |  |  |
| At least 1x/week | 0.15 | 0.20 | 0.47 | -0.25 | 0.54 | 0.69 |
| 1-3x/month | 0.15 | 0.20 | 0.43 | -0.23 | 0.54 | . |
| <1x/month | 0.06 | 0.20 | 0.76 | -0.34 | 0.46 | . |
| Gender (Ref: Male) |  |  |  |  |  |  |
| Female | 0.13 | 0.06 | 0.03 | 0.02 | 0.24 | 0.01 |
| Other | -0.87 | 0.42 | 0.04 | -1.70 | -0.05 | . |
| Birth year/current age (Ref: 1998-2005; age 18-24) |  |  |  |  |  |  |
| 1993-1998; age 25-29 | 0.25 | 0.10 | 0.01 | 0.06 | 0.44 | 0.02 |
| 1983-1993; age 30-39 | 0.23 | 0.08 | 0.01 | 0.07 | 0.39 | . |
| 1973-1983; age 40-49 | 0.16 | 0.09 | 0.10 | -0.03 | 0.34 | . |
| 1963-1973; age 50-59 | 0.03 | 0.11 | 0.81 | -0.19 | 0.24 | . |
| 1953-1963; age 60-69 | 0.22 | 0.12 | 0.07 | -0.01 | 0.46 | . |
| 1943-1953; age 70-79 | -0.39 | 0.28 | 0.16 | -0.94 | 0.16 | . |
| 1943 or earlier; age 80 or older | -0.38 | 0.50 | 0.44 | -1.36 | 0.60 | . |
| Mother absence/presence (Ref: Present) |  |  |  |  |  |  |
| Absent | 0.03 | 0.24 | 0.90 | -0.44 | 0.50 | 0.90 |
| Father absence/presence (Ref: Present) |  |  |  |  |  |  |
| Absent | -0.07 | 0.16 | 0.66 | -0.39 | 0.25 | 0.66 |
| Childhood religion (Ref: Christianity) |  |  |  |  |  |  |
| Islam | 0.15 | 0.13 | 0.23 | -0.10 | 0.41 | 0.49 |
| Some other religion | 0.03 | 0.40 | 0.94 | -0.75 | 0.81 | . |
| Race/ethnicity (Ref: Ethnic plurality) |  |  |  |  |  |  |
| Ethnic minority | -0.17 | 0.06 | 0.01 | -0.30 | -0.05 | 0.01 |
| *Note*. *N* = 5,292. SE, standard error; LCI, lower confidence interval; UCI, upper confidence interval. | | | | | | |

*Table S14d. E-Values and E-Value Limits for the Coefficients Shown in Table S14c (Philippines)*

| Characteristic | *E*-value for estimate^a^ | *E*-value for 95% CI^b^ |
| --- | --- | --- |
| Relationship with mother (Ref: Very/somewhat bad) |  |  |
| Very/somewhat good | 1.15 | 1.00 |
| Relationship with father (Ref: Very/somewhat bad) |  |  |
| Very/somewhat good | 1.27 | 1.00 |
| Parent marital status (Ref: Married) |  |  |
| Divorced | 2.00 | 1.27 |
| Never married | 1.29 | 1.00 |
| One or both parents had died | 1.23 | 1.00 |
| Subjective financial status growing up (Ref: Got by) |  |  |
| Lived comfortably | 1.22 | 1.00 |
| Found it difficult | 1.13 | 1.00 |
| Found it very difficult | 1.31 | 1.00 |
| Childhood abuse (Ref: No) |  |  |
| Yes | 1.41 | 1.16 |
| Outsider growing up (Ref: No) |  |  |
| Yes | 1.08 | 1.00 |
| Childhood health (Ref: Good) |  |  |
| Excellent | 1.07 | 1.00 |
| Very good | 1.12 | 1.00 |
| Fair | 1.31 | 1.13 |
| Poor | 1.53 | 1.22 |
| Immigration status (Ref: Born in this country) |  |  |
| Born in another country | 1.40 | 1.00 |
| Childhood religious service attendance (Ref: Never) |  |  |
| At least 1x/week | 1.26 | 1.00 |
| 1-3x/month | 1.27 | 1.00 |
| <1x/month | 1.16 | 1.00 |
| Gender (Ref: Male) |  |  |
| Female | 1.24 | 1.08 |
| Other | 1.93 | 1.14 |
| Birth year/current age (Ref: 1998-2005; age 18-24) |  |  |
| 1993-1998; age 25-29 | 1.37 | 1.15 |
| 1983-1993; age 30-39 | 1.35 | 1.17 |
| 1973-1983; age 40-49 | 1.27 | 1.00 |
| 1963-1973; age 50-59 | 1.10 | 1.00 |
| 1953-1963; age 60-69 | 1.34 | 1.00 |
| 1943-1953; age 70-79 | 1.50 | 1.00 |
| 1943 or earlier; age 80 or older | 1.49 | 1.00 |
| Mother absence/presence (Ref: Present) |  |  |
| Absent | 1.11 | 1.00 |
| Father absence/presence (Ref: Present) |  |  |
| Absent | 1.17 | 1.00 |
| Childhood religion (Ref: Christianity) |  |  |
| Islam | 1.27 | 1.00 |
| Some other religion | 1.11 | 1.00 |
| Race/ethnicity (Ref: Ethnic plurality) |  |  |
| Ethnic minority | 1.29 | 1.14 |

*Note*. CI, confidence interval. ^a^The formula for calculating *E*-values can be found in VanderWeele and Ding (2017) The *E*-value for the effect estimate is the minimum strength of association (on the risk ratio scale) that an unmeasured confounder would need to have with both the predictor and the outcome to entirely explain away the observed association between them, conditional on the measured covariates. ^b^The *E*-value for the limit of the 95% confidence interval closest to the null denote the minimum strength of association (on the risk ratio scale) that an unmeasured confounder would need to have with both the predictor and the outcome to shift the confidence interval to include the null value, conditional on the measured covariates.

*Table S15a. Nationally Representative Descriptive Statistics of the Observed Sample (Poland)*

| Variable | Proportion | Frequency |
| --- | --- | --- |
| Sociodemographic characteristics |  |  |
| Birth year/current age |  |  |
| 1998-2005; age 18-24 | 0.09 | 955 |
| 1993-1998; age 25-29 | 0.07 | 761 |
| 1983-1993; age 30-39 | 0.21 | 2159 |
| 1973-1983; age 40-49 | 0.19 | 1956 |
| 1963-1973; age 50-59 | 0.16 | 1670 |
| 1953-1963; age 60-69 | 0.18 | 1909 |
| 1943-1953; age 70-79 | 0.08 | 833 |
| 1943 or earlier; 80 or older | 0.01 | 145 |
| Missing | 0.00 | 1 |
| Gender |  |  |
| Male | 0.48 | 4974 |
| Female | 0.52 | 5387 |
| Other | 0.00 | 3 |
| Missing | 0.00 | 26 |
| Marital status |  |  |
| Single, never married | 0.17 | 1811 |
| Married | 0.58 | 6065 |
| Separated | 0.01 | 111 |
| Divorced | 0.05 | 529 |
| Widowed | 0.10 | 990 |
| Domestic partner | 0.05 | 504 |
| Missing | 0.04 | 379 |
| Employment |  |  |
| Employed for an employer | 0.56 | 5837 |
| Self-employed | 0.07 | 686 |
| Retired | 0.23 | 2434 |
| Student | 0.05 | 515 |
| Homemaker | 0.03 | 338 |
| Unemployed and looking for a job | 0.03 | 284 |
| None of these/other | 0.02 | 169 |
| Missing | 0.01 | 126 |
| Education |  |  |
| Up to 8 years | 0.12 | 1238 |
| 9-15 years | 0.59 | 6130 |
| 16+ years | 0.29 | 3020 |
| Missing | 0.00 | 1 |
| Religious service attendance |  |  |
| >1x/week | 0.03 | 305 |
| 1x/week | 0.31 | 3263 |
| 1-3x/month | 0.20 | 2081 |
| A few times a year | 0.29 | 3064 |
| Never | 0.15 | 1597 |
| Missing | 0.01 | 78 |
| Immigration status |  |  |
| Born in this country | 0.99 | 10258 |
| Born in another country | 0.01 | 108 |
| Missing | 0.00 | 23 |
| Religion |  |  |
| Christianity | 0.90 | 9378 |
| Islam | 0.00 | 2 |
| Hinduism | . | . |
| Buddhism | 0.00 | 2 |
| Judaism | . | . |
| Sikhism | 0.00 | 1 |
| Baha'i | . | . |
| Jainism | 0.00 | 3 |
| Shinto | 0.00 | 1 |
| Taoism | . | . |
| Confucianism | . | . |
| Primal, Animist, or Folk Religion | 0.00 | 11 |
| Spiritism | . | . |
| African-derived | . | . |
| Chinese | . | . |
| Some other religion | . | . |
| No religion/atheist/agnostic | 0.09 | 942 |
| Missing | 0.00 | 50 |
| Race/ethnicity |  |  |
| Polish | 0.99 | 10309 |
| German | 0.00 | 4 |
| Belarussian | 0.00 | 2 |
| Ukranian | 0.00 | 38 |
| Roma | . | . |
| Russian | . | . |
| Ethnic Jewish | . | . |
| Lemko | . | . |
| Silesia | 0.00 | 14 |
| Kashubians | 0.00 | 3 |
| Other | 0.00 | 4 |
| Missing | 0.00 | 14 |
| Childhood factors |  |  |
| Relationship with mother |  |  |
| Very good | 0.47 | 4879 |
| Somewhat good | 0.48 | 4973 |
| Somewhat bad | 0.03 | 285 |
| Very bad | 0.01 | 58 |
| Not applicable | 0.01 | 80 |
| Missing | 0.01 | 112 |
| Relationship with father |  |  |
| Very good | 0.41 | 4231 |
| Somewhat good | 0.48 | 4984 |
| Somewhat bad | 0.05 | 516 |
| Very bad | 0.01 | 78 |
| Not applicable | 0.04 | 407 |
| Missing | 0.02 | 173 |
| Parent marital status |  |  |
| Married | 0.86 | 8972 |
| Divorced | 0.06 | 587 |
| Never married | 0.02 | 193 |
| One or both parents had died | 0.03 | 313 |
| Missing | 0.03 | 324 |
| Subjective financial status growing up |  |  |
| Lived comfortably | 0.13 | 1384 |
| Got by | 0.60 | 6257 |
| Found it difficult | 0.21 | 2133 |
| Found it very difficult | 0.05 | 509 |
| Missing | 0.01 | 106 |
| Childhood abuse |  |  |
| Yes | 0.03 | 325 |
| No | 0.96 | 10009 |
| Missing | 0.01 | 55 |
| Outsider growing up |  |  |
| Yes | 0.05 | 490 |
| No | 0.93 | 9615 |
| Not applicable | 0.00 | 33 |
| Missing | 0.02 | 252 |
| Childhood health |  |  |
| Excellent | 0.26 | 2676 |
| Very good | 0.52 | 5371 |
| Good | 0.17 | 1779 |
| Fair | 0.04 | 406 |
| Poor | 0.01 | 123 |
| Missing | 0.00 | 34 |
| Childhood religious service attendance |  |  |
| At least 1x/week | 0.46 | 4751 |
| 1-3x/month | 0.26 | 2689 |
| <1x/month | 0.21 | 2161 |
| Never | 0.03 | 354 |
| Missing | 0.04 | 434 |
| Childhood religion |  |  |
| Christianity | 0.95 | 9861 |
| Islam | 0.00 | 3 |
| Hinduism | . | . |
| Buddhism | 0.00 | 2 |
| Judaism | . | . |
| Sikhism | 0.00 | 1 |
| Baha'i | . | . |
| Jainism | . | . |
| Shinto | . | . |
| Taoism | . | . |
| Confucianism | . | . |
| Primal, Animist, or Folk Religion | 0.00 | 5 |
| Spiritism | . | . |
| African-derived | . | . |
| Chinese | . | . |
| Some other religion | . | . |
| No religion/atheist/agnostic | 0.05 | 482 |
| Missing | 0.00 | 35 |

*Note*. *N* = 10,389.

*Table S15b. Variations Across Sociodemographic Characteristics (Poland)*

| Characteristic | Mean | SE | LCI | UCI | Global *p*-value |
| --- | --- | --- | --- | --- | --- |
| Age group |  |  |  |  |  |
| 18-24 | 7.63 | 0.15 | 7.33 | 7.93 | 0.02 |
| 25-29 | 7.77 | 0.09 | 7.58 | 7.95 | . |
| 30-39 | 7.95 | 0.07 | 7.82 | 8.09 | . |
| 40-49 | 7.80 | 0.08 | 7.65 | 7.95 | . |
| 50-59 | 7.60 | 0.12 | 7.37 | 7.83 | . |
| 60-69 | 7.88 | 0.10 | 7.68 | 8.07 | . |
| 70-79 | 7.97 | 0.16 | 7.65 | 8.29 | . |
| 80 or older | 7.41 | 0.22 | 6.97 | 7.85 | . |
| Gender |  |  |  |  |  |
| Male | 7.58 | 0.08 | 7.43 | 7.72 | 0.00 |
| Female | 8.02 | 0.06 | 7.89 | 8.14 | . |
| Other | 5.34 | . | . | . | . |
| Marital status |  |  |  |  |  |
| Single, never married | 7.31 | 0.12 | 7.07 | 7.54 | 0.00 |
| Married | 8.02 | 0.06 | 7.90 | 8.14 | . |
| Separated | 7.40 | 0.16 | 7.08 | 7.72 | . |
| Divorced | 7.10 | 0.22 | 6.66 | 7.54 | . |
| Widowed | 7.68 | 0.14 | 7.41 | 7.95 | . |
| Domestic partner | 8.06 | 0.08 | 7.90 | 8.21 | . |
| Employment |  |  |  |  |  |
| Employed for an employer | 7.83 | 0.06 | 7.72 | 7.95 | 0.04 |
| Self-employed | 7.75 | 0.12 | 7.51 | 7.98 | . |
| Retired | 7.89 | 0.10 | 7.70 | 8.08 | . |
| Student | 7.54 | 0.21 | 7.13 | 7.95 | . |
| Homemaker | 7.98 | 0.22 | 7.54 | 8.43 | . |
| Unemployed and looking for a job | 7.03 | 0.32 | 6.40 | 7.66 | . |
| None of these/other | 7.59 | 0.19 | 7.22 | 7.96 | . |
| Education |  |  |  |  |  |
| Up to 8 years | 7.11 | 0.21 | 6.69 | 7.52 | 0.00 |
| 9-15 years | 7.83 | 0.06 | 7.71 | 7.95 | . |
| 16+ years | 8.03 | 0.06 | 7.91 | 8.14 | . |
| Religious service attendance |  |  |  |  |  |
| >1x/week | 7.97 | 0.25 | 7.47 | 8.46 | 0.00 |
| 1x/week | 8.09 | 0.08 | 7.94 | 8.24 | . |
| 1-3x/month | 7.72 | 0.11 | 7.50 | 7.93 | . |
| A few times a year | 7.74 | 0.06 | 7.61 | 7.87 | . |
| Never | 7.43 | 0.11 | 7.21 | 7.66 | . |
| Immigration status |  |  |  |  |  |
| Born in this country | 7.81 | 0.06 | 7.69 | 7.93 | 0.24 |
| Born in another country | 7.21 | 0.49 | 6.20 | 8.21 | . |
| Religion |  |  |  |  |  |
| Christianity | 7.83 | 0.06 | 7.70 | 7.95 | 0.00 |
| Islam | 9.66 | . | . | . | . |
| Hinduism | . | . | . | . | . |
| Buddhism | 6.26 | 2.45 | -14.90 | 27.42 | . |
| Judaism | . | . | . | . | . |
| Sikhism | 5.00 | . | . | . | . |
| Baha'i | . | . | . | . | . |
| Jainism | 9.00 | . | . | . | . |
| Shinto | 6.00 | . | . | . | . |
| Taoism | . | . | . | . | . |
| Confucianism | . | . | . | . | . |
| Primal, Animist, or Folk Religion | 7.88 | 0.39 | -65.21 | 80.97 | . |
| Spiritism | . | . | . | . | . |
| African-derived | . | . | . | . | . |
| Chinese | . | . | . | . | . |
| Some other religion | . | . | . | . | . |
| No religion/atheist/agnostic | 7.57 | 0.15 | 7.28 | 7.86 | . |
| Race/ethnicity |  |  |  |  |  |
| Polish | 7.80 | 0.06 | 7.68 | 7.92 | 0.00 |
| German | 6.61 | . | . | . | . |
| Belarussian | 7.20 | . | . | . | . |
| Ukranian | 8.40 | 0.18 | 7.93 | 8.86 | . |
| Roma | . | . | . | . | . |
| Russian | . | . | . | . | . |
| Ethnic Jewish | . | . | . | . | . |
| Lemko | . | . | . | . | . |
| Silesia | 8.49 | 0.25 | 7.40 | 9.58 | . |
| Kashubians | 6.58 | . | . | . | . |
| Other | 6.13 | 0.06 | -3.25 | 15.51 | . |

*Note*. *N* = 10,389. SE, standard error; LCI, lower confidence interval; UCI, upper confidence interval.

*Table S15c. Variations Across Childhood Predictors (Poland)*

| Characteristic | Coef. | SE | Prob. | LCI | UCI | Global *p*-value |
| --- | --- | --- | --- | --- | --- | --- |
| Relationship with mother (Ref: Very/somewhat bad) |  |  |  |  |  |  |
| Very/somewhat good | 0.31 | 0.23 | 0.17 | -0.14 | 0.75 | 0.17 |
| Relationship with father (Ref: Very/somewhat bad) |  |  |  |  |  |  |
| Very/somewhat good | 0.24 | 0.15 | 0.10 | -0.05 | 0.54 | 0.10 |
| Parent marital status (Ref: Married) |  |  |  |  |  |  |
| Divorced | -0.45 | 0.13 | 0.00 | -0.70 | -0.19 | 0.00 |
| Never married | -0.82 | 0.32 | 0.01 | -1.45 | -0.19 | . |
| One or both parents had died | -0.10 | 0.23 | 0.66 | -0.55 | 0.35 | . |
| Subjective financial status growing up (Ref: Got by) |  |  |  |  |  |  |
| Lived comfortably | -0.25 | 0.11 | 0.02 | -0.47 | -0.04 | 0.09 |
| Found it difficult | -0.03 | 0.09 | 0.75 | -0.21 | 0.15 | . |
| Found it very difficult | -0.29 | 0.27 | 0.29 | -0.81 | 0.24 | . |
| Childhood abuse (Ref: No) |  |  |  |  |  |  |
| Yes | -0.44 | 0.26 | 0.09 | -0.94 | 0.07 | 0.09 |
| Outsider growing up (Ref: No) |  |  |  |  |  |  |
| Yes | -0.27 | 0.20 | 0.19 | -0.68 | 0.14 | 0.19 |
| Childhood health (Ref: Good) |  |  |  |  |  |  |
| Excellent | 0.42 | 0.13 | 0.00 | 0.16 | 0.68 | 0.04 |
| Very good | 0.20 | 0.10 | 0.05 | 0.00 | 0.39 | . |
| Fair | -0.06 | 0.25 | 0.81 | -0.56 | 0.44 | . |
| Poor | 0.08 | 0.34 | 0.81 | -0.58 | 0.74 | . |
| Immigration status (Ref: Born in this country) |  |  |  |  |  |  |
| Born in another country | -0.60 | 0.61 | 0.33 | -1.80 | 0.60 | 0.33 |
| Childhood religious service attendance (Ref: Never) |  |  |  |  |  |  |
| At least 1x/week | 0.80 | 0.22 | 0.00 | 0.36 | 1.23 | 0.00 |
| 1-3x/month | 0.51 | 0.23 | 0.03 | 0.06 | 0.96 | . |
| <1x/month | 0.31 | 0.22 | 0.16 | -0.13 | 0.74 | . |
| Gender (Ref: Male) |  |  |  |  |  |  |
| Female | 0.40 | 0.06 | 0.00 | 0.28 | 0.52 | 0.00 |
| Other | -1.84 | 0.69 | 0.01 | -3.19 | -0.50 | . |
| Birth year/current age (Ref: 1998-2005; age 18-24) |  |  |  |  |  |  |
| 1993-1998; age 25-29 | 0.11 | 0.15 | 0.45 | -0.18 | 0.41 | 0.00 |
| 1983-1993; age 30-39 | 0.27 | 0.14 | 0.05 | 0.00 | 0.55 | . |
| 1973-1983; age 40-49 | 0.10 | 0.14 | 0.48 | -0.17 | 0.37 | . |
| 1963-1973; age 50-59 | -0.14 | 0.14 | 0.32 | -0.41 | 0.13 | . |
| 1953-1963; age 60-69 | 0.02 | 0.15 | 0.88 | -0.27 | 0.32 | . |
| 1943-1953; age 70-79 | 0.18 | 0.22 | 0.41 | -0.25 | 0.61 | . |
| 1943 or earlier; age 80 or older | -0.37 | 0.36 | 0.30 | -1.07 | 0.33 | . |
| Mother absence/presence (Ref: Present) |  |  |  |  |  |  |
| Absent | -0.31 | 0.65 | 0.63 | -1.59 | 0.97 | 0.63 |
| Father absence/presence (Ref: Present) |  |  |  |  |  |  |
| Absent | 0.03 | 0.25 | 0.89 | -0.45 | 0.52 | 0.89 |
| Childhood religion (Ref: No religion/atheist/agnostic) |  |  |  |  |  |  |
| Christianity | -0.25 | 0.20 | 0.22 | -0.64 | 0.15 | 0.40 |
| Some other religion | -0.38 | 0.50 | 0.44 | -1.36 | 0.59 | . |
| Race/ethnicity (Ref: Ethnic plurality) |  |  |  |  |  |  |
| Ethnic minority | 0.76 | 0.44 | 0.08 | -0.10 | 1.62 | 0.08 |
| *Note*. *N* = 10,389. SE, standard error; LCI, lower confidence interval; UCI, upper confidence interval. | | | | | | |

*Table S15c. E-Values and E-Value Limits for the Coefficients Shown in Table S15d (Poland)*

| Characteristic | *E*-value for estimate^a^ | *E*-value for 95% CI^b^ |
| --- | --- | --- |
| Relationship with mother (Ref: Very/somewhat bad) |  |  |
| Very/somewhat good | 1.45 | 1.00 |
| Relationship with father (Ref: Very/somewhat bad) |  |  |
| Very/somewhat good | 1.38 | 1.00 |
| Parent marital status (Ref: Married) |  |  |
| Divorced | 1.58 | 1.33 |
| Never married | 1.94 | 1.32 |
| One or both parents had died | 1.22 | 1.00 |
| Subjective financial status growing up (Ref: Got by) |  |  |
| Lived comfortably | 1.39 | 1.12 |
| Found it difficult | 1.11 | 1.00 |
| Found it very difficult | 1.43 | 1.00 |
| Childhood abuse (Ref: No) |  |  |
| Yes | 1.57 | 1.00 |
| Outsider growing up (Ref: No) |  |  |
| Yes | 1.41 | 1.00 |
| Childhood health (Ref: Good) |  |  |
| Excellent | 1.56 | 1.29 |
| Very good | 1.33 | 1.02 |
| Fair | 1.16 | 1.00 |
| Poor | 1.19 | 1.00 |
| Immigration status (Ref: Born in this country) |  |  |
| Born in another country | 1.73 | 1.00 |
| Childhood religious service attendance (Ref: Never) |  |  |
| At least 1x/week | 1.92 | 1.51 |
| 1-3x/month | 1.65 | 1.17 |
| <1x/month | 1.45 | 1.00 |
| Gender (Ref: Male) |  |  |
| Female | 1.54 | 1.42 |
| Other | 3.04 | 1.63 |
| Birth year/current age (Ref: 1998-2005; age 18-24) |  |  |
| 1993-1998; age 25-29 | 1.24 | 1.00 |
| 1983-1993; age 30-39 | 1.41 | 1.03 |
| 1973-1983; age 40-49 | 1.22 | 1.00 |
| 1963-1973; age 50-59 | 1.26 | 1.00 |
| 1953-1963; age 60-69 | 1.10 | 1.00 |
| 1943-1953; age 70-79 | 1.31 | 1.00 |
| 1943 or earlier; age 80 or older | 1.51 | 1.00 |
| Mother absence/presence (Ref: Present) |  |  |
| Absent | 1.45 | 1.00 |
| Father absence/presence (Ref: Present) |  |  |
| Absent | 1.11 | 1.00 |
| Childhood religion (Ref: No religion/atheist/agnostic) |  |  |
| Christianity | 1.38 | 1.00 |
| Some other religion | 1.52 | 1.00 |
| Race/ethnicity (Ref: Ethnic plurality) |  |  |
| Ethnic minority | 1.88 | 1.00 |

*Note*. CI, confidence interval. ^a^The formula for calculating *E*-values can be found in VanderWeele and Ding (2017) The *E*-value for the effect estimate is the minimum strength of association (on the risk ratio scale) that an unmeasured confounder would need to have with both the predictor and the outcome to entirely explain away the observed association between them, conditional on the measured covariates. ^b^The *E*-value for the limit of the 95% confidence interval closest to the null denote the minimum strength of association (on the risk ratio scale) that an unmeasured confounder would need to have with both the predictor and the outcome to shift the confidence interval to include the null value, conditional on the measured covariates.

*Table S16a. Nationally Representative Descriptive Statistics of the Observed Sample (South Africa)*

| Variable | Proportion | Frequency |
| --- | --- | --- |
| Sociodemographic characteristics |  |  |
| Birth year/current age |  |  |
| 1998-2005; age 18-24 | 0.17 | 461 |
| 1993-1998; age 25-29 | 0.14 | 364 |
| 1983-1993; age 30-39 | 0.25 | 655 |
| 1973-1983; age 40-49 | 0.20 | 522 |
| 1963-1973; age 50-59 | 0.12 | 309 |
| 1953-1963; age 60-69 | 0.07 | 195 |
| 1943-1953; age 70-79 | 0.05 | 120 |
| 1943 or earlier; 80 or older | 0.01 | 17 |
| Missing | 0.00 | 9 |
| Gender |  |  |
| Male | 0.49 | 1288 |
| Female | 0.51 | 1356 |
| Other | 0.00 | 2 |
| Missing | 0.00 | 4 |
| Marital status |  |  |
| Single, never married | 0.59 | 1561 |
| Married | 0.20 | 539 |
| Separated | 0.03 | 76 |
| Divorced | 0.02 | 51 |
| Widowed | 0.05 | 133 |
| Domestic partner | 0.10 | 264 |
| Missing | 0.01 | 28 |
| Employment |  |  |
| Employed for an employer | 0.21 | 569 |
| Self-employed | 0.16 | 412 |
| Retired | 0.09 | 243 |
| Student | 0.08 | 204 |
| Homemaker | 0.05 | 137 |
| Unemployed and looking for a job | 0.38 | 1008 |
| None of these/other | 0.03 | 74 |
| Missing | 0.00 | 3 |
| Education |  |  |
| Up to 8 years | 0.25 | 668 |
| 9-15 years | 0.68 | 1796 |
| 16+ years | 0.07 | 183 |
| Missing | 0.00 | 4 |
| Religious service attendance |  |  |
| >1x/week | 0.16 | 414 |
| 1x/week | 0.34 | 891 |
| 1-3x/month | 0.22 | 574 |
| A few times a year | 0.16 | 431 |
| Never | 0.13 | 334 |
| Missing | 0.00 | 7 |
| Immigration status |  |  |
| Born in this country | 0.95 | 2511 |
| Born in another country | 0.05 | 139 |
| Missing | 0.00 | 1 |
| Religion |  |  |
| Christianity | 0.82 | 2163 |
| Islam | 0.02 | 62 |
| Hinduism | 0.00 | 1 |
| Buddhism | 0.00 | 12 |
| Judaism | . | . |
| Sikhism | . | . |
| Baha'i | . | . |
| Jainism | 0.00 | 2 |
| Shinto | 0.00 | 2 |
| Taoism | 0.00 | 1 |
| Confucianism | . | . |
| Primal, Animist, or Folk Religion | 0.05 | 127 |
| Spiritism | . | . |
| African-derived | . | . |
| Chinese | . | . |
| Some other religion | 0.00 | 5 |
| No religion/atheist/agnostic | 0.10 | 253 |
| Missing | 0.01 | 23 |
| Race/ethnicity |  |  |
| Black | 0.90 | 2381 |
| Asian/Indian | 0.00 | 6 |
| Colored | 0.10 | 252 |
| White | 0.00 | 8 |
| Other | 0.00 | 1 |
| Missing | 0.00 | 3 |
| Childhood factors |  |  |
| Relationship with mother |  |  |
| Very good | 0.82 | 2186 |
| Somewhat good | 0.10 | 263 |
| Somewhat bad | 0.02 | 51 |
| Very bad | 0.01 | 39 |
| Not applicable | 0.03 | 90 |
| Missing | 0.01 | 21 |
| Relationship with father |  |  |
| Very good | 0.62 | 1656 |
| Somewhat good | 0.13 | 333 |
| Somewhat bad | 0.03 | 86 |
| Very bad | 0.06 | 159 |
| Not applicable | 0.12 | 331 |
| Missing | 0.03 | 85 |
| Parent marital status |  |  |
| Married | 0.50 | 1321 |
| Divorced | 0.05 | 131 |
| Never married | 0.34 | 904 |
| One or both parents had died | 0.05 | 140 |
| Missing | 0.06 | 155 |
| Subjective financial status growing up |  |  |
| Lived comfortably | 0.40 | 1050 |
| Got by | 0.33 | 875 |
| Found it difficult | 0.16 | 432 |
| Found it very difficult | 0.11 | 289 |
| Missing | 0.00 | 5 |
| Childhood abuse |  |  |
| Yes | 0.17 | 450 |
| No | 0.81 | 2149 |
| Missing | 0.02 | 52 |
| Outsider growing up |  |  |
| Yes | 0.16 | 434 |
| No | 0.83 | 2211 |
| Not applicable | 0.00 | 3 |
| Missing | 0.00 | 3 |
| Childhood health |  |  |
| Excellent | 0.46 | 1225 |
| Very good | 0.22 | 590 |
| Good | 0.14 | 370 |
| Fair | 0.10 | 266 |
| Poor | 0.07 | 183 |
| Missing | 0.01 | 17 |
| Childhood religious service attendance |  |  |
| At least 1x/week | 0.63 | 1681 |
| 1-3x/month | 0.21 | 552 |
| <1x/month | 0.07 | 175 |
| Never | 0.08 | 217 |
| Missing | 0.01 | 26 |
| Childhood religion |  |  |
| Christianity | 0.88 | 2323 |
| Islam | 0.02 | 52 |
| Hinduism | 0.00 | 2 |
| Buddhism | 0.00 | 11 |
| Judaism | . | . |
| Sikhism | . | . |
| Baha'i | . | . |
| Jainism | . | . |
| Shinto | 0.00 | 2 |
| Taoism | 0.00 | 1 |
| Confucianism | . | . |
| Primal, Animist, or Folk Religion | 0.04 | 117 |
| Spiritism | . | . |
| African-derived | . | . |
| Chinese | . | . |
| Some other religion | 0.00 | 7 |
| No religion/atheist/agnostic | 0.04 | 107 |
| Missing | 0.01 | 27 |

*Note*. *N* = 2,651.

*Table S16b. Variations Across Sociodemographic Characteristics (South Africa)*

| Characteristic | Mean | SE | LCI | UCI | Global *p*-value |
| --- | --- | --- | --- | --- | --- |
| Age group |  |  |  |  |  |
| 18-24 | 8.62 | 0.11 | 8.40 | 8.84 | 0.24 |
| 25-29 | 8.37 | 0.12 | 8.12 | 8.61 | . |
| 30-39 | 8.65 | 0.10 | 8.46 | 8.84 | . |
| 40-49 | 8.52 | 0.12 | 8.27 | 8.76 | . |
| 50-59 | 8.70 | 0.19 | 8.32 | 9.08 | . |
| 60-69 | 8.97 | 0.22 | 8.53 | 9.42 | . |
| 70-79 | 8.16 | 0.40 | 7.33 | 8.99 | . |
| 80 or older | 8.92 | 0.51 | 4.53 | 13.30 | . |
| Gender |  |  |  |  |  |
| Male | 8.59 | 0.08 | 8.42 | 8.76 | 0.00 |
| Female | 8.59 | 0.08 | 8.44 | 8.74 | . |
| Other | 6.29 | . | . | . | . |
| Marital status |  |  |  |  |  |
| Single, never married | 8.49 | 0.07 | 8.36 | 8.62 | 0.06 |
| Married | 8.77 | 0.15 | 8.47 | 9.07 | . |
| Separated | 8.54 | 0.23 | 8.07 | 9.01 | . |
| Divorced | 8.28 | 0.32 | 7.60 | 8.96 | . |
| Widowed | 9.11 | 0.22 | 8.66 | 9.56 | . |
| Domestic partner | 8.60 | 0.15 | 8.30 | 8.91 | . |
| Employment |  |  |  |  |  |
| Employed for an employer | 8.59 | 0.09 | 8.40 | 8.77 | 0.32 |
| Self-employed | 8.44 | 0.18 | 8.08 | 8.80 | . |
| Retired | 8.78 | 0.23 | 8.32 | 9.24 | . |
| Student | 8.67 | 0.13 | 8.41 | 8.93 | . |
| Homemaker | 8.64 | 0.21 | 8.21 | 9.07 | . |
| Unemployed and looking for a job | 8.54 | 0.08 | 8.38 | 8.71 | . |
| None of these/other | 9.11 | 0.21 | 8.68 | 9.54 | . |
| Education |  |  |  |  |  |
| Up to 8 years | 8.74 | 0.14 | 8.46 | 9.02 | 0.39 |
| 9-15 years | 8.54 | 0.07 | 8.41 | 8.67 | . |
| 16+ years | 8.55 | 0.13 | 8.28 | 8.81 | . |
| Religious service attendance |  |  |  |  |  |
| >1x/week | 8.75 | 0.14 | 8.46 | 9.03 | 0.21 |
| 1x/week | 8.64 | 0.09 | 8.46 | 8.82 | . |
| 1-3x/month | 8.63 | 0.10 | 8.43 | 8.83 | . |
| A few times a year | 8.30 | 0.15 | 8.01 | 8.59 | . |
| Never | 8.56 | 0.14 | 8.29 | 8.83 | . |
| Immigration status |  |  |  |  |  |
| Born in this country | 8.62 | 0.06 | 8.50 | 8.73 | 0.13 |
| Born in another country | 8.04 | 0.38 | 7.27 | 8.82 | . |
| Religion |  |  |  |  |  |
| Christianity | 8.57 | 0.07 | 8.44 | 8.71 | 0.00 |
| Islam | 8.47 | 0.38 | 7.67 | 9.26 | . |
| Hinduism | 10.00 | . | . | . | . |
| Buddhism | 9.05 | 0.40 | 7.91 | 10.20 | . |
| Judaism | . | . | . | . | . |
| Sikhism | . | . | . | . | . |
| Baha'i | . | . | . | . | . |
| Jainism | 10.00 | . | . | . | . |
| Shinto | 9.41 | . | . | . | . |
| Taoism | 10.00 | . | . | . | . |
| Confucianism | . | . | . | . | . |
| Primal, Animist, or Folk Religion | 8.41 | 0.28 | 7.84 | 8.97 | . |
| Spiritism | . | . | . | . | . |
| African-derived | . | . | . | . | . |
| Chinese | . | . | . | . | . |
| Some other religion | 7.74 | 0.76 | -806.01 | 821.48 | . |
| No religion/atheist/agnostic | 8.80 | 0.12 | 8.57 | 9.04 | . |
| Race/ethnicity |  |  |  |  |  |
| Black | 8.59 | 0.06 | 8.46 | 8.71 | 0.02 |
| Asian/Indian | 9.14 | 0.25 | -31.63 | 49.92 | . |
| Colored | 8.59 | 0.16 | 8.27 | 8.90 | . |
| White | 8.41 | 0.73 | 2.13 | 14.69 | . |
| Other | 10.00 | . | . | . | . |

*Note*. *N* = 2,651. SE, standard error; LCI, lower confidence interval; UCI, upper confidence interval.

*Table S16c. Variations Across Childhood Predictors (South Africa)*

| Characteristic | Coef. | SE | Prob. | LCI | UCI | Global *p*-value |
| --- | --- | --- | --- | --- | --- | --- |
| Relationship with mother (Ref: Very/somewhat bad) |  |  |  |  |  |  |
| Very/somewhat good | -0.24 | 0.22 | 0.28 | -0.69 | 0.20 | 0.28 |
| Relationship with father (Ref: Very/somewhat bad) |  |  |  |  |  |  |
| Very/somewhat good | -0.30 | 0.14 | 0.03 | -0.57 | -0.03 | 0.03 |
| Parent marital status (Ref: Married) |  |  |  |  |  |  |
| Divorced | 0.03 | 0.24 | 0.89 | -0.45 | 0.52 | 0.74 |
| Never married | -0.13 | 0.12 | 0.29 | -0.36 | 0.11 | . |
| One or both parents had died | -0.09 | 0.26 | 0.74 | -0.59 | 0.42 | . |
| Subjective financial status growing up (Ref: Got by) |  |  |  |  |  |  |
| Lived comfortably | 0.17 | 0.11 | 0.14 | -0.05 | 0.39 | 0.35 |
| Found it difficult | 0.00 | 0.16 | 0.99 | -0.32 | 0.32 | . |
| Found it very difficult | -0.04 | 0.23 | 0.85 | -0.49 | 0.40 | . |
| Childhood abuse (Ref: No) |  |  |  |  |  |  |
| Yes | -0.46 | 0.16 | 0.00 | -0.77 | -0.16 | 0.00 |
| Outsider growing up (Ref: No) |  |  |  |  |  |  |
| Yes | 0.11 | 0.13 | 0.41 | -0.15 | 0.37 | 0.41 |
| Childhood health (Ref: Good) |  |  |  |  |  |  |
| Excellent | 0.14 | 0.15 | 0.35 | -0.16 | 0.44 | 0.22 |
| Very good | 0.08 | 0.16 | 0.64 | -0.25 | 0.40 | . |
| Fair | 0.29 | 0.22 | 0.19 | -0.14 | 0.72 | . |
| Poor | -0.39 | 0.26 | 0.13 | -0.90 | 0.11 | . |
| Immigration status (Ref: Born in this country) |  |  |  |  |  |  |
| Born in another country | -0.63 | 0.36 | 0.09 | -1.34 | 0.09 | 0.09 |
| Childhood religious service attendance (Ref: Never) |  |  |  |  |  |  |
| At least 1x/week | 0.18 | 0.29 | 0.55 | -0.40 | 0.75 | 0.45 |
| 1-3x/month | 0.17 | 0.29 | 0.56 | -0.41 | 0.75 | . |
| <1x/month | -0.18 | 0.37 | 0.62 | -0.92 | 0.55 | . |
| Gender (Ref: Male) |  |  |  |  |  |  |
| Female | -0.03 | 0.10 | 0.75 | -0.24 | 0.17 | 0.00 |
| Other | -2.37 | 0.46 | 0.00 | -3.27 | -1.47 | . |
| Birth year/current age (Ref: 1998-2005; age 18-24) |  |  |  |  |  |  |
| 1993-1998; age 25-29 | -0.22 | 0.16 | 0.16 | -0.53 | 0.09 | 0.24 |
| 1983-1993; age 30-39 | 0.07 | 0.14 | 0.62 | -0.21 | 0.35 | . |
| 1973-1983; age 40-49 | -0.03 | 0.17 | 0.88 | -0.35 | 0.30 | . |
| 1963-1973; age 50-59 | 0.16 | 0.20 | 0.43 | -0.23 | 0.55 | . |
| 1953-1963; age 60-69 | 0.36 | 0.25 | 0.15 | -0.13 | 0.85 | . |
| 1943-1953; age 70-79 | -0.45 | 0.41 | 0.27 | -1.26 | 0.35 | . |
| 1943 or earlier; age 80 or older | 0.22 | 0.55 | 0.69 | -0.86 | 1.30 | . |
| Mother absence/presence (Ref: Present) |  |  |  |  |  |  |
| Absent | 0.02 | 0.33 | 0.96 | -0.63 | 0.66 | 0.96 |
| Father absence/presence (Ref: Present) |  |  |  |  |  |  |
| Absent | -0.32 | 0.17 | 0.07 | -0.66 | 0.02 | 0.07 |
| Childhood religion (Ref: No religion/atheist/agnostic) |  |  |  |  |  |  |
| Christianity | -0.62 | 0.34 | 0.07 | -1.29 | 0.05 | 0.27 |
| Primal, Animist, or Folk Religion | -0.47 | 0.38 | 0.21 | -1.22 | 0.28 | . |
| Some other religion | -0.80 | 0.46 | 0.09 | -1.72 | 0.11 | . |
| Race/ethnicity (Ref: Ethnic plurality) |  |  |  |  |  |  |
| Ethnic minority | -0.05 | 0.17 | 0.78 | -0.39 | 0.29 | 0.78 |
| *Note*. *N* = 2,651. SE, standard error; LCI, lower confidence interval; UCI, upper confidence interval. | | | | | | |

*Table S16d. E-Values and E-Value Limits for the Coefficients Shown in Table S16c (South Africa)*

| Characteristic | *E*-value for estimate^a^ | *E*-value for 95% CI^b^ |
| --- | --- | --- |
| Relationship with mother (Ref: Very/somewhat bad) |  |  |
| Very/somewhat good | 1.37 | 1.00 |
| Relationship with father (Ref: Very/somewhat bad) |  |  |
| Very/somewhat good | 1.43 | 1.12 |
| Parent marital status (Ref: Married) |  |  |
| Divorced | 1.11 | 1.00 |
| Never married | 1.24 | 1.00 |
| One or both parents had died | 1.19 | 1.00 |
| Subjective financial status growing up (Ref: Got by) |  |  |
| Lived comfortably | 1.29 | 1.00 |
| Found it difficult | 1.02 | 1.00 |
| Found it very difficult | 1.13 | 1.00 |
| Childhood abuse (Ref: No) |  |  |
| Yes | 1.58 | 1.28 |
| Outsider growing up (Ref: No) |  |  |
| Yes | 1.22 | 1.00 |
| Childhood health (Ref: Good) |  |  |
| Excellent | 1.26 | 1.00 |
| Very good | 1.18 | 1.00 |
| Fair | 1.41 | 1.00 |
| Poor | 1.51 | 1.00 |
| Immigration status (Ref: Born in this country) |  |  |
| Born in another country | 1.73 | 1.00 |
| Childhood religious service attendance (Ref: Never) |  |  |
| At least 1x/week | 1.30 | 1.00 |
| 1-3x/month | 1.29 | 1.00 |
| <1x/month | 1.31 | 1.00 |
| Gender (Ref: Male) |  |  |
| Female | 1.11 | 1.00 |
| Other | 3.59 | 2.54 |
| Birth year/current age (Ref: 1998-2005; age 18-24) |  |  |
| 1993-1998; age 25-29 | 1.35 | 1.00 |
| 1983-1993; age 30-39 | 1.17 | 1.00 |
| 1973-1983; age 40-49 | 1.10 | 1.00 |
| 1963-1973; age 50-59 | 1.28 | 1.00 |
| 1953-1963; age 60-69 | 1.48 | 1.00 |
| 1943-1953; age 70-79 | 1.57 | 1.00 |
| 1943 or earlier; age 80 or older | 1.35 | 1.00 |
| Mother absence/presence (Ref: Present) |  |  |
| Absent | 1.07 | 1.00 |
| Father absence/presence (Ref: Present) |  |  |
| Absent | 1.44 | 1.00 |
| Childhood religion (Ref: No religion/atheist/agnostic) |  |  |
| Christianity | 1.72 | 1.00 |
| Primal, Animist, or Folk Religion | 1.59 | 1.00 |
| Some other religion | 1.88 | 1.00 |
| Race/ethnicity (Ref: Ethnic plurality) |  |  |
| Ethnic minority | 1.14 | 1.00 |

*Note*. CI, confidence interval. ^a^The formula for calculating *E*-values can be found in VanderWeele and Ding (2017) The *E*-value for the effect estimate is the minimum strength of association (on the risk ratio scale) that an unmeasured confounder would need to have with both the predictor and the outcome to entirely explain away the observed association between them, conditional on the measured covariates. ^b^The *E*-value for the limit of the 95% confidence interval closest to the null denote the minimum strength of association (on the risk ratio scale) that an unmeasured confounder would need to have with both the predictor and the outcome to shift the confidence interval to include the null value, conditional on the measured covariates.

*Table S17a. Nationally Representative Descriptive Statistics of the Observed Sample (Spain)*

| Variable | Proportion | Frequency |
| --- | --- | --- |
| Sociodemographic characteristics |  |  |
| Birth year/current age |  |  |
| 1998-2005; age 18-24 | 0.09 | 594 |
| 1993-1998; age 25-29 | 0.07 | 450 |
| 1983-1993; age 30-39 | 0.18 | 1111 |
| 1973-1983; age 40-49 | 0.22 | 1396 |
| 1963-1973; age 50-59 | 0.20 | 1252 |
| 1953-1963; age 60-69 | 0.16 | 977 |
| 1943-1953; age 70-79 | 0.07 | 467 |
| 1943 or earlier; 80 or older | 0.01 | 43 |
| Missing | . | . |
| Gender |  |  |
| Male | 0.50 | 3142 |
| Female | 0.50 | 3119 |
| Other | 0.00 | 6 |
| Missing | 0.00 | 23 |
| Marital status |  |  |
| Single, never married | 0.28 | 1742 |
| Married | 0.47 | 2947 |
| Separated | 0.04 | 237 |
| Divorced | 0.08 | 518 |
| Widowed | 0.03 | 189 |
| Domestic partner | 0.09 | 589 |
| Missing | 0.01 | 67 |
| Employment |  |  |
| Employed for an employer | 0.45 | 2862 |
| Self-employed | 0.09 | 576 |
| Retired | 0.20 | 1278 |
| Student | 0.07 | 448 |
| Homemaker | 0.05 | 345 |
| Unemployed and looking for a job | 0.10 | 646 |
| None of these/other | 0.02 | 123 |
| Missing | 0.00 | 11 |
| Education |  |  |
| Up to 8 years | 0.13 | 802 |
| 9-15 years | 0.66 | 4145 |
| 16+ years | 0.21 | 1341 |
| Missing | 0.00 | 2 |
| Religious service attendance |  |  |
| >1x/week | 0.05 | 317 |
| 1x/week | 0.11 | 662 |
| 1-3x/month | 0.07 | 437 |
| A few times a year | 0.31 | 1972 |
| Never | 0.46 | 2875 |
| Missing | 0.00 | 27 |
| Immigration status |  |  |
| Born in this country | 0.87 | 5479 |
| Born in another country | 0.13 | 788 |
| Missing | 0.00 | 23 |
| Religion |  |  |
| Christianity | 0.65 | 4074 |
| Islam | 0.02 | 135 |
| Hinduism | 0.00 | 7 |
| Buddhism | 0.01 | 36 |
| Judaism | 0.00 | 4 |
| Sikhism | 0.00 | 3 |
| Baha'i | 0.00 | 2 |
| Jainism | 0.00 | 1 |
| Shinto | . | . |
| Taoism | 0.00 | 5 |
| Confucianism | 0.00 | 3 |
| Primal, Animist, or Folk Religion | 0.00 | 7 |
| Spiritism | . | . |
| African-derived | . | . |
| Chinese | . | . |
| Some other religion | 0.00 | 27 |
| No religion/atheist/agnostic | 0.31 | 1932 |
| Missing | 0.01 | 55 |
| Race/ethnicity |  |  |
| No data | . | . |
| Childhood factors |  |  |
| Relationship with mother |  |  |
| Very good | 0.72 | 4557 |
| Somewhat good | 0.20 | 1258 |
| Somewhat bad | 0.04 | 248 |
| Very bad | 0.01 | 92 |
| Not applicable | 0.02 | 107 |
| Missing | 0.00 | 28 |
| Relationship with father |  |  |
| Very good | 0.66 | 4131 |
| Somewhat good | 0.22 | 1397 |
| Somewhat bad | 0.05 | 309 |
| Very bad | 0.03 | 178 |
| Not applicable | 0.04 | 243 |
| Missing | 0.01 | 33 |
| Parent marital status |  |  |
| Married | 0.84 | 5285 |
| Divorced | 0.06 | 378 |
| Never married | 0.05 | 312 |
| One or both parents had died | 0.02 | 126 |
| Missing | 0.03 | 188 |
| Subjective financial status growing up |  |  |
| Lived comfortably | 0.32 | 2041 |
| Got by | 0.47 | 2956 |
| Found it difficult | 0.18 | 1154 |
| Found it very difficult | 0.02 | 110 |
| Missing | 0.00 | 29 |
| Childhood abuse |  |  |
| Yes | 0.10 | 659 |
| No | 0.88 | 5510 |
| Missing | 0.02 | 122 |
| Outsider growing up |  |  |
| Yes | 0.09 | 579 |
| No | 0.90 | 5637 |
| Not applicable | 0.01 | 36 |
| Missing | 0.01 | 39 |
| Childhood health |  |  |
| Excellent | 0.39 | 2450 |
| Very good | 0.36 | 2286 |
| Good | 0.20 | 1235 |
| Fair | 0.03 | 164 |
| Poor | 0.02 | 135 |
| Missing | 0.00 | 20 |
| Childhood religious service attendance |  |  |
| At least 1x/week | 0.38 | 2391 |
| 1-3x/month | 0.18 | 1132 |
| <1x/month | 0.20 | 1287 |
| Never | 0.23 | 1445 |
| Missing | 0.01 | 36 |
| Childhood religion |  |  |
| Christianity | 0.81 | 5119 |
| Islam | 0.02 | 132 |
| Hinduism | 0.00 | 5 |
| Buddhism | 0.00 | 8 |
| Judaism | 0.00 | 5 |
| Sikhism | 0.00 | 2 |
| Baha'i | . | . |
| Jainism | . | . |
| Shinto | . | . |
| Taoism | . | . |
| Confucianism | 0.00 | 1 |
| Primal, Animist, or Folk Religion | 0.00 | 4 |
| Spiritism | . | . |
| African-derived | . | . |
| Chinese | . | . |
| Some other religion | 0.00 | 13 |
| No religion/atheist/agnostic | 0.15 | 972 |
| Missing | 0.00 | 29 |

*Note*. *N* = 6,290.

*Table S17b. Variations Across Sociodemographic Characteristics (Spain)*

| Characteristic | Mean | SE | LCI | UCI | Global *p*-value |
| --- | --- | --- | --- | --- | --- |
| Age group |  |  |  |  |  |
| 18-24 | 7.79 | 0.12 | 7.56 | 8.02 | 0.00 |
| 25-29 | 8.06 | 0.12 | 7.82 | 8.31 | . |
| 30-39 | 8.13 | 0.07 | 7.99 | 8.27 | . |
| 40-49 | 8.33 | 0.05 | 8.22 | 8.43 | . |
| 50-59 | 8.50 | 0.06 | 8.37 | 8.62 | . |
| 60-69 | 8.45 | 0.09 | 8.27 | 8.63 | . |
| 70-79 | 8.35 | 0.17 | 8.01 | 8.68 | . |
| 80 or older | 9.10 | 0.19 | 8.70 | 9.50 | . |
| Gender |  |  |  |  |  |
| Male | 8.10 | 0.05 | 8.00 | 8.19 | 0.00 |
| Female | 8.47 | 0.04 | 8.39 | 8.56 | . |
| Other | 7.84 | 0.41 | 6.90 | 8.78 | . |
| Marital status |  |  |  |  |  |
| Single, never married | 7.96 | 0.06 | 7.84 | 8.09 | 0.00 |
| Married | 8.47 | 0.04 | 8.38 | 8.56 | . |
| Separated | 8.04 | 0.17 | 7.71 | 8.36 | . |
| Divorced | 8.26 | 0.13 | 8.01 | 8.51 | . |
| Widowed | 8.18 | 0.24 | 7.71 | 8.66 | . |
| Domestic partner | 8.45 | 0.08 | 8.29 | 8.62 | . |
| Employment |  |  |  |  |  |
| Employed for an employer | 8.31 | 0.04 | 8.23 | 8.39 | 0.00 |
| Self-employed | 8.33 | 0.09 | 8.15 | 8.51 | . |
| Retired | 8.34 | 0.09 | 8.16 | 8.53 | . |
| Student | 7.74 | 0.13 | 7.48 | 7.99 | . |
| Homemaker | 8.57 | 0.12 | 8.32 | 8.81 | . |
| Unemployed and looking for a job | 8.25 | 0.09 | 8.08 | 8.43 | . |
| None of these/other | 8.07 | 0.21 | 7.65 | 8.49 | . |
| Education |  |  |  |  |  |
| Up to 8 years | 8.22 | 0.12 | 7.98 | 8.47 | 0.27 |
| 9-15 years | 8.27 | 0.04 | 8.19 | 8.34 | . |
| 16+ years | 8.37 | 0.06 | 8.26 | 8.49 | . |
| Religious service attendance |  |  |  |  |  |
| >1x/week | 8.41 | 0.14 | 8.13 | 8.69 | 0.21 |
| 1x/week | 8.39 | 0.09 | 8.21 | 8.58 | . |
| 1-3x/month | 8.09 | 0.13 | 7.83 | 8.36 | . |
| A few times a year | 8.33 | 0.06 | 8.22 | 8.45 | . |
| Never | 8.24 | 0.05 | 8.14 | 8.33 | . |
| Immigration status |  |  |  |  |  |
| Born in this country | 8.24 | 0.04 | 8.17 | 8.31 | 0.00 |
| Born in another country | 8.56 | 0.08 | 8.40 | 8.73 | . |
| Religion |  |  |  |  |  |
| Christianity | 8.36 | 0.04 | 8.28 | 8.44 | 0.00 |
| Islam | 7.43 | 0.30 | 6.85 | 8.02 | . |
| Hinduism | 7.67 | 0.58 | 5.19 | 10.15 | . |
| Buddhism | 8.05 | 0.70 | 6.63 | 9.48 | . |
| Judaism | 8.09 | 1.16 | 3.11 | 13.07 | . |
| Sikhism | 5.32 | 0.62 | -97.06 | 107.70 | . |
| Baha'i | 9.60 | . | . | . | . |
| Jainism | 10.00 | . | . | . | . |
| Shinto | . | . | . | . | . |
| Taoism | 7.43 | 0.73 | -113.20 | 128.06 | . |
| Confucianism | 3.48 | 2.66 | -19.50 | 26.47 | . |
| Primal, Animist, or Folk Religion | 7.54 | 0.61 | 6.21 | 8.86 | . |
| Spiritism | . | . | . | . | . |
| African-derived | . | . | . | . | . |
| Chinese | . | . | . | . | . |
| Some other religion | 8.83 | 0.39 | 8.04 | 9.62 | . |
| No religion/atheist/agnostic | 8.19 | 0.06 | 8.08 | 8.30 | . |
| Race/ethnicity |  |  |  |  |  |
| No data | . | . | . | . | . |

*Note*. *N* = 6,290. SE, standard error; LCI, lower confidence interval; UCI, upper confidence interval.

*Table S17c. Variations Across Childhood Predictors (Spain)*

| Characteristic | Coef. | SE | Prob. | LCI | UCI | Global *p*-value |
| --- | --- | --- | --- | --- | --- | --- |
| Relationship with mother (Ref: Very/somewhat bad) |  |  |  |  |  |  |
| Very/somewhat good | 0.66 | 0.17 | 0.00 | 0.32 | 1.00 | 0.00 |
| Relationship with father (Ref: Very/somewhat bad) |  |  |  |  |  |  |
| Very/somewhat good | -0.02 | 0.13 | 0.85 | -0.27 | 0.22 | 0.85 |
| Parent marital status (Ref: Married) |  |  |  |  |  |  |
| Divorced | -0.23 | 0.15 | 0.13 | -0.53 | 0.07 | 0.23 |
| Never married | -0.15 | 0.14 | 0.31 | -0.43 | 0.14 | . |
| One or both parents had died | -0.37 | 0.28 | 0.19 | -0.91 | 0.18 | . |
| Subjective financial status growing up (Ref: Got by) |  |  |  |  |  |  |
| Lived comfortably | -0.03 | 0.07 | 0.70 | -0.17 | 0.11 | 0.90 |
| Found it difficult | 0.04 | 0.09 | 0.63 | -0.13 | 0.22 | . |
| Found it very difficult | 0.08 | 0.29 | 0.78 | -0.49 | 0.65 | . |
| Childhood abuse (Ref: No) |  |  |  |  |  |  |
| Yes | 0.02 | 0.11 | 0.85 | -0.20 | 0.24 | 0.85 |
| Outsider growing up (Ref: No) |  |  |  |  |  |  |
| Yes | -0.39 | 0.13 | 0.00 | -0.64 | -0.14 | 0.00 |
| Childhood health (Ref: Good) |  |  |  |  |  |  |
| Excellent | 0.66 | 0.09 | 0.00 | 0.47 | 0.84 | 0.00 |
| Very good | 0.32 | 0.09 | 0.00 | 0.14 | 0.50 | . |
| Fair | 0.48 | 0.21 | 0.02 | 0.07 | 0.89 | . |
| Poor | -0.03 | 0.29 | 0.91 | -0.60 | 0.54 | . |
| Immigration status (Ref: Born in this country) |  |  |  |  |  |  |
| Born in another country | 0.49 | 0.09 | 0.00 | 0.32 | 0.66 | 0.00 |
| Childhood religious service attendance (Ref: Never) |  |  |  |  |  |  |
| At least 1x/week | 0.24 | 0.09 | 0.01 | 0.06 | 0.42 | 0.00 |
| 1-3x/month | -0.01 | 0.10 | 0.95 | -0.20 | 0.19 | . |
| <1x/month | -0.09 | 0.10 | 0.36 | -0.29 | 0.11 | . |
| Gender (Ref: Male) |  |  |  |  |  |  |
| Female | 0.41 | 0.06 | 0.00 | 0.29 | 0.53 | 0.00 |
| Other | -0.25 | 0.43 | 0.56 | -1.09 | 0.59 | . |
| Birth year/current age (Ref: 1998-2005; age 18-24) |  |  |  |  |  |  |
| 1993-1998; age 25-29 | 0.18 | 0.16 | 0.26 | -0.14 | 0.50 | 0.00 |
| 1983-1993; age 30-39 | 0.23 | 0.13 | 0.08 | -0.03 | 0.49 | . |
| 1973-1983; age 40-49 | 0.40 | 0.12 | 0.00 | 0.16 | 0.65 | . |
| 1963-1973; age 50-59 | 0.55 | 0.13 | 0.00 | 0.30 | 0.80 | . |
| 1953-1963; age 60-69 | 0.49 | 0.14 | 0.00 | 0.21 | 0.77 | . |
| 1943-1953; age 70-79 | 0.39 | 0.21 | 0.06 | -0.01 | 0.80 | . |
| 1943 or earlier; age 80 or older | 1.20 | 0.30 | 0.00 | 0.61 | 1.79 | . |
| Mother absence/presence (Ref: Present) |  |  |  |  |  |  |
| Absent | -0.10 | 0.18 | 0.57 | -0.46 | 0.26 | 0.57 |
| Father absence/presence (Ref: Present) |  |  |  |  |  |  |
| Absent | -0.14 | 0.14 | 0.32 | -0.41 | 0.13 | 0.32 |
| Childhood religion (Ref: No religion/atheist/agnostic) |  |  |  |  |  |  |
| Christianity | 0.17 | 0.10 | 0.08 | -0.02 | 0.37 | 0.07 |
| Some other religion | -0.20 | 0.27 | 0.44 | -0.72 | 0.32 | . |
| *Note*. *N* = 6,290. SE, standard error; LCI, lower confidence interval; UCI, upper confidence interval. | | | | | | |

*Table S17d. E-Values and E-Value Limits for the Coefficients Shown in Table S17c (Spain)*

| Characteristic | *E*-value for estimate^a^ | *E*-value for 95% CI^b^ |
| --- | --- | --- |
| Relationship with mother (Ref: Very/somewhat bad) |  |  |
| Very/somewhat good | 1.77 | 1.45 |
| Relationship with father (Ref: Very/somewhat bad) |  |  |
| Very/somewhat good | 1.09 | 1.00 |
| Parent marital status (Ref: Married) |  |  |
| Divorced | 1.36 | 1.00 |
| Never married | 1.27 | 1.00 |
| One or both parents had died | 1.49 | 1.00 |
| Subjective financial status growing up (Ref: Got by) |  |  |
| Lived comfortably | 1.10 | 1.00 |
| Found it difficult | 1.13 | 1.00 |
| Found it very difficult | 1.19 | 1.00 |
| Childhood abuse (Ref: No) |  |  |
| Yes | 1.09 | 1.00 |
| Outsider growing up (Ref: No) |  |  |
| Yes | 1.51 | 1.26 |
| Childhood health (Ref: Good) |  |  |
| Excellent | 1.76 | 1.60 |
| Very good | 1.45 | 1.26 |
| Fair | 1.60 | 1.18 |
| Poor | 1.11 | 1.00 |
| Immigration status (Ref: Born in this country) |  |  |
| Born in another country | 1.61 | 1.45 |
| Childhood religious service attendance (Ref: Never) |  |  |
| At least 1x/week | 1.37 | 1.16 |
| 1-3x/month | 1.04 | 1.00 |
| <1x/month | 1.20 | 1.00 |
| Gender (Ref: Male) |  |  |
| Female | 1.54 | 1.42 |
| Other | 1.38 | 1.00 |
| Birth year/current age (Ref: 1998-2005; age 18-24) |  |  |
| 1993-1998; age 25-29 | 1.31 | 1.00 |
| 1983-1993; age 30-39 | 1.36 | 1.00 |
| 1973-1983; age 40-49 | 1.53 | 1.29 |
| 1963-1973; age 50-59 | 1.67 | 1.43 |
| 1953-1963; age 60-69 | 1.61 | 1.34 |
| 1943-1953; age 70-79 | 1.52 | 1.00 |
| 1943 or earlier; age 80 or older | 2.28 | 1.72 |
| Mother absence/presence (Ref: Present) |  |  |
| Absent | 1.22 | 1.00 |
| Father absence/presence (Ref: Present) |  |  |
| Absent | 1.26 | 1.00 |
| Childhood religion (Ref: No religion/atheist/agnostic) |  |  |
| Christianity | 1.30 | 1.00 |
| Some other religion | 1.33 | 1.00 |

*Note*. CI, confidence interval. ^a^The formula for calculating *E*-values can be found in VanderWeele and Ding (2017) The *E*-value for the effect estimate is the minimum strength of association (on the risk ratio scale) that an unmeasured confounder would need to have with both the predictor and the outcome to entirely explain away the observed association between them, conditional on the measured covariates. ^b^The *E*-value for the limit of the 95% confidence interval closest to the null denote the minimum strength of association (on the risk ratio scale) that an unmeasured confounder would need to have with both the predictor and the outcome to shift the confidence interval to include the null value, conditional on the measured covariates.

*Table S18a. Nationally Representative Descriptive Statistics of the Observed Sample (Sweden)*

| Variable | Proportion | Frequency |
| --- | --- | --- |
| Sociodemographic characteristics |  |  |
| Birth year/current age |  |  |
| 1998-2005; age 18-24 | 0.10 | 1515 |
| 1993-1998; age 25-29 | 0.09 | 1399 |
| 1983-1993; age 30-39 | 0.16 | 2398 |
| 1973-1983; age 40-49 | 0.15 | 2221 |
| 1963-1973; age 50-59 | 0.17 | 2493 |
| 1953-1963; age 60-69 | 0.14 | 2168 |
| 1943-1953; age 70-79 | 0.15 | 2253 |
| 1943 or earlier; 80 or older | 0.04 | 621 |
| Missing | . | . |
| Gender |  |  |
| Male | 0.50 | 7536 |
| Female | 0.50 | 7493 |
| Other | 0.00 | 27 |
| Missing | 0.00 | 12 |
| Marital status |  |  |
| Single, never married | 0.26 | 3854 |
| Married | 0.43 | 6408 |
| Separated | 0.03 | 426 |
| Divorced | 0.05 | 801 |
| Widowed | 0.03 | 433 |
| Domestic partner | 0.20 | 3073 |
| Missing | 0.00 | 72 |
| Employment |  |  |
| Employed for an employer | 0.52 | 7907 |
| Self-employed | 0.08 | 1243 |
| Retired | 0.25 | 3832 |
| Student | 0.09 | 1332 |
| Homemaker | 0.00 | 75 |
| Unemployed and looking for a job | 0.02 | 324 |
| None of these/other | 0.02 | 337 |
| Missing | 0.00 | 18 |
| Education |  |  |
| Up to 8 years | 0.02 | 252 |
| 9-15 years | 0.72 | 10790 |
| 16+ years | 0.27 | 4026 |
| Missing | . | . |
| Religious service attendance |  |  |
| >1x/week | 0.02 | 236 |
| 1x/week | 0.03 | 434 |
| 1-3x/month | 0.03 | 486 |
| A few times a year | 0.26 | 3950 |
| Never | 0.66 | 9918 |
| Missing | 0.00 | 45 |
| Immigration status |  |  |
| Born in this country | 0.92 | 13922 |
| Born in another country | 0.07 | 1052 |
| Missing | 0.01 | 94 |
| Religion |  |  |
| Christianity | 0.55 | 8346 |
| Islam | 0.03 | 470 |
| Hinduism | 0.00 | 22 |
| Buddhism | 0.01 | 110 |
| Judaism | 0.00 | 54 |
| Sikhism | 0.00 | 4 |
| Baha'i | 0.00 | 6 |
| Jainism | . | . |
| Shinto | 0.00 | 0 |
| Taoism | 0.00 | 4 |
| Confucianism | . | . |
| Primal, Animist, or Folk Religion | 0.01 | 83 |
| Spiritism | . | . |
| African-derived | . | . |
| Chinese | . | . |
| Some other religion | 0.01 | 198 |
| No religion/atheist/agnostic | 0.38 | 5697 |
| Missing | 0.00 | 74 |
| Race/ethnicity |  |  |
| No data | . | . |
| Childhood factors |  |  |
| Relationship with mother |  |  |
| Very good | 0.58 | 8743 |
| Somewhat good | 0.30 | 4513 |
| Somewhat bad | 0.08 | 1194 |
| Very bad | 0.02 | 372 |
| Not applicable | 0.01 | 216 |
| Missing | 0.00 | 30 |
| Relationship with father |  |  |
| Very good | 0.47 | 7134 |
| Somewhat good | 0.32 | 4885 |
| Somewhat bad | 0.11 | 1588 |
| Very bad | 0.05 | 725 |
| Not applicable | 0.05 | 720 |
| Missing | 0.00 | 16 |
| Parent marital status |  |  |
| Married | 0.72 | 10887 |
| Divorced | 0.13 | 1927 |
| Never married | 0.12 | 1747 |
| One or both parents had died | 0.02 | 362 |
| Missing | 0.01 | 145 |
| Subjective financial status growing up |  |  |
| Lived comfortably | 0.39 | 5951 |
| Got by | 0.51 | 7717 |
| Found it difficult | 0.08 | 1238 |
| Found it very difficult | 0.01 | 140 |
| Missing | 0.00 | 22 |
| Childhood abuse |  |  |
| Yes | 0.15 | 2288 |
| No | 0.85 | 12735 |
| Missing | 0.00 | 45 |
| Outsider growing up |  |  |
| Yes | 0.12 | 1867 |
| No | 0.86 | 13034 |
| Not applicable | 0.01 | 139 |
| Missing | 0.00 | 29 |
| Childhood health |  |  |
| Excellent | 0.38 | 5733 |
| Very good | 0.34 | 5124 |
| Good | 0.18 | 2669 |
| Fair | 0.07 | 1108 |
| Poor | 0.03 | 397 |
| Missing | 0.00 | 38 |
| Childhood religious service attendance |  |  |
| At least 1x/week | 0.06 | 955 |
| 1-3x/month | 0.09 | 1362 |
| <1x/month | 0.41 | 6224 |
| Never | 0.43 | 6472 |
| Missing | 0.00 | 54 |
| Childhood religion |  |  |
| Christianity | 0.70 | 10617 |
| Islam | 0.03 | 462 |
| Hinduism | 0.00 | 16 |
| Buddhism | 0.00 | 41 |
| Judaism | 0.00 | 51 |
| Sikhism | 0.00 | 9 |
| Baha'i | 0.00 | 3 |
| Jainism | . | . |
| Shinto | 0.00 | 1 |
| Taoism | . | . |
| Confucianism | 0.00 | 4 |
| Primal, Animist, or Folk Religion | 0.00 | 31 |
| Spiritism | . | . |
| African-derived | . | . |
| Chinese | . | . |
| Some other religion | 0.00 | 69 |
| No religion/atheist/agnostic | 0.25 | 3738 |
| Missing | 0.00 | 26 |

*Note*. *N* = 15,068.

*Table S18b. Variations Across Sociodemographic Characteristics (Sweden)*

| Characteristic | Mean | SE | LCI | UCI | Global *p*-value |
| --- | --- | --- | --- | --- | --- |
| Age group |  |  |  |  |  |
| 18-24 | 7.37 | 0.07 | 7.24 | 7.50 | 0.00 |
| 25-29 | 7.82 | 0.07 | 7.69 | 7.95 | . |
| 30-39 | 7.98 | 0.05 | 7.89 | 8.07 | . |
| 40-49 | 8.14 | 0.05 | 8.04 | 8.24 | . |
| 50-59 | 8.21 | 0.04 | 8.13 | 8.30 | . |
| 60-69 | 8.32 | 0.04 | 8.23 | 8.40 | . |
| 70-79 | 8.50 | 0.04 | 8.42 | 8.58 | . |
| 80 or older | 8.49 | 0.08 | 8.34 | 8.65 | . |
| Gender |  |  |  |  |  |
| Male | 7.77 | 0.03 | 7.72 | 7.83 | 0.00 |
| Female | 8.46 | 0.02 | 8.41 | 8.50 | . |
| Other | 8.01 | 0.54 | 6.92 | 9.11 | . |
| Marital status |  |  |  |  |  |
| Single, never married | 7.18 | 0.04 | 7.10 | 7.27 | 0.00 |
| Married | 8.51 | 0.02 | 8.46 | 8.56 | . |
| Separated | 8.23 | 0.10 | 8.03 | 8.44 | . |
| Divorced | 8.46 | 0.06 | 8.34 | 8.58 | . |
| Widowed | 8.57 | 0.09 | 8.39 | 8.74 | . |
| Domestic partner | 8.29 | 0.03 | 8.22 | 8.35 | . |
| Employment |  |  |  |  |  |
| Employed for an employer | 8.06 | 0.03 | 8.01 | 8.11 | 0.00 |
| Self-employed | 8.27 | 0.07 | 8.13 | 8.42 | . |
| Retired | 8.45 | 0.03 | 8.39 | 8.51 | . |
| Student | 7.47 | 0.07 | 7.33 | 7.60 | . |
| Homemaker | 8.65 | 0.19 | 8.28 | 9.02 | . |
| Unemployed and looking for a job | 7.53 | 0.14 | 7.26 | 7.80 | . |
| None of these/other | 7.85 | 0.15 | 7.55 | 8.16 | . |
| Education |  |  |  |  |  |
| Up to 8 years | 8.14 | 0.15 | 7.83 | 8.44 | 0.08 |
| 9-15 years | 8.09 | 0.02 | 8.04 | 8.13 | . |
| 16+ years | 8.18 | 0.03 | 8.11 | 8.24 | . |
| Religious service attendance |  |  |  |  |  |
| >1x/week | 8.61 | 0.15 | 8.32 | 8.91 | 0.00 |
| 1x/week | 7.86 | 0.13 | 7.60 | 8.12 | . |
| 1-3x/month | 7.87 | 0.10 | 7.68 | 8.06 | . |
| A few times a year | 8.37 | 0.03 | 8.30 | 8.43 | . |
| Never | 8.02 | 0.02 | 7.98 | 8.07 | . |
| Immigration status |  |  |  |  |  |
| Born in this country | 8.11 | 0.02 | 8.08 | 8.15 | 0.86 |
| Born in another country | 8.10 | 0.08 | 7.95 | 8.25 | . |
| Religion |  |  |  |  |  |
| Christianity | 8.28 | 0.02 | 8.24 | 8.33 | 0.00 |
| Islam | 7.87 | 0.15 | 7.57 | 8.16 | . |
| Hinduism | 7.72 | 0.59 | 6.35 | 9.10 | . |
| Buddhism | 7.79 | 0.27 | 7.24 | 8.33 | . |
| Judaism | 8.21 | 0.39 | 7.40 | 9.03 | . |
| Sikhism | 7.45 | 0.49 | -73.88 | 88.77 | . |
| Baha'i | 8.22 | 1.31 | -3.11 | 19.56 | . |
| Jainism | . | . | . | . | . |
| Shinto | 7.00 | . | . | . | . |
| Taoism | 8.11 | 1.25 | -198.29 | 214.50 | . |
| Confucianism | . | . | . | . | . |
| Primal, Animist, or Folk Religion | 7.72 | 0.31 | 7.09 | 8.34 | . |
| Spiritism | . | . | . | . | . |
| African-derived | . | . | . | . | . |
| Chinese | . | . | . | . | . |
| Some other religion | 8.07 | 0.22 | 7.64 | 8.51 | . |
| No religion/atheist/agnostic | 7.90 | 0.03 | 7.84 | 7.95 | . |
| Race/ethnicity |  |  |  |  |  |
| No data | . | . | . | . | . |

*Note*. *N* = 15,068. SE, standard error; LCI, lower confidence interval; UCI, upper confidence interval.

*Table S18c. Variations Across Childhood Predictors (Sweden)*

| Characteristic | Coef. | SE | Prob. | LCI | UCI | Global *p*-value |
| --- | --- | --- | --- | --- | --- | --- |
| Relationship with mother (Ref: Very/somewhat bad) |  |  |  |  |  |  |
| Very/somewhat good | 0.15 | 0.07 | 0.03 | 0.01 | 0.28 | 0.03 |
| Relationship with father (Ref: Very/somewhat bad) |  |  |  |  |  |  |
| Very/somewhat good | 0.10 | 0.06 | 0.11 | -0.02 | 0.21 | 0.11 |
| Parent marital status (Ref: Married) |  |  |  |  |  |  |
| Divorced | 0.18 | 0.06 | 0.00 | 0.06 | 0.30 | 0.04 |
| Never married | 0.04 | 0.06 | 0.58 | -0.09 | 0.16 | . |
| One or both parents had died | 0.02 | 0.13 | 0.85 | -0.22 | 0.27 | . |
| Subjective financial status growing up (Ref: Got by) |  |  |  |  |  |  |
| Lived comfortably | 0.18 | 0.04 | 0.00 | 0.10 | 0.25 | 0.00 |
| Found it difficult | 0.03 | 0.07 | 0.64 | -0.11 | 0.18 | . |
| Found it very difficult | 0.27 | 0.20 | 0.19 | -0.13 | 0.67 | . |
| Childhood abuse (Ref: No) |  |  |  |  |  |  |
| Yes | 0.08 | 0.06 | 0.18 | -0.04 | 0.19 | 0.18 |
| Outsider growing up (Ref: No) |  |  |  |  |  |  |
| Yes | -0.28 | 0.07 | 0.00 | -0.42 | -0.13 | 0.00 |
| Childhood health (Ref: Good) |  |  |  |  |  |  |
| Excellent | 0.48 | 0.06 | 0.00 | 0.37 | 0.59 | 0.00 |
| Very good | 0.13 | 0.06 | 0.02 | 0.02 | 0.23 | . |
| Fair | 0.01 | 0.08 | 0.94 | -0.15 | 0.17 | . |
| Poor | 0.04 | 0.14 | 0.78 | -0.24 | 0.32 | . |
| Immigration status (Ref: Born in this country) |  |  |  |  |  |  |
| Born in another country | 0.03 | 0.08 | 0.67 | -0.12 | 0.18 | 0.67 |
| Childhood religious service attendance (Ref: Never) |  |  |  |  |  |  |
| At least 1x/week | 0.20 | 0.08 | 0.01 | 0.04 | 0.35 | 0.05 |
| 1-3x/month | 0.06 | 0.07 | 0.37 | -0.07 | 0.19 | . |
| <1x/month | 0.08 | 0.04 | 0.06 | 0.00 | 0.15 | . |
| Gender (Ref: Male) |  |  |  |  |  |  |
| Female | 0.74 | 0.04 | 0.00 | 0.66 | 0.81 | 0.00 |
| Other | 0.96 | 0.51 | 0.06 | -0.04 | 1.97 | . |
| Birth year/current age (Ref: 1998-2005; age 18-24) |  |  |  |  |  |  |
| 1993-1998; age 25-29 | 0.53 | 0.09 | 0.00 | 0.35 | 0.71 | 0.00 |
| 1983-1993; age 30-39 | 0.63 | 0.08 | 0.00 | 0.47 | 0.79 | . |
| 1973-1983; age 40-49 | 0.79 | 0.08 | 0.00 | 0.63 | 0.96 | . |
| 1963-1973; age 50-59 | 0.81 | 0.08 | 0.00 | 0.65 | 0.97 | . |
| 1953-1963; age 60-69 | 0.87 | 0.08 | 0.00 | 0.71 | 1.04 | . |
| 1943-1953; age 70-79 | 1.05 | 0.08 | 0.00 | 0.89 | 1.21 | . |
| 1943 or earlier; age 80 or older | 1.07 | 0.11 | 0.00 | 0.85 | 1.28 | . |
| Mother absence/presence (Ref: Present) |  |  |  |  |  |  |
| Absent | -0.04 | 0.12 | 0.73 | -0.29 | 0.20 | 0.73 |
| Father absence/presence (Ref: Present) |  |  |  |  |  |  |
| Absent | 0.09 | 0.09 | 0.31 | -0.09 | 0.27 | 0.31 |
| Childhood religion (Ref: No religion/atheist/agnostic) |  |  |  |  |  |  |
| Christianity | 0.15 | 0.05 | 0.00 | 0.06 | 0.25 | 0.00 |
| Some other religion | 0.22 | 0.12 | 0.07 | -0.02 | 0.46 | . |
| *Note*. *N* = 15,068. SE, standard error; LCI, lower confidence interval; UCI, upper confidence interval. | | | | | | |

*Table S18c. E-Values and E-Value Limits for the Coefficients Shown in Table S18c (Sweden)*

| Characteristic | *E*-value for estimate^a^ | *E*-value for 95% CI^b^ |
| --- | --- | --- |
| Relationship with mother (Ref: Very/somewhat bad) |  |  |
| Very/somewhat good | 1.27 | 1.06 |
| Relationship with father (Ref: Very/somewhat bad) |  |  |
| Very/somewhat good | 1.21 | 1.00 |
| Parent marital status (Ref: Married) |  |  |
| Divorced | 1.31 | 1.16 |
| Never married | 1.12 | 1.00 |
| One or both parents had died | 1.09 | 1.00 |
| Subjective financial status growing up (Ref: Got by) |  |  |
| Lived comfortably | 1.30 | 1.22 |
| Found it difficult | 1.12 | 1.00 |
| Found it very difficult | 1.40 | 1.00 |
| Childhood abuse (Ref: No) |  |  |
| Yes | 1.18 | 1.00 |
| Outsider growing up (Ref: No) |  |  |
| Yes | 1.41 | 1.26 |
| Childhood health (Ref: Good) |  |  |
| Excellent | 1.61 | 1.51 |
| Very good | 1.25 | 1.08 |
| Fair | 1.05 | 1.00 |
| Poor | 1.13 | 1.00 |
| Immigration status (Ref: Born in this country) |  |  |
| Born in another country | 1.11 | 1.00 |
| Childhood religious service attendance (Ref: Never) |  |  |
| At least 1x/week | 1.33 | 1.14 |
| 1-3x/month | 1.16 | 1.00 |
| <1x/month | 1.18 | 1.00 |
| Gender (Ref: Male) |  |  |
| Female | 1.84 | 1.78 |
| Other | 2.06 | 1.00 |
| Birth year/current age (Ref: 1998-2005; age 18-24) |  |  |
| 1993-1998; age 25-29 | 1.66 | 1.48 |
| 1983-1993; age 30-39 | 1.74 | 1.60 |
| 1973-1983; age 40-49 | 1.90 | 1.74 |
| 1963-1973; age 50-59 | 1.92 | 1.77 |
| 1953-1963; age 60-69 | 1.98 | 1.82 |
| 1943-1953; age 70-79 | 2.14 | 1.99 |
| 1943 or earlier; age 80 or older | 2.16 | 1.96 |
| Mother absence/presence (Ref: Present) |  |  |
| Absent | 1.13 | 1.00 |
| Father absence/presence (Ref: Present) |  |  |
| Absent | 1.21 | 1.00 |
| Childhood religion (Ref: No religion/atheist/agnostic) |  |  |
| Christianity | 1.28 | 1.16 |
| Some other religion | 1.35 | 1.00 |

*Note*. CI, confidence interval. ^a^The formula for calculating *E*-values can be found in VanderWeele and Ding (2017) The *E*-value for the effect estimate is the minimum strength of association (on the risk ratio scale) that an unmeasured confounder would need to have with both the predictor and the outcome to entirely explain away the observed association between them, conditional on the measured covariates. ^b^The *E*-value for the limit of the 95% confidence interval closest to the null denote the minimum strength of association (on the risk ratio scale) that an unmeasured confounder would need to have with both the predictor and the outcome to shift the confidence interval to include the null value, conditional on the measured covariates.

*Table S19a. Nationally Representative Descriptive Statistics of the Observed Sample (Tanzania)*

| Variable | Proportion | Frequency |
| --- | --- | --- |
| Sociodemographic characteristics |  |  |
| Birth year/current age |  |  |
| 1998-2005; age 18-24 | 0.25 | 2284 |
| 1993-1998; age 25-29 | 0.15 | 1349 |
| 1983-1993; age 30-39 | 0.23 | 2060 |
| 1973-1983; age 40-49 | 0.17 | 1503 |
| 1963-1973; age 50-59 | 0.10 | 912 |
| 1953-1963; age 60-69 | 0.06 | 575 |
| 1943-1953; age 70-79 | 0.03 | 297 |
| 1943 or earlier; 80 or older | 0.01 | 93 |
| Missing | 0.00 | 2 |
| Gender |  |  |
| Male | 0.47 | 4299 |
| Female | 0.53 | 4776 |
| Other | . | . |
| Missing | . | . |
| Marital status |  |  |
| Single, never married | 0.25 | 2260 |
| Married | 0.61 | 5577 |
| Separated | 0.04 | 404 |
| Divorced | 0.01 | 103 |
| Widowed | 0.05 | 450 |
| Domestic partner | 0.03 | 275 |
| Missing | 0.00 | 7 |
| Employment |  |  |
| Employed for an employer | 0.06 | 513 |
| Self-employed | 0.51 | 4625 |
| Retired | 0.02 | 139 |
| Student | 0.04 | 319 |
| Homemaker | 0.20 | 1796 |
| Unemployed and looking for a job | 0.16 | 1491 |
| None of these/other | 0.02 | 186 |
| Missing | 0.00 | 6 |
| Education |  |  |
| Up to 8 years | 0.74 | 6699 |
| 9-15 years | 0.25 | 2252 |
| 16+ years | 0.01 | 122 |
| Missing | 0.00 | 2 |
| Religious service attendance |  |  |
| >1x/week | 0.29 | 2622 |
| 1x/week | 0.47 | 4268 |
| 1-3x/month | 0.12 | 1082 |
| A few times a year | 0.09 | 814 |
| Never | 0.03 | 288 |
| Missing | 0.00 | 1 |
| Immigration status |  |  |
| Born in this country | 1.00 | 9048 |
| Born in another country | 0.00 | 25 |
| Missing | 0.00 | 1 |
| Religion |  |  |
| Christianity | 0.62 | 5647 |
| Islam | 0.35 | 3189 |
| Hinduism | . | . |
| Buddhism | . | . |
| Judaism | . | . |
| Sikhism | . | . |
| Baha'i | . | . |
| Jainism | . | . |
| Shinto | . | . |
| Taoism | 0.00 | 1 |
| Confucianism | . | . |
| Primal, Animist, or Folk Religion | 0.00 | 12 |
| Spiritism | . | . |
| African-derived | . | . |
| Chinese | . | . |
| Some other religion | . | . |
| No religion/atheist/agnostic | 0.02 | 216 |
| Missing | 0.00 | 10 |
| Race/ethnicity |  |  |
| African | 1.00 | 9060 |
| Indian | 0.00 | 3 |
| Arab | 0.00 | 11 |
| Other | . | . |
| Missing | 0.00 | 2 |
| Childhood factors |  |  |
| Relationship with mother |  |  |
| Very good | 0.85 | 7739 |
| Somewhat good | 0.09 | 796 |
| Somewhat bad | 0.01 | 84 |
| Very bad | 0.01 | 84 |
| Not applicable | 0.03 | 303 |
| Missing | 0.01 | 70 |
| Relationship with father |  |  |
| Very good | 0.75 | 6831 |
| Somewhat good | 0.12 | 1101 |
| Somewhat bad | 0.02 | 203 |
| Very bad | 0.03 | 247 |
| Not applicable | 0.06 | 550 |
| Missing | 0.02 | 142 |
| Parent marital status |  |  |
| Married | 0.76 | 6929 |
| Divorced | 0.07 | 678 |
| Never married | 0.08 | 751 |
| One or both parents had died | 0.03 | 313 |
| Missing | 0.04 | 404 |
| Subjective financial status growing up |  |  |
| Lived comfortably | 0.29 | 2611 |
| Got by | 0.32 | 2909 |
| Found it difficult | 0.30 | 2679 |
| Found it very difficult | 0.09 | 814 |
| Missing | 0.01 | 61 |
| Childhood abuse |  |  |
| Yes | 0.08 | 716 |
| No | 0.92 | 8328 |
| Missing | 0.00 | 32 |
| Outsider growing up |  |  |
| Yes | 0.08 | 734 |
| No | 0.92 | 8320 |
| Not applicable | 0.00 | 4 |
| Missing | 0.00 | 17 |
| Childhood health |  |  |
| Excellent | 0.27 | 2406 |
| Very good | 0.22 | 2036 |
| Good | 0.32 | 2946 |
| Fair | 0.13 | 1177 |
| Poor | 0.05 | 456 |
| Missing | 0.01 | 54 |
| Childhood religious service attendance |  |  |
| At least 1x/week | 0.61 | 5580 |
| 1-3x/month | 0.26 | 2383 |
| <1x/month | 0.04 | 333 |
| Never | 0.07 | 595 |
| Missing | 0.02 | 184 |
| Childhood religion |  |  |
| Christianity | 0.62 | 5651 |
| Islam | 0.34 | 3060 |
| Hinduism | . | . |
| Buddhism | . | . |
| Judaism | . | . |
| Sikhism | . | . |
| Baha'i | 0.00 | 1 |
| Jainism | . | . |
| Shinto | . | . |
| Taoism | . | . |
| Confucianism | . | . |
| Primal, Animist, or Folk Religion | 0.00 | 11 |
| Spiritism | . | . |
| African-derived | . | . |
| Chinese | . | . |
| Some other religion | . | . |
| No religion/atheist/agnostic | 0.04 | 345 |
| Missing | 0.00 | 7 |

*Note*. *N* = 9,075.

*Table S19b. Variations Across Sociodemographic Characteristics (Tanzania)*

| Characteristic | Mean | SE | LCI | UCI | Global *p*-value |
| --- | --- | --- | --- | --- | --- |
| Age group |  |  |  |  |  |
| 18-24 | 8.92 | 0.07 | 8.77 | 9.06 | 0.01 |
| 25-29 | 8.81 | 0.08 | 8.64 | 8.97 | . |
| 30-39 | 8.59 | 0.08 | 8.44 | 8.74 | . |
| 40-49 | 8.63 | 0.09 | 8.46 | 8.80 | . |
| 50-59 | 8.69 | 0.11 | 8.48 | 8.91 | . |
| 60-69 | 8.66 | 0.14 | 8.38 | 8.95 | . |
| 70-79 | 8.49 | 0.29 | 7.91 | 9.07 | . |
| 80 or older | 8.29 | 0.45 | 7.38 | 9.21 | . |
| Gender |  |  |  |  |  |
| Male | 8.73 | 0.06 | 8.61 | 8.85 | 0.75 |
| Female | 8.71 | 0.06 | 8.59 | 8.83 | . |
| Other | . | . | . | . | . |
| Marital status |  |  |  |  |  |
| Single, never married | 8.86 | 0.07 | 8.72 | 9.00 | 0.14 |
| Married | 8.68 | 0.06 | 8.56 | 8.81 | . |
| Separated | 8.65 | 0.15 | 8.35 | 8.95 | . |
| Divorced | 8.87 | 0.23 | 8.40 | 9.34 | . |
| Widowed | 8.59 | 0.19 | 8.23 | 8.96 | . |
| Domestic partner | 8.55 | 0.17 | 8.21 | 8.88 | . |
| Employment |  |  |  |  |  |
| Employed for an employer | 8.94 | 0.10 | 8.74 | 9.13 | 0.03 |
| Self-employed | 8.71 | 0.07 | 8.58 | 8.84 | . |
| Retired | 8.82 | 0.31 | 8.21 | 9.43 | . |
| Student | 9.12 | 0.13 | 8.86 | 9.37 | . |
| Homemaker | 8.62 | 0.09 | 8.44 | 8.81 | . |
| Unemployed and looking for a job | 8.67 | 0.08 | 8.51 | 8.84 | . |
| None of these/other | 8.82 | 0.21 | 8.39 | 9.25 | . |
| Education |  |  |  |  |  |
| Up to 8 years | 8.63 | 0.06 | 8.50 | 8.75 | 0.00 |
| 9-15 years | 8.98 | 0.06 | 8.86 | 9.09 | . |
| 16+ years | 9.00 | 0.13 | 8.75 | 9.26 | . |
| Religious service attendance |  |  |  |  |  |
| >1x/week | 8.88 | 0.07 | 8.75 | 9.00 | 0.00 |
| 1x/week | 8.74 | 0.07 | 8.60 | 8.88 | . |
| 1-3x/month | 8.39 | 0.12 | 8.16 | 8.62 | . |
| A few times a year | 8.62 | 0.11 | 8.41 | 8.84 | . |
| Never | 8.52 | 0.19 | 8.15 | 8.89 | . |
| Immigration status |  |  |  |  |  |
| Born in this country | 8.72 | 0.05 | 8.61 | 8.83 | 0.89 |
| Born in another country | 8.79 | 0.43 | 7.81 | 9.76 | . |
| Religion |  |  |  |  |  |
| Christianity | 8.78 | 0.06 | 8.66 | 8.89 | 0.00 |
| Islam | 8.65 | 0.10 | 8.46 | 8.84 | . |
| Hinduism | . | . | . | . | . |
| Buddhism | . | . | . | . | . |
| Judaism | . | . | . | . | . |
| Sikhism | . | . | . | . | . |
| Baha'i | . | . | . | . | . |
| Jainism | . | . | . | . | . |
| Shinto | . | . | . | . | . |
| Taoism | 10.00 | . | . | . | . |
| Confucianism | . | . | . | . | . |
| Primal, Animist, or Folk Religion | 9.46 | 0.33 | 8.06 | 10.86 | . |
| Spiritism | . | . | . | . | . |
| African-derived | . | . | . | . | . |
| Chinese | . | . | . | . | . |
| Some other religion | . | . | . | . | . |
| No religion/atheist/agnostic | 8.26 | 0.31 | 7.64 | 8.89 | . |
| Race/ethnicity |  |  |  |  |  |
| African | 8.72 | 0.05 | 8.61 | 8.83 | 0.24 |
| Indian | 8.01 | . | . | . | . |
| Arab | 7.72 | 0.85 | 4.07 | 11.38 | . |
| Other | . | . | . | . | . |

*Note*. *N* = 9,075. SE, standard error; LCI, lower confidence interval; UCI, upper confidence interval.

*Table S19c. Variations Across Childhood Predictors (Tanzania)*

| Characteristic | Coef. | SE | Prob. | LCI | UCI | Global *p*-value |
| --- | --- | --- | --- | --- | --- | --- |
| Relationship with mother (Ref: Very/somewhat bad) |  |  |  |  |  |  |
| Very/somewhat good | -0.04 | 0.20 | 0.86 | -0.42 | 0.35 | 0.86 |
| Relationship with father (Ref: Very/somewhat bad) |  |  |  |  |  |  |
| Very/somewhat good | -0.19 | 0.14 | 0.17 | -0.46 | 0.08 | 0.17 |
| Parent marital status (Ref: Married) |  |  |  |  |  |  |
| Divorced | -0.36 | 0.14 | 0.01 | -0.63 | -0.09 | 0.07 |
| Never married | 0.08 | 0.12 | 0.51 | -0.16 | 0.32 | . |
| One or both parents had died | 0.04 | 0.15 | 0.79 | -0.25 | 0.33 | . |
| Subjective financial status growing up (Ref: Got by) |  |  |  |  |  |  |
| Lived comfortably | 0.01 | 0.08 | 0.89 | -0.15 | 0.17 | 0.89 |
| Found it difficult | 0.06 | 0.08 | 0.47 | -0.11 | 0.23 | . |
| Found it very difficult | 0.04 | 0.12 | 0.74 | -0.20 | 0.28 | . |
| Childhood abuse (Ref: No) |  |  |  |  |  |  |
| Yes | -0.21 | 0.13 | 0.10 | -0.47 | 0.04 | 0.10 |
| Outsider growing up (Ref: No) |  |  |  |  |  |  |
| Yes | -0.18 | 0.14 | 0.19 | -0.45 | 0.09 | 0.19 |
| Childhood health (Ref: Good) |  |  |  |  |  |  |
| Excellent | 0.17 | 0.08 | 0.04 | 0.01 | 0.33 | 0.01 |
| Very good | 0.16 | 0.09 | 0.09 | -0.03 | 0.35 | . |
| Fair | 0.11 | 0.09 | 0.24 | -0.07 | 0.28 | . |
| Poor | -0.44 | 0.17 | 0.01 | -0.78 | -0.09 | . |
| Immigration status (Ref: Born in this country) |  |  |  |  |  |  |
| Born in another country | 0.05 | 0.39 | 0.91 | -0.73 | 0.82 | 0.91 |
| Childhood religious service attendance (Ref: Never) |  |  |  |  |  |  |
| At least 1x/week | 0.21 | 0.21 | 0.32 | -0.20 | 0.62 | 0.11 |
| 1-3x/month | 0.01 | 0.22 | 0.96 | -0.42 | 0.44 | . |
| <1x/month | 0.09 | 0.23 | 0.69 | -0.37 | 0.55 | . |
| Gender (Ref: Male) |  |  |  |  |  |  |
| Female | -0.04 | 0.07 | 0.55 | -0.17 | 0.09 | 0.55 |
| Other | . | . | . | . | . | . |
| Birth year/current age (Ref: 1998-2005; age 18-24) |  |  |  |  |  |  |
| 1993-1998; age 25-29 | -0.14 | 0.10 | 0.16 | -0.33 | 0.05 | 0.01 |
| 1983-1993; age 30-39 | -0.33 | 0.09 | 0.00 | -0.51 | -0.16 | . |
| 1973-1983; age 40-49 | -0.30 | 0.09 | 0.00 | -0.48 | -0.12 | . |
| 1963-1973; age 50-59 | -0.22 | 0.12 | 0.07 | -0.45 | 0.02 | . |
| 1953-1963; age 60-69 | -0.27 | 0.15 | 0.07 | -0.56 | 0.03 | . |
| 1943-1953; age 70-79 | -0.42 | 0.29 | 0.15 | -1.00 | 0.16 | . |
| 1943 or earlier; age 80 or older | -0.63 | 0.43 | 0.15 | -1.49 | 0.22 | . |
| Mother absence/presence (Ref: Present) |  |  |  |  |  |  |
| Absent | 0.01 | 0.19 | 0.97 | -0.37 | 0.38 | 0.97 |
| Father absence/presence (Ref: Present) |  |  |  |  |  |  |
| Absent | -0.05 | 0.17 | 0.77 | -0.39 | 0.29 | 0.77 |
| Childhood religion (Ref: No religion/atheist/agnostic) |  |  |  |  |  |  |
| Christianity | 0.59 | 0.31 | 0.06 | -0.02 | 1.19 | 0.09 |
| Islam | 0.51 | 0.33 | 0.12 | -0.13 | 1.15 | . |
| Some other religion | 1.01 | 0.48 | 0.04 | 0.07 | 1.96 | . |
| Race/ethnicity (Ref: Ethnic plurality) |  |  |  |  |  |  |
| Ethnic minority | -0.90 | 0.58 | 0.12 | -2.04 | 0.24 | 0.12 |
| *Note*. *N* = 9,075. SE, standard error; LCI, lower confidence interval; UCI, upper confidence interval. | | | | | | |

*Table S19d. E-Values and E-Value Limits for the Coefficients Shown in Table S19c (Tanzania)*

| Characteristic | *E*-value for estimate^a^ | *E*-value for 95% CI^b^ |
| --- | --- | --- |
| Relationship with mother (Ref: Very/somewhat bad) |  |  |
| Very/somewhat good | 1.12 | 1.00 |
| Relationship with father (Ref: Very/somewhat bad) |  |  |
| Very/somewhat good | 1.31 | 1.00 |
| Parent marital status (Ref: Married) |  |  |
| Divorced | 1.48 | 1.21 |
| Never married | 1.18 | 1.00 |
| One or both parents had died | 1.12 | 1.00 |
| Subjective financial status growing up (Ref: Got by) |  |  |
| Lived comfortably | 1.06 | 1.00 |
| Found it difficult | 1.16 | 1.00 |
| Found it very difficult | 1.12 | 1.00 |
| Childhood abuse (Ref: No) |  |  |
| Yes | 1.34 | 1.00 |
| Outsider growing up (Ref: No) |  |  |
| Yes | 1.30 | 1.00 |
| Childhood health (Ref: Good) |  |  |
| Excellent | 1.29 | 1.06 |
| Very good | 1.28 | 1.00 |
| Fair | 1.22 | 1.00 |
| Poor | 1.55 | 1.21 |
| Immigration status (Ref: Born in this country) |  |  |
| Born in another country | 1.13 | 1.00 |
| Childhood religious service attendance (Ref: Never) |  |  |
| At least 1x/week | 1.33 | 1.00 |
| 1-3x/month | 1.06 | 1.00 |
| <1x/month | 1.20 | 1.00 |
| Gender (Ref: Male) |  |  |
| Female | 1.12 | 1.00 |
| Other | . | . |
| Birth year/current age (Ref: 1998-2005; age 18-24) |  |  |
| 1993-1998; age 25-29 | 1.25 | 1.00 |
| 1983-1993; age 30-39 | 1.45 | 1.28 |
| 1973-1983; age 40-49 | 1.42 | 1.24 |
| 1963-1973; age 50-59 | 1.34 | 1.00 |
| 1953-1963; age 60-69 | 1.39 | 1.00 |
| 1943-1953; age 70-79 | 1.53 | 1.00 |
| 1943 or earlier; age 80 or older | 1.73 | 1.00 |
| Mother absence/presence (Ref: Present) |  |  |
| Absent | 1.05 | 1.00 |
| Father absence/presence (Ref: Present) |  |  |
| Absent | 1.14 | 1.00 |
| Childhood religion (Ref: No religion/atheist/agnostic) |  |  |
| Christianity | 1.68 | 1.00 |
| Islam | 1.61 | 1.00 |
| Some other religion | 2.07 | 1.18 |
| Race/ethnicity (Ref: Ethnic plurality) |  |  |
| Ethnic minority | 1.97 | 1.00 |

*Note*. CI, confidence interval. ^a^The formula for calculating *E*-values can be found in VanderWeele and Ding (2017) The *E*-value for the effect estimate is the minimum strength of association (on the risk ratio scale) that an unmeasured confounder would need to have with both the predictor and the outcome to entirely explain away the observed association between them, conditional on the measured covariates. ^b^The *E*-value for the limit of the 95% confidence interval closest to the null denote the minimum strength of association (on the risk ratio scale) that an unmeasured confounder would need to have with both the predictor and the outcome to shift the confidence interval to include the null value, conditional on the measured covariates.

*Table S20a. Nationally Representative Descriptive Statistics of the Observed Sample (Turkey)*

| Variable | Proportion | Frequency |
| --- | --- | --- |
| Sociodemographic characteristics |  |  |
| Birth year/current age |  |  |
| 1998-2005; age 18-24 | 0.15 | 222 |
| 1993-1998; age 25-29 | 0.10 | 152 |
| 1983-1993; age 30-39 | 0.21 | 315 |
| 1973-1983; age 40-49 | 0.21 | 312 |
| 1963-1973; age 50-59 | 0.15 | 225 |
| 1953-1963; age 60-69 | 0.11 | 164 |
| 1943-1953; age 70-79 | 0.04 | 65 |
| 1943 or earlier; 80 or older | 0.01 | 18 |
| Missing | . | . |
| Gender |  |  |
| Male | 0.51 | 754 |
| Female | 0.49 | 719 |
| Other | . | . |
| Missing | . | . |
| Marital status |  |  |
| Single, never married | 0.26 | 379 |
| Married | 0.64 | 936 |
| Separated | 0.01 | 13 |
| Divorced | 0.04 | 64 |
| Widowed | 0.04 | 64 |
| Domestic partner | . | . |
| Missing | 0.01 | 17 |
| Employment |  |  |
| Employed for an employer | 0.28 | 413 |
| Self-employed | 0.17 | 255 |
| Retired | 0.14 | 205 |
| Student | 0.07 | 107 |
| Homemaker | 0.24 | 347 |
| Unemployed and looking for a job | 0.06 | 87 |
| None of these/other | 0.04 | 59 |
| Missing | . | . |
| Education |  |  |
| Up to 8 years | 0.30 | 436 |
| 9-15 years | 0.48 | 711 |
| 16+ years | 0.22 | 326 |
| Missing | . | . |
| Religious service attendance |  |  |
| >1x/week | 0.33 | 493 |
| 1x/week | 0.18 | 271 |
| 1-3x/month | 0.12 | 174 |
| A few times a year | 0.17 | 255 |
| Never | 0.19 | 274 |
| Missing | 0.00 | 6 |
| Immigration status |  |  |
| Born in this country | 0.96 | 1415 |
| Born in another country | 0.04 | 58 |
| Missing | . | . |
| Religion |  |  |
| Christianity | 0.00 | 2 |
| Islam | 0.94 | 1381 |
| Hinduism | . | . |
| Buddhism | 0.00 | 1 |
| Judaism | 0.00 | 1 |
| Sikhism | 0.00 | 1 |
| Baha'i | . | . |
| Jainism | . | . |
| Shinto | . | . |
| Taoism | . | . |
| Confucianism | . | . |
| Primal, Animist, or Folk Religion | 0.00 | 1 |
| Spiritism | . | . |
| African-derived | . | . |
| Chinese | . | . |
| Some other religion | 0.00 | 1 |
| No religion/atheist/agnostic | 0.04 | 66 |
| Missing | 0.01 | 19 |
| Race/ethnicity |  |  |
| Turkish | 0.70 | 1030 |
| Kurdish/Zaza | 0.17 | 252 |
| Arab | 0.03 | 51 |
| Laz | 0.02 | 25 |
| Circassian | 0.01 | 19 |
| Bosnian | 0.00 | 5 |
| Armenian | 0.00 | 1 |
| Georgian | 0.00 | 4 |
| Uyghur | 0.00 | 1 |
| Jewish | . | . |
| Albanian | 0.01 | 8 |
| Greek | 0.00 | 1 |
| Azeri | 0.01 | 9 |
| Other | 0.04 | 58 |
| Missing | 0.01 | 9 |
| Childhood factors |  |  |
| Relationship with mother |  |  |
| Very good | 0.66 | 970 |
| Somewhat good | 0.27 | 401 |
| Somewhat bad | 0.03 | 48 |
| Very bad | 0.02 | 26 |
| Not applicable | 0.01 | 21 |
| Missing | 0.00 | 7 |
| Relationship with father |  |  |
| Very good | 0.54 | 795 |
| Somewhat good | 0.29 | 425 |
| Somewhat bad | 0.05 | 73 |
| Very bad | 0.06 | 95 |
| Not applicable | 0.04 | 60 |
| Missing | 0.02 | 25 |
| Parent marital status |  |  |
| Married | 0.90 | 1325 |
| Divorced | 0.04 | 57 |
| Never married | 0.00 | 7 |
| One or both parents had died | 0.04 | 61 |
| Missing | 0.02 | 23 |
| Subjective financial status growing up |  |  |
| Lived comfortably | 0.34 | 498 |
| Got by | 0.44 | 647 |
| Found it difficult | 0.15 | 218 |
| Found it very difficult | 0.07 | 108 |
| Missing | 0.00 | 2 |
| Childhood abuse |  |  |
| Yes | 0.11 | 158 |
| No | 0.88 | 1290 |
| Missing | 0.02 | 25 |
| Outsider growing up |  |  |
| Yes | 0.11 | 157 |
| No | 0.89 | 1306 |
| Not applicable | 0.00 | 5 |
| Missing | 0.00 | 5 |
| Childhood health |  |  |
| Excellent | 0.26 | 377 |
| Very good | 0.28 | 410 |
| Good | 0.28 | 419 |
| Fair | 0.15 | 220 |
| Poor | 0.03 | 47 |
| Missing | 0.00 | 1 |
| Childhood religious service attendance |  |  |
| At least 1x/week | 0.41 | 609 |
| 1-3x/month | 0.16 | 238 |
| <1x/month | 0.15 | 225 |
| Never | 0.26 | 383 |
| Missing | 0.01 | 18 |
| Childhood religion |  |  |
| Christianity | 0.00 | 1 |
| Islam | 0.98 | 1439 |
| Hinduism | . | . |
| Buddhism | . | . |
| Judaism | 0.00 | 1 |
| Sikhism | . | . |
| Baha'i | . | . |
| Jainism | . | . |
| Shinto | . | . |
| Taoism | . | . |
| Confucianism | . | . |
| Primal, Animist, or Folk Religion | . | . |
| Spiritism | . | . |
| African-derived | . | . |
| Chinese | . | . |
| Some other religion | . | . |
| No religion/atheist/agnostic | 0.01 | 13 |
| Missing | 0.01 | 19 |

*Note*. *N* = 1,473.

*Table S20b. Variations Across Sociodemographic Characteristics (Turkey)*

| Characteristic | Mean | SE | LCI | UCI | Global *p*-value |
| --- | --- | --- | --- | --- | --- |
| Age group |  |  |  |  |  |
| 18-24 | 7.56 | 0.17 | 7.22 | 7.90 | 0.06 |
| 25-29 | 8.12 | 0.25 | 7.63 | 8.62 | . |
| 30-39 | 7.47 | 0.19 | 7.10 | 7.85 | . |
| 40-49 | 8.13 | 0.15 | 7.83 | 8.43 | . |
| 50-59 | 8.20 | 0.22 | 7.76 | 8.64 | . |
| 60-69 | 7.94 | 0.29 | 7.36 | 8.52 | . |
| 70-79 | 8.13 | 0.29 | 7.49 | 8.76 | . |
| 80 or older | 7.53 | 0.64 | -98.31 | 113.37 | . |
| Gender |  |  |  |  |  |
| Male | 7.64 | 0.11 | 7.43 | 7.86 | 0.00 |
| Female | 8.14 | 0.12 | 7.90 | 8.38 | . |
| Other | . | . | . | . | . |
| Marital status |  |  |  |  |  |
| Single, never married | 7.54 | 0.14 | 7.27 | 7.81 | 0.11 |
| Married | 8.01 | 0.11 | 7.80 | 8.23 | . |
| Separated | 8.34 | 0.57 | 6.71 | 9.97 | . |
| Divorced | 7.90 | 0.51 | 6.88 | 8.93 | . |
| Widowed | 7.94 | 0.51 | 6.88 | 9.00 | . |
| Domestic partner | . | . | . | . | . |
| Employment |  |  |  |  |  |
| Employed for an employer | 7.85 | 0.14 | 7.58 | 8.13 | 0.00 |
| Self-employed | 8.07 | 0.18 | 7.72 | 8.41 | . |
| Retired | 8.38 | 0.21 | 7.97 | 8.80 | . |
| Student | 7.34 | 0.22 | 6.90 | 7.77 | . |
| Homemaker | 7.98 | 0.20 | 7.60 | 8.37 | . |
| Unemployed and looking for a job | 7.37 | 0.40 | 6.57 | 8.17 | . |
| None of these/other | 6.76 | 0.44 | 5.88 | 7.65 | . |
| Education |  |  |  |  |  |
| Up to 8 years | 7.90 | 0.20 | 7.51 | 8.28 | 1.00 |
| 9-15 years | 7.88 | 0.12 | 7.65 | 8.11 | . |
| 16+ years | 7.88 | 0.10 | 7.68 | 8.08 | . |
| Religious service attendance |  |  |  |  |  |
| >1x/week | 8.28 | 0.13 | 8.03 | 8.53 | 0.00 |
| 1x/week | 7.86 | 0.23 | 7.41 | 8.30 | . |
| 1-3x/month | 7.96 | 0.23 | 7.50 | 8.42 | . |
| A few times a year | 7.62 | 0.18 | 7.27 | 7.97 | . |
| Never | 7.41 | 0.22 | 6.98 | 7.83 | . |
| Immigration status |  |  |  |  |  |
| Born in this country | 7.89 | 0.09 | 7.72 | 8.06 | 0.88 |
| Born in another country | 7.81 | 0.43 | 6.93 | 8.68 | . |
| Religion |  |  |  |  |  |
| Christianity | 7.60 | 0.26 | . | . | 0.00 |
| Islam | 7.90 | 0.09 | 7.72 | 8.07 | . |
| Hinduism | . | . | . | . | . |
| Buddhism | 10.00 | . | . | . | . |
| Judaism | 10.00 | . | . | . | . |
| Sikhism | 8.00 | . | . | . | . |
| Baha'i | . | . | . | . | . |
| Jainism | . | . | . | . | . |
| Shinto | . | . | . | . | . |
| Taoism | . | . | . | . | . |
| Confucianism | . | . | . | . | . |
| Primal, Animist, or Folk Religion | 10.00 | . | . | . | . |
| Spiritism | . | . | . | . | . |
| African-derived | . | . | . | . | . |
| Chinese | . | . | . | . | . |
| Some other religion | 7.79 | . | . | . | . |
| No religion/atheist/agnostic | 7.53 | 0.28 | 6.97 | 8.10 | . |
| Race/ethnicity |  |  |  |  |  |
| Turkish | 7.93 | 0.09 | 7.75 | 8.12 | 0.00 |
| Kurdish/Zaza | 7.57 | 0.25 | 7.08 | 8.06 | . |
| Arab | 8.51 | 0.30 | 7.90 | 9.12 | . |
| Laz | 7.68 | 0.67 | 6.23 | 9.13 | . |
| Circassian | 8.44 | 0.43 | 7.44 | 9.45 | . |
| Bosnian | 9.03 | 0.83 | 1.85 | 16.21 | . |
| Armenian | 10.00 | . | . | . | . |
| Georgian | 9.76 | 0.25 | -31.41 | 50.93 | . |
| Uyghur | 10.00 | . | . | . | . |
| Jewish | . | . | . | . | . |
| Albanian | 4.91 | 0.83 | -131.97 | 141.80 | . |
| Greek | 7.16 | . | . | . | . |
| Azeri | 6.56 | 0.25 | 5.75 | 7.38 | . |
| Other | 8.04 | 0.37 | 7.30 | 8.79 | . |

*Note*. *N* = 1,473. SE, standard error; LCI, lower confidence interval; UCI, upper confidence interval.

*Table S20c. Variations Across Childhood Predictors (Turkey)*

| Characteristic | Coef. | SE | Prob. | LCI | UCI | Global *p*-value |
| --- | --- | --- | --- | --- | --- | --- |
| Relationship with mother (Ref: Very/somewhat bad) |  |  |  |  |  |  |
| Very/somewhat good | -0.42 | 0.33 | 0.21 | -1.07 | 0.24 | 0.21 |
| Relationship with father (Ref: Very/somewhat bad) |  |  |  |  |  |  |
| Very/somewhat good | 0.30 | 0.30 | 0.32 | -0.29 | 0.90 | 0.32 |
| Parent marital status (Ref: Married) |  |  |  |  |  |  |
| Divorced | 0.63 | 0.41 | 0.12 | -0.16 | 1.43 | 0.06 |
| Never married | -1.23 | 1.17 | 0.29 | -3.52 | 1.06 | . |
| One or both parents had died | 0.81 | 0.39 | 0.04 | 0.04 | 1.57 | . |
| Subjective financial status growing up (Ref: Got by) |  |  |  |  |  |  |
| Lived comfortably | 0.30 | 0.18 | 0.09 | -0.05 | 0.65 | 0.34 |
| Found it difficult | -0.07 | 0.27 | 0.80 | -0.59 | 0.46 | . |
| Found it very difficult | -0.08 | 0.46 | 0.86 | -0.98 | 0.81 | . |
| Childhood abuse (Ref: No) |  |  |  |  |  |  |
| Yes | -0.13 | 0.27 | 0.64 | -0.67 | 0.41 | 0.64 |
| Outsider growing up (Ref: No) |  |  |  |  |  |  |
| Yes | 0.04 | 0.28 | 0.87 | -0.50 | 0.59 | 0.87 |
| Childhood health (Ref: Good) |  |  |  |  |  |  |
| Excellent | -0.11 | 0.22 | 0.60 | -0.54 | 0.32 | 0.13 |
| Very good | -0.15 | 0.22 | 0.49 | -0.59 | 0.28 | . |
| Fair | -0.53 | 0.27 | 0.06 | -1.06 | 0.01 | . |
| Poor | -1.43 | 0.69 | 0.04 | -2.78 | -0.08 | . |
| Immigration status (Ref: Born in this country) |  |  |  |  |  |  |
| Born in another country | 0.00 | 0.54 | 1.00 | -1.05 | 1.06 | 1.00 |
| Childhood religious service attendance (Ref: Never) |  |  |  |  |  |  |
| At least 1x/week | 0.64 | 0.24 | 0.01 | 0.16 | 1.12 | 0.07 |
| 1-3x/month | 0.33 | 0.29 | 0.26 | -0.24 | 0.90 | . |
| <1x/month | 0.36 | 0.25 | 0.14 | -0.12 | 0.84 | . |
| Gender (Ref: Male) |  |  |  |  |  |  |
| Female | 0.57 | 0.18 | 0.00 | 0.22 | 0.92 | 0.00 |
| Other | . | . | . | . | . | . |
| Birth year/current age (Ref: 1998-2005; age 18-24) |  |  |  |  |  |  |
| 1993-1998; age 25-29 | 0.61 | 0.29 | 0.03 | 0.05 | 1.17 | 0.02 |
| 1983-1993; age 30-39 | 0.01 | 0.25 | 0.96 | -0.48 | 0.50 | . |
| 1973-1983; age 40-49 | 0.69 | 0.23 | 0.00 | 0.24 | 1.15 | . |
| 1963-1973; age 50-59 | 0.75 | 0.29 | 0.01 | 0.18 | 1.32 | . |
| 1953-1963; age 60-69 | 0.51 | 0.33 | 0.12 | -0.13 | 1.15 | . |
| 1943-1953; age 70-79 | 0.70 | 0.55 | 0.20 | -0.38 | 1.78 | . |
| 1943 or earlier; age 80 or older | -0.02 | 1.02 | 0.98 | -2.02 | 1.98 | . |
| Mother absence/presence (Ref: Present) |  |  |  |  |  |  |
| Absent | -0.81 | 0.49 | 0.10 | -1.78 | 0.16 | 0.10 |
| Father absence/presence (Ref: Present) |  |  |  |  |  |  |
| Absent | -0.36 | 0.46 | 0.44 | -1.27 | 0.55 | 0.44 |
| Childhood religion (Ref: Islam) |  |  |  |  |  |  |
| Some other religion | -0.14 | 0.66 | 0.83 | -1.43 | 1.15 | 0.83 |
| Race/ethnicity (Ref: Ethnic plurality) |  |  |  |  |  |  |
| Ethnic minority | 0.01 | 0.20 | 0.95 | -0.39 | 0.41 | 0.95 |
| *Note*. *N* = 1,473. SE, standard error; LCI, lower confidence interval; UCI, upper confidence interval. | | | | | | |

*Table S20d. E-Values and E-Value Limits for the Coefficients Shown in Table S20c (Turkey)*

| Characteristic | *E*-value for estimate^a^ | *E*-value for 95% CI^b^ |
| --- | --- | --- |
| Relationship with mother (Ref: Very/somewhat bad) |  |  |
| Very/somewhat good | 1.55 | 1.00 |
| Relationship with father (Ref: Very/somewhat bad) |  |  |
| Very/somewhat good | 1.44 | 1.00 |
| Parent marital status (Ref: Married) |  |  |
| Divorced | 1.76 | 1.00 |
| Never married | 2.34 | 1.00 |
| One or both parents had died | 1.92 | 1.13 |
| Subjective financial status growing up (Ref: Got by) |  |  |
| Lived comfortably | 1.44 | 1.00 |
| Found it difficult | 1.17 | 1.00 |
| Found it very difficult | 1.19 | 1.00 |
| Childhood abuse (Ref: No) |  |  |
| Yes | 1.25 | 1.00 |
| Outsider growing up (Ref: No) |  |  |
| Yes | 1.14 | 1.00 |
| Childhood health (Ref: Good) |  |  |
| Excellent | 1.23 | 1.00 |
| Very good | 1.28 | 1.00 |
| Fair | 1.66 | 1.00 |
| Poor | 2.56 | 1.20 |
| Immigration status (Ref: Born in this country) |  |  |
| Born in another country | 1.03 | 1.00 |
| Childhood religious service attendance (Ref: Never) |  |  |
| At least 1x/week | 1.76 | 1.29 |
| 1-3x/month | 1.47 | 1.00 |
| <1x/month | 1.50 | 1.00 |
| Gender (Ref: Male) |  |  |
| Female | 1.69 | 1.35 |
| Other | . | . |
| Birth year/current age (Ref: 1998-2005; age 18-24) |  |  |
| 1993-1998; age 25-29 | 1.73 | 1.14 |
| 1983-1993; age 30-39 | 1.07 | 1.00 |
| 1973-1983; age 40-49 | 1.81 | 1.37 |
| 1963-1973; age 50-59 | 1.87 | 1.32 |
| 1953-1963; age 60-69 | 1.64 | 1.00 |
| 1943-1953; age 70-79 | 1.82 | 1.00 |
| 1943 or earlier; age 80 or older | 1.09 | 1.00 |
| Mother absence/presence (Ref: Present) |  |  |
| Absent | 1.93 | 1.00 |
| Father absence/presence (Ref: Present) |  |  |
| Absent | 1.50 | 1.00 |
| Childhood religion (Ref: Islam) |  |  |
| Some other religion | 1.27 | 1.00 |
| Race/ethnicity (Ref: Ethnic plurality) |  |  |
| Ethnic minority | 1.07 | 1.00 |

*Note*. CI, confidence interval. ^a^The formula for calculating *E*-values can be found in VanderWeele and Ding (2017) The *E*-value for the effect estimate is the minimum strength of association (on the risk ratio scale) that an unmeasured confounder would need to have with both the predictor and the outcome to entirely explain away the observed association between them, conditional on the measured covariates. ^b^The *E*-value for the limit of the 95% confidence interval closest to the null denote the minimum strength of association (on the risk ratio scale) that an unmeasured confounder would need to have with both the predictor and the outcome to shift the confidence interval to include the null value, conditional on the measured covariates.

*Table S21a. Nationally Representative Descriptive Statistics of the Observed Sample (United Kingdom)*

| Variable | Proportion | Frequency |
| --- | --- | --- |
| Sociodemographic characteristics |  |  |
| Birth year/current age |  |  |
| 1998-2005; age 18-24 | 0.09 | 490 |
| 1993-1998; age 25-29 | 0.07 | 391 |
| 1983-1993; age 30-39 | 0.18 | 946 |
| 1973-1983; age 40-49 | 0.15 | 827 |
| 1963-1973; age 50-59 | 0.18 | 949 |
| 1953-1963; age 60-69 | 0.17 | 889 |
| 1943-1953; age 70-79 | 0.13 | 711 |
| 1943 or earlier; 80 or older | 0.03 | 163 |
| Missing | 0.00 | 1 |
| Gender |  |  |
| Male | 0.48 | 2557 |
| Female | 0.52 | 2789 |
| Other | 0.00 | 14 |
| Missing | 0.00 | 9 |
| Marital status |  |  |
| Single, never married | 0.27 | 1456 |
| Married | 0.47 | 2510 |
| Separated | 0.02 | 114 |
| Divorced | 0.08 | 435 |
| Widowed | 0.05 | 294 |
| Domestic partner | 0.10 | 512 |
| Missing | 0.01 | 48 |
| Employment |  |  |
| Employed for an employer | 0.52 | 2798 |
| Self-employed | 0.09 | 469 |
| Retired | 0.24 | 1262 |
| Student | 0.04 | 229 |
| Homemaker | 0.03 | 184 |
| Unemployed and looking for a job | 0.04 | 215 |
| None of these/other | 0.04 | 201 |
| Missing | 0.00 | 11 |
| Education |  |  |
| Up to 8 years | 0.24 | 1314 |
| 9-15 years | 0.39 | 2072 |
| 16+ years | 0.37 | 1974 |
| Missing | 0.00 | 8 |
| Religious service attendance |  |  |
| >1x/week | 0.05 | 291 |
| 1x/week | 0.09 | 499 |
| 1-3x/month | 0.05 | 293 |
| A few times a year | 0.22 | 1165 |
| Never | 0.58 | 3110 |
| Missing | 0.00 | 10 |
| Immigration status |  |  |
| Born in this country | 0.87 | 4659 |
| Born in another country | 0.13 | 682 |
| Missing | 0.00 | 27 |
| Religion |  |  |
| Christianity | 0.51 | 2750 |
| Islam | 0.04 | 218 |
| Hinduism | 0.01 | 61 |
| Buddhism | 0.01 | 30 |
| Judaism | 0.01 | 44 |
| Sikhism | 0.01 | 29 |
| Baha'i | 0.00 | 6 |
| Jainism | 0.00 | 4 |
| Shinto | . | . |
| Taoism | 0.00 | 4 |
| Confucianism | 0.00 | 2 |
| Primal, Animist, or Folk Religion | 0.01 | 36 |
| Spiritism | . | . |
| African-derived | . | . |
| Chinese | . | . |
| Some other religion | 0.01 | 61 |
| No religion/atheist/agnostic | 0.39 | 2099 |
| Missing | 0.00 | 25 |
| Race/ethnicity |  |  |
| Asian | 0.08 | 426 |
| Black | 0.03 | 152 |
| White | 0.87 | 4647 |
| Other | 0.02 | 96 |
| Missing | 0.01 | 47 |
| Childhood factors |  |  |
| Relationship with mother |  |  |
| Very good | 0.64 | 3435 |
| Somewhat good | 0.25 | 1338 |
| Somewhat bad | 0.06 | 325 |
| Very bad | 0.03 | 150 |
| Not applicable | 0.02 | 92 |
| Missing | 0.01 | 27 |
| Relationship with father |  |  |
| Very good | 0.54 | 2907 |
| Somewhat good | 0.26 | 1383 |
| Somewhat bad | 0.08 | 407 |
| Very bad | 0.06 | 321 |
| Not applicable | 0.06 | 321 |
| Missing | 0.01 | 29 |
| Parent marital status |  |  |
| Married | 0.81 | 4343 |
| Divorced | 0.09 | 481 |
| Never married | 0.06 | 315 |
| One or both parents had died | 0.03 | 154 |
| Missing | 0.01 | 75 |
| Subjective financial status growing up |  |  |
| Lived comfortably | 0.48 | 2552 |
| Got by | 0.36 | 1933 |
| Found it difficult | 0.12 | 632 |
| Found it very difficult | 0.04 | 230 |
| Missing | 0.00 | 22 |
| Childhood abuse |  |  |
| Yes | 0.16 | 864 |
| No | 0.83 | 4455 |
| Missing | 0.01 | 49 |
| Outsider growing up |  |  |
| Yes | 0.19 | 1017 |
| No | 0.80 | 4308 |
| Not applicable | 0.01 | 32 |
| Missing | 0.00 | 12 |
| Childhood health |  |  |
| Excellent | 0.40 | 2154 |
| Very good | 0.32 | 1736 |
| Good | 0.19 | 995 |
| Fair | 0.06 | 332 |
| Poor | 0.02 | 130 |
| Missing | 0.00 | 20 |
| Childhood religious service attendance |  |  |
| At least 1x/week | 0.32 | 1732 |
| 1-3x/month | 0.14 | 733 |
| <1x/month | 0.17 | 903 |
| Never | 0.37 | 1972 |
| Missing | 0.01 | 28 |
| Childhood religion |  |  |
| Christianity | 0.64 | 3461 |
| Islam | 0.04 | 230 |
| Hinduism | 0.02 | 88 |
| Buddhism | 0.00 | 15 |
| Judaism | 0.01 | 59 |
| Sikhism | 0.01 | 30 |
| Baha'i | 0.00 | 5 |
| Jainism | 0.00 | 0 |
| Shinto | . | . |
| Taoism | 0.00 | 2 |
| Confucianism | 0.00 | 3 |
| Primal, Animist, or Folk Religion | 0.00 | 22 |
| Spiritism | . | . |
| African-derived | . | . |
| Chinese | . | . |
| Some other religion | 0.00 | 24 |
| No religion/atheist/agnostic | 0.26 | 1409 |
| Missing | 0.00 | 21 |

*Note*. *N* = 5,368.

*Table S21b. Variations Across Sociodemographic Characteristics (United Kingdom)*

| Characteristic | Mean | SE | LCI | UCI | Global *p*-value |
| --- | --- | --- | --- | --- | --- |
| Age group |  |  |  |  |  |
| 18-24 | 7.60 | 0.16 | 7.29 | 7.91 | 0.00 |
| 25-29 | 8.13 | 0.13 | 7.87 | 8.38 | . |
| 30-39 | 7.90 | 0.09 | 7.72 | 8.07 | . |
| 40-49 | 8.18 | 0.09 | 8.01 | 8.36 | . |
| 50-59 | 8.27 | 0.08 | 8.12 | 8.42 | . |
| 60-69 | 8.17 | 0.10 | 7.97 | 8.36 | . |
| 70-79 | 8.45 | 0.10 | 8.24 | 8.65 | . |
| 80 or older | 8.58 | 0.24 | 8.11 | 9.06 | . |
| Gender |  |  |  |  |  |
| Male | 7.82 | 0.06 | 7.71 | 7.94 | 0.00 |
| Female | 8.42 | 0.05 | 8.33 | 8.52 | . |
| Other | 7.64 | 0.89 | 5.52 | 9.77 | . |
| Marital status |  |  |  |  |  |
| Single, never married | 7.50 | 0.09 | 7.33 | 7.67 | 0.00 |
| Married | 8.39 | 0.05 | 8.29 | 8.48 | . |
| Separated | 8.10 | 0.18 | 7.74 | 8.46 | . |
| Divorced | 7.99 | 0.15 | 7.69 | 8.29 | . |
| Widowed | 8.65 | 0.15 | 8.35 | 8.95 | . |
| Domestic partner | 8.55 | 0.09 | 8.37 | 8.74 | . |
| Employment |  |  |  |  |  |
| Employed for an employer | 8.11 | 0.05 | 8.01 | 8.20 | 0.00 |
| Self-employed | 7.99 | 0.13 | 7.73 | 8.25 | . |
| Retired | 8.45 | 0.08 | 8.30 | 8.60 | . |
| Student | 7.64 | 0.19 | 7.26 | 8.03 | . |
| Homemaker | 8.49 | 0.17 | 8.17 | 8.82 | . |
| Unemployed and looking for a job | 7.40 | 0.31 | 6.79 | 8.00 | . |
| None of these/other | 7.97 | 0.19 | 7.59 | 8.35 | . |
| Education |  |  |  |  |  |
| Up to 8 years | 8.19 | 0.11 | 7.97 | 8.41 | 0.85 |
| 9-15 years | 8.12 | 0.05 | 8.02 | 8.22 | . |
| 16+ years | 8.12 | 0.05 | 8.03 | 8.21 | . |
| Religious service attendance |  |  |  |  |  |
| >1x/week | 8.49 | 0.13 | 8.24 | 8.75 | 0.00 |
| 1x/week | 8.19 | 0.11 | 7.98 | 8.39 | . |
| 1-3x/month | 8.18 | 0.17 | 7.86 | 8.51 | . |
| A few times a year | 8.31 | 0.07 | 8.17 | 8.45 | . |
| Never | 8.02 | 0.05 | 7.92 | 8.13 | . |
| Immigration status |  |  |  |  |  |
| Born in this country | 8.15 | 0.04 | 8.07 | 8.23 | 0.37 |
| Born in another country | 8.05 | 0.10 | 7.85 | 8.25 | . |
| Religion |  |  |  |  |  |
| Christianity | 8.40 | 0.05 | 8.30 | 8.50 | 0.00 |
| Islam | 7.64 | 0.21 | 7.23 | 8.05 | . |
| Hinduism | 7.44 | 0.31 | 6.82 | 8.06 | . |
| Buddhism | 7.98 | 0.37 | 7.22 | 8.74 | . |
| Judaism | 8.49 | 0.35 | 7.77 | 9.21 | . |
| Sikhism | 6.19 | 0.39 | 5.32 | 7.05 | . |
| Baha'i | 8.41 | 0.43 | -62.02 | 78.84 | . |
| Jainism | 5.07 | . | . | . | . |
| Shinto | . | . | . | . | . |
| Taoism | 8.90 | 0.11 | -9.83 | 27.63 | . |
| Confucianism | 10.00 | . | . | . | . |
| Primal, Animist, or Folk Religion | 8.03 | 0.41 | 7.16 | 8.90 | . |
| Spiritism | . | . | . | . | . |
| African-derived | . | . | . | . | . |
| Chinese | . | . | . | . | . |
| Some other religion | 8.38 | 0.21 | 7.95 | 8.80 | . |
| No religion/atheist/agnostic | 7.88 | 0.06 | 7.76 | 8.00 | . |
| Race/ethnicity |  |  |  |  |  |
| Asian | 7.53 | 0.16 | 7.22 | 7.84 | 0.00 |
| Black | 8.34 | 0.17 | 8.01 | 8.67 | . |
| White | 8.19 | 0.04 | 8.11 | 8.27 | . |
| Other | 7.79 | 0.35 | 7.10 | 8.48 | . |

*Note*. *N* = 5,368. SE, standard error; LCI, lower confidence interval; UCI, upper confidence interval.

*Table S21c. Variations Across Childhood Predictors (United Kingdom)*

| Characteristic | Coef. | SE | Prob. | LCI | UCI | Global *p*-value |
| --- | --- | --- | --- | --- | --- | --- |
| Relationship with mother (Ref: Very/somewhat bad) |  |  |  |  |  |  |
| Very/somewhat good | 0.19 | 0.14 | 0.16 | -0.07 | 0.46 | 0.16 |
| Relationship with father (Ref: Very/somewhat bad) |  |  |  |  |  |  |
| Very/somewhat good | 0.36 | 0.12 | 0.00 | 0.13 | 0.60 | 0.00 |
| Parent marital status (Ref: Married) |  |  |  |  |  |  |
| Divorced | 0.05 | 0.13 | 0.70 | -0.21 | 0.32 | 0.38 |
| Never married | 0.21 | 0.20 | 0.28 | -0.17 | 0.60 | . |
| One or both parents had died | -0.26 | 0.22 | 0.23 | -0.69 | 0.17 | . |
| Subjective financial status growing up (Ref: Got by) |  |  |  |  |  |  |
| Lived comfortably | 0.03 | 0.08 | 0.73 | -0.13 | 0.18 | 0.19 |
| Found it difficult | -0.18 | 0.13 | 0.16 | -0.43 | 0.07 | . |
| Found it very difficult | -0.42 | 0.27 | 0.12 | -0.95 | 0.11 | . |
| Childhood abuse (Ref: No) |  |  |  |  |  |  |
| Yes | 0.10 | 0.10 | 0.32 | -0.10 | 0.31 | 0.32 |
| Outsider growing up (Ref: No) |  |  |  |  |  |  |
| Yes | -0.05 | 0.11 | 0.67 | -0.26 | 0.17 | 0.67 |
| Childhood health (Ref: Good) |  |  |  |  |  |  |
| Excellent | 0.37 | 0.11 | 0.00 | 0.16 | 0.58 | 0.00 |
| Very good | 0.26 | 0.10 | 0.01 | 0.05 | 0.46 | . |
| Fair | -0.25 | 0.19 | 0.19 | -0.62 | 0.12 | . |
| Poor | -0.23 | 0.35 | 0.51 | -0.91 | 0.45 | . |
| Immigration status (Ref: Born in this country) |  |  |  |  |  |  |
| Born in another country | 0.02 | 0.11 | 0.89 | -0.20 | 0.23 | 0.89 |
| Childhood religious service attendance (Ref: Never) |  |  |  |  |  |  |
| At least 1x/week | 0.18 | 0.10 | 0.09 | -0.03 | 0.38 | 0.32 |
| 1-3x/month | 0.15 | 0.11 | 0.17 | -0.07 | 0.38 | . |
| <1x/month | 0.05 | 0.12 | 0.65 | -0.17 | 0.28 | . |
| Gender (Ref: Male) |  |  |  |  |  |  |
| Female | 0.62 | 0.07 | 0.00 | 0.47 | 0.76 | 0.00 |
| Other | 0.41 | 0.81 | 0.61 | -1.17 | 2.00 | . |
| Birth year/current age (Ref: 1998-2005; age 18-24) |  |  |  |  |  |  |
| 1993-1998; age 25-29 | 0.47 | 0.19 | 0.02 | 0.09 | 0.85 | 0.00 |
| 1983-1993; age 30-39 | 0.27 | 0.17 | 0.11 | -0.06 | 0.61 | . |
| 1973-1983; age 40-49 | 0.55 | 0.17 | 0.00 | 0.21 | 0.88 | . |
| 1963-1973; age 50-59 | 0.57 | 0.17 | 0.00 | 0.24 | 0.91 | . |
| 1953-1963; age 60-69 | 0.42 | 0.18 | 0.02 | 0.07 | 0.78 | . |
| 1943-1953; age 70-79 | 0.67 | 0.18 | 0.00 | 0.31 | 1.03 | . |
| 1943 or earlier; age 80 or older | 0.75 | 0.26 | 0.00 | 0.25 | 1.26 | . |
| Mother absence/presence (Ref: Present) |  |  |  |  |  |  |
| Absent | -0.03 | 0.21 | 0.88 | -0.43 | 0.37 | 0.88 |
| Father absence/presence (Ref: Present) |  |  |  |  |  |  |
| Absent | 0.14 | 0.17 | 0.39 | -0.18 | 0.47 | 0.39 |
| Childhood religion (Ref: No religion/atheist/agnostic) |  |  |  |  |  |  |
| Christianity | 0.27 | 0.10 | 0.01 | 0.08 | 0.46 | 0.01 |
| Islam | -0.15 | 0.24 | 0.53 | -0.63 | 0.32 | . |
| Some other religion | 0.03 | 0.19 | 0.89 | -0.34 | 0.39 | . |
| Race/ethnicity (Ref: Ethnic plurality) |  |  |  |  |  |  |
| Ethnic minority | -0.25 | 0.14 | 0.08 | -0.54 | 0.03 | 0.08 |
| *Note*. *N* = 5,368. SE, standard error; LCI, lower confidence interval; UCI, upper confidence interval. | | | | | | |

*Table S21d. E-Values and E-Value Limits for the Coefficients Shown in Table S21c (United Kingdom)*

| Characteristic | *E*-value for estimate^a^ | *E*-value for 95% CI^b^ |
| --- | --- | --- |
| Relationship with mother (Ref: Very/somewhat bad) |  |  |
| Very/somewhat good | 1.32 | 1.00 |
| Relationship with father (Ref: Very/somewhat bad) |  |  |
| Very/somewhat good | 1.49 | 1.25 |
| Parent marital status (Ref: Married) |  |  |
| Divorced | 1.15 | 1.00 |
| Never married | 1.34 | 1.00 |
| One or both parents had died | 1.40 | 1.00 |
| Subjective financial status growing up (Ref: Got by) |  |  |
| Lived comfortably | 1.10 | 1.00 |
| Found it difficult | 1.31 | 1.00 |
| Found it very difficult | 1.55 | 1.00 |
| Childhood abuse (Ref: No) |  |  |
| Yes | 1.22 | 1.00 |
| Outsider growing up (Ref: No) |  |  |
| Yes | 1.14 | 1.00 |
| Childhood health (Ref: Good) |  |  |
| Excellent | 1.50 | 1.29 |
| Very good | 1.39 | 1.15 |
| Fair | 1.38 | 1.00 |
| Poor | 1.36 | 1.00 |
| Immigration status (Ref: Born in this country) |  |  |
| Born in another country | 1.07 | 1.00 |
| Childhood religious service attendance (Ref: Never) |  |  |
| At least 1x/week | 1.31 | 1.00 |
| 1-3x/month | 1.28 | 1.00 |
| <1x/month | 1.15 | 1.00 |
| Gender (Ref: Male) |  |  |
| Female | 1.73 | 1.60 |
| Other | 1.54 | 1.00 |
| Birth year/current age (Ref: 1998-2005; age 18-24) |  |  |
| 1993-1998; age 25-29 | 1.59 | 1.20 |
| 1983-1993; age 30-39 | 1.41 | 1.00 |
| 1973-1983; age 40-49 | 1.67 | 1.34 |
| 1963-1973; age 50-59 | 1.69 | 1.37 |
| 1953-1963; age 60-69 | 1.55 | 1.18 |
| 1943-1953; age 70-79 | 1.78 | 1.44 |
| 1943 or earlier; age 80 or older | 1.86 | 1.39 |
| Mother absence/presence (Ref: Present) |  |  |
| Absent | 1.11 | 1.00 |
| Father absence/presence (Ref: Present) |  |  |
| Absent | 1.27 | 1.00 |
| Childhood religion (Ref: No religion/atheist/agnostic) |  |  |
| Christianity | 1.40 | 1.19 |
| Islam | 1.28 | 1.00 |
| Some other religion | 1.10 | 1.00 |
| Race/ethnicity (Ref: Ethnic plurality) |  |  |
| Ethnic minority | 1.39 | 1.00 |

*Note*. CI, confidence interval. ^a^The formula for calculating *E*-values can be found in VanderWeele and Ding (2017) The *E*-value for the effect estimate is the minimum strength of association (on the risk ratio scale) that an unmeasured confounder would need to have with both the predictor and the outcome to entirely explain away the observed association between them, conditional on the measured covariates. ^b^The *E*-value for the limit of the 95% confidence interval closest to the null denote the minimum strength of association (on the risk ratio scale) that an unmeasured confounder would need to have with both the predictor and the outcome to shift the confidence interval to include the null value, conditional on the measured covariates.

*Table S22a. Nationally Representative Descriptive Statistics of the Observed Sample (United States)*

| Variable | Proportion | Frequency |
| --- | --- | --- |
| Sociodemographic characteristics |  |  |
| Birth year/current age |  |  |
| 1998-2005; age 18-24 | 0.07 | 2682 |
| 1993-1998; age 25-29 | 0.09 | 3540 |
| 1983-1993; age 30-39 | 0.19 | 7284 |
| 1973-1983; age 40-49 | 0.15 | 5649 |
| 1963-1973; age 50-59 | 0.18 | 6745 |
| 1953-1963; age 60-69 | 0.18 | 6832 |
| 1943-1953; age 70-79 | 0.11 | 4054 |
| 1943 or earlier; 80 or older | 0.04 | 1525 |
| Missing | . | . |
| Gender |  |  |
| Male | 0.48 | 18222 |
| Female | 0.51 | 19562 |
| Other | 0.01 | 392 |
| Missing | 0.00 | 136 |
| Marital status |  |  |
| Single, never married | 0.25 | 9431 |
| Married | 0.53 | 20360 |
| Separated | 0.02 | 727 |
| Divorced | 0.09 | 3636 |
| Widowed | 0.05 | 1978 |
| Domestic partner | 0.05 | 1971 |
| Missing | 0.01 | 207 |
| Employment |  |  |
| Employed for an employer | 0.51 | 19502 |
| Self-employed | 0.09 | 3445 |
| Retired | 0.24 | 9016 |
| Student | 0.03 | 1145 |
| Homemaker | 0.05 | 2049 |
| Unemployed and looking for a job | 0.05 | 1777 |
| None of these/other | 0.03 | 1292 |
| Missing | 0.00 | 87 |
| Education |  |  |
| Up to 8 years | 0.01 | 210 |
| 9-15 years | 0.66 | 25322 |
| 16+ years | 0.33 | 12705 |
| Missing | 0.00 | 75 |
| Religious service attendance |  |  |
| >1x/week | 0.07 | 2633 |
| 1x/week | 0.15 | 5887 |
| 1-3x/month | 0.07 | 2819 |
| A few times a year | 0.23 | 8870 |
| Never | 0.47 | 17975 |
| Missing | 0.00 | 128 |
| Immigration status |  |  |
| Born in this country | 0.91 | 34865 |
| Born in another country | 0.08 | 3020 |
| Missing | 0.01 | 427 |
| Religion |  |  |
| Christianity | 0.60 | 22954 |
| Islam | 0.01 | 205 |
| Hinduism | 0.00 | 167 |
| Buddhism | 0.01 | 336 |
| Judaism | 0.02 | 638 |
| Sikhism | 0.00 | 24 |
| Baha'i | 0.00 | 13 |
| Jainism | 0.00 | 18 |
| Shinto | 0.00 | 12 |
| Taoism | 0.00 | 93 |
| Confucianism | 0.00 | 8 |
| Primal, Animist, or Folk Religion | 0.01 | 240 |
| Spiritism | . | . |
| African-derived | . | . |
| Chinese | . | . |
| Some other religion | 0.03 | 1267 |
| No religion/atheist/agnostic | 0.31 | 11870 |
| Missing | 0.01 | 467 |
| Race/ethnicity |  |  |
| White | 0.62 | 23605 |
| Other | 0.03 | 997 |
| Black | 0.12 | 4501 |
| Asian | 0.06 | 2466 |
| Hispanic | 0.18 | 6724 |
| Other | . | . |
| Missing | 0.00 | 20 |
| Childhood factors |  |  |
| Relationship with mother |  |  |
| Very good | 0.54 | 20590 |
| Somewhat good | 0.30 | 11525 |
| Somewhat bad | 0.09 | 3523 |
| Very bad | 0.05 | 1874 |
| Not applicable | 0.02 | 694 |
| Missing | 0.00 | 106 |
| Relationship with father |  |  |
| Very good | 0.40 | 15313 |
| Somewhat good | 0.33 | 12666 |
| Somewhat bad | 0.13 | 4879 |
| Very bad | 0.07 | 2604 |
| Not applicable | 0.07 | 2811 |
| Missing | 0.00 | 38 |
| Parent marital status |  |  |
| Married | 0.72 | 27415 |
| Divorced | 0.17 | 6325 |
| Never married | 0.08 | 3048 |
| One or both parents had died | 0.03 | 1024 |
| Missing | 0.01 | 500 |
| Subjective financial status growing up |  |  |
| Lived comfortably | 0.39 | 15116 |
| Got by | 0.41 | 15682 |
| Found it difficult | 0.13 | 5152 |
| Found it very difficult | 0.06 | 2342 |
| Missing | 0.00 | 19 |
| Childhood abuse |  |  |
| Yes | 0.26 | 10026 |
| No | 0.73 | 28045 |
| Missing | 0.01 | 242 |
| Outsider growing up |  |  |
| Yes | 0.27 | 10185 |
| No | 0.72 | 27714 |
| Not applicable | 0.01 | 305 |
| Missing | 0.00 | 109 |
| Childhood health |  |  |
| Excellent | 0.44 | 16866 |
| Very good | 0.32 | 12108 |
| Good | 0.17 | 6444 |
| Fair | 0.06 | 2303 |
| Poor | 0.01 | 520 |
| Missing | 0.00 | 71 |
| Childhood religious service attendance |  |  |
| At least 1x/week | 0.49 | 18609 |
| 1-3x/month | 0.17 | 6644 |
| <1x/month | 0.15 | 5829 |
| Never | 0.18 | 7085 |
| Missing | 0.00 | 145 |
| Childhood religion |  |  |
| Christianity | 0.79 | 30444 |
| Islam | 0.01 | 220 |
| Hinduism | 0.01 | 203 |
| Buddhism | 0.00 | 172 |
| Judaism | 0.02 | 787 |
| Sikhism | 0.00 | 47 |
| Baha'i | 0.00 | 4 |
| Jainism | 0.00 | 18 |
| Shinto | 0.00 | 6 |
| Taoism | 0.00 | 17 |
| Confucianism | 0.00 | 8 |
| Primal, Animist, or Folk Religion | 0.00 | 67 |
| Spiritism | . | . |
| African-derived | . | . |
| Chinese | . | . |
| Some other religion | 0.01 | 359 |
| No religion/atheist/agnostic | 0.15 | 5845 |
| Missing | 0.00 | 115 |

*Note. N* = 38,312.

*Table S22b. Variations Across Sociodemographic Characteristics (United States)*

| Characteristic | Mean | SE | LCI | UCI | Global *p*-value |
| --- | --- | --- | --- | --- | --- |
| Age group |  |  |  |  |  |
| 18-24 | 7.72 | 0.18 | 7.36 | 8.07 | 0.00 |
| 25-29 | 8.10 | 0.13 | 7.85 | 8.36 | . |
| 30-39 | 8.33 | 0.07 | 8.19 | 8.46 | . |
| 40-49 | 8.60 | 0.05 | 8.50 | 8.70 | . |
| 50-59 | 8.71 | 0.03 | 8.64 | 8.78 | . |
| 60-69 | 8.85 | 0.02 | 8.80 | 8.89 | . |
| 70-79 | 8.95 | 0.03 | 8.90 | 9.01 | . |
| 80 or older | 9.05 | 0.06 | 8.92 | 9.17 | . |
| Gender |  |  |  |  |  |
| Male | 8.32 | 0.04 | 8.24 | 8.39 | 0.00 |
| Female | 8.79 | 0.03 | 8.72 | 8.86 | . |
| Other | 8.08 | 0.29 | 7.52 | 8.65 | . |
| Marital status |  |  |  |  |  |
| Single, never married | 7.78 | 0.08 | 7.63 | 7.93 | 0.00 |
| Married | 8.86 | 0.02 | 8.82 | 8.91 | . |
| Separated | 8.42 | 0.20 | 8.03 | 8.82 | . |
| Divorced | 8.72 | 0.04 | 8.63 | 8.80 | . |
| Widowed | 8.89 | 0.06 | 8.76 | 9.01 | . |
| Domestic partner | 8.54 | 0.12 | 8.30 | 8.78 | . |
| Employment |  |  |  |  |  |
| Employed for an employer | 8.51 | 0.03 | 8.45 | 8.57 | 0.00 |
| Self-employed | 8.62 | 0.09 | 8.43 | 8.80 | . |
| Retired | 8.90 | 0.03 | 8.84 | 8.96 | . |
| Student | 7.54 | 0.23 | 7.09 | 7.99 | . |
| Homemaker | 8.91 | 0.07 | 8.78 | 9.04 | . |
| Unemployed and looking for a job | 7.66 | 0.24 | 7.19 | 8.13 | . |
| None of these/other | 8.33 | 0.21 | 7.91 | 8.74 | . |
| Education |  |  |  |  |  |
| Up to 8 years | 8.38 | 0.43 | 7.49 | 9.26 | 0.80 |
| 9-15 years | 8.55 | 0.04 | 8.48 | 8.63 | . |
| 16+ years | 8.57 | 0.02 | 8.54 | 8.61 | . |
| Religious service attendance |  |  |  |  |  |
| >1x/week | 8.99 | 0.08 | 8.83 | 9.15 | 0.00 |
| 1x/week | 8.93 | 0.04 | 8.86 | 9.01 | . |
| 1-3x/month | 8.71 | 0.09 | 8.54 | 8.88 | . |
| A few times a year | 8.72 | 0.04 | 8.64 | 8.80 | . |
| Never | 8.27 | 0.04 | 8.18 | 8.36 | . |
| Immigration status |  |  |  |  |  |
| Born in this country | 8.58 | 0.03 | 8.52 | 8.63 | 0.06 |
| Born in another country | 8.36 | 0.11 | 8.15 | 8.58 | . |
| Religion |  |  |  |  |  |
| Christianity | 8.80 | 0.02 | 8.75 | 8.85 | 0.00 |
| Islam | 7.88 | 0.46 | 6.98 | 8.79 | . |
| Hinduism | 8.36 | 0.21 | 7.94 | 8.77 | . |
| Buddhism | 8.33 | 0.17 | 8.00 | 8.66 | . |
| Judaism | 8.70 | 0.08 | 8.54 | 8.85 | . |
| Sikhism | 8.59 | 0.55 | 7.39 | 9.78 | . |
| Baha'i | 8.36 | 0.76 | 6.76 | 9.97 | . |
| Jainism | 7.10 | 0.81 | 5.16 | 9.04 | . |
| Shinto | 9.16 | 0.45 | 8.15 | 10.18 | . |
| Taoism | 8.73 | 0.52 | 7.67 | 9.78 | . |
| Confucianism | 9.16 | 0.44 | 7.84 | 10.49 | . |
| Primal, Animist, or Folk Religion | 8.77 | 0.44 | 7.90 | 9.64 | . |
| Spiritism | . | . | . | . | . |
| African-derived | . | . | . | . | . |
| Chinese | . | . | . | . | . |
| Some other religion | 8.53 | 0.12 | 8.29 | 8.76 | . |
| No religion/atheist/agnostic | 8.10 | 0.06 | 7.98 | 8.22 | . |
| Race/ethnicity |  |  |  |  |  |
| White | 8.65 | 0.02 | 8.61 | 8.68 | 0.00 |
| Other | 8.71 | 0.08 | 8.55 | 8.86 | . |
| Black | 8.61 | 0.08 | 8.45 | 8.76 | . |
| Asian | 8.19 | 0.10 | 8.00 | 8.38 | . |
| Hispanic | 8.33 | 0.11 | 8.12 | 8.54 | . |
| Other | . | . | . | . | . |

*Note*. *N* = 38,312. SE, standard error; LCI, lower confidence interval; UCI, upper confidence interval.

*Table S22c. Variations Across Childhood Predictors (United States)*

| Characteristic | Coef. | SE | Prob. | LCI | UCI | Global *p*-value |
| --- | --- | --- | --- | --- | --- | --- |
| Relationship with mother (Ref: Very/somewhat bad) |  |  |  |  |  |  |
| Very/somewhat good | 0.25 | 0.09 | 0.01 | 0.07 | 0.42 | 0.01 |
| Relationship with father (Ref: Very/somewhat bad) |  |  |  |  |  |  |
| Very/somewhat good | 0.26 | 0.08 | 0.00 | 0.11 | 0.41 | 0.00 |
| Parent marital status (Ref: Married) |  |  |  |  |  |  |
| Divorced | 0.20 | 0.07 | 0.00 | 0.07 | 0.34 | 0.03 |
| Never married | 0.10 | 0.17 | 0.57 | -0.24 | 0.43 | . |
| One or both parents had died | 0.20 | 0.19 | 0.30 | -0.18 | 0.57 | . |
| Subjective financial status growing up (Ref: Got by) |  |  |  |  |  |  |
| Lived comfortably | 0.14 | 0.04 | 0.00 | 0.05 | 0.22 | 0.01 |
| Found it difficult | 0.20 | 0.08 | 0.01 | 0.05 | 0.35 | . |
| Found it very difficult | 0.20 | 0.17 | 0.22 | -0.12 | 0.53 | . |
| Childhood abuse (Ref: No) |  |  |  |  |  |  |
| Yes | 0.15 | 0.06 | 0.02 | 0.02 | 0.27 | 0.02 |
| Outsider growing up (Ref: No) |  |  |  |  |  |  |
| Yes | -0.28 | 0.07 | 0.00 | -0.41 | -0.14 | 0.00 |
| Childhood health (Ref: Good) |  |  |  |  |  |  |
| Excellent | 0.49 | 0.07 | 0.00 | 0.34 | 0.63 | 0.00 |
| Very good | 0.21 | 0.08 | 0.01 | 0.05 | 0.36 | . |
| Fair | -0.22 | 0.18 | 0.22 | -0.57 | 0.13 | . |
| Poor | 0.10 | 0.40 | 0.80 | -0.67 | 0.88 | . |
| Immigration status (Ref: Born in this country) |  |  |  |  |  |  |
| Born in another country | -0.20 | 0.11 | 0.06 | -0.40 | 0.01 | 0.06 |
| Childhood religious service attendance (Ref: Never) |  |  |  |  |  |  |
| At least 1x/week | 0.05 | 0.08 | 0.59 | -0.12 | 0.21 | 0.03 |
| 1-3x/month | -0.08 | 0.09 | 0.36 | -0.26 | 0.09 | . |
| <1x/month | -0.12 | 0.10 | 0.24 | -0.31 | 0.08 | . |
| Gender (Ref: Male) |  |  |  |  |  |  |
| Female | 0.51 | 0.05 | 0.00 | 0.42 | 0.60 | 0.00 |
| Other | 0.36 | 0.31 | 0.25 | -0.25 | 0.97 | . |
| Birth year/current age (Ref: 1998-2005; age 18-24) |  |  |  |  |  |  |
| 1993-1998; age 25-29 | 0.42 | 0.21 | 0.04 | 0.01 | 0.84 | 0.00 |
| 1983-1993; age 30-39 | 0.61 | 0.18 | 0.00 | 0.25 | 0.97 | . |
| 1973-1983; age 40-49 | 0.81 | 0.18 | 0.00 | 0.45 | 1.16 | . |
| 1963-1973; age 50-59 | 0.84 | 0.18 | 0.00 | 0.49 | 1.18 | . |
| 1953-1963; age 60-69 | 0.90 | 0.17 | 0.00 | 0.56 | 1.24 | . |
| 1943-1953; age 70-79 | 1.00 | 0.18 | 0.00 | 0.66 | 1.35 | . |
| 1943 or earlier; age 80 or older | 1.04 | 0.19 | 0.00 | 0.67 | 1.40 | . |
| Mother absence/presence (Ref: Present) |  |  |  |  |  |  |
| Absent | -0.55 | 0.24 | 0.02 | -1.02 | -0.08 | 0.02 |
| Father absence/presence (Ref: Present) |  |  |  |  |  |  |
| Absent | 0.05 | 0.14 | 0.70 | -0.22 | 0.33 | 0.70 |
| Childhood religion (Ref: No religion/atheist/agnostic) |  |  |  |  |  |  |
| Christianity | 0.31 | 0.10 | 0.00 | 0.11 | 0.51 | 0.01 |
| Some other religion | 0.27 | 0.14 | 0.04 | 0.01 | 0.54 | . |
| Race/ethnicity (Ref: Ethnic plurality) |  |  |  |  |  |  |
| Ethnic minority | -0.01 | 0.05 | 0.91 | -0.10 | 0.09 | 0.91 |
| *Note*. *N* = 38,312. SE, standard error; LCI, lower confidence interval; UCI, upper confidence interval. | | | | | | |

*Table S22d. E-Values and E-Value Limits for the Coefficients Shown in Table S22c (United States)*

| Characteristic | *E*-value for estimate^a^ | *E*-value for 95% CI^b^ |
| --- | --- | --- |
| Relationship with mother (Ref: Very/somewhat bad) |  |  |
| Very/somewhat good | 1.37 | 1.17 |
| Relationship with father (Ref: Very/somewhat bad) |  |  |
| Very/somewhat good | 1.39 | 1.23 |
| Parent marital status (Ref: Married) |  |  |
| Divorced | 1.33 | 1.17 |
| Never married | 1.21 | 1.00 |
| One or both parents had died | 1.32 | 1.00 |
| Subjective financial status growing up (Ref: Got by) |  |  |
| Lived comfortably | 1.26 | 1.14 |
| Found it difficult | 1.32 | 1.13 |
| Found it very difficult | 1.33 | 1.00 |
| Childhood abuse (Ref: No) |  |  |
| Yes | 1.27 | 1.09 |
| Outsider growing up (Ref: No) |  |  |
| Yes | 1.40 | 1.26 |
| Childhood health (Ref: Good) |  |  |
| Excellent | 1.60 | 1.47 |
| Very good | 1.33 | 1.14 |
| Fair | 1.35 | 1.00 |
| Poor | 1.21 | 1.00 |
| Immigration status (Ref: Born in this country) |  |  |
| Born in another country | 1.32 | 1.00 |
| Childhood religious service attendance (Ref: Never) |  |  |
| At least 1x/week | 1.13 | 1.00 |
| 1-3x/month | 1.19 | 1.00 |
| <1x/month | 1.23 | 1.00 |
| Gender (Ref: Male) |  |  |
| Female | 1.62 | 1.54 |
| Other | 1.48 | 1.00 |
| Birth year/current age (Ref: 1998-2005; age 18-24) |  |  |
| 1993-1998; age 25-29 | 1.54 | 1.06 |
| 1983-1993; age 30-39 | 1.71 | 1.38 |
| 1973-1983; age 40-49 | 1.89 | 1.57 |
| 1963-1973; age 50-59 | 1.92 | 1.60 |
| 1953-1963; age 60-69 | 1.98 | 1.67 |
| 1943-1953; age 70-79 | 2.07 | 1.75 |
| 1943 or earlier; age 80 or older | 2.11 | 1.77 |
| Mother absence/presence (Ref: Present) |  |  |
| Absent | 1.65 | 1.19 |
| Father absence/presence (Ref: Present) |  |  |
| Absent | 1.15 | 1.00 |
| Childhood religion (Ref: No religion/atheist/agnostic) |  |  |
| Christianity | 1.44 | 1.23 |
| Some other religion | 1.40 | 1.05 |
| Race/ethnicity (Ref: Ethnic plurality) |  |  |
| Ethnic minority | 1.04 | 1.00 |

*Note*. CI, confidence interval. ^a^The formula for calculating *E*-values can be found in VanderWeele and Ding (2017) The *E*-value for the effect estimate is the minimum strength of association (on the risk ratio scale) that an unmeasured confounder would need to have with both the predictor and the outcome to entirely explain away the observed association between them, conditional on the measured covariates. ^b^The *E*-value for the limit of the 95% confidence interval closest to the null denote the minimum strength of association (on the risk ratio scale) that an unmeasured confounder would need to have with both the predictor and the outcome to shift the confidence interval to include the null value, conditional on the measured covariates.

Table S23. Population Weighted Meta-Analysis of Results Demographic Group Means

| Characteristic | Category | Mean | 95% CI | SE |
| --- | --- | --- | --- | --- |
| Overall |  | 8.08 | (7.99,8.16) |  |
| Age group |  |  |  |  |
|  | 18-24 | 7.74 | (7.62,7.86) | 0.062 |
|  | 25-29 | 7.85 | (7.71,7.99) | 0.071 |
|  | 30-39 | 7.79 | (7.71,7.87) | 0.041 |
|  | 40-49 | 7.97 | (7.89,8.05) | 0.042 |
|  | 50-59 | 8.14 | (8.04,8.23) | 0.049 |
|  | 60-69 | 8.18 | (8.05,8.30) | 0.065 |
|  | 70-79 | 8.06 | (7.83,8.29) | 0.116 |
|  | 80 or older | 8.69 | (8.39,9.00) | 0.154 |
| Gender |  |  |  |  |
|  | Male | 7.86 | (7.81,7.92) | 0.030 |
|  | Female | 8.13 | (8.07,8.18) | 0.027 |
|  | Other | 7.09 | (6.47,7.72) | 0.319 |
| Marital status |  |  |  |  |
|  | Married | 8.25 | (8.18,8.32) | 0.037 |
|  | Separated | 7.65 | (7.26,8.04) | 0.199 |
|  | Divorced | 7.76 | (7.51,8.01) | 0.127 |
|  | Widowed | 7.83 | (7.42,8.23) | 0.206 |
|  | Domestic partner | 7.75 | (7.57,7.94) | 0.095 |
|  | Single, never married | 7.46 | (7.39,7.54) | 0.037 |
| Employment status |  |  |  |  |
|  | Employed for an employer | 7.98 | (7.93,8.04) | 0.027 |
|  | Self-employed | 8.12 | (7.98,8.25) | 0.069 |
|  | Retired | 8.17 | (8.03,8.31) | 0.071 |
|  | Student | 7.63 | (7.46,7.81) | 0.088 |
|  | Homemaker | 8.01 | (7.83,8.20) | 0.093 |
|  | Unemployed and looking for a job | 7.25 | (6.95,7.54) | 0.149 |
|  | None of these/other | 7.80 | (7.35,8.26) | 0.231 |
| Education |  |  |  |  |
|  | Up to 8 years | 8.07 | (7.86,8.28) | 0.106 |
|  | 9-15 years | 7.99 | (7.94,8.04) | 0.025 |
|  | 16+ years | 7.92 | (7.84,8.00) | 0.042 |
| Religious service attendance |  |  |  |  |
|  | >1x/week | 8.72 | (8.62,8.81) | 0.048 |
|  | 1x/week | 8.30 | (8.22,8.38) | 0.040 |
|  | 1-3x/month | 8.20 | (8.10,8.30) | 0.051 |
|  | A few times a year | 7.95 | (7.86,8.05) | 0.048 |
|  | Never | 7.68 | (7.60,7.75) | 0.039 |
| Immigration status |  |  |  |  |
|  | Born in this country | 8.02 | (7.98,8.06) | 0.021 |
|  | Born in another country | 7.72 | (7.52,7.93) | 0.103 |
| *Note*. *N* = 202,898. CI, confidence interval; SE, standard error. | | | | |

Table S24. Population Weighted Meta-Analysis of Childhood Predictors Regression Results

| Characteristic | Predictor (level) | Estimate | 95% CI | SE | *E*-value for estimate^a^ | | *E*-value for 95% CI^b^ | |
| --- | --- | --- | --- | --- | --- | --- | --- | --- |
| Relationship with mother | (Ref: Very bad/somewhat bad) |  |  |  |  |  | |  |
|  | Very/somewhat good | 0.05 | (-0.09,0.19) | 0.071 | 1.18 | 1.00 | |  |
| Relationship with father | (Ref: Very bad/somewhat bad) |  |  |  |  |  | |  |
|  | Very/somewhat good | 0.10 | (-0.01,0.21) | 0.055 | 1.26 | 1.00 | |  |
| Parent marital status | (Ref: Parents married) |  |  |  |  |  | |  |
|  | No, divorced | 0.03 | (-0.16,0.21) | 0.094 | 1.12 | 1.00 | |  |
|  | Single, never married | 0.15 | (-0.09,0.38) | 0.121 | 1.33 | 1.00 | |  |
|  | No, one or both had died | -0.04 | (-0.36,0.28) | 0.162 | 1.15 | 1.00 | |  |
| Subjective financial status growing up growing up | (Ref: Got by) |  |  |  |  |  | |  |
|  | Lived comfortably | 0.28 | (0.20,0.36) | 0.041 | 1.51 | 1.41 | |  |
|  | Found it difficult | -0.06 | (-0.18,0.05) | 0.060 | 1.20 | 1.00 | |  |
|  | Found it very difficult | -0.29 | (-0.54,-0.04) | 0.127 | 1.52 | 1.15 | |  |
| Abuse | (Ref: No) |  |  |  |  |  | |  |
|  | Yes | -0.02 | (-0.12,0.09) | 0.055 | 1.09 | 1.00 | |  |
| Outsider growing up growing up | (Ref: No) |  |  |  |  |  | |  |
|  | Yes | -0.09 | (-0.19,0.00) | 0.049 | 1.25 | 1.00 | |  |
| Self-rated health growing up | (Ref: Good) |  |  |  |  |  | |  |
|  | Excellent | 0.50 | (0.39,0.62) | 0.059 | 1.79 | 1.65 | |  |
|  | Very good | 0.25 | (0.15,0.35) | 0.052 | 1.47 | 1.33 | |  |
|  | Fair | -0.24 | (-0.40,-0.08) | 0.081 | 1.45 | 1.23 | |  |
|  | Poor | -0.43 | (-0.77,-0.08) | 0.176 | 1.69 | 1.23 | |  |
| Immigration status | (Ref: Born in this country) |  |  |  |  |  | |  |
|  | No | -0.18 | (-0.37,0.01) | 0.096 | 1.38 | 1.00 | |  |
| Childhood religious service attendance | (Ref: Never) |  |  |  |  |  | |  |
|  | At least 1x/week | 0.41 | (0.25,0.56) | 0.079 | 1.67 | 1.47 | |  |
|  | 1-3x/month | 0.32 | (0.17,0.47) | 0.076 | 1.56 | 1.36 | |  |
|  | Less than 1x/month | 0.07 | (-0.10,0.24) | 0.089 | 1.21 | 1.00 | |  |
| Birth year/current age | (Ref: 1998-2005; age 18-24; age 18-24) |  |  |  |  |  | |  |
|  | 1993-1998; age 25-29 | 0.19 | (0.03,0.34) | 0.080 | 1.38 | 1.12 | |  |
|  | 1983-1993; age 30-39 | 0.19 | (0.05,0.32) | 0.069 | 1.39 | 1.18 | |  |
|  | 1973-1983; age 40-49 | 0.29 | (0.15,0.43) | 0.070 | 1.52 | 1.34 | |  |
|  | 1963-1973; age 50-59 | 0.39 | (0.25,0.53) | 0.073 | 1.65 | 1.46 | |  |
|  | 1953-1963; age 60-69 | 0.46 | (0.30,0.63) | 0.085 | 1.74 | 1.53 | |  |
|  | 1943-1953; age 70-79 | 0.44 | (0.19,0.69) | 0.128 | 1.71 | 1.39 | |  |
|  | 1943 or earlier; age 80+ | 0.72 | (0.42,1.02) | 0.154 | 2.06 | 1.68 | |  |
| Gender | (Ref: Male) |  |  |  |  |  | |  |
|  | Female | 0.31 | (0.24,0.38) | 0.035 | 1.55 | 1.46 | |  |
|  | Other | -0.55 | (-1.09,-0.01) | 0.276 | 1.85 | 1.07 | |  |

*Note*. CI, confidence interval; SE, standard error. ^a^The formula for calculating *E*-values can be found in VanderWeele and Ding (2017) The *E*-value for the effect estimate is the minimum strength of association (on the risk ratio scale) that an unmeasured confounder would need to have with both the predictor and the outcome to entirely explain away the observed association between them, conditional on the measured covariates. ^b^The *E*-value for the limit of the 95% confidence interval closest to the null denote the minimum strength of association (on the risk ratio scale) that an unmeasured confounder would need to have with both the predictor and the outcome to shift the confidence interval to include the null value, conditional on the measured covariates.
