# Supplementary material for: Sociodemographic variation and childhood predictors of showing love and care for others in 22 countries
Source: Sci Rep. 2025 Dec 26;16:1796. doi: 10.1038/s41598-025-31380-9 (PMC12804932; doi:10.1038/s41598-025-31380-9)
Supplement: Supplementary file 2 — Supplementary Material 2 [file 41598_2025_31380_MOESM2_ESM.docx]

**Supplemental Figures for “Sociodemographic Variation and Childhood Predictors of Showing Love and Care for Others in 22 Countries”**

Matthew T. Lee, Renae Wilkinson, Katelyn N. G. Long, Brendan W. Case,

James L. Ritchie-Dunham, Matt Bradshaw, R. Noah Padgett,

Byron R. Johnson, Tyler J. VanderWeele

Figure S1. Forest plot for “Age group: 18-24”


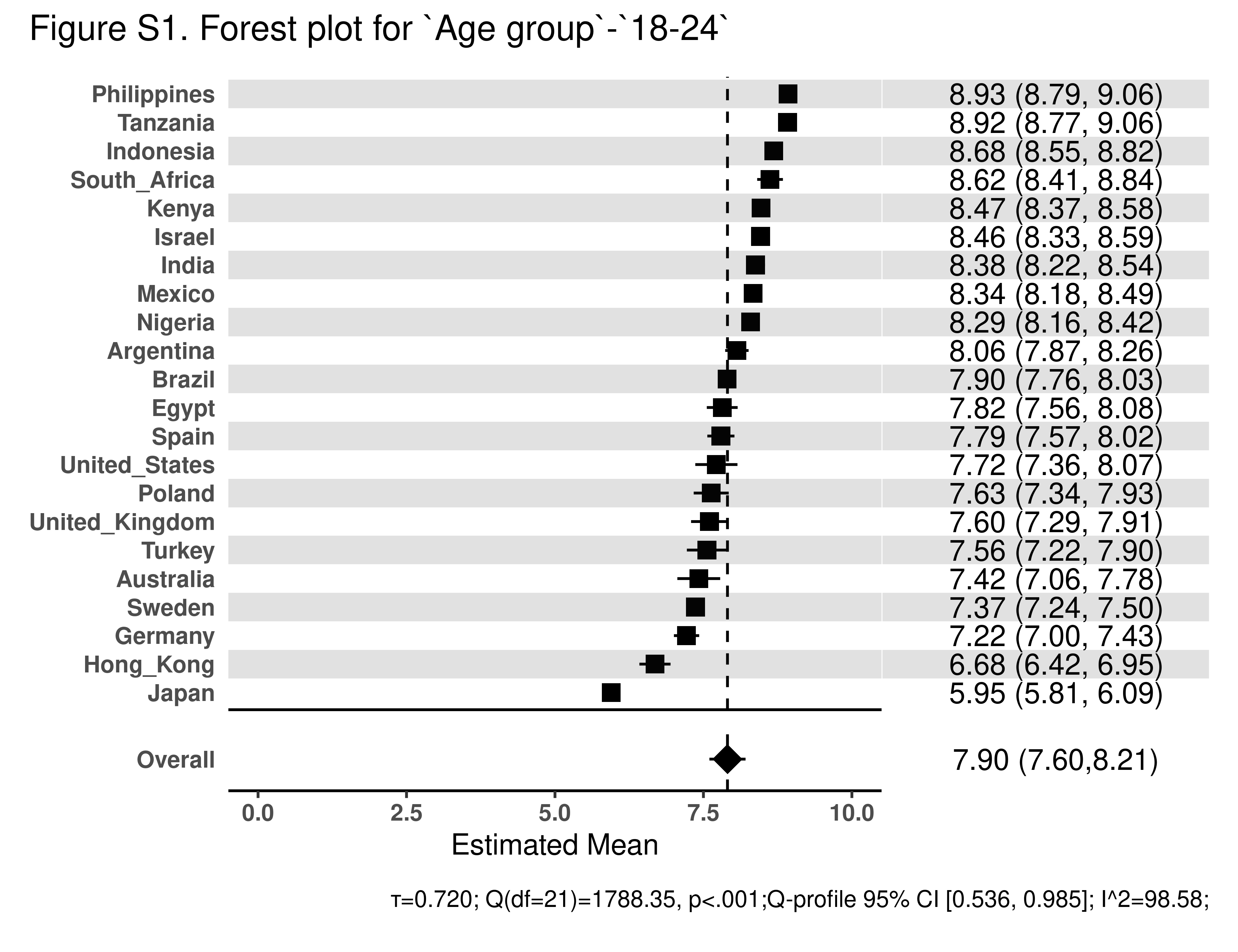


Figure S2. Forest plot for “Age group: 25-29”


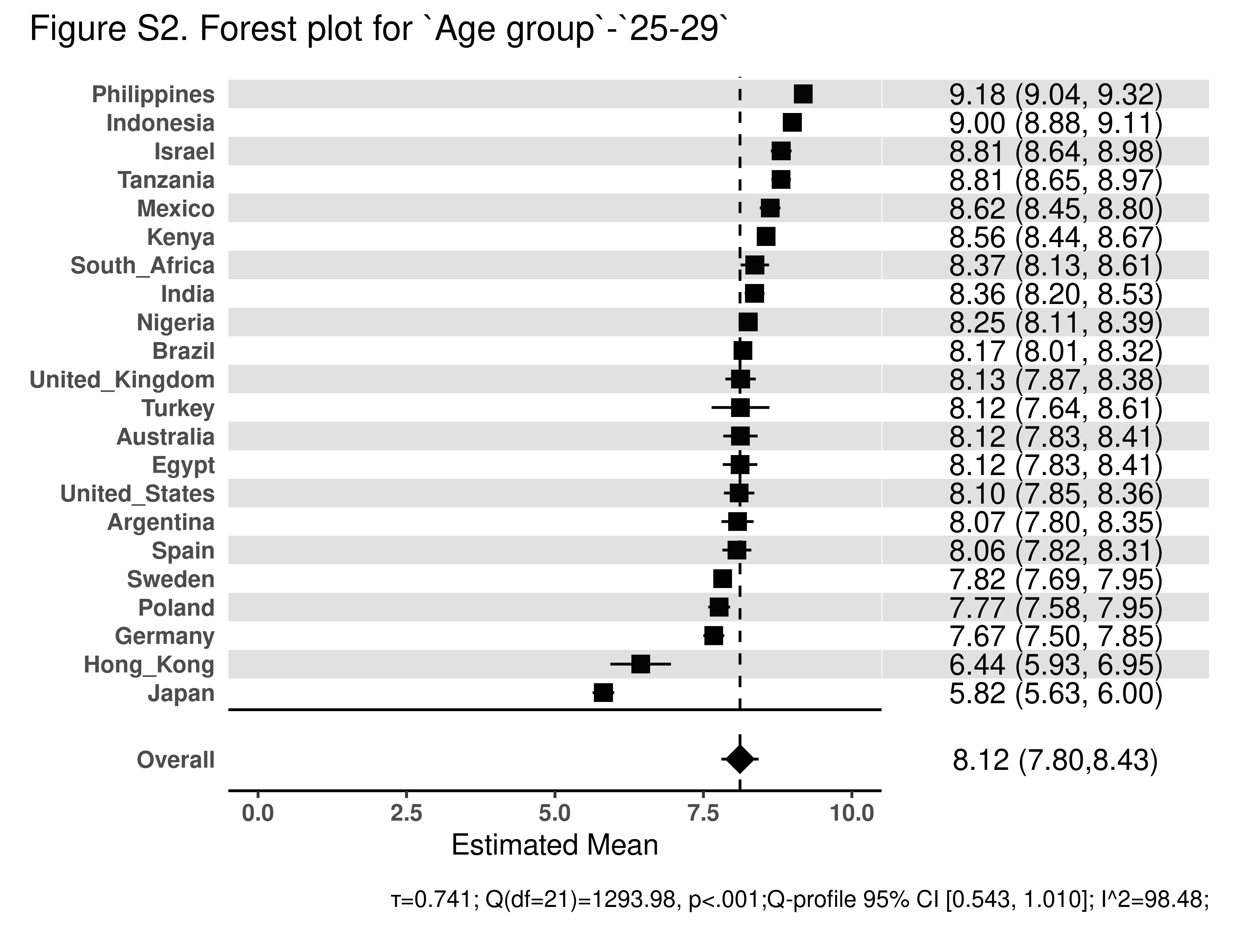


Figure S3. Forest plot for “Age group: 30-39”


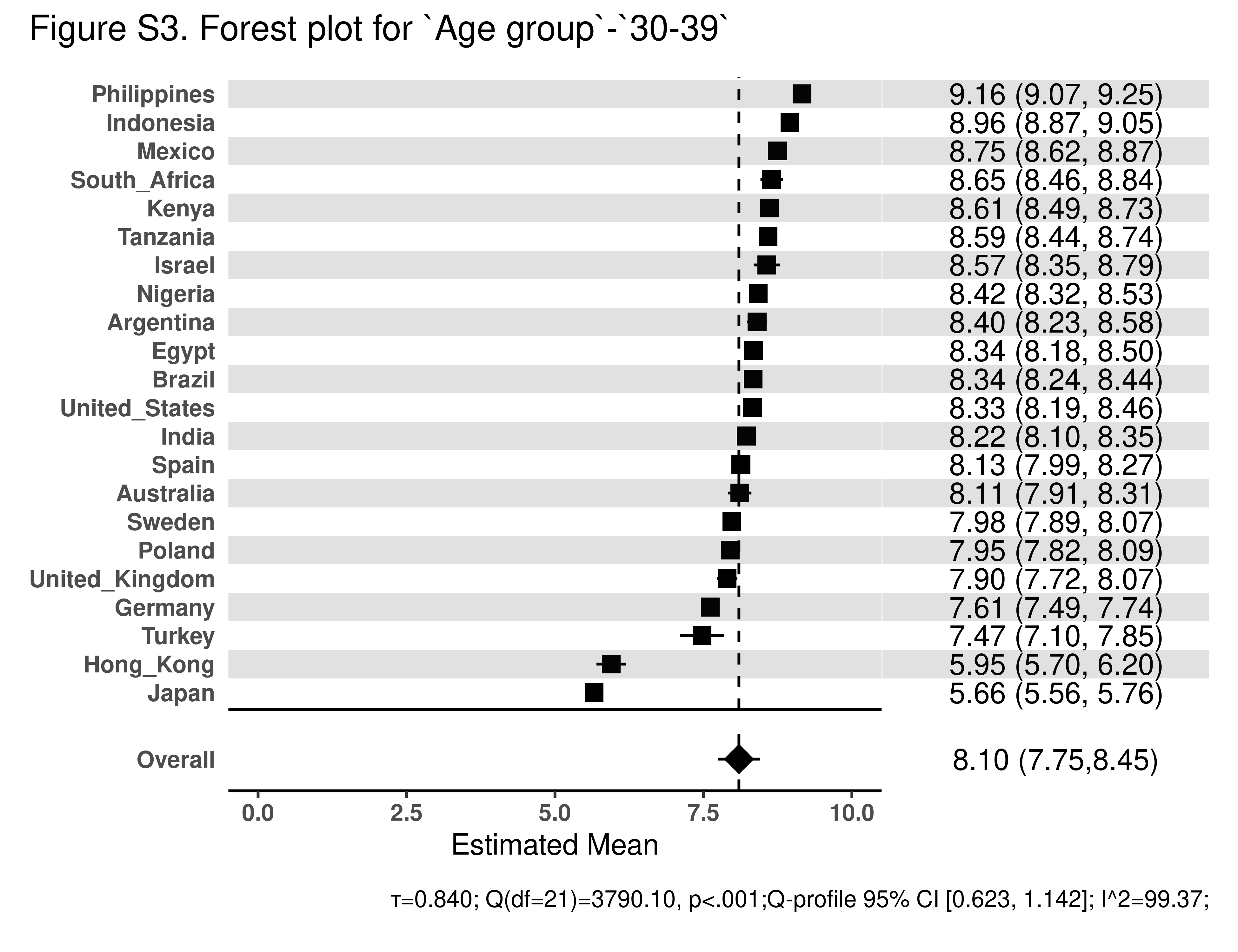


Figure S4. Forest plot for “Age group: 40-49”
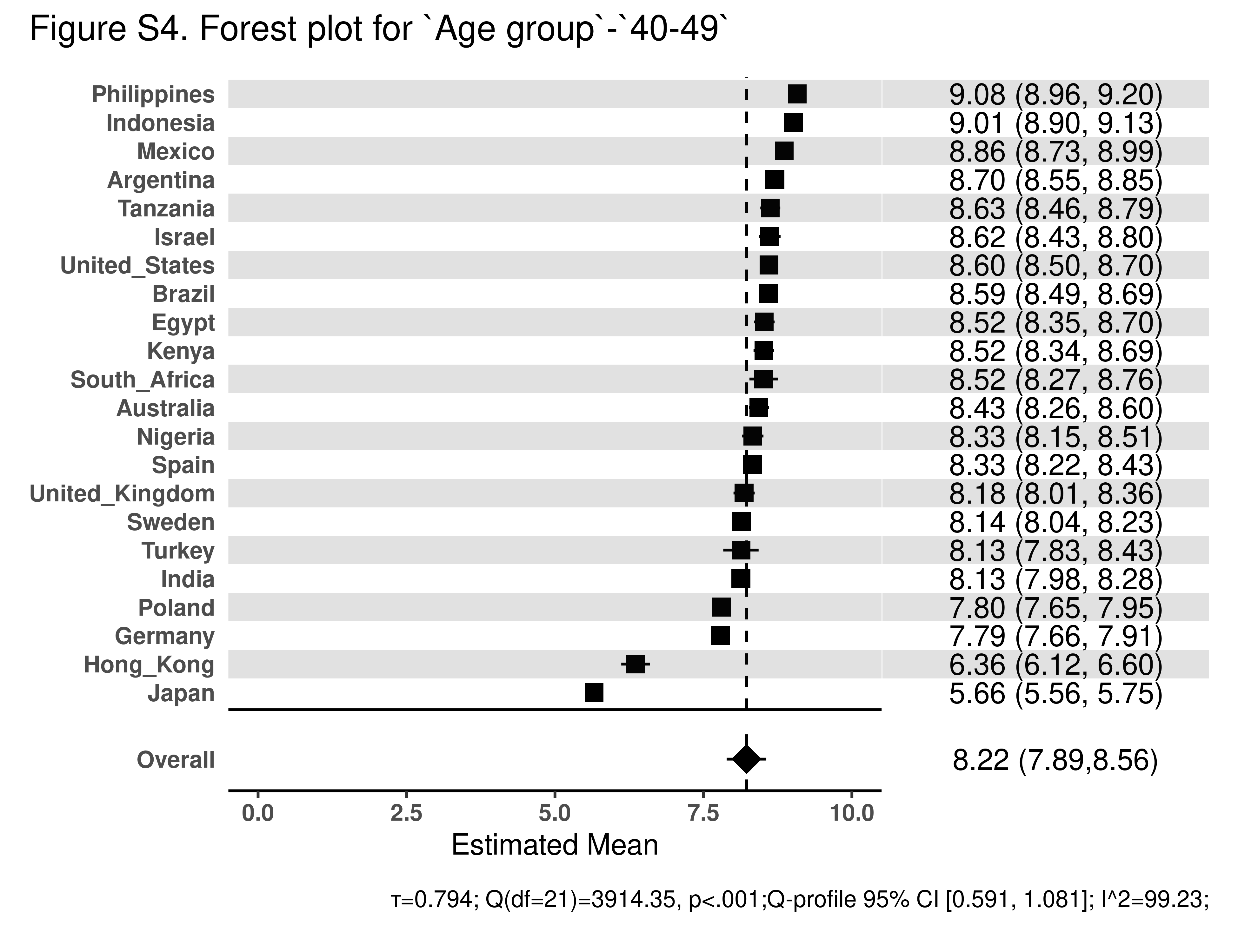


Figure S5. Forest plot for “Age group: 50-59”


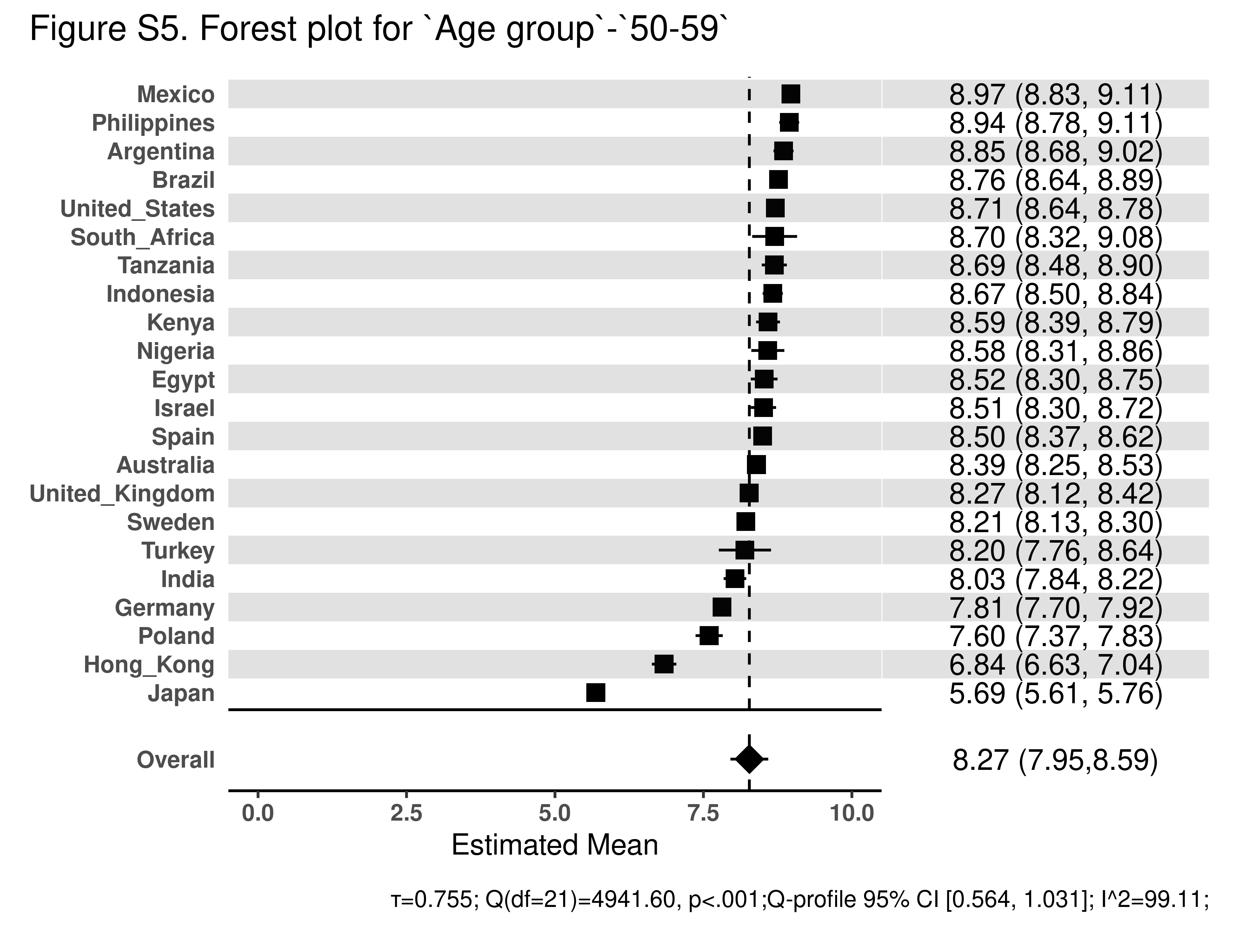


Figure S6. Forest plot for “Age group: 60-69”
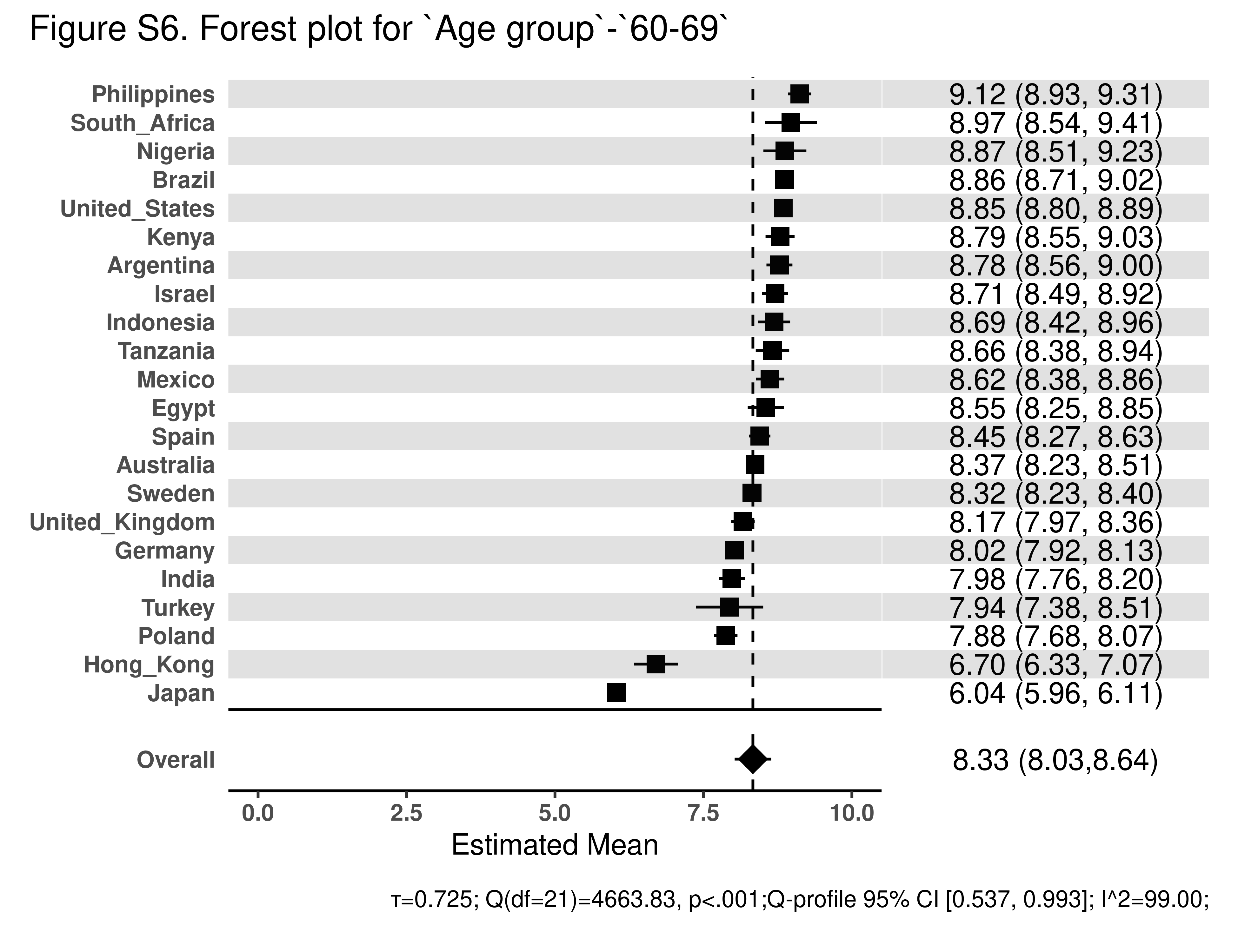


Figure S7. Forest plot for “Age group: 70-79”


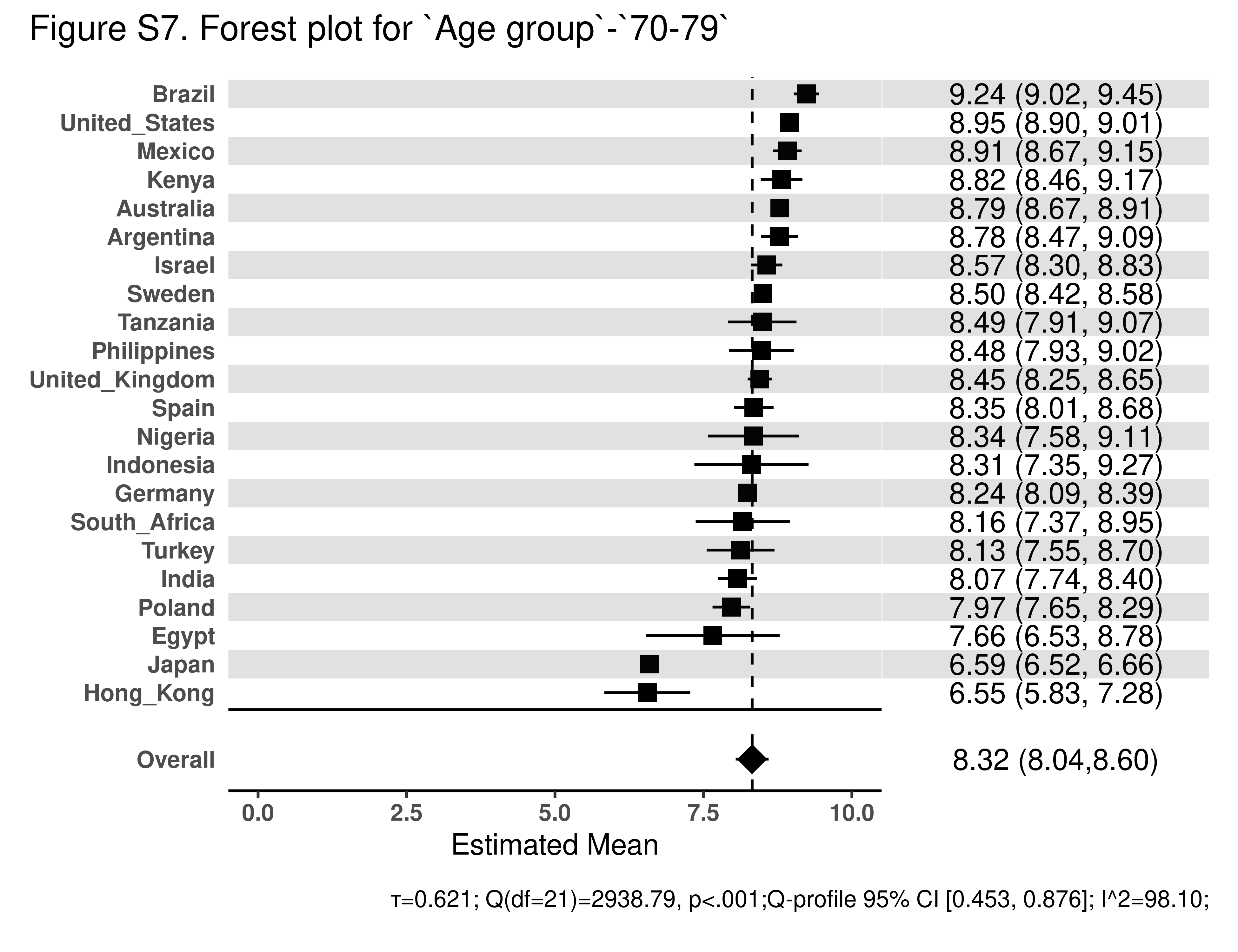


Figure S8. Forest plot for “Age group: 80 or older”


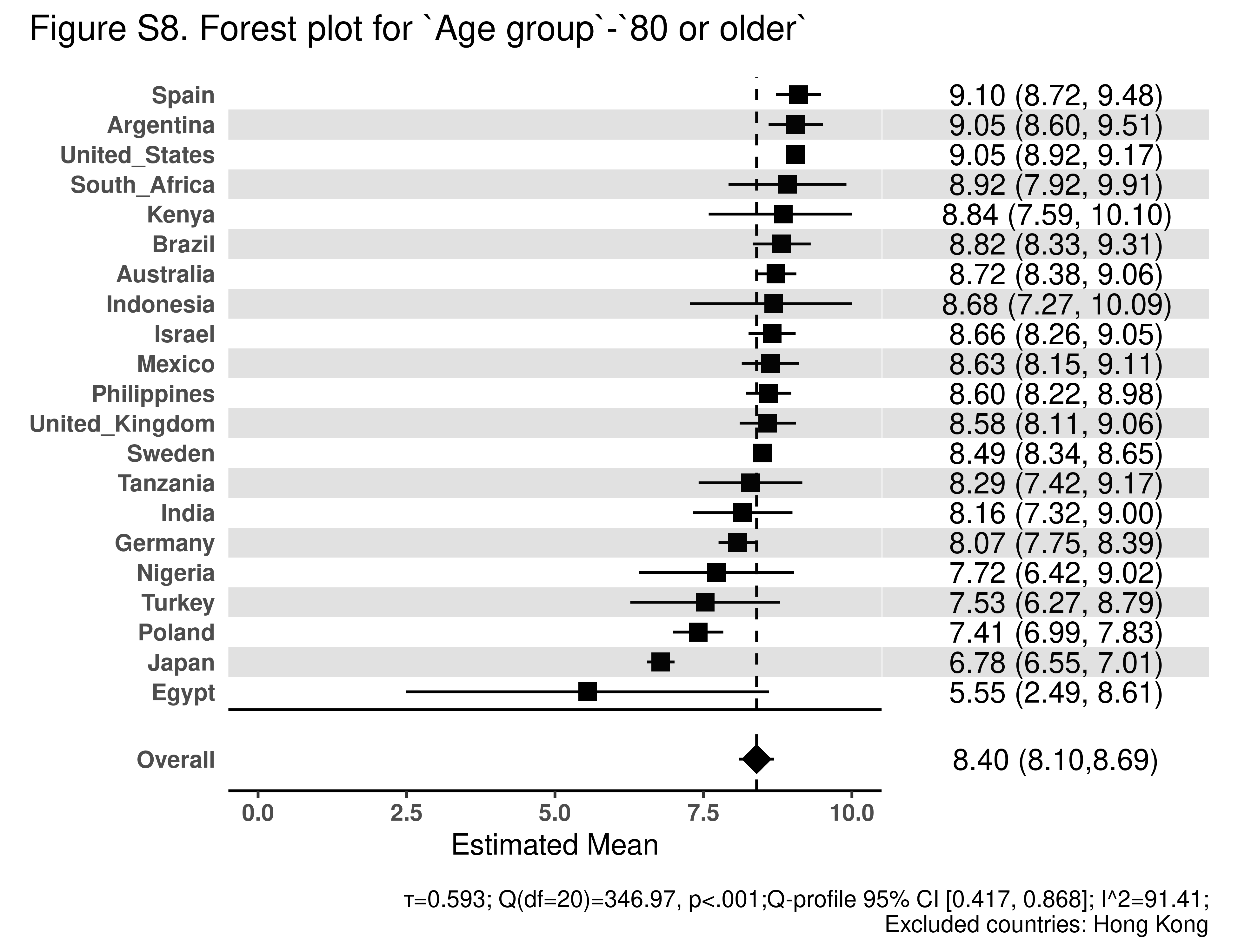


Figure S9. Forest plot for “Gender: Male”


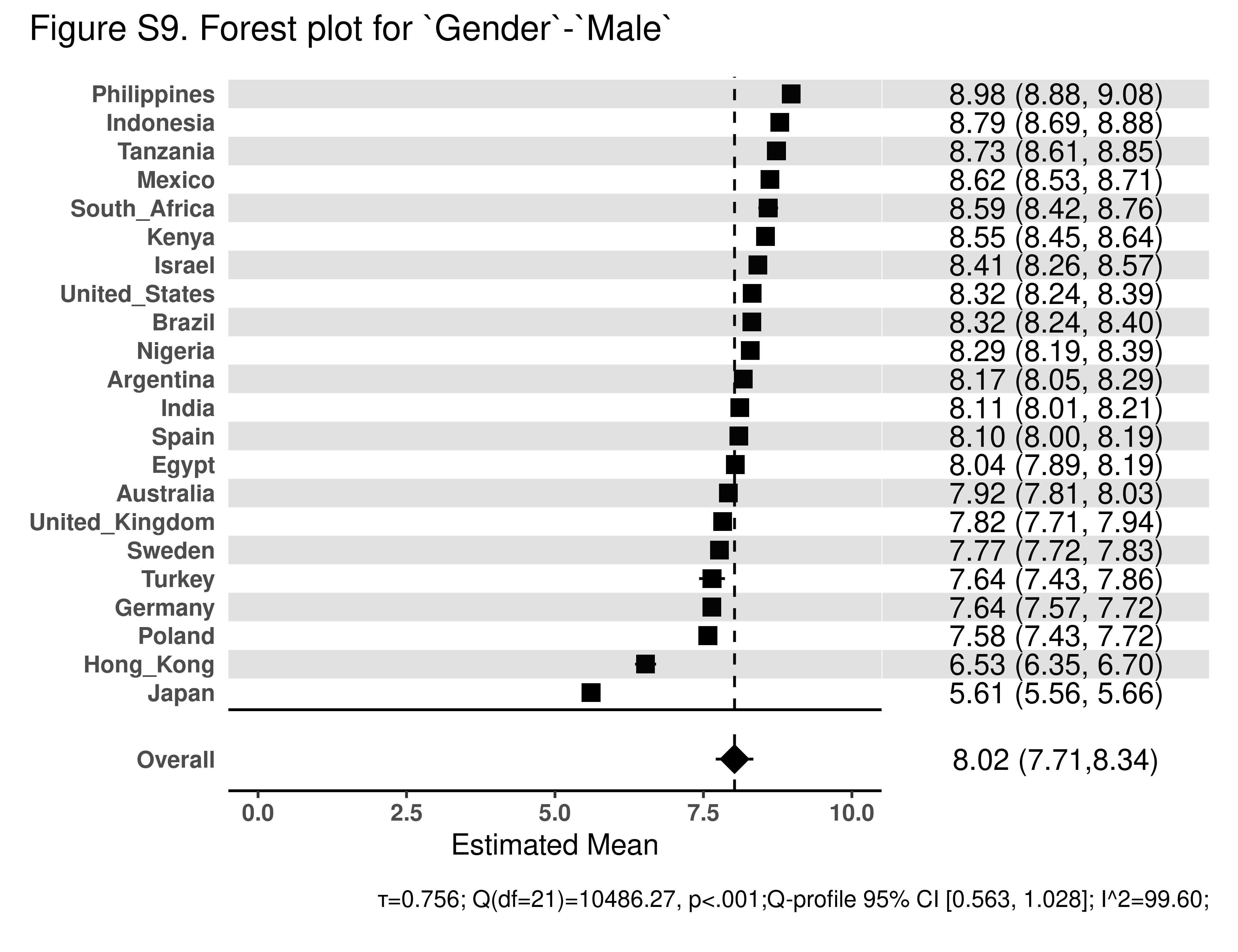


Figure S10. Forest plot for “Gender: Female”


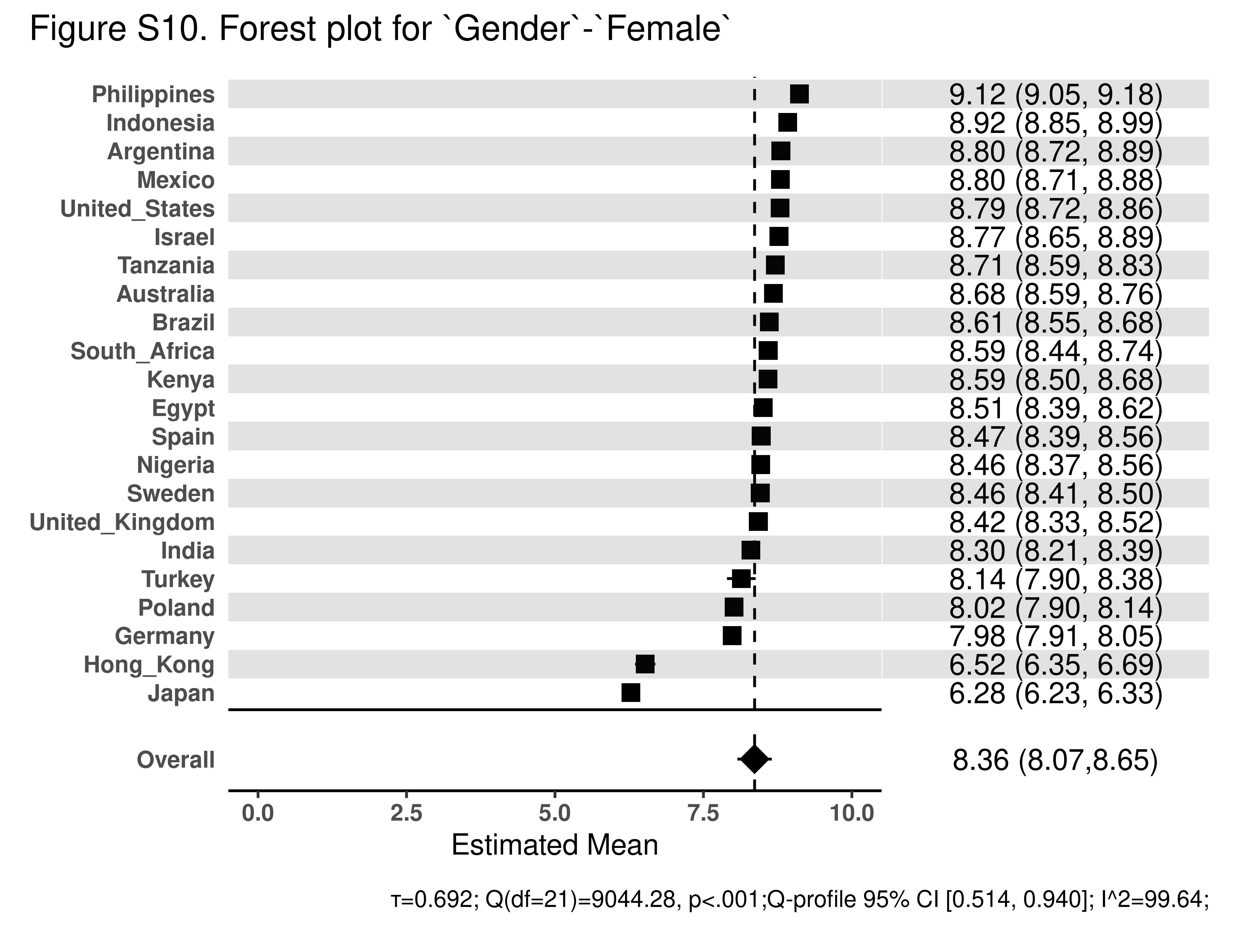


Figure S11. Forest plot for “Gender: Other”


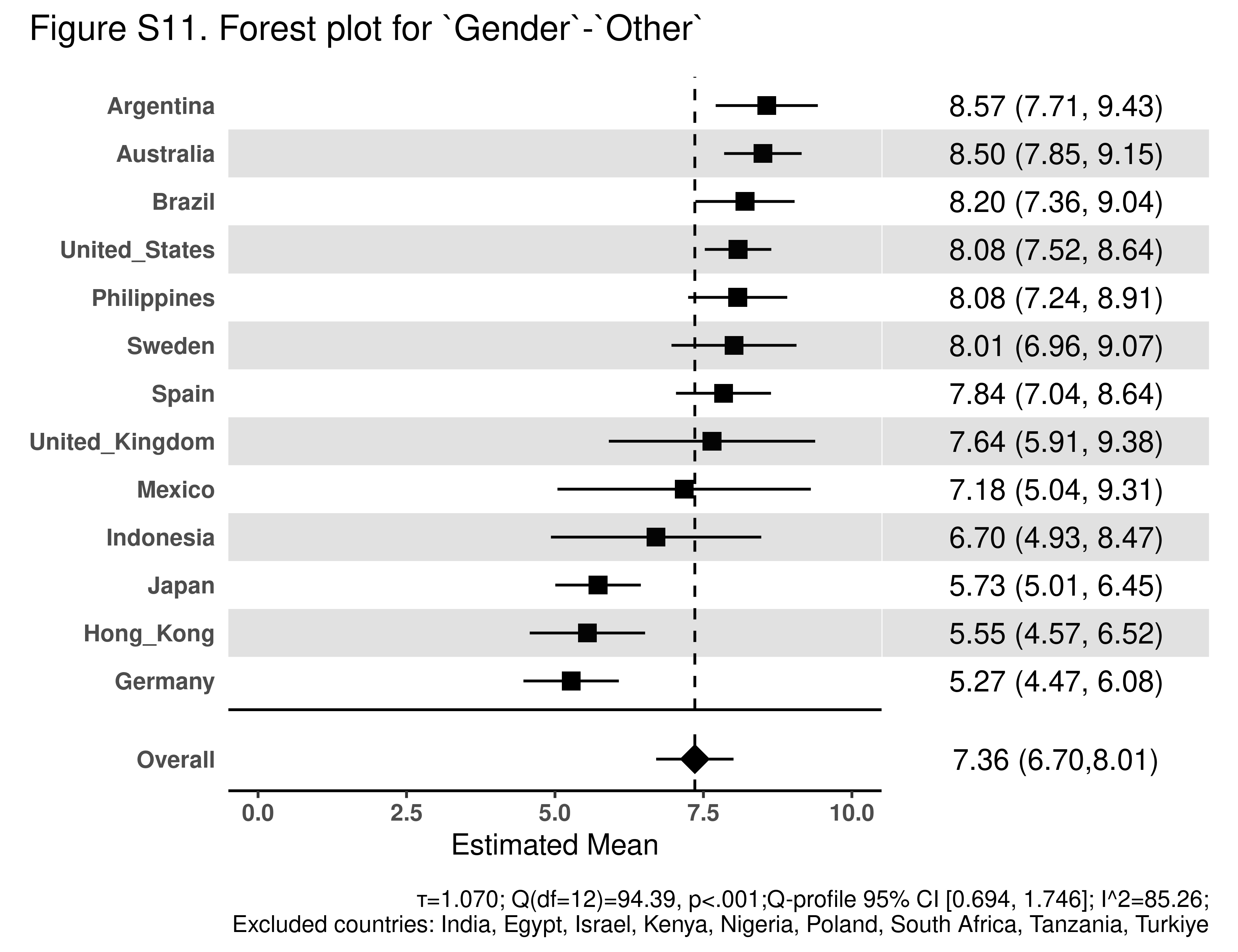


Figure S12. Forest plot for “Marital status: Single, never married”


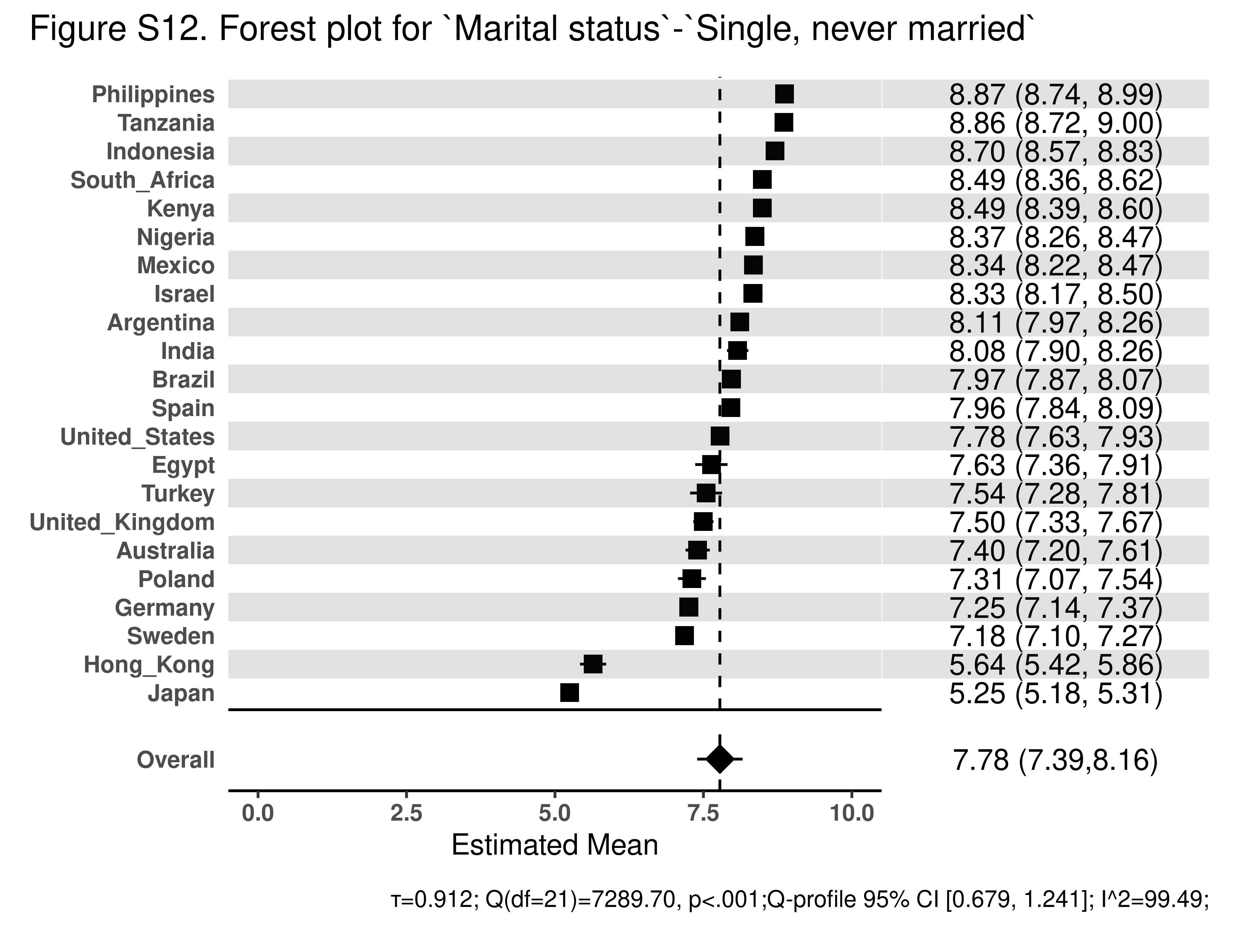


Figure S13. Forest plot for “Marital status: Married”


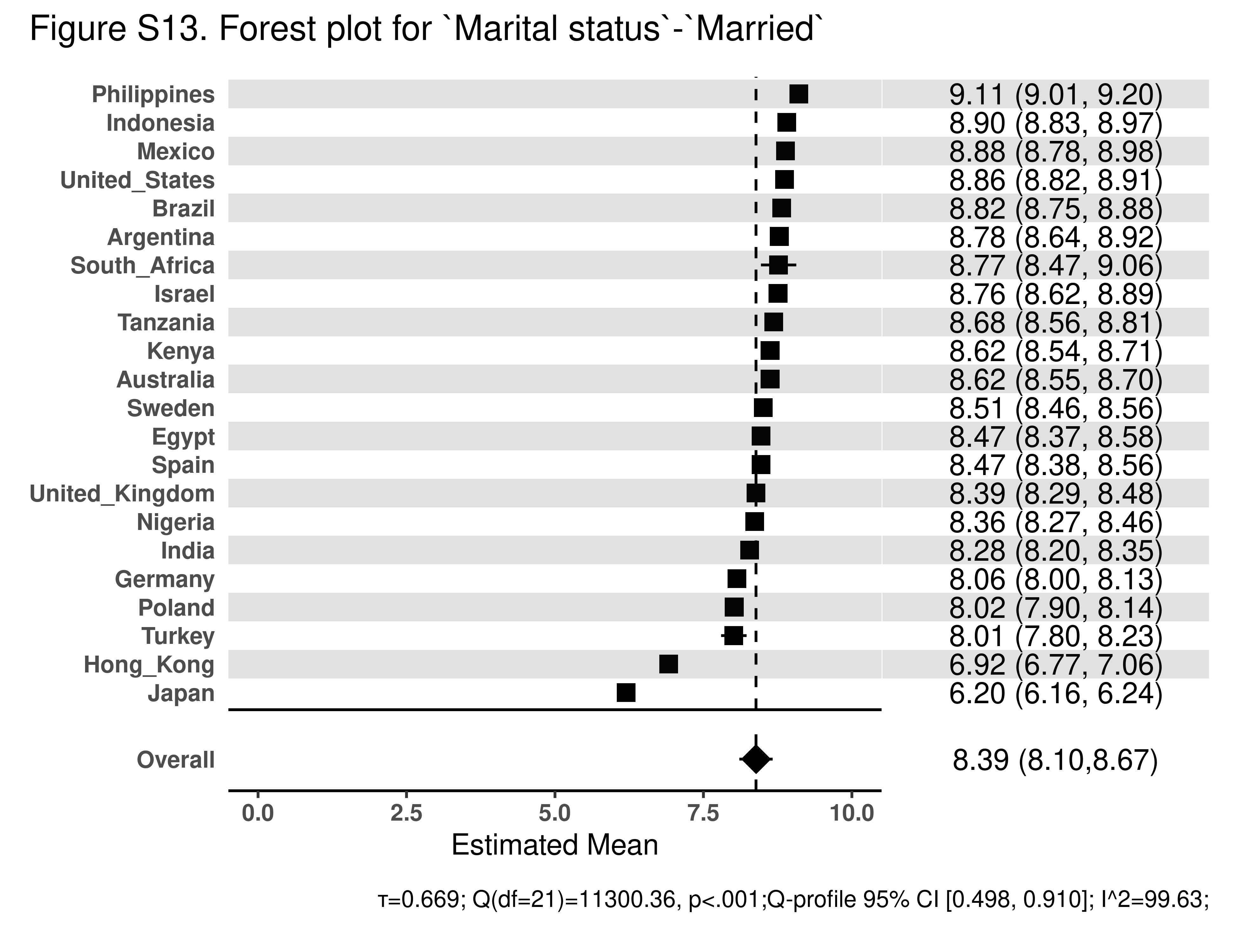


Figure S14. Forest plot for “Marital status: Separated”


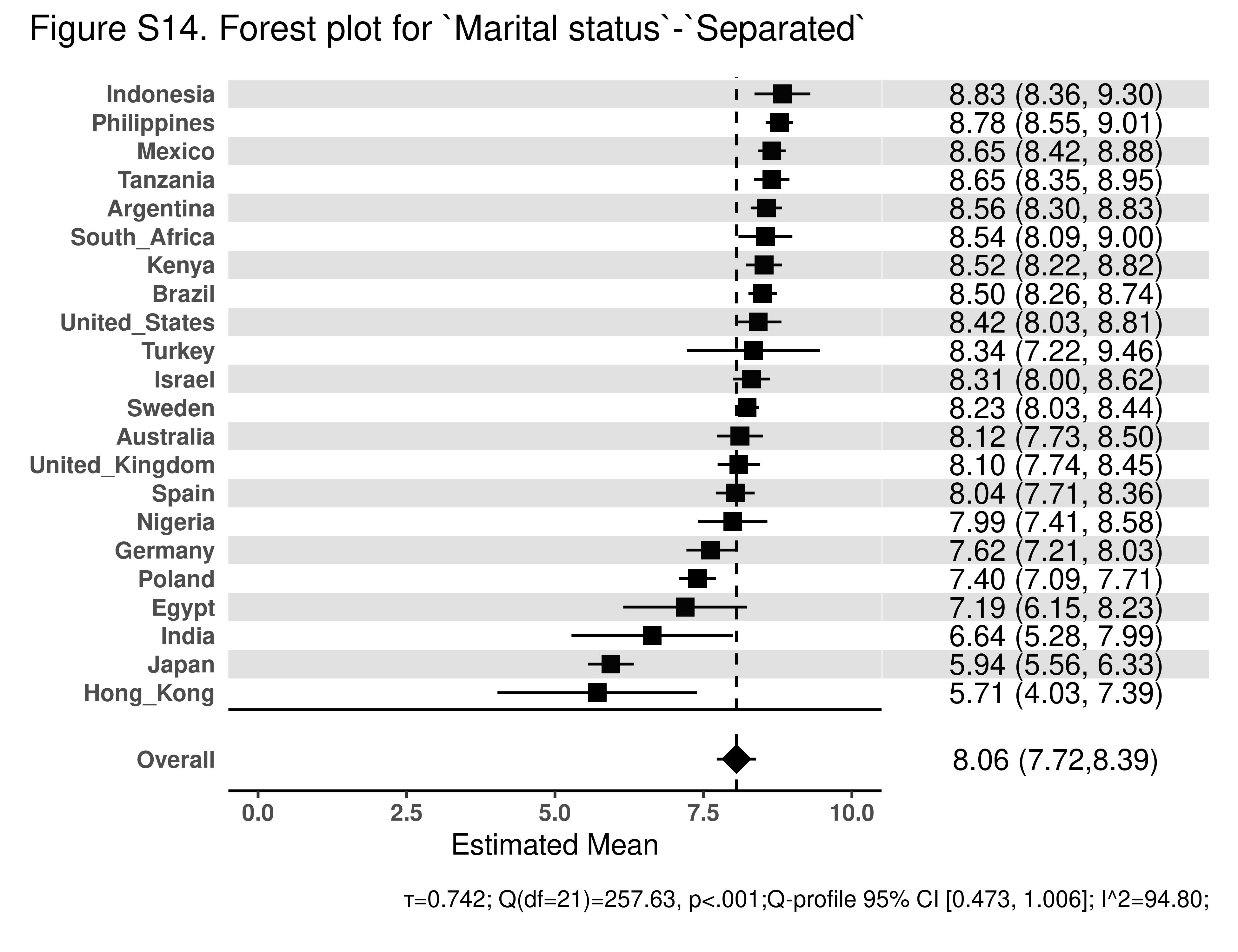


Figure S15. Forest plot for “Marital status: Divorced”


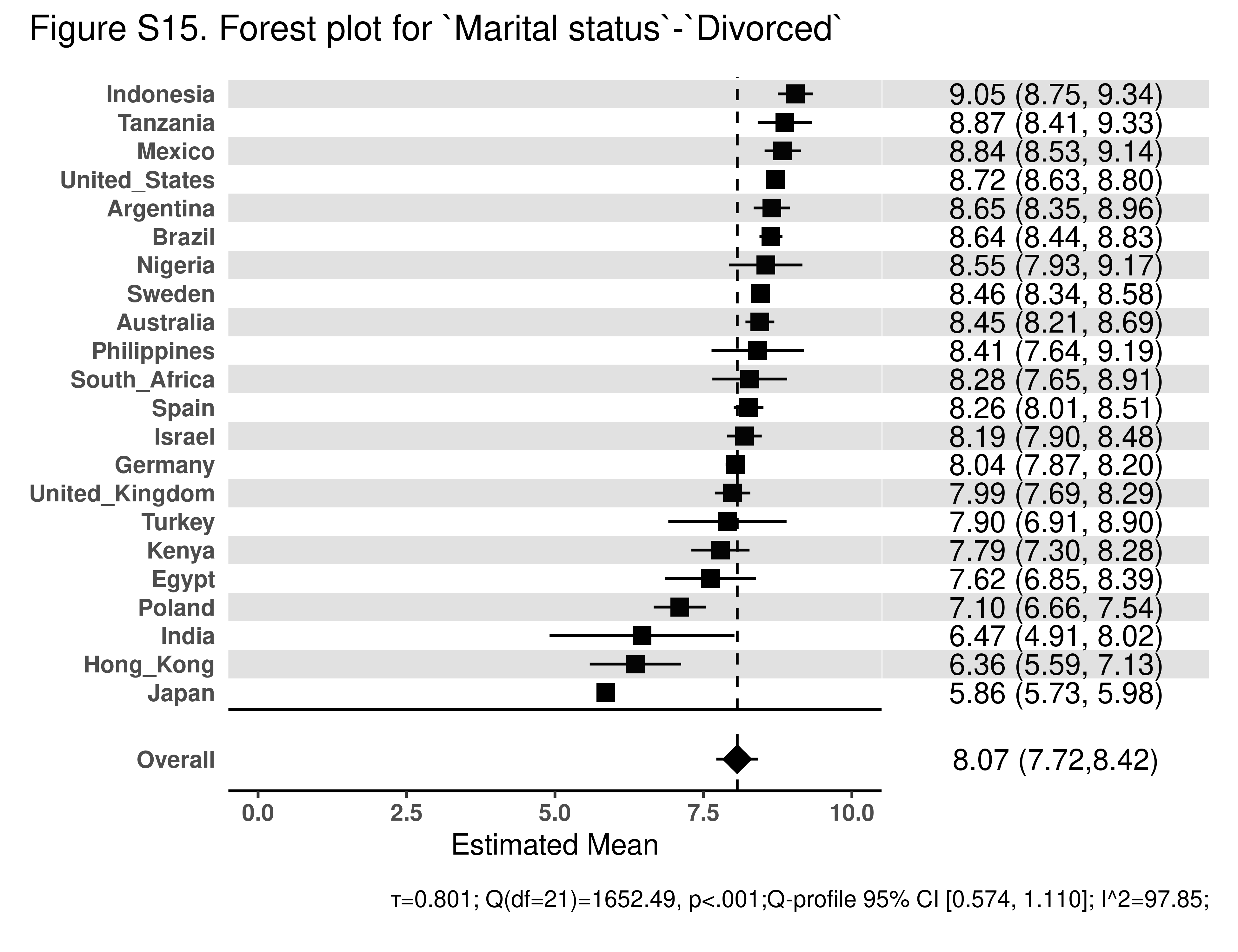


Figure S16. Forest plot for “Marital status: Widowed”


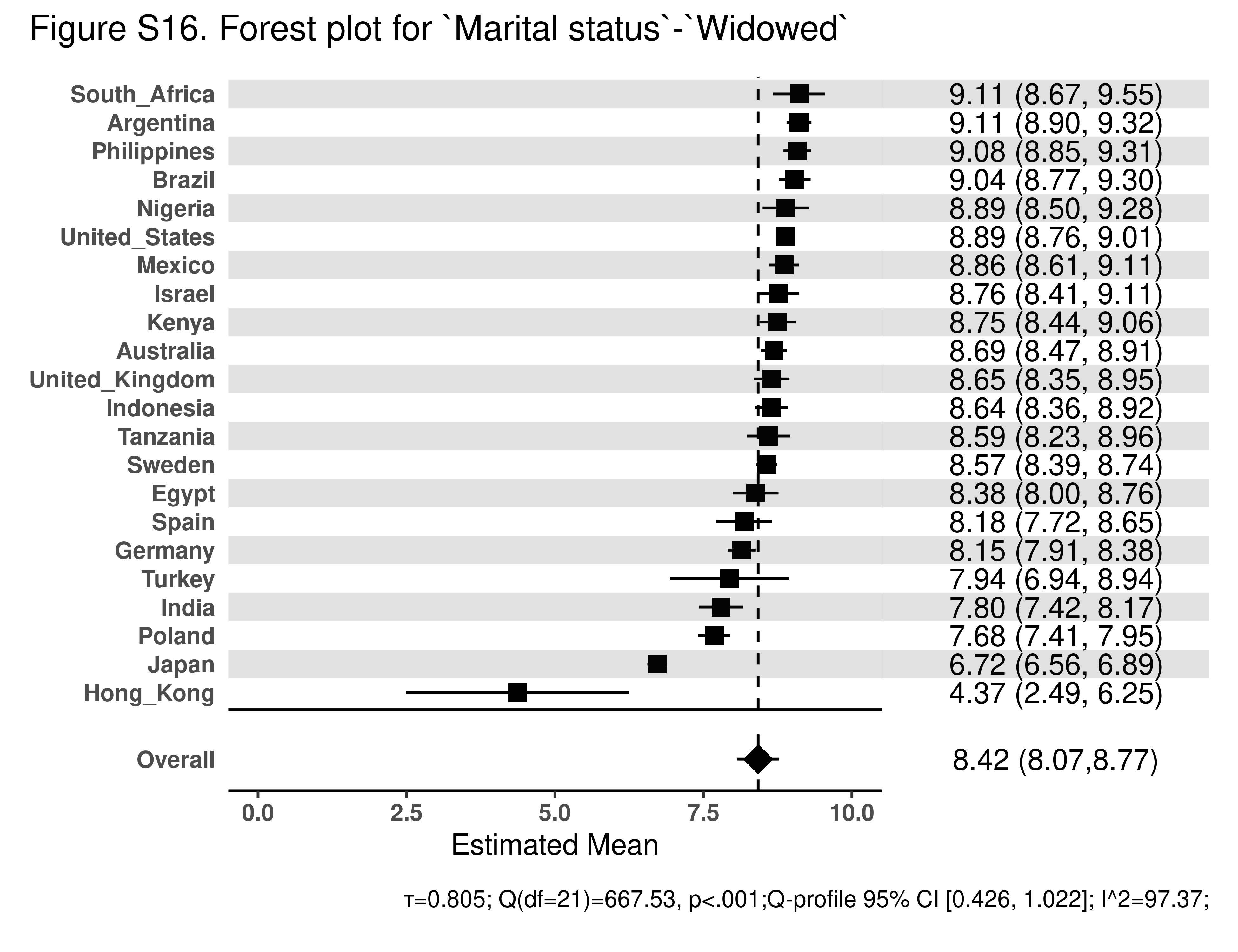


Figure S17. Forest plot for “Marital status: Domestic partner”


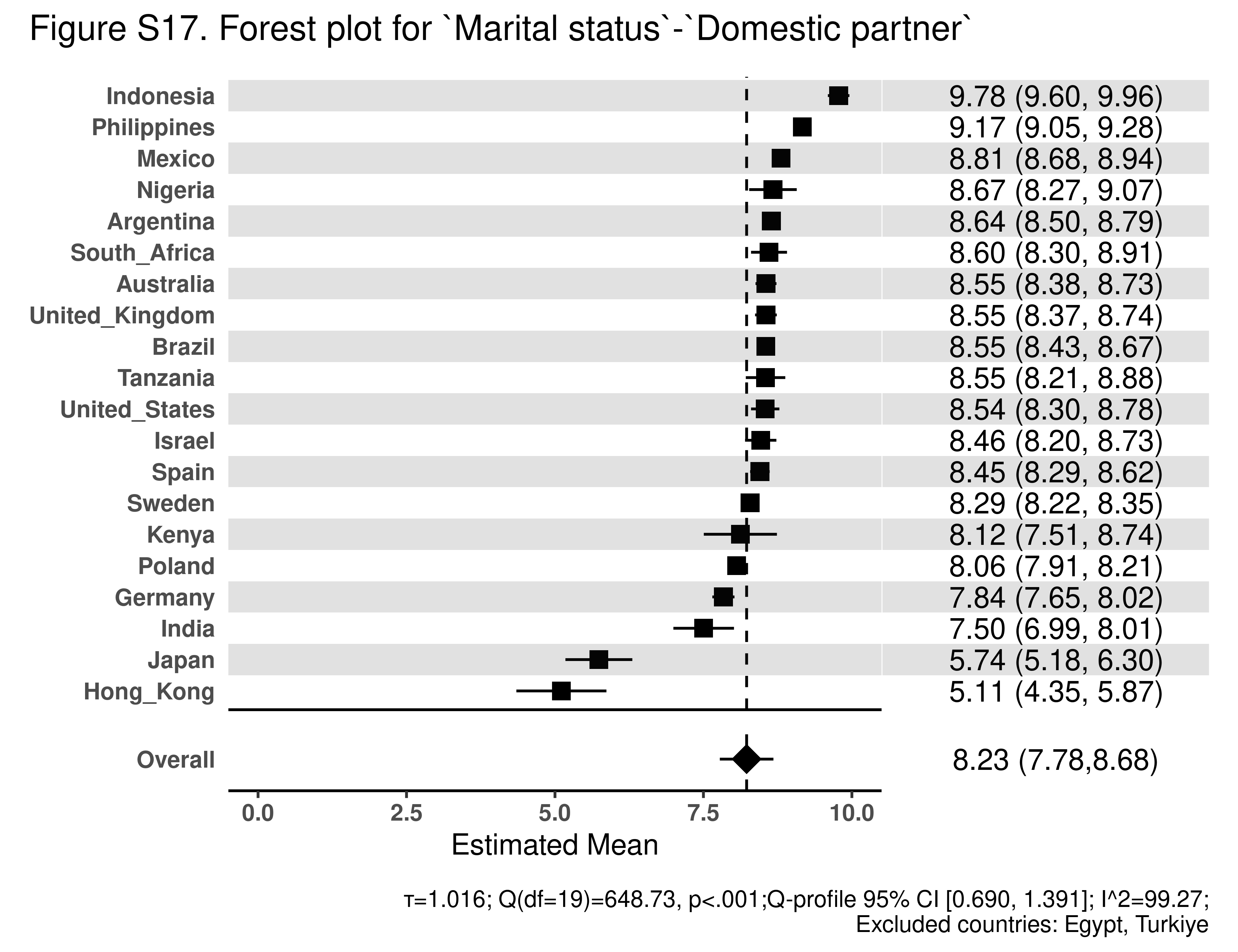


Figure S18. Forest plot for “Employment status: Employed for an employer”


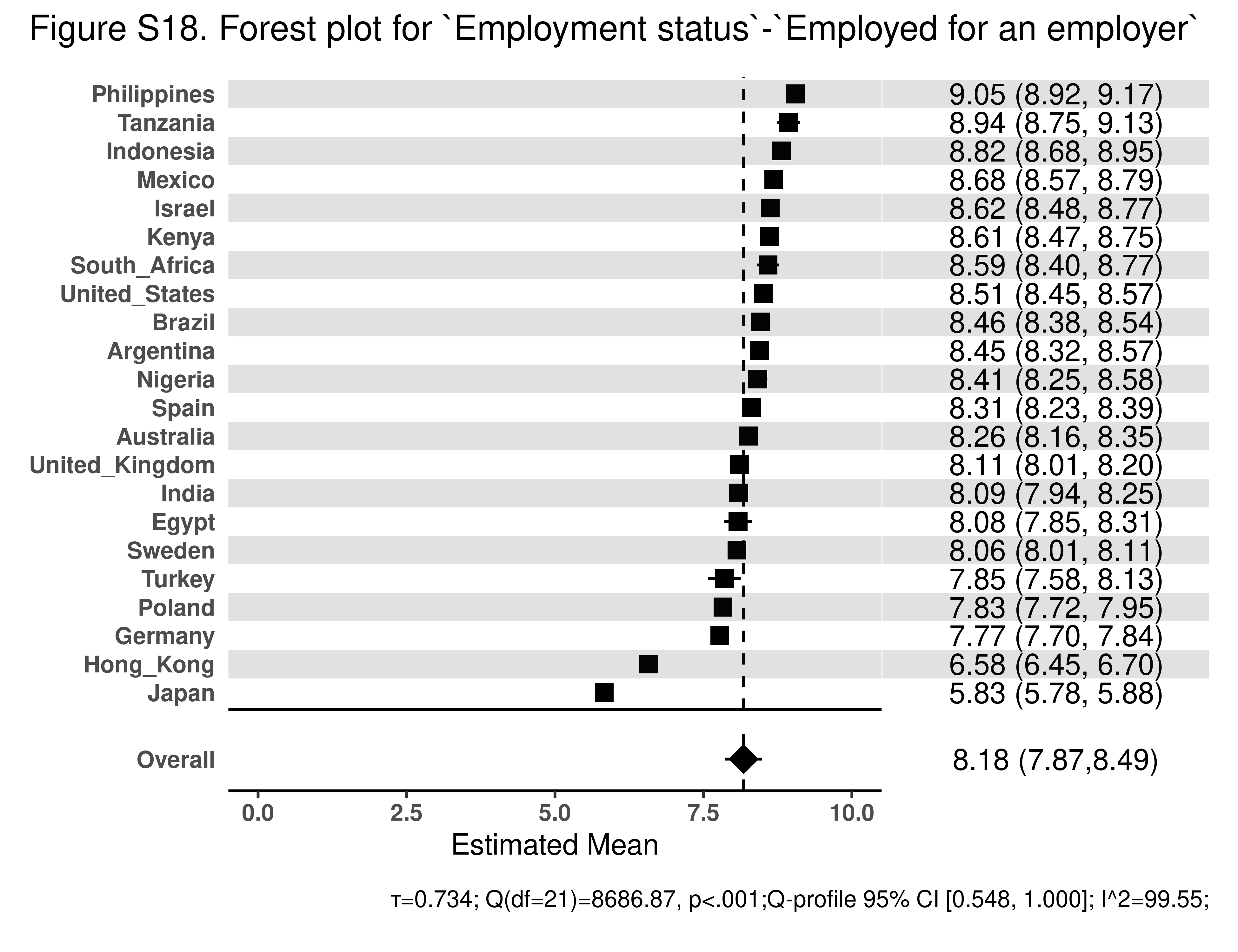


Figure S19. Forest plot for “Employment status: Self-employed”


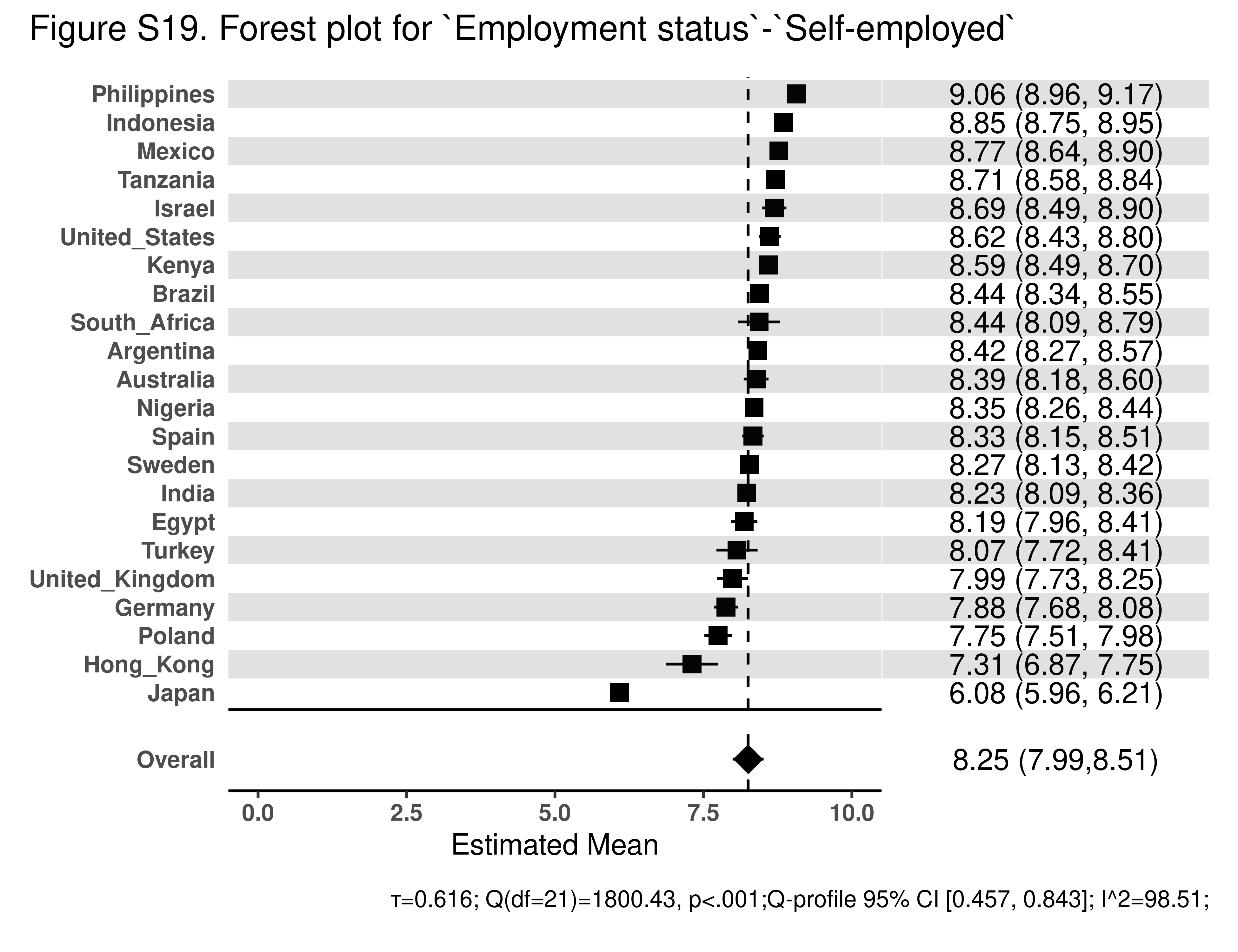


Figure S20. Forest plot for “Employment status: Retired”


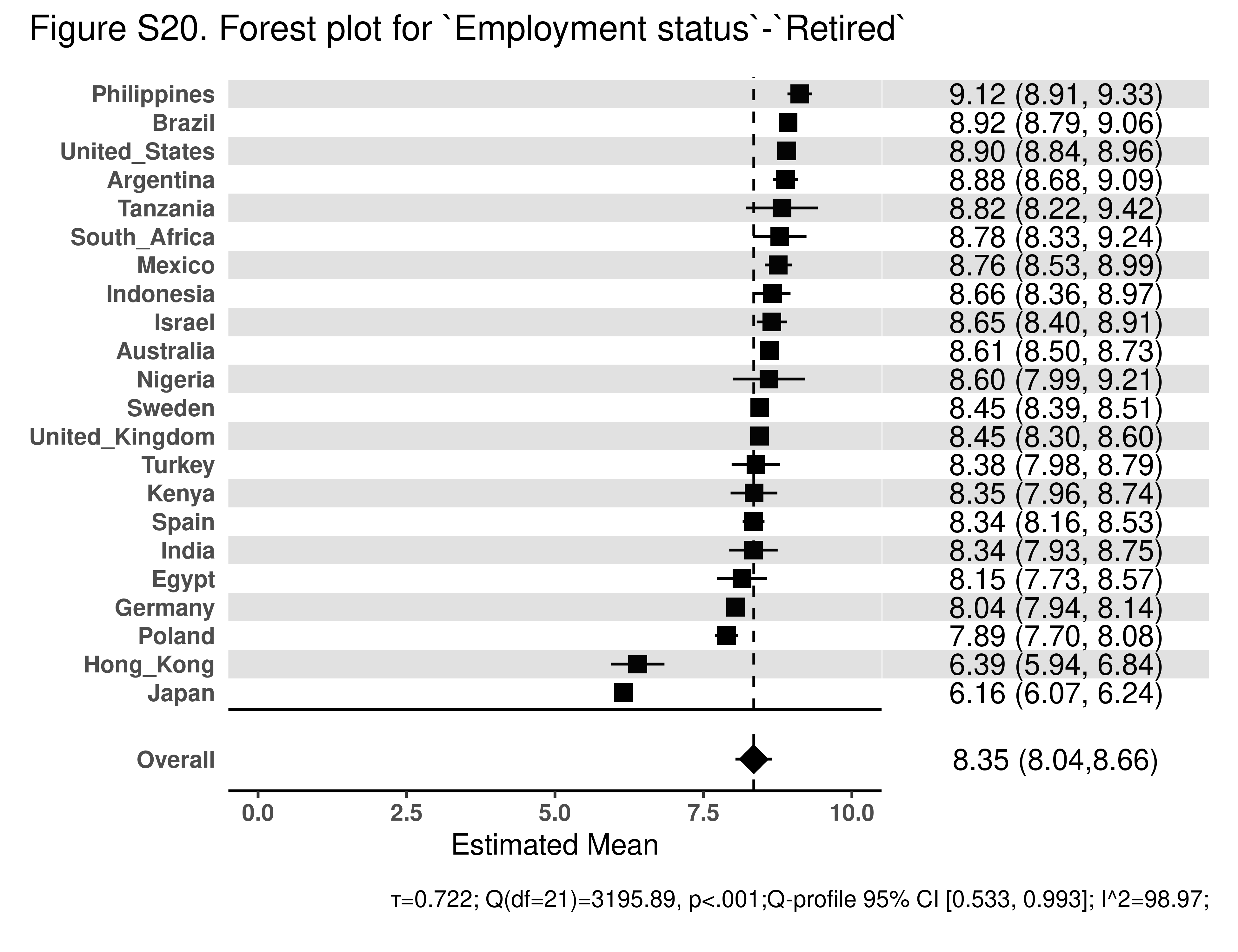


Figure S21. Forest plot for “Employment status: Student”


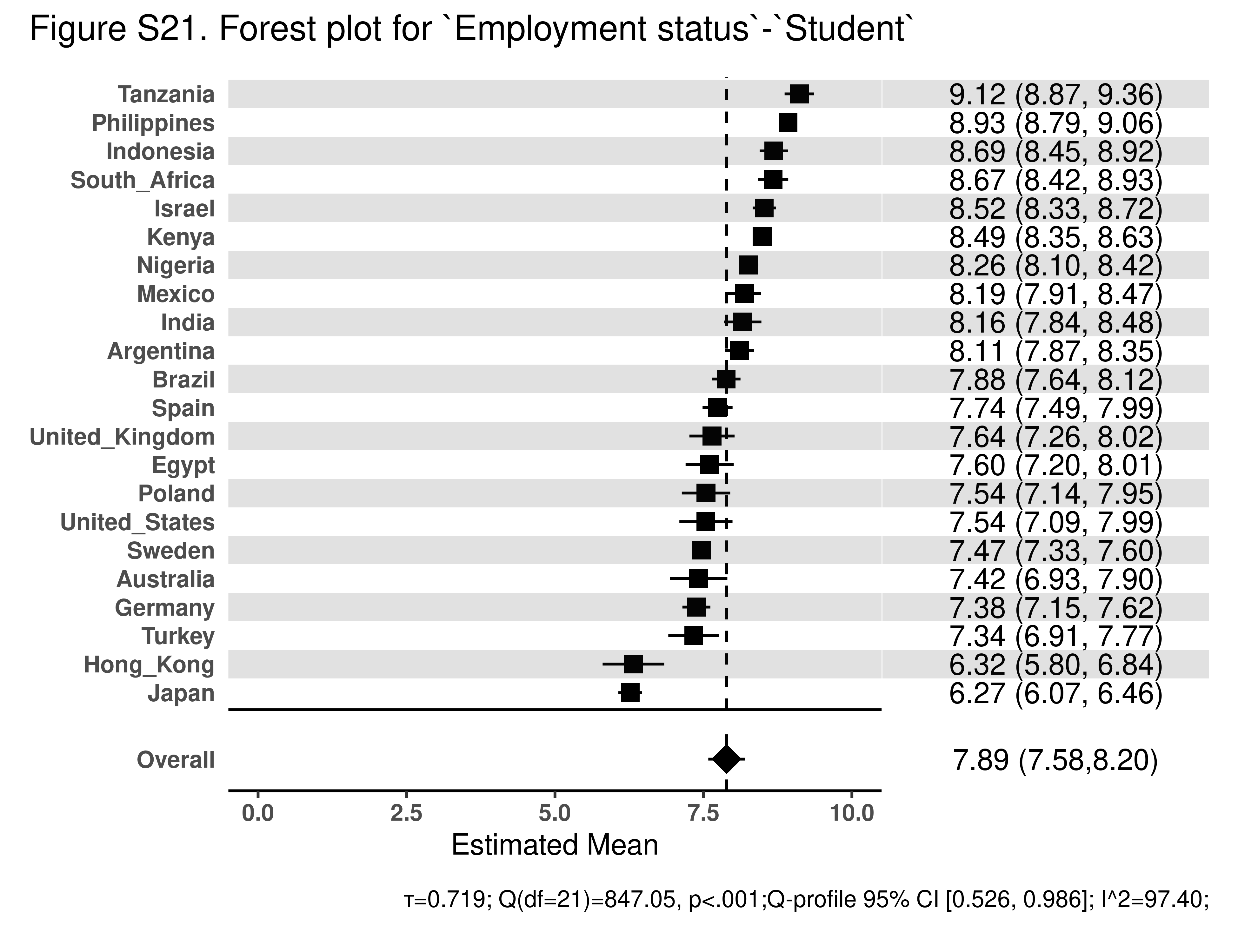


Figure S22. Forest plot for “Employment status: Homemaker”


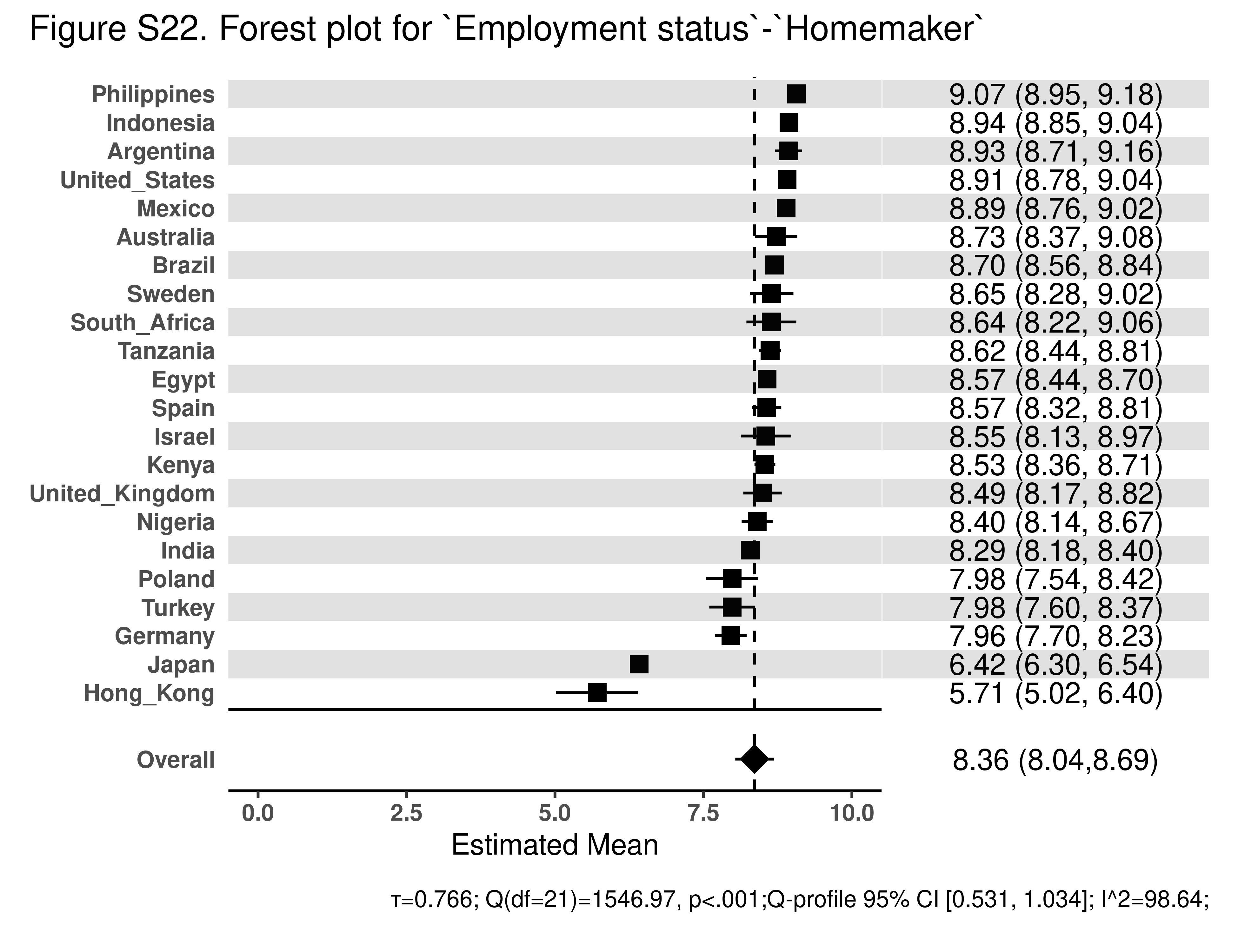


Figure S23. Forest plot for “Employment status: Unemployed and looking for a job”


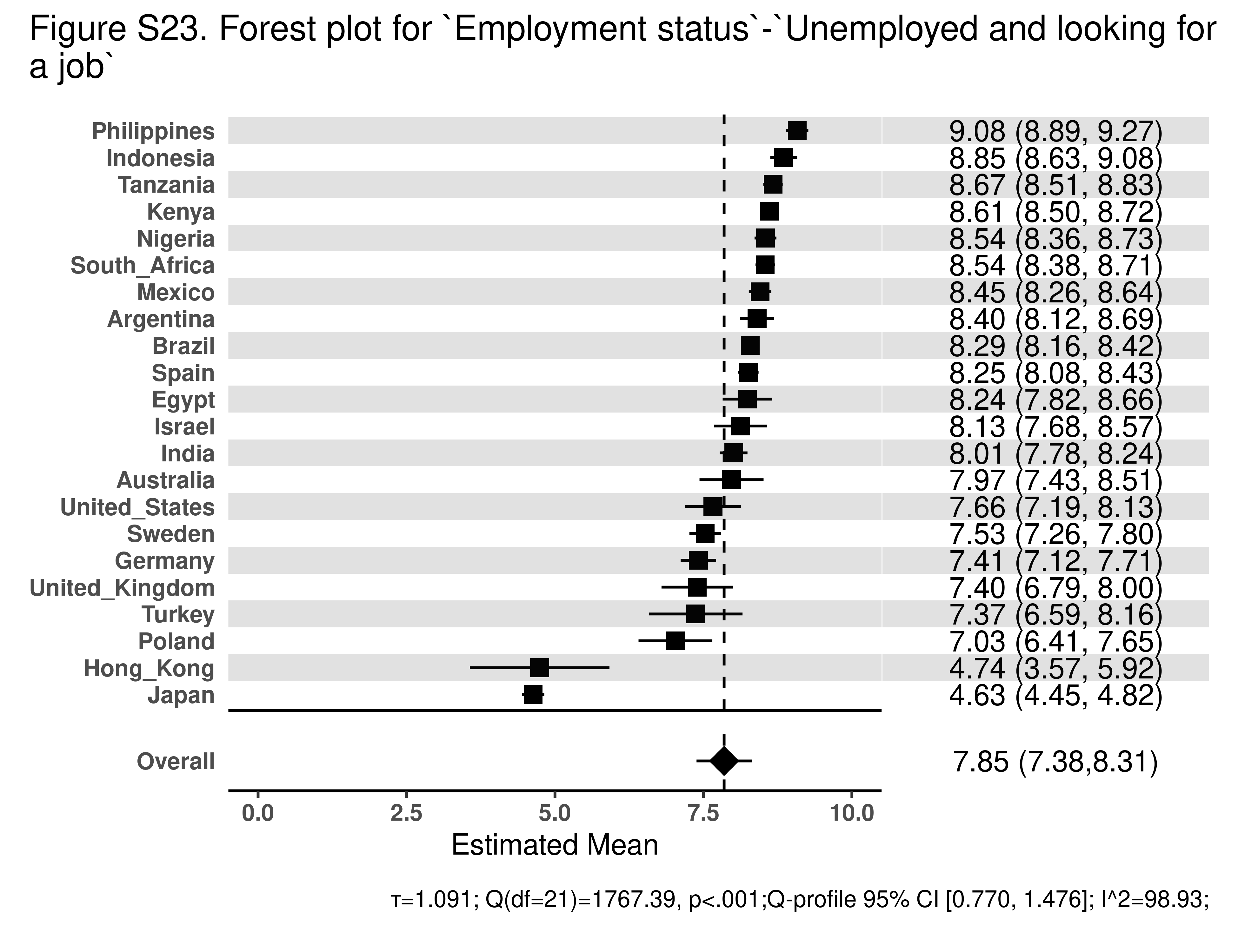


Figure S24. Forest plot for “Employment status: None of these/other”


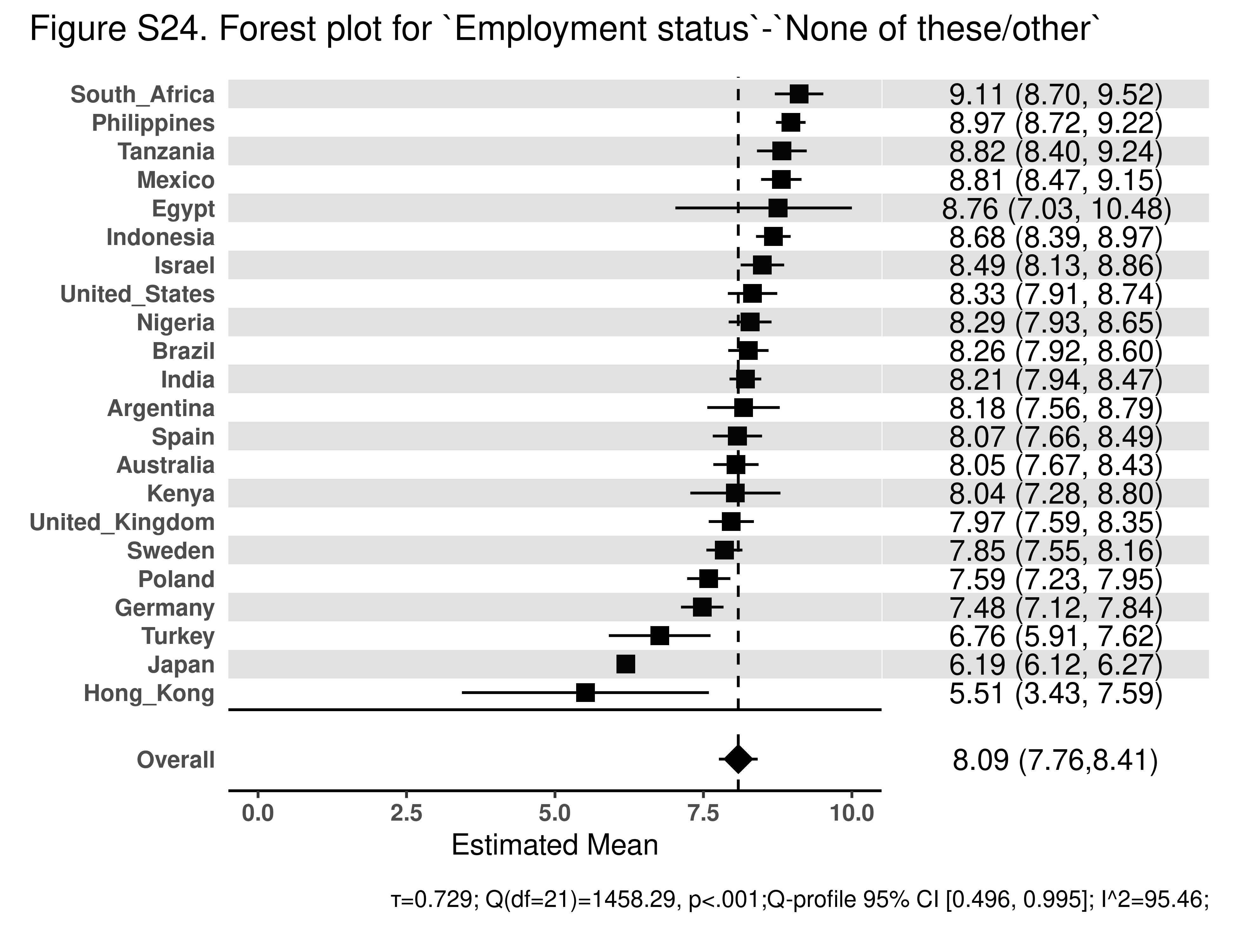


Figure S25. Forest plot for “Education: Up to 8 years”


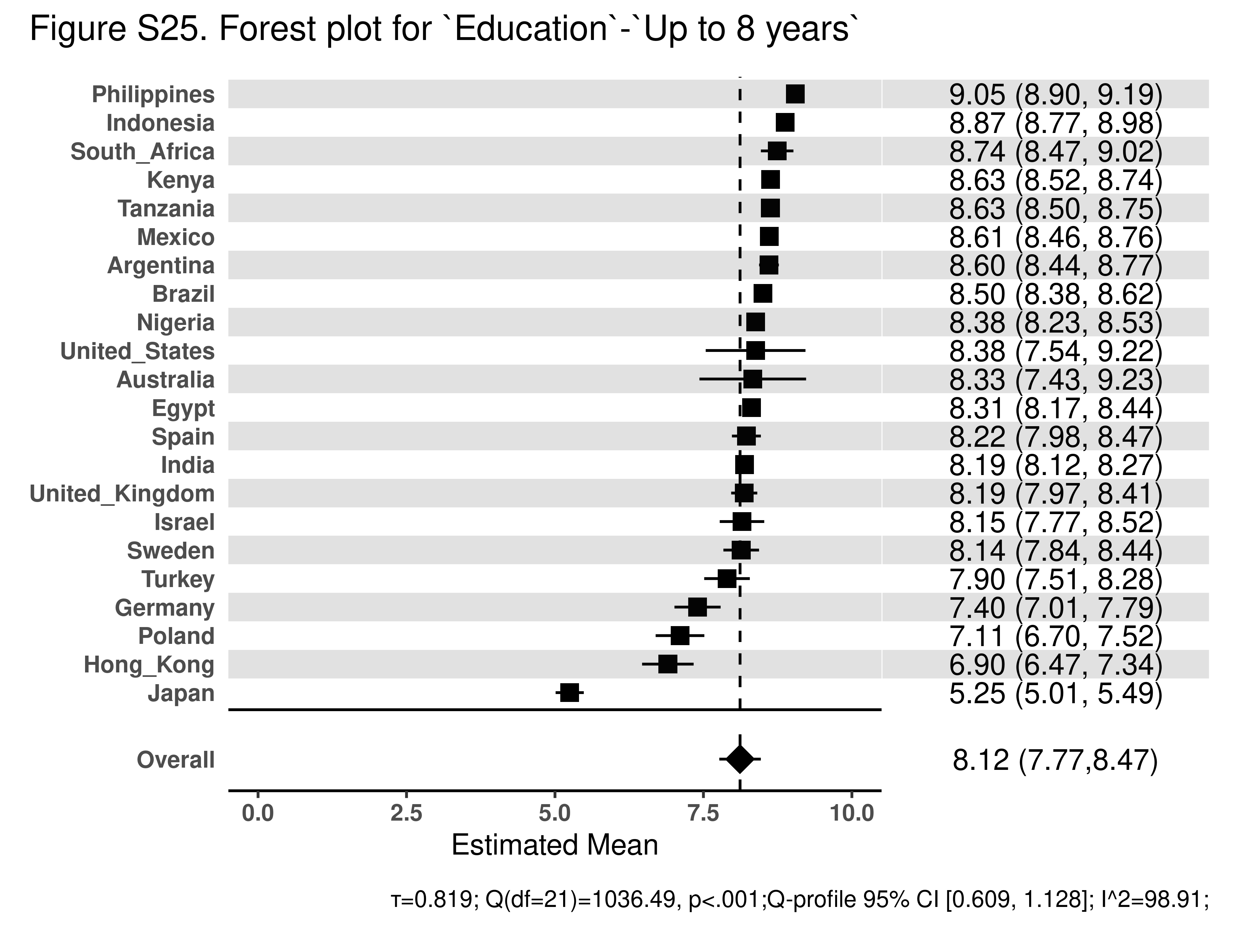


Figure S26. Forest plot for “Education: 9-15 years”


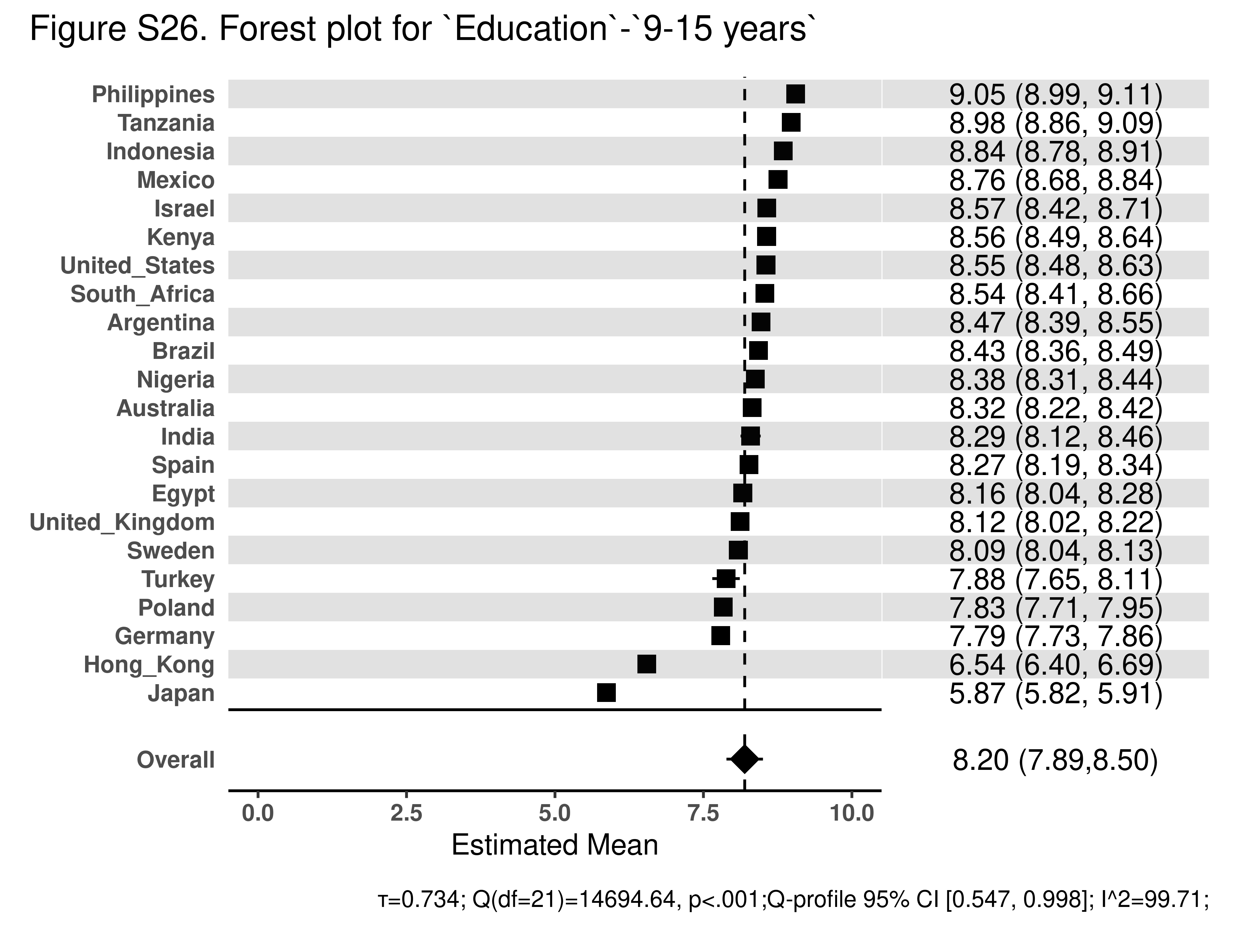


Figure S27. Forest plot for “Education: 16+ years”


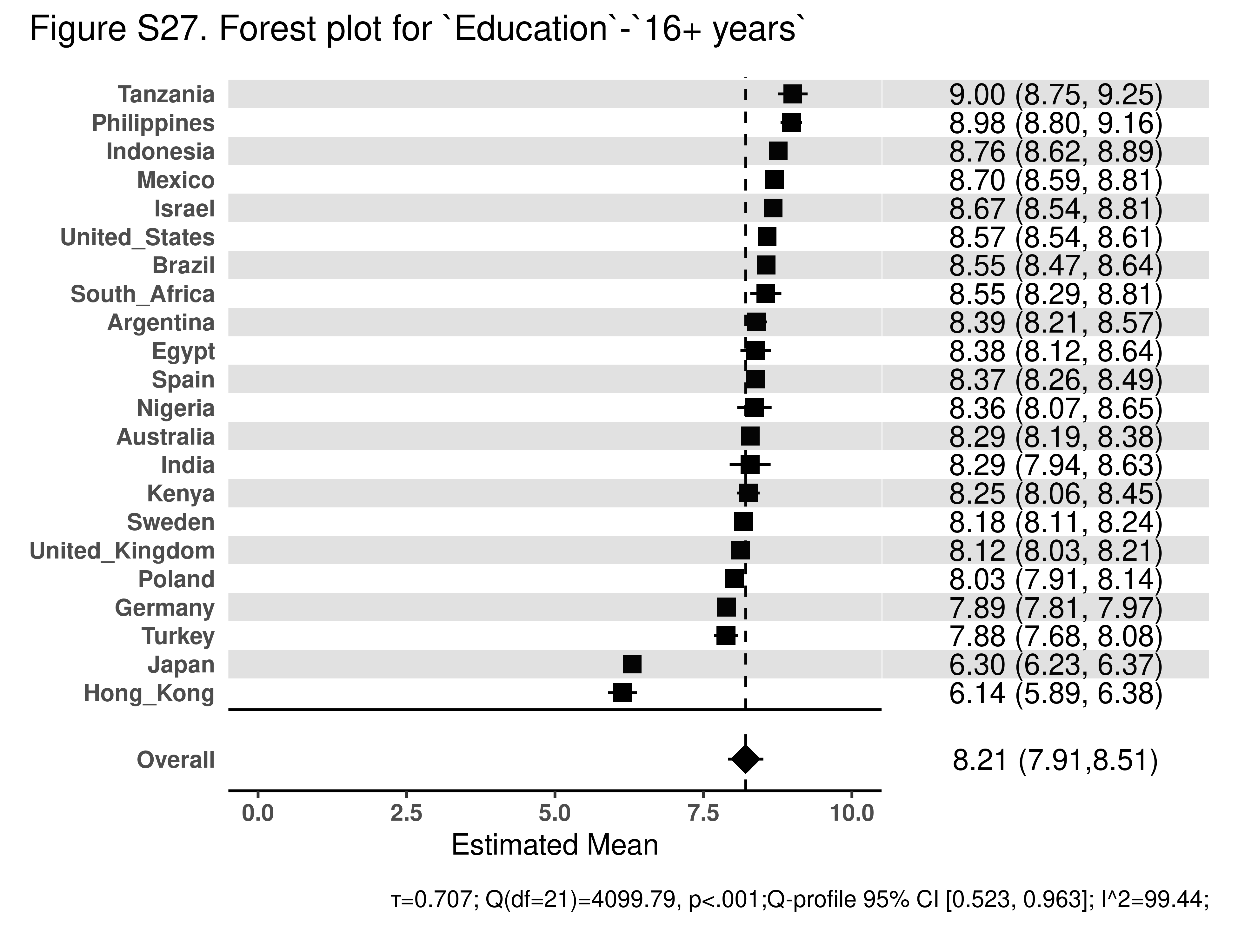


Figure S28. Forest plot for “Religious service attendance: >1x/week”


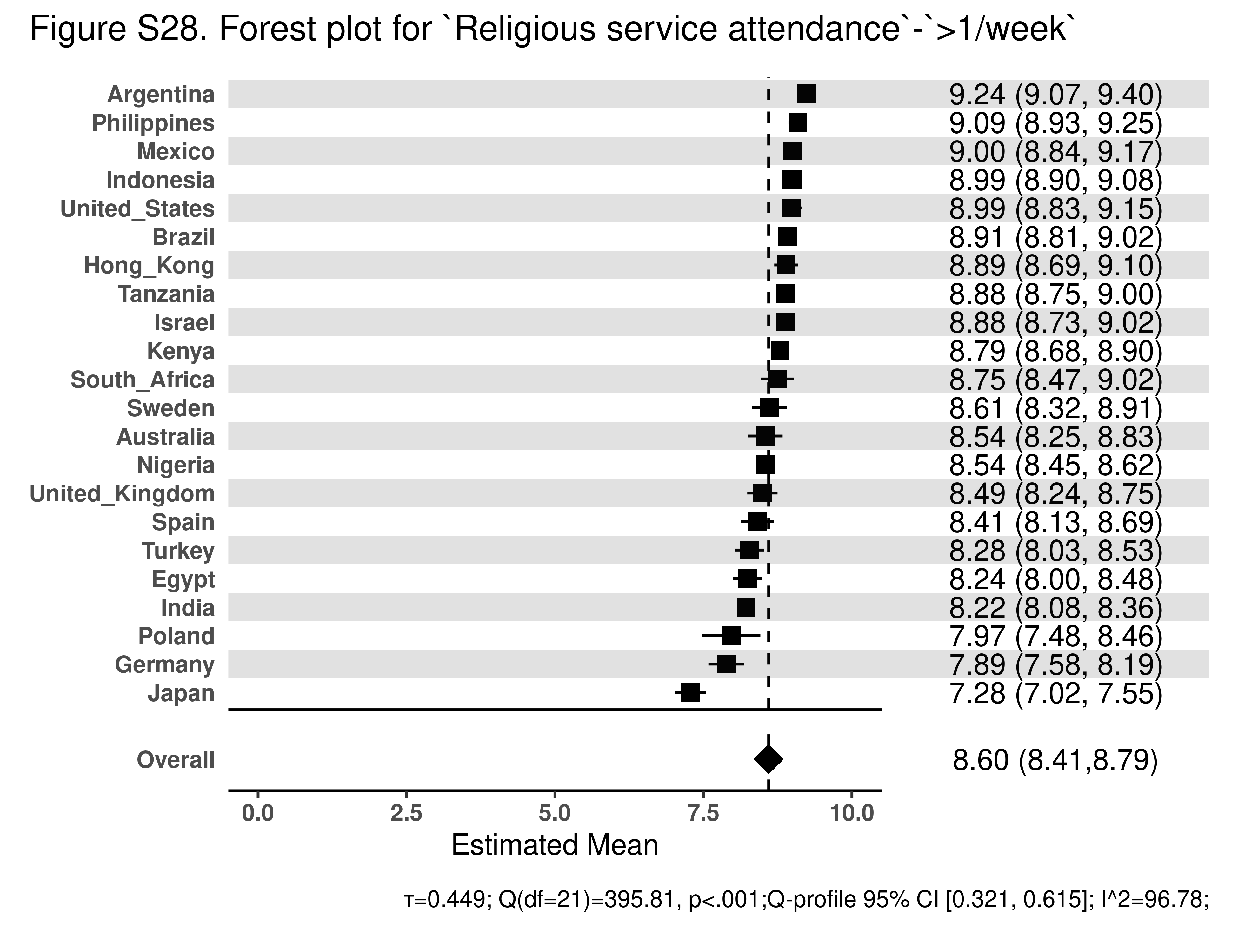


Figure S29. Forest plot for “Religious service attendance: 1x/week”


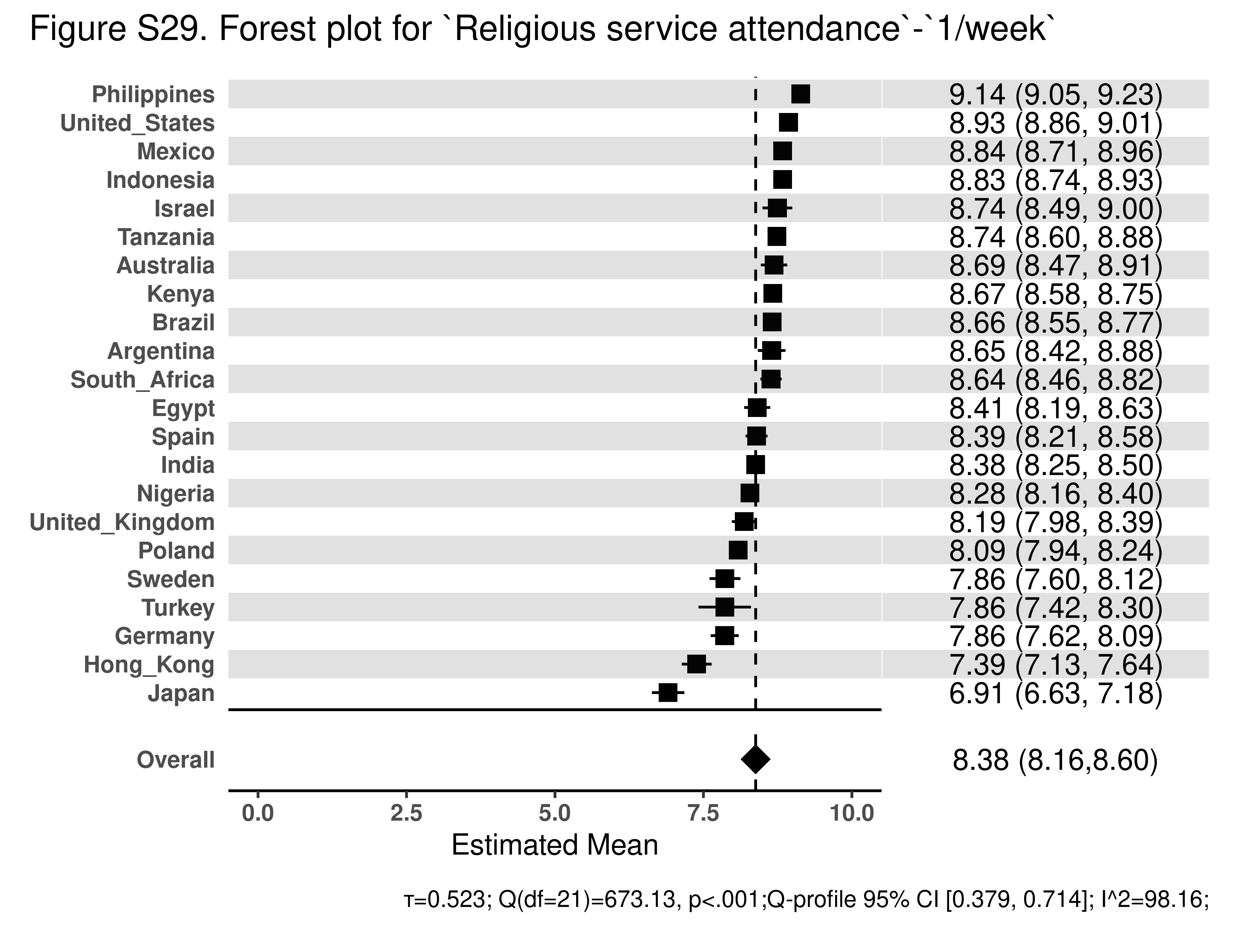


Figure S30. Forest plot for “Religious service attendance: 1-3x/month”


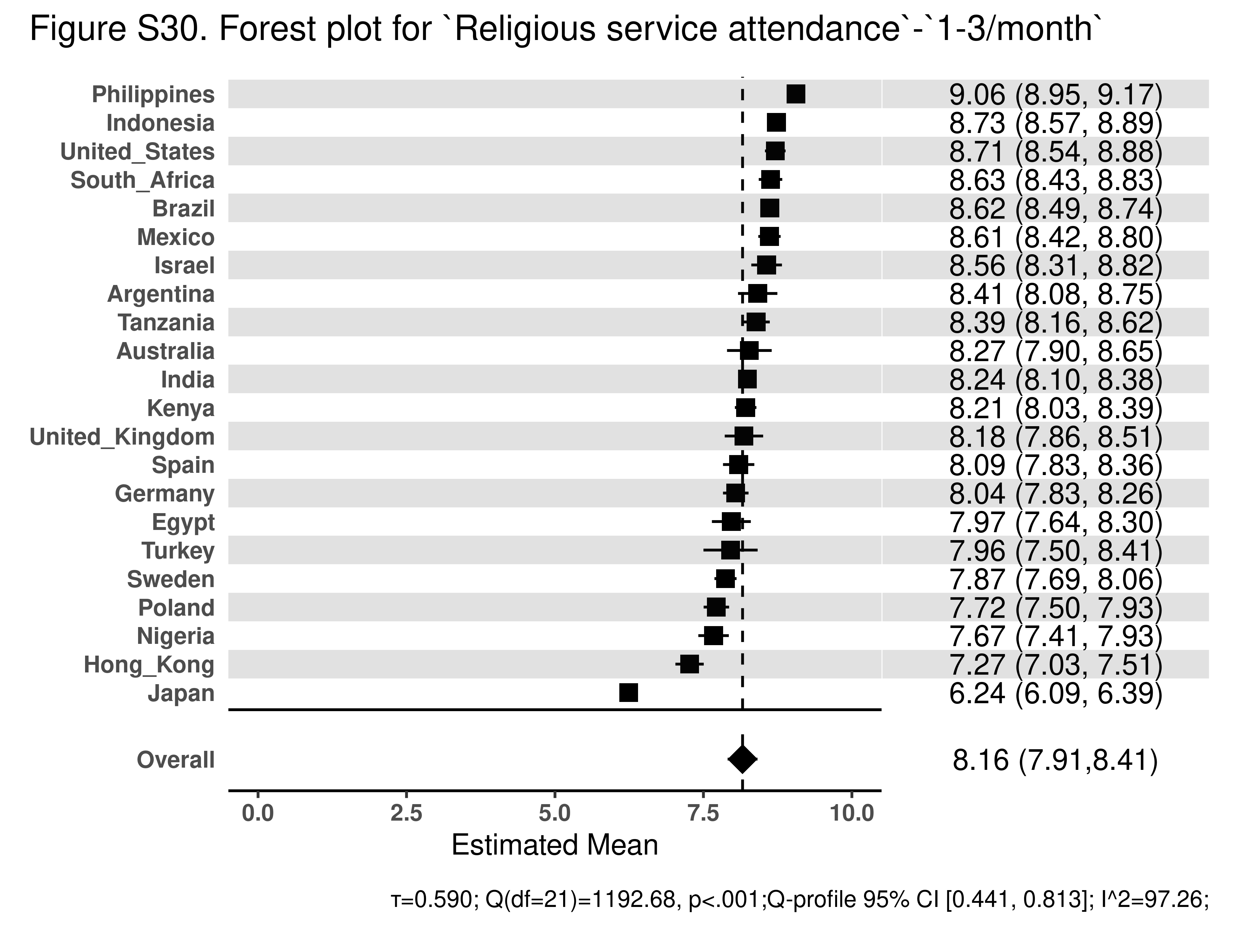


Figure S31. Forest plot for “Religious service attendance: A few times a year”


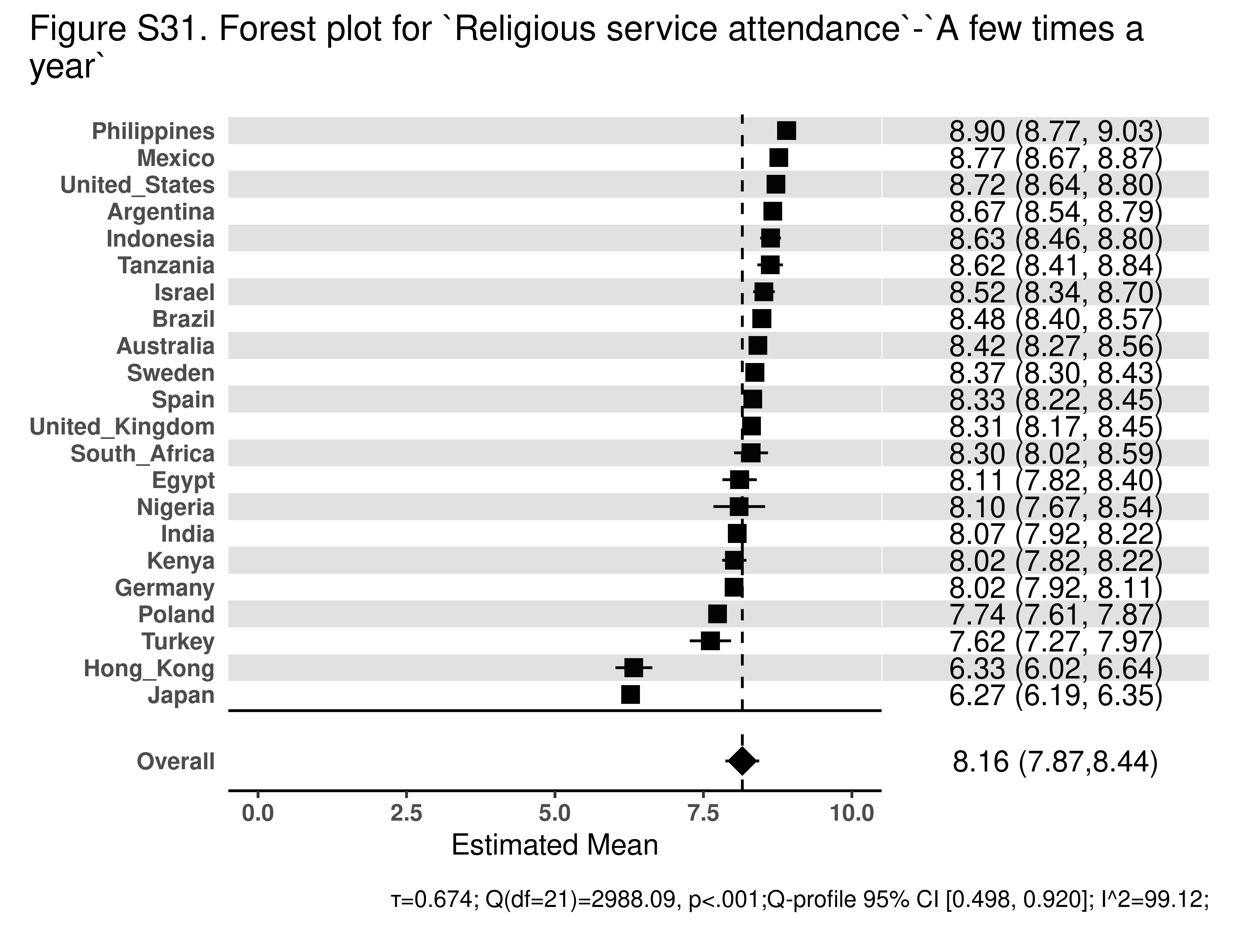


Figure S32. Forest plot for “Religious service attendance: Never”


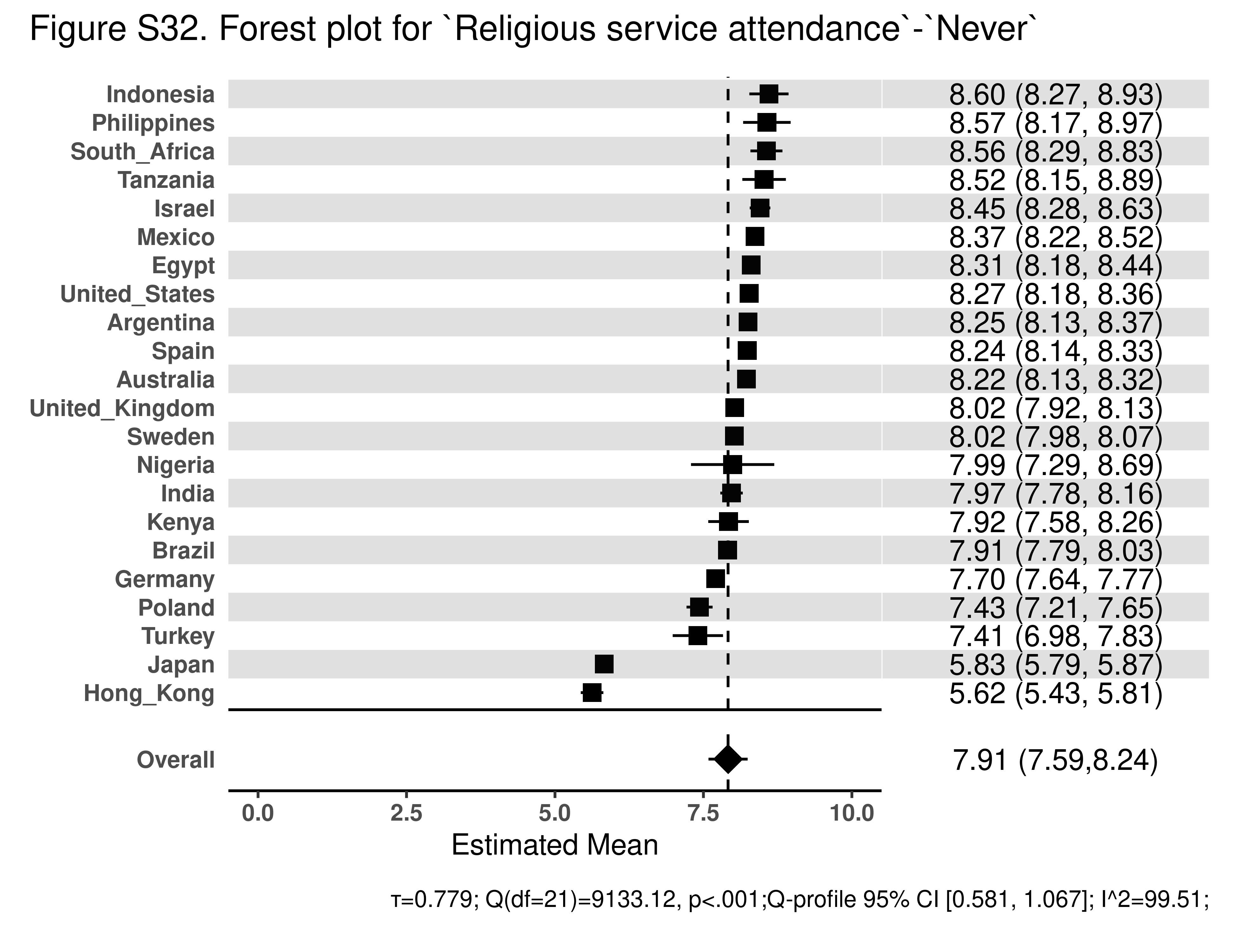


Figure S33. Forest plot for “Immigration status: Born in this country”


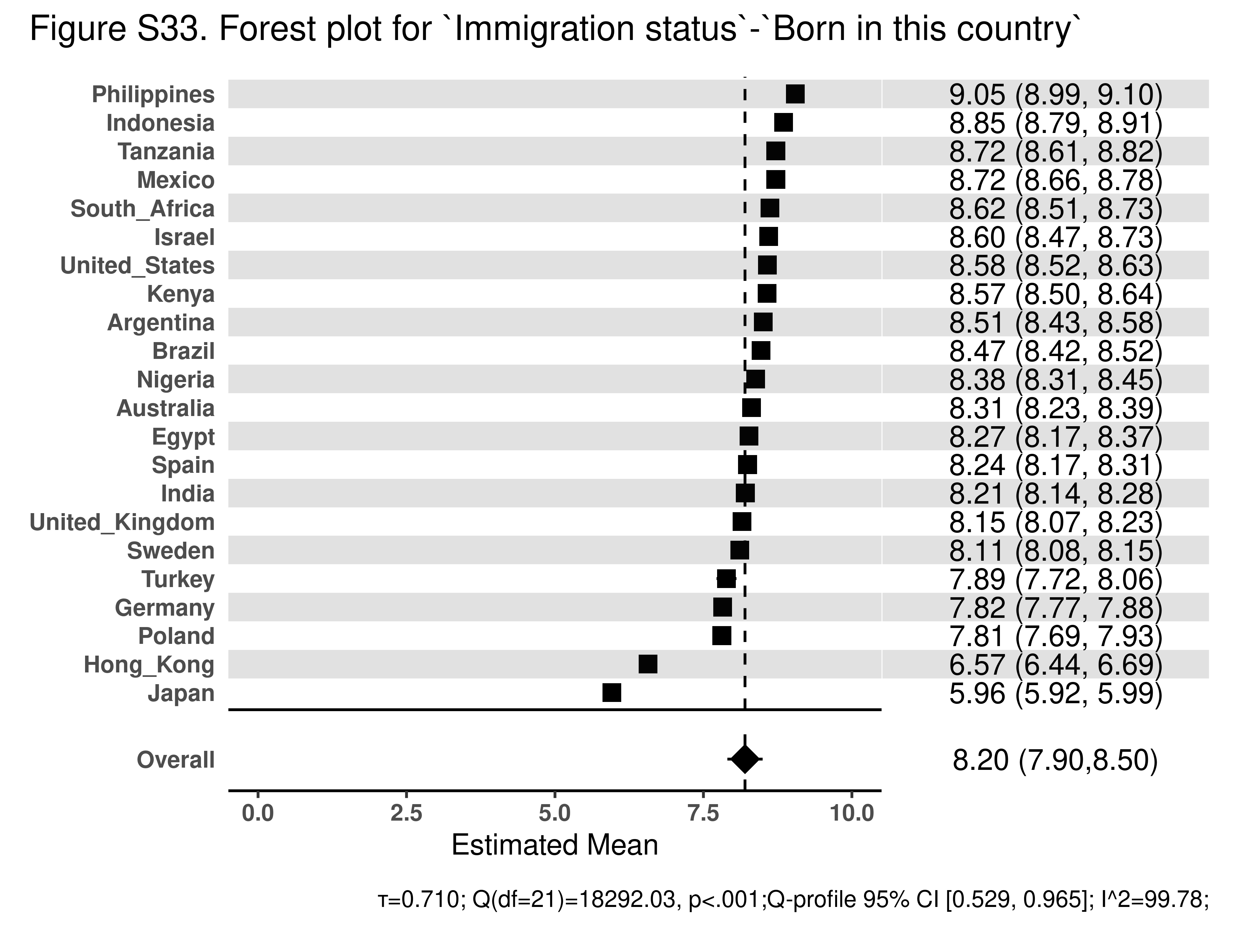


Figure S34. Forest plot for “Immigration status: Born in another country”


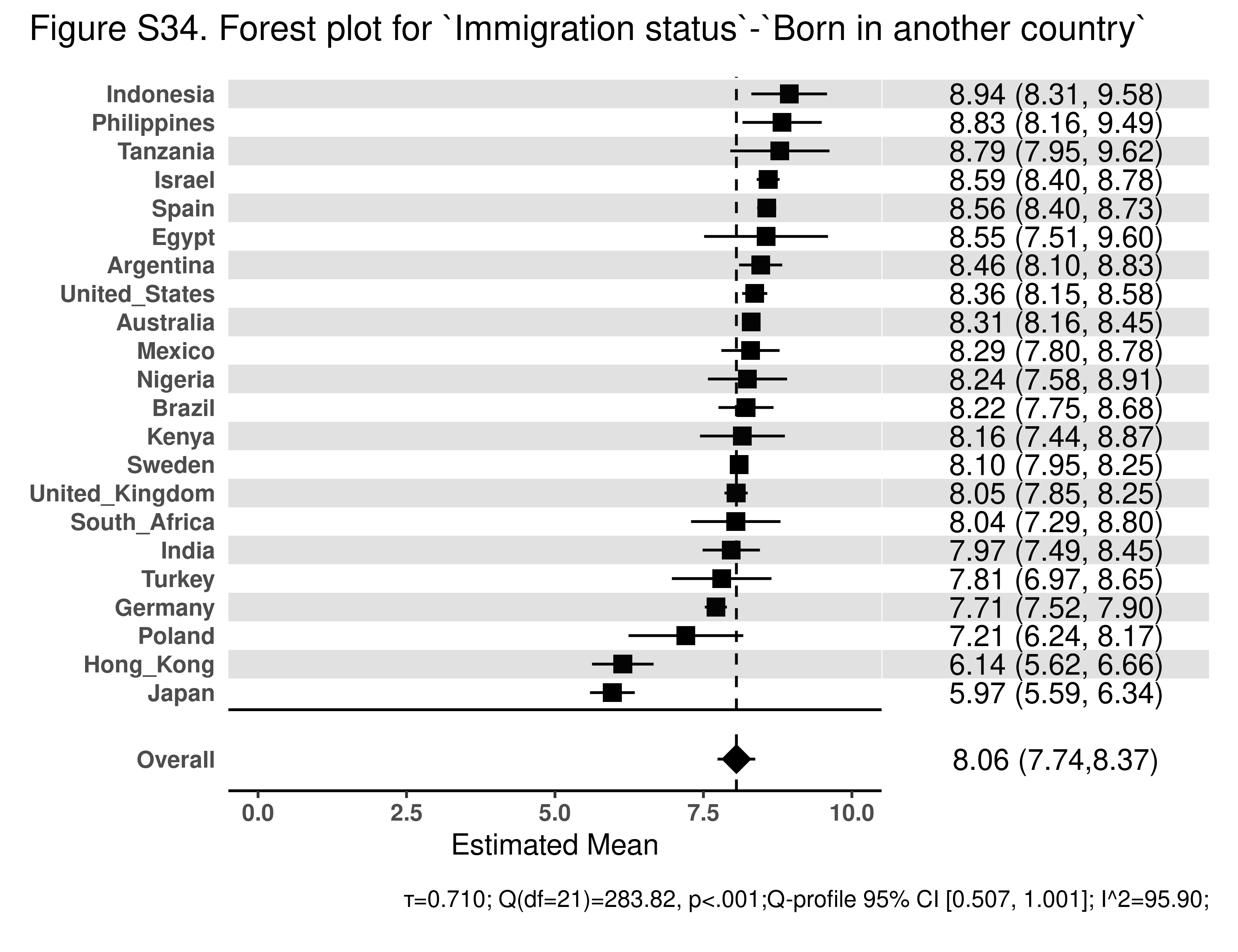


Figure S35. Forest plot for “Age group: (Ref: 18-24) 25-29”


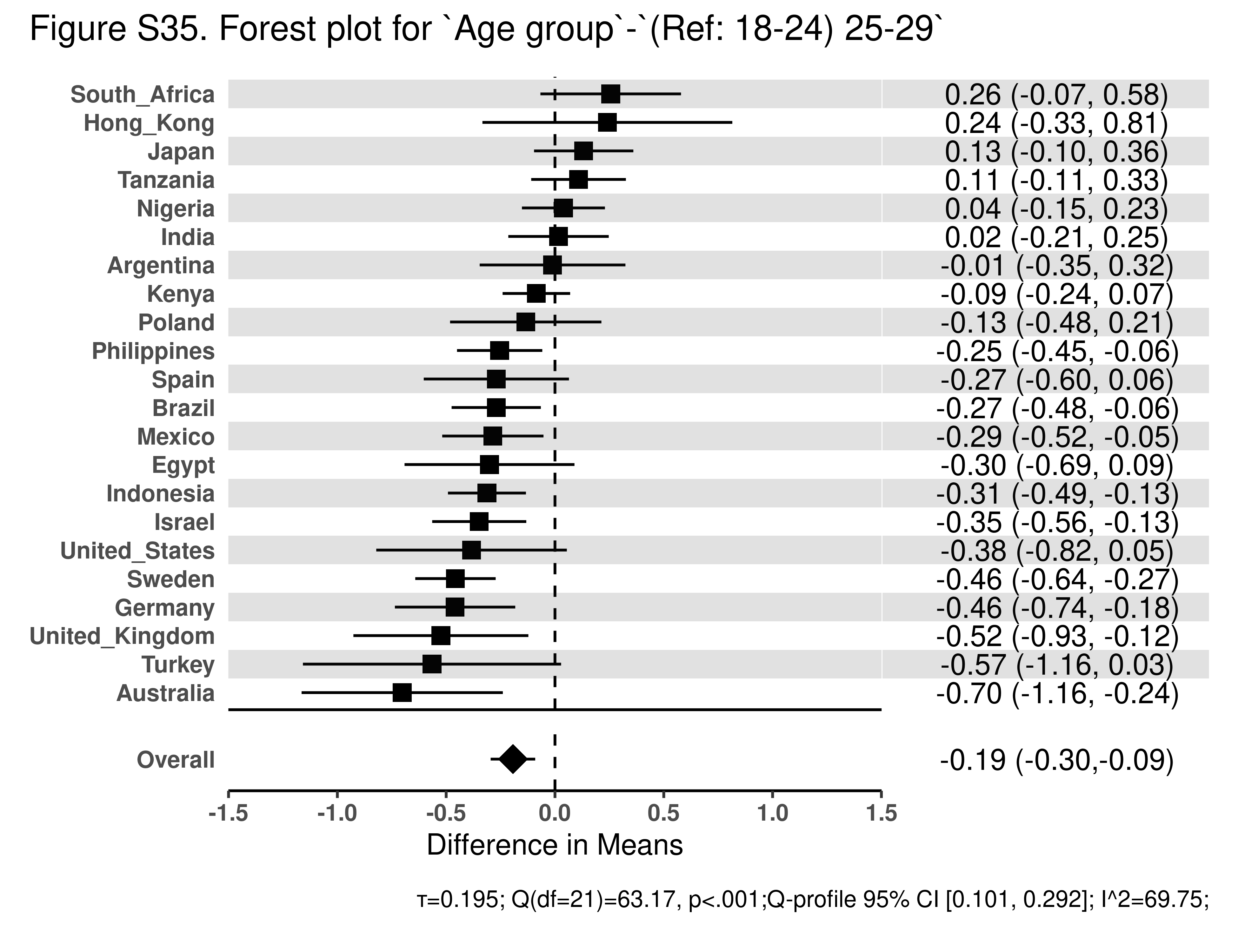


Figure S36. Forest plot for “Age group: (Ref: 18-24) 30-39”


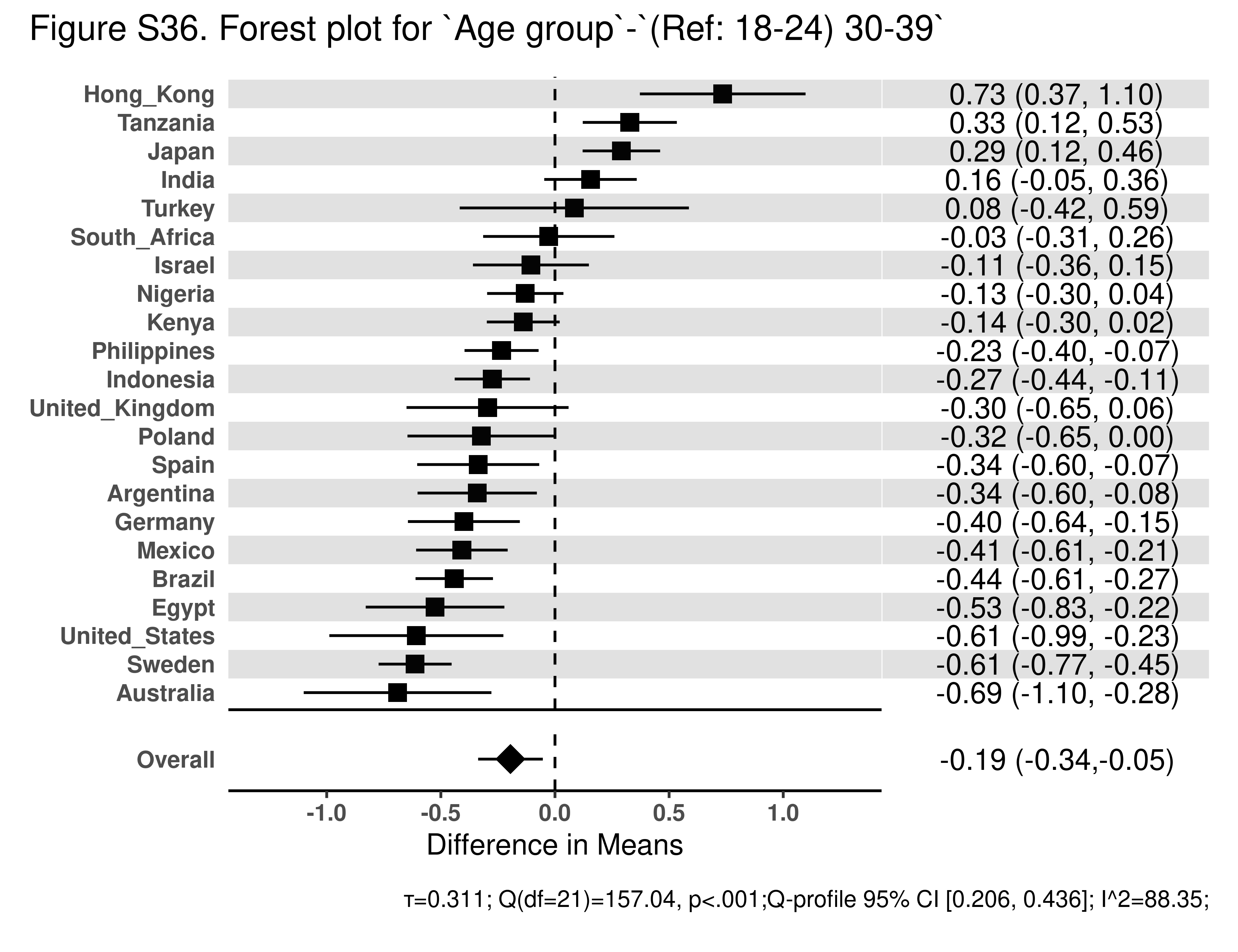


Figure S37. Forest plot for “Age group: (Ref: 18-24) 40-49”


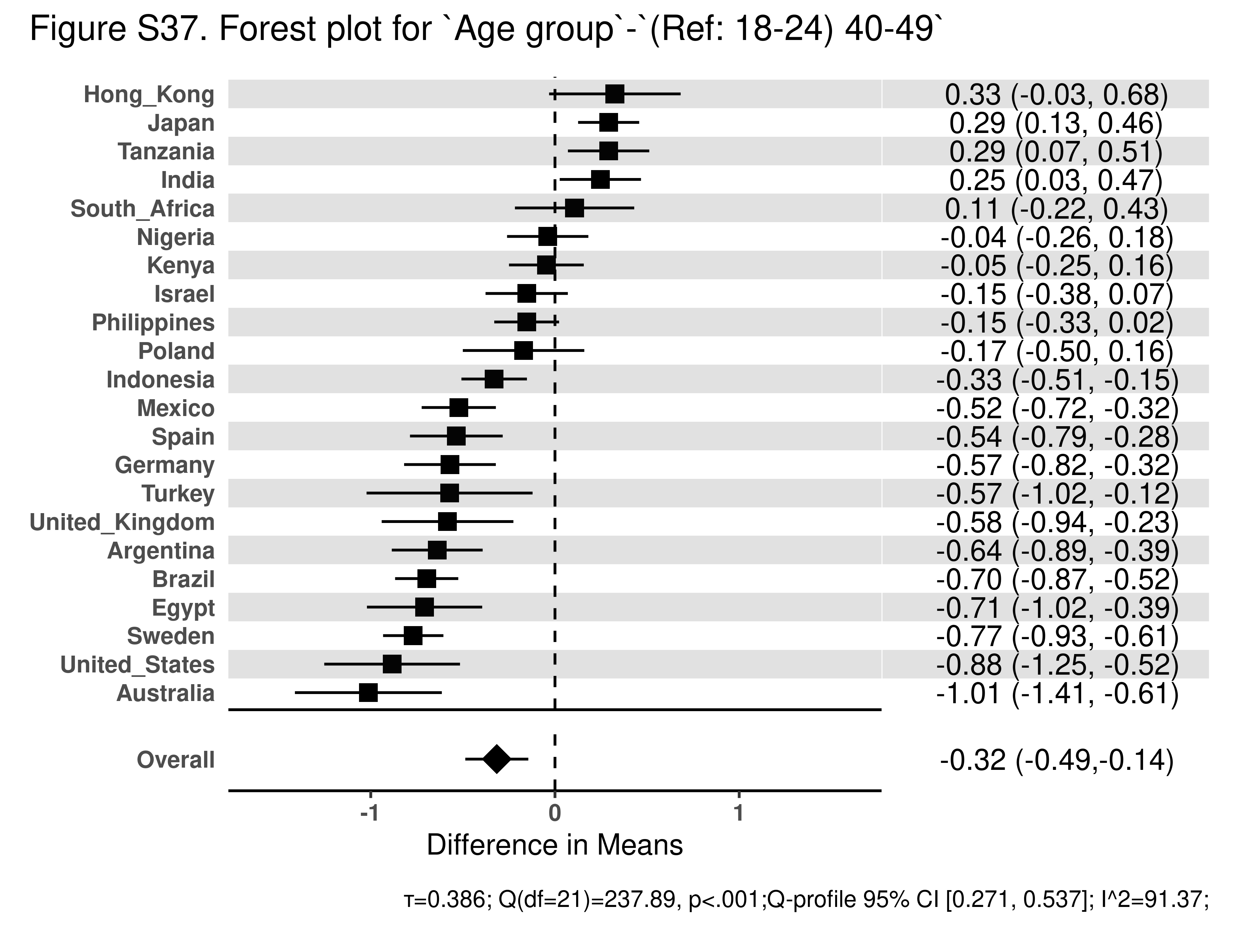


Figure S38. Forest plot for “Age group: (Ref: 18-24) 50-59”


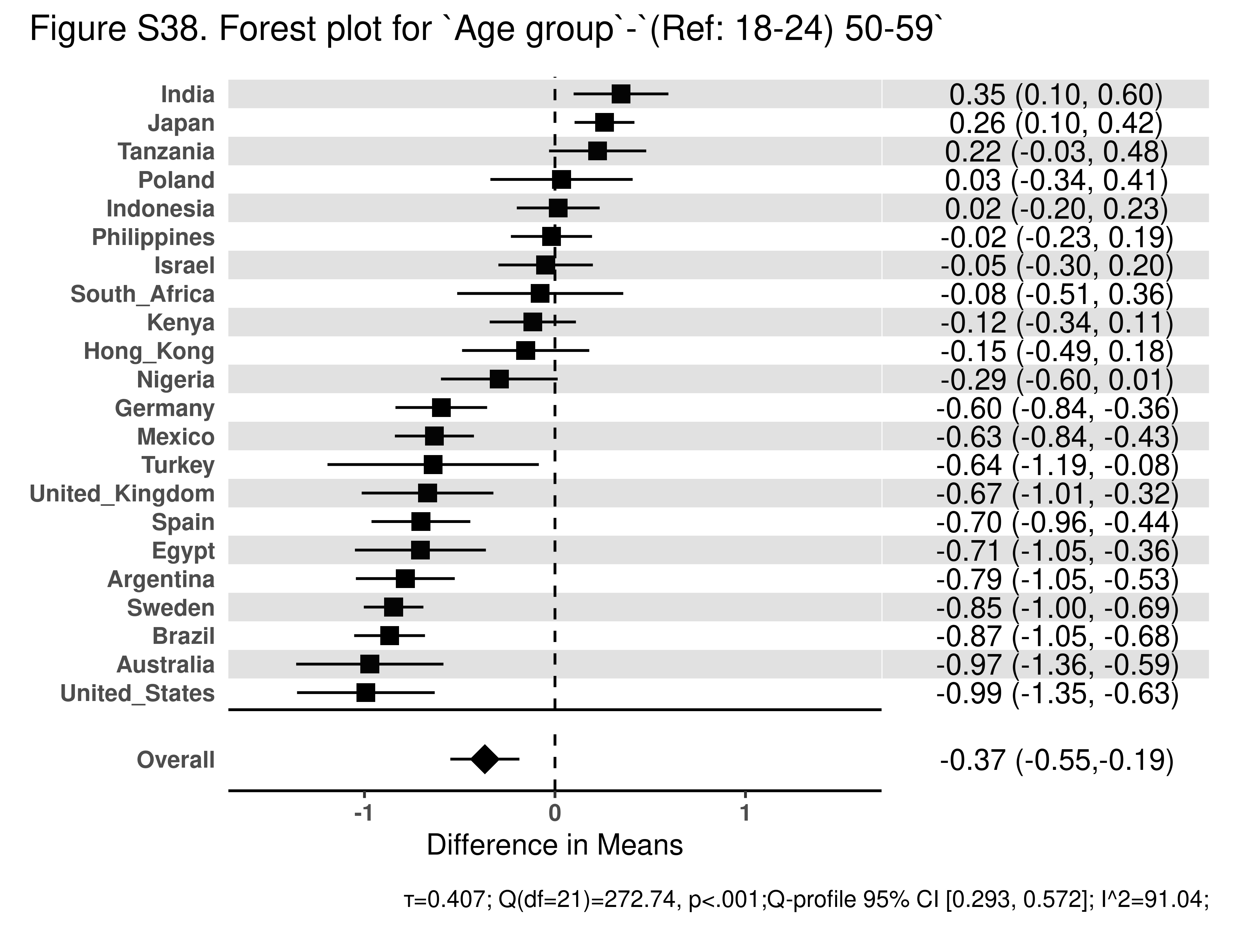


Figure S39. Forest plot for “Age group: (Ref: 18-24) 60-69”


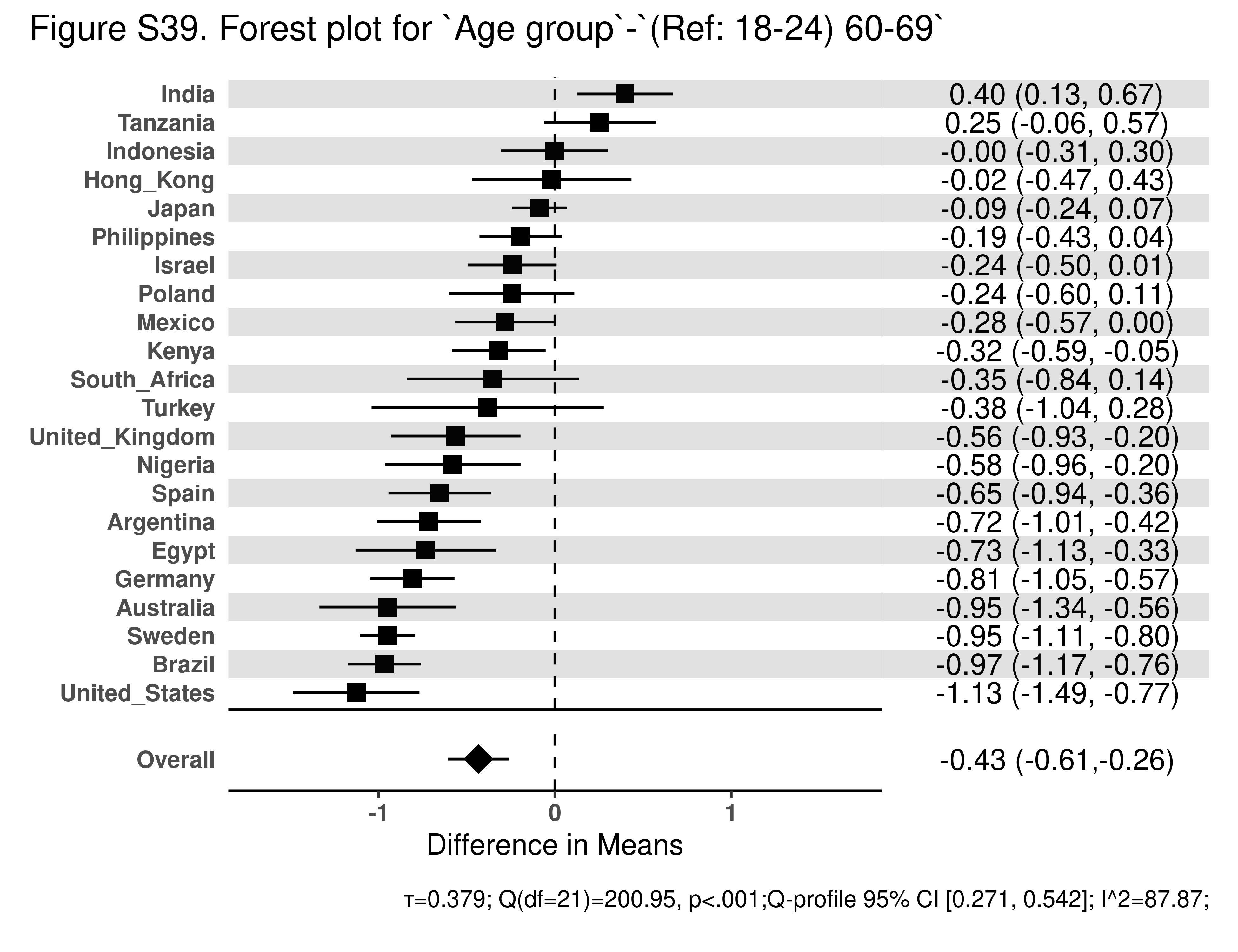


Figure S40. Forest plot for “Age group: (Ref: 18-24) 70-79”


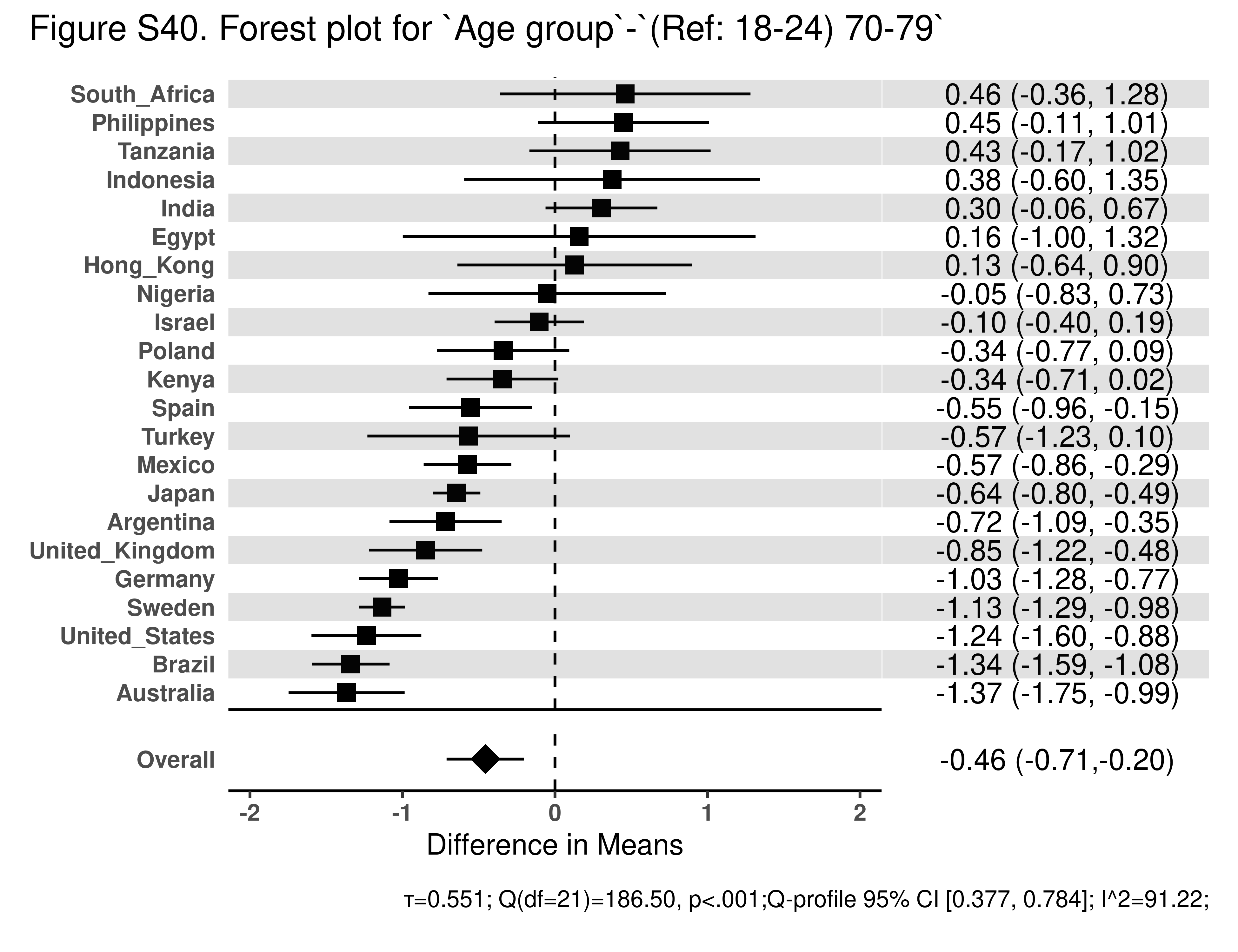


Figure S41. Forest plot for “Age group: (Ref: 18-24) 80 or older”


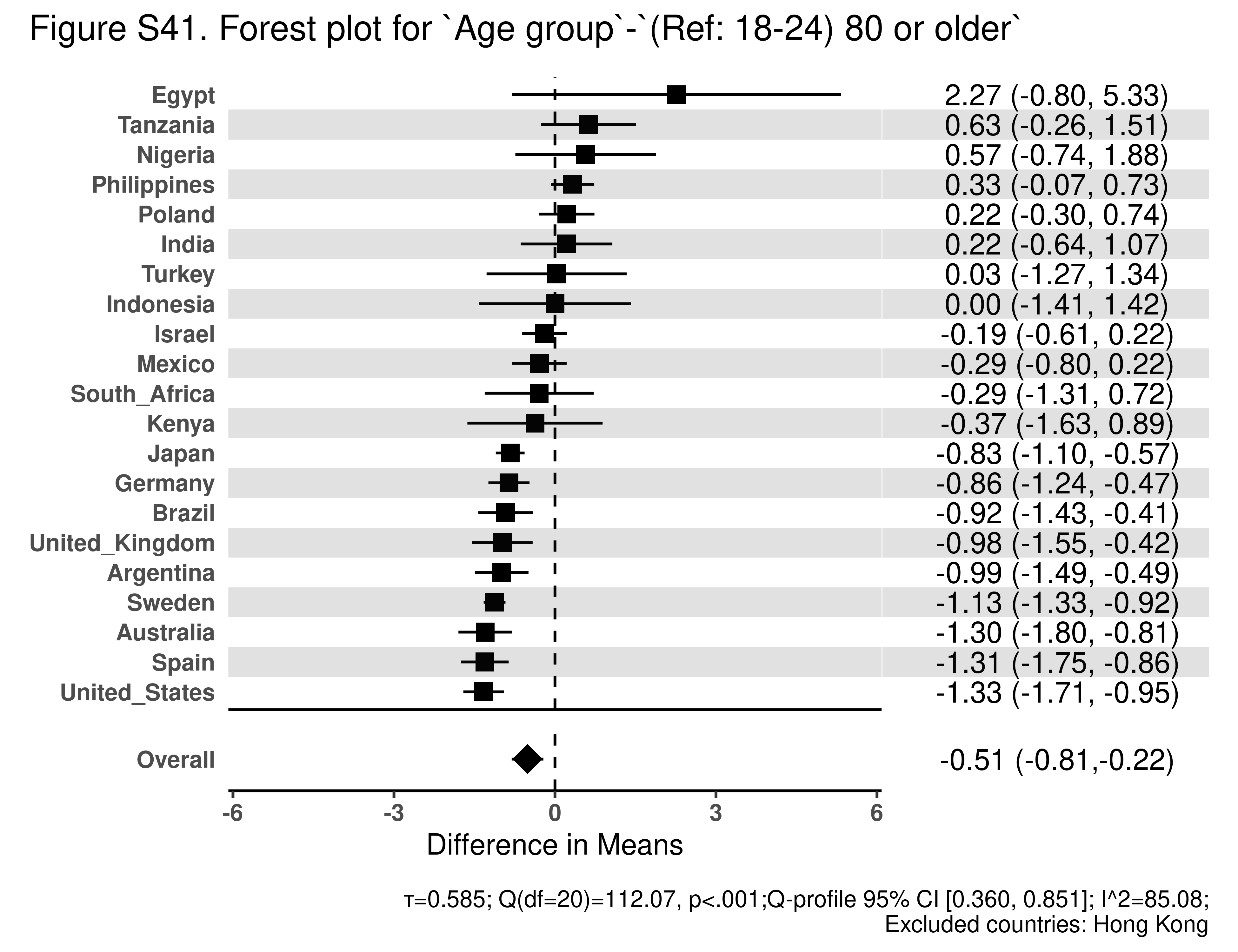


Figure S42. Forest plot for “Age group: (Ref: 25-29) 30-39”


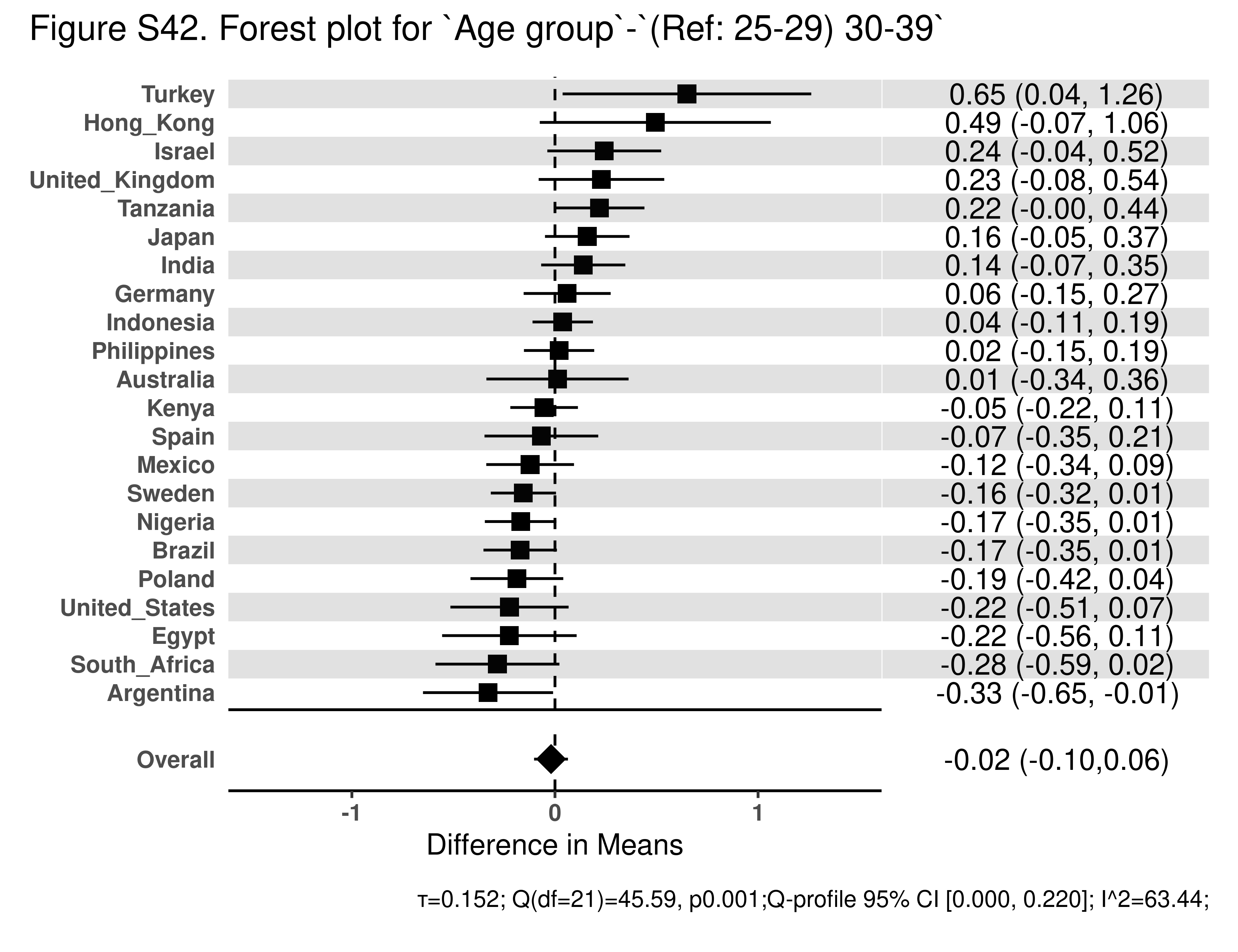


Figure S43. Forest plot for “Age group: (Ref: 25-29) 40-49”


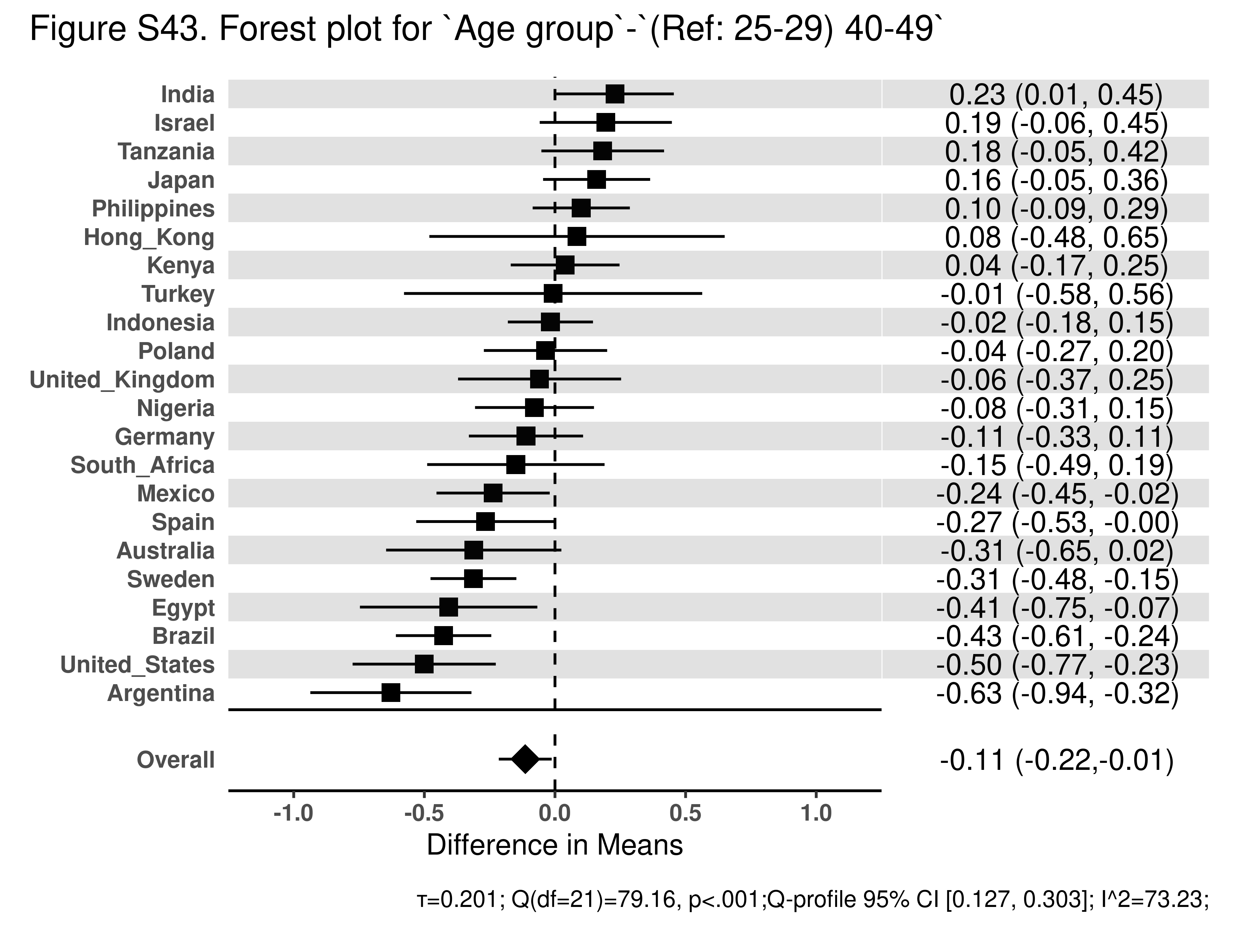


Figure S44. Forest plot for “Age group: (Ref: 25-29) 50-59”


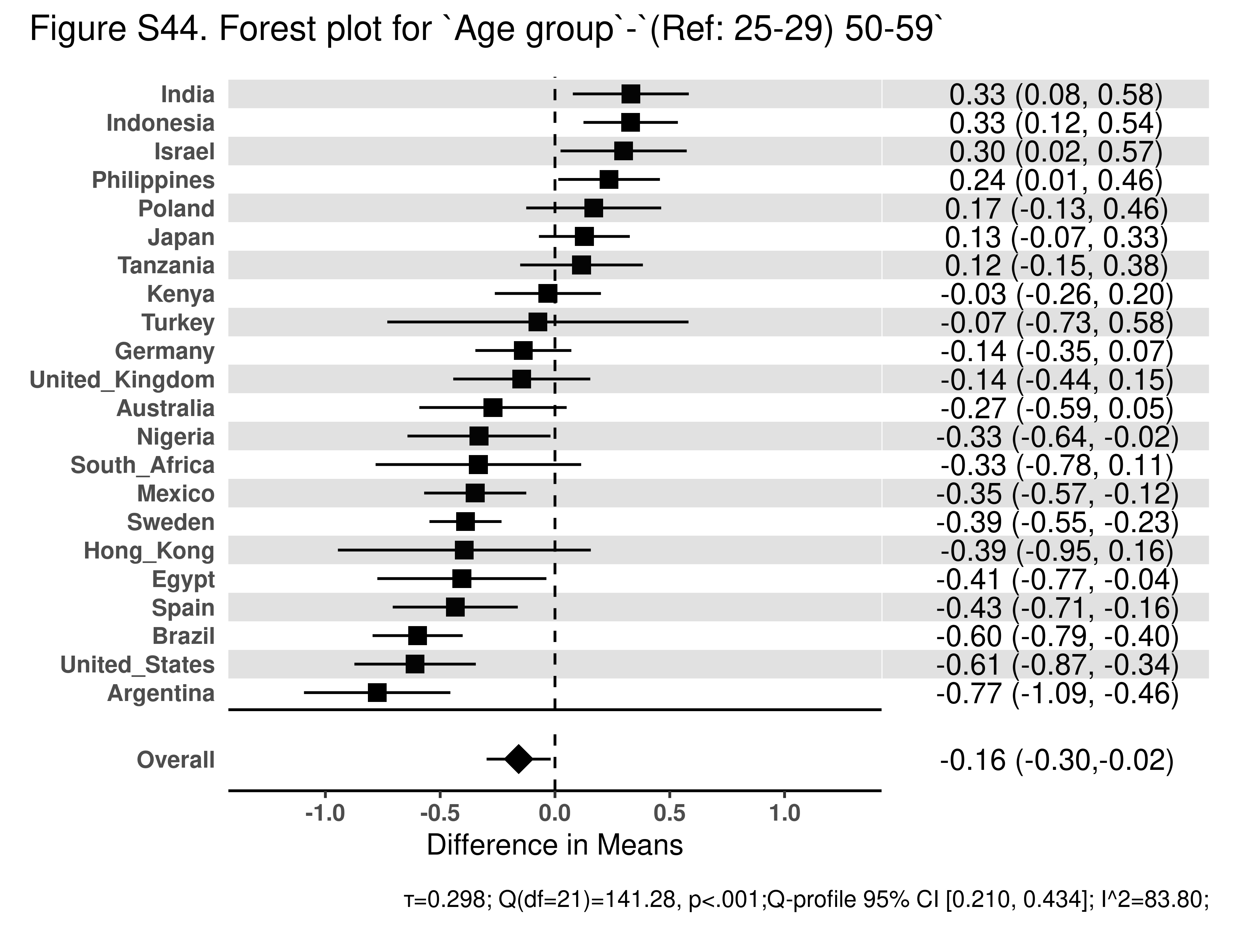


Figure S45. Forest plot for “Age group: (Ref: 25-29) 60-69”


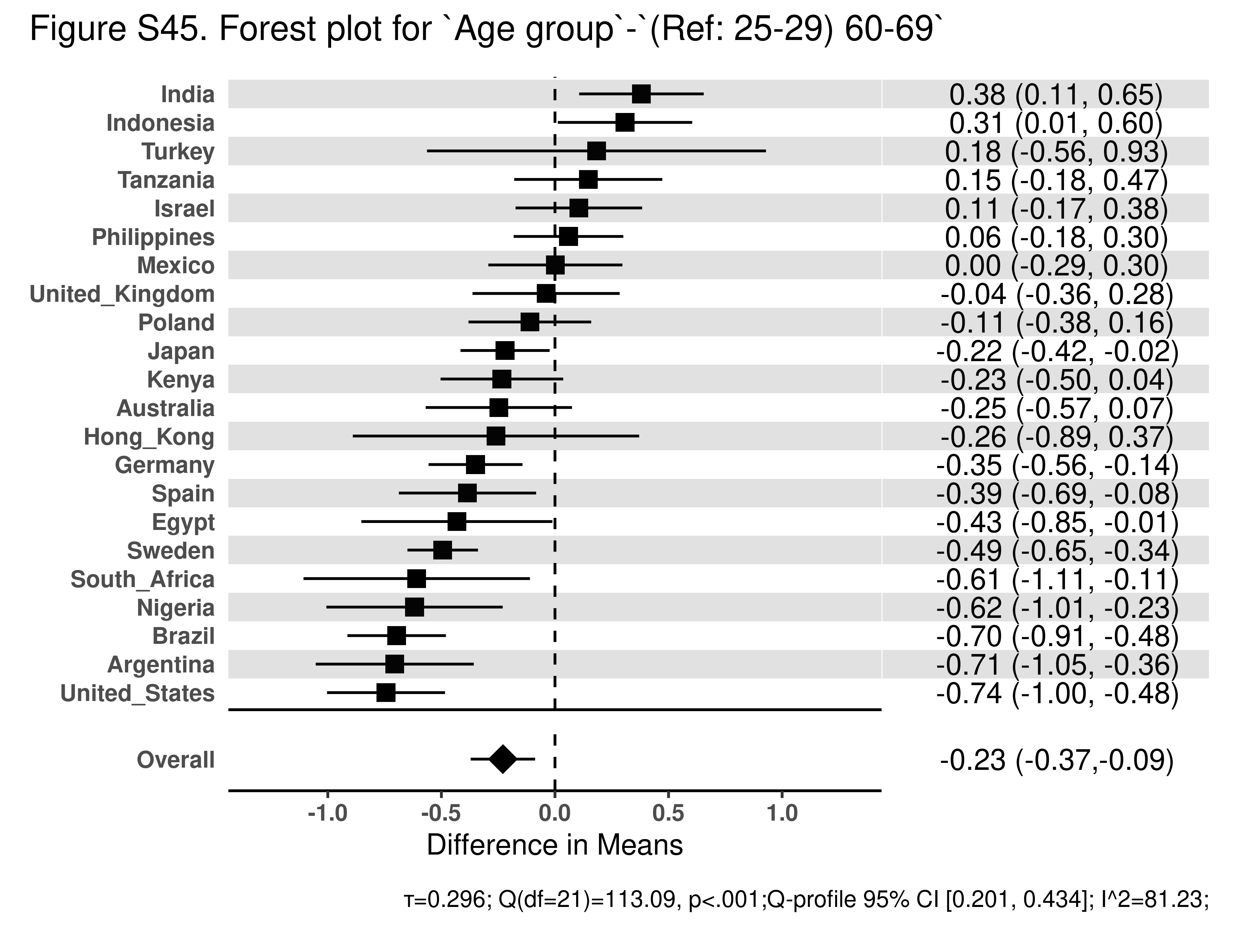


Figure S46. Forest plot for “Age group: (Ref: 25-29) 70-79”


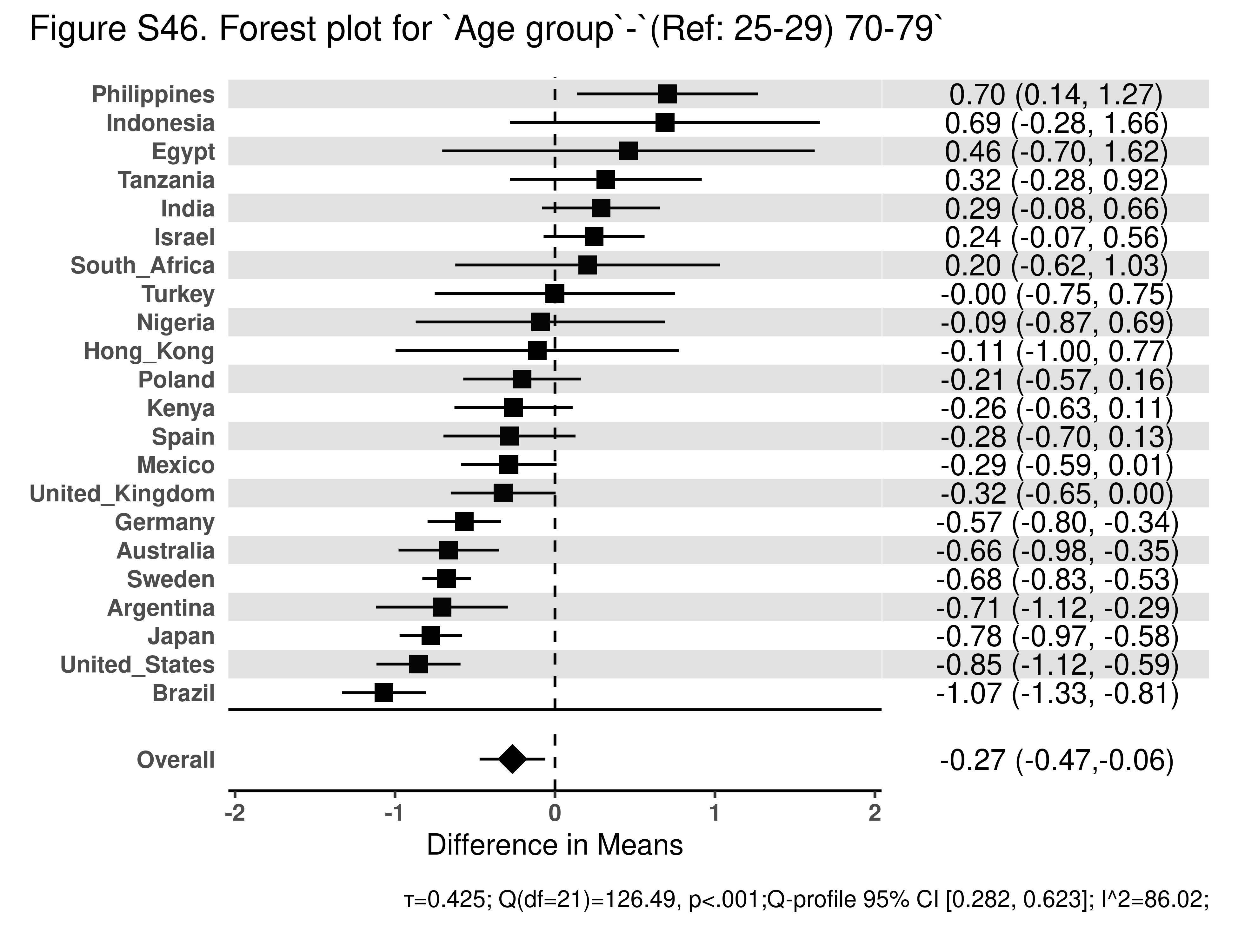


Figure S47. Forest plot for “Age group: (Ref: 25-29) 80 or older”


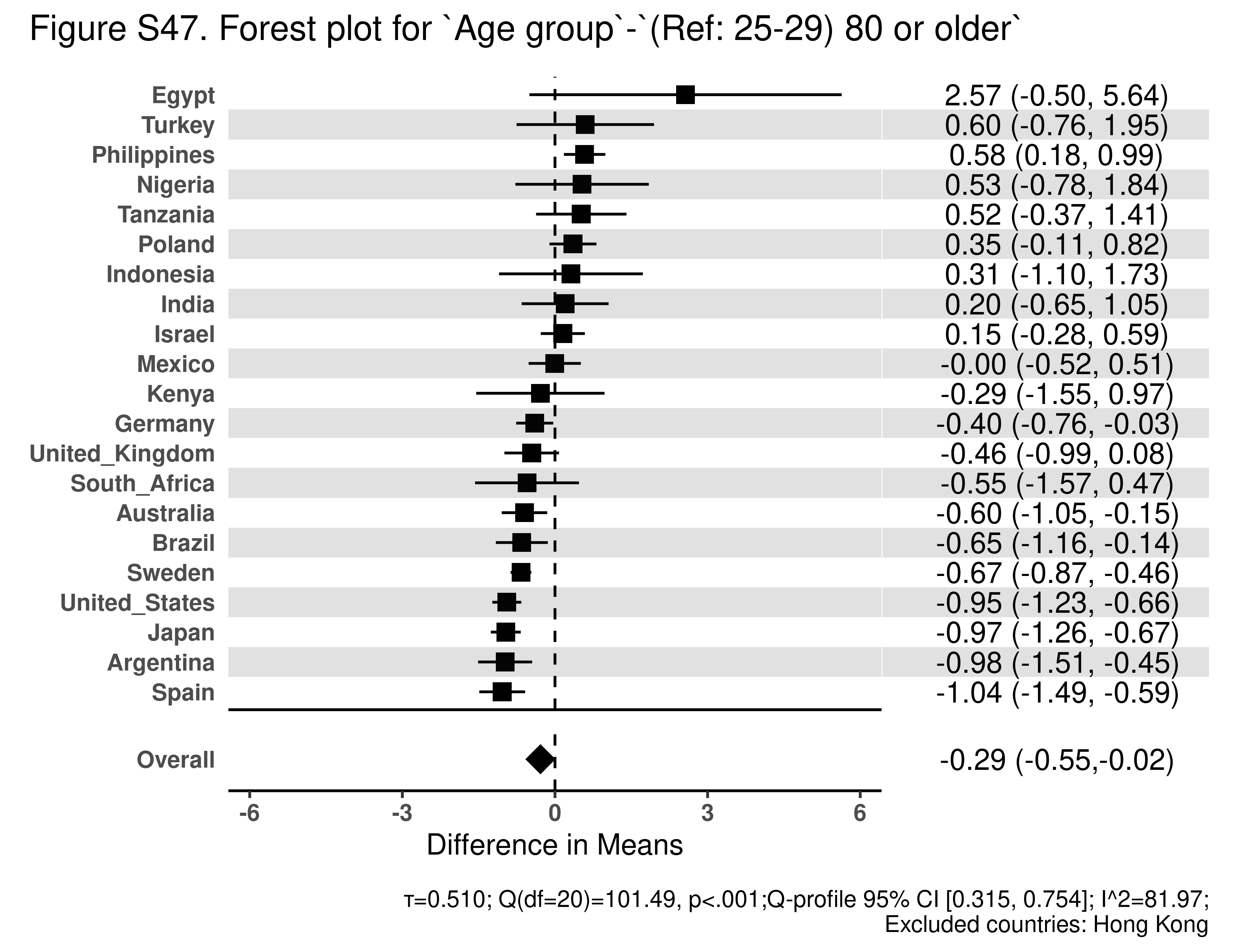


Figure S48. Forest plot for “Age group: (Ref: 30-39) 40-49”


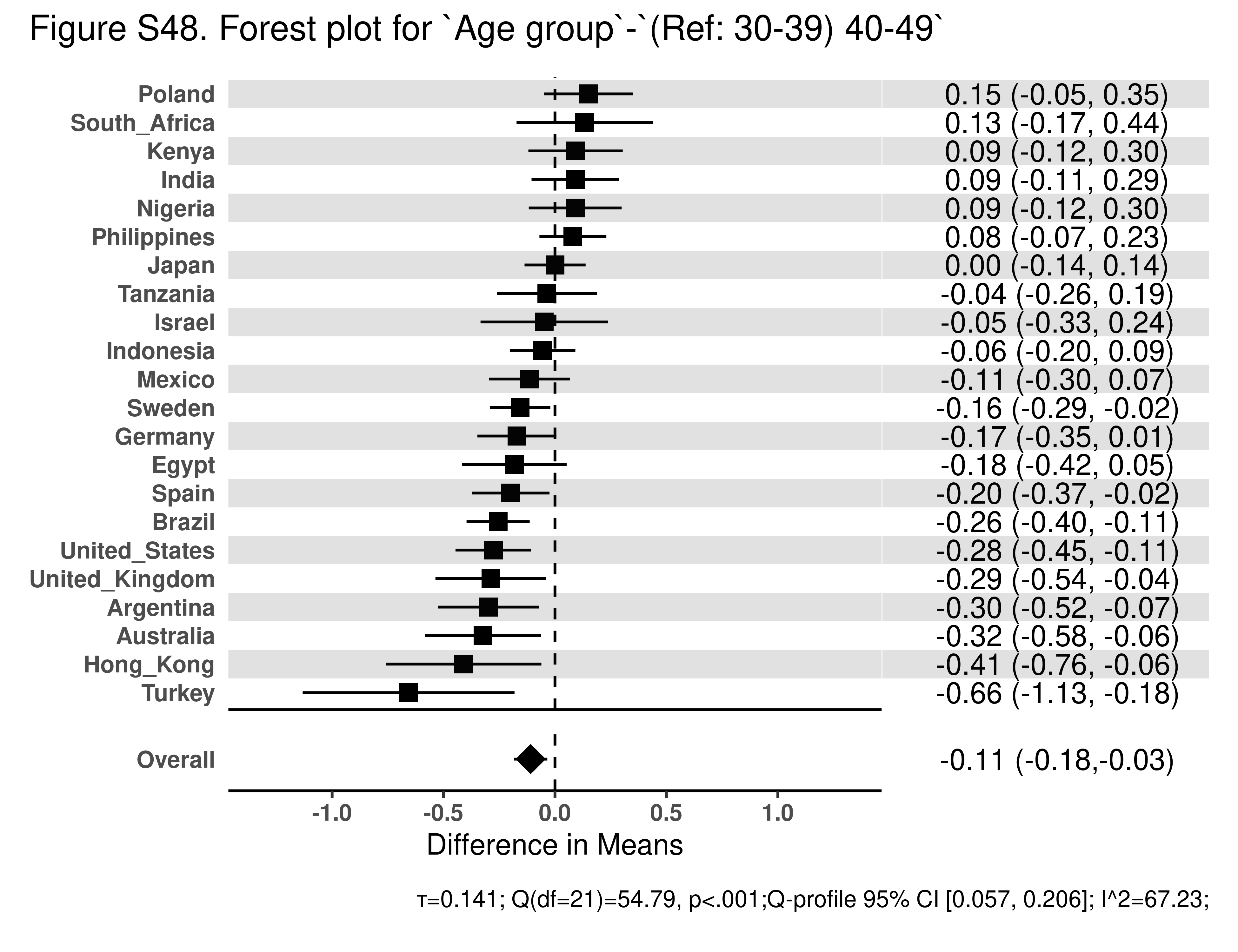


Figure S49. Forest plot for “Age group: (Ref: 30-39) 50-59”


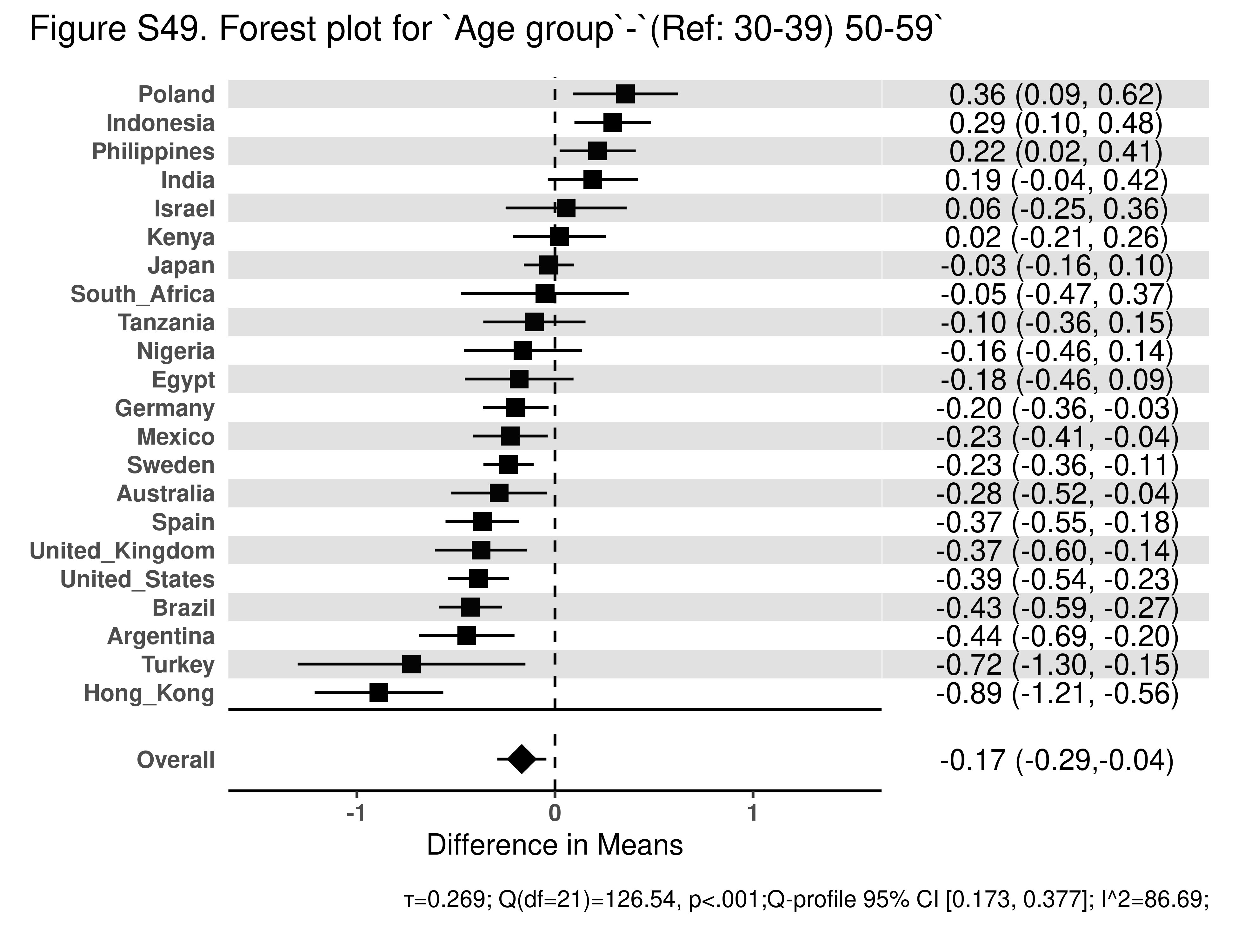


Figure S50. Forest plot for “Age group: (Ref: 30-39) 60-69”


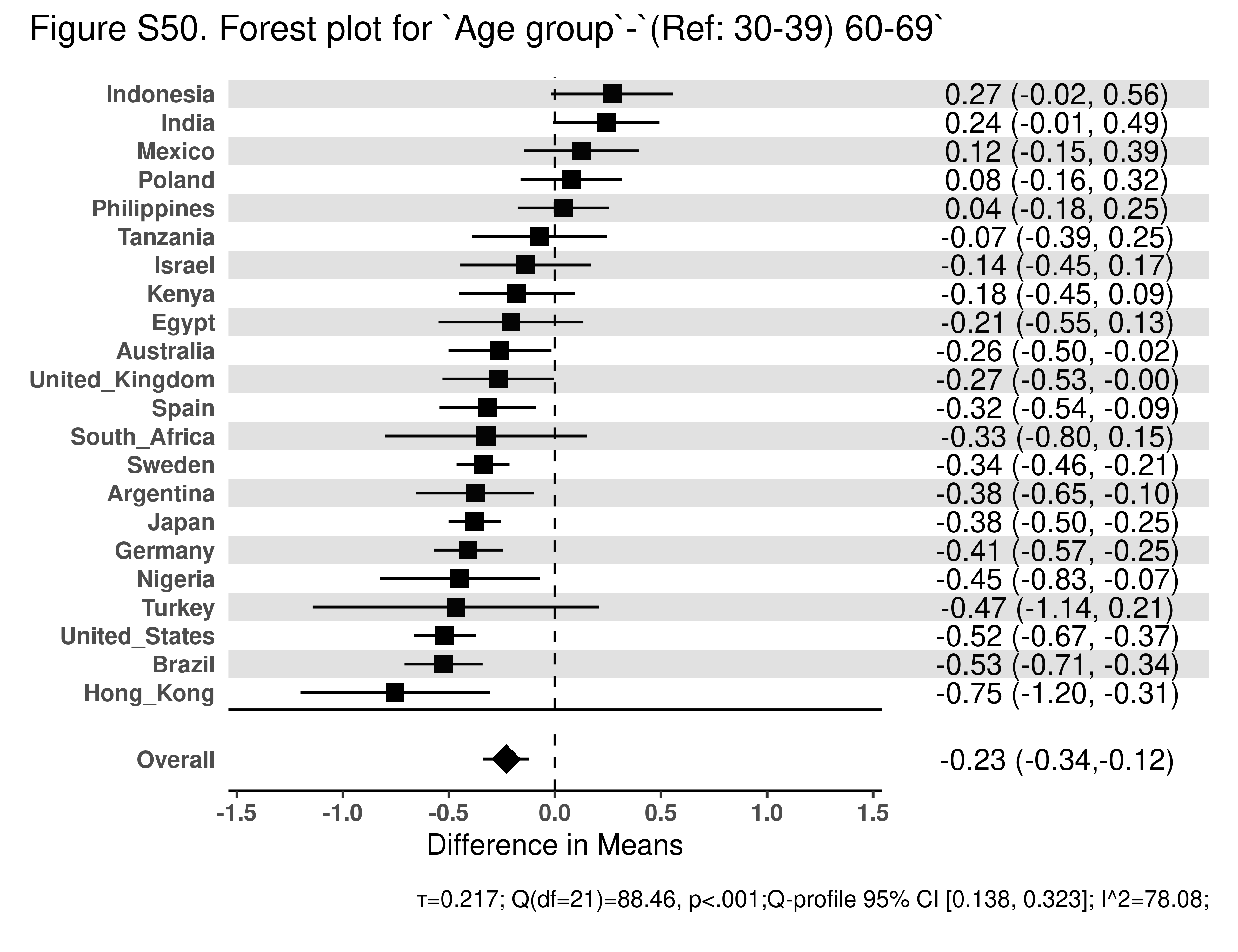


Figure S51. Forest plot for “Age group: (Ref: 30-39) 70-79”


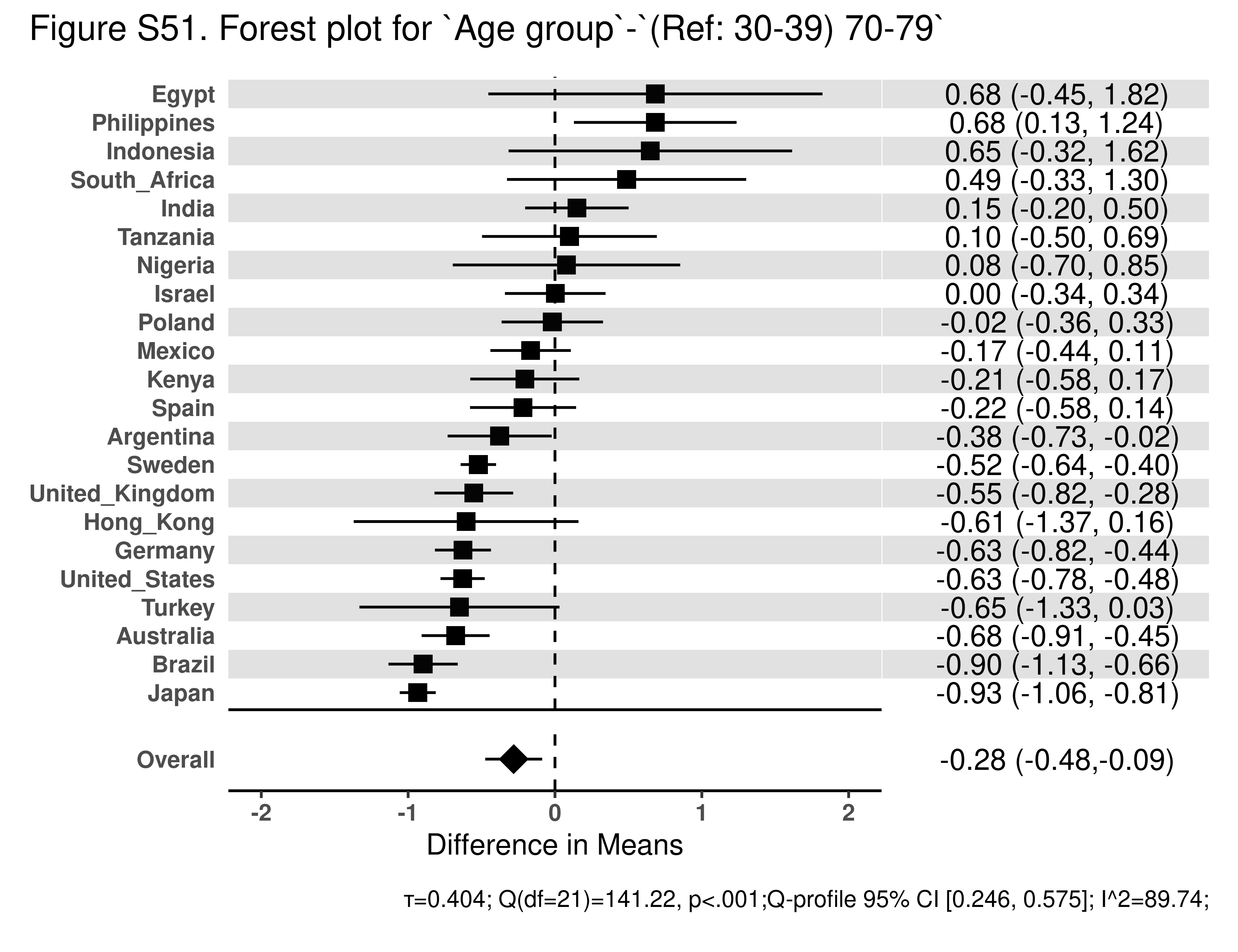


Figure S52. Forest plot for “Age group: (Ref: 30-39) 80 or older”


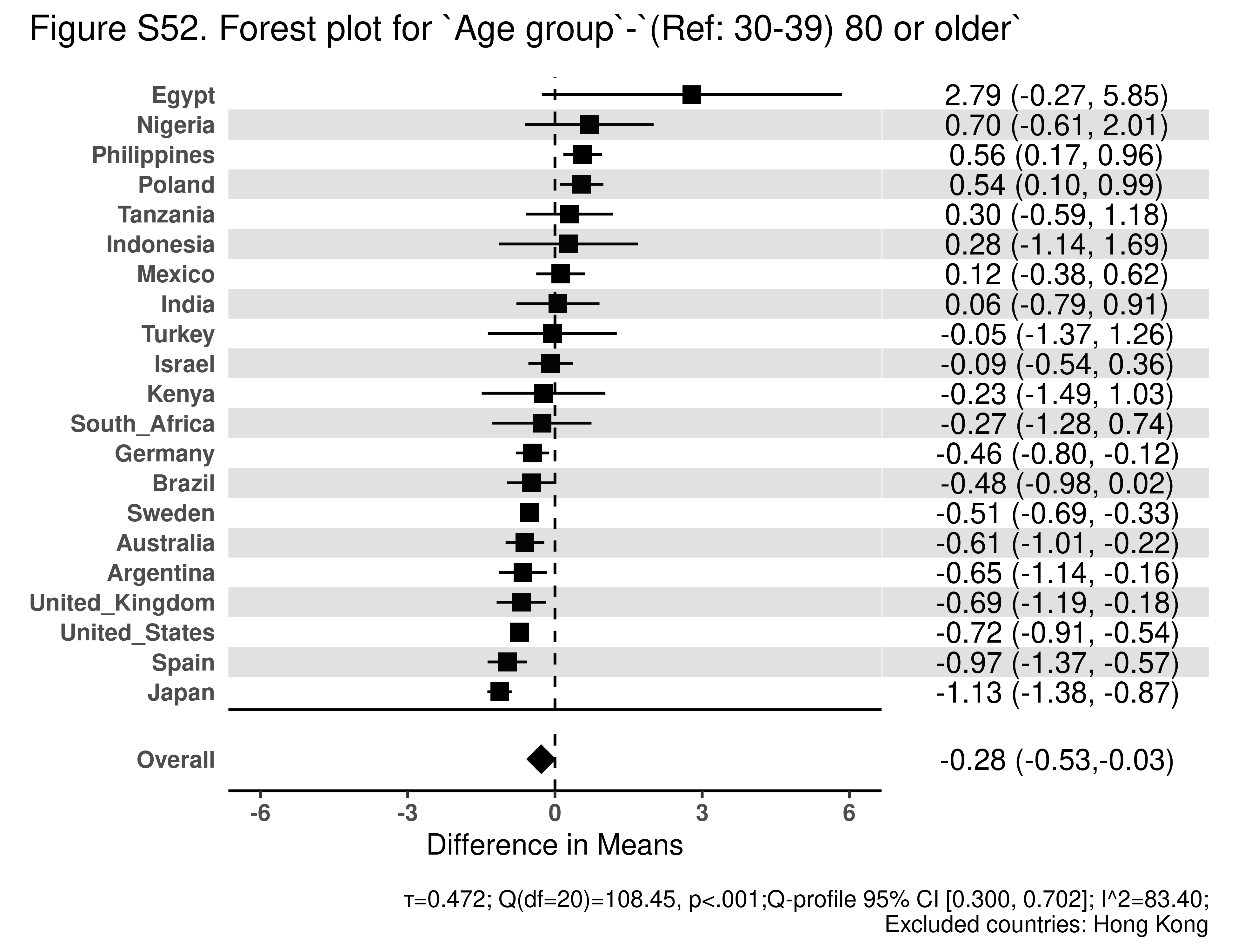


Figure S53. Forest plot for “Age group: (Ref: 40-49) 50-59”


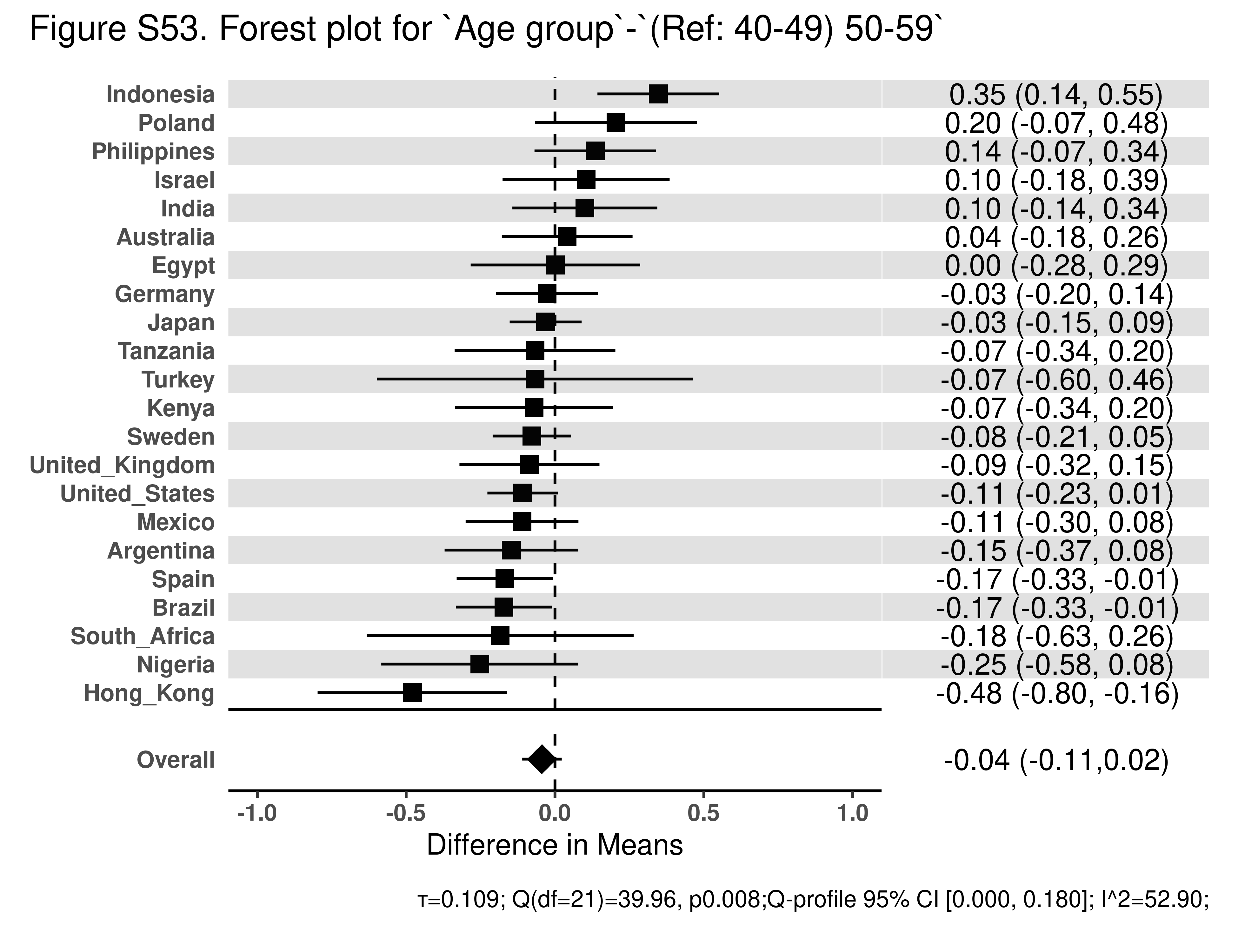


Figure S54. Forest plot for “Age group: (Ref: 40-49) 60-69”


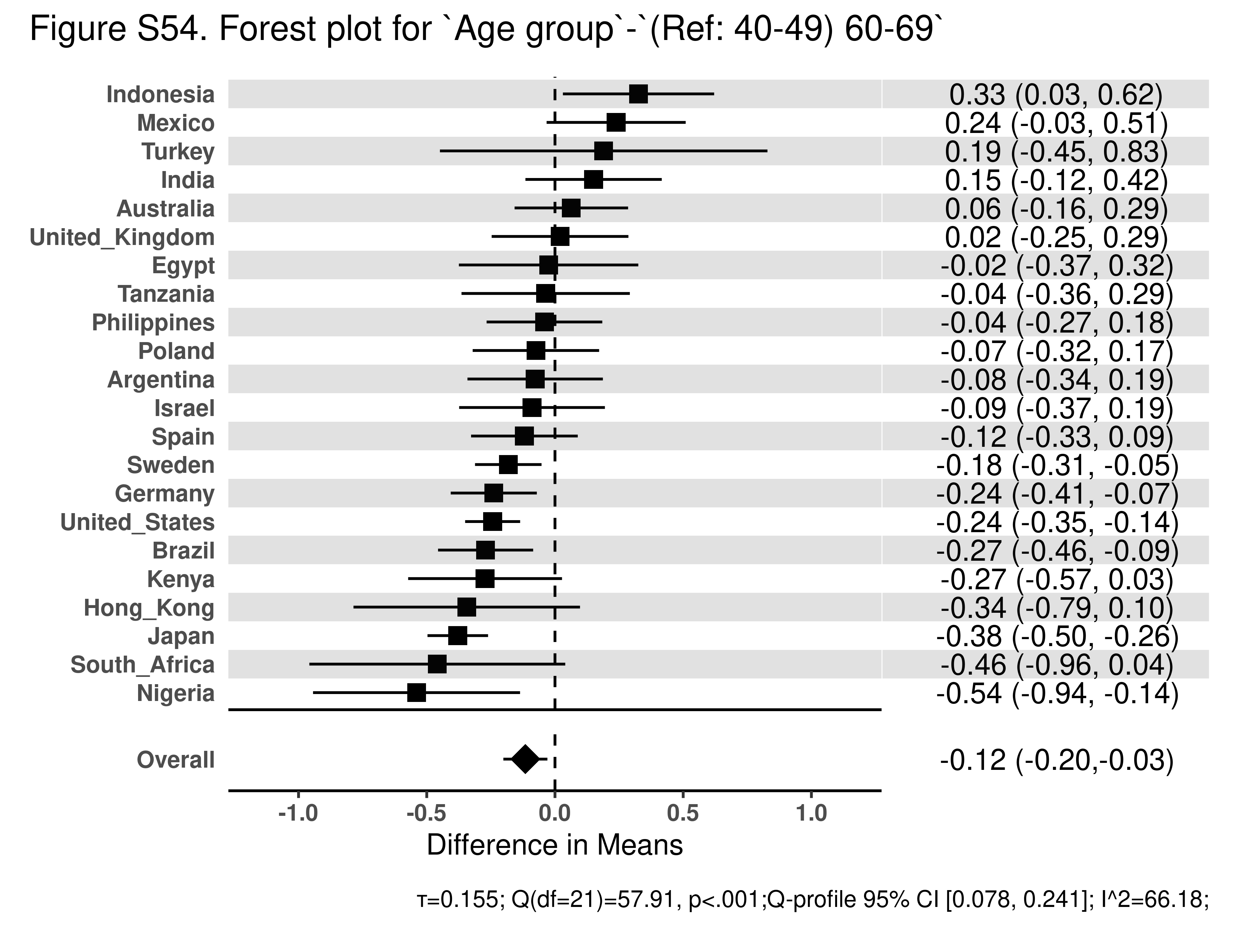


Figure S55. Forest plot for “Age group: (Ref: 40-49) 70-79”


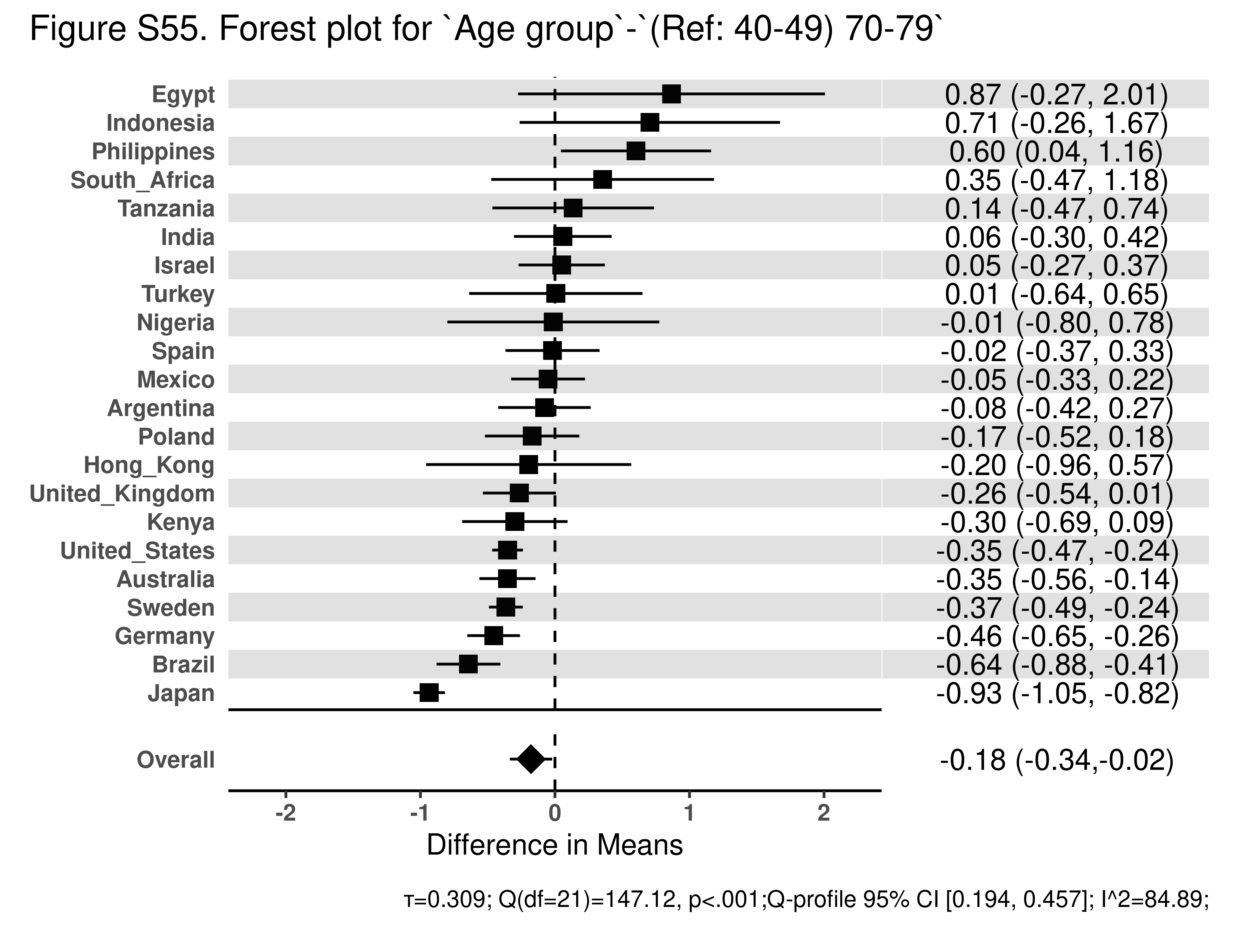


Figure S56. Forest plot for “Age group: (Ref: 40-49) 80 or older”


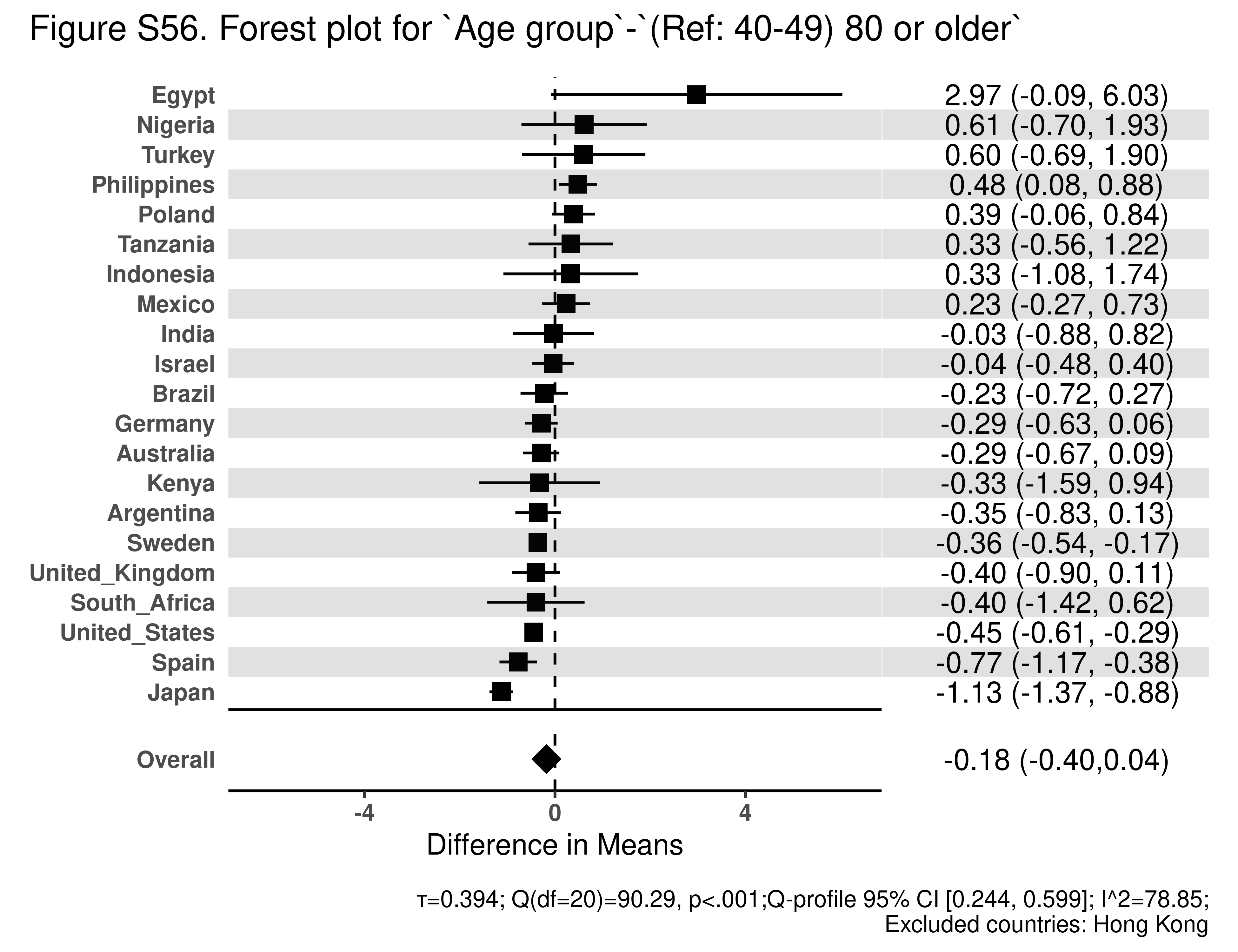


Figure S57. Forest plot for “Age group: (Ref: 50-59) 60-69”


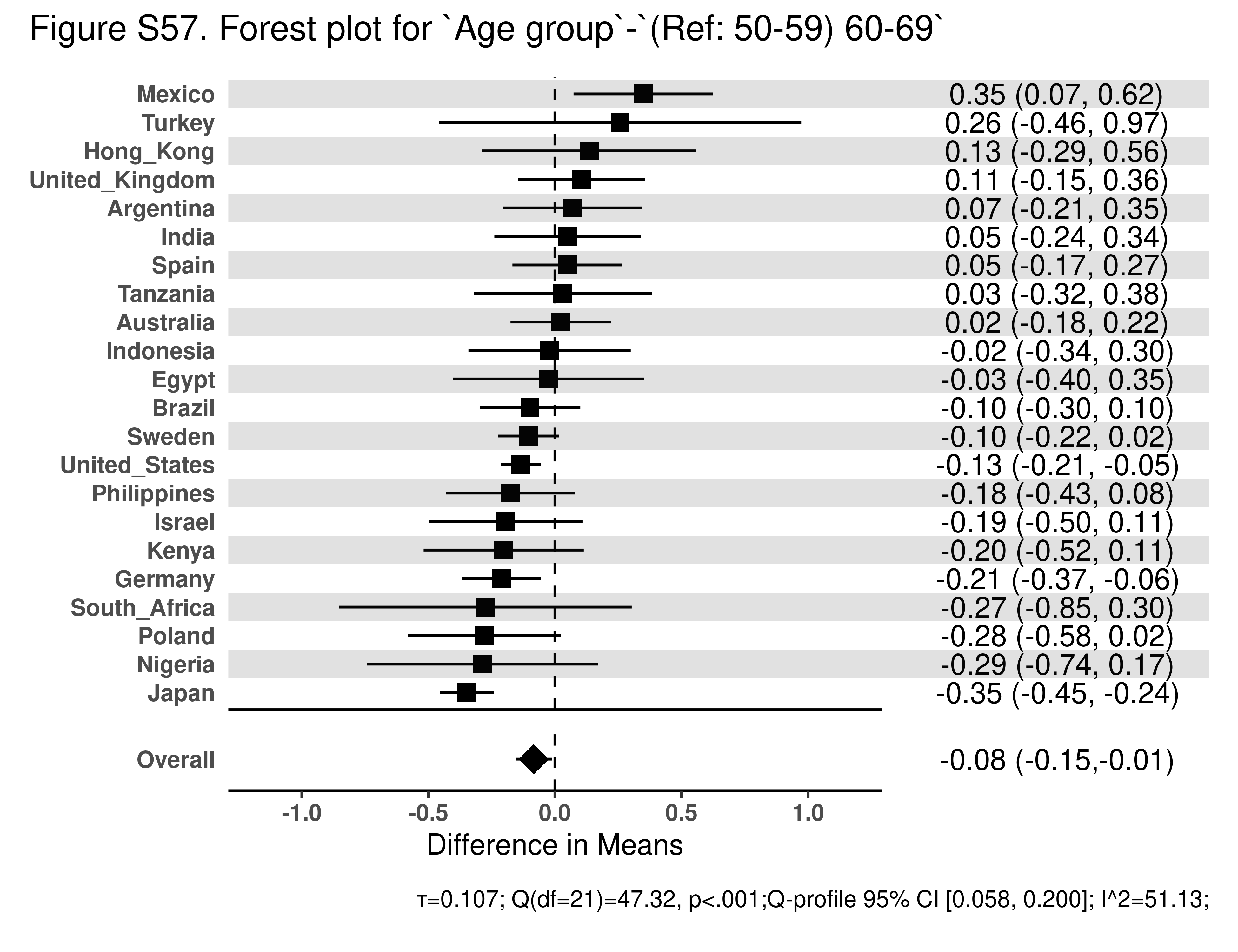


Figure S58. Forest plot for “Age group: (Ref: 50-59) 70-79”


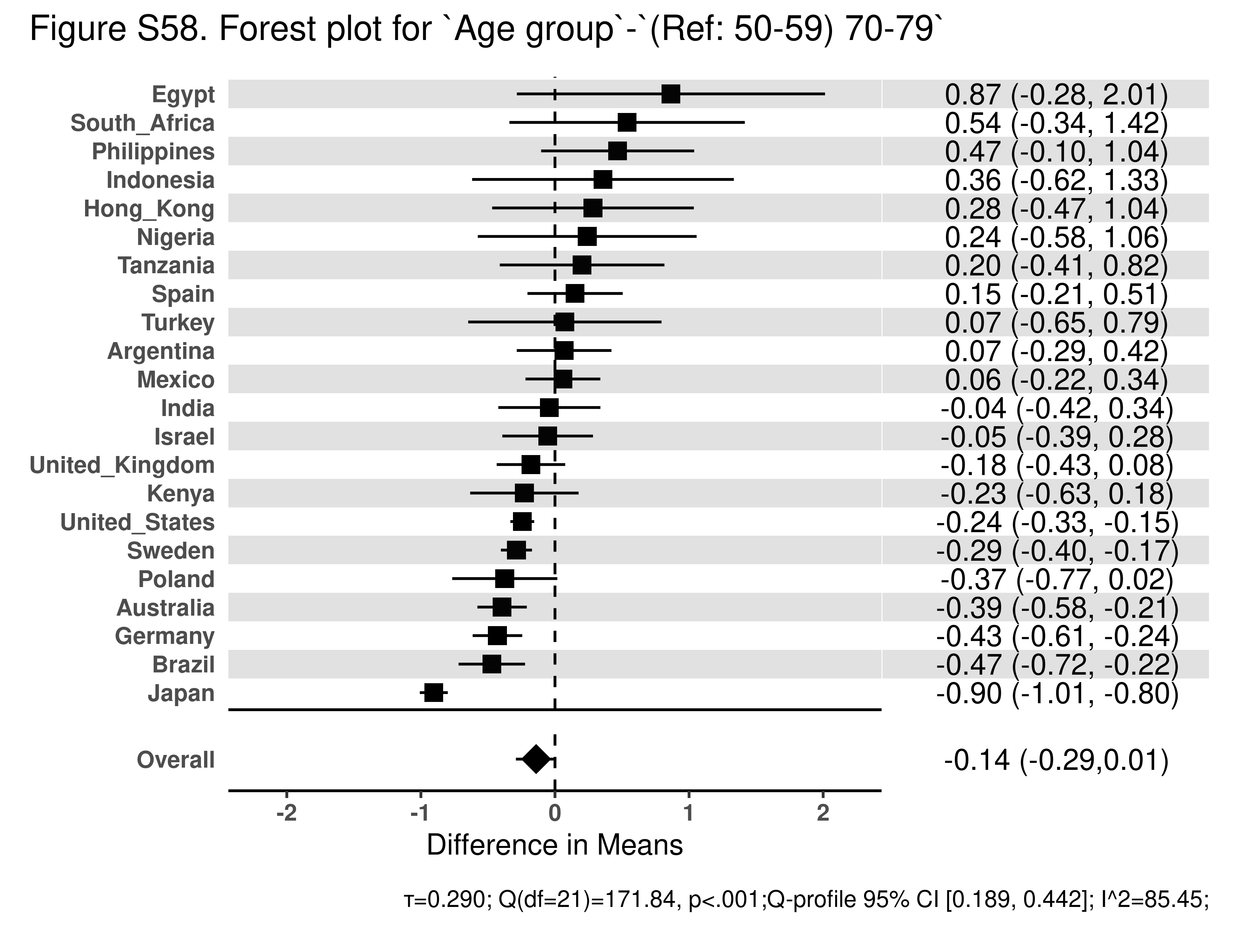


Figure S59. Forest plot for “Age group: (Ref: 50-59) 80 or older”


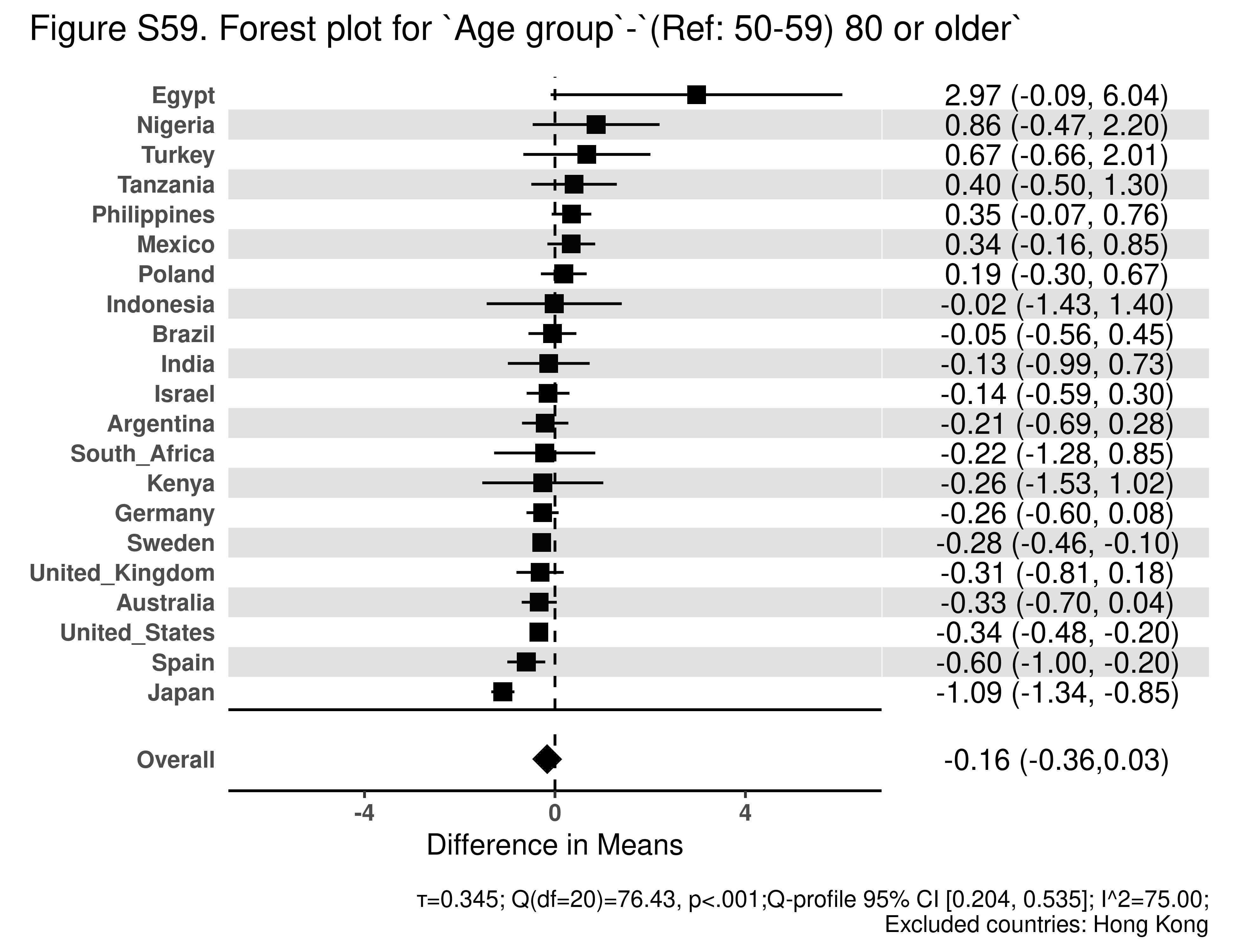


Figure S60. Forest plot for “Age group: (Ref: 60-69) 70-79”


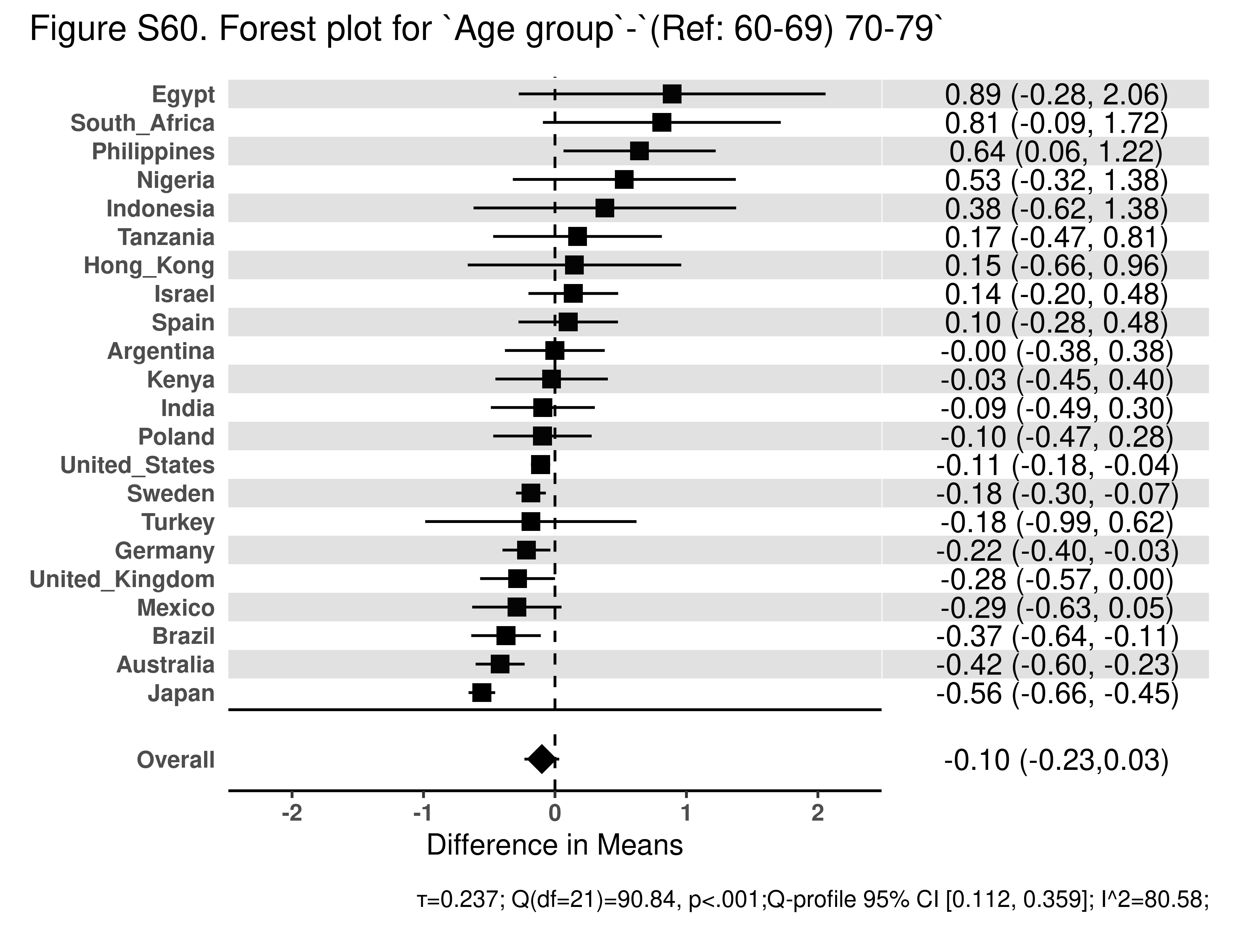


Figure S61. Forest plot for “Age group: (Ref: 60-69) 80 or older”


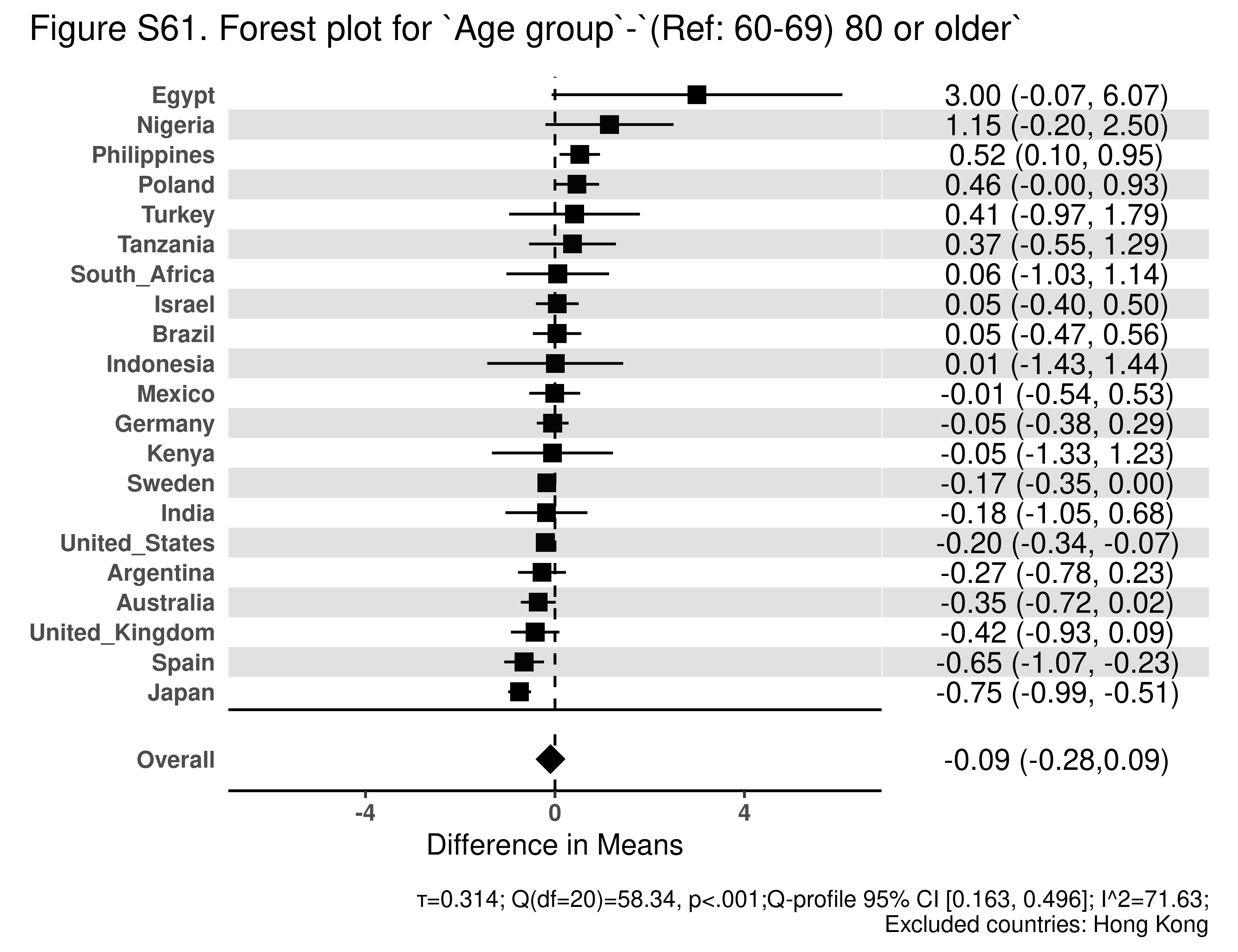


Figure S62. Forest plot for “Age group: (Ref: 70-79) 80 or older”


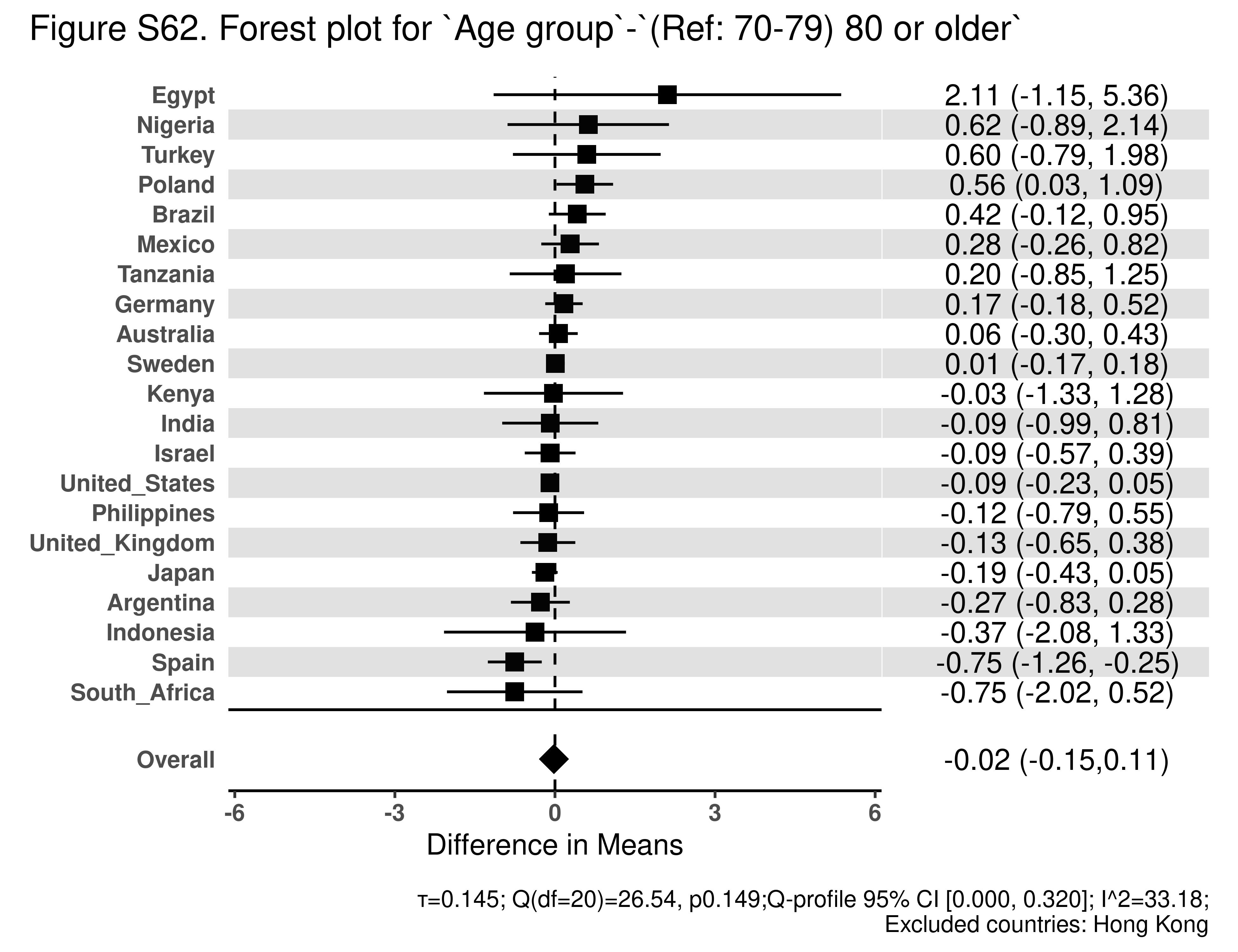


Figure S63. Forest plot for “Gender: (Ref: Male) Female”


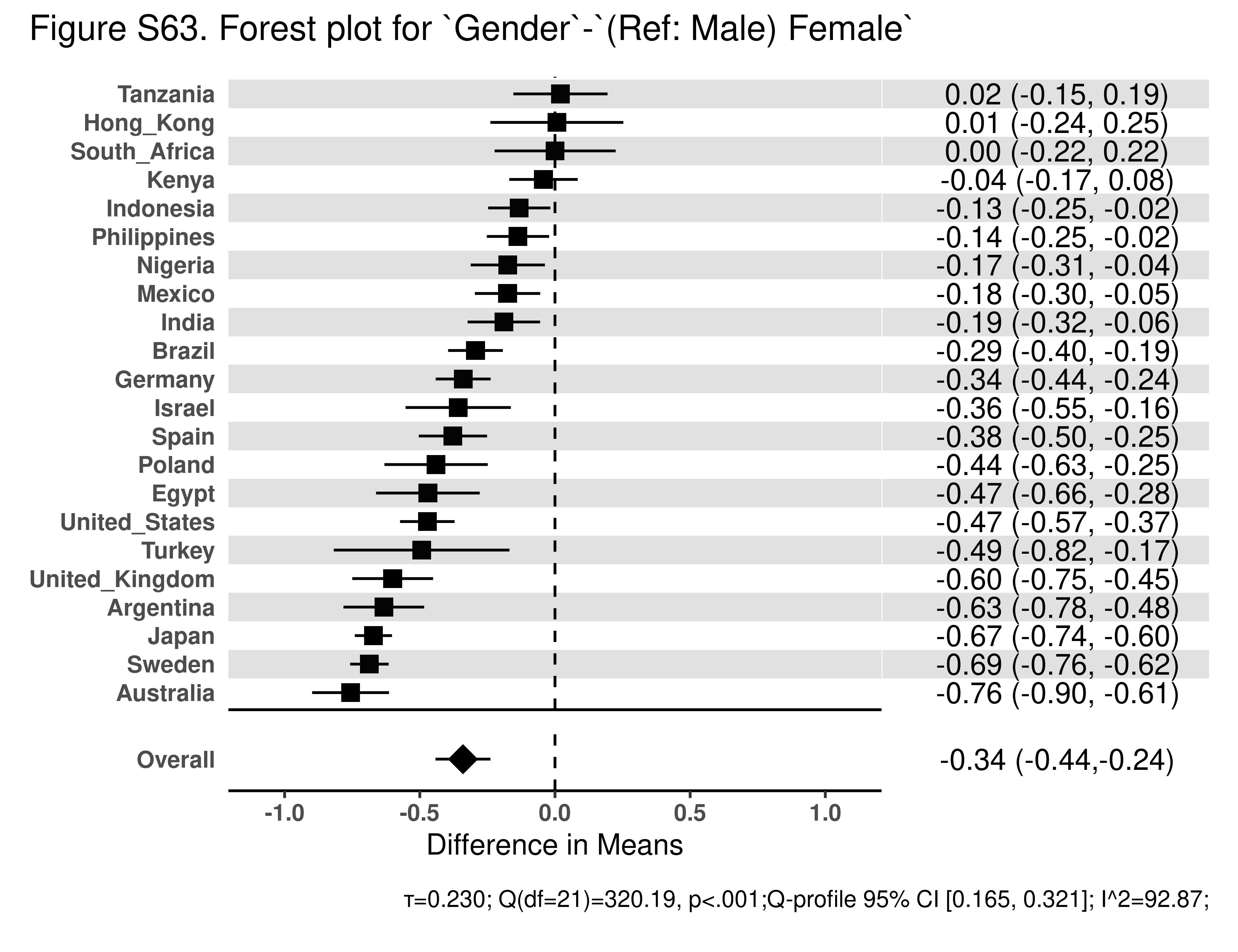


Figure S64. Forest plot for “Gender: (Ref: Male) Other”


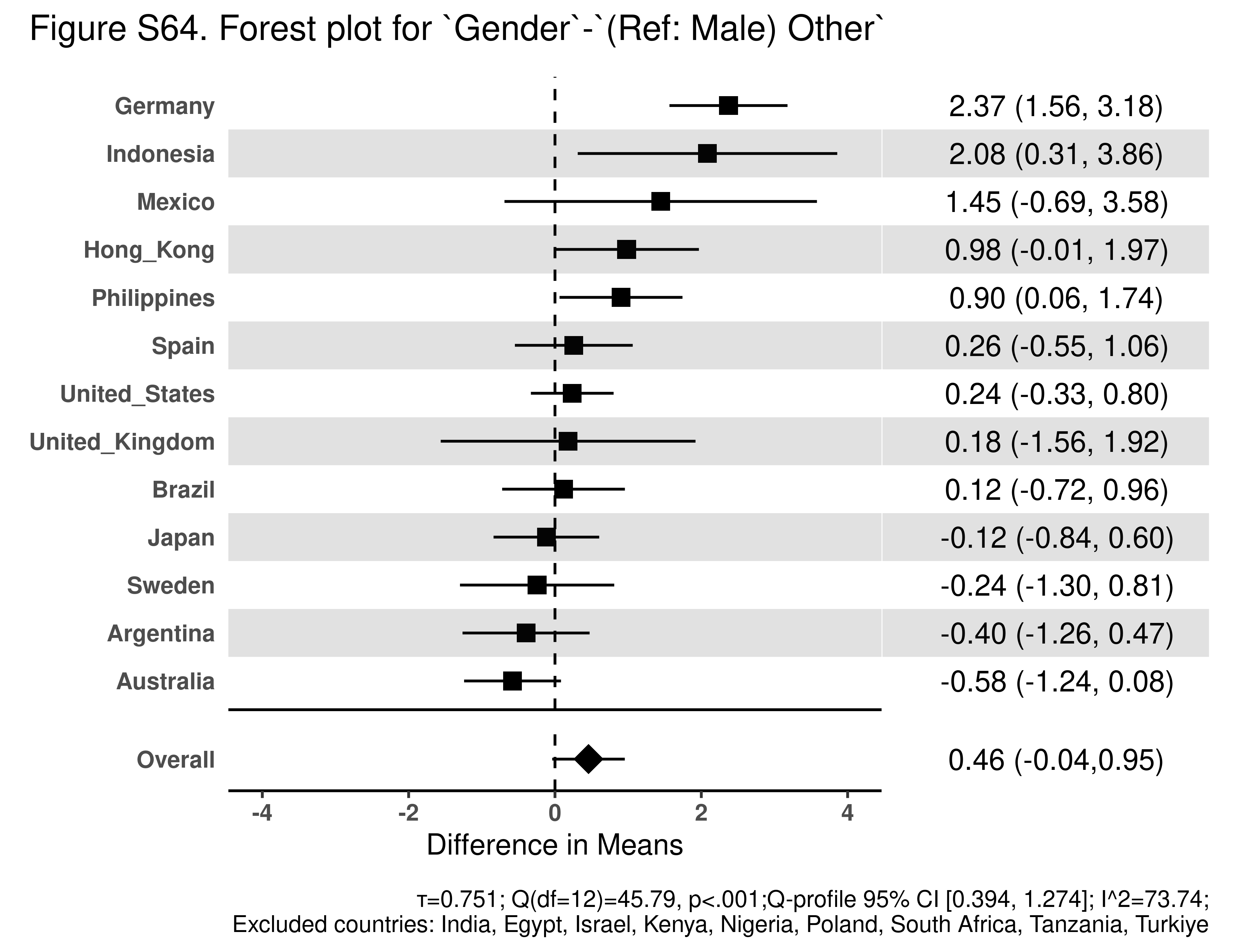


Figure S65. Forest plot for “Gender: (Ref: Female) Other”


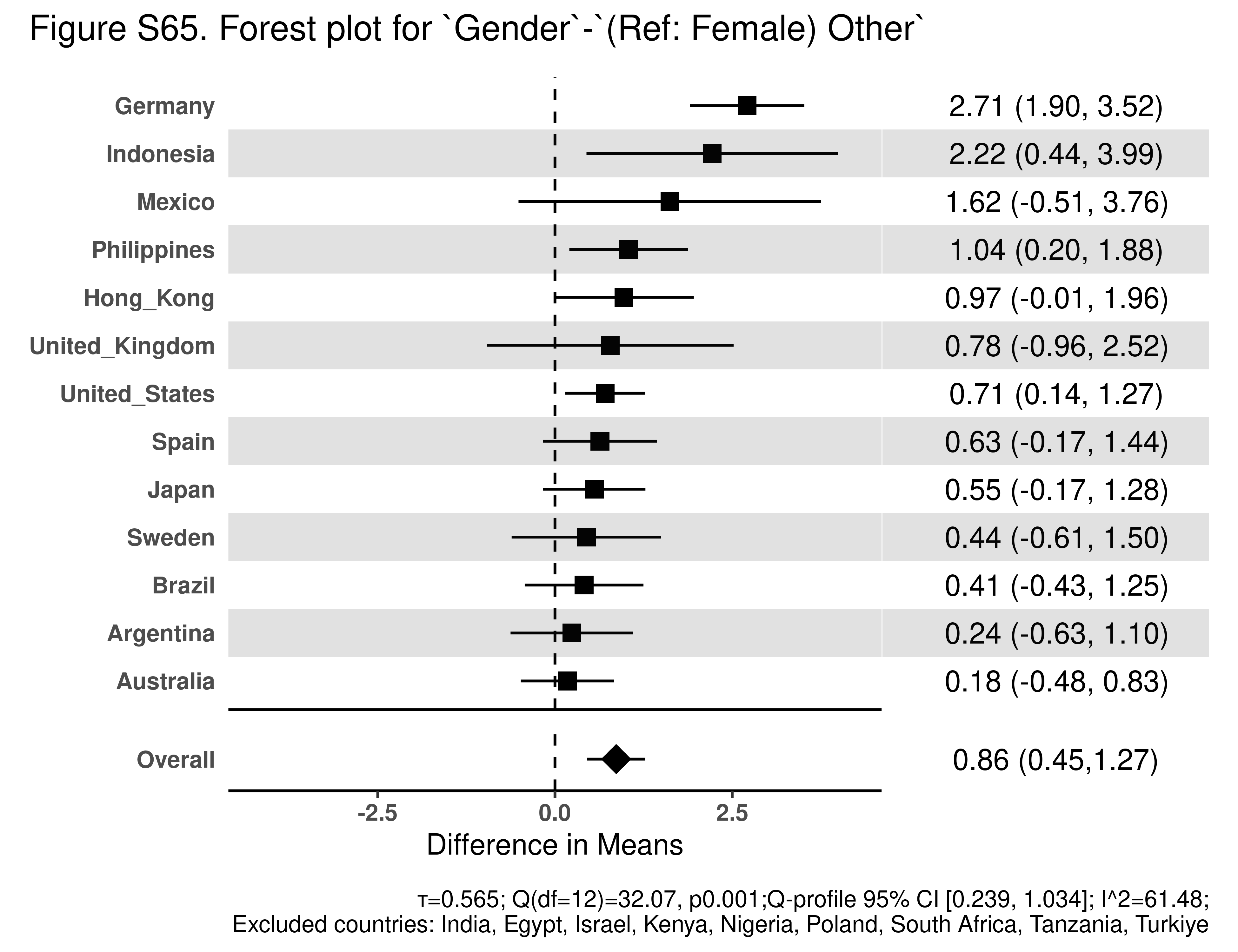


Figure S66. Forest plot for “Marital status: (Ref: Single, never married) Married”


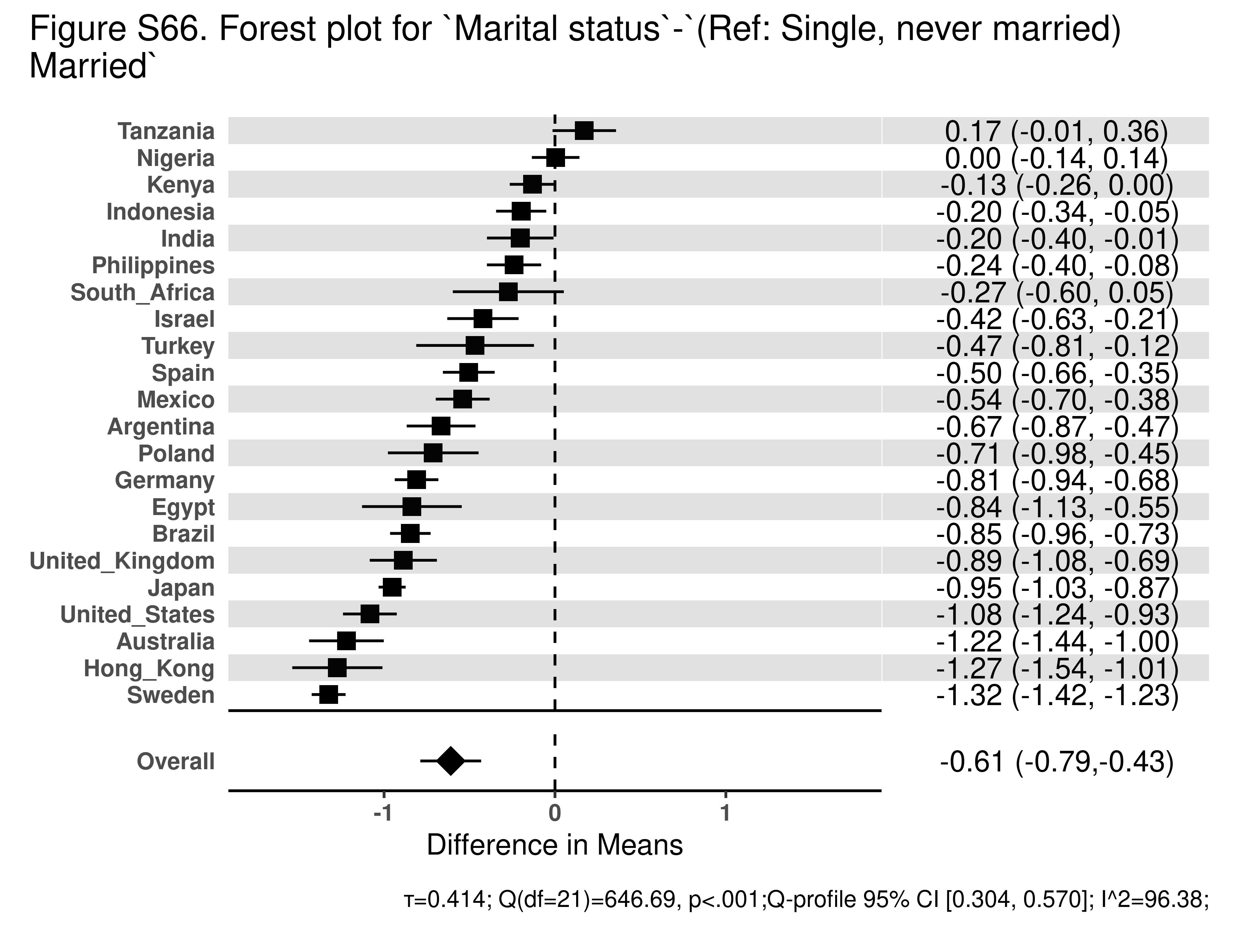


Figure S67. Forest plot for “Marital status: (Ref: Single, never married) Separated”


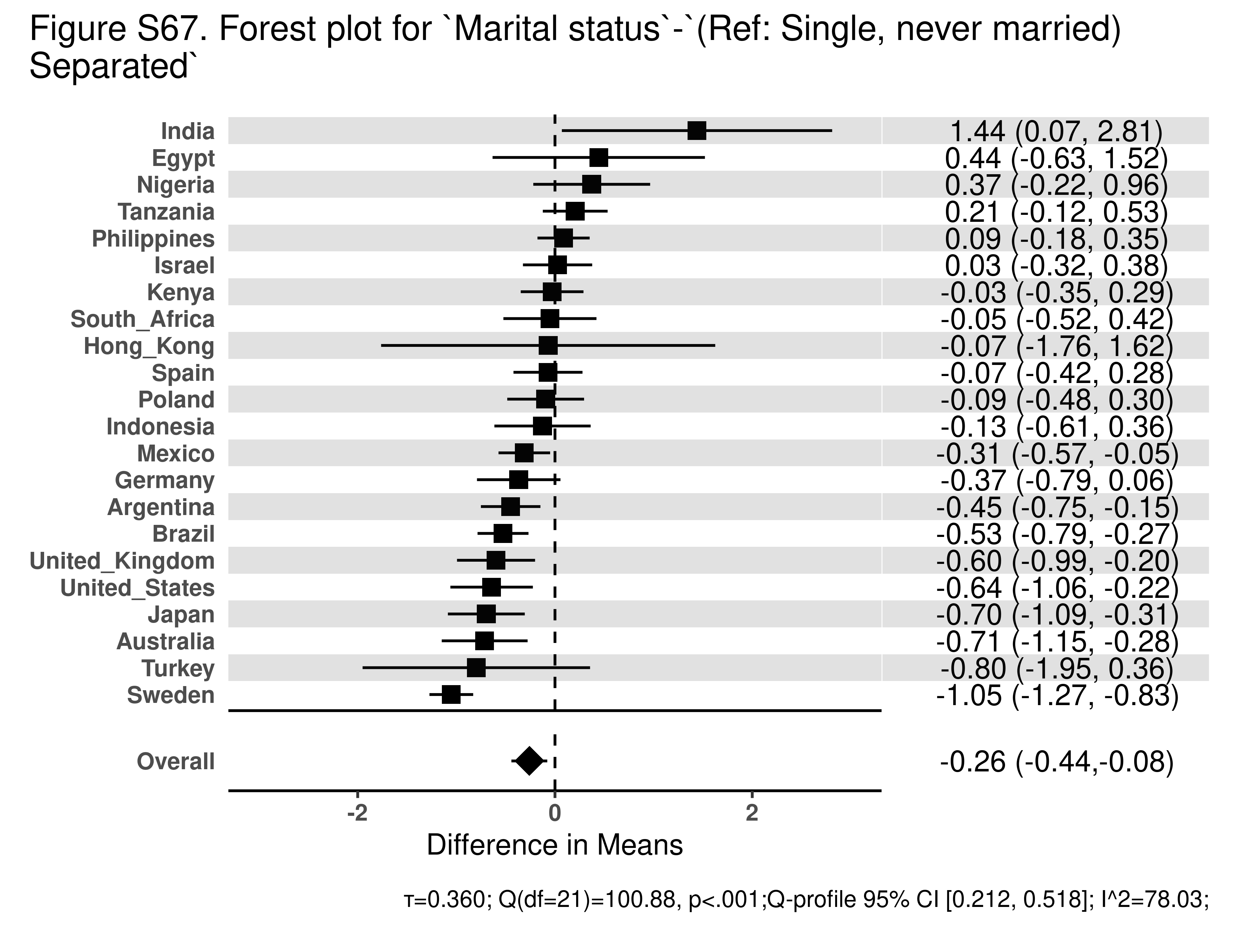


Figure S68. Forest plot for “Marital status: (Ref: Single, never married) Divorced”


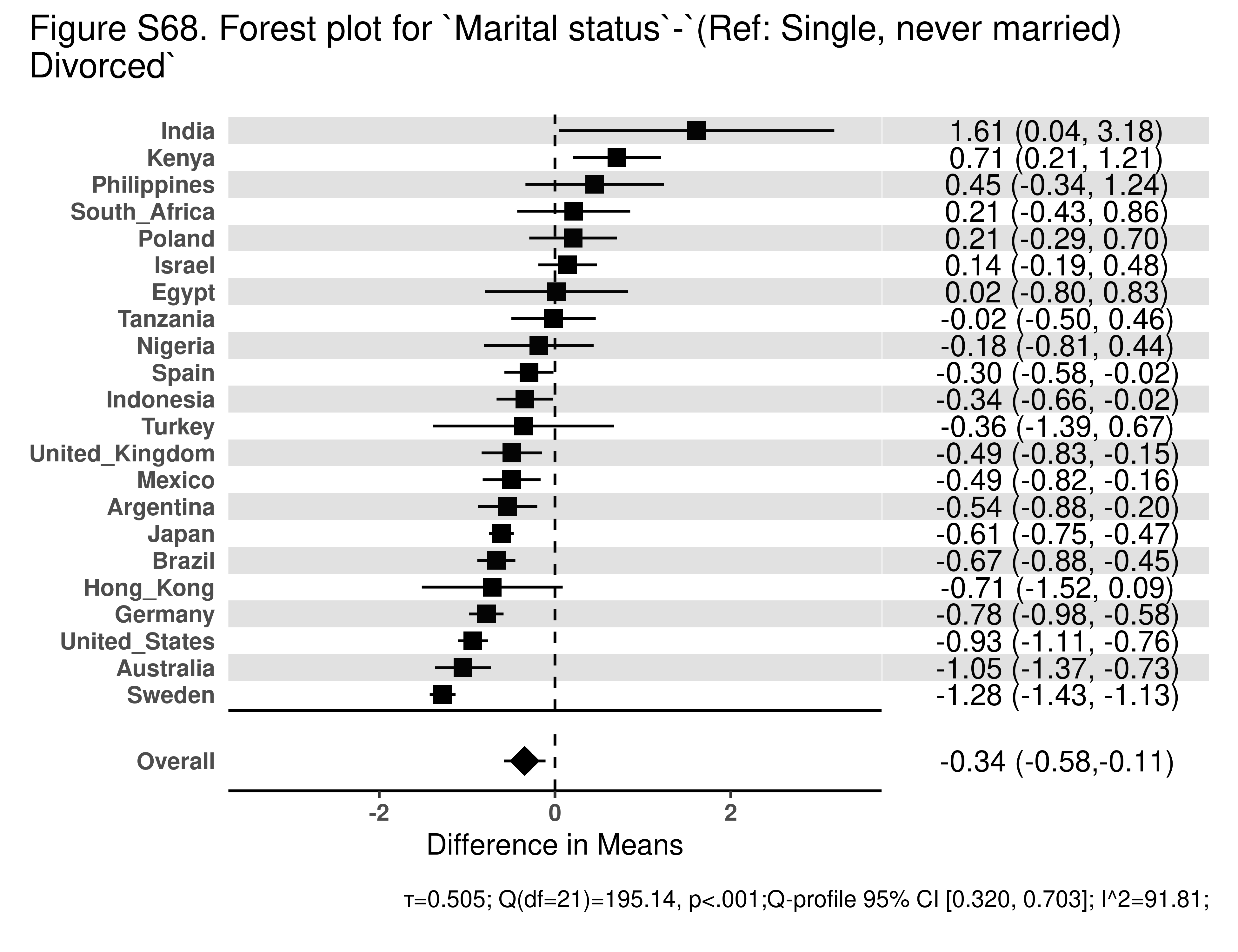


Figure S69. Forest plot for “Marital status: (Ref: Single, never married) Widowed”


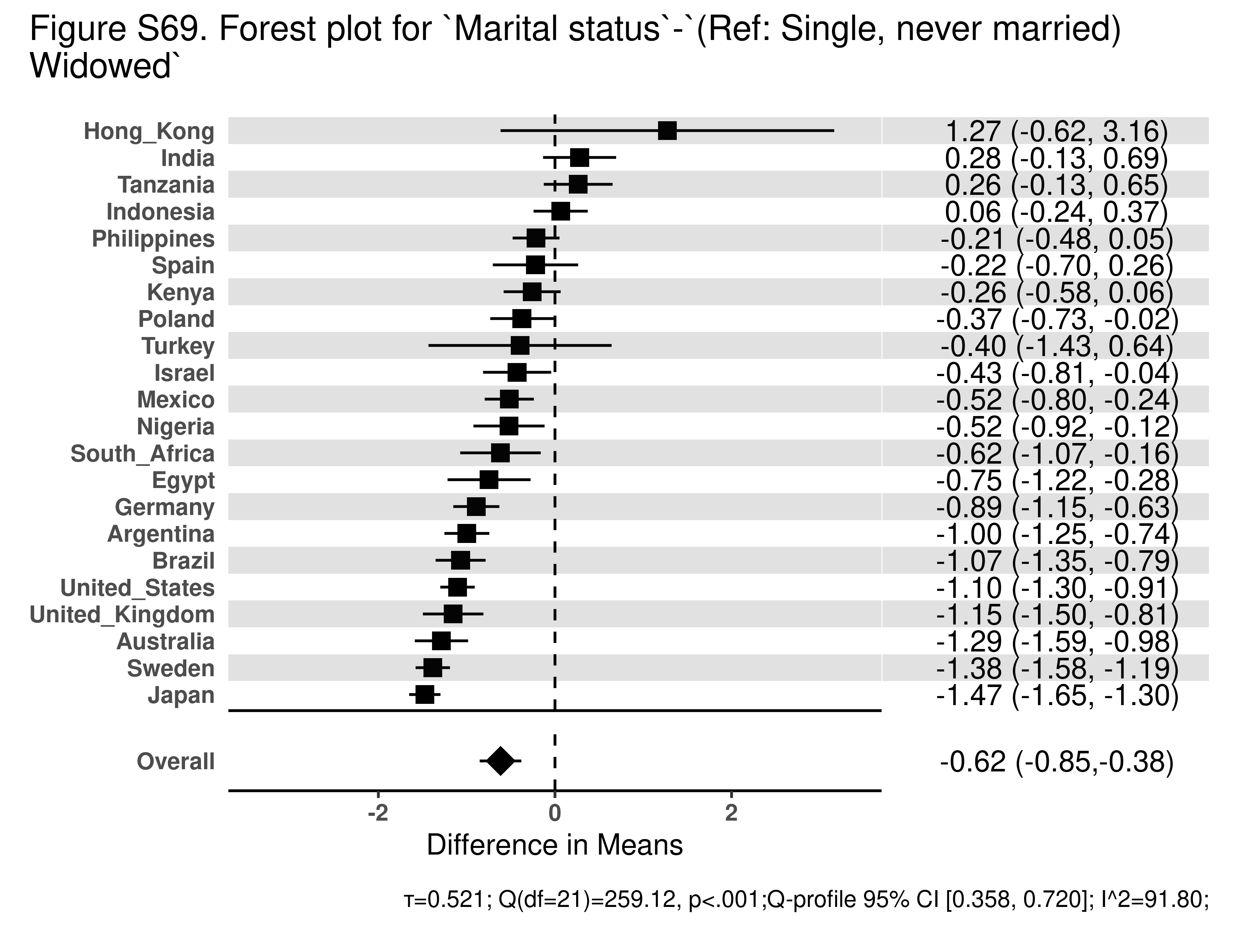


Figure S70. Forest plot for “Marital status: (Ref: Single, never married) Domestic partner”


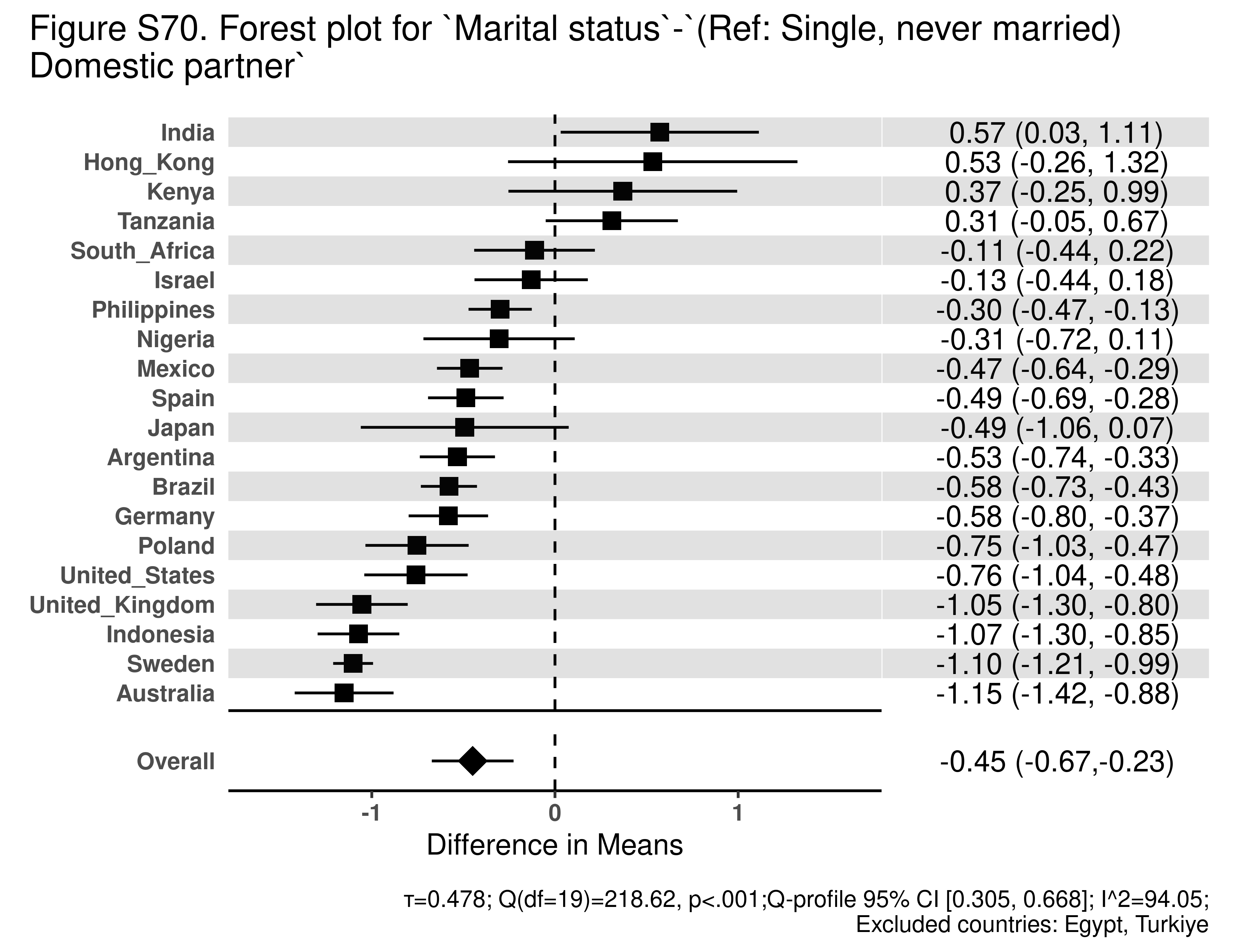


Figure S71. Forest plot for “Marital status: (Ref: Married) Separated”


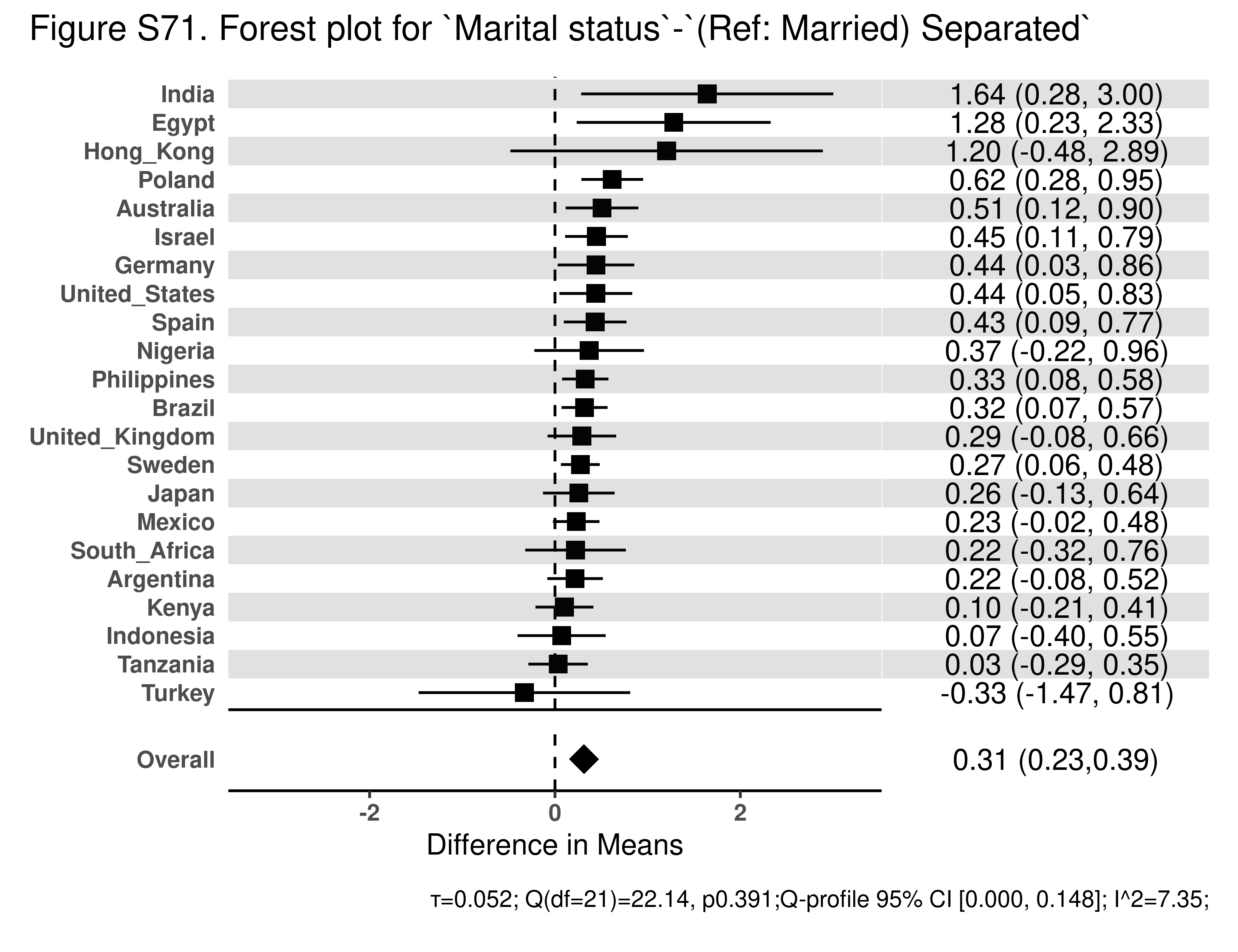


Figure S72. Forest plot for “Marital status: (Ref: Married) Divorced”


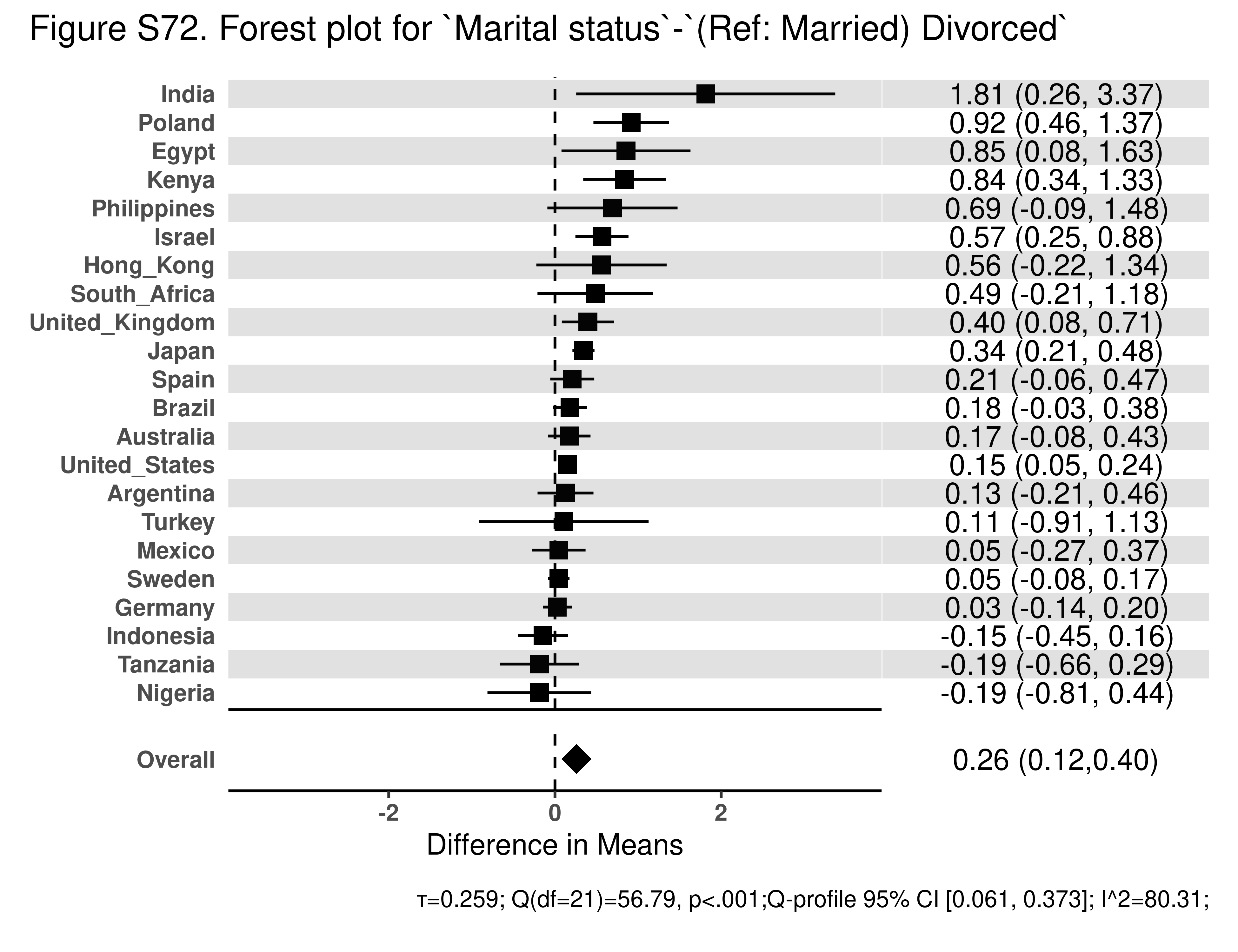


Figure S73. Forest plot for “Marital status: (Ref: Married) Widowed”


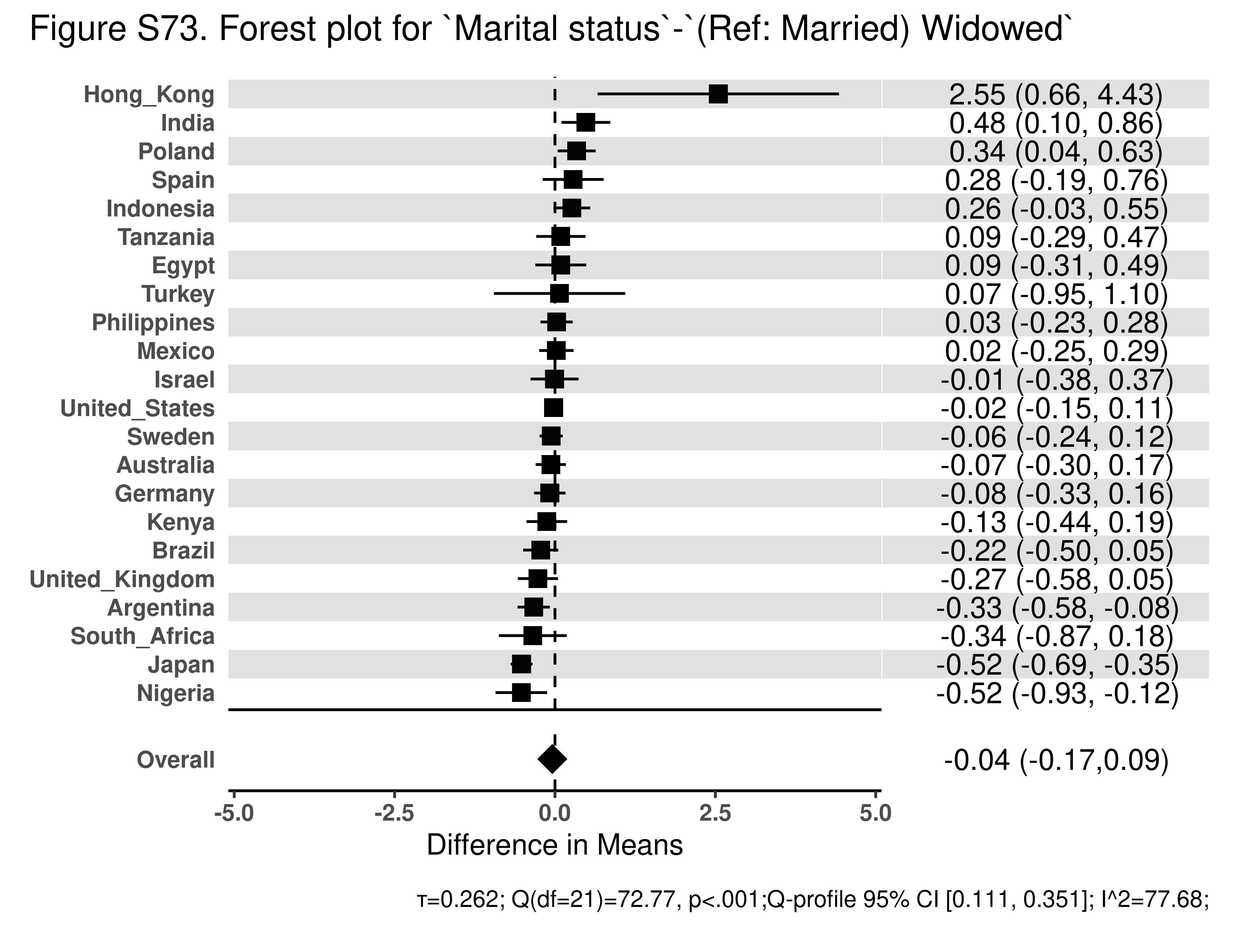


Figure S74. Forest plot for “Marital status: (Ref: Married) Domestic partner”


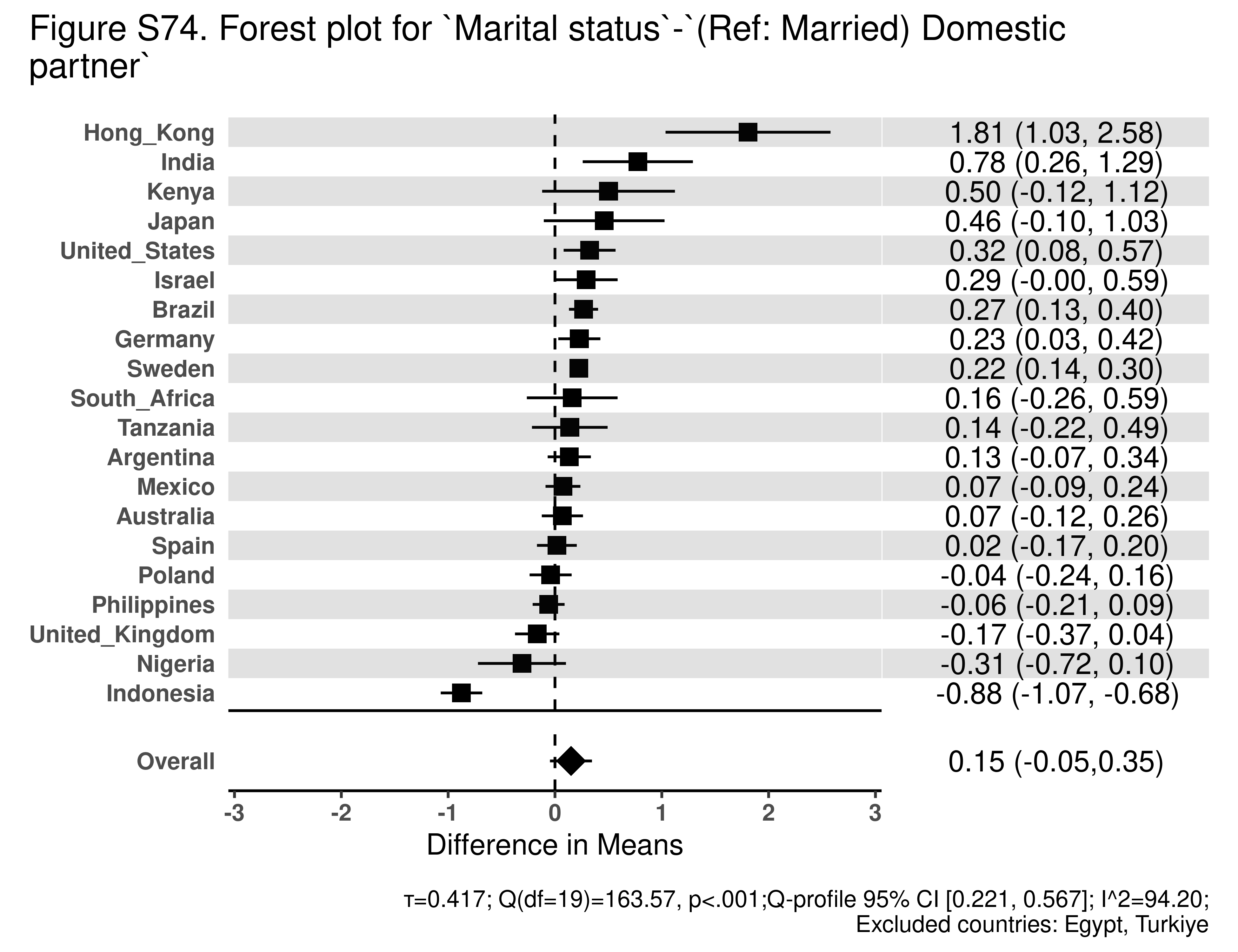


Figure S75. Forest plot for “Marital status: (Ref: Separated) Divorced”


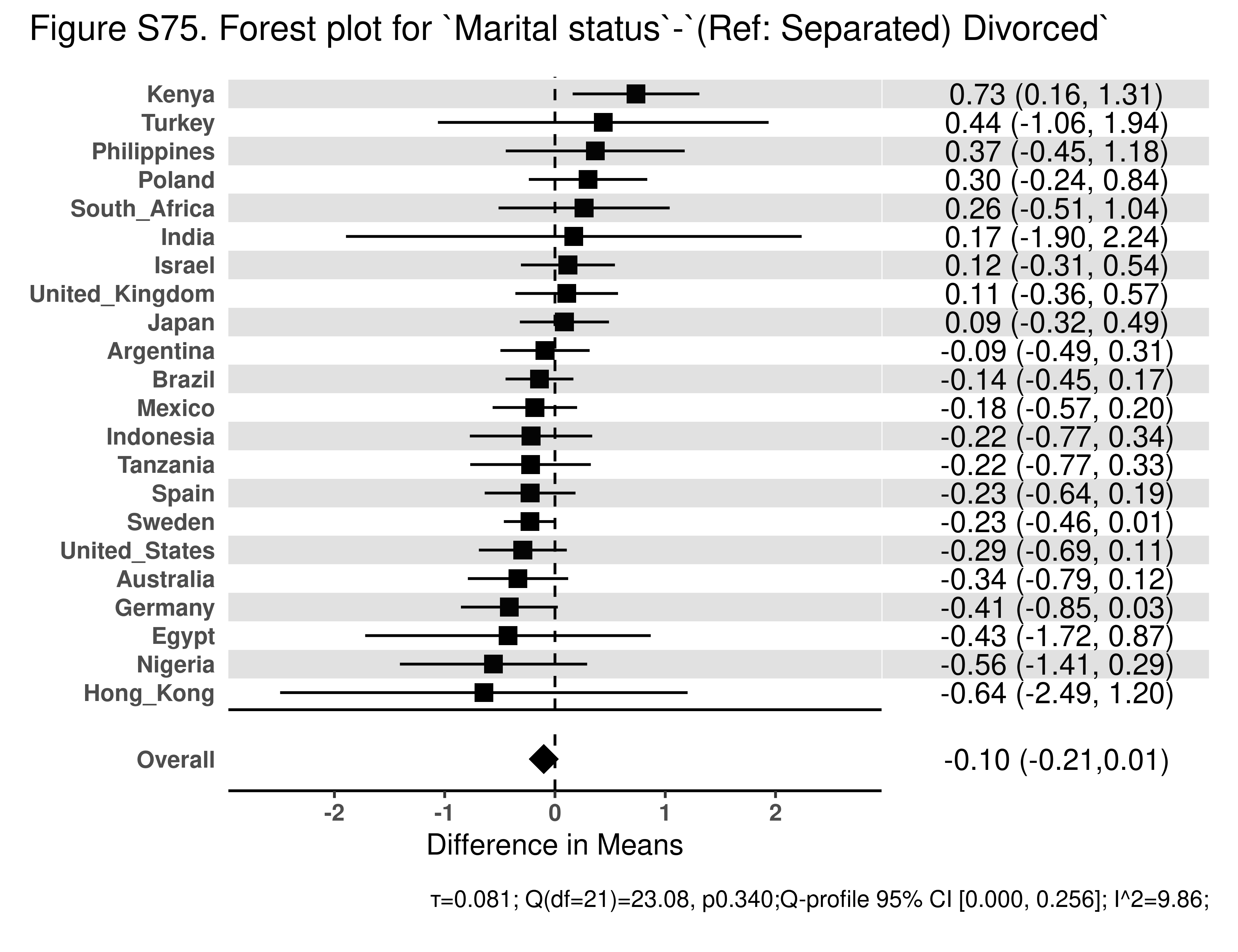


Figure S76. Forest plot for “Marital status: (Ref: Separated) Widowed”


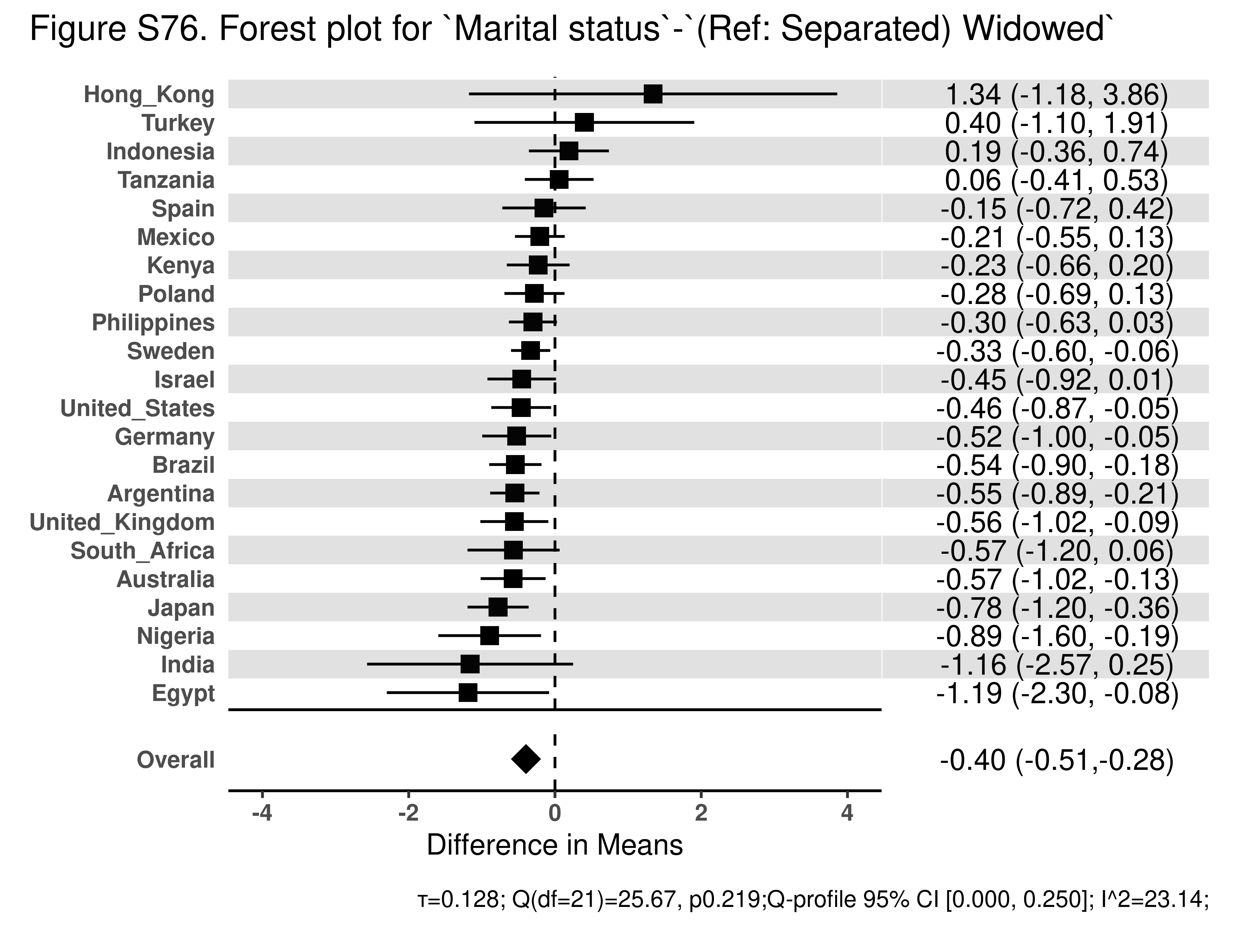


Figure S77. Forest plot for “Marital status: (Ref: Separated) Domestic partner”


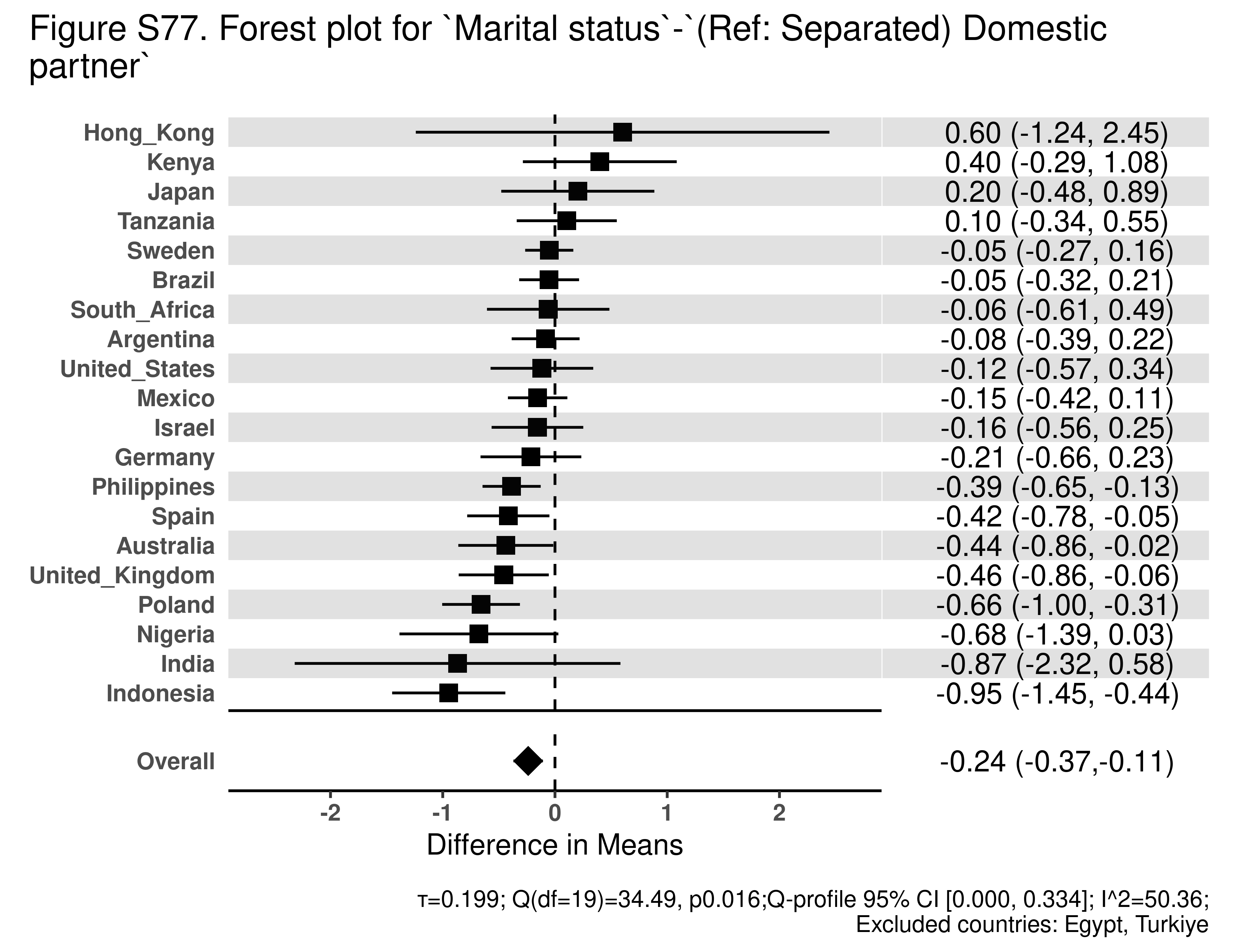


Figure S78. Forest plot for “Marital status: (Ref: Divorced) Widowed”


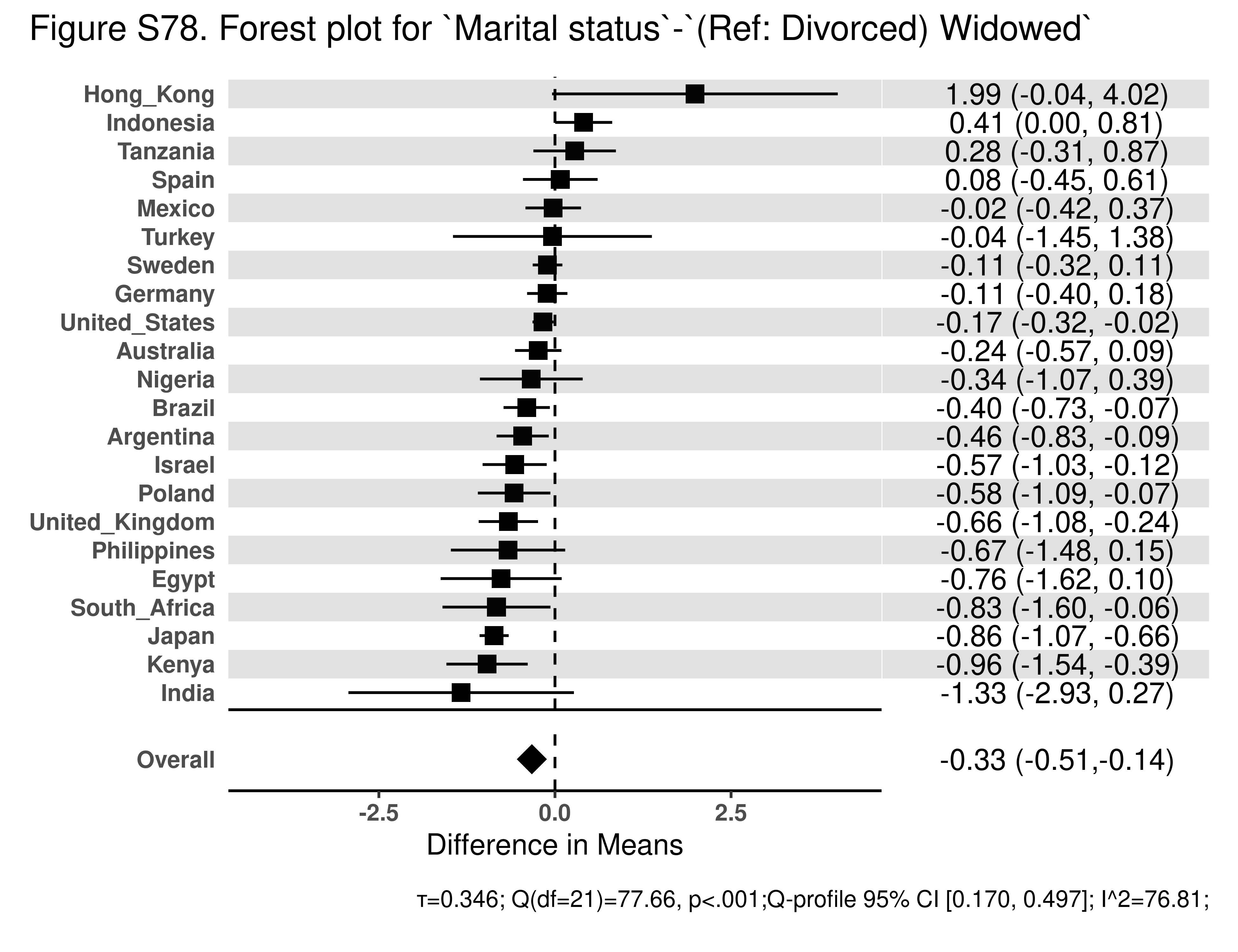


Figure S79. Forest plot for “Marital status: (Ref: Divorced) Domestic partner”


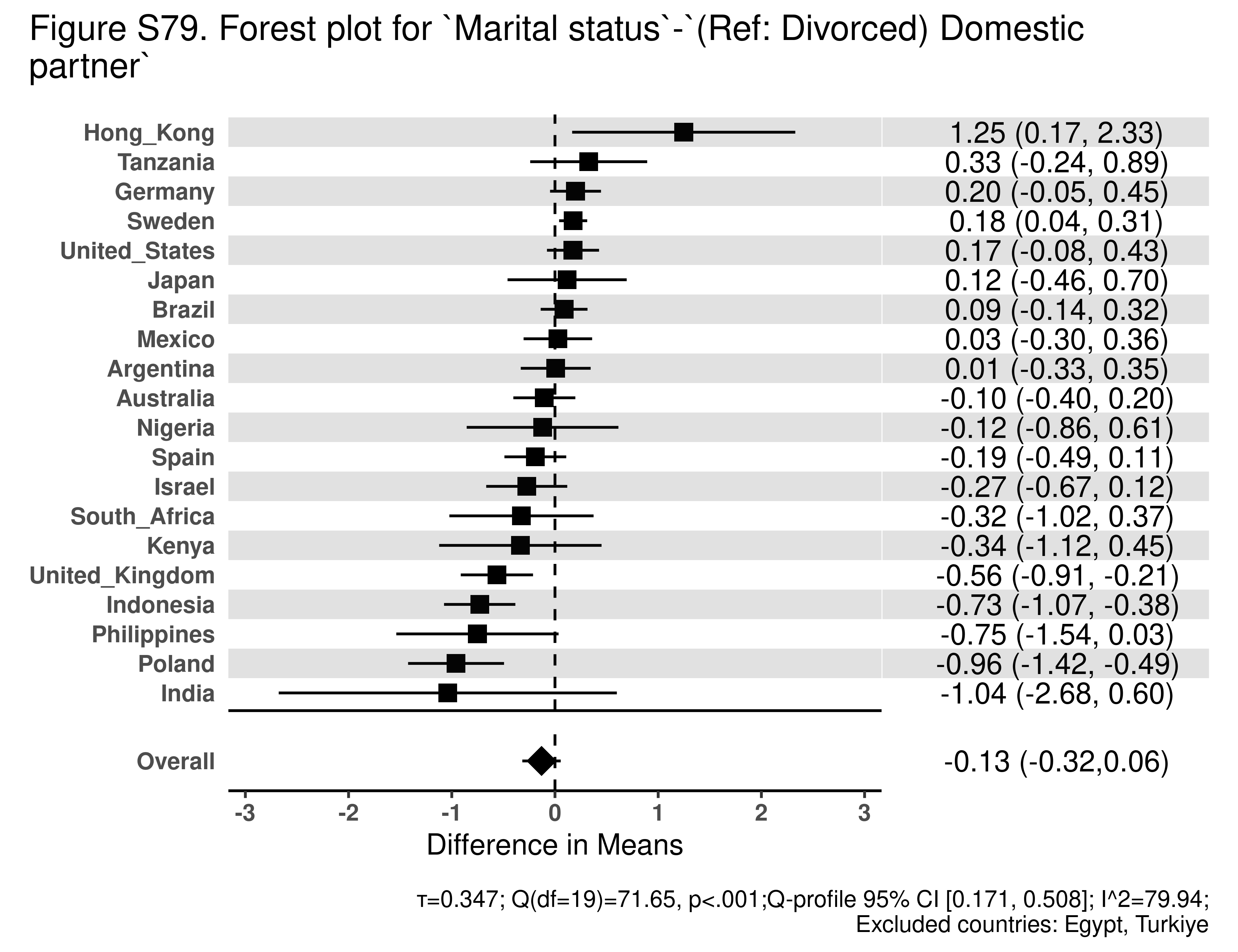


Figure S80. Forest plot for “Marital status: (Ref: Widowed) Domestic partner”


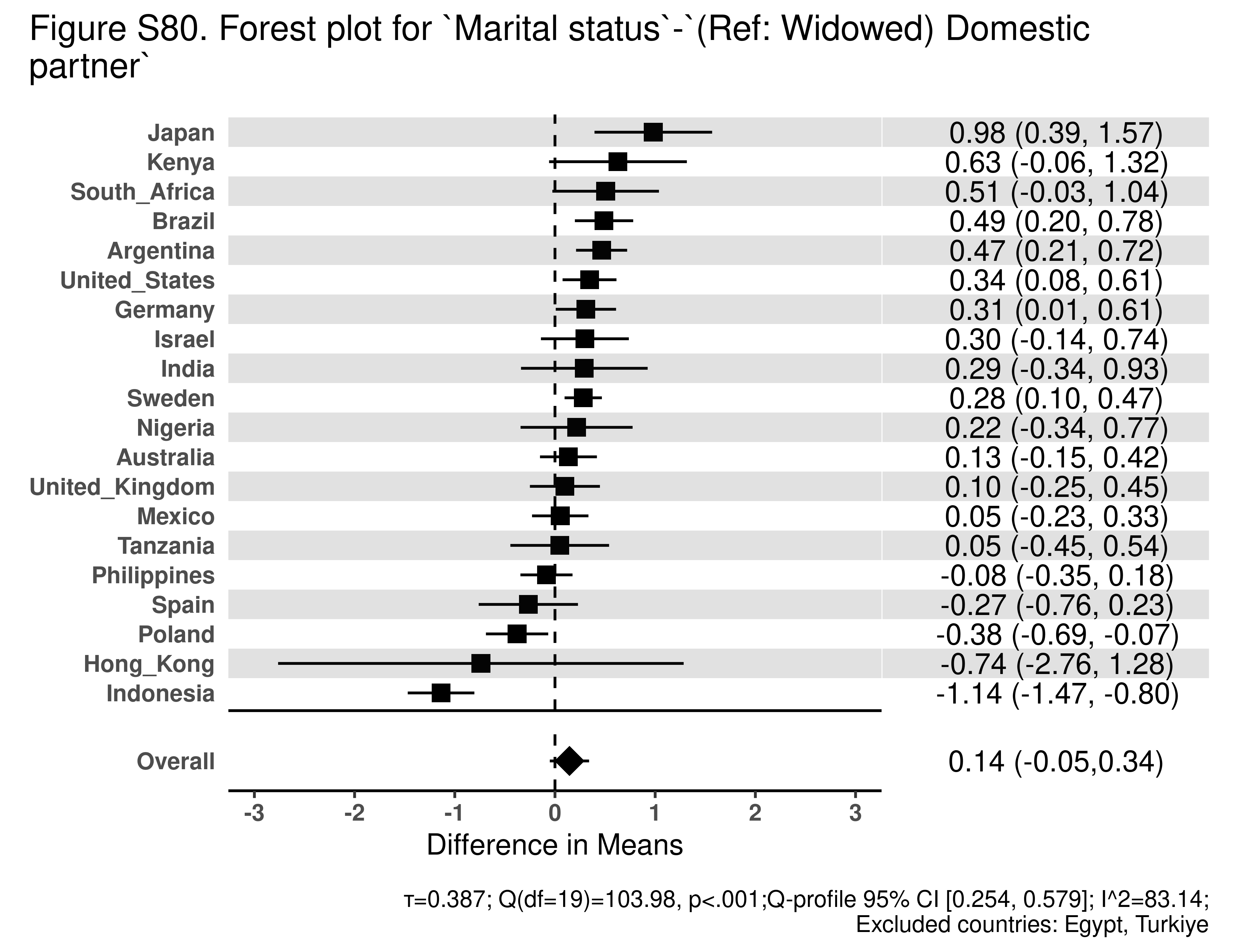


Figure S81. Forest plot for “Employment status: (Ref: Employed for an employer) Self-employed”


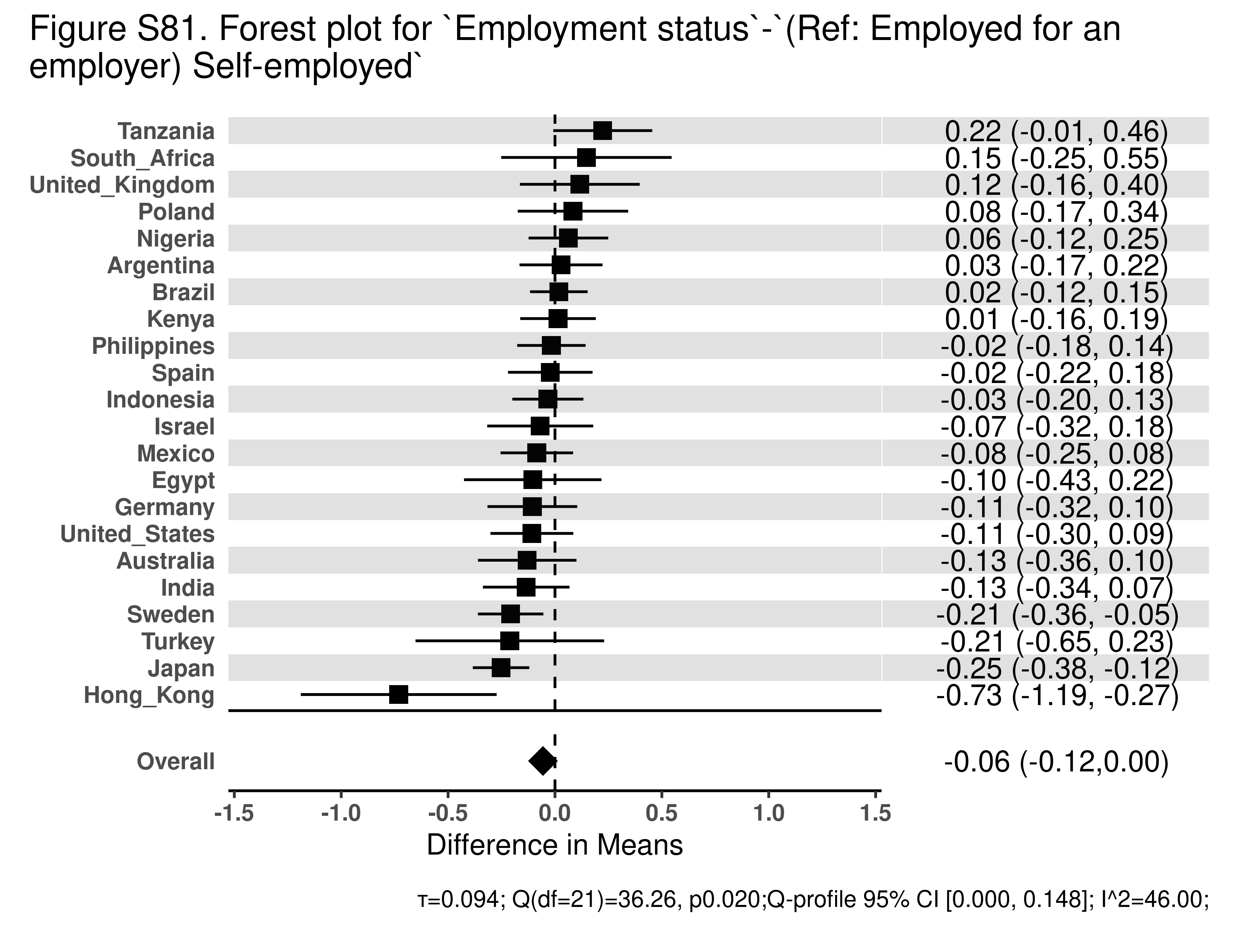


Figure S82. Forest plot for “Employment status: (Ref: Employed for an employer) Retired”


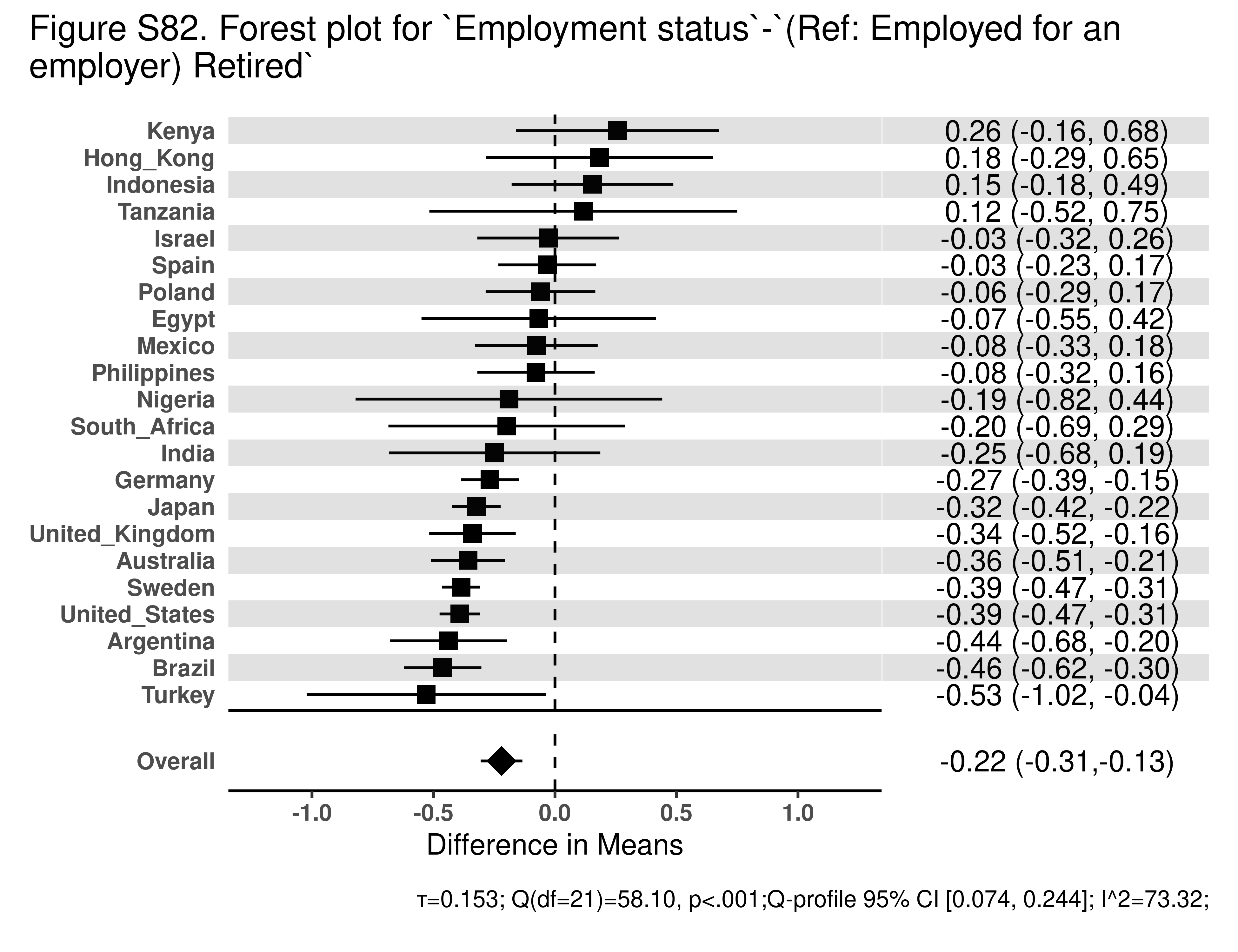


Figure S83. Forest plot for “Employment status: (Ref: Employed for an employer) Student”


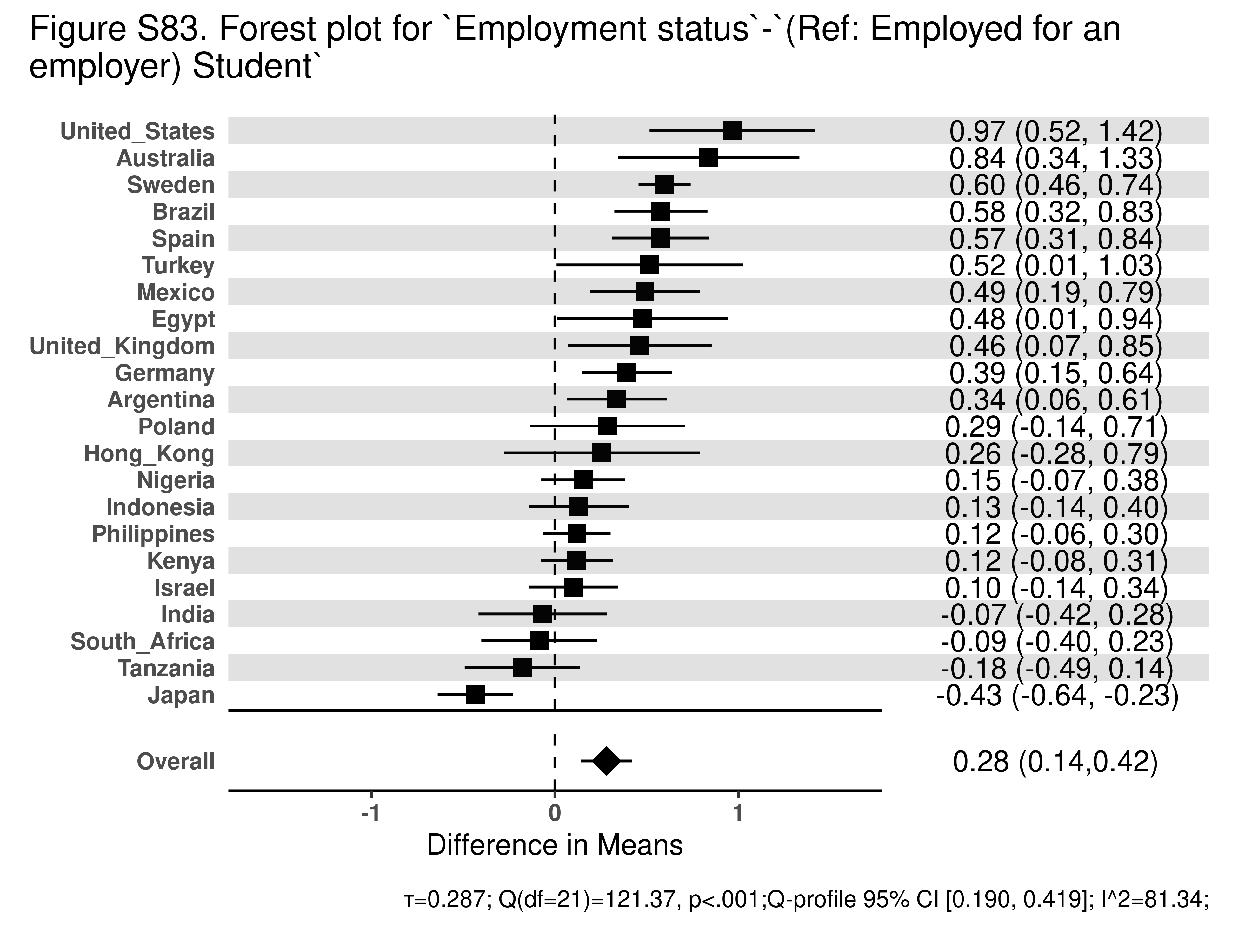


Figure S84. Forest plot for “Employment status: (Ref: Employed for an employer) Homemaker”


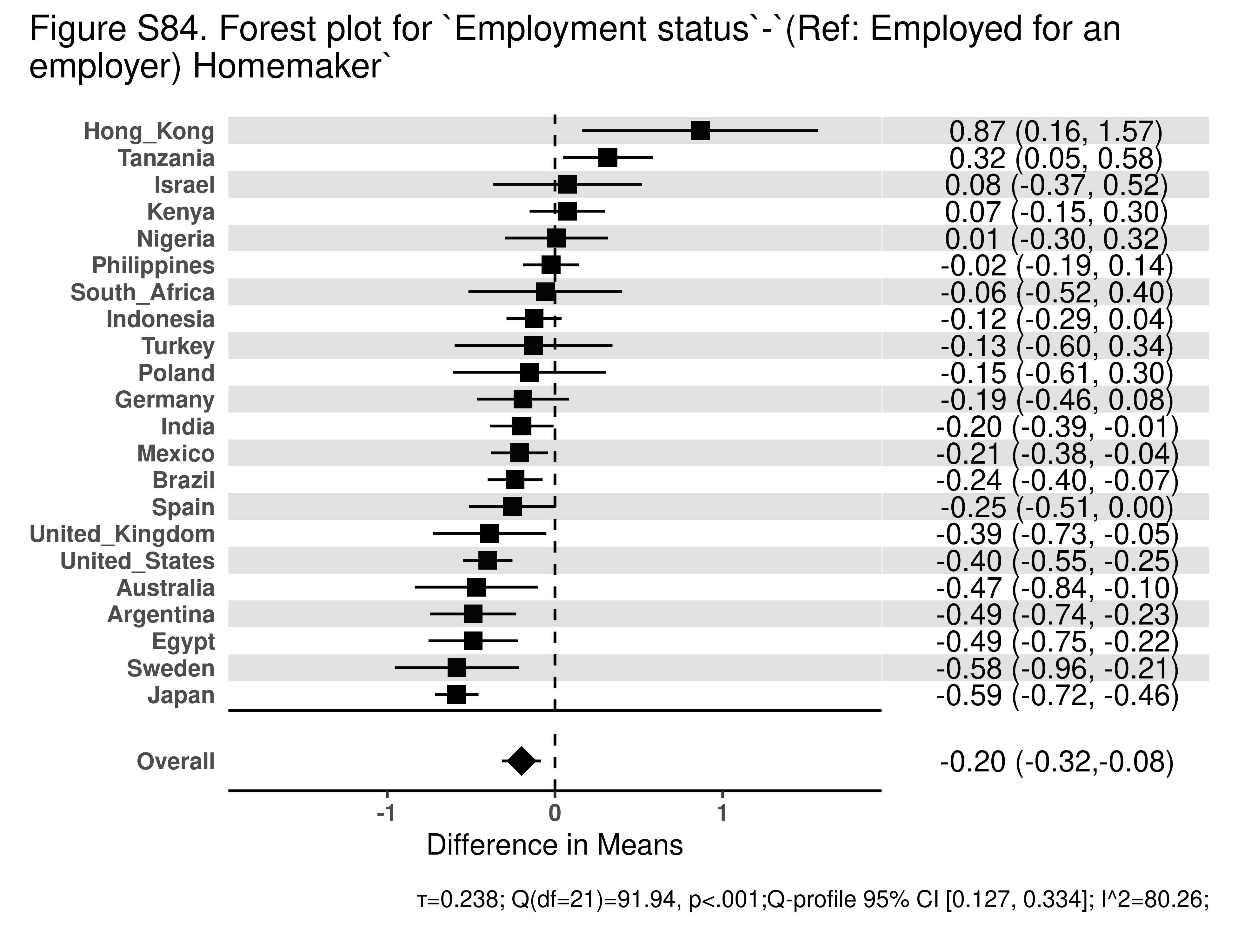


Figure S85. Forest plot for “Employment status: (Ref: Employed for an employer) Unemployed and looking for a job”


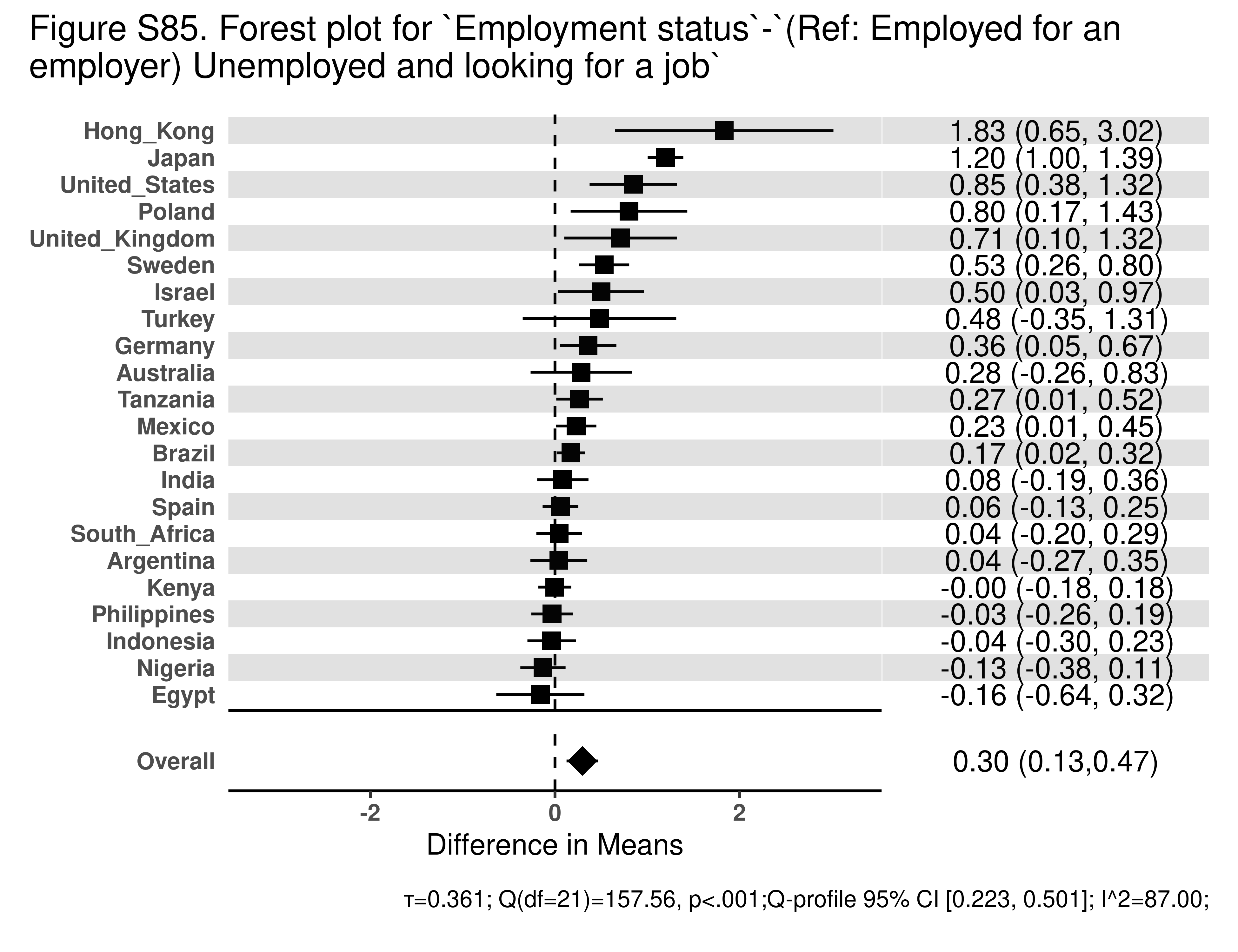


Figure S86. Forest plot for “Employment status: (Ref: Employed for an employer) None of these/other”


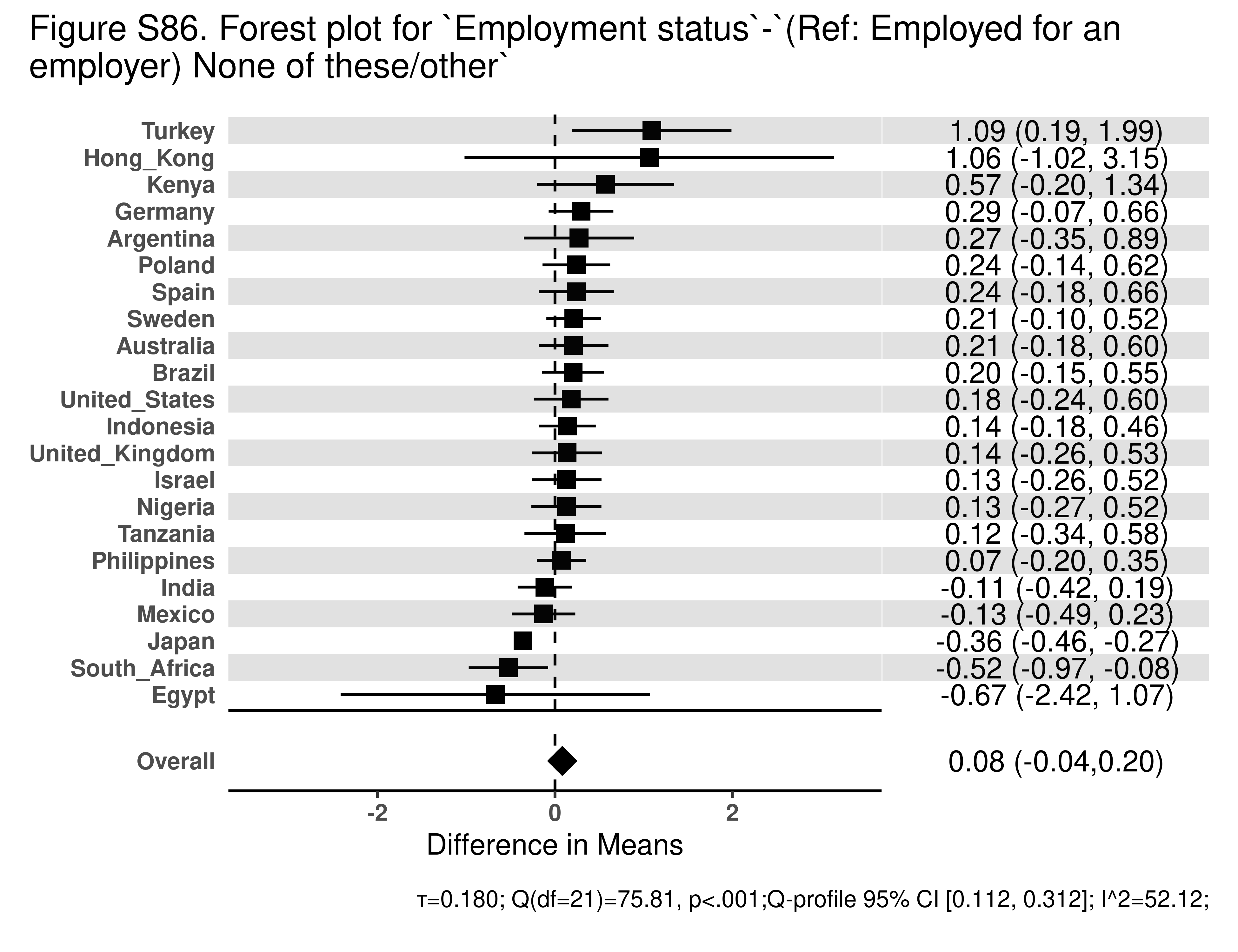


Figure S87. Forest plot for “Employment status: (Ref: Self-employed) Retired”


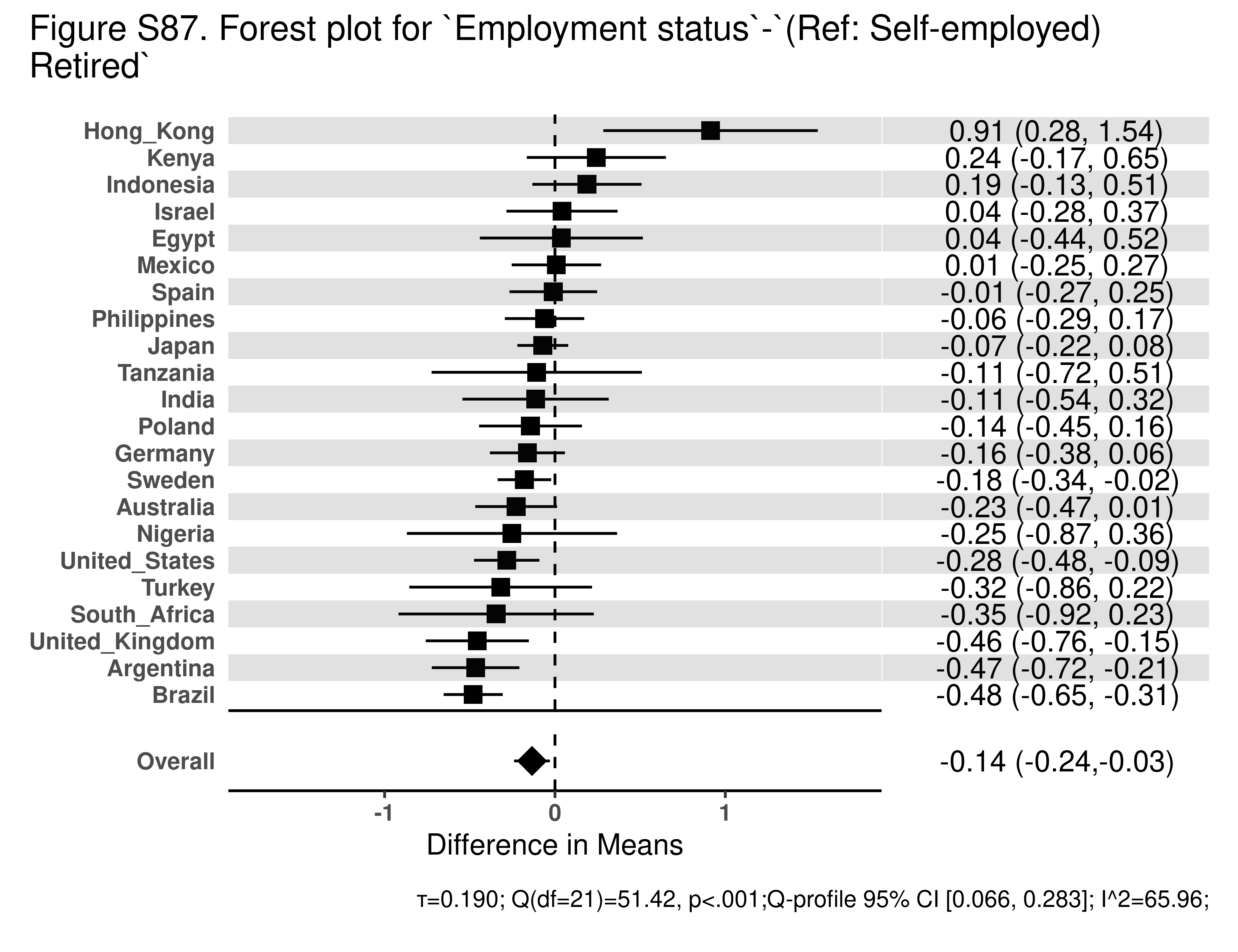


Figure S88. Forest plot for “Employment status: (Ref: Self-employed) Student”


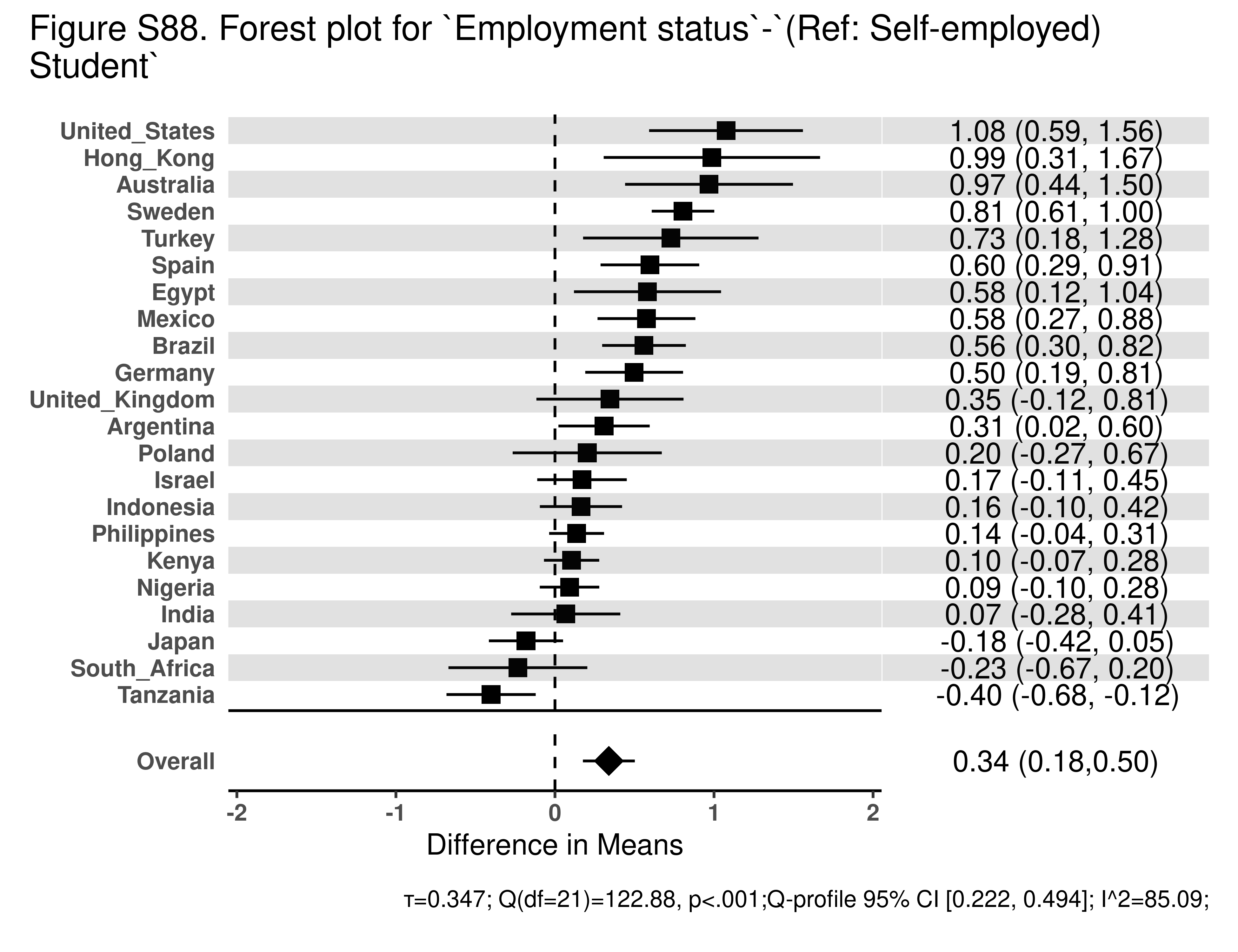


Figure S89. Forest plot for “Employment status: (Ref: Self-employed) Homemaker”


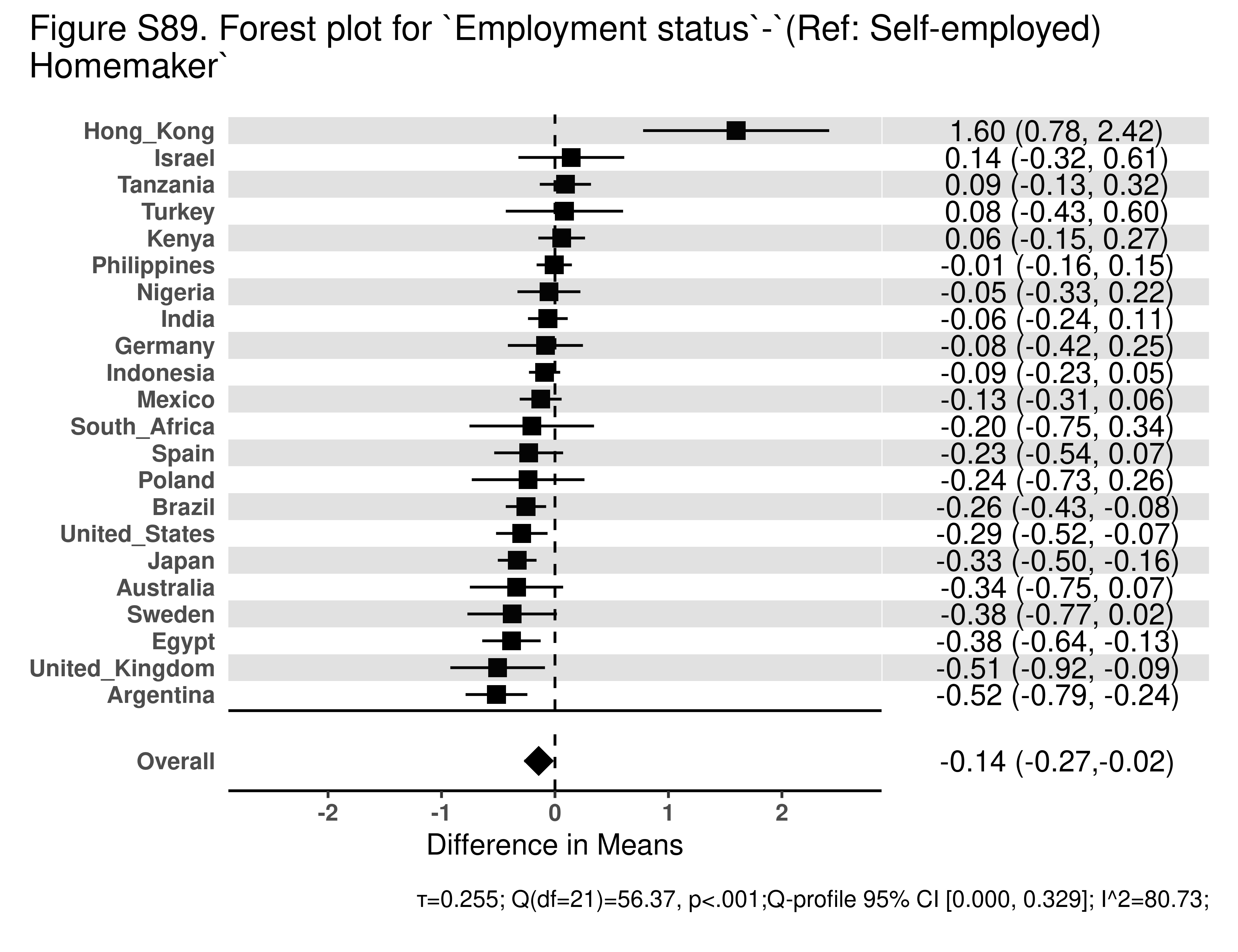


Figure S90. Forest plot for “Employment status: (Ref: Self-employed) Unemployed and looking for a job”


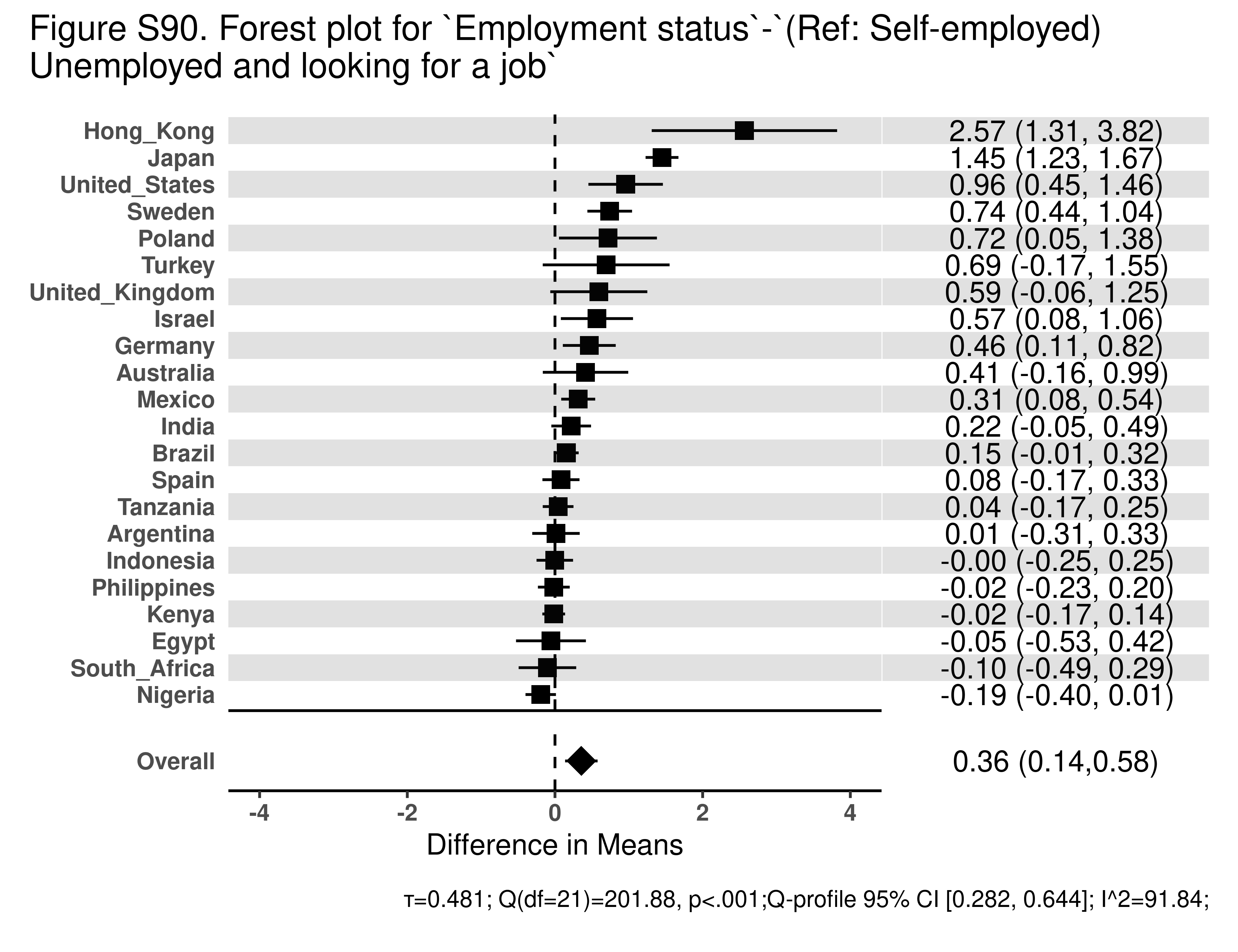


Figure S91. Forest plot for “Employment status: (Ref: Self-employed) None of these/other”


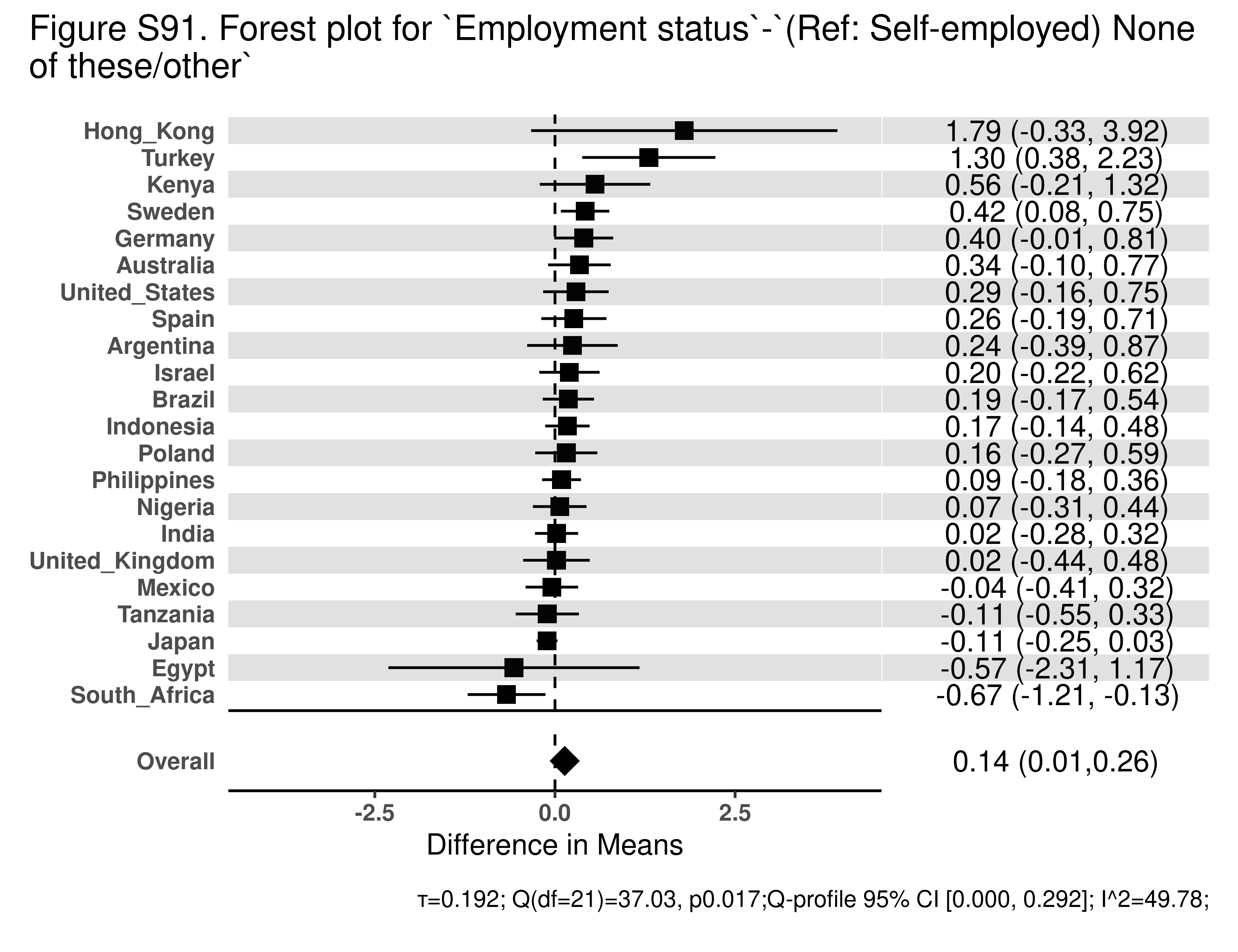


Figure S92. Forest plot for “Employment status: (Ref: Retired) Student”


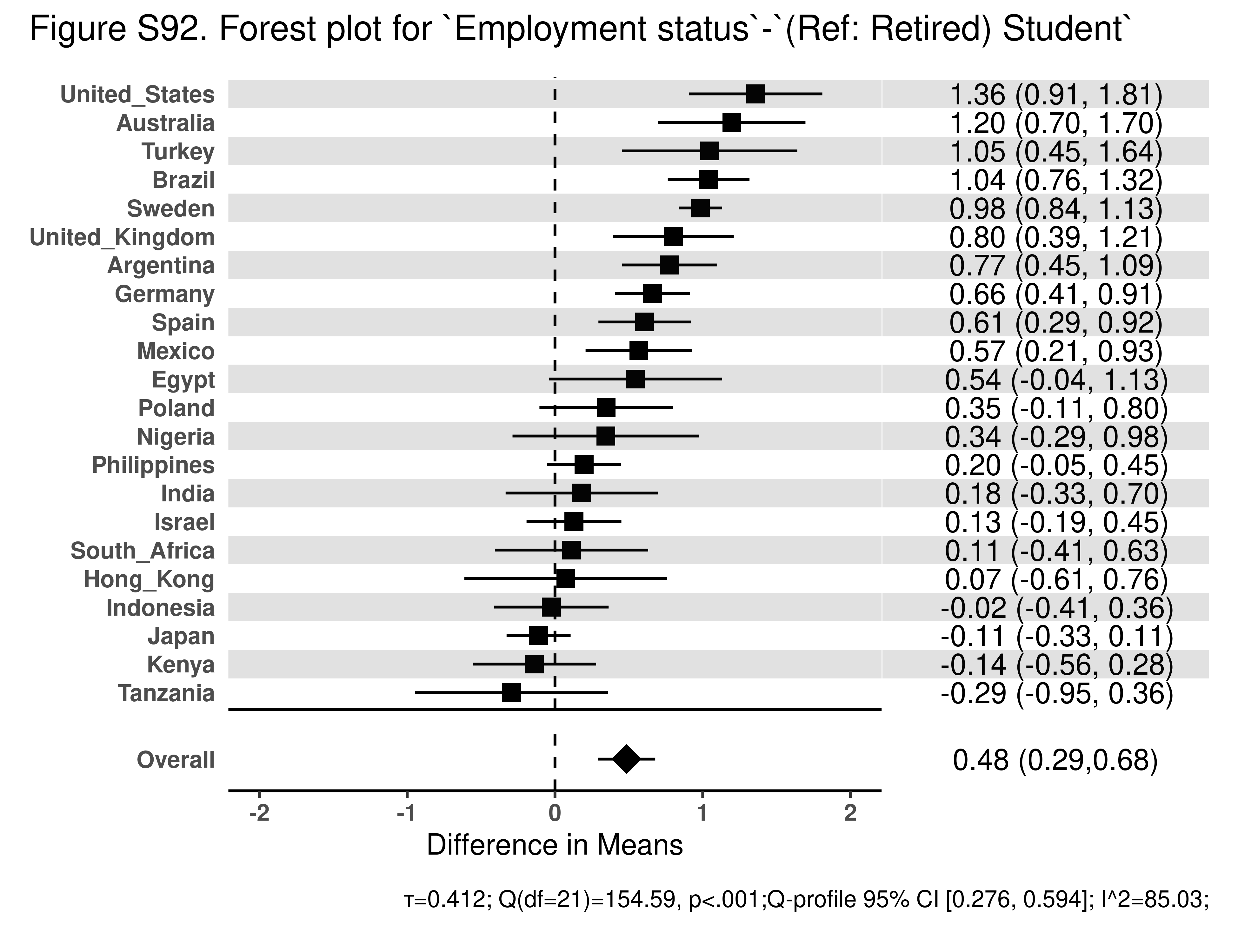


Figure S93. Forest plot for “Employment status: (Ref: Retired) Homemaker”


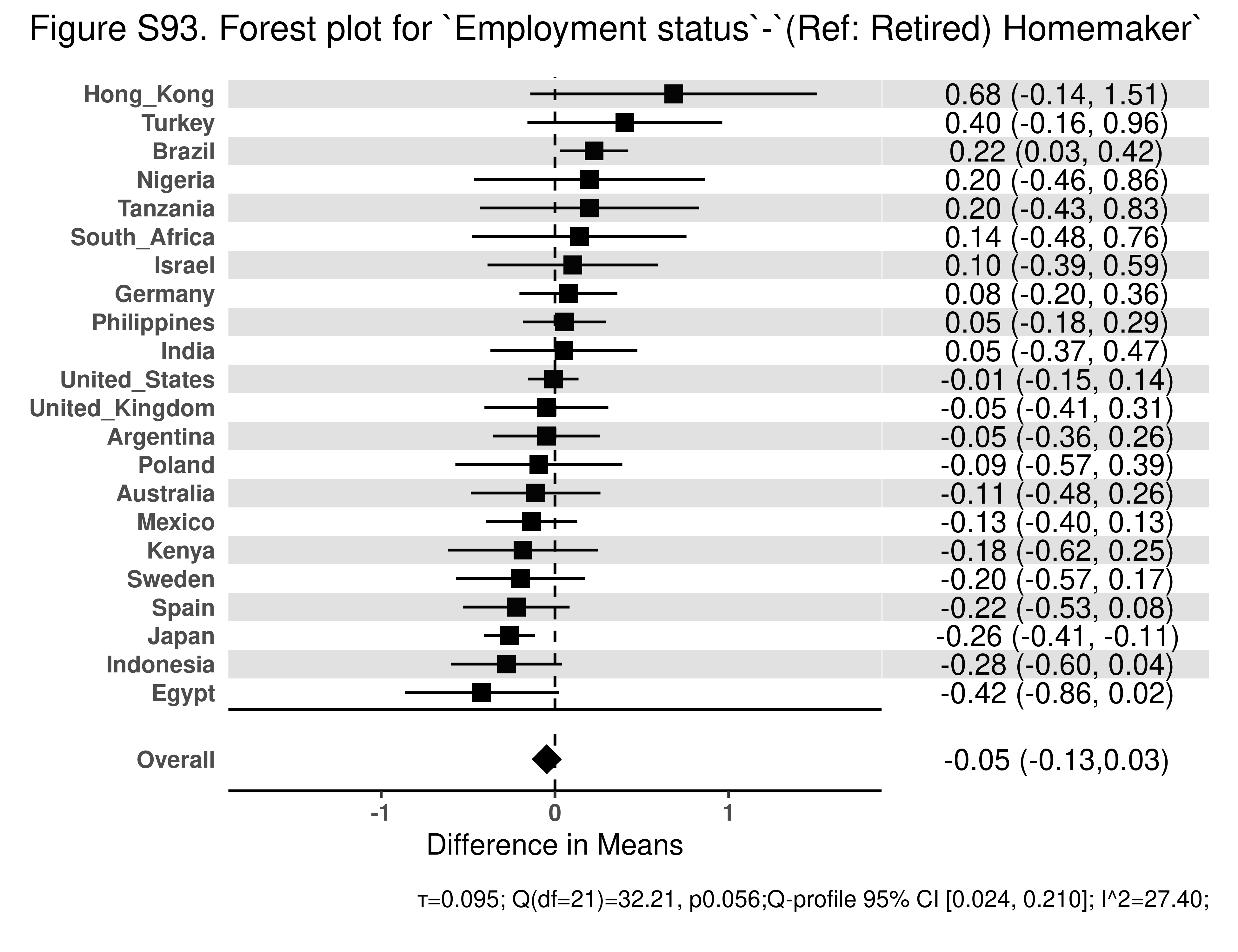


Figure S94. Forest plot for “Employment status: (Ref: Retired) Unemployed and looking for a job”


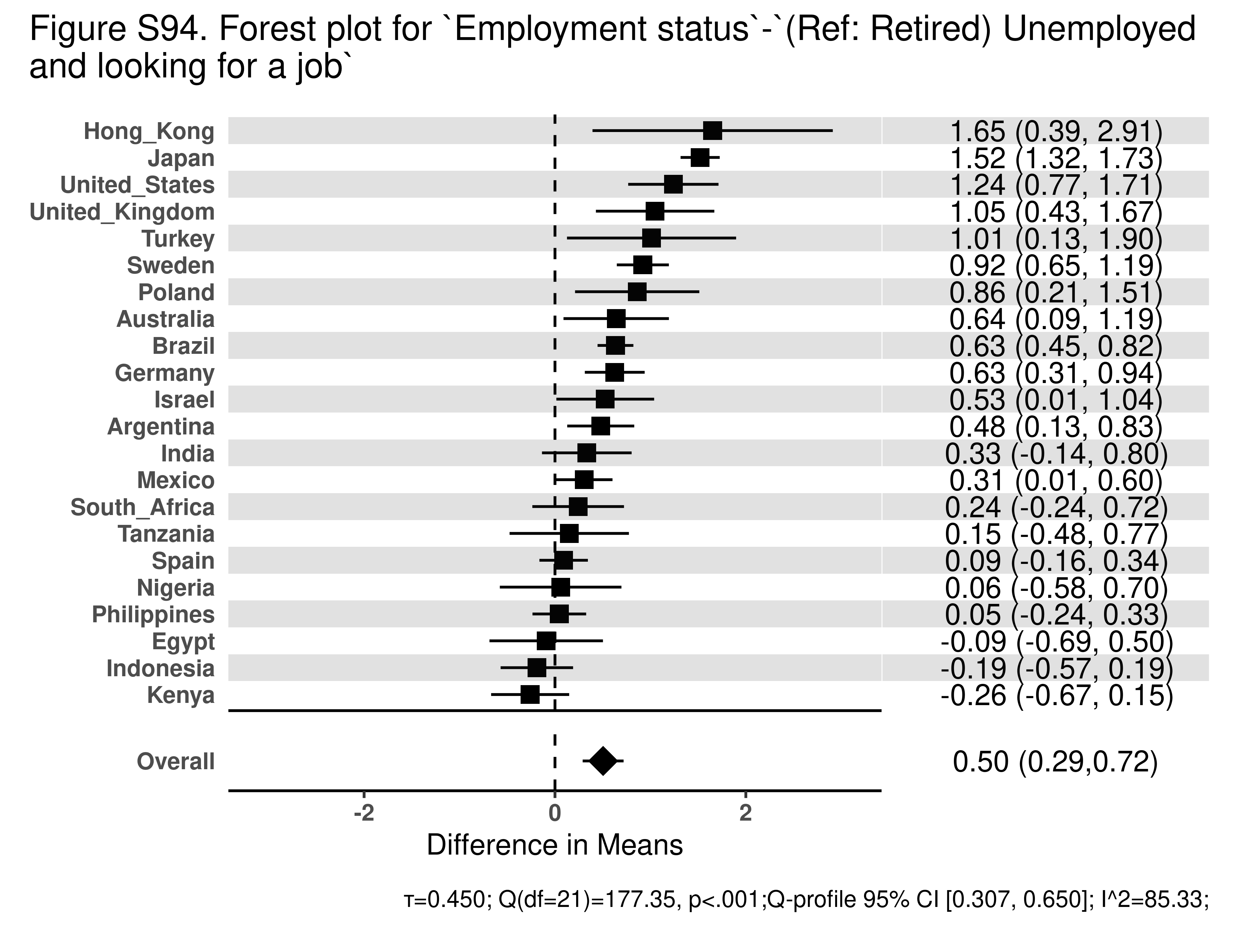


Figure S95. Forest plot for “Employment status: (Ref: Retired) None of these/other”


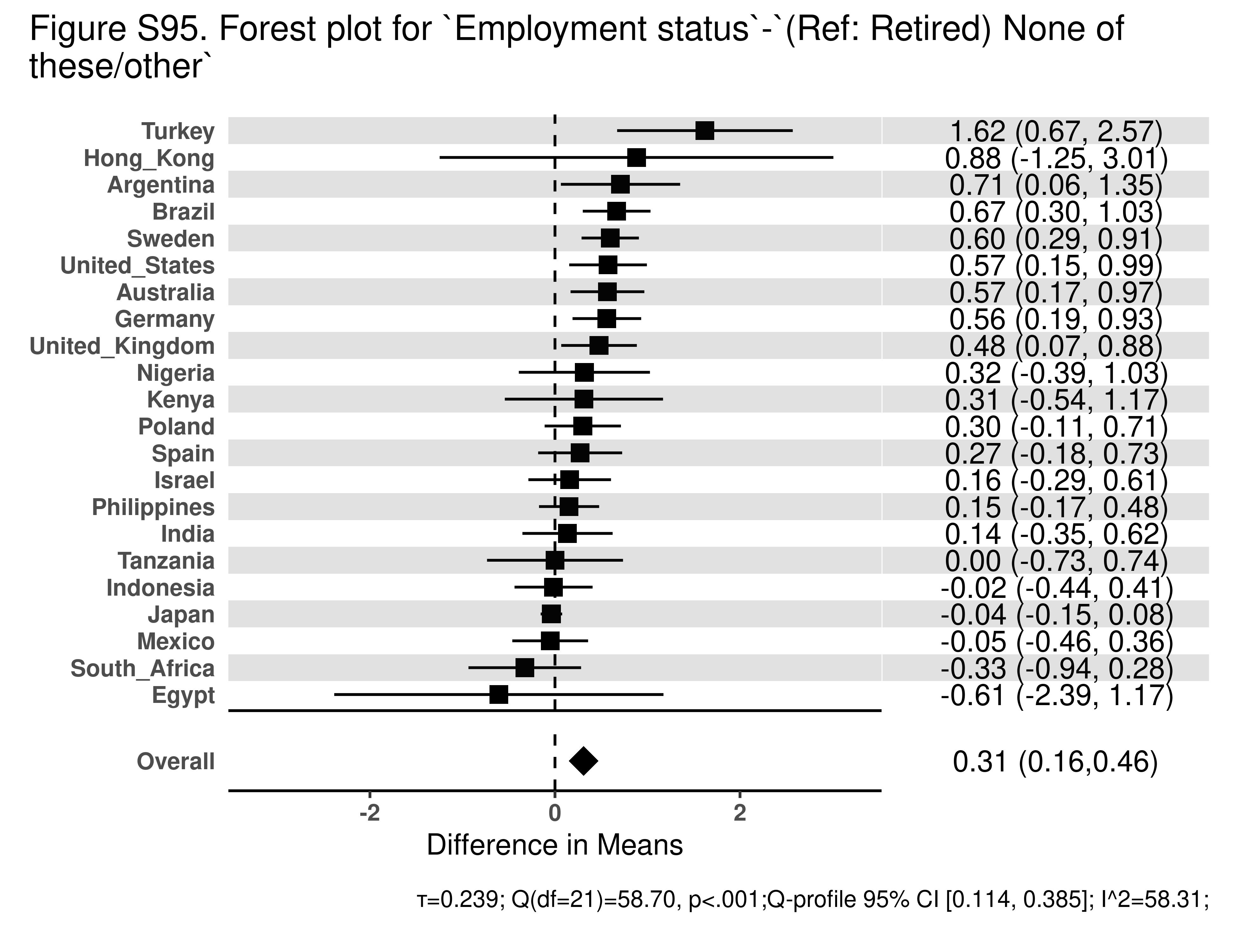


Figure S96. Forest plot for “Employment status: (Ref: Student) Homemaker”


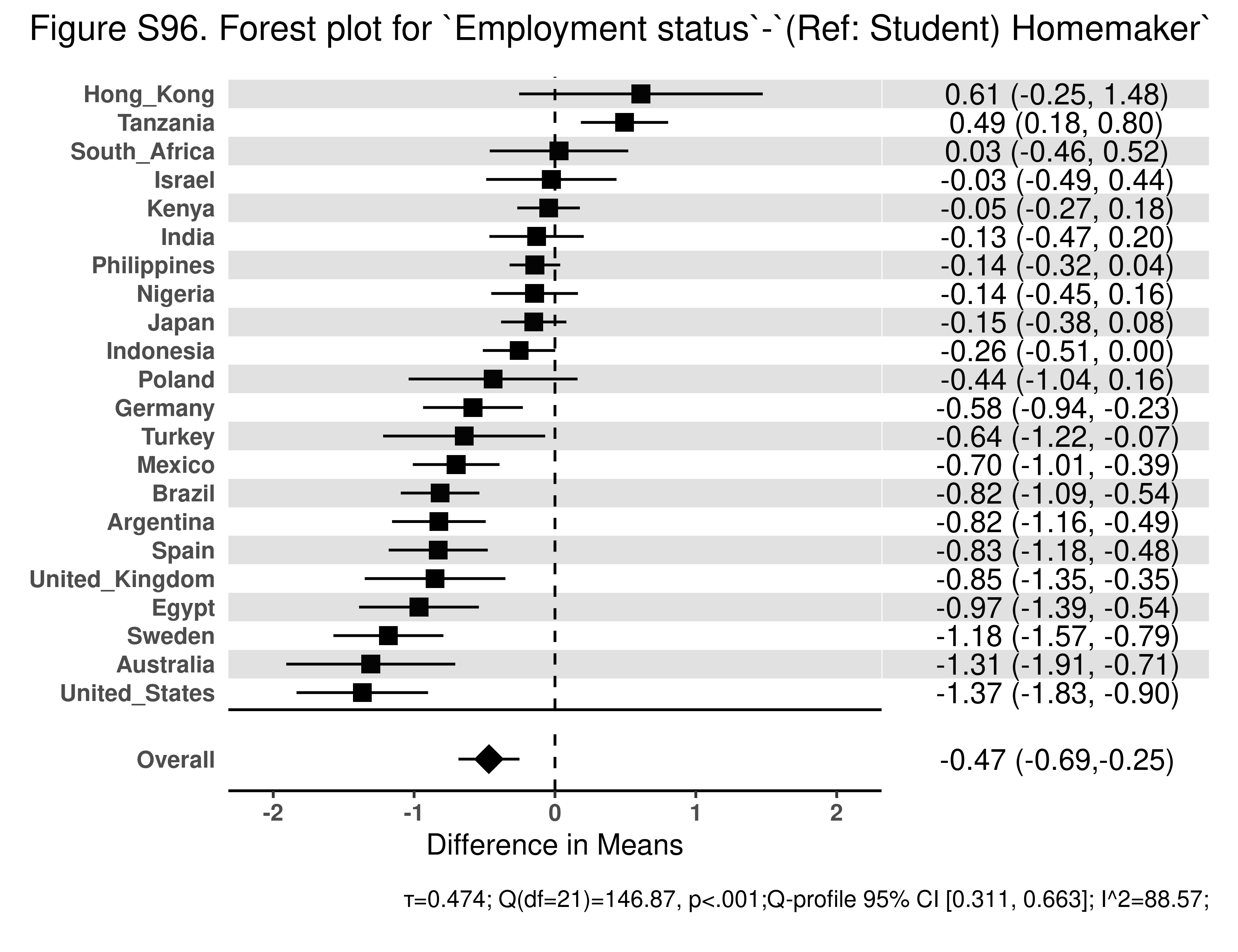


Figure S97. Forest plot for “Employment status: (Ref: Student) Unemployed and looking for a job”


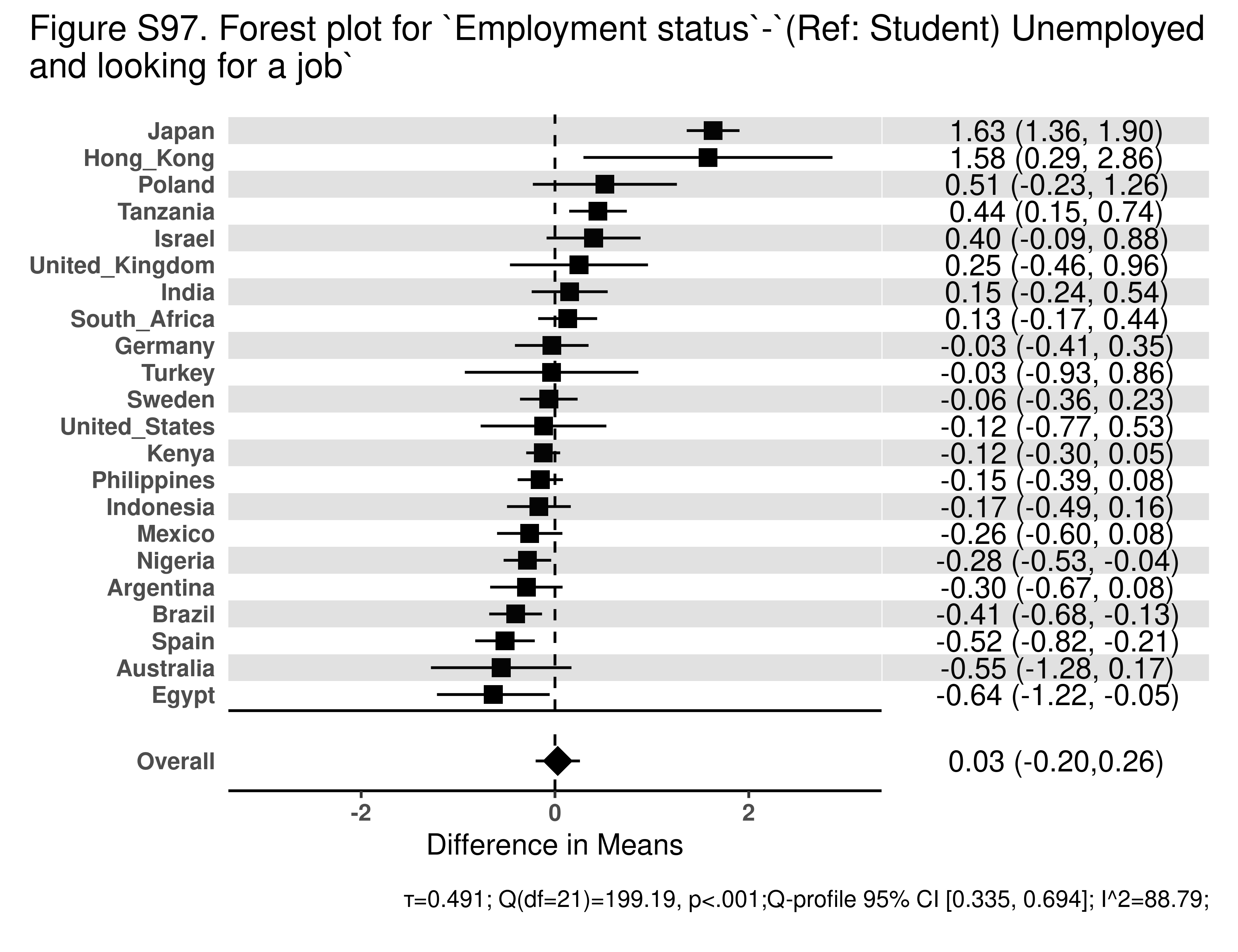


Figure S98. Forest plot for “Employment status: (Ref: Student) None of these/other”


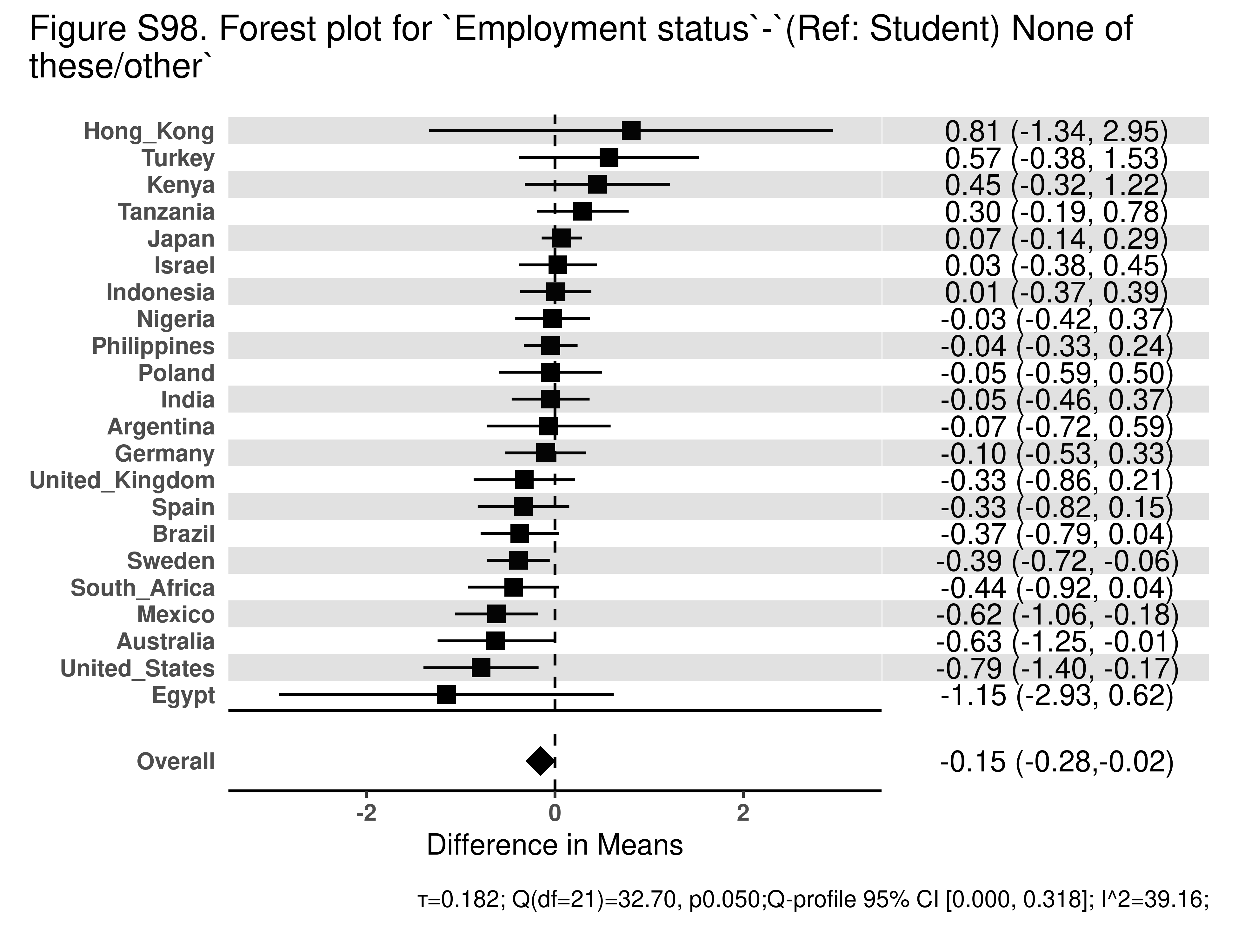


Figure S99. Forest plot for “Employment status: (Ref: Homemaker) Unemployed and looking for a job”


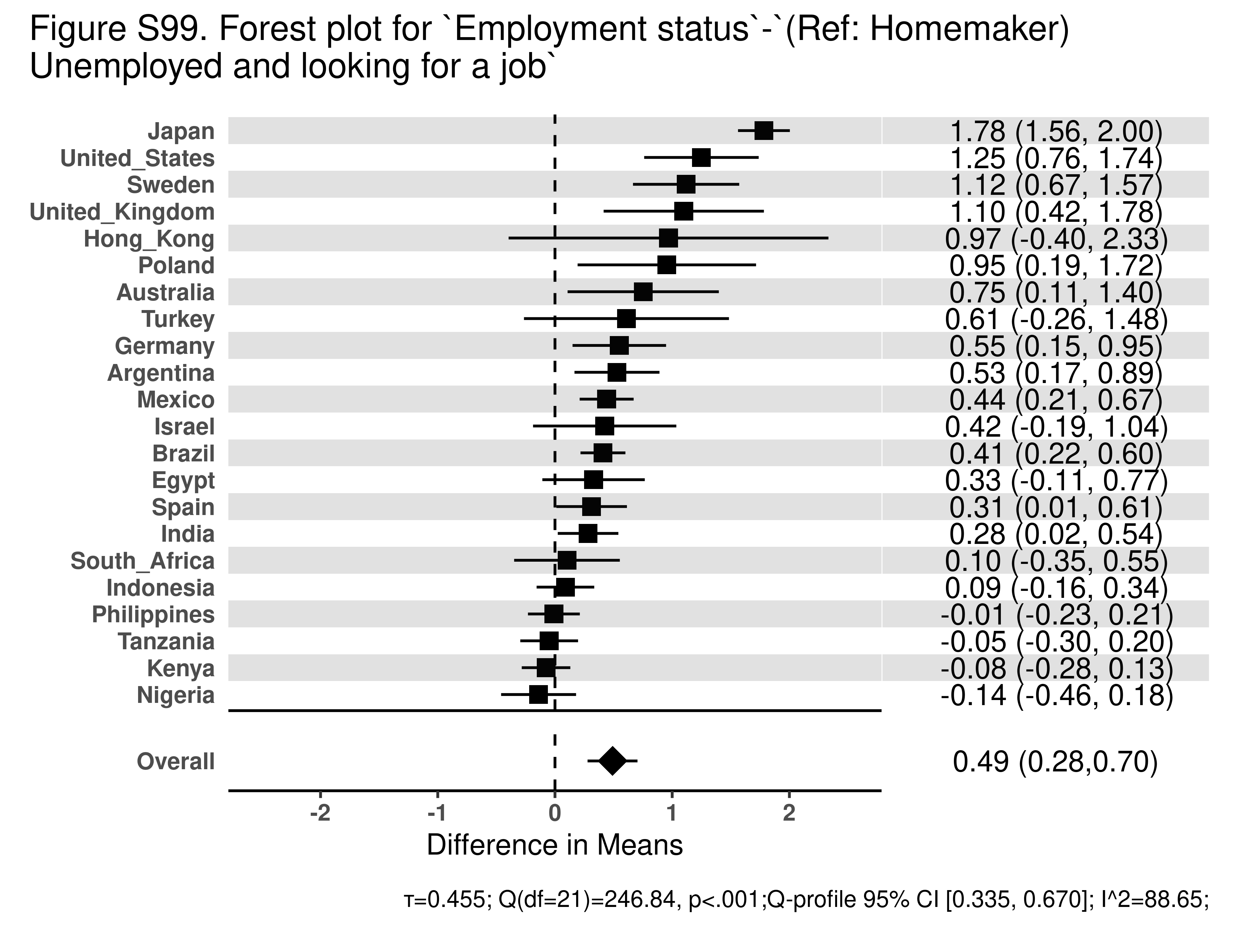


Figure S100. Forest plot for “Employment status: (Ref: Homemaker) None of these/other”


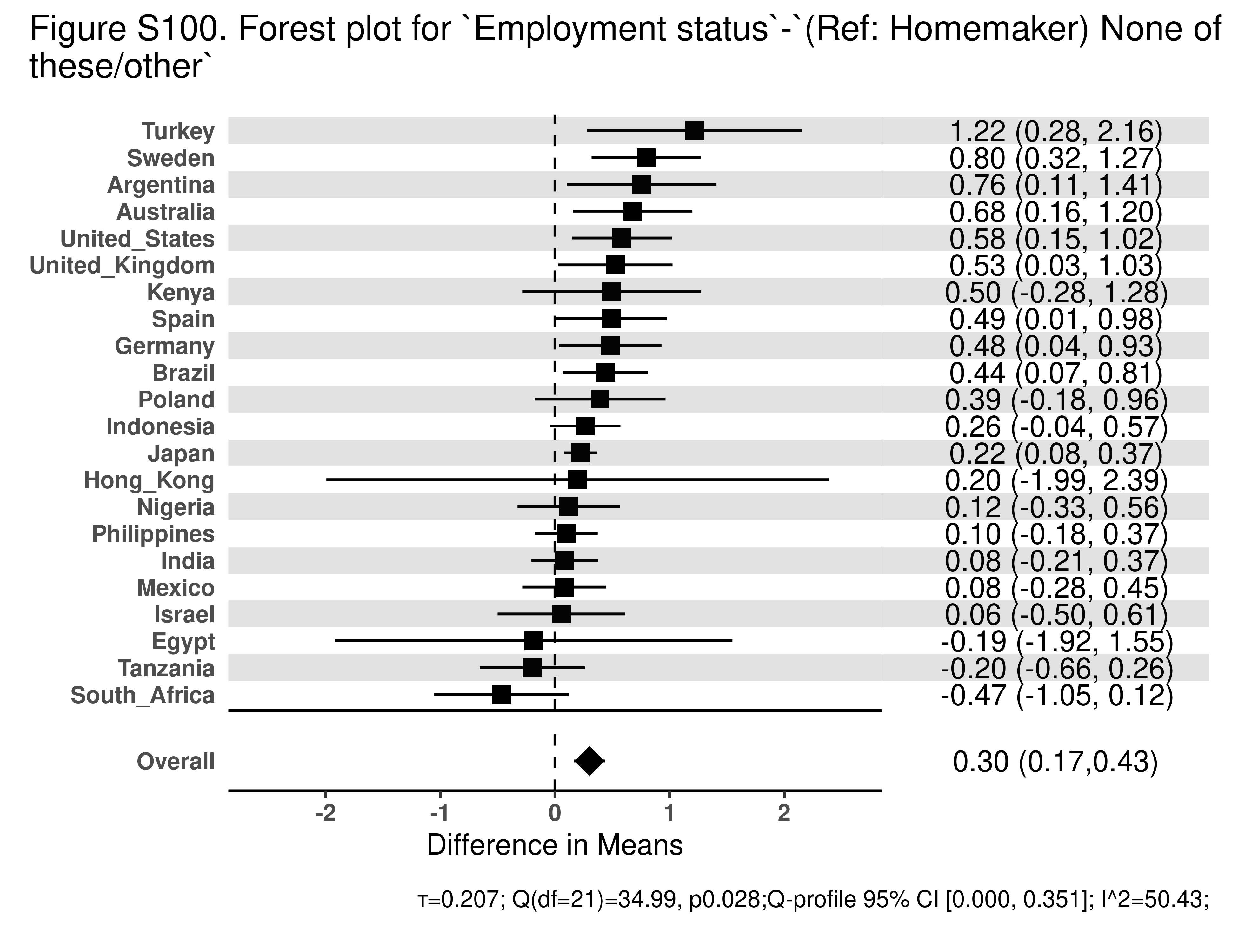


Figure S101. Forest plot for “Employment status: (Ref: Unemployed and looking for a job) None of these/other”

Figure S102. Forest plot for “Education: (Ref: Up to 8 years) 9-15 years”

Figure S103. Forest plot for “Education: (Ref: Up to 8 years) 16+ years”

Figure S104. Forest plot for “Education: (Ref: 9-15 years) 16+ years”

Figure S105. Forest plot for “Religious service attendance: (Ref: >1x/week) 1x/week”

Figure S106. Forest plot for “Religious service attendance: (Ref: >1x/week) 1-3x/month”

Figure S107. Forest plot for “Religious service attendance: (Ref: >1x/week) A few times a year”

Figure S108. Forest plot for “Religious service attendance: (Ref: >1x/week) Never”

Figure S109. Forest plot for “Religious service attendance: (Ref: 1x/week) 1-3x/month”

Figure S110. Forest plot for “Religious service attendance: (Ref: 1x/week) A few times a year”

Figure S111. Forest plot for “Religious service attendance: (Ref: 1x/week) Never”

Figure S112. Forest plot for “Religious service attendance: (Ref: 1-3x/month) A few times a year”

Figure S113. Forest plot for “Religious service attendance: (Ref: 1-3x/month) Never”

Figure S114. Forest plot for “Religious service attendance: (Ref: A few times a year) Never”

Figure S115. Forest plot for “Immigration status: (Ref: Born in this country) Born in another country”

Figure S116. Forest plot for relationship with mother: very/somewhat good

(Ref: Very bad/somewhat bad)

Figure S117. Forest plot for relationship with father: very/somewhat good

(Ref: Very bad/somewhat bad)

Figure S118. Forest plot for parent marital status: divorced

(Ref: Parents married)

Figure S119. Forest plot for parent marital status: never married

(Ref: Parents married)

Figure S110. Forest plot for parent marital status: one or both parents had died

(Ref: Parents married)

Figure S111. Forest plot for subjective financial status of family growing up: lived comfortably

(Ref: Got by)

Figure S112. Forest plot for subjective financial status of family growing up: found it difficult

(Ref: Got by)

Figure S113. Forest plot for subjective financial status of family growing up: found it very difficult

(Ref: Got by)

Figure S114. Forest plot for abuse: yes

(Ref: No)

Figure S115. Forest plot for outsider growing up: yes

(Ref: No)

Figure S116. Forest plot for self-rated health growing up: excellent

(Ref: Good)

Figure S117. Forest plot for self-rated health growing up: very good

(Ref: Good)

Figure S118. Forest plot for self-rated health growing up: fair

(Ref: Good)

Figure S119. Forest plot for self-rated health growing up: poor

(Ref: Good)

Figure S120. Forest plot for immigration status (adjusting for childhood variables): born in another country

(Ref: Born in this country)

Figure S121. Forest plot for age 12 religious service attendance: at least 1x/week

(Ref: Never)

Figure S122. Forest plot for age 12 religious service attendance: 1-3x/month

(Ref: Never)

Figure S123. Forest plot for age 12 religious service attendance: less than 1x/month

(Ref: Never)

Figure S124. Forest plot for gender (adjusting for childhood variables): female

(Ref: Male)

Figure S125. Forest plot for gender (adjusting for childhood variables): other

(Ref: Male)

Figure S126. Forest plot for year of birth: 1993-1998, age 25-29

(Ref: 1998-2005, age 18-24)

Figure S127. Forest plot for year of birth: 1983-1993, age 30-39

(Ref: 1998-2005, age 18-24)

Figure S128. Forest plot for year of birth: 1973-1983, age 40-49

(Ref: 1998-2005, age 18-24)

Figure S129. Forest plot for year of birth: 1963-1973, age 50-59

(Ref: 1998-2005, age 18-24)

Figure S130. Forest plot for year of birth: 1953-1963, age 60-69

(Ref: 1998-2005, age 18-24)

Figure S131. Forest plot for year of birth: 1943-1953, age 70-79

(Ref: 1998-2005, age 18-24)

Figure S132. Forest plot for year of birth: 1943 or earlier, age 80 or older

(Ref: 1998-2005, age 18-24)
